# A Versatile Route to Unstable Diazo Compounds via Oxadiazolines and their Use in Aryl–Alkyl Cross‐Coupling Reactions

**DOI:** 10.1002/anie.201710445

**Published:** 2017-12-05

**Authors:** Andreas Greb, Jian‐Siang Poh, Stephanie Greed, Claudio Battilocchio, Patrick Pasau, David C. Blakemore, Steven V. Ley

**Affiliations:** ^1^ Department of Chemistry University of Cambridge Lensfield Road Cambridge CB2 1EW UK; ^2^ UCB Biopharma SPRL Chemical Research R5 Chemin du Foriest, 1420 Braine-L'Alleud Belgium; ^3^ Medicine Design Pfizer Inc. Eastern Point Road Groton CT 06340 USA

**Keywords:** boronic acids, cross-coupling, diazo compounds, flow chemistry, photochemistry

## Abstract

Coupling of readily available boronic acids and diazo compounds has emerged recently as a powerful metal‐free carbon–carbon bond forming method. However, the difficulty in forming the unstable diazo compound partner in a mild fashion has hitherto limited their general use and the scope of the transformation. Here, we report the application of oxadiazolines as precursors for the generation of an unstable family of diazo compounds using flow UV photolysis and their first use in divergent protodeboronative and oxidative C(sp^2^)−C(sp^3^) cross‐coupling processes, with excellent functional‐group tolerance.

Diazo compounds represent a highly useful class of compounds in organic synthesis,^1^ for example in cyclopropanation,^2^ and heteroatom−H^3^ and C−H insertion^4^ reactions. In particular, the reaction of diazo compounds with organoboron species has attracted considerable interest over the past few years, such as in C(sp^2^)−C(sp^3^) cross‐coupling reactions.^5^

Our previous studies on the flow oxidation of hydrazones,^6^ showed the effectiveness of utilizing mild conditions to generate and react diazo compounds with boronic acid. In particular, this allowed us to intercept the putative unstable boronic species and thereby enable access to alcohols^6^ and powerful iterative bond‐forming processes.^7^ Nevertheless, these studies were limited to the generation of “semi‐stabilized diazo compounds”, bearing either an aryl or vinyl group adjacent to the diazo moiety. To truly generalize this concept would require expansion into the elusive realm of “non‐stabilized diazo compounds”, a class of compounds notorious for their intrinsic instability, toxic/hazardous nature, and difficulty of preparation (Scheme 1).1b Within this class of reactive intermediates, dialkyl‐substituted diazo compounds pose a major challenge to both access and safely utilize these reactive species. If a mild method to generate these non‐stabilized diazo compounds could therefore be realized, a more general method to enable aryl–alkyl cross‐coupling may become possible, along with potential interception of the intermediate boron species to afford new reactions.

**Scheme 1:**
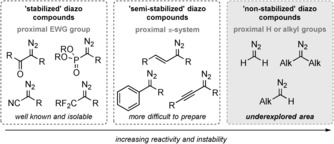
Families of diazo compounds.

While a multitude of methods have been developed to access diazo compounds, few allow the generation of the non‐stabilized members of the family with sufficient generality. An interesting report by Warkentin et al. in 1989 showed that UV photolysis of 1,3,4‐oxadiazolines at around 300 nm led to diazoalkanes.^8^ Surprisingly, this approach has been largely overlooked by chemists as a potential route to forming unstable diazo compounds, and has never before been engaged in the development of new synthetic methods. Oxadiazolines are available by a two‐step, one‐pot procedure from readily available ketones through condensation of acetic hydrazide and subsequent PhI(OAc)_2_‐mediated oxidation. In contrast to alternative diazo precursors such as hydrazones and nitrosoamides, oxadiazolines were found to be bench‐stable over many months (also see Supporting Information for differential scanning calorimetry data).

At the outset of our investigation, we decided to exploit enabling flow technologies to achieve more efficient irradiation compared with a batch reactor,^9^ and to avoid the build‐up of hazardous quantities of any unstable diazo compounds.^10^, ^11^, ^12^ Initial studies began with the generation of oxadiazoline **1**, a potential precursor for the cyclic, non‐stabilized diazo compound, 4‐diazotetrahydropyran (**2**; Scheme 2). Passage of an ethereal solution of **1** through a 10 mL reactor coil held at 10 °C and irradiated at 310 nm with a 9 W UV lamp, at a 0.125 mL min^−1^ flow rate (residence time=80 min) led to the generation of a distinctively red solution. In‐line IR spectroscopic analysis revealed two important features: a peak at 2040 cm^−1^ corresponding to the stretch of a diazo group confirmed the presence of diazo compound **2**; in addition, a peak at 1746 cm^−1^ corresponding to the C=O stretch of methyl acetate was observed, a useful gauge of assessing photolytic conversion of the oxadiazolines. Consequently, we could now efficiently access non‐stabilized diazo compounds using a flow method and then study their use in C(sp^2^)−C(sp^3^) cross‐coupling (see the Supporting Information for optimization results). This procedure is divergent depending upon the “workup” conditions of the intermediate boron species.

**Scheme 2:**
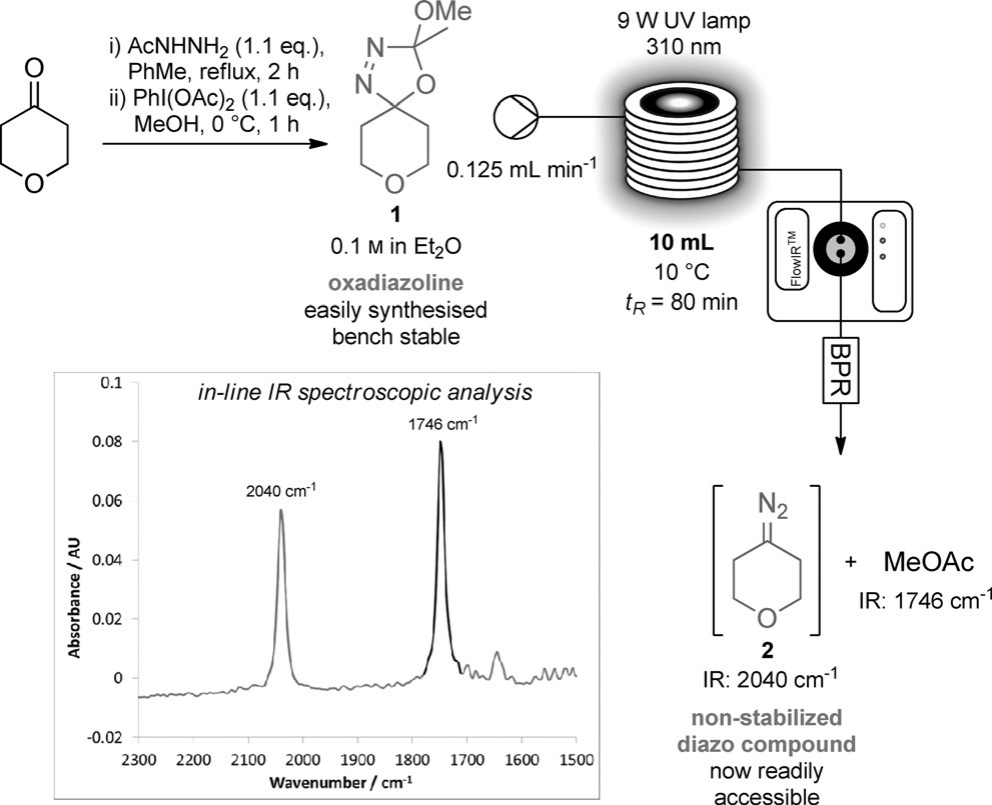
Oxadiazolines as diazo precursors and access to non‐stabilized diazo compound **2**.

Using a number of oxadiazolines and readily available boronic acids (forming boroxines in situ),^13^ we were able to demonstrate a remarkably broad reaction scope and unusually high functional‐group compatibility (Table 1). With respect to the oxadiazoline component, a variety of pharmaceutically relevant 4‐, 5‐ and 6‐membered saturated heterocyclic examples were viable coupling partners, including pyran (**3 a**), tetrahydrofuran (**3 b**), tetrahydrothiopyran (**3 c**), tetrahydrothiophene (**3 d**), thietane (**3 e**), *N*‐Boc piperidine (**3 f**), *N*‐Boc pyrrolidine (**3 g**), and *N*‐Boc azetidine (**3 h**) rings, providing moderate to excellent yields of the desired C(sp^2^)−C(sp^3^) cross‐coupling products. It is particularly notable that the 4‐membered rings were viable examples given the instability of 4‐membered cyclic diazo compounds,^14^ which arises from the relief of ring strain when moving from an sp^2^ to an sp^3^ carbon center on reaction with electrophiles. Furthermore, tolerance of the tetrahydrothiophene and thietane moieties highlights examples where approaches using a tosylhydrazone route or carbon‐centered radical approaches would fail, due to the tendency of these systems to undergo elimination/ring‐opening. A number of carbocyclic examples containing cycloalkyl groups (**3 i**–**3 l**) were also permissible substrates, along with a highly hindered adamantane substituent (**3 m**). Tolerance of a cyclopropyl group (**3 n**) is not only indicative of a non‐radical based process for this C(sp^2^)−C(sp^3^) cross‐coupling, but also further exemplifies the advantages of this procedure over methods utilizing carbon‐centered radicals (where cyclopropane ring‐opened products would be obtained instead). In terms of functional‐group compatibility for the oxadiazoline component, olefins (**3 o**), alkynes (**3 p**), acetals (**3 q**), phosphonates (**3 r**), sulfones (**3 s**), furans (**3 v**), and pyrimidines (**3 w**) were all viable substrates. Remarkably, both an epoxide (**3 t**) and an alkyl bromide (**3 u**) could participate in this coupling process, products that would be intractable to access using metal‐catalyzed methods or harsh basic conditions. With respect to the boronic acid component, a variety of electron‐deficient aromatic rings harboring various functional groups were suitable substrates, including those with 4‐bromo (**3 x**), 4‐trifluoromethyl (**3 y**), 4‐cyano (**3 z**), and 4‐methoxycarbonyl (**3 aa**) substituents. Electron‐rich groups were also tolerated, including 3‐acetamide (**3 ab**), 4‐methoxy (**3 ac**), and *o*‐methyl (**3 ad**) substituents, although for the two latter cases, a higher temperature was required to achieve protodeboronation. Although lower‐yielding, heterocyclic boronic acids could also be employed in this method to provide useful amounts of desired cross‐coupled product, for example, 3‐pyridyl (**3 ae**) and 2‐thienyl (**3 af**) substituents.

**Table 1:** Metal‐free protodeboronative and oxidative C(sp^2^)−C(sp^3^) cross‐coupling. 
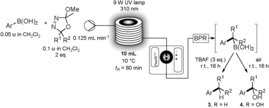

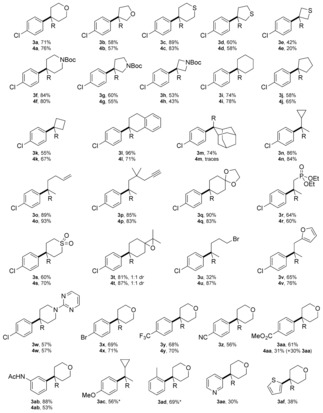

Yields stated are of isolated product. Protodeboronation: 0.5 mmol of boronic acid, 1.0 mmol of oxadiazoline and 1.0 mmol of DIPEA, then workup using TBAF (3 equiv.) and stirred further at RT for 16 h. * protodeboronation was conducted at 75 °C for 16 h. Oxidation: 0.5 mmol of boronic acid, 1.0 mmol of oxadiazoline and 1.0 mmol of DIPEA, then workup by stirring further under air at RT for 16 h.

A simple switch in the workup procedure to stirring under air provided the oxidized C(sp^2^)−C(sp^3^) cross‐coupled products, and this reaction exhibited a similar broad scope and high functional‐group compatibility (Table 1). Again, a variety of 4‐, 5‐ and 6‐membered saturated heterocyclic oxadiazolines could be coupled with 4‐chlorophenylboronic acid to generate a variety of tertiary alcohols (**4 a**–**4 h**) in generally similar yields to the protodeboronative method. Similar yields were obtained for carbocyclic examples (**4 i**–**4 l**, **4 n**), with the exception of adamantane derivative **4 m**, where oxidation appeared to be extremely slow. In terms of functional‐group compatibility, olefins (**4 o**), alkynes (**4 p**), acetals (**4 q**), phosphonates (**4 r**), sulfones (**4 s**), furans (**4 v**) and pyrimidines (**4 w**) all proceeded smoothly. A particular highlight was the tolerance of highly reactive functionalities such as an epoxide (**4 t**) and alkyl bromide (**4 u**). Electron‐poor boronic acids (**4 x**, **4 y**) and electron‐rich boronic acids (**4 ab**) were also viable coupling partners, although in the case of **4 aa**, protodeboronation appeared to be facile and 30 % of the protodeboronative C(sp^2^)−C(sp^3^) cross‐coupled product **3 aa** was also obtained. This overall method is complementary to conventional Grignard addition but in many cases afforded products that are not compatible with organometallic species.

To illustrate the utility of our newly developed method, we first turned to the trapping of the tertiary boronic acid derived from the coupling of 4‐chlorophenylboronic acid and the cyclobutane oxadiazoline precursor, using excess pinacol as the final workup quench, which provided the valuable Bpin product **5** in 74 % yield (Scheme 3 a). General methods to access and assess the reactivity of these tertiary alkylboron pinacol esters are vastly underdeveloped, so these examples serve as testament to this versatile cross‐coupling route, for the synthesis of highly valued organoboron intermediates. Utilization of **5** for the generation of valuable and pharmaceutically relevant tertiary cyclobutylamines was successfully demonstrated, providing the benzyl protected derivative **6** in 70 % yield over two steps.^15^ Furthermore, we were also able to construct quaternary carbon centers using two recently developed methodologies for C(sp^2^)−C(sp^3^) cross‐coupling of boronic esters: reaction of **5** with 2‐lithiofuran and a subsequent quench with *N*‐bromosuccinimide (NBS) led to cyclobutylated furan derivative **7** in 47 % yield;^16^ whereas an iridium‐catalyzed photoredox flow process^17^ allowed us to couple 1‐isoquinolinecarbonitrile to **5**, leading to cyclobutylated isoquinoline derivative **8** in 49 % yield. Finally, we were further able to apply this process to the short three‐step synthesis of the GABA receptor agonist drug, baclofen (**10**; Scheme 3 b).^18^

**Scheme 3:**
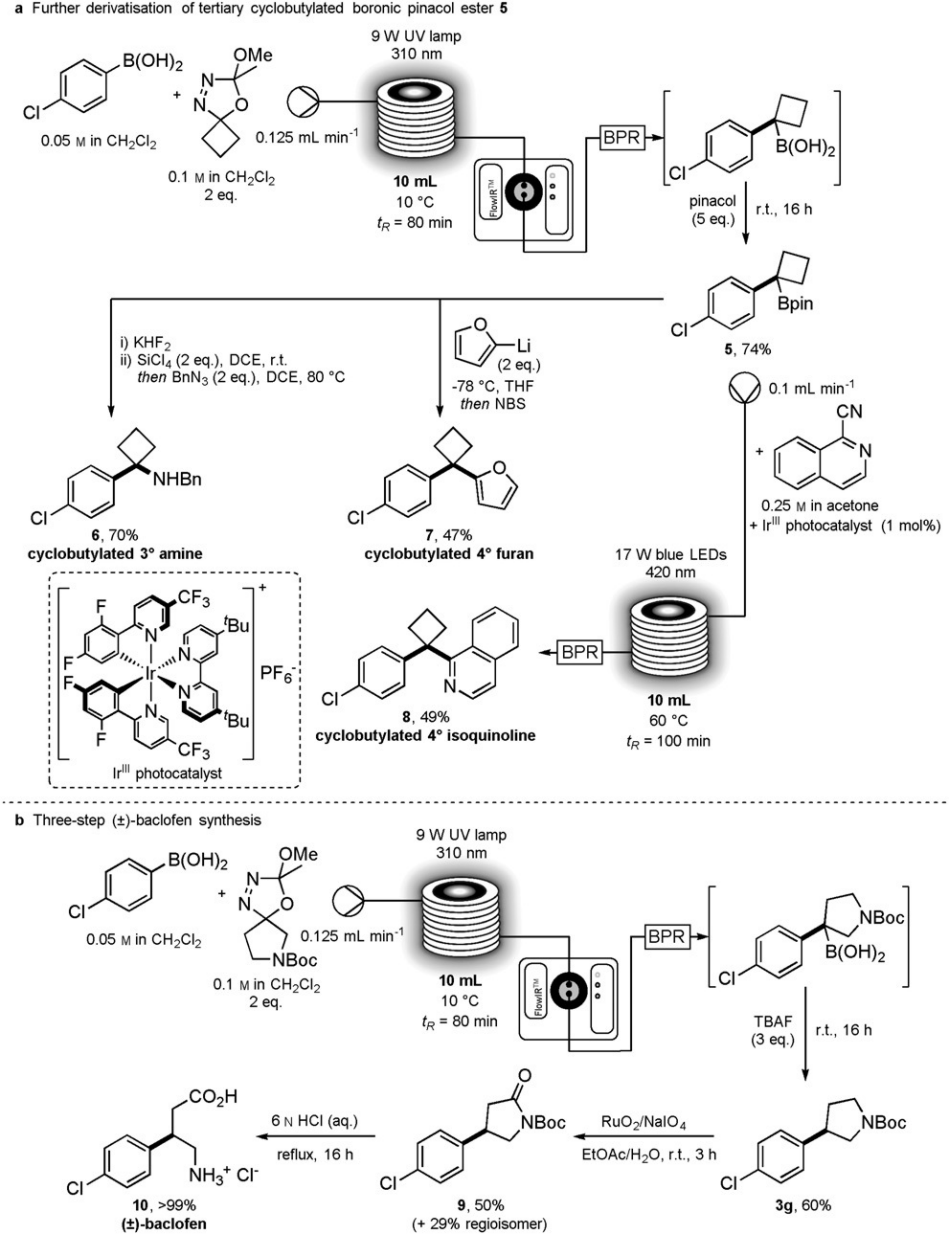
Applications of the divergent C(sp^2^)−C(sp^3^) cross‐coupling process.

In summary, this work clearly demonstrates how oxadiazolines may be used as efficient, bench‐stable precursors for non‐stabilized diazo compounds. Indeed, with these improvements in metal‐free C(sp^2^)−C(sp^3^) cross‐coupling, this process complements the well‐established organometallic and organohalide cross‐coupling procedures. In combination with readily available arylboronic acids, this newly developed flow method allows a myriad of potent, divergent, metal‐free C(sp^2^)−C(sp^3^) cross‐coupling processes exhibiting a very general reaction scope and unparalleled functional‐group tolerance, as well as access to new tertiary alkyl boronic pinacol ester derivatives. Further applications of these unstable diazo compounds are currently ongoing in our laboratories.

## Conflict of interest

P.P. is an employee of UCB Biopharma SPRL. D.C.B. is an employee and stockholder of Pfizer Inc.

## Supporting information

As a service to our authors and readers, this journal provides supporting information supplied by the authors. Such materials are peer reviewed and may be re‐organized for online delivery, but are not copy‐edited or typeset. Technical support issues arising from supporting information (other than missing files) should be addressed to the authors.

Supplementary
