# Supplementary material for: A Versatile Route to Unstable Diazo Compounds via Oxadiazolines and their Use in Aryl–Alkyl Cross‐Coupling Reactions
Source: Angew Chem Int Ed Engl. 2017 Dec 5;56(52):16602–5. doi: 10.1002/anie.201710445 (PMC5814725; doi:10.1002/anie.201710445)
Supplement: Supplementary file 1 — Supplementary [file ANIE-56-16602-s001.pdf]

Angewandte Chemie
Eine Zeitschrift der Gesellschaft Deutscher Chemiker

Supporting Information

A Versatile Route to Unstable Diazo Compounds via Oxadiazolines and their Use in Aryl–Alkyl Cross-Coupling Reactions

*Andreas Greb[+], Jian-Siang Poh[+], Stephanie Greed, Claudio Battilocchio, Patrick Pasau, David C. Blakemore, and Steven V. Ley**

anie_201710445_sm_miscellaneous_information.pdf

Contents

1. General experimental details 3
2. Synthetic procedures and characterisation for oxadiazolines 5
3. Optimisation results 20
4. Synthetic procedures and characterisation for cross-coupled products 21
    4.1. Protodeboronative couplings 21
    4.2. Oxidative couplings 37
5. Synthesis of cyclobutylated boronic ester **5** and further derivatisation 51
6. Synthesis of (±)-baclofen 54
7. Differential scanning calorimetry data 56
8. NMR spectra 57
    8.1. NMR spectra for oxadiazolines 57
    8.2. NMR spectra for protodeboronative coupling products 85
    8.3. NMR spectra for oxidative coupling products 120
    8.4. NMR spectra for compound **5** and derivatised products 149
    8.5. NMR spectra for (±)-baclofen synthesis 155
9. Differential scanning calorimetry curves 158

References 160

1. General experimental details

All batch reactions were performed using oven-dried glassware (200 °C) under an atmosphere of argon unless otherwise stated. All flow reactions were performed using a Vapourtec E-series system.[1] Solvents were freshly distilled over sodium benzophenone ketyl (Et_2O, THF) or calcium hydride (EtOAc, acetone, MeOH, CH_2Cl_2 and toluene). Additional anhydrous solvents were obtained from commercial sources and used directly (DMSO and DCE). DIPEA was freshly distilled over calcium hydride and stored over 4 Å molecular sieves. ^{n}BuLi was titrated using BHT and 1,10-phenanthroline as indicator prior to use. All reagents were obtained from commercial sources and used without further purification.

Flash column chromatography was performed using high-purity grade silica gel (Merck grade 9385) with a pore size 60 Å and 230–400 mesh particle size under air pressure. Analytical thin layer chromatography (TLC) was performed using silica gel 60 F_{254} pre-coated glass backed plates and visualised by ultraviolet radiation (254 nm) and/or potassium permanganate solution as appropriate.

^{1}H NMR spectra were recorded on a 600 MHz Avance 600 BBI Spectrometer as indicated. Chemical shifts are reported in ppm with the resonance resulting from incomplete deuteration of the solvent as the internal standard ($CDCl_3$: 7.26 ppm; MeOD-d_4: 3.31 ppm, qn; MeCN-d_3: 1.94 ppm, qn; D_2O: 4.79 ppm). ^{13}C NMR spectra were recorded the same spectrometer with complete proton decoupling. Chemical shifts are reported in ppm with the solvent resonance as the internal standard ($^{13}CDCl_3$: 77.16 ppm, t; MeOD-d_4: 49.00 ppm, sept; MeCN-d_3: 1.32 ppm, sept). ^{19}F NMR spectra were recorded on a 376 MHz Avance III HD Spectrometer with complete proton decoupling. Chemical shifts are reported in ppm with $CFCl_3$ as the external standard ($CFCl_3$: 0.00 ppm). ^{31}P NMR spectra were recorded on an Avance 600 BBI Spectrometer with complete proton decoupling. Chemical shifts are reported in ppm with 85% phosphoric acid in D_2O as the external standard (H_3PO_4: 0.00 ppm). Data are reported as follows: chemical shift δ/ppm, integration (^{1}H, ^{19}F and ^{31}P), multiplicity (s = singlet, d = doublet, t = triplet, q = quartet, qn = quintet, sept = septet, oct = octet, br = broad, m = multiplet or combinations thereof; ^{13}C signals are singlets unless otherwise stated), coupling constants *J* in Hz, assignment. Spectra are assigned as fully as possible, using ^{1}H-COSY, DEPT-135, HSQC, HMBC and NOESY where appropriate to facilitate structural determination. Signals that cannot be unambiguously assigned are reported with all possible assignments separated by a slash (e.g. H1/H2) or descriptions of their environments (e.g. ArH, NH, OH). Multiple signals arising from diastereotopic or (pseudo)axial/equatorial positions are suffixed alphabetically (e.g. H1a, H1b). Overlapping signals that cannot be resolved are reported with their assignments denoted in list format (e.g. H1, H2 and H3). ^{1}H NMR signals are reported to 2 decimal places and ^{13}C signals to 1 decimal place unless rounding would produce a value identical to another signal. In this case, an additional decimal place is reported for both signals concerned.

Infrared spectra were recorded neat as thin films on a Perkin-Elmer Spectrum One FTIR spectrometer and selected peaks are reported (s = strong, m = medium, w = weak, br = broad).

High resolution mass spectrometry (HRMS) was performed using positive or negative electrospray ionisation (ESI), on either a Waters Micromass LCT Premier spectrometer or performed by the Mass Spectrometry Service for the Department of Chemistry at the University of Cambridge. All *m/z* values are reported to 4 decimal places and are within ± 5 ppm of theoretical values.

Melting points were recorded on a Stanford Research Systems OptiMelt Automated Melting Point System calibrated against vanillin (m.p. 83 °C), phenacetin (m.p. 136 °C) and caffeine (m.p. 237 °C).

2. Synthetic procedures and characterisation for oxadiazolines

General procedure for oxadiazoline synthesis: A solution of the appropriate ketone (20.0 mmol, 1 equiv.) and acetic hydrazide (1.63 g, 22.0 mmol, 1.1 equiv.) in toluene (60 mL) was heated under reflux with equipped Dean-Stark apparatus for 2 h. The mixture was then evaporated under reduced pressure and the residue redissolved in MeOH (60 mL) and cooled to 0 °C. (Diacetoxyiodo)benzene (7.09 g, 22.0 mmol, 1.1 equiv.) was added portionwise, then the mixture stirred further at this temperature for 1 h. The mixture was evaporated under reduced pressure and the residue purified by silica gel column chromatography.

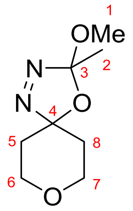

1

3-methoxy-3-methyl-4,8-dioxa-1,2-diazaspiro[4.5]dec-1-ene (1): Following the general procedure for oxadiazoline synthesis using tetrahydro-4*H*-pyran-4-one (4.00 g, 40.0 mmol), purified by silica gel column chromatography (eluent: hexane → 10% EtOAc/hexane) provided the title compound as a colourless oil (6.39 g, 34.3 mmol, 86%).

^{1}H NMR (600 MHz, $CDCl_3$): δ 4.12 – 4.06 (m, 1 H, H6/H7), 4.06 – 4.00 (m, 1 H, H6/H7), 3.91 – 3.83 (m, 2 H, H6/H7), 3.13 (s, 3 H, H1), 2.26 – 2.18 (m, 1 H, H5/H8), 2.08 – 2.01 (m, 1 H, H5/H8), 1.79 – 1.72 (m, 1 H, H5/H8), 1.66 (s, 3 H, H2), 1.61 – 1.54 (m, 1 H, H5/H8).
^{13}C NMR (150 MHz, $CDCl_3$): δ 133.2 (C3), 118.8 (C4), 65.1 (C6 and C7), 50.7 (C1), 35.2 (C5/C8), 34.1 (C5/C8), 23.9 (C2).
FTIR (ν_{max}, cm^{-1}): 2963 (w), 2865 (w), 1739 (w), 1575 (w), 1470 (w), 1436 (w), 1378 (m), 1350 (w), 1304 (w), 1234 (m), 1200 (s), 1152 (s), 1119 (m), 1098 (s), 1054 (s), 1029 (m), 1019 (m), 1008 (m), 990 (m), 941 (m), 906 (s), 868 (s), 842 (w), 807 (w), 770 (w).
HRMS (ESI): calculated for $C_8H_{15}N_2O_3$ $[M+H]^+$ 187.1077, found 187.1070.
R_f = 0.13 (10% EtOAc/hexane).

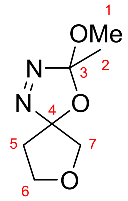

3-methoxy-3-methyl-4,7-dioxa-1,2-diazaspiro[4.4]non-1-ene: Following the general procedure for oxadiazoline synthesis using tetrahydrofuran-3-one (3.44 g, 40.0 mmol), purified by silica gel column chromatography (eluent: hexane → 10% EtOAc/hexane) provided the title compound as an inseparable mixture of diastereomers (1:1) as a colourless oil (4.90 g, 28.5 mmol, 71%).

^{1}H NMR (600 MHz, $CDCl_3$): δ 4.25 – 4.17 (m, 1.5 H, H6a of diastereomers A and B, H7a of diastereomer A), 4.12 (dt, *J* = 8.8, 7.2 Hz, 0.5 H, H6b of diastereomer B), 4.10 – 4.04 (m, 0.5 H, H6b of diastereomer A), 4.00 (d, *J* = 10.0 Hz, 0.5 H, H7a of diastereomer B), 3.76 (d, *J* = 10.3 Hz, 0.5 H, H7b of diastereomer A), 3.64 (d, *J* = 10.0 Hz, 0.5 H, H7b of diastereomer B), 3.08 and 3.03 (two s, 3 H, H1), 2.65 – 2.57 (m, 0.5 H, H5a of diastereomer B), 2.43 (dt, *J* = 13.2, 7.7 Hz, 0.5 H, H5a of diastereomer A), 2.17 (ddd, *J* = 13.3, 7.2, 6.1 Hz, 0.5 H, H5b of

diastereomer B), 2.03 – 1.97 (m, 0.5 H, H5b of diastereomer A), 1.63 and 1.59 (two s, 3 H, H2).
^{13}C NMR (150 MHz, $CDCl_3$): δ 134.4 and 134.2 (C3), 126.7 and 126.6 (C4), 73.4 and 73.1 (C7), 68.9 and 68.8 (C6), 50.5 and 50.2 (C1), 36.8 and 36.2 (C5), 22.9 and 22.6 (C2).
FTIR (ν_{max}, cm^{-1}): 2993 (w), 2947 (w), 2867 (w), 1571 (w), 1459 (w), 1435 (w), 1380 (m), 1334 (w), 1206 (s), 1148 (s), 1105 (m), 1051 (s), 965 (w), 911 (s), 869 (m), 764 (w).
HRMS (ESI): calculated for $C_7H_{13}N_2O_3$ $[M+H]^+$ 173.0921, found 173.0928.
R_f = 0.23 (10% EtOAc/hexane).

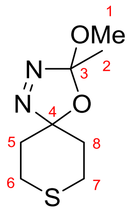

3-methoxy-3-methyl-4-oxa-8-thia-1,2-diazaspiro[4.5]dec-1-ene: Following the general procedure for oxadiazoline synthesis using tetrahydro-4*H*-thiopyran-4-one (2.32 g, 20.0 mmol), purified by silica gel column chromatography (eluent: hexane → 10% EtOAc/hexane) provided the title compound as a colourless oil (3.15 g, 15.6 mmol, 78%).

^{1}H NMR (600 MHz, $CDCl_3$): δ 3.14 (s, 3 H, H1), 2.95 – 2.79 (m, 4 H, H6 and H7), 2.41 (ddd, *J* = 13.8, 10.0, 4.0 Hz, 1 H, H5/H8), 2.26 (ddd, *J* = 13.8, 10.4, 3.6 Hz, 1 H, H5/H8), 1.94 – 1.88 (m, 1 H, H5/H8), 1.69 (ddd, *J* = 13.8, 6.1, 2.8 Hz, 1 H, H5/H8), 1.66 (s, 3 H, H2).
^{13}C NMR (150 MHz, $CDCl_3$): δ 133.5 (C3), 120.2 (C4), 50.8 (C1), 35.8 (C5/C8), 34.8 (C5/C8), 25.4 (C6/C7), 25.3 (C6/C7), 23.9 (C2).
FTIR (ν_{max}, cm^{-1}): 2998 (w), 2947 (w), 2920 (w), 2836 (w), 1574 (w), 1430 (m), 1377 (m), 1353 (w), 1321 (w), 1275 (w), 1226 (m), 1200 (s), 1156 (s), 1093 (m), 1083 (s), 1048 (s), 1010 (w), 986 (w), 946 (s), 901 (s), 850 (m), 786 (w), 767 (w).
HRMS (ESI): calculated for $C_8H_{15}N_2O_2S$ $[M+H]^+$ 203.0849, found 203.0851.
R_f = 0.39 (10% EtOAc/hexane).

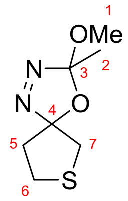

3-methoxy-3-methyl-4-oxa-7-thia-1,2-diazaspiro[4.4]non-1-ene: Following the general procedure for oxadiazoline synthesis using 4,5-dihydro-3(2*H*)-thiophenone (2.04 g, 20.0 mmol), purified by silica gel column chromatography (eluent: hexane → 10% EtOAc/hexane) provided the title compound as an inseparable mixture of diastereomers (1:1) as a pale yellow oil (2.30 g, 12.2 mmol, 61%).

^{1}H NMR (600 MHz, $CDCl_3$): δ 3.44 (d, *J* = 12.0 Hz, 0.5 H, H7a of diastereomer A), 3.27 (d, *J* = 11.8 Hz, 0.5 H, H7a of diastereomer B), 3.23 – 3.14 (m, 1 H, H6a of diastereomers A and B), 3.12 and 3.11 (two s, 3 H, H1), 3.11 – 3.06 (m, 1 H, H6b of diastereomers A and B), 2.94 (dd, *J* = 12.0, 0.9 Hz, 0.5 H, H7b of diastereomer A), 2.75 (dd, *J* = 11.8, 0.9 Hz, 0.5 H, H7b of diastereomer B), 2.59 (dt, *J* = 13.2, 7.7 Hz, 0.5 H, H5a of diastereomer A), 2.44 – 2.36 (m, 0.5 H, H5a of diastereomer B), 2.28 – 2.20 (m, 0.5 H, H5b of diastereomer A), 2.09 – 2.03 (m, 0.5 H, H5b of diastereomer B), 1.643 and 1.642 (two s, 3 H, H2).

^{13}C NMR (150 MHz, $CDCl_3$): δ 134.6 and 134.5 (C3), 128.45 and 128.39 (C4), 50.73 and 50.69 (C1), 39.8 and 39.0 (C5), 37.6 and 36.8 (C7), 29.49 and 29.48 (C6), 23.1 and 22.9 (C2).
FTIR (ν_{max}, cm^{-1}): 2972 (w), 2941 (w), 1738 (w), 1570 (w), 1456 (w), 1435 (w), 1378 (m), 1315 (w), 1225 (m), 1204 (s), 1153 (s), 1124 (m), 1088 (m), 1051 (s), 1017 (w), 977 (w), 947 (m), 908 (s), 866 (m), 834 (w), 770 (w).
HRMS (ESI): calculated for $C_7H_{13}N_2O_2S$ $[M+H]^+$ 189.0692, found 189.0690.
R_f = 0.37 (10% EtOAc/hexane).

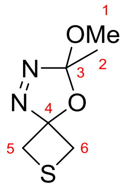

7-methoxy-7-methyl-8-oxa-2-thia-5,6-diazaspiro[3.4]oct-5-ene: Following the general procedure for oxadiazoline synthesis using 3-thietanone (0.97 g, 11.0 mmol), purified by silica gel column chromatography (eluent: hexane → 40% CH_2Cl_2/hexane) provided the title compound as a pale yellow oil (0.57 g, 3.26 mmol, 30%).

^{1}H NMR (600 MHz, $CDCl_3$): δ 3.84 (d, *J* = 10.2 Hz, 1 H, H5a), 3.77 (d, *J* = 10.2 Hz, 1 H, H6a), 3.45 (dd, *J* = 10.2, 1.8 Hz, 1 H, H5b), 3.29 (dd, *J* = 10.2, 1.8 Hz, 1 H, H6b), 2.92 (s, 3 H, H1), 1.57 (s, 3 H, H2).
^{13}C NMR (150 MHz, $CDCl_3$): δ 136.1 (C3), 117.8 (C4), 50.2 (C1), 37.3 (C5/C6), 37.1 (C5/C6), 23.3 (C2).
FTIR (ν_{max}, cm^{-1}): 2999 (w), 2942 (w), 2837 (w), 1568 (w), 1459 (w), 1437 (w), 1418 (w), 1380 (m), 1237 (m), 1204 (s), 1174 (m), 1158 (m), 1134 (m), 1097 (s), 1049 (s), 959 (w), 909 (s), 862 (m), 776 (w).
HRMS (ESI): calculated for $C_6H_{11}N_2O_2S$ $[M+H]^+$ 175.0536, found 175.0540.
R_f = 0.29 (40% CH_2Cl_2/hexane).

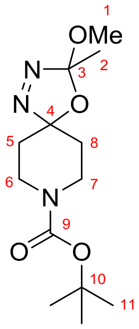

***tert*-butyl 3-methoxy-3-methyl-4-oxa-1,2,8-triazaspiro[4.5]dec-1-ene-8-carboxylate:** Following the general procedure for oxadiazoline synthesis using *N*-Boc-4-piperidone (4.28 g, 25.0 mmol), purified by silica gel column chromatography (eluent: hexane → 20% EtOAc/hexane) provided the title compound as a white amorphous solid (5.43 g, 19.0 mmol, 76%), m.p. 60-62 °C.

^{1}H NMR (600 MHz, $CDCl_3$): δ 4.02 – 3.79 (m, 2 H, H6/H7), 3.51 – 3.41 (m, 2 H, H6/H7), 3.14 (s, 3 H, H1), 2.19 – 2.09 (m, 1 H, H5/H8), 2.04 – 1.92 (m, 1 H, H5/H8), 1.73 – 1.62 (m, 1 H, H5/H8), 1.66 (s, 3 H, H2), 1.52 – 1.46 (m, 1 H, H5/H8), 1.48 (s, 9 H, H11).
^{13}C NMR (150 MHz, $CDCl_3$): δ 154.7 (C9), 133.4 (C3), 119.7 (C4), 80.2 (C10), 50.8 (C1), 41.8 – 41.1 (br, C6/C7), 41.1 – 40.5 (br, C6/C7), 34.4 – 34.3 (br, C5/C8), 33.4 – 33.2 (br, C5/C8), 28.6 (C11), 23.8 (C2).

FTIR (ν_{max}, cm^{-1}): 2973 (w), 1693 (s, C=O), 1574 (w), 1468 (w), 1418 (m), 1378 (w), 1366 (m), 1351 (w), 1278 (w), 1246 (m), 1223 (m), 1202 (m), 1154 (s), 1097 (s), 1055 (s), 1003 (w), 966 (w), 933 (m), 906 (s), 867 (m), 825 (w), 804 (w), 769 (w).
HRMS (ESI): calculated for $C_{13}H_{23}N_3O_4Na$ $[M+Na]^+$ 308.1581, found 308.1590.
R$_f$ = 0.37 (20% EtOAc/hexane).

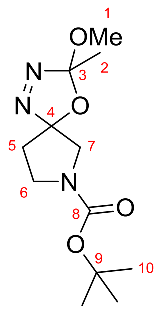

***tert*-butyl 3-methoxy-3-methyl-4-oxa-1,2,7-triazaspiro[4.4]non-1-ene-7-carboxylate:** Following the general procedure for oxadiazoline synthesis using *N*-Boc-3-pyrrolidinone (3.70 g, 20.0 mmol), purified by silica gel column chromatography (eluent: hexane → 20% EtOAc/hexane) provided the title compound as an inseparable mixture of diastereomers (1:1) as a yellow oil (4.19 g, 15.4 mmol, 77%).

^{1}H NMR (600 MHz, MeOD-*d*$_4$): δ 3.82 (d, *J* = 12.2 Hz, 0.5 H, H7a of diastereomer A), 3.79 – 3.60 (m, 2.5 H, H6 of diastereomers A and B, H7a of diastereomer B), 3.49 (d, *J* = 12.2 Hz, 0.5 H, H7b of diastereomer A), 3.40 – 3.32 (m, 0.5 H, H7b of diastereomer B), 3.07 and 3.04 (two s, 3 H, H1), 2.47 (m, 0.5 H, H5a of diastereomer A), 2.33 – 2.20 (m, 1 H, H5b of diastereomer A, H5a of diastereomer B), 2.18 – 2.08 (m, 0.5 H, H5b of diastereomer B), 1.60 and 1.58 (two s, 3 H, H2), 1.50 (br s, 9 H, H10).
^{13}C NMR (150 MHz, MeOD-*d*$_4$): δ 154.5 (C8), 134.6 and 134.3 (C3), 124.1 and 124.00 and 123.3 (rotameric, C4), 80.1 (C9), 52.0 and 51.7 and 51.5 and 51.2 (rotameric, C7), 49.6 and 49.3 (C1), 44.8 and 44.7 and 44.3 and 44.2 (rotameric, C6), 34.3 and 33.55 and 33.51 and 32.8 (rotameric, C5), 27.4 (C10), 21.7 (C2).
FTIR (ν_{max}, cm^{-1}): 2976 (w), 2889 (w), 1740 (w), 1696 (s, C=O), 1479 (w), 1457 (w), 1399 (s), 1366 (m), 1236 (m), 1207 (m), 1145 (s), 1095 (s), 1052 (s), 995 (w), 913 (s), 880 (m), 868 (m), 771 (m).
HRMS (ESI): calculated for $C_{12}H_{21}N_3O_4Na$ $[M+Na]^+$ 294.1424, found 294.1421.
R$_f$ = 0.36 (20% EtOAc/hexane).

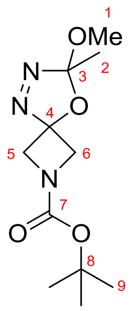

***tert*-butyl 7-methoxy-7-methyl-8-oxa-2,5,6-triazaspiro[3.4]oct-5-ene-2-carboxylate:** Following the general procedure for oxadiazoline synthesis using *N*-Boc-3-azetidinone (2.57 g, 15.0 mmol), purified by silica gel column chromatography (eluent: 10% → 20% EtOAc/hexane) provided the title compound as a white amorphous solid (0.98 g, 3.81 mmol, 25%), m.p. 64-66 °C.

^{1}H NMR (600 MHz, $CDCl_3$): δ 4.49 (dd, *J* = 9.8, 1.1 Hz, 1 H, H5a), 4.38 (dd, *J* = 9.7, 1.1 Hz, 1 H, H6a), 4.20 (dd, *J* = 9.8, 0.9 Hz, 1 H, H5b), 4.14 (dd, *J* = 9.7, 0.9 Hz, 1 H, H6b), 2.93 (s, 3 H, H1), 1.62 (s, 3 H, H2), 1.47 (s, 9 H, H9).
^{13}C NMR (150 MHz, $CDCl_3$): δ 156.0 (C7), 135.8 (C3), 111.7 (C4), 80.7 (C8), 59.4 – 58.0 (br, C5 and C6), 50.1 (C1), 28.4 (C9), 22.7 (C2).
FTIR (ν_{max}, cm^{-1}): 2976 (w), 2946 (w), 1706 (s, C=O), 1564 (w), 1457 (w), 1392 (s), 1382 (s), 1368 (s), 1322 (w), 1248 (m), 1204 (m), 1157 (m), 1098 (s), 1059 (s), 910 (m), 865 (m), 806 (w), 772 (w).
HRMS (ESI): calculated for $C_{11}H_{19}N_3O_4Na$ $[M+Na]^+$ 280.1268, found 280.1265.
***R*$_f$** = 0.44 (20% EtOAc/hexane).

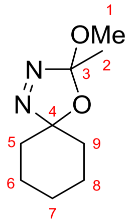

3-methoxy-3-methyl-4-oxa-1,2-diazaspiro[4.5]dec-1-ene: Following the general procedure for oxadiazoline synthesis using cyclohexanone (3.93 g, 40.0 mmol), purified by silica gel column chromatography (eluent: hexane → 5% EtOAc/hexane) provided the title compound as a colourless oil (5.89 g, 32.0 mmol, 80%). Data are consistent with a reported example.[2]

^{1}H NMR (600 MHz, $CDCl_3$): δ 3.11 (s, 3 H, H1), 2.10 – 2.01 (m, 1 H, H5/H9), 1.95 – 1.80 (m, 3 H, H5/H9 and H6/H7/H8), 1.73 – 1.60 (m, 4 H, H5/H9 and H6/H7/H8), 1.64 (s, 3 H, H2), 1.58 – 1.50 (m, 1 H, H6/H7/H8), 1.47 – 1.39 (m, 1 H, H5/H9).
^{13}C NMR (150 MHz, $CDCl_3$): δ 132.5 (C3), 122.0 (C4), 50.6 (C1), 34.9 (C5/C9), 33.6 (C5/C9), 25.0 (C6/C7/C8), 24.2 (C2), 23.1 (C6/C7/C8), 22.9 (C6/C7/C8).
FTIR (ν_{max}, cm^{-1}): 2999 (w), 2940 (m), 2864 (w), 1572 (w), 1450 (m), 1376 (m), 1345 (w), 1233 (m), 1198 (s), 1156 (m), 1127 (m), 1092 (m), 1083 (m), 1056 (s), 1037 (w), 982 (m), 920 (m), 904 (s), 868 (m), 831 (w), 817 (w), 766 (w).
HRMS (ESI): calculated for $C_9H_{17}N_2O_2$ $[M+H]^+$ 185.1285, found 185.1289.
***R*$_f$** = 0.35 (5% EtOAc/hexane).

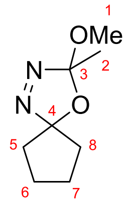

3-methoxy-3-methyl-4-oxa-1,2-diazaspiro[4.4]non-1-ene: Following the general procedure for oxadiazoline synthesis using cyclopentanone (3.36 g, 40.0 mmol), purified by silica gel column chromatography (eluent: hexane → 5% EtOAc/hexane) provided the title compound as a colourless oil (6.41 g, 37.7 mmol, 94%). Data are consistent with a reported example.[2]

^{1}H NMR (600 MHz, $CDCl_3$): δ 3.01 (s, 3 H, H1), 2.29 – 2.20 (m, 1 H, H5/H8), 2.05 – 1.97 (m, 1 H, H5/H8), 1.97 – 1.81 (m, 5 H, H6 and H7, H5/H8), 1.72 – 1.64 (m, 1 H, H5/H8), 1.55 (s, 3 H, H2).
^{13}C NMR (150 MHz, $CDCl_3$): δ 133.1 (C3), 128.8 (C4), 50.2 (C1), 36.5 (C5/C8), 35.6 (C5/C8), 25.4 (C6/C7), 25.2 (C6/C7), 23.1 (C2).

FTIR (ν_{max}, cm^{-1}): 2966 (w), 2878 (w), 2837 (w), 1570 (w), 1455 (w), 1435 (w), 1377 (m), 1329 (w), 1238 (m), 1201 (s), 1155 (s), 1132 (m), 1095 (m), 1055 (s), 1018 (w), 973 (w), 950 (w), 911 (s), 870 (m), 760 (w).
HRMS (ESI): calculated for $C_8H_{15}N_2O_2$ $[M+H]^+$ 171.1128, found 171.1128.
$\boldsymbol{R_f}$ = 0.33 (5% EtOAc/hexane).

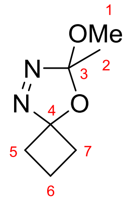

7-methoxy-7-methyl-8-oxa-5,6-diazaspiro[3.4]oct-5-ene: Following the general procedure for oxadiazoline synthesis using cyclobutanone (2.80 g, 40.0 mmol), purified by silica gel column chromatography (eluent: pentane → 20% Et_2O/pentane) provided the title compound as a volatile colourless oil (3.01 g, 19.3 mmol, 48%).

^{1}H NMR (600 MHz, $CDCl_3$): δ 2.93 (s, 3 H, H1), 2.59 – 2.49 (m, 3 H, H5/H7), 2.44 – 2.37 (m, 1 H, H5/H7), 2.28 – 2.17 (m, 1 H, H6a), 1.98 – 1.88 (m, 1 H, H6b), 1.59 (s, 3 H, H2).
^{13}C NMR (150 MHz, $CDCl_3$): δ 133.7 (C3), 119.0 (C4), 50.0 (C1), 33.6 (C5/C7), 33.5 (C5/C7), 23.3 (C2), 11.9 (C6).
FTIR (ν_{max}, cm^{-1}): 2999 (w), 2945 (w), 2837 (w), 1563 (w), 1460 (w), 1438 (w), 1419 (w), 1379 (m), 1275 (m), 1246 (m), 1204 (s), 1182 (s), 1148 (s), 1110 (m), 1066 (s), 1052 (s), 959 (m), 910 (s), 869 (m), 821 (w), 768 (w).
HRMS (ESI): calculated for $C_7H_{13}N_2O_2$ $[M+H]^+$ 157.0972, found 157.0974.
$\boldsymbol{R_f}$ = 0.73 (30% EtOAc/hexane).

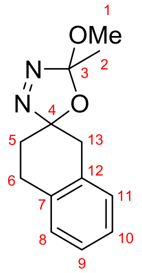

5'-methoxy-5'-methyl-3,4-dihydro-1*H*,5'*H*-spiro[naphthalene-2,2'-[1,3,4]oxadiazole]: Following the general procedure for oxadiazoline synthesis using 2-tetralone (6.50 g, 44.5 mmol), purified by silica gel column chromatography (eluent: hexane → 5% EtOAc/hexane) provided the title compound as an inseparable mixture of diastereomers (1:1) as a red viscous oil (9.01 g, 38.8 mmol, 87%).

^{1}H NMR (600 MHz, $CDCl_3$): δ 7.23 – 7.15 (m, 3 H, H8, H9 and H10), 7.13 – 7.05 (m, 1 H, H11), 3.48 (d, J = 16.9 Hz, 0.5 H, H13a of diastereomer A), 3.35 (d, J = 16.7 Hz, 0.5 H, H13a of diastereomer B), 3.19 and 3.18 (two s, 3 H, H1), 3.17 – 3.02 (m, 2 H, H6), 2.96 (d, J = 16.9 Hz, 0.5 H, H13b of diastereomer A), 2.73 (d, J = 16.7 Hz, 0.5 H, H13b of diastereomer B), 2.42 (ddd, J = 13.4, 9.5, 6.1 Hz, 0.5 H, H5a of diastereomer B), 2.27 – 2.20 (m, 0.5 H, H5a of diastereomer A), 2.03 – 1.96 (m, 0.5 H, H5b of diastereomer B), 1.86 – 1.79 (m, 0.5 H, H5b of diastereomer A), 1.71 and 1.70 (two s, 3 H, H2).
^{13}C NMR (150 MHz, $CDCl_3$): δ 135.08 and 135.05 (C7), 133.59 and 133.57 (C3), 132.5 (C12), 129.1 (C11), 128.77 and 128.76 (C8), 126.67 and 126.65 (C9), 126.46 and 126.44

(C10), 120.8 and 120.7 (C4), 50.7 (C1), 37.8 and 36.9 (C13), 31.9 and 30.7 (C6), 27.0 and 26.7 (C5), 23.91 and 23.88 (C2).
FTIR (ν_{max}, cm^{-1}): 3000 (w), 2941 (w), 2836 (w), 1573 (w), 1497 (w), 1454 (w), 1435 (w), 1377 (m), 1344 (w), 1299 (w), 1227 (m), 1200 (s), 1156 (s), 1136 (m), 1118 (m), 1089 (m), 1050 (s), 1003 (w), 968 (m), 906 (s), 869 (m), 849 (w), 832 (w), 775 (w).
HRMS (ESI): calculated for $C_{13}H_{17}N_2O_2$ $[M+H]^+$ 233.1285, found 233.1293.
R_f = 0.27 (5% EtOAc/hexane).

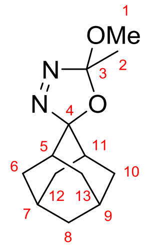

5'-methoxy-5'-methyl-5'*H*-spiro[adamantane-2,2'-[1,3,4]oxadiazole]: Following the general procedure for oxadiazoline synthesis using 2-adamantanone (6.01 g, 40.0 mmol), purified by silica gel column chromatography (eluent: hexane → 10% EtOAc/hexane) provided the title compound as a white crystalline solid (7.55 g, 31.9 mmol, 80%), m.p. 70-72 °C (lit. m.p.[3] 72 °C). Data are consistent with a reported example.[3]

^{1}H NMR (600 MHz, CDCl$_3$): δ 3.07 (s, 3 H, H1), 2.65 – 2.60 (m, 1 H, H6/H10/H12/H13), 2.48 – 2.43 (m, 1 H, H6/H10/H12/H13), 2.10 – 2.04 (m, 2 H, H7/H9, H6/H10/H12/H13), 2.04 – 1.99 (m, 1 H, H6/H10/H12/H13), 1.92 – 1.86 (m, 2 H, H6/H10/H12/H13), 1.85 – 1.76 (m, 4 H, H5/H11, H7/H9, H8), 1.76 – 1.68 (m, 2 H, H6/H10/H12/H13), 1.61 (s, 3 H, H2), 1.55 – 1.51 (m, 1 H, H5/H11).
^{13}C NMR (150 MHz, CDCl$_3$): δ 131.7 (C3), 125.3 (C4), 50.3 (C1), 38.3 (C5/C11), 37.3 (C8), 36.5 (C5/C11), 35.2 (C6/C10/C12/C13), 35.1 (C6/C10/C12/C13), 34.5 (C6/C10/C12/C13), 34.1 (C6/C10/C12/C13), 27.4 (C7/C9), 26.7 (C7/C9), 24.4 (C2).
FTIR (ν_{max}, cm^{-1}): 2998 (w), 2937 (m), 2908 (s), 2854 (m), 1575 (w), 1468 (w), 1451 (m), 1433 (w), 1375 (m), 1363 (w), 1351 (w), 1280 (w), 1247 (m), 1222 (m), 1196 (s), 1155 (m), 1109 (s), 1092 (m), 1059 (s), 1022 (m), 999 (w), 948 (w), 911 (s), 880 (m), 859 (m), 839 (w), 801 (w), 779 (w), 764 (w).
HRMS (ESI): calculated for $C_{13}H_{21}N_2O_2$ $[M+H]^+$ 237.1598, found 237.1590.
R_f = 0.48 (10% EtOAc/hexane).

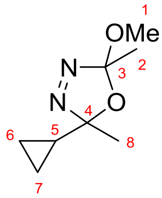

2-cyclopropyl-5-methoxy-2,5-dimethyl-2,5-dihydro-1,3,4-oxadiazole: Following the general procedure for oxadiazoline synthesis using cyclopropyl methyl ketone (3.36 g, 40.0 mmol), purified by silica gel column chromatography (eluent: hexane → 10% EtOAc/hexane) provided the title compound as an inseparable mixture of diastereomers (1:1) as a colourless oil (4.74 g, 27.8 mmol, 70%). Compound has been prepared previously,[2] but NMR spectra were recorded in C_6D_6.

^{1}H NMR (600 MHz, $CDCl_3$): δ 3.27 and 3.17 (two s, 3 H, H1), 1.68 and 1.62 (two s, 3 H, H2), 1.58 and 1.40 (two s, 3 H, H8), 1.33 – 1.23 (m, 1 H, H5), 0.73 – 0.66 (m, 0.5 H, H6/H7), 0.63 – 0.46 (m, 3 H, H6/H7), 0.38 – 0.32 (m, 0.5 H, H6/H7).
^{13}C NMR (150 MHz, $CDCl_3$): δ 133.3 and 133.1 (C3), 122.2 and 122.1 (C4), 51.1 and 50.8 (C1), 23.0 and 22.66 (C2), 22.70 and 22.66 (C8), 18.3 and 17.5 (C5), 2.4 and 1.5 (C6/C7), 1.7 and 1.6 (C6/C7).
FTIR (ν_{max}, cm^{-1}): 2998 (w), 2945 (w), 2837 (w), 1575 (w), 1456 (w), 1376 (m), 1230 (m), 1195 (m), 1144 (s), 1105 (m), 1055 (s), 1027 (m), 968 (m), 930 (m), 908 (m), 885 (m), 870 (m), 840 (w), 822 (w), 786 (w), 757 (w).
HRMS (ESI): calculated for $C_8H_{15}N_2O_2$ $[M+H]^+$ 171.1128, found 171.1130.
R_f = 0.54 (10% EtOAc/hexane).

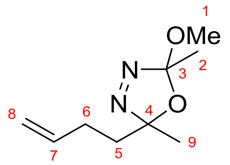

2-(but-3-en-1-yl)-5-methoxy-2,5-dimethyl-2,5-dihydro-1,3,4-oxadiazole: Following the general procedure for oxadiazoline synthesis using 5-hexen-2-one (3.93 g, 40.0 mmol), purified by silica gel column chromatography (eluent: hexane → 10% EtOAc/hexane) provided the title compound as an inseparable mixture of diastereomers (1.5:1) as a colourless oil (4.92 g, 26.7 mmol, 67%).

^{1}H NMR (600 MHz, $CDCl_3$): δ 5.89 – 5.73 (m, 1 H, H7), 5.08 – 4.95 (m, 2 H, H8), 3.17 and 3.16 (two s, 3 H, H1), 2.39 – 2.29 (m, 0.4 H, H6a of diastereomer A), 2.28 – 2.12 (m, 1 H, H6b of diastereomer A, H6a of diastereomer B), 2.11 – 2.00 (m, 1 H, H5a of diastereomer A, H6b of diastereomer B), 1.93 (ddd, J = 13.9, 11.5, 5.1 Hz, 0.6 H, H5a of diastereomer B), 1.79 – 1.70 (m, 1 H, H5b of diastereomers A and B), 1.66 and 1.63 (two s, 3 H, H2), 1.55 and 1.44 (two s, 3 H, H9).
^{13}C NMR (150 MHz, $CDCl_3$): δ 137.5 and 137.3 (C7), 133.4 and 133.2 (C3), 122.3 and 122.1 (C4), 115.34 and 115.30 (C8), 50.80 and 50.75 (C1), 37.6 and 36.8 (C5), 28.2 and 28.0 (C6), 23.4 and 22.6 (C2), 22.5 and 22.4 (C9).
FTIR (ν_{max}, cm^{-1}): 2999 (w), 2946 (w), 1643 (w), 1574 (w), 1455 (w), 1376 (m), 1226 (m), 1190 (m), 1150 (s), 1055 (m), 978 (w), 907 (s), 869 (m).
HRMS (ESI): calculated for $C_9H_{17}N_2O_2$ $[M+H]^+$ 185.1285, found 185.1280.
R_f = 0.33 (5% EtOAc/hexane).

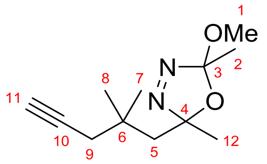

2-(2,2-dimethylpent-4-yn-1-yl)-5-methoxy-2,5-dimethyl-2,5-dihydro-1,3,4-oxadiazole: Following the general procedure for oxadiazoline synthesis using 4,4-dimethylhept-6-yn-2-one (1.38 g, 10.0 mmol), purified by silica gel column chromatography (eluent: hexane → 5% EtOAc/hexane) provided the title compound as an inseparable mixture of diastereomers (1:1) as a colourless oil (1.82 g, 8.1 mmol, 81%).

^{1}H NMR (600 MHz, $CDCl_3$): δ 3.18 and 3.15 (two s, 3 H, H1), 2.30 – 2.16 (m, 2.5 H, H5a of diastereomer A and H9), 2.05 (d, J = 14.8 Hz, 0.5 H, H5a of diastereomer B), 1.99 (t, J = 2.6 Hz, 1 H, H11), 1.67 and 1.61 (two s, 3 H, H2), 1.66 (d, J = 14.8 Hz, 0.5 H, H5b of

diastereomer A), 1.59 and 1.47 (two s, 3 H, H12), 1.54 (d, J = 14.8 Hz, 0.5 H, H5b of diastereomer B), 1.17 and 1.16 (two s, 3 H, H7/H8), 1.114 and 1.107 (two s, 3 H, H7/H8).
^{13}C NMR (150 MHz, $CDCl_3$): δ 134.3 and 133.8 (C3), 122.6 and 122.4 (C4), 82.2 and 82.1 (C10), 70.85 and 70.83 (C11), 51.1 and 50.7 (C1), 47.1 and 46.8 (C5), 33.9 and 33.8 (C6), 33.7 and 33.4 (C9), 28.6 and 28.5 (C7/C8), 28.4 and 28.3 (C7/C8), 24.4 and 24.1 (C12), 23.5 and 22.8 (C2).
FTIR (ν_{max}, cm^{-1}): 3296 (w, alkyne CH), 2964 (m), 2838 (w), 1718 (w), 1577 (w), 1456 (m), 1376 (m), 1195 (s), 1153 (s), 1081 (m), 1056 (s), 1020 (w), 995 (w), 959 (m), 914 (m), 882 (m), 858 (m), 763 (w).
HRMS (ESI): calculated for $C_{12}H_{21}N_2O_2$ $[M+H]^+$ 225.1598, found 225.1602.
R_f = 0.22 (5% EtOAc/hexane).

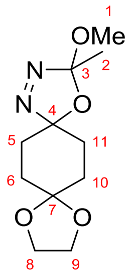

3-methoxy-3-methyl-4,9,12-trioxa-1,2-diazadispiro[4.2.4^8.2^5]tetradec-1-ene: Following the general procedure for oxadiazoline synthesis using 1,4-cyclohexanedione monoethylene acetal (3.12 g, 20.0 mmol), purified by silica gel column chromatography (eluent: 20% EtOAc/hexane) provided the title compound as a white crystalline solid (3.94 g, 16.3 mmol, 81%), m.p. 57-58 °C.

^{1}H NMR (600 MHz, $CDCl_3$): δ 3.98 (s, 4 H, H8 and H9), 3.11 (s, 3 H, H1), 2.34 – 2.26 (m, 1 H, H5/H6/H10/H11), 2.18 – 2.11 (m, 1 H, H5/H6/H10/H11), 2.04 – 1.92 (m, 2 H, H5/H6/H10/H11), 1.91 – 1.84 (m, 2 H, H5/H6/H10/H11), 1.80 – 1.74 (m, 1 H, H5/H6/H10/H11), 1.63 (s, 3 H, H2), 1.59 – 1.52 (m, 1 H, H5/H6/H10/H11).
^{13}C NMR (150 MHz, $CDCl_3$): δ 133.0 (C3), 120.8 (C4), 107.6 (C7), 64.7 (C8/C9), 64.6 (C8/C9), 50.6 (C1), 32.4 (C5/C6/C10/C11), 31.7 (C5/C6/C10/C11), 31.6 (C5/C6/C10/C11), 31.2 (C5/C6/C10/C11), 23.9 (C2).
FTIR (ν_{max}, cm^{-1}): 2941 (w), 2888 (w), 1572 (w), 1439 (w), 1375 (m), 1337 (w), 1299 (w), 1263 (w), 1233 (m), 1200 (m), 1169 (m), 1158 (m), 1107 (s), 1074 (w), 1055 (m), 1034 (s), 995 (m), 968 (m), 947 (m), 921 (m), 906 (s), 864 (w), 854 (w), 766 (w).
HRMS (ESI): calculated for $C_{11}H_{19}N_2O_4$ $[M+H]^+$ 243.1339, found 243.1329.
R_f = 0.31 (20% EtOAc/hexane).

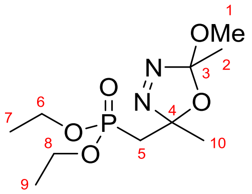

Diethyl ((5-methoxy-2,5-dimethyl-2,5-dihydro-1,3,4-oxadiazol-2-yl)methyl)phosphonate: Following the general procedure for oxadiazoline synthesis using diethyl (2-oxopropyl)phosphonate (3.88 g, 20.0 mmol), purified by silica gel column chromatography (eluent: hexane → 60% EtOAc/hexane) provided the title compound as an inseparable mixture of diastereomers (1.5:1) as a pale yellow oil (3.33 g, 11.9 mmol, 59%).

^{1}H NMR (600 MHz, $CDCl_3$): δ 4.21 – 4.09 (m, 4 H, H6 and H8), 3.16 and 3.15 (two s, 3 H, H1), 2.55 (dd, J = 20.0, 15.3 Hz, 0.4 H, H5a of diastereomer A), 2.42 (dd, J = 19.4, 15.5 Hz, 0.6 H, H5a of diastereomer B), 2.09 (dd, J = 19.2, 15.3 Hz, 0.4 H, H5b of diastereomer A), 1.97 (dd, J = 19.1, 15.5 Hz, 0.6 H, H5b of diastereomer B), 1.77 and 1.669 (two s, 3 H, H10), 1.673 and 1.65 (s, 3 H, H2), 1.36 – 1.30 (m, 6 H, H7 and H9).
^{13}C NMR (150 MHz, $CDCl_3$): δ 135.2 and 135.1 (C3), 118.7 and 118.5 (d, J = 2.9 Hz; d, J = 2.3 Hz, C4), 62.42 and 62.26 (d, J = 6.3 Hz; d, J = 6.4 Hz, C6/C8), 62.15 and 62.11 (d, J = 6.4 Hz; d, J = 6.4 Hz, C6/C8), 50.9 and 50.8 (C1), 35.3 and 34.7 (d, J = 142.5 Hz; d, J = 142.6 Hz, C5), 23.8 and 22.9 (C2), 23.43 and 23.42 (d, J = 4.3 Hz; d, J = 2.0 Hz, C10), 16.54 and 16.50 (d, J = 6.4 Hz; d, J = 6.4 Hz, C7 and C9).
^{31}P NMR (245 MHz, $CDCl_3$): δ 23.4 and 23.2 (two s, 1 P, C5-P).
FTIR (ν_{max}, cm^{-1}): 2987 (w), 1740 (w), 1576 (w), 1444 (w), 1379 (w), 1243 (m), 1202 (m), 1157 (m), 1098 (w), 1049 (s), 1020 (s), 958 (s), 912 (m), 881 (w), 835 (w), 795 (m).
HRMS (ESI): calculated for $C_{10}H_{22}N_2O_5P$ $[M+H]^+$ 281.1261, found 281.1265.
R_f = 0.20 (60% EtOAc/hexane).

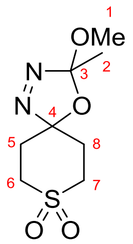

3-methoxy-3-methyl-4-oxa-8-thia-1,2-diazaspiro[4.5]dec-1-ene 8,8-dioxide: To a solution of 3-methoxy-3-methyl-4-oxa-8-thia-1,2-diazaspiro[4.5]dec-1-ene (1.01 g, 5.0 mmol, 1 equiv.) in CH_2Cl_2 (25 mL) was added *m*-CPBA (3.97 g, 11.5 mmol, 2.3 equiv., 50% purity) in four portions at r.t. and the mixture stirred further for 3 h. The mixture was filtered to remove precipitated *m*-chlorobenzoic acid, washed on the filter with CH_2Cl_2 (2 × 15 mL), then the filtrate washed with saturated aqueous Na_2CO_3 solution (50 mL). The organic phase was dried ($MgSO_4$), evaporated under reduced pressure and purified by silica gel column chromatography (eluent: 40% EtOAc/hexane) to provide the title compound as a white amorphous solid (1.13 g, 4.83 mmol, 97%), m.p. 122-123 °C.

^{1}H NMR (600 MHz, $CDCl_3$): δ 3.36 – 3.23 (m, 4 H, H6 and H7), 3.20 (s, 3 H, H1), 2.80 (ddd, J = 14.6, 10.8, 3.9 Hz, 1 H, H5/H8), 2.74 – 2.64 (m, 1 H, H5/H8), 2.17 – 2.10 (m, 1 H, H5/H8), 1.98 – 1.90 (m, 1 H, H5/H8), 1.70 (s, 3 H, H2).
^{13}C NMR (150 MHz, $CDCl_3$): δ 134.9 (C3), 117.3 (C4), 51.2 (C1), 48.2 (C6/C7), 48.1 (C6/C7), 32.8 (C5/C8), 32.0 (C5/C8), 23.1 (C2).
FTIR (ν_{max}, cm^{-1}): 2989 (w), 2944 (w), 1576 (w), 1436 (w), 1402 (w), 1382 (w), 1358 (w), 1337 (m), 1298 (s), 1225 (m), 1203 (m), 1133 (s), 1087 (s), 1051 (m), 1012 (w), 950 (m), 930 (w), 903 (m), 852 (s), 768 (w).
HRMS (ESI): calculated for $C_8H_{15}N_2O_4S$ $[M+H]^+$ 235.0747, found 235.0750.
R_f = 0.33 (40% EtOAc/hexane).

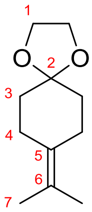

8-(propan-2-ylidene)-1,4-dioxaspiro[4.5]decane: To a suspension of isopropyltriphenylphosphonium bromide (9.40 g, 24.4 mmol, 1 equiv.) and sodium hydride (1.17 g, 29.3 mmol, 1.2 equiv., 60% dispersion in mineral oil) in anhydrous DMSO (15 mL) was heated at 50 °C until formation of a red solution. A solution of 1,4-cyclohexanedione monoethylene acetal (3.81 g, 24.4 mmol) in anhydrous DMSO (15 mL) was then added to the reaction mixture and stirred further at 50 °C for 16 h. The mixture was cooled to r.t., quenched with water (15 mL) then extracted with Et_2O (3 × 25 mL). The combined organic extracts were dried ($MgSO_4$), evaporated under reduced pressure and purified by silica gel column chromatography (eluent: 5% EtOAc/hexane) to provide the title compound as a colourless oil (1.57 g, 8.6 mmol, 35%). Data are consistent with a reported example.[4]

^{1}H NMR (600 MHz, $CDCl_3$): δ 3.95 (s, 4 H, H1), 2.32 – 2.25 (m, 4 H, H4), 1.67 (s, 6 H, H7), 1.65 – 1.61 (m, 4 H, H3).
^{13}C NMR (150 MHz, $CDCl_3$): δ 129.4 (C5), 122.0 (C6), 109.2 (C2), 64.4 (C1), 35.8 (C3), 26.9 (C4), 20.3 (C7).
FTIR (ν_{max}, cm^{-1}): 2994 (m), 2877 (m), 1445 (w), 1364 (w), 1343 (w), 1280 (w), 1233 (w), 1136 (m), 1109 (s), 1070 (m), 1036 (m), 995 (w), 944 (m), 909 (m), 865 (w), 770 (w).
HRMS (ESI): calculated for $C_{11}H_{19}O_2$ $[M+H]^+$ 183.1380, found 183.1381.
R_f = 0.15 (5% EtOAc/hexane).

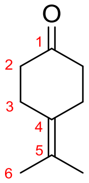

4-(propan-2-ylidene)cyclohexan-1-one: To a suspension of silica gel (3.4 g) in CH_2Cl_2 (10 mL) was added 15% aqueous H_2SO_4 (0.6 mL) and stirred for 5 min. 8-(propan-2-ylidene)-1,4-dioxaspiro[4.5]decane (1.50 g, 8.23 mmol) was added and the mixture stirred at r.t. for 2 h. The silica was filtered off, washed with CH_2Cl_2 (25 mL) and the filtrate evaporated under reduced pressure. The residue was purified by silica gel column chromatography (eluent: 10% EtOAc/hexane) to provide the title compound as a colourless oil (0.819 g, 5.93 mmol, 72%). Data are consistent with a reported example.[4]

^{1}H NMR (600 MHz, $CDCl_3$): δ 2.58 – 2.49 (m, 4 H, H3), 2.43 – 2.36 (m, 4 H, H2), 1.71 (s, 6 H, H6).
^{13}C NMR (150 MHz, $CDCl_3$): δ 213.2 (C1), 126.3 (C4), 125.0 (C5), 40.5 (C2), 27.1 (C3), 20.4 (C6).
FTIR (ν_{max}, cm^{-1}): 2912 (w), 2858 (w), 1715 (s, C=O), 1442 (w), 1376 (w), 1342 (w), 1300 (w), 1233 (w), 1167 (w), 1129 (w), 946 (w), 909 (w), 811 (w), 765 (w).
HRMS (ESI): calculated for $C_9H_{15}O$ $[M+H]^+$ 139.1117, found 139.1115.
R_f = 0.33 (10% EtOAc/hexane).

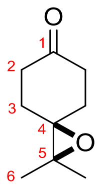

2,2-dimethyl-1-oxaspiro[2.5]octan-6-one: To a solution of 4-(propan-2-ylidene)cyclohexan-1-one (0.819 g, 5.93 mmol, 1 equiv.) in CH_2Cl_2 (40 mL) was added *m*-CPBA (2.25 g, 6.52 mmol, 50% purity) in three portions at 0 °C and the mixture warmed to r.t. and stirred further for 2 h. The mixture was filtered to remove precipitated *m*-chlorobenzoic acid, washed on the filter with CH_2Cl_2 (2 × 15 mL), then the filtrate washed with saturated aqueous Na_2CO_3 solution (25 mL). The aqueous layer was extracted with CH_2Cl_2 (3 × 25 mL) and the combined organic extracts were dried ($MgSO_4$), evaporated under reduced pressure and purified by silica gel column chromatography (eluent: 30% EtOAc/hexane) to provide the title compound as a white amorphous solid (0.734 g, 4.76 mmol, 80%), m.p. 50-52 °C (lit. m.p.[5] 50-51 °C). Data are consistent with a reported example.[5]

^{1}H NMR (600 MHz, $CDCl_3$): δ 2.65 – 2.56 (m, 2 H, H2a), 2.44 – 2.36 (m, 2 H, H2b), 2.10 – 2.02 (m, 2 H, H3a), 1.99 – 1.91 (m, 2 H, H3b), 1.40 (s, 6 H, H6).
^{13}C NMR (150 MHz, $CDCl_3$): δ 210.7 (C1), 64.1 (C4), 63.4 (C5), 38.6 (C2), 29.3 (C3), 21.1 (C6).
FTIR (ν_{max}, cm^{-1}): 2965 (w), 2923 (w), 1717 (s, C=O), 1435 (w), 1378 (w), 1340 (w), 1308 (w), 1233 (w), 1124 (w), 1000 (w), 955 (w), 890 (w), 869 (w).
HRMS (ESI): calculated for $C_9H_{14}O_2Na$ $[M+Na]^+$ 177.0886, found 177.0893.
R_f = 0.31 (30% EtOAc/hexane).

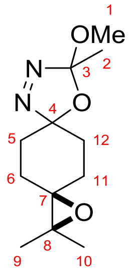

9-methoxy-2,2,9-trimethyl-1,10-dioxa-7,8-diazadispiro[2.2.4^{6}.2^{3}]dodec-7-ene: To a solution of 2,2-dimethyl-1-oxaspiro[2.5]octan-6-one (0.734 g, 4.76 mmol, 1 equiv.) in MeOH (20 mL) was added acetic hydrazide (0.388 g, 5.24 mmol, 1.1 equiv.). The mixture was stirred at r.t. for 2 h, then cooled to 0 °C. (Diacetoxy)iodobenzene (1.69 g, 5.24 mmol, 1.1 equiv.) was added portionwise, then the mixture stirred further at this temperature for 1 h. The mixture was evaporated under reduced pressure and the residue purified by silica gel column chromatography (eluent: 20% EtOAc/hexane) to provide the title compound as an inseparable mixture of diastereomers (1:1) as a white amorphous solid (1.03 g, 4.29 mmol, 90%).

^{1}H NMR (600 MHz, $CDCl_3$): δ 3.15 and 3.14 (two s, 3 H, H1), 2.48 (td, *J* = 12.7, 4.4 Hz, 0.5 H, H5a of diastereomer A), 2.35 (td, *J* = 12.7, 4.4 Hz, 0.5 H, H12a of diastereomer A), 2.18 – 2.11 (m, 0.5 H, H6a of diastereomer B), 2.11 – 1.94 (m, 2.5 H, H6/H11 of diastereomer A, H11a of diastereomer B, H12 of diastereomer B), 1.94 – 1.79 (m, 3 H, H6/H11 of diastereomer A, H5 of diastereomer B, H6b of diastereomer B, H11b of diastereomer B), 1.75 – 1.68 (m, 0.5 H, H5b of diastereomer A), 1.67 and 1.66 (two s, 3 H,

H2), 1.48 – 1.42 (m, 0.5 H, H12b of diastereomer A), 1.42 and 1.38 (two s, 3 H, H9/H10), 1.41 and 1.37 (two s, 3 H, H9/H10).
^{13}C NMR (150 MHz, $CDCl_3$): δ 133.1 and 133.0 (C3), 121.1 and 120.8 (C4), 64.8 and 64.3 (C7), 63.0 and 62.9 (C8), 50.73 and 50.70 (C1), 33.7 and 32.2 (C5/C12), 32.4 and 31.1 (C5/C12), 27.7 and 26.8 (C6/C11), 27.6 and 26.7 (C6/C11), 24.0 and 23.9 (C2), 20.9 and 20.7 (C9 and C10).
FTIR (ν_{max}, cm^{-1}): 2953 (m), 1574 (w), 1439 (m), 1378 (m), 1359 (w), 1225 (m), 1201 (s), 1157 (m), 1102 (s), 1056 (s), 1003 (w), 979 (w), 954 (w), 907 (s), 889 (m), 871 (m), 770 (w).
HRMS (ESI): calculated for $C_{12}H_{20}N_2O_3Na$ $[M+Na]^+$ 263.1366, found 263.1361.
R_f = 0.40 (20% EtOAc/hexane).

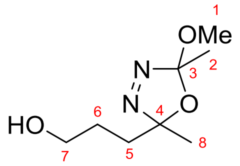

3-(5-methoxy-2,5-dimethyl-2,5-dihydro-1,3,4-oxadiazol-2-yl)propan-1-ol: Following the general procedure for oxadiazoline synthesis using 5-hydroxy-2-pentanone (4.09 g, 40.0 mmol), purified by silica gel column chromatography (eluent: hexane → 40% EtOAc/hexane) provided the title compound as an inseparable mixture of diastereomers (1.4:1) as a colourless oil (4.07 g, 21.6 mmol, 54%).

^{1}H NMR (600 MHz, $CDCl_3$): δ 3.73 – 3.63 (m, 2 H, H7), 3.18 and 3.17 (two s, 3 H, H1), 2.12 – 1.51 (m, 5 H, H5, H6 and OH), 1.68 and 1.64 (two s, 3 H, H2), 1.57 and 1.46 (two s, 3 H, H8).
^{13}C NMR (150 MHz, $CDCl_3$): δ 133.4 and 133.3 (C3), 122.4 and 122.3 (C4), 62.59 and 62.56 (C7), 50.9 and 50.8 (C1), 34.9 and 34.1 (C5), 27.2 and 27.0 (C6), 23.4 and 22.8 (C2), 22.5 and 22.3 (C8).
FTIR (ν_{max}, cm^{-1}): 3431 (br w, OH), 2990 (w), 2945 (w), 1574 (w), 1455 (w), 1377 (m), 1197 (s), 1150 (s), 1102 (w), 1053 (s), 1024 (s), 963 (w), 907 (s), 868 (m).
HRMS (ESI): calculated for $C_8H_{16}N_2O_3Na$ $[M+Na]^+$ 211.1053, found 211.1055.
R_f = 0.26 (40% EtOAc/hexane).

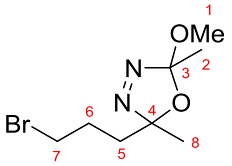

2-(3-bromopropyl)-5-methoxy-2,5-dimethyl-2,5-dihydro-1,3,4-oxadiazole: To a solution of 3-(5-methoxy-2,5-dimethyl-2,5-dihydro-1,3,4-oxadiazol-2-yl)propan-1-ol (0.941 g, 5.0 mmol, 1 equiv.) in CH_2Cl_2 (25 mL) at r.t. was added carbon tetrabromide (1.99 g, 6.0 mmol, 1.2 equiv.) then triphenylphosphine (1.57 g, 6.0 mmol, 1.2 equiv.) in three portions. The mixture was further stirred for 1 h, evaporated under reduced pressure and the residue purified by silica gel column chromatography (eluent: 5% EtOAc/hexane) to provide the title compound as inseparable diastereomers (1.4:1) as a colourless oil (0.991 g, 3.95 mmol, 79%).

^{1}H NMR (600 MHz, $CDCl_3$): δ 3.49 – 3.39 (m, 2 H, H7), 3.20 and 3.18 (two s, 3 H), 2.20 – 1.76 (m, 4 H, H5 and H6), 1.68 and 1.64 (two s, 3 H, H2), 1.56 and 1.44 (two s, 3 H, H8).
^{13}C NMR (150 MHz, $CDCl_3$): δ 133.7 and 133.5 (C3), 121.9 and 121.7 (C4), 51.0 and 50.8 (C1), 36.8 and 36.2 (C5), 33.4 and 33.2 (C7), 27.3 and 27.2 (C6), 23.3 and 22.68 (C2), 22.70 and 22.4 (C8).

FTIR (ν_{max}, cm^{-1}): 2990 (w), 2943 (w), 2837 (w), 1574 (w), 1455 (w), 1377 (m), 1298 (w), 1264 (w), 1239 (m), 1196 (s), 1152 (s), 1089 (m), 1053 (s), 1003 (w), 975 (w), 909 (s), 869 (m), 835 (w), 767 (w).
HRMS (ESI): calculated for $C_8H_{16}N_2O_2Br$ $[M+H]^+$ 251.0390, found 251.0378.
$\boldsymbol{R_f}$ = 0.22 (5% EtOAc/hexane).

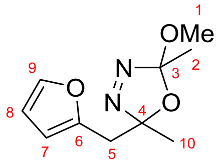

2-(furan-2-ylmethyl)-5-methoxy-2,5-dimethyl-2,5-dihydro-1,3,4-oxadiazole: Following the general procedure for oxadiazoline synthesis using 2-furylacetone (2.48 g, 20.0 mmol), purified by silica gel column chromatography (eluent: hexane → 10% EtOAc/hexane) provided the title compound as an inseparable mixture of diastereomers (2:1) as an orange oil (3.36 g, 16.0 mmol, 80%).

^{1}H NMR (600 MHz, $CDCl_3$): δ 7.37 (d, *J* = 1.3 Hz, 0.33 H, H9 of diastereomer A), 7.32 (d, *J* = 1.3 Hz, 0.67 H, H9 of diastereomer B), 6.35 – 6.31 (m, 0.33 H, H8 of diastereomer A), 6.31 – 6.26 (m, 0.67 H, H8 of diastereomer B), 6.22 (d, *J* = 3.1 Hz, 0.33 H, H7 of diastereomer A), 6.11 (d, *J* = 3.2 Hz, 0.67 H, H7 of diastereomer B), 3.33 (d, *J* = 15.2 Hz, 0.33 H, H5a of diastereomer A), 3.21 – 3.14 (m, 1.33 H, H5 of diastereomer B), 3.13 and 3.12 (two s, 3 H, H1), 3.05 (d, *J* = 15.2 Hz, 0.33 H, H5b of diastereomer A), 1.63 and 1.34 (two s, 3 H, H2), 1.60 and 1.44 (two s, 3 H, H10).
^{13}C NMR (150 MHz, $CDCl_3$): δ 149.2 and 149.0 (C6), 142.2 and 142.1 (C9), 134.5 and 134.1 (C3), 121.3 and 120.5 (C4), 110.73 and 110.71 (C8), 109.3 and 109.1 (C7), 50.8 and 50.7 (C1), 37.1 and 36.4 (C5), 23.4 and 22.3 (C2), 22.8 (C10).
FTIR (ν_{max}, cm^{-1}): 2990 (w), 2945 (w), 1597 (w), 1573 (w), 1505 (w), 1455 (w), 1377 (m), 1188 (m), 1146 (s), 1089 (m), 1054 (s), 1012 (m), 968 (m), 939 (m), 907 (s), 886 (w), 865 (m), 835 (w), 812 (w).
HRMS (ESI): calculated for $C_{10}H_{15}N_2O_3$ $[M+H]^+$ 211.1077, found 211.1076.
$\boldsymbol{R_f}$ = 0.33 (10% EtOAc/hexane).

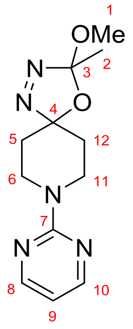

3-methoxy-3-methyl-8-(pyrimidin-2-yl)-4-oxa-1,2,8-triazaspiro[4.5]dec-1-ene: Following the general procedure for oxadiazoline synthesis using 1-(pyrimidin-2-yl)piperidin-4-one (0.98 g, 5.5 mmol), purified by silica gel column chromatography (eluent: hexane → 30% EtOAc/hexane) provided the title compound as a white crystalline solid (1.25 g, 4.7 mmol, 86%), m.p. 96-98 °C.

^{1}H NMR (600 MHz, $CDCl_3$): δ 8.32 (d, *J* = 4.8 Hz, 2 H, H8 and H10), 6.51 (t, *J* = 4.8 Hz, 1 H, H9), 4.44 – 4.33 (m, 2 H, H6/H11), 3.87 – 3.80 (m, 2 H, H6/H11), 3.15 (s, 3 H, H1),

2.23 – 2.16 (m, 1 H, H5/H12), 2.07 – 2.00 (m, 1 H, H5/H12), 1.79 – 1.72 (m, 1 H, H5/H12), 1.68 (s, 3 H, H2), 1.59 – 1.53 (m, 1 H, H5/H12).
^{13}C NMR (150 MHz, $CDCl_3$): δ 161.5 (C7), 158.0 (C8 and C10), 133.3 (C3), 120.3 (C4), 110.2 (C9), 50.8 (C1), 41.12 (C6/C11), 41.07 (C6/C11), 34.2 (C5/C12), 33.1 (C5/C12), 23.9 (C2).
FTIR (ν_{max}, cm^{-1}): 2999 (w), 2962 (w), 2865 (w), 1585 (s), 1548 (s), 1499 (s), 1456 (m), 1394 (w), 1365 (s), 1307 (w), 1262 (m), 1232 (m), 1201 (m), 1157 (m), 1132 (m), 1118 (m), 1096 (m), 1054 (m), 984 (w), 950 (w), 930 (m), 907 (m), 865 (w), 798 (m), 782 (w).
HRMS (ESI): calculated for $C_{12}H_{18}N_5O_2$ $[M+H]^+$ 264.1455, found 264.1465.
R_f = 0.44 (30% EtOAc/hexane).

3. Optimisation results

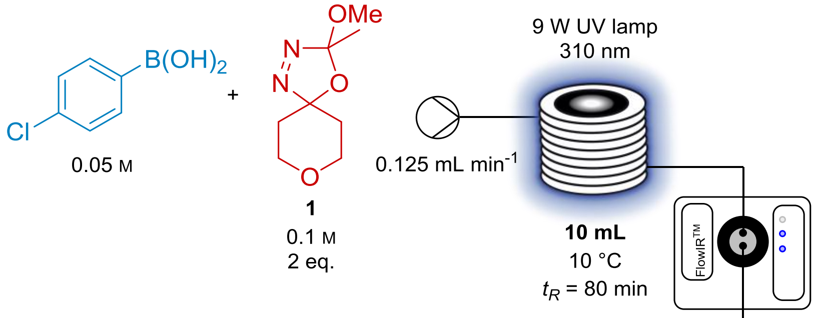

entry	base additive	solvent	'workup'	**3a**, R = H yield[a] / %	**4a**, R = OH yield[a] / %
1[b]	DIPEA (2 eq.)	Et_2O	air	-	46
2	DIPEA (2 eq.)	Et_2O	air	-	55
3[b]	DIPEA (2 eq.)	CH_2Cl_2	air	-	18
**4**	**DIPEA (2 eq.)**	$\mathbf{CH_2Cl_2}$	**air**	**-**	**76**
5[c]	DIPEA (2 eq.)	CH_2Cl_2	air	-	68
6	DIPEA (1 eq.)	CH_2Cl_2	air	-	70
7	TMG (2 eq.)	Et_2O	air	-	traces
8[d]	DIPEA (2 eq.)	CH_2Cl_2	air	-	75
9[e]	DIPEA (2 eq.)	CH_2Cl_2	air	-	76
**10**	**DIPEA (2 eq.)**	$\mathbf{CH_2Cl_2}$	**TBAF**	**71**	**-**

[a] 0.5 mmol of boronic acid, yields stated are of isolated product; [b] *ex situ* generation of diazo compound; [c] t_R = 40 min using 0.25 mL min^{-1} flow rate; [d] 0 °C photoreactor temperature; [e] -10 °C photoreactor temperature.

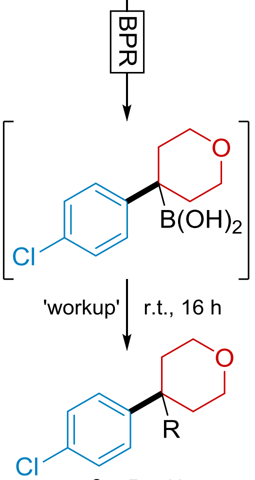

3a: R = H
4a: R = OH

4. Synthetic procedures and characterisation for cross-coupled products

4.1. Protodeboronative couplings

General procedure for protodeboronative coupling: A solution of the appropriate oxadiazoline (1.0 mmol, 2 equiv.), boronic acid (0.5 mmol, 1.0 equiv.) and DIPEA (0.17 mL, 1.0 mmol, 2 equiv.) in CH_2Cl_2 (10 mL) was pumped at a flow rate of 0.125 mL min^{-1} through a Vapourtec UV-150 photochemical reactor (10 mL reactor volume, FEP tubing), irradiated by a 310 nm UV lamp (9 W output power), held at 10 °C and the reactor output was monitored using a FlowIR® device (SiComp head, 2100-2000 cm^{-1} and 1750-1700 cm^{-1}). After 80 min once the reaction mixture has fully been taken up by the pump, the input was swapped to CH_2Cl_2 solvent. When the FlowIR® showed that the reaction plug was exiting the output stream (by monitoring the MeOAc C=O stretch at 1750-1700 cm^{-1}), the reaction plug was directed into a sealed vial containing TBAF (1.5 mL, 1.5 mmol, 3.0 equiv., 1.0 M in THF) and stirred for 16 h. The mixture was then evaporated under reduced pressure, the residue redissolved in EtOAc (5 mL) and filtered through a pad of Celite, eluting with EtOAc. The filtrate was evaporated under reduced pressure and purified by silica gel column chromatography.

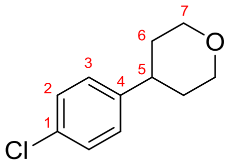

3a

4-(4-chlorophenyl)tetrahydro-2*H*-pyran (3a): Following the general procedure for protodeboronative coupling using 3-methoxy-3-methyl-4,8-dioxa-1,2-diazaspiro[4.5]dec-1-ene (0.186 g, 1.0 mmol) and 4-chlorophenylboronic acid (78.2 mg, 0.5 mmol), purified by silica gel column chromatography (eluent: 10% EtOAc/hexane) provided the title compound as a colourless oil (69.9 mg, 0.355 mmol, 71%). Data are consistent with a reported example.[6]

^{1}H NMR (600 MHz, CDCl$_3$): δ 7.28 (d, *J* = 8.4 Hz, 2 H, H2), 7.15 (d, *J* = 8.4 Hz, 2 H, H3), 4.17 – 4.00 (m, 2 H, H7a), 3.52 (td, *J* = 11.5, 2.7 Hz, 2 H, H7b), 2.73 (tt, *J* = 11.4, 4.5 Hz, 1 H, H5), 1.85 – 1.68 (m, 4 H, H6a and H6b).
^{13}C NMR (150 MHz, CDCl$_3$): δ 144.4 (C4), 132.0 (C1), 128.7 (C2), 128.2 (C3), 68.7 (C7), 41.1 (C5), 34.0 (C6).
FTIR (ν_{max}, cm^{-1}): 2938 (m), 2842 (m), 2757 (w), 1493 (m), 1466 (w), 1442 (w), 1430 (w), 1386 (m), 1365 (w), 1303 (w), 1263 (w), 1237 (m), 1197 (w), 1129 (m), 1089 (s), 1013 (s), 980 (m), 911 (w), 896 (m), 838 (m), 824 (m), 792 (m).
HRMS (ESI): calculated for $C_{11}H_{14}OCl$ [M+H]$^+$ 197.0728, found 197.0737.
R$_f$ = 0.39 (10% EtOAc/hexane).

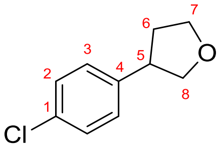

3b

3-(4-chlorophenyl)tetrahydrofuran (3b): Following the general procedure for protodeboronative coupling using 3-methoxy-3-methyl-4,7-dioxa-1,2-diazaspiro[4.4]non-1-ene (0.172 g, 1.0 mmol) and 4-chlorophenylboronic acid (78.2 mg, 0.5 mmol), purified by

silica gel column chromatography (eluent: 10% EtOAc/hexane) provided the title compound as a colourless oil (53.2 mg, 0.291 mmol, 58%).

^{1}H NMR (600 MHz, $CDCl_3$): δ 7.27 (d, *J* = 8.5 Hz, 2 H, H2), 7.18 (d, *J* = 8.5 Hz, 2 H, H3), 4.11 (dd, *J* = 8.5, 7.6 Hz, 1 H, H8a), 4.05 (td, *J* = 8.4, 4.6 Hz, 1 H, H7a), 3.94 – 3.87 (m, 1 H, H7b), 3.69 (dd, *J* = 8.5, 7.2 Hz, 1 H, H8b), 3.42 – 3.32 (m, 1 H, H5), 2.41 – 2.31 (m, 1 H, H6a), 1.99 – 1.92 (m, 1 H, H6b).
^{13}C NMR (150 MHz, $CDCl_3$): δ 141.5 (C4), 132.3 (C1), 128.8 (C2), 128.7 (C3), 74.7 (C8), 68.5 (C7), 44.5 (C5), 34.8 (C6).
FTIR (ν_{max}, cm^{-1}): 2971 (w), 2938 (w), 2861 (w), 1493 (s), 1452 (w), 1413 (w), 1362 (w), 1181 (w), 1091 (s), 1055 (s), 1014 (s), 970 (w), 903 (m), 822 (s).
HRMS (ESI): calculated for $C_{10}H_{12}OCl$ $[M+H]^+$ 183.0571, found 183.0573.
R_f = 0.29 (10% EtOAc/hexane).

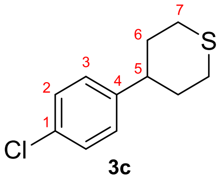

3c

4-(4-chlorophenyl)tetrahydro-2*H*-thiopyran (3c): Following the general procedure for protodeboronative coupling using 3-methoxy-3-methyl-4-oxa-8-thia-1,2-diazaspiro[4.5]dec-1-ene (0.202 g, 1.0 mmol) and 4-chlorophenylboronic acid (78.2 mg, 0.5 mmol), purified by silica gel column chromatography (eluent: 2% EtOAc/hexane) provided the title compound as a white crystalline solid (53.2 mg, 0.291 mmol, 58%), m.p. 69-70 °C (lit. m.p.[7] 70-71 °C).

^{1}H NMR (600 MHz, $CDCl_3$): δ 7.27 (d, *J* = 8.4 Hz, 2 H, H2), 7.12 (d, *J* = 8.4 Hz, 2 H, H3), 2.87 – 2.79 (m, 2 H, H7a), 2.72 – 2.66 (m, 2 H, H7b), 2.50 (tt, *J* = 12.5, 3.1 Hz, 1 H, H5), 2.15 – 2.07 (m, 2 H, H6a), 1.82 (qd, *J* = 12.5, 3.2 Hz, 2 H, H6b).
^{13}C NMR (150 MHz, $CDCl_3$): δ 145.4 (C4), 132.0 (C1), 128.7 (C2), 128.3 (C3), 43.9 (C5), 35.2 (C6), 29.3 (C7).
FTIR (ν_{max}, cm^{-1}): 2926 (w), 2904 (w), 2841 (w), 1596 (w), 1493 (s), 1441 (w), 1428 (w), 1409 (w), 1306 (w), 1269 (m), 1174 (w), 1093 (s), 1013 (m), 985 (w), 952 (m), 902 (w), 825 (s), 783 (m).
HRMS (ESI): calculated for $C_{11}H_{14}SCl$ $[M+H]^+$ 213.0499, found 213.0497.
R_f = 0.19 (2% EtOAc/hexane).

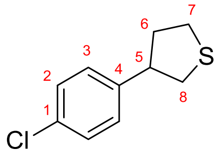

3d

3-(4-chlorophenyl)tetrahydrothiophene (3d): Following the general procedure for protodeboronative coupling using 3-methoxy-3-methyl-4-oxa-7-thia-1,2-diazaspiro[4.4]non-1-ene (0.188 g, 1.0 mmol) and 4-chlorophenylboronic acid (78.2 mg, 0.5 mmol), purified by silica gel column chromatography (eluent: 2% EtOAc/hexane) provided the title compound as a white crystalline solid (59.4 mg, 0.299 mmol, 60%), m.p. 42-44 °C.

^{1}H NMR (600 MHz, $CDCl_3$): δ 7.28 (d, *J* = 8.5 Hz, 2 H, H2), 7.22 (d, *J* = 8.5 Hz, 2 H, H3), 3.35 – 3.26 (m, 1 H, H5), 3.15 (dd, *J* = 10.5, 6.8 Hz, 1 H, H8a), 3.00 – 2.94 (m, 2 H, H7a and

H7b), 2.86 (dd, J = 10.5, 9.6 Hz, 1 H, H8b), 2.42 – 2.35 (m, 1 H, H6a), 2.06 – 1.97 (m, 1 H, H6b).
^{13}C NMR (150 MHz, $CDCl_3$): δ 140.7 (C4), 132.5 (C1), 128.8 (C2), 128.5 (C3), 49.1 (C5), 38.1 (C6), 37.7 (C8), 30.9 (C7).
FTIR (ν_{max}, cm^{-1}): 2932 (w), 2860 (w), 1493 (s), 1456 (w), 1437 (w), 1410 (w), 1262 (w), 1211 (w), 1091 (m), 1014 (m), 886 (w), 865 (w), 824 (m).
HRMS (ESI): calculated for $C_{10}H_{12}SCl$ $[M+H]^+$ 199.0343, found 199.0345.
R$_f$ = 0.38 (2% EtOAc/hexane).

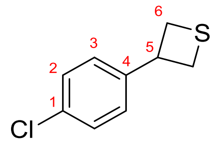

3e

3-(4-chlorophenyl)thietane (3e): Following the general procedure for protodeboronative coupling using 7-methoxy-7-methyl-8-oxa-2-thia-5,6-diazaspiro[3.4]oct-5-ene (0.174 g, 1.0 mmol) and 4-chlorophenylboronic acid (78.2 mg, 0.5 mmol), purified by silica gel column chromatography (eluent: 2% EtOAc/hexane) provided the title compound as a colourless oil (38.9 mg, 0.211 mmol, 42%).

^{1}H NMR (600 MHz, $CDCl_3$): δ 7.31 (d, J = 8.5 Hz, 2 H, H2), 7.23 (d, J = 8.5 Hz, 2 H, H3), 4.52 (qn, J = 9.0 Hz, 1 H, H5), 3.53 (t, J = 9.0 Hz, 2 H, H6a), 3.36 (t, J = 9.0 Hz, 2 H, H6b).
^{13}C NMR (150 MHz, $CDCl_3$): δ 142.6 (C4), 132.8 (C1), 128.9 (C2), 127.4 (C3), 44.6 (C5), 33.1 (C6).
FTIR (ν_{max}, cm^{-1}): 2977 (w), 2938 (w), 2860 (w), 1595 (w), 1492 (s), 1451 (w), 1409 (w), 1326 (w), 1236 (w), 1174 (m), 1091 (s), 1014 (s), 941 (w), 908 (w), 846 (m), 816 (s).
HRMS (ESI): calculated for $C_9H_{10}SCl$ $[M+H]^+$ 185.0186, found 185.0179.
R$_f$ = 0.33 (2% EtOAc/hexane).

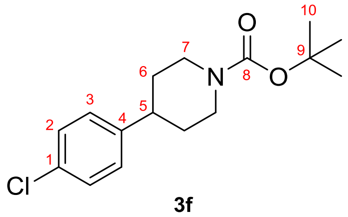

3f

***tert*-butyl 4-(4-chlorophenyl)piperidine-1-carboxylate (3f):** Following the general procedure for protodeboronative coupling using *tert*-butyl 3-methoxy-3-methyl-4-oxa-1,2,8-triazaspiro[4.5]dec-1-ene-8-carboxylate (0.285 g, 1.0 mmol) and 4-chlorophenylboronic acid (78.2 mg, 0.5 mmol), purified by silica gel column chromatography (eluent: 10% EtOAc/hexane) provided the title compound as a colourless gum (124.4 mg, 0.421 mmol, 84%). Data are consistent with a reported example.[8]

^{1}H NMR (600 MHz, $CDCl_3$): δ 7.26 (d, J = 8.5 Hz, 2 H, H2), 7.12 (d, J = 8.5 Hz, 2 H, H3), 4.23 (br s, 2 H, H7a), 2.78 (br s, 2 H, H7b), 2.61 (tt, J = 12.2, 3.5 Hz, 1 H, H5), 1.78 (br d, J = 12.8 Hz, 2 H, H6a), 1.62 – 1.52 (br m, 2 H, H6b), 1.47 (s, 9 H, H10).
^{13}C NMR (150 MHz, $CDCl_3$): δ 154.9 (C8), 144.3 (C4), 132.0 (C1), 128.7 (C2), 128.2 (C3), 79.6 (C9), 45.5 – 43.4 (br, C7), 42.2 (C5), 33.2 (br, C6), 28.6 (C10).
FTIR (ν_{max}, cm^{-1}): 2976 (w), 2934 (w), 2853 (w), 1687 (s, C=O), 1493 (m), 1466 (m), 1445 (m), 1420 (m), 1365 (m), 1320 (w), 1294 (w), 1276 (m), 1230 (s), 1161 (s), 1123 (m), 1091 (m), 1012 (m), 987 (w), 933 (w), 908 (w), 884 (w), 862 (w), 824 (m), 769 (w).

HRMS (ESI): calculated for $C_{16}H_{22}NO_2ClNa$ $[M+Na]^+$ 318.1231, found 318.1229.
$\boldsymbol{R_f}$ = 0.30 (10% EtOAc/hexane).

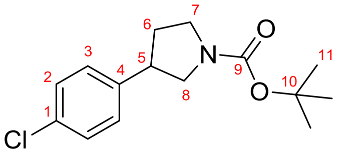

3g

***tert*-butyl 3-(4-chlorophenyl)pyrrolidine-1-carboxylate (3g):** Following the general procedure for protodeboronative coupling using *tert*-butyl 3-methoxy-3-methyl-4-oxa-1,2,7-triazaspiro[4.4]non-1-ene-7-carboxylate (0.271 g, 1.0 mmol) and 4-chlorophenylboronic acid (78.2 mg, 0.5 mmol), purified by silica gel column chromatography (eluent: 10% EtOAc/hexane) provided the title compound as a colourless gum (84.6 mg, 0.300 mmol, 60%). Data are consistent with a reported example.[9]

^{1}H NMR (600 MHz, $CDCl_3$): δ 7.27 (d, *J* = 8.3 Hz, 2 H, H2), 7.15 (two superimposed d from rotamers, *J* = 8.3 Hz, 2 H, H3), 3.88 – 3.79 (m, 0.5 H, H8a of rotamer A), 3.79 – 3.72 (m, 0.5 H, H8a of rotamer B), 3.66 – 3.58 (m, 0.5 H, H7a of rotamer B), 3.57 – 3.49 (m, 0.5 H, H7a of rotamer A), 3.44 – 3.34 (m, 1 H, H7b of rotamers A and B), 3.34 – 3.25 (m, 1.5 H, H5 of rotamers A and B, H8b of rotamer A), 3.22 (t, *J* = 9.9 Hz, 0.5 H, H8b of rotamer B), 2.30 – 2.18 (m, 1 H, H6a of rotamers A and B), 1.99 – 1.87 (m, 1 H, H6b of rotamers A and B), 1.47 and 1.46 (two superimposed s from rotamers, 9 H, H11).
^{13}C NMR (150 MHz, $CDCl_3$): δ 154.6 (C9), 140.1 (C4), 132.6 (C1), 128.8 (C2), 128.5 (C3), 79.4 (C10), 52.6 and 51.8 (rotameric, C8), 45.9 and 45.6 (rotameric, C7), 43.8 and 42.9 (rotameric, C5), 33.4 and 32.5 (rotameric, C6), 28.6 (C11).
FTIR (ν_{max}, cm^{-1}): 2976 (w), 2880 (w), 1688 (s, C=O), 1494 (m), 1478 (w), 1454 (w), 1399 (s), 1365 (s), 1342 (w), 1254 (w), 1164 (s), 1122 (s), 1092 (s), 1014 (m), 984 (w), 922 (w), 879 (m), 826 (m), 772 (m).
HRMS (ESI): calculated for $C_{15}H_{20}NO_2ClNa$ $[M+Na]^+$ 304.1075, found 304.1063.
$\boldsymbol{R_f}$ = 0.22 (10% EtOAc/hexane).

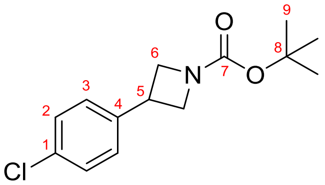

3h

***tert*-butyl 3-(4-chlorophenyl)azetidine-1-carboxylate (3h):** Following the general procedure for protodeboronative coupling using *tert*-butyl 7-methoxy-7-methyl-8-oxa-2,5,6-triazaspiro[3.4]oct-5-ene-2-carboxylate (0.257 g, 1.0 mmol) and 4-chlorophenylboronic acid (78.2 mg, 0.5 mmol), purified by silica gel column chromatography (eluent: 20% EtOAc/hexane) provided the title compound as a colourless gum (70.9 mg, 0.265 mmol, 53%).

^{1}H NMR (600 MHz, $CDCl_3$): δ 7.30 (d, *J* = 8.5 Hz, 2 H, H2), 7.23 (d, *J* = 8.5 Hz, 2 H, H3), 4.31 (t, *J* = 8.7 Hz, 2 H, H6a), 3.97 – 3.87 (dd, *J* = 8.7, 6.0 Hz, 2 H, H6b), 3.68 (tt, *J* = 8.7, 6.0 Hz, 1 H, H5), 1.45 (s, 9 H, H9).
^{13}C NMR (150 MHz, $CDCl_3$): δ 156.4 (C7), 140.8 (C4), 132.7 (C1), 128.9 (C2), 128.3 (C3), 79.8 (C8), 57.2 and 56.0 (br, C6), 33.0 (C5), 28.5 (C9).

FTIR (ν_{max}, cm^{-1}): 2976 (w), 2887 (w), 1697 (s, C=O), 1494 (m), 1478 (w), 1457 (w), 1390 (s), 1365 (s), 1338 (m), 1297 (w), 1250 (w), 1129 (s), 1093 (s), 1060 (w), 1014 (m), 967 (w), 909 (w), 859 (w), 821 (m), 773 (m).
HRMS (ESI): calculated for $C_{14}H_{18}NO_2ClNa$ $[M+Na]^+$ 290.0918, found 290.0910.
$\boldsymbol{R_f}$ = 0.43 (20% EtOAc/hexane).

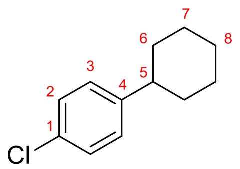

3i

1-chloro-4-cyclohexylbenzene (3i): Following the general procedure for protodeboronative coupling using 3-methoxy-3-methyl-4-oxa-1,2-diazaspiro[4.5]dec-1-ene (0.184 g, 1.0 mmol) and 4-chlorophenylboronic acid (78.2 mg, 0.5 mmol), purified by silica gel column chromatography (eluent: hexane) provided the title compound as a colourless oil (71.9 mg, 0.369 mmol, 74%). Data are consistent with a reported example.[10]

^{1}H NMR (600 MHz, $CDCl_3$): δ 7.26 (d, *J* = 8.4 Hz, 2 H, H2), 7.15 (d, *J* = 8.4 Hz, 2 H, H3), 2.54 – 2.44 (m, 1 H, H5), 1.91 – 1.81 (m, 4 H, H6a and H7a), 1.80 – 1.73 (m, 1 H, H8a), 1.46 – 1.34 (m, 4 H, H6b and H7b), 1.32 – 1.20 (m, 1 H, H8b).
^{13}C NMR (150 MHz, $CDCl_3$): δ 146.6 (C4), 131.4 (C1), 128.5 (C2), 128.3 (C3), 44.1 (C5), 34.6 (C6), 26.9 (C7), 26.2 (C8).
FTIR (ν_{max}, cm^{-1}): 2923 (m), 2851 (m), 1492 (m), 1448 (m), 1409 (w), 1351 (w), 1262 (w), 1178 (w), 1090 (m), 1014 (m), 1000 (w), 892 (w), 818 (s), 775 (w).
HRMS (ESI): calculated for $C_{12}H_{16}Cl$ $[M+H]^+$ 195.0935, found 195.0941.
$\boldsymbol{R_f}$ = 0.76 (hexane).

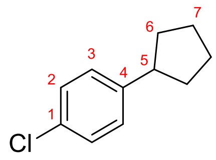

3j

1-chloro-4-cyclopentylbenzene (3j): Following the general procedure for protodeboronative coupling using 3-methoxy-3-methyl-4-oxa-1,2-diazaspiro[4.4]non-1-ene (0.170 g, 1.0 mmol) and 4-chlorophenylboronic acid (78.2 mg, 0.5 mmol), purified by silica gel column chromatography (eluent: hexane) provided the title compound as a colourless oil (52.0 mg, 0.288 mmol, 58%). Data are consistent with a reported example.[11]

^{1}H NMR (600 MHz, $CDCl_3$): δ 7.25 (d, *J* = 8.4 Hz, 2 H, H2), 7.17 (d, *J* = 8.4 Hz, 2 H, H3), 3.01 – 2.90 (m, 1 H, H5), 2.10 – 2.01 (m, 2 H, H6a), 1.86 – 1.75 (m, 2 H, H7a), 1.74 – 1.62 (m, 2 H, H7b), 1.60 – 1.49 (m, 2 H, H6b).
^{13}C NMR (150 MHz, $CDCl_3$): δ 145.1 (C4), 131.3 (C1), 128.6 (C3), 128.4 (C2), 45.5 (C5), 34.7 (C6), 25.6 (C7).
FTIR (ν_{max}, cm^{-1}): 2953 (m), 2870 (m), 1597 (w), 1493 (s), 1451 (w), 1337 (w), 1179 (w), 1091 (s), 1014 (m), 947 (w), 818 (s).
HRMS (ESI): calculated for $C_{11}H_{14}Cl$ $[M+H]^+$ 181.0779, found 181.0784.
$\boldsymbol{R_f}$ = 0.75 (hexane).

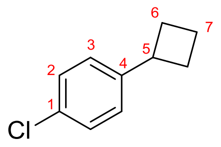

3k

1-chloro-4-cyclobutylbenzene (3k): Following the general procedure for protodeboronative coupling using 7-methoxy-7-methyl-8-oxa-5,6-diazaspiro[3.4]oct-5-ene (0.156 g, 1.0 mmol) and 4-chlorophenylboronic acid (78.2 mg, 0.5 mmol), purified by silica gel column chromatography (eluent: hexane) provided the title compound as a colourless oil (46.0 mg, 0.276 mmol, 55%).

^{1}H NMR (600 MHz, $CDCl_3$): δ 7.26 (d, *J* = 8.4 Hz, 2 H, H2), 7.14 (d, *J* = 8.4 Hz, 2 H, H3), 3.51 (qn, *J* = 8.7 Hz, 1 H, H5), 2.40 – 2.30 (m, 2 H, H6a), 2.16 – 2.07 (m, 2 H, H6b), 2.07 – 1.97 (m, 1 H, H7a), 1.90 – 1.82 (m, 1 H, H7b).
^{13}C NMR (150 MHz, $CDCl_3$): δ 144.8 (C4), 131.4 (C1), 128.4 (C2), 127.8 (C3), 39.9 (C5), 29.9 (C6), 18.3 (C7).
FTIR (ν_{max}, cm^{-1}): 2964 (m), 2940 (m), 2863 (w), 1596 (w), 1491 (m), 1445 (w), 1399 (w), 1333 (w), 1243 (w), 1091 (s), 1014 (m), 916 (w), 872 (w), 820 (s), 752 (w).
HRMS (ESI): calculated for $C_{10}H_{12}Cl$ $[M+H]^+$ 167.0622, found 167.0617.
R_f = 0.78 (hexane).

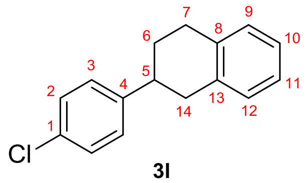

3l

2-(4-chlorophenyl)-1,2,3,4-tetrahydronaphthalene (3l): Following the general procedure for protodeboronative coupling using 5'-methoxy-5'-methyl-3,4-dihydro-1*H*,5'*H*-spiro[naphthalene-2,2'-[1,3,4]oxadiazole] (0.232 g, 1.0 mmol) and 4-chlorophenylboronic acid (78.2 mg, 0.5 mmol), purified by silica gel column chromatography (eluent: hexane) provided the title compound as a pale brown amorphous solid (116.2 mg, 0.479 mmol, 96%), m.p. 93-94 °C.

^{1}H NMR (600 MHz, $CDCl_3$): δ 7.30 (d, *J* = 8.4 Hz, 2 H, H2), 7.21 (d, *J* = 8.4 Hz, 2 H, H3), 7.17 – 7.07 (m, 4 H, H9, H10, H11 and H12), 3.06 – 2.84 (m, 5 H, H5, H7 and H14), 2.15 – 2.09 (m, 1 H, H6a), 1.96 – 1.87 (m, 1 H, H6b).
^{13}C NMR (150 MHz, $CDCl_3$): δ 145.2 (C4), 136.4 (C8/C13), 136.2 (C8/C13), 132.0 (C1), 129.14 (C9/C10/C11/C12), 129.09 (C9/C10/C11/C12), 128.7 (C2), 128.4 (C3), 126.0 (C9/C10/C11/C12), 125.9 (C9/C10/C11/C12), 40.3 (C5), 37.7 (C14), 30.5 (C6), 29.7 (C7).
FTIR (ν_{max}, cm^{-1}): 3019 (w), 2924 (w), 2836 (w), 1599 (w), 1579 (w), 1492 (s), 1451 (m), 1436 (m), 1410 (w), 1344 (w), 1297 (w), 1216 (s), 1180 (w), 1108 (w), 1088 (m), 1056 (w), 1036 (w), 1014 (m), 951 (w), 924 (w), 890 (w), 837 (w), 818 (s), 797 (w).
HRMS (ESI): calculated for $C_{16}H_{16}Cl$ $[M+H]^+$ 243.0935, found 243.0934.
R_f = 0.47 (hexane).

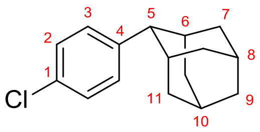

3m

2-(4-chlorophenyl)adamantane (3m): Following the general procedure for protodeboronative coupling using 5'-methoxy-5'-methyl-5'*H*-spiro[adamantane-2,2'-[1,3,4]oxadiazole] (0.236 g, 1.0 mmol) and 4-chlorophenylboronic acid (78.2 mg, 0.5 mmol), purified by silica gel column chromatography (eluent: hexane) provided the title compound as a colourless oil (91.0 mg, 0.369 mmol, 74%).

^{1}H NMR (600 MHz, $CDCl_3$): δ 7.29 (appears s, 4 H, H2 and H3), 2.97 (br s, 1 H, H5), 2.44 (br s, 2 H, H6), 2.05 – 1.97 (m, 3 H, H10 and H11a), 1.94 (br d, *J* = 11.1 Hz, 2 H, H11b), 1.83 – 1.76 (m, 5 H, H7a, H8 and H9), 1.57 (br d, *J* = 12.2 Hz, 2 H, H7b).
^{13}C NMR (150 MHz, $CDCl_3$): δ 143.0 (C4), 130.9 (C1), 128.4 (C2/C3), 128.3 (C2/C3), 46.5 (C5), 39.2 (C11), 37.9 (C9), 32.0 (C7), 31.2 (C6), 28.1 (C10), 27.8 (C8).
FTIR (ν_{max}, cm^{-1}): 2902 (s), 2849 (m), 1594 (w), 1568 (w), 1493 (s), 1469 (w), 1450 (m), 1400 (w), 1355 (w), 1342 (w), 1329 (w), 1282 (w), 1227 (w), 1208 (w), 1092 (m), 1070 (w), 1040 (w), 1013 (m), 971 (m), 950 (w), 913 (w), 870 (w), 851 (m), 832 (m), 818 (m), 784 (m), 761 (m).
HRMS (ESI): calculated for $C_{16}H_{20}Cl$ $[M+H]^+$ 247.1248, found 247.1256.
***R**$_f$* = 0.70 (hexane).

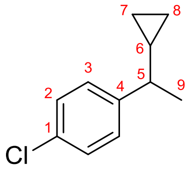

3n

1-chloro-4-(1-cyclopropylethyl)benzene (3n): Following the general procedure for protodeboronative coupling using 2-cyclopropyl-5-methoxy-2,5-dimethyl-2,5-dihydro-1,3,4-oxadiazole (0.236 g, 1.0 mmol) and 4-chlorophenylboronic acid (78.2 mg, 0.5 mmol), purified by silica gel column chromatography (eluent: hexane) provided the title compound as a colourless oil (78.0 mg, 0.432 mmol, 86%).

^{1}H NMR (600 MHz, $CDCl_3$): δ 7.28 (d, *J* = 8.4 Hz, 2 H, H2), 7.20 (d, *J* = 8.4 Hz, 2 H, H3), 2.04 – 1.93 (m, 1 H, H5), 1.33 (d, *J* = 7.1 Hz, 3 H, H9), 0.96 – 0.87 (m, 1 H, H6), 0.61 – 0.54 (m, 1 H, H7/H8), 0.48 – 0.40 (m, 1 H, H7/H8), 0.25 – 0.18 (m, 1 H, H7/H8), 0.17 – 0.11 (m, 1 H, H7/H8).
^{13}C NMR (150 MHz, $CDCl_3$): δ 145.9 (C4), 131.6 (C1), 128.5 (C2/C3), 128.4 (C2/C3), 44.2 (C5), 21.6 (C9), 18.6 (C6), 4.8 (C7/C8), 4.4 (C7/C8).
FTIR (ν_{max}, cm^{-1}): 3077 (w), 3000 (w), 2964 (w), 2875 (w), 1492 (s), 1454 (w), 1428 (w), 1410 (w), 1370 (w), 1282 (w), 1168 (w), 1091 (s), 1036 (w), 1014 (s), 973 (w), 926 (m), 822 (s), 791 (w), 753 (w).
HRMS (ESI): calculated for $C_{11}H_{14}Cl$ $[M+H]^+$ 181.0779, found 181.0783.
***R**$_f$* = 0.69 (hexane).

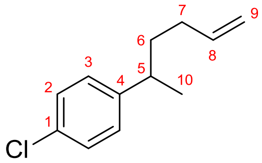

3o

1-chloro-4-(hex-5-en-2-yl)benzene (3o): Following the general procedure for protodeboronative coupling using 2-(but-3-en-1-yl)-5-methoxy-2,5-dimethyl-2,5-dihydro-1,3,4-oxadiazole (0.184 g, 1.0 mmol) and 4-chlorophenylboronic acid (78.2 mg, 0.5 mmol), purified by silica gel column chromatography (eluent: hexane) provided the title compound as a colourless oil (86.6 mg, 0.445 mmol, 89%).

^{1}H NMR (600 MHz, $CDCl_3$): δ 7.26 (d, J = 8.4 Hz, 2 H, H2), 7.12 (d, J = 8.4 Hz, 2 H, H3), 5.78 (ddt, J = 16.9, 10.2, 6.6 Hz, 1 H, H8), 5.00 – 4.93 (m, 2 H, H9), 2.74 – 2.66 (m, 1 H, H5), 2.02 – 1.89 (m, 2 H, H7), 1.71 – 1.60 (m, 2 H, H6), 1.23 (d, J = 7.0 Hz, 3 H, H10).
^{13}C NMR (150 MHz, $CDCl_3$): δ 145.9 (C4), 138.6 (C8), 131.6 (C1), 128.6 (C2/C3), 128.5 (C2/C3), 114.7 (C9), 38.9 (C5), 37.5 (C6), 31.9 (C7), 22.3 (C10).
FTIR (ν_{max}, cm^{-1}): 2960 (w), 2925 (w), 1641 (w), 1493 (m), 1456 (w), 1411 (w), 1376 (w), 1344 (w), 1300 (w), 1180 (w), 1094 (m), 1014 (m), 993 (w), 910 (m), 826 (s), 788 (w), 760 (w).
HRMS (ESI): calculated for $C_{12}H_{16}Cl$ $[M+H]^+$ 195.0935, found 195.0935.
R_f = 0.72 (hexane).

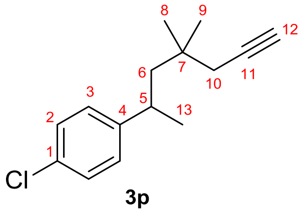

3p

1-chloro-4-(4,4-dimethylhept-6-yn-2-yl)benzene (3p): Following the general procedure for protodeboronative coupling using 2-(2,2-dimethylpent-4-yn-1-yl)-5-methoxy-2,5-dimethyl-2,5-dihydro-1,3,4-oxadiazole (0.224 g, 1.0 mmol) and 4-chlorophenylboronic acid (78.2 mg, 0.5 mmol), purified by silica gel column chromatography (eluent: hexane) provided the title compound as a colourless oil (100.2 mg, 0.427 mmol, 85%).

^{1}H NMR (600 MHz, $CDCl_3$): δ 7.24 (d, J = 8.5 Hz, 2 H, H2), 7.15 (d, J = 8.5 Hz, 2 H, H3), 2.86 – 2.78 (m, 1 H, H5), 2.02 (dd, J = 16.4, 2.8 Hz, 1 H, H10a), 1.97 (t, J = 2.8 Hz, 1 H, H12), 1.93 (dd, J = 16.4, 2.8 Hz, 1 H, H10b), 1.79 (dd, J = 14.3, 8.6 Hz, 1 H, H6a), 1.63 (dd, J = 14.3, 4.2 Hz, 1 H, H6b), 1.22 (d, J = 7.0 Hz, 3 H, H13), 0.89 (s, 3 H, H8/H9), 0.85 (s, 3 H, H8/H9).
^{13}C NMR (150 MHz, $CDCl_3$): δ 147.5 (C4), 131.4 (C1), 128.6 (C2/C3), 128.5 (C2/C3), 82.6 (C11), 70.2 (C12), 48.9 (C6), 36.2 (C5), 34.2 (C7), 32.4 (C10), 27.5 (two superimposed s, C8 and C9), 26.0 (C13).
FTIR (ν_{max}, cm^{-1}): 3307 (w, alkyne CH), 2959 (m), 2926 (w), 1493 (m), 1470 (m), 1454 (w), 1410 (w), 1388 (w), 1367 (w), 1263 (w), 1179 (w), 1097 (m), 1014 (m), 825 (s), 771 (w).
HRMS (ESI): calculated for $C_{15}H_{20}Cl$ $[M+H]^+$ 232.1248, found 232.1243.
R_f = 0.35 (hexane).

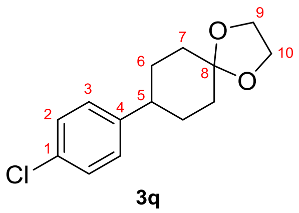

3q

8-(4-chlorophenyl)-1,4-dioxaspiro[4.5]decane (3q): Following the general procedure for protodeboronative coupling using 3-methoxy-3-methyl-4,9,12-trioxa-1,2-diazadispiro[4.2.4^{8}.2^{5}]tetradec-1-ene (0.242 g, 1.0 mmol) and 4-chlorophenylboronic acid (78.2 mg, 0.5 mmol), purified by silica gel column chromatography (eluent: 10% EtOAc/hexane) provided the title compound as a white crystalline solid (113.6 mg, 0.449 mmol, 90%), m.p. 82-84 °C.

^{1}H NMR (600 MHz, $CDCl_3$): δ 7.25 (d, *J* = 8.5 Hz, 2 H, H2), 7.16 (d, *J* = 8.5 Hz, 2 H, H3), 3.98 (s, 4 H, H9 and H10), 2.53 (tt, *J* = 12.0, 3.4 Hz, 1 H, H5), 1.89 – 1.80 (m, 4 H, H6a and H7a), 1.80 – 1.71 (m, 2 H, H6b), 1.71 – 1.64 (m, 2 H, H7b).
^{13}C NMR (150 MHz, $CDCl_3$): δ 145.1 (C4), 131.7 (C1), 128.5 (C2), 128.3 (C3), 108.5 (C8), 64.45 (C9/C10), 64.44 (C9/C10), 42.8 (C5), 35.2 (C7), 31.6 (C6).
FTIR (ν_{max}, cm^{-1}): 2938 (m), 2880 (m), 1493 (m), 1445 (w), 1410 (w), 1371 (w), 1335 (w), 1241 (w), 1172 (w), 1132 (m), 1102 (s), 1035 (m), 1014 (m), 926 (m), 867 (w), 827 (m).
HRMS (ESI): calculated for $C_{14}H_{18}O_2Cl$ [M+H]$^+$ 253.0990, found 253.0991.
R$_f$ = 0.27 (10% EtOAc/hexane).

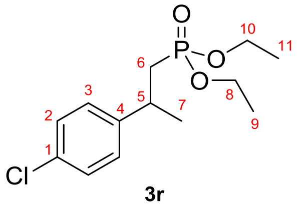

3r

Diethyl (2-(4-chlorophenyl)propyl)phosphonate (3r): Following the general procedure for protodeboronative coupling using diethyl ((5-methoxy-2,5-dimethyl-2,5-dihydro-1,3,4-oxadiazol-2-yl)methyl)phosphonate (0.280 g, 1.0 mmol) and 4-chlorophenylboronic acid (78.2 mg, 0.5 mmol), purified by silica gel column chromatography (eluent: 2% MeOH/CH_2Cl_2) provided the title compound as a colourless oil (92.8 mg, 0.319 mmol, 64%). Data are consistent with a reported example.[12]

^{1}H NMR (600 MHz, $CDCl_3$): δ 7.25 (d, *J* = 8.5 Hz, 2 H, H2), 7.14 (d, *J* = 8.5 Hz, 2 H, H3), 4.03 – 3.86 (m, 4 H, H8 and H10), 3.23 – 3.14 (m, 1 H, H5), 2.08 – 1.94 (m, 2 H, H6), 1.34 (d, *J* = 7.0 Hz, 3 H, H7), 1.23 (t, *J* = 7.1 Hz, 3 H, H9/H11), 1.19 (t, *J* = 7.1 Hz, 3 H, H9/H11).
^{13}C NMR (150 MHz, $CDCl_3$): δ 145.2 (d, *J* = 11.4 Hz, C4), 132.1 (C1), 128.7 (C2), 128.2 (C3), 61.5 (d, *J* = 6.6 Hz, C8/C10), 61.4 (d, *J* = 6.5 Hz, C8/C10), 34.4 (d, *J* = 139.0 Hz, C6), 34.3 (d, *J* = 3.6 Hz, C5), 23.7 (d, *J* = 10.1 Hz, C7), 16.5 (d, *J* = 6.1 Hz, C9/C11), 16.4 (d, *J* = 6.1 Hz, C9/C11).
^{31}P NMR (245 MHz, $CDCl_3$): δ 29.6 (s, 1 P, C6-P).
FTIR (ν_{max}, cm^{-1}): 2981 (w), 2906 (w), 1493 (w), 1456 (w), 1411 (w), 1392 (w), 1243 (m), 1163 (w), 1095 (m), 1052 (s), 1023 (s), 955 (s), 826 (m), 789 (m), 759 (w).
HRMS (ESI): calculated for $C_{13}H_{21}O_3PCl$ [M+H]$^+$ 291.0911, found 291.0922.
R$_f$ = 0.12 (2% MeOH/CH_2Cl_2).

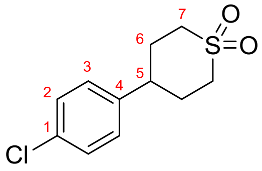

3s

4-(4-chlorophenyl)tetrahydro-2*H*-thiopyran 1,1-dioxide (3s): Following the general procedure for protodeboronative coupling using 3-methoxy-3-methyl-4-oxa-8-thia-1,2-diazaspiro[4.5]dec-1-ene 8,8-dioxide (0.234 g, 1.0 mmol) and 4-chlorophenylboronic acid (78.2 mg, 0.5 mmol), purified by silica gel column chromatography (eluent: 50% EtOAc/hexane) provided the title compound as a white crystalline solid (73.6 mg, 0.301 mmol, 60%), m.p. 208-210 °C (lit. m.p.[7] 208-209 °C).

^{1}H NMR (600 MHz, $CDCl_3$): δ 7.30 (d, J = 8.4 Hz, 2 H, H2), 7.16 (d, J = 8.4 Hz, 2 H, H3), 3.18 – 3.09 (m, 4 H, H7a and H7b), 2.77 (tt, J = 12.3, 3.1 Hz, 1 H, H5), 2.43 – 2.30 (m, 2 H, H6a), 2.23 – 2.14 (m, 2 H, H6b).
^{13}C NMR (150 MHz, $CDCl_3$): δ 141.7 (C4), 132.9 (C1), 129.1 (C2), 128.1 (C3), 51.5 (C7), 41.7 (C5), 31.4 (C6).
FTIR (ν_{max}, cm^{-1}): 2932 (w), 1495 (m), 1409 (w), 1342 (m), 1287 (s), 1245 (m), 1171 (w), 1122 (s), 1090 (m), 1050 (w), 1013 (w), 987 (w), 950 (w), 896 (w), 853 (w), 825 (m), 797 (w).
HRMS (ESI): calculated for $C_{11}H_{14}O_2SCl$ $[M+H]^+$ 245.0398, found 245.0392.
R$_f$ = 0.44 (50% EtOAc/hexane).

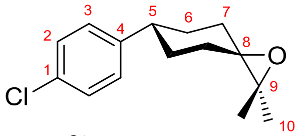

3t, *diastereomer A*

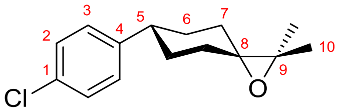

3t, *diastereomer B*

6-(4-chlorophenyl)-2,2-dimethyl-1-oxaspiro[2.5]octane (3t): Following the general procedure for protodeboronative coupling using 9-methoxy-2,2,9-trimethyl-1,10-dioxa-7,8-diazadispiro[2.2.4^6.2^3]dodec-7-ene (0.240 g, 1.0 mmol) and 4-chlorophenylboronic acid (78.2 mg, 0.5 mmol), purified by silica gel column chromatography (eluent: 10% Et_2O/hexane) provided the title compound as separable diastereomers (1:1) as a white crystalline solids (A: 47.2 mg, 0.188 mmol; B: 54.8 mg, 0.219 mmol; combined yield 81%), m.p. 91-92 °C for diastereomer A and m.p. 80-82 °C for diastereomer B.

Diastereomer A:
^{1}H NMR (600 MHz, $CDCl_3$): δ 7.26 (d, J = 8.5 Hz, 2 H, H2), 7.17 (d, J = 8.5 Hz, 2 H, H3), 2.62 – 2.53 (m, 1 H, H5), 1.94 – 1.85 (m, 2 H, H6a), 1.85 – 1.74 (m, 4 H, H6b and H7a), 1.75 – 1.64 (m, 2 H, H7b), 1.34 (s, 6 H, H10).
^{13}C NMR (150 MHz, $CDCl_3$): δ 145.4 (C4), 131.8 (C1), 128.6 (C2), 128.3 (C3), 65.1 (C8), 63.0 (C9), 43.2 (C5), 31.6 (C6), 30.3 (C7), 20.7 (C10).
FTIR (ν_{max}, cm^{-1}): 2926 (m), 1493 (s), 1441 (w), 1377 (m), 1274 (w), 1222 (w), 1178 (w), 1126 (m), 1094 (s), 1073 (m), 1013 (m), 970 (w), 926 (w), 880 (w), 862 (m), 827 (s).
HRMS (ESI): calculated for $C_{15}H_{19}OClNa$ $[M+Na]^+$ 273.1017, found 273.1010.
R$_f$ = 0.37 (10% Et_2O/hexane).

Diastereomer B:
^{1}H NMR (600 MHz, CDCl$_3$): δ 7.26 (d, J = 8.5 Hz, 2 H), 7.16 (d, J = 8.5 Hz, 2 H), 2.64 (tt, J = 12.1, 3.6 Hz, 1 H, H5), 2.05 – 1.97 (m, 2 H, H6a), 1.85 (td, J = 13.4, 3.5 Hz, 2 H, H7a), 1.81 – 1.74 (m, 2 H, H7b), 1.58 – 1.46 (m, 2 H, H6b), 1.40 (s, 6 H, H10).
^{13}C NMR (150 MHz, CDCl$_3$): δ 144.7 (C4), 131.9 (C1), 128.6 (C2), 128.2 (C3), 66.3 (C8), 62.5 (C9), 43.2 (C5), 33.6 (C6), 31.5 (C7), 21.0 (C10).
FTIR (ν_{max}, cm^{-1}): 3005 (w), 2979 (w), 2921 (m), 2856 (m), 1492 (m), 1470 (w), 1412 (w), 1377 (m), 1214 (w), 1168 (w), 1120 (m), 1087 (m), 1060 (w), 1015 (m), 985 (w), 896 (w), 857 (m), 821 (s).
HRMS (ESI): calculated for $C_{15}H_{19}OClNa$ [M+Na]$^+$ 273.1017, found 273.1012.
R$_f$ = 0.21 (10% Et_2O/hexane).

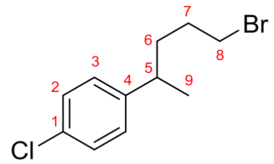

3u

1-(5-bromopentan-2-yl)-4-chlorobenzene (3u): Following the general procedure for protodeboronative coupling using 2-(3-bromopropyl)-5-methoxy-2,5-dimethyl-2,5-dihydro-1,3,4-oxadiazole (0.251 g, 1.0 mmol) and 4-chlorophenylboronic acid (78.2 mg, 0.5 mmol), purified by silica gel column chromatography (eluent: hexane) provided the title compound as a colourless oil (42.2 mg, 0.161 mmol, 32%).

^{1}H NMR (600 MHz, CDCl$_3$): δ 7.27 (d, J = 8.4 Hz, 2 H, H2), 7.11 (d, J = 8.4 Hz, 2 H, H3), 3.41 – 3.28 (m, 2 H, H8), 2.74 – 2.65 (m, 1 H, H5), 1.86 – 1.62 (m, 4 H, H6 and H7), 1.25 (d, J = 7.0 Hz, 3 H, H9).
^{13}C NMR (150 MHz, CDCl$_3$): δ 145.4 (C4), 131.8 (C1), 128.7 (C2), 128.4 (C3), 39.0 (C5), 36.8 (C6), 33.9 (C8), 31.0 (C7), 22.5 (C9).
FTIR (ν_{max}, cm^{-1}): 2961 (m), 2927 (w), 1596 (w), 1493 (m), 1455 (m), 1411 (w), 1377 (w), 1295 (w), 1245 (w), 1214 (w), 1091 (m), 1013 (m), 946 (w), 824 (s), 787 (w), 769 (w).
HRMS (ESI): calculated for $C_{11}H_{15}BrCl$ [M+H]$^+$ 261.0040, found 261.0045.
R$_f$ = 0.32 (hexane).

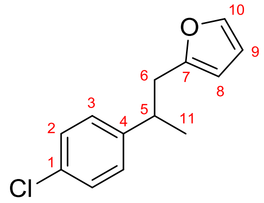

3v

2-(2-(4-chlorophenyl)propyl)furan (3v): Following the general procedure for protodeboronative coupling using 2-(furan-2-ylmethyl)-5-methoxy-2,5-dimethyl-2,5-dihydro-1,3,4-oxadiazole (0.210 g, 1.0 mmol) and 4-chlorophenylboronic acid (78.2 mg, 0.5 mmol), purified by silica gel column chromatography (eluent: hexane) provided the title compound as an orange oil (72.0 mg, 0.326 mmol, 65%).

^{1}H NMR (600 MHz, CDCl$_3$): δ 7.31 – 7.28 (m, 1 H, H10), 7.26 (d, J = 8.5 Hz, 2 H, H2), 7.11 (d, J = 8.5 Hz, 2 H, H3), 6.24 (dd, J = 3.1, 1.9 Hz, 1 H, H9), 5.88 (d, J = 3.1 Hz, 1 H, H8), 3.17 – 3.09 (m, 1 H, H5), 2.89 (dd, J = 14.9, 7.0 Hz, 1 H, H6a), 2.83 (dd, J = 14.9, 7.8 Hz, 1 H, H6b), 1.27 (d, J = 7.0 Hz, 3 H, H11).

^{13}C NMR (150 MHz, $CDCl_3$): δ 154.3 (C7), 145.0 (C4), 141.1 (C10), 131.9 (C1), 128.6 (C2), 128.4 (C3), 110.2 (C9), 106.4 (C8), 38.8 (C5), 36.9 (C6), 21.5 (C11).
FTIR (ν_{max}, cm^{-1}): 2964 (w), 2930 (w), 1795 (w), 1597 (w), 1493 (s), 1455 (w), 1411 (w), 1376 (w), 1210 (w), 1146 (m), 1093 (s), 1012 (s), 933 (m), 883 (w), 824 (s), 767 (w).
HRMS (ESI): calculated for $C_{13}H_{14}OCl$ $[M+H]^+$ 221.0728, found 221.0734.
***R**f* = 0.47 (hexane).

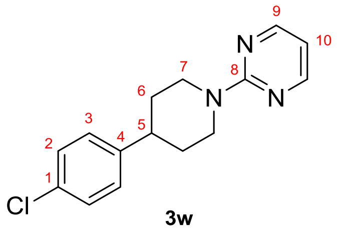

3w

2-(4-(4-chlorophenyl)piperidin-1-yl)pyrimidine (3w): Following the general procedure for protodeboronative coupling using 3-methoxy-3-methyl-8-(pyrimidin-2-yl)-4-oxa-1,2,8-triazaspiro[4.5]dec-1-ene (0.263 g, 1.0 mmol) and 4-chlorophenylboronic acid (78.2 mg, 0.5 mmol), purified by silica gel column chromatography (eluent: 20% EtOAc/hexane) provided the title compound as a white crystalline solid (77.4 mg, 0.283 mmol, 57%), m.p. 88-90 °C.

^{1}H NMR (600 MHz, $CDCl_3$): δ 8.31 (d, *J* = 4.7 Hz, 2 H, H9), 7.26 (d, *J* = 8.4 Hz, 2 H, H2), 7.14 (d, *J* = 8.4 Hz, 2 H, H3), 6.46 (t, *J* = 4.7 Hz, 1 H, H10), 4.97 – 4.88 (m, 2 H, H7a), 2.94 (td, *J* = 12.7, 2.4 Hz, 2 H, H7b), 2.77 (tt, *J* = 12.7, 3.5 Hz, 1 H, H5), 1.94 – 1.87 (m, 2 H, H6a), 1.65 (qd, *J* = 12.7, 4.2 Hz, 2 H, H6b).
^{13}C NMR (150 MHz, $CDCl_3$): δ 161.7 (C8), 157.9 (C9), 144.5 (C4), 132.0 (C1), 128.7 (C2), 128.3 (C3), 109.6 (C10), 44.5 (C7), 42.6 (C5), 33.2 (C6).
FTIR (ν_{max}, cm^{-1}): 3026 (w), 2991 (w), 2934 (w), 2849 (w), 1584 (s), 1545 (m), 1493 (s), 1459 (m), 1446 (m), 1410 (w), 1393 (w), 1361 (s), 1306 (w), 1273 (w), 1239 (w), 1180 (w), 1094 (w), 1012 (w), 979 (m), 947 (w), 827 (w), 796 (m).
HRMS (ESI): calculated for $C_{15}H_{17}N_3Cl$ $[M+H]^+$ 274.1106, found 274.1116.
***R**f* = 0.24 (20% EtOAc/hexane).

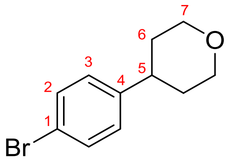

3x

4-(4-bromophenyl)tetrahydro-2*H*-pyran (3x): Following the general procedure for protodeboronative coupling using 3-methoxy-3-methyl-4,8-dioxa-1,2-diazaspiro[4.5]dec-1-ene (0.186 g, 1.0 mmol) and 4-bromophenylboronic acid (100.4 mg, 0.5 mmol), purified by silica gel column chromatography (eluent: 10% EtOAc/hexane) provided the title compound as a white amorphous solid (82.9 mg, 0.344 mmol, 69%), m.p. 72-74 °C (lit. m.p.[13] 61-62 °C). Data are consistent with a reported example.[13]

^{1}H NMR (600 MHz, $CDCl_3$): δ 7.43 (d, *J* = 8.4 Hz, 2 H, H2), 7.10 (d, *J* = 8.4 Hz, 2 H, H3), 4.12 – 4.03 (m, 2 H, H7a), 3.51 (td, *J* = 11.5, 2.8 Hz, 2 H, H7b), 2.72 (tt, *J* = 11.4, 4.5 Hz, 1 H, H5), 1.83 – 1.70 (m, 4 H, H6).
^{13}C NMR (150 MHz, $CDCl_3$): δ 144.9 (C4), 131.7 (C2), 128.6 (C3), 120.1 (C1), 68.4 (C7), 41.2 (C5), 33.9 (C6).

FTIR (ν_{max}, cm^{-1}): 2963 (w), 2939 (m), 2915 (m), 2871 (w), 2841 (m), 1590 (w), 1490 (m), 1440 (w), 1428 (w), 1408 (w), 1383 (m), 1300 (w), 1295 (w), 1263 (w), 1250 (w), 1236 (m), 1199 (w), 1126 (s), 1085 (s), 1075 (m), 1020 (m), 1008 (m), 979 (m), 910 (w), 896 (m), 837 (m), 819 (s).
HRMS (ESI): calculated for $C_{11}H_{14}OBr$ $[M+H]^+$ 241.0223, found 241.0226.
R_f = 0.25 (10% EtOAc/hexane).

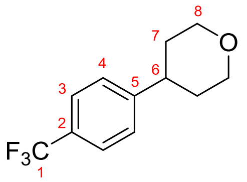

3y

4-(4-(trifluoromethyl)phenyl)tetrahydro-2*H*-pyran (3y): Following the general procedure for protodeboronative coupling using 3-methoxy-3-methyl-4,8-dioxa-1,2-diazaspiro[4.5]dec-1-ene (0.186 g, 1.0 mmol) and 4-(trifluoromethyl)phenylboronic acid (95.0 mg, 0.5 mmol), purified by silica gel column chromatography (eluent: 10% EtOAc/hexane) provided the title compound as a colourless oil (78.0 mg, 0.339 mmol, 68%). Data are consistent with a reported example.[14]

^{1}H NMR (600 MHz, $CDCl_3$): δ 7.57 (d, *J* = 8.2 Hz, 2 H, H3), 7.34 (d, *J* = 8.2 Hz, 2 H, H4), 4.16 – 4.04 (m, 2 H, H8a), 3.54 (td, *J* = 11.7, 2.3 Hz, 2 H, H8b), 2.83 (tt, *J* = 11.8, 4.0 Hz, 1 H, H6), 1.91 – 1.72 (m, 4 H, H7).
^{13}C NMR (150 MHz, $CDCl_3$): δ 149.9 (C5), 128.8 (q, *J* = 32.3 Hz, C2), 127.2 (C4), 125.6 (q, *J* = 3.8 Hz, C3), 124.4 (q, *J* = 271.8 Hz, C1), 68.3 (C8), 41.6 (C6), 33.8 (C7).
^{19}F NMR (376 MHz, $CDCl_3$): δ -62.4 (s, 3 F, F1).
FTIR (ν_{max}, cm^{-1}): 2939 (w), 2845 (w), 1619 (w), 1468 (w), 1444 (w), 1420 (w), 1387 (w), 1324 (s), 1259 (w), 1239 (w), 1190 (w), 1162 (m), 1116 (s), 1098 (s), 1086 (m), 1068 (s), 1016 (m), 982 (w), 955 (w), 913 (w), 896 (w), 837 (m), 802 (w), 761 (w).
HRMS (ESI): calculated for $C_{12}H_{14}F_3O$ $[M+H]^+$ 231.0991, found 231.0984.
R_f = 0.21 (10% EtOAc/hexane).

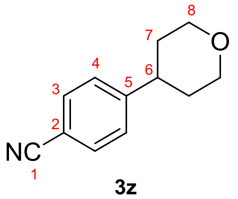

3z

4-(tetrahydro-2*H*-pyran-4-yl)benzonitrile (3z): Following the general procedure for protodeboronative coupling using 3-methoxy-3-methyl-4,8-dioxa-1,2-diazaspiro[4.5]dec-1-ene (0.186 g, 1.0 mmol) and 4-cyanophenylboronic acid (73.5 mg, 0.5 mmol), purified by silica gel column chromatography (eluent: 20% EtOAc/hexane) provided the title compound as a white amorphous solid (52.7 mg, 0.281 mmol, 56%), m.p. 58-60 °C. Compound has been prepared previously,[14] but NMR spectra were recorded in acetone-d_6.

^{1}H NMR (600 MHz, $CDCl_3$): δ 7.59 (d, *J* = 8.3 Hz, 2 H, H3), 7.31 (d, *J* = 8.3 Hz, 2 H, H4), 4.11 – 4.04 (m, 2 H, H8a), 3.51 (td, *J* = 11.5, 2.7 Hz, 2 H, H8b), 2.81 (tt, *J* = 11.5, 4.4 Hz, 1 H, H6), 1.84 – 1.70 (m, 4 H, H7).
^{13}C NMR (150 MHz, $CDCl_3$): δ 151.2 (C5), 132.5 (C3), 127.7 (C4), 119.0 (C1), 110.3 (C2), 68.1 (C8), 41.8 (C6), 33.5 (C7).

FTIR (ν_{max}, cm^{-1}): 2939 (m), 2842 (m), 2226 (m, C≡N), 1608 (m), 1505 (m), 1467 (w), 1443 (m), 1417 (w), 1387 (m), 1292 (w), 1265 (w), 1239 (m), 1200 (w), 1179 (w), 1124 (s), 1100 (m), 1084 (s), 1018 (m), 982 (m), 913 (w), 895 (m), 838 (s), 808 (m).
HRMS (ESI): calculated for $C_{12}H_{14}NO$ $[M+H]^+$ 188.1070, found 188.1086.
***R**$_f$* = 0.22 (20% EtOAc/hexane).

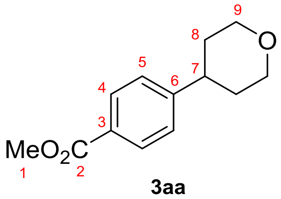

3aa

Methyl 4-(tetrahydro-2*H*-pyran-4-yl)benzoate (3aa): Following the general procedure for protodeboronative coupling using 3-methoxy-3-methyl-4,8-dioxa-1,2-diazaspiro[4.5]dec-1-ene (0.186 g, 1.0 mmol) and 4-methoxycarbonylphenylboronic acid (90.0 mg, 0.5 mmol), purified by silica gel column chromatography (eluent: 5% Et_2O/CH_2Cl_2) provided the title compound as a white crystalline solid (63.4 mg, 0.306 mmol, 61%), m.p. 71-72 °C (lit. m.p.[15] 74-75 °C). Data are consistent with a reported example.[15]

^{1}H NMR (600 MHz, $CDCl_3$): δ 7.98 (d, *J* = 8.3 Hz, 2 H, H4), 7.29 (d, *J* = 8.3 Hz, 2 H, H5), 4.08 (dd, *J* = 11.5, 4.2 Hz, 2 H, H9a), 3.90 (s, 3 H, H1), 3.53 (td, *J* = 11.5, 2.3 Hz, 2 H, H9b), 2.81 (tt, *J* = 11.8, 4.0 Hz, 1 H, H7), 1.88 – 1.73 (m, 4 H, H8).
^{13}C NMR (150 MHz, $CDCl_3$): δ 167.1 (C2), 151.2 (C6), 130.0 (C4), 128.4 (C3), 126.9 (C5), 68.3 (C9), 52.1 (C1), 41.8 (C7), 33.7 (C8).
FTIR (ν_{max}, cm^{-1}): 2964 (w), 2933 (w), 2907 (w), 2853 (w), 1719 (s, C=O), 1610 (w), 1573 (w), 1440 (m), 1415 (w), 1390 (w), 1362 (w), 1289 (m), 1278 (m), 1236 (w), 1198 (w), 1180 (w), 1168 (w), 1130 (w), 1110 (m), 1097 (m), 1082 (m), 1018 (m), 978 (w), 962 (w), 915 (w), 894 (w), 858 (w), 842 (w), 825 (w), 764 (m).
HRMS (ESI): calculated for $C_{13}H_{17}O_3$ $[M+H]^+$ 221.1172, found 221.1179.
***R**$_f$* = 0.38 (5% Et_2O/CH_2Cl_2).

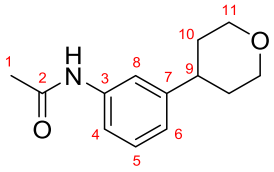

3ab

***N*-(3-(tetrahydro-2*H*-pyran-4-yl)phenyl)acetamide (3ab):** Following the general procedure for protodeboronative coupling using 3-methoxy-3-methyl-4,8-dioxa-1,2-diazaspiro[4.5]dec-1-ene (0.186 g, 1.0 mmol) and 3-acetamidophenylboronic acid (89.5 mg, 0.5 mmol), purified by silica gel column chromatography (eluent: 5% $MeOH/CH_2Cl_2$) provided the title compound as an off-white amorphous solid (96.9 mg, 0.442 mmol, 88%), m.p. 128-131 °C.

^{1}H NMR (600 MHz, $CDCl_3$): δ 7.58 (br s, 1 H, NH), 7.41 (s, 1 H, H8), 7.35 (d, *J* = 8.0 Hz, 1 H, H4), 7.28 – 7.21 (m, 1 H, H5), 6.96 (d, *J* = 7.6 Hz, 1 H, H6), 4.06 (dd, *J* = 11.3, 3.5 Hz, 2 H, H11a), 3.51 (td, *J* = 11.3, 2.4 Hz, 2 H, H11b), 2.73 (tt, *J* = 11.6, 4.2 Hz, 1 H, H9), 2.16 (s, 3 H, H1), 1.85 – 1.70 (m, 4 H, H10).
^{13}C NMR (150 MHz, $CDCl_3$): δ 168.6 (C2), 147.0 (C7), 138.3 (C3), 129.2 (C5), 122.7 (C6), 118.4 (C8), 118.0 (C4), 68.4 (C11), 41.6 (C9), 34.0 (C10), 24.7 (C1).

FTIR (ν_{max}, cm^{-1}): 3290 (w, NH), 2938 (m), 2845 (m), 1668 (s, C=O), 1611 (s), 1594 (m), 1555 (s), 1490 (m), 1442 (m), 1372 (m), 1303 (m), 1259 (m), 1238 (m), 1130 (m), 1084 (m), 1015 (m), 980 (w), 917 (w), 869 (w), 824 (w), 791 (w).
HRMS (ESI): calculated for $C_{13}H_{18}NO_2$ $[M+H]^+$ 220.1332, found 220.1328.
$\boldsymbol{R_f}$ = 0.27 (5% MeOH/CH_2Cl_2).

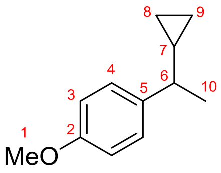

3ac

1-(1-cyclopropylethyl)-4-methoxybenzene (3ac): Following a modified version of the general procedure for protodeboronative coupling using 2-cyclopropyl-5-methoxy-2,5-dimethyl-2,5-dihydro-1,3,4-oxadiazole (0.236 g, 1.0 mmol) and 4-methoxyphenylboronic acid (76.0 mg, 0.5 mmol) – the output of the reactor was heated in the sealed vial at 75 °C for 16 h. Purification by silica gel column chromatography (eluent: 2% EtOAc/hexane) provided the title compound as a colourless oil (49.0 mg, 0.278 mmol, 56%).

^{1}H NMR (600 MHz, $CDCl_3$): δ 7.20 (d, *J* = 8.6 Hz, 2 H, H4), 6.87 (d, *J* = 8.6 Hz, 2 H, H3), 3.81 (s, 3 H, H1), 2.02 – 1.92 (m, 1 H, H6), 1.33 (d, *J* = 7.1 Hz, 3 H, H10), 0.97 – 0.87 (m, 1 H, H7), 0.59 – 0.51 (m, 1 H, H8a), 0.48 – 0.40 (m, 1 H, H9a), 0.24 – 0.12 (m, 2 H, H8b and H9b).
^{13}C NMR (150 MHz, $CDCl_3$): δ 157.9 (C2), 139.6 (C5), 127.9 (C4), 113.7 (C3), 55.4 (C1), 43.8 (C6), 21.8 (C10), 18.9 (C7), 4.7 (C8/C9), 4.4 (C8/C9).
FTIR (ν_{max}, cm^{-1}): 3076 (w), 2998 (w), 2959 (w), 2934 (w), 2835 (w), 1612 (w), 1584 (w), 1510 (s), 1455 (w), 1442 (w), 1428 (w), 1369 (w), 1333 (w), 1303 (w), 1288 (w), 1268 (w), 1241 (s), 1178 (m), 1111 (w), 1073 (w), 1030 (m), 1015 (m), 972 (w), 925 (w), 830 (m), 806 (m), 769 (w).
HRMS (ESI): calculated for $C_{12}H_{17}O$ $[M+H]^+$ 177.1274, found 177.1266.
$\boldsymbol{R_f}$ = 0.37 (2% EtOAc/hexane).

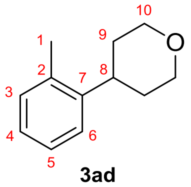

3ad

4-(*o*-tolyl)tetrahydro-2*H*-pyran (3ad): Following a modified version of the general procedure for protodeboronative coupling using 3-methoxy-3-methyl-4,8-dioxa-1,2-diazaspiro[4.5]dec-1-ene (0.186 g, 1.0 mmol) and *o*-tolylboronic acid (68.0 mg, 0.5 mmol) – the output of the reactor was heated in the sealed vial at 75 °C for 16 h. Purification by silica gel column chromatography (eluent: 5% EtOAc/hexane) provided the title compound as a colourless oil (60.9 mg, 0.346 mmol, 69%). Data are consistent with a reported example.[14]

^{1}H NMR (600 MHz, $CDCl_3$): δ 7.27 – 7.20 (m, 2 H, H5 and H6), 7.18 (d, *J* = 6.9 Hz, 1 H, H3), 7.15 – 7.11 (m, 1 H, H4), 4.12 (dd, *J* = 11.8, 4.4 Hz, 2 H, H10a), 3.58 (td, *J* = 11.8, 1.9 Hz, 2 H, H10b), 3.00 (tt, *J* = 12.0, 3.6 Hz, 1 H, H8), 2.38 (s, 3 H, H1), 1.90 – 1.80 (m, 2 H, H9a), 1.74 – 1.67 (m, 2 H, H9b).
^{13}C NMR (150 MHz, $CDCl_3$): δ 143.8 (C7), 135.2 (C2), 130.5 (C3), 126.5 (C5), 126.1 (C4), 125.6 (C6), 68.8 (C10), 37.5 (C8), 33.3 (C9), 19.4 (C1).

FTIR (ν_{max}, cm^{-1}): 3020 (w), 2944 (m), 2840 (m), 1605 (w), 1493 (m), 1462 (m), 1442 (w), 1386 (m), 1367 (w), 1297 (w), 1257 (w), 1235 (m), 1217 (w), 1173 (w), 1132 (m), 1121 (m), 1091 (m), 1052 (w), 1021 (m), 1011 (m), 980 (m), 940 (w), 895 (m), 834 (m), 809 (w), 779 (w), 750 (s).
HRMS (ESI): calculated for $C_{12}H_{17}O$ $[M+H]^+$ 177.1274, found 177.1277.
R_f = 0.22 (5% EtOAc/hexane).

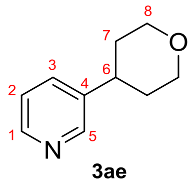

3ae

3-(tetrahydro-2*H*-pyran-4-yl)pyridine (3ae): Following the general procedure for protodeboronative coupling using 3-methoxy-3-methyl-4,8-dioxa-1,2-diazaspiro[4.5]dec-1-ene (0.186 g, 1.0 mmol) and 3-pyridinylboronic acid (61.5 mg, 0.5 mmol), purified by silica gel column chromatography (eluent: 5% MeOH/CH_2Cl_2) provided the title compound as a yellow oil (24.6 mg, 0.151 mmol, 30%). Compound has been prepared previously,[14] but NMR spectra were recorded in acetone-d_6.

^{1}H NMR (600 MHz, $CDCl_3$): δ 8.51 (br s, 1 H, H5), 8.47 (d, *J* = 3.9 Hz, 1 H, H1), 7.55 (dt, *J* = 7.9, 1.8 Hz, 1 H, H3), 7.28 – 7.24 (m, 1 H, H2), 4.12 – 4.07 (m, 2 H, H8a), 3.54 (td, *J* = 11.7, 2.4 Hz, 2 H, H8b), 2.80 (tt, *J* = 11.8, 4.2 Hz, 1 H, H6), 1.87 – 1.74 (m, 4 H, H7).
^{13}C NMR (150 MHz, $CDCl_3$): δ 148.9 (C5), 147.9 (C1), 141.0 (C4), 134.3 (C3), 123.7 (C2), 68.3 (C8), 39.2 (C6), 33.7 (C7).
FTIR (ν_{max}, cm^{-1}): 2938 (m), 2846 (m), 1576 (w), 1480 (w), 1443 (w), 1426 (m), 1388 (m), 1275 (w), 1263 (w), 1239 (m), 1182 (w), 1126 (s), 1098 (m), 1085 (s), 1050 (w), 1020 (s), 982 (m), 895 (m), 840 (m), 812 (w).
HRMS (ESI): calculated for $C_{10}H_{14}NO$ $[M+H]^+$ 164.1070, found 164.1062.
R_f = 0.37 (5% MeOH/CH_2Cl_2).

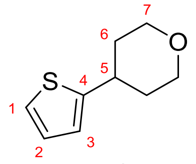

3af

4-(thiophen-2-yl)tetrahydro-2*H*-pyran (3af): Following the general procedure for protodeboronative coupling using 3-methoxy-3-methyl-4,8-dioxa-1,2-diazaspiro[4.5]dec-1-ene (0.186 g, 1.0 mmol) and 2-thienylboronic acid (64.0 mg, 0.5 mmol), purified by silica gel column chromatography (eluent: 30% CH_2Cl_2/hexane) provided the title compound as a colourless oil (32.3 mg, 0.192 mmol, 38%).

^{1}H NMR (600 MHz, $CDCl_3$): δ 7.15 (dd, *J* = 5.1, 1.1 Hz, 1 H, H1), 6.95 (dd, *J* = 5.1, 3.5 Hz, 1 H, H2), 6.85 – 6.82 (m, 1 H, H3), 4.08 – 4.02 (m, 2 H, H7a), 3.52 (td, *J* = 11.8, 2.1 Hz, 2 H, H7b), 3.07 (tt, *J* = 11.8, 3.9 Hz, 1 H, H5), 1.97 – 1.92 (m, 2 H, H6a), 1.87 – 1.79 (m, 2 H, H6b).
^{13}C NMR (150 MHz, $CDCl_3$): δ 150.2 (C4), 126.8 (C2), 122.8 (C1), 122.3 (C3), 68.1 (C7), 36.7 (C5), 35.1 (C6).
FTIR (ν_{max}, cm^{-1}): 2937 (m), 2842 (m), 1533 (w), 1466 (w), 1442 (m), 1387 (m), 1311 (w), 1257 (m), 1240 (m), 1125 (s), 1088 (s), 1015 (m), 981 (m), 879 (m), 852 (m), 821 (m).
HRMS (ESI): calculated for $C_9H_{13}OS$ $[M+H]^+$ 169.0682, found 169.0682.
R_f = 0.24 (30% CH_2Cl_2/hexane).

4.2. Oxidative couplings

General procedure for oxidative coupling: A solution of the appropriate oxadiazoline (1.0 mmol, 2 equiv.), boronic acid (0.5 mmol, 1.0 equiv.) and DIPEA (0.17 mL, 1.0 mmol, 2 equiv.) in CH_2Cl_2 (10 mL) was pumped at a flow rate of 0.125 mL min^{-1} through a Vapourtec UV-150 photochemical reactor (10 mL reactor volume, FEP tubing), irradiated by a 310 nm UV lamp (9 W output power), held at 10 °C and the reactor output was monitored using a FlowIR® device (SiComp head, 2100-2000 cm^{-1} and 1750-1700 cm^{-1}). After 80 min once the reaction mixture has fully been taken up by the pump, the input was swapped to CH_2Cl_2 solvent. When the FlowIR® showed that the reaction plug was exiting the output stream (by monitoring the MeOAc C=O stretch at 1750-1700 cm^{-1}), the reaction plug was directed into a round-bottomed flask and stirred for 16 h under air. The mixture was then evaporated under reduced pressure and the residue purified by silica gel column chromatography.

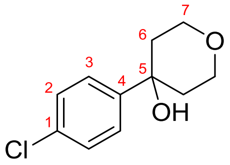

4a

4-(4-chlorophenyl)tetrahydro-2*H*-pyran-4-ol (4a): Following the general procedure for oxidative coupling using 3-methoxy-3-methyl-4,8-dioxa-1,2-diazaspiro[4.5]dec-1-ene (0.186 g, 1.0 mmol) and 4-chlorophenylboronic acid (78.2 mg, 0.5 mmol), purified by silica gel column chromatography (eluent: 40% EtOAc/hexane) provided the title compound as a white crystalline solid (80.6 mg, 0.378 mmol, 76%), m.p. 69-71 °C (lit. m.p.[16] 77-78 °C). Data are consistent with a reported example.[16]

^{1}H NMR (600 MHz, $CDCl_3$): δ 7.41 (d, *J* = 8.8 Hz, 2 H, H2), 7.33 (d, *J* = 8.8 Hz, 2 H, H3), 3.89 (td, *J* = 11.8, 2.1 Hz, 2 H, H7a), 3.86 – 3.81 (m, 2 H, H7b), 2.14 – 2.06 (m, 2 H, H6a), 1.99 (br s, 1 H, OH), 1.67 – 1.60 (m, 2 H, H6b).
^{13}C NMR (150 MHz, $CDCl_3$): δ 146.8 (C4), 133.1 (C1), 128.7 (C3), 126.1 (C2), 70.5 (C5), 63.9 (C7), 38.8 (C6).
FTIR (ν_{max}, cm^{-1}): 3399 (br m, OH), 2955 (m), 2871 (m), 1595 (w), 1494 (m), 1467 (w), 1388 (m), 1302 (w), 1238 (m), 1222 (w), 1125 (m), 1095 (s), 1033 (m), 1013 (s), 965 (w), 915 (w), 826 (s), 798 (w).
HRMS (ESI): calculated for $C_{11}H_{13}O_2ClNa$ $[M+Na]^+$ 235.0496, found 235.0505.
R_f = 0.28 (40% EtOAc/hexane).

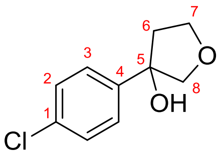

4b

3-(4-chlorophenyl)tetrahydrofuran-3-ol (4b): Following the general procedure for oxidative coupling using 3-methoxy-3-methyl-4,7-dioxa-1,2-diazaspiro[4.4]non-1-ene (0.172 g, 1.0 mmol) and 4-chlorophenylboronic acid (78.2 mg, 0.5 mmol), purified by silica gel column chromatography (eluent: 40% EtOAc/hexane) provided the title compound as a colourless oil (57.0 mg, 0.287 mmol, 57%).

^{1}H NMR (600 MHz, $CDCl_3$): δ 7.42 (d, *J* = 8.6 Hz, 2 H, H2), 7.33 (d, *J* = 8.6 Hz, 2 H, H3), 4.19 (td, *J* = 8.8, 7.0 Hz, 1 H, H7a), 4.10 (td, *J* = 8.8, 3.5 Hz, 1 H, H7b), 3.93 (dd, *J* = 9.5, 1.3 Hz, 1 H, H8a), 3.84 (d, *J* = 9.5 Hz, 1 H, H8b), 2.56 (br s, 1 H, OH), 2.37 (dt, *J* = 13.1, 8.8 Hz, 1 H, H6a), 2.24 (dddd, *J* = 13.1, 7.0, 3.5, 1.3 Hz, 1 H, H6b).
^{13}C NMR (150 MHz, $CDCl_3$): δ 140.9 (C4), 133.5 (C1), 128.7 (C3), 126.9 (C2), 81.6 (C5), 80.5 (C8), 68.1 (C7), 42.2 (C6).
FTIR (ν_{max}, cm^{-1}): 3386 (br m, OH), 2955 (w), 2882 (w), 1599 (w), 1493 (m), 1440 (w), 1400 (w), 1359 (w), 1252 (w), 1136 (m), 1094 (s), 1057 (s), 1013 (s), 976 (m), 923 (m), 892 (m), 823 (s), 775 (m).
HRMS (ESI): calculated for $C_{10}H_{11}O_2ClNa$ $[M+Na]^+$ 221.0340, found 221.0334.
R_f = 0.28 (40% EtOAc/hexane).

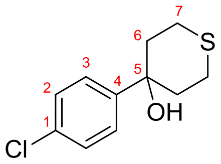

4c

4-(4-chlorophenyl)tetrahydro-2*H*-thiopyran-4-ol (4c): Following the general procedure for oxidative coupling using 3-methoxy-3-methyl-4-oxa-8-thia-1,2-diazaspiro[4.5]dec-1-ene (0.202 g, 1.0 mmol) and 4-chlorophenylboronic acid (78.2 mg, 0.5 mmol), purified by silica gel column chromatography (eluent: 30% Et_2O/hexane) provided the title compound as an off-white crystalline solid (95.2 mg, 0.416 mmol, 83%), m.p. 88-90 °C (lit. m.p.[16] 86-87 °C). Data are consistent with a reported example.[16]

^{1}H NMR (600 MHz, $CDCl_3$): δ 7.40 (d, *J* = 8.7 Hz, 2 H, H2), 7.32 (d, *J* = 8.7 Hz, 2 H, H3), 3.24 – 3.12 (m, 2 H, H7a), 2.51 – 2.42 (m, 2 H, H7b), 2.18 – 2.08 (m, 2 H, H6a), 2.02 – 1.94 (m, 2 H, H6b), 1.60 (br s, 1 H, OH).
^{13}C NMR (150 MHz, $CDCl_3$): δ 147.7 (C4), 133.0 (C1), 128.6 (C3), 125.9 (C2), 71.9 (C5), 39.6 (C6), 24.2 (C7).
FTIR (ν_{max}, cm^{-1}): 3428 (br m, OH), 2916 (m), 1596 (w), 1494 (s), 1424 (m), 1399 (w), 1304 (w), 1275 (m), 1227 (m), 1179 (w), 1136 (w), 1095 (s), 1067 (s), 1027 (w), 1013 (s), 968 (s), 927 (s), 881 (w), 827 (s), 777 (w).
HRMS (ESI): calculated for $C_{11}H_{13}OSClNa$ $[M+Na]^+$ 251.0268, found 251.0270.
R_f = 0.22 (30% Et_2O/hexane).

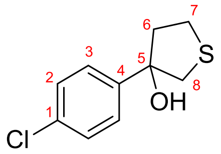

4d

3-(4-chlorophenyl)tetrahydrothiophen-3-ol (4d): Following the general procedure for oxidative coupling using 3-methoxy-3-methyl-4-oxa-7-thia-1,2-diazaspiro[4.4]non-1-ene (0.188 g, 1.0 mmol) and 4-chlorophenylboronic acid (78.2 mg, 0.5 mmol), purified by silica gel column chromatography (eluent: 30% Et_2O/hexane) provided the title compound as a colourless oil (62.6 mg, 0.292 mmol, 58%).

^{1}H NMR (600 MHz, $CDCl_3$): δ 7.47 (d, *J* = 8.6 Hz, 2 H, H2), 7.33 (d, *J* = 8.6 Hz, 2 H, H3), 3.20 (d, *J* = 11.6 Hz, 1 H, H8a), 3.13 (td, *J* = 10.5, 7.0 Hz, 1 H, H7a), 3.05 (ddd, *J* = 10.5, 7.7, 2.5 Hz, 1 H, H7b), 2.96 (dd, *J* = 11.6, 1.4 Hz, 1 H, H8b), 2.71 (br s, 1 H, OH), 2.33 – 2.23 (m, 2 H, H6).

^{13}C NMR (150 MHz, CDCl$_3$): δ 141.1 (C4), 133.5 (C1), 128.6 (C3), 126.8 (C2), 83.5 (C5), 45.5 (C8), 43.4 (C6), 29.0 (C7).
FTIR (ν_{max}, cm^{-1}): 3418 (br w, OH), 2938 (w), 1706 (w), 1596 (w), 1493 (m), 1427 (w), 1400 (m), 1358 (m), 1269 (w), 1208 (m), 1176 (w), 1093 (s), 1065 (w), 1035 (s), 1013 (s), 984 (w), 957 (m), 935 (m), 822 (s).
HRMS (ESI): calculated for $C_{10}H_{11}OSClNa$ $[M+Na]^+$ 237.0111, found 237.0110.
R$_f$ = 0.29 (30% Et_2O/hexane).

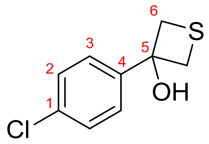

4e

3-(4-chlorophenyl)thietan-3-ol (4e): Following the general procedure for oxidative coupling using 7-methoxy-7-methyl-8-oxa-2-thia-5,6-diazaspiro[3.4]oct-5-ene (0.174 g, 1.0 mmol) and 4-chlorophenylboronic acid (78.2 mg, 0.5 mmol), purified by silica gel column chromatography (eluent: 10% EtOAc/hexane) provided the title compound as a yellow oil (20.0 mg, 0.100 mmol, 20%).

^{1}H NMR (600 MHz, CDCl$_3$): δ 7.63 (d, J = 8.6 Hz, 2 H, H2), 7.38 (d, J = 8.6 Hz, 2 H, H3), 3.59 (s, 4 H, H6), 2.86 (br s, 1 H, OH).
^{13}C NMR (150 MHz, CDCl$_3$): δ 143.1 (C4), 134.0 (C1), 128.9 (C3), 125.9 (C2), 78.8 (C5), 42.8 (C6).
FTIR (ν_{max}, cm^{-1}): 3365 (br w, OH), 2987 (w), 2939 (w), 2850 (w), 1682 (w), 1598 (w), 1575 (w), 1491 (m), 1426 (w), 1400 (w), 1368 (w), 1304 (w), 1265 (w), 1212 (m), 1175 (m), 1127 (w), 1092 (m), 1053 (m), 1013 (m), 954 (m), 880 (w), 826 (s), 771 (w).
HRMS (ESI): calculated for $C_9H_9OSClNa$ $[M+Na]^+$ 222.9955, found 222.9948.
R$_f$ = 0.21 (10% EtOAc/hexane).

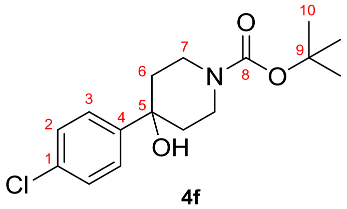

4f

***tert*-butyl 4-(4-chlorophenyl)-4-hydroxypiperidine-1-carboxylate (4f):** Following the general procedure for oxidative coupling using *tert*-butyl 3-methoxy-3-methyl-4-oxa-1,2,8-triazaspiro[4.5]dec-1-ene-8-carboxylate (0.285 g, 1.0 mmol) and 4-chlorophenylboronic acid (78.2 mg, 0.5 mmol), purified by silica gel column chromatography (eluent: 20% → 30% EtOAc/hexane) provided the title compound as a colourless gum (125.1 mg, 0.401 mmol, 80%).

^{1}H NMR (600 MHz, CDCl$_3$): δ 7.40 (d, J = 8.7 Hz, 2 H, H2), 7.31 (d, J = 8.7 Hz, 2 H, H3), 3.99 (br s, 2 H, H7a), 3.20 (br s, 2 H, H7b), 2.02 (br s, 1 H, OH), 1.93 (br s, 2 H, H6a), 1.72 – 1.66 (m, 2 H, H6b), 1.46 (s, 9 H, H10).
^{13}C NMR (150 MHz, CDCl$_3$): δ 155.0 (C8), 146.8 (C4), 133.1 (C1), 128.6 (C3), 126.2 (C2), 79.8 (C9), 71.4 (C5), 40.7 – 38.9 (br, C7), 38.5 – 37.6 (br, C6), 28.6 (C10).
FTIR (ν_{max}, cm^{-1}): 3431 (br w, OH), 2975 (w), 2927 (w), 1662 (s, C=O), 1479 (m), 1425 (m), 1392 (m), 1366 (m), 1319 (w), 1277 (m), 1248 (m), 1216 (m), 1163 (s), 1138 (m), 1093 (m), 1029 (m), 1013 (m), 958 (w), 909 (m), 861 (m), 824 (m), 795 (w), 769 (m).

HRMS (ESI): calculated for $C_{16}H_{22}NO_3ClNa$ $[M+Na]^+$ 334.1180, found 334.1193.
***R**_f* = 0.14 (20% EtOAc/hexane).

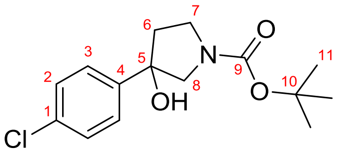

4g

***tert*-butyl 3-(4-chlorophenyl)-3-hydroxypyrrolidine-1-carboxylate (4g):** Following the general procedure for oxidative coupling using *tert*-butyl 3-methoxy-3-methyl-4-oxa-1,2,7-triazaspiro[4.4]non-1-ene-7-carboxylate (0.271 g, 1.0 mmol) and 4-chlorophenylboronic acid (78.2 mg, 0.5 mmol), purified by silica gel column chromatography (eluent: 60% Et_2O/hexane) provided the title compound as a colourless gum (82.2 mg, 0.276 mmol, 55%).

^{1}H NMR (600 MHz, $CDCl_3$): δ 7.41 and 7.39 (two d from rotamers, *J* = 8.6 Hz, 2 H, H2), 7.31 (two superimposed d from rotamers, *J* = 8.6 Hz, 2 H, H3), 3.72 – 3.45 (m, 4 H, H7 and H8), 2.90 and 2.88 (two br s from rotamers, 1 H, OH), 2.31 – 2.07 (m, 2 H, H6), 1.44 and 1.43 (two s from rotamers, 9 H, H11).
^{13}C NMR (150 MHz, $CDCl_3$): δ 154.9 and 154.7 (rotameric, C9), 141.7 and 141.6 (rotameric, C4), 133.6 (C1), 128.7 (C3), 126.9 (C2), 80.2 and 79.4 (C5), 79.8 (C10), 59.7 and 58.9 (C8), 45.2 and 44.7 (C7), 39.9 and 39.0 (C6), 28.6 (C11).
FTIR (ν_{max}, cm^{-1}): 3405 (br w, OH), 2978 (w), 2892 (w), 1668 (s, C=O), 1493 (w), 1478 (w), 1416 (s), 1367 (m), 1254 (w), 1170 (m), 1136 (s), 1094 (m), 1015 (w), 924 (w), 878 (w), 827 (m), 757 (w).
HRMS (ESI): calculated for $C_{15}H_{20}NO_3ClNa$ $[M+Na]^+$ 320.1024, found 320.1025.
***R**_f* = 0.27 (60% Et_2O/hexane).

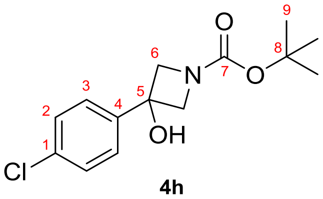

4h

***tert*-butyl 3-(4-chlorophenyl)-3-hydroxyazetidine-1-carboxylate (4h):** Following the general procedure for oxidative coupling using *tert*-butyl 7-methoxy-7-methyl-8-oxa-2,5,6-triazaspiro[3.4]oct-5-ene-2-carboxylate (0.257 g, 1.0 mmol) and 4-chlorophenylboronic acid (78.2 mg, 0.5 mmol), purified by silica gel column chromatography (eluent: 30% EtOAc/hexane) provided the title compound as a white amorphous solid (60.3 mg, 0.213 mmol, 43%), m.p. 128-131 °C (lit. m.p.[17] 139.0-140.6 °C). Data are consistent with a reported example.[17]

^{1}H NMR (600 MHz, $CDCl_3$): δ 7.43 (d, *J* = 8.7 Hz, 2 H, H2), 7.34 (d, *J* = 8.7 Hz, 2 H, H3), 4.15 (s, 4 H, H6), 3.65 (br s, 1 H, OH), 1.44 (s, 9 H, H9).
^{13}C NMR (150 MHz, $CDCl_3$): δ 156.6 (C7), 142.1 (C4), 133.7 (C1), 128.8 (C3), 126.2 (C2), 80.3 (C8), 70.8 (C5), 65.5 – 63.5 (br, C6), 28.5 (C9).
FTIR (ν_{max}, cm^{-1}): 3372 (br w, OH), 2978 (w), 2882 (w), 1675 (s, C=O), 1493 (m), 1478 (m), 1416 (s), 1367 (s), 1250 (m), 1160 (s), 1119 (m), 1094 (m), 1013 (m), 936 (w), 860 (w), 826 (w), 772 (w).
HRMS (ESI): calculated for $C_{14}H_{18}NO_3ClNa$ $[M+Na]^+$ 306.0867, found 306.0855.
***R**_f* = 0.38 (30% EtOAc/hexane).

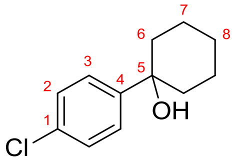

4i

1-(4-chlorophenyl)cyclohexan-1-ol (4i): Following the general procedure for oxidative coupling using 3-methoxy-3-methyl-4-oxa-1,2-diazaspiro[4.5]dec-1-ene (0.184 g, 1.0 mmol) and 4-chlorophenylboronic acid (78.2 mg, 0.5 mmol), purified by silica gel column chromatography (eluent: 10% EtOAc/hexane) provided the title compound as a white crystalline solid (82.2 mg, 0.390 mmol, 78%), m.p. 74-76 °C (lit. m.p.[18] 77 °C). Data are consistent with a reported example.[19]

^{1}H NMR (600 MHz, CDCl$_3$): δ 7.43 (d, *J* = 8.7 Hz, 2 H, H2), 7.30 (d, *J* = 8.7 Hz, 2 H, H3), 1.82 – 1.72 (m, 7 H, H6, H7a and H8a), 1.69 (br s, 1 H, OH), 1.67 – 1.60 (m, 2 H, H7b), 1.36 – 1.21 (m, 1 H, H8b).
^{13}C NMR (150 MHz, CDCl$_3$): δ 148.1 (C4), 132.5 (C1), 128.4 (C3), 126.3 (C2), 73.0 (C5), 38.9 (C6), 25.5 (C8), 22.2 (C7).
FTIR (ν_{max}, cm^{-1}): 3380 (br m, OH), 2933 (s), 2857 (m), 1595 (w), 1494 (m), 1448 (m), 1399 (w), 1257 (w), 1209 (w), 1174 (w), 1134 (w), 1095 (m), 1011 (m), 973 (m), 903 (w), 849 (w), 821 (s).
HRMS (ESI): calculated for $C_{12}H_{15}OClNa$ $[M+Na]^+$ 233.0704, found 233.0700.
R$_f$ = 0.24 (10% EtOAc/hexane).

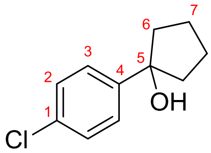

4j

1-(4-chlorophenyl)cyclopentan-1-ol (4j): Following the general procedure for oxidative coupling using 3-methoxy-3-methyl-4-oxa-1,2-diazaspiro[4.5]dec-1-ene (0.184 g, 1.0 mmol) and 4-chlorophenylboronic acid (78.2 mg, 0.5 mmol), purified by silica gel column chromatography (eluent: 10% EtOAc/hexane) provided the title compound as a colourless oil (63.5 mg, 0.323 mmol, 65%).

^{1}H NMR (600 MHz, CDCl$_3$): δ 7.42 (d, *J* = 8.6 Hz, 2 H, H2), 7.30 (d, *J* = 8.6 Hz, 2 H, H3), 2.03 – 1.92 (m, 6 H, H6 and H7a), 1.88 – 1.78 (m, 2 H, H7b), 1.68 (br s, 1 H, OH).
^{13}C NMR (150 MHz, CDCl$_3$): δ 145.7 (C4), 132.6 (C1), 128.4 (C3), 126.7 (C2), 83.2 (C5), 42.1 (C6), 24.0 (C7).
FTIR (ν_{max}, cm^{-1}): 3365 (br w, OH), 2964 (m), 2874 (w), 1597 (w), 1492 (m), 1450 (w), 1399 (w), 1323 (w), 1295 (w), 1178 (w), 1093 (s), 1039 (w), 1012 (s), 1003 (s), 960 (w), 904 (w), 882 (w), 824 (s).
HRMS (ESI): calculated for $C_{11}H_{13}OClNa$ $[M+Na]^+$ 219.0547, found 219.0554.
R$_f$ = 0.21 (10% EtOAc/hexane).

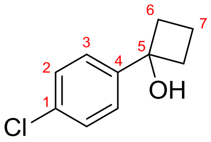

4k

1-(4-chlorophenyl)cyclobutan-1-ol (4k): Following the general procedure for oxidative coupling using 7-methoxy-7-methyl-8-oxa-5,6-diazaspiro[3.4]oct-5-ene (0.156 g, 1.0 mmol) and 4-chlorophenylboronic acid (78.2 mg, 0.5 mmol), purified by silica gel column chromatography (eluent: 20% EtOAc/hexane) provided the title compound as a colourless oil (60.9 mg, 0.333 mmol, 67%). Data are consistent with a reported example.[20]

^{1}H NMR (600 MHz, $CDCl_3$): δ 7.43 (d, J = 8.6 Hz, 2 H, H2), 7.33 (d, J = 8.6 Hz, 2 H, H3), 2.55 – 2.48 (m, 2 H, H6a), 2.39 – 2.32 (m, 2 H, H6b), 2.14 (br s, 1 H, OH), 2.06 – 1.98 (m, 1 H, H7a), 1.73 – 1.64 (m, 1 H, H7b).
^{13}C NMR (150 MHz, $CDCl_3$): δ 144.9 (C4), 133.1 (C1), 128.6 (C3), 126.6 (C2), 76.7 (C5), 37.1 (C6), 13.0 (C7).
FTIR (ν_{max}, cm^{-1}): 3341 (br w, OH), 2988 (w), 2940 (w), 1599 (w), 1493 (m), 1423 (w), 1399 (w), 1282 (w), 1244 (m), 1181 (w), 1133 (m), 1093 (s), 1067 (w), 1032 (w), 1012 (s), 957 (w), 890 (w), 828 (s).
HRMS (ESI): calculated for $C_{10}H_{11}OClNa$ $[M+Na]^+$ 205.0391, found 205.0400.
R$_f$ = 0.29 (20% EtOAc/hexane).

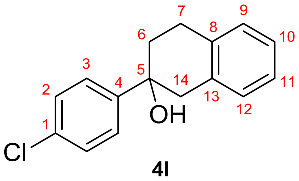

4l

2-(4-chlorophenyl)-1,2,3,4-tetrahydronaphthalen-2-ol (4l): Following the general procedure for oxidative coupling using 5'-methoxy-5'-methyl-3,4-dihydro-1*H*,5'*H*-spiro[naphthalene-2,2'-[1,3,4]oxadiazole] (0.232 g, 1.0 mmol) and 4-chlorophenylboronic acid (78.2 mg, 0.5 mmol), purified by silica gel column chromatography (eluent: 30% Et_2O/hexane) provided the title compound as a colourless gum (92.3 mg, 0.357 mmol, 71%).

^{1}H NMR (600 MHz, $CDCl_3$): δ 7.46 (d, J = 8.6 Hz, 2 H, H2), 7.33 (d, J = 8.6 Hz, 2 H, H3), 7.21 – 7.08 (m, 4 H, H9, H10, H11 and H12), 3.29 (d, J = 16.9 Hz, 1 H, H14a), 3.15 – 3.05 (m, 1 H, H7a), 3.01 (d, J = 16.9 Hz, 1 H, H14b), 2.79 (dt, J = 17.1, 5.3 Hz, 1 H, H7b), 2.27 – 2.18 (m, 1 H, H6a), 2.11 – 2.04 (m, 1 H, H6b), 2.01 (br s, 1 H, OH).
^{13}C NMR (150 MHz, $CDCl_3$): δ 146.3 (C4), 135.3 (C8/C13), 134.2 (C8/C13), 133.0 (C1), 129.5 (C9/C10/C11/C12), 129.0 (C9/C10/C11/C12), 128.5 (C3), 126.5 (C2), 126.4 (C9/C10/C11/C12), 126.2 (C9/C10/C11/C12), 72.4 (C5), 43.8 (C14), 35.5 (C6), 26.4 (C7).
FTIR (ν_{max}, cm^{-1}): 3367 (br w, OH), 3061 (w), 3019 (w), 2921 (w), 2845 (w), 1596 (w), 1583 (w), 1494 (m), 1453 (w), 1433 (w), 1398 (w), 1346 (w), 1317 (w), 1298 (w), 1243 (w), 1175 (w), 1092 (m), 1077 (m), 1037 (w), 1013 (m), 964 (m), 908 (m), 876 (w), 853 (w), 816 (s), 762 (m).
HRMS (ESI): calculated for $C_{16}H_{15}OClNa$ $[M+Na]^+$ 281.0704, found 281.0713.
R$_f$ = 0.31 (30% Et_2O/hexane).

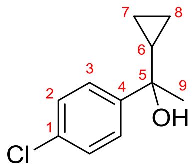

4n

1-(4-chlorophenyl)-1-cyclopropylethan-1-ol (4n): Following the general procedure for oxidative coupling using 2-cyclopropyl-5-methoxy-2,5-dimethyl-2,5-dihydro-1,3,4-oxadiazole (0.170 g, 1.0 mmol) and 4-chlorophenylboronic acid (78.2 mg, 0.5 mmol), purified by silica gel column chromatography (eluent: 10% EtOAc/hexane) provided the title compound as a colourless oil (82.8 mg, 0.421 mmol, 84%). Data are consistent with a reported example.[21]

^{1}H NMR (600 MHz, CDCl$_3$): δ 7.46 (d, J = 8.6 Hz, 2 H, H2), 7.30 (d, J = 8.6 Hz, 2 H, H3), 1.63 (br s, 1 H, OH), 1.46 (s, 3 H, H9), 1.22 (tt, J = 8.3, 5.6 Hz, 1 H, H6), 0.58 – 0.49 (m, 1 H, H7a), 0.49 – 0.35 (m, 3 H, H7b and H8).
^{13}C NMR (150 MHz, CDCl$_3$): δ 146.7 (C4), 132.6 (C1), 128.2 (C3), 126.8 (C2), 73.1 (C5), 28.6 (C9), 23.0 (C6), 2.2 (C7/C8), 1.2 (C7/C8).
FTIR (ν_{max}, cm^{-1}): 3419 (br w, OH), 3085 (w), 3009 (w), 2978 (w), 1597 (w), 1489 (m), 1455 (w), 1400 (w), 1368 (w), 1227 (w), 1175 (w), 1091 (s), 1043 (m), 1012 (s), 952 (w), 925 (m), 897 (m), 828 (s).
HRMS (ESI): calculated for $C_{11}H_{13}OClNa$ [M+Na]$^+$ 219.0547, found 219.0546.
R_f = 0.23 (10% EtOAc/hexane).

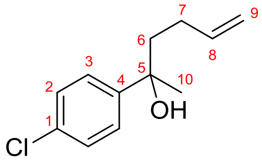

4o

2-(4-chlorophenyl)hex-5-en-2-ol (4o): Following the general procedure for oxidative coupling using 2-(but-3-en-1-yl)-5-methoxy-2,5-dimethyl-2,5-dihydro-1,3,4-oxadiazole (0.184 g, 1.0 mmol) and 4-chlorophenylboronic acid (78.2 mg, 0.5 mmol), purified by silica gel column chromatography (eluent: 10% EtOAc/hexane) provided the title compound as a colourless oil (97.5 mg, 0.463 mmol, 93%).

^{1}H NMR (600 MHz, CDCl$_3$): δ 7.36 (d, J = 8.6 Hz, 2 H, H2), 7.30 (d, J = 8.6 Hz, 2 H, H3), 5.84 – 5.72 (m, 1 H, H8), 5.00 – 4.94 (m, 1 H, H9$_{trans}$), 4.93 (d, J = 10.3 Hz, 1 H, H9$_{cis}$), 2.10 – 1.97 (m, 1 H, H7a), 1.95 – 1.84 (m, 4 H, H6, H7b and OH), 1.54 (s, 3 H, H10).
^{13}C NMR (150 MHz, CDCl$_3$): δ 146.3 (C4), 138.6 (C8), 132.5 (C1), 128.4 (C3), 126.5 (C2), 114.9 (C9), 74.6 (C5), 43.1 (C6), 30.5 (C10), 28.6 (C7).
FTIR (ν_{max}, cm^{-1}): 3406 (br w, OH), 2976 (w), 2930 (w), 1641 (w), 1598 (w), 1490 (m), 1452 (w), 1397 (w), 1373 (w), 1304 (w), 1219 (w), 1093 (s), 1013 (s), 996 (w), 934 (w), 910 (m), 881 (w), 829 (s).
HRMS (ESI): calculated for $C_{12}H_{15}OClNa$ [M+Na]$^+$ 233.0704, found 233.0699.
R_f = 0.26 (10% EtOAc/hexane).

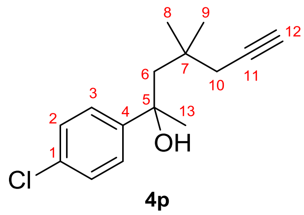

4p

2-(4-chlorophenyl)-4,4-dimethylhept-6-yn-2-ol (4p): Following the general procedure for oxidative coupling using 2-(2,2-dimethylpent-4-yn-1-yl)-5-methoxy-2,5-dimethyl-2,5-dihydro-1,3,4-oxadiazole (0.224 g, 1.0 mmol) and 4-chlorophenylboronic acid (78.2 mg, 0.5 mmol), purified by silica gel column chromatography (eluent: 10% EtOAc/hexane) provided the title compound as a colourless oil (104.4 mg, 0.416 mmol, 83%).

^{1}H NMR (600 MHz, $CDCl_3$): δ 7.40 (d, *J* = 8.7 Hz, 2 H, H2), 7.28 (d, *J* = 8.7 Hz, 2 H, H3), 2.20 (dd, *J* = 16.5, 2.7 Hz, 1 H, H10a), 2.04 (t, *J* = 2.7 Hz, 1 H, H12), 1.99 (s, 2 H, H6), 1.943 (br s, 1 H, OH), 1.940 (dd, *J* = 16.5, 2.7 Hz, 1 H, H10b), 1.54 (s, 3 H, H13), 0.87 (s, 3 H, H8/H9), 0.76 (s, 3 H, H8/H9).
^{13}C NMR (150 MHz, $CDCl_3$): δ 146.8 (C4), 132.2 (C1), 128.2 (C3), 126.6 (C2), 83.3 (C11), 75.2 (C5), 70.9 (C12), 52.6 (C6), 34.9 (C13), 34.5 (C7), 32.8 (C10), 29.4 (C8/C9), 28.7 (C8/C9).
FTIR (ν_{max}, cm^{-1}): 3429 (br w, OH), 3304 (w, alkyne CH), 2961 (w), 2927 (w), 1598 (w), 1491 (m), 1471 (w), 1425 (w), 1398 (w), 1367 (w), 1265 (w), 1174 (w), 1093 (m), 1075 (m), 1013 (s), 946 (w), 923 (w), 829 (s).
HRMS (ESI): calculated for $C_{15}H_{19}OClNa$ $[M+Na]^+$ 273.1017, found 273.1020.
R_f = 0.28 (10% EtOAc/hexane).

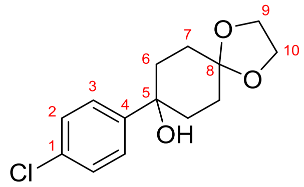

4q

8-(4-chlorophenyl)-1,4-dioxaspiro[4.5]decan-8-ol (4q): Following the general procedure for oxidative coupling using 3-methoxy-3-methyl-4,9,12-trioxa-1,2-diazadispiro[4.2.4^8.2^5]tetradec-1-ene (0.242 g, 1.0 mmol) and 4-chlorophenylboronic acid (78.2 mg, 0.5 mmol), purified by silica gel column chromatography (eluent: 10% EtOAc/CH_2Cl_2) provided the title compound as a white crystalline solid (111.1 mg, 0.413 mmol, 83%), m.p. 158-160 °C (lit m.p.[22] 147-149 °C). Data are consistent with a reported example.[23]

^{1}H NMR (600 MHz, $CDCl_3$): δ 7.45 (d, *J* = 8.6 Hz, 2 H, H2), 7.30 (d, *J* = 8.6 Hz, 2 H, H3), 4.02 – 3.93 (m, 4 H, H9/H10), 2.17 – 2.04 (m, 4 H, H6a and H7a), 1.81 – 1.75 (m, 2 H, H6b), 1.72 – 1.66 (m, 2 H, H7b), 1.64 (br s, 1 H, OH).
^{13}C NMR (150 MHz, $CDCl_3$): δ 147.2 (C4), 132.8 (C1), 128.5 (C3), 126.2 (C2), 108.4 (C8), 72.4 (C5), 64.5 (C9/C10), 64.4 (C9/C10), 36.7 (C6), 30.8 (C7).
FTIR (ν_{max}, cm^{-1}): 3459 (br w, OH), 2931 (m), 2884 (w), 1493 (m), 1435 (w), 1398 (w), 1369 (w), 1251 (w), 1216 (w), 1181 (w), 1145 (w), 1096 (s), 1034 (m), 1013 (m), 987 (m), 943 (m), 889 (w), 826 (m), 772 (w).
HRMS (ESI): calculated for $C_{14}H_{17}O_3ClNa$ $[M+Na]^+$ 291.0758, found 291.0768.
R_f = 0.23 (10% EtOAc/CH_2Cl_2).

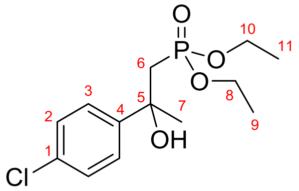

4r

Diethyl (2-(4-chlorophenyl)-2-hydroxypropyl)phosphonate (4r): Following the general procedure for oxidative coupling using diethyl ((5-methoxy-2,5-dimethyl-2,5-dihydro-1,3,4-oxadiazol-2-yl)methyl)phosphonate (0.280 g, 1.0 mmol) and 4-chlorophenylboronic acid (78.2 mg, 0.5 mmol), purified by silica gel column chromatography (eluent: 35% EtOAc/60% hexane/5% MeOH) provided the title compound as a colourless oil (92.6 mg, 0.302 mmol, 60%).

^{1}H NMR (600 MHz, $CDCl_3$): δ 7.40 (d, *J* = 8.6 Hz, 2 H, H2), 7.29 (d, *J* = 8.6 Hz, 2 H, H3), 5.03 (s, 1 H, OH), 4.13 – 3.96 (m, 2 H, H8/H10), 3.79 – 3.69 (m, 1 H, H8/H10), 3.55 – 3.46 (m, 1 H, H8/H10), 2.40 (dd, *J* = 17.5, 15.5 Hz, 1 H, H6a), 2.29 (t, *J* = 16.5, 15.5 Hz, 1 H, H6b), 1.58 (d, *J* = 2.1 Hz, 3 H, H7), 1.30 (t, *J* = 7.1 Hz, 3 H, H9/H11), 1.02 (t, *J* = 7.1 Hz, 3 H, H9/H11).
^{13}C NMR (150 MHz, $CDCl_3$): δ 145.9 (d, *J* = 7.3 Hz, C4), 132.7 (C1), 128.2 (C3), 126.5 (C2), 71.8 (d, *J* = 4.9 Hz, C5), 61.9 (d, *J* = 6.4 Hz, C8/C10), 61.8 (d, *J* = 6.5 Hz, C8/C10), 39.6 (d, *J* = 135.6 Hz, C6), 32.5 (d, *J* = 14.1 Hz, C7), 16.4 (d, *J* = 6.2 Hz, C9/C11), 16.2 (d, *J* = 6.1 Hz, C9/C11).
^{31}P NMR (245 MHz, $CDCl_3$): δ 28.5 (s, 1 P, C6-P).
FTIR (ν_{max}, cm^{-1}): 3386 (br w, OH), 2981 (w), 2932 (w), 2908 (w), 1491 (w), 1443 (w), 1394 (w), 1369 (w), 1218 (m), 1164 (w), 1093 (m), 1049 (s), 1025 (s), 964 (m), 912 (w), 833 (m), 782 (w).
HRMS (ESI): calculated for $C_{13}H_{20}O_4PClNa$ $[M+Na]^+$ 329.0680, found 329.0678.
R_f = 0.36 (35% EtOAc/60% hexane/5% MeOH).

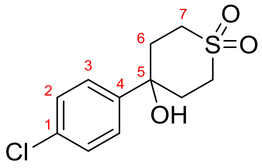

4s

4-(4-chlorophenyl)-4-hydroxytetrahydro-2*H*-thiopyran 1,1-dioxide (4s): Following the general procedure for oxidative coupling using 3-methoxy-3-methyl-4-oxa-8-thia-1,2-diazaspiro[4.5]dec-1-ene 8,8-dioxide (0.234 g, 1.0 mmol) and 4-chlorophenylboronic acid (78.2 mg, 0.5 mmol), purified by silica gel column chromatography (eluent: 20% → 40% EtOAc/CH_2Cl_2) provided the title compound as a white crystalline solid (90.8 mg, 0.348 mmol, 70%), m.p. 196-198 °C.

^{1}H NMR (600 MHz, $CDCl_3$): δ 7.43 (d, *J* = 8.7 Hz, 2 H, H2), 7.37 (d, *J* = 8.7 Hz, 2 H, H3), 3.58 (td, *J* = 13.8, 3.5 Hz, 2 H, H7a), 3.00 – 2.91 (m, 2 H, H7b), 2.68 (td, *J* = 14.5, 2.6 Hz, 2 H, H6a), 2.18 – 2.10 (m, 2 H, H6b), 1.78 (br s, 1 H, OH).
^{13}C NMR (150 MHz, $CDCl_3$): δ 144.6 (C4), 134.2 (C1), 129.1 (C3), 125.8 (C2), 70.6 (C5), 47.3 (C7), 37.1 (C6).
FTIR (ν_{max}, cm^{-1}): 3404 (br m, OH), 1595 (w), 1485 (m), 1433 (w), 1393 (m), 1363 (w), 1334 (m), 1284 (s), 1272 (s), 1243 (w), 1224 (w), 1193 (w), 1172 (m), 1119 (s), 1093 (m), 1068 (s), 1023 (m), 1013 (m), 984 (w), 968 (m), 931 (s), 878 (w), 853 (w), 823 (s).

HRMS (ESI): calculated for $C_{11}H_{13}O_3SClNa$ $[M+Na]^+$ 283.0166, found 283.0170.
R_f = 0.34 (20% EtOAc/CH_2Cl_2).

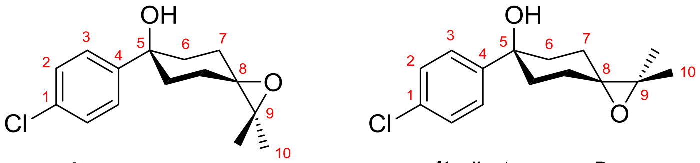

4t, *diastereomer A* **4t**, *diastereomer B*

6-(4-chlorophenyl)-2,2-dimethyl-1-oxaspiro[2.5]octan-6-ol (4t): Following the general procedure for oxidative coupling using 9-methoxy-2,2,9-trimethyl-1,10-dioxa-7,8-diazadispiro[2.2.4^6.2^3]dodec-7-ene (0.240 g, 1.0 mmol) and 4-chlorophenylboronic acid (78.2 mg, 0.5 mmol), purified by silica gel column chromatography (eluent: 30% EtOAc/hexane) provided the title compound as separable diastereomers (1:1) as a white crystalline solids (A: 55.5 mg, 0.208 mmol; B: 60.3 mg, 0.226 mmol; combined yield 87%), m.p. 168-171 °C for diastereomer A and m.p. 156-159 °C for diastereomer B.

Diastereomer A:
^{1}H NMR (600 MHz, $CDCl_3$): δ 7.45 (d, J = 8.6 Hz, 2 H, H2), 7.30 (d, J = 8.6 Hz, 2 H, H3), 2.20 (td, J = 13.5, 3.6 Hz, 2 H, H7a), 2.12 (td, J = 13.5, 3.6 Hz, 2 H, H6a), 1.84 – 1.79 (m, 2 H, H6b), 1.78 (br s, 1 H, OH), 1.55 – 1.49 (m, 2 H, H7b), 1.35 (s, 6 H, H10).
^{13}C NMR (150 MHz, $CDCl_3$): δ 147.5 (C4), 132.8 (C1), 128.5 (C3), 126.2 (C2), 72.5 (C5), 65.1 (C8), 63.3 (C9), 36.4 (C6), 25.8 (C7), 20.7 (C10).
FTIR (ν_{max}, cm^{-1}): 3390 (br m, OH), 2928 (m), 1735 (w), 1489 (w), 1432 (w), 1379 (m), 1313 (w), 1245 (m), 1195 (m), 1133 (w), 1095 (s), 1036 (m), 1011 (m), 988 (m), 961 (m), 903 (w), 889 (w), 849 (s), 833 (m), 817 (s), 756 (w).
HRMS (ESI): calculated for $C_{15}H_{19}O_2ClNa$ $[M+Na]^+$ 289.0966, found 289.0960.
R_f = 0.38 (30% EtOAc/hexane).

Diastereomer B:
^{1}H NMR (600 MHz, $CDCl_3$): δ 7.44 (d, J = 8.6 Hz, 2 H, H2), 7.31 (d, J = 8.6 Hz, 2 H, H3), 2.26 – 2.15 (m, 2 H, H7a), 1.98 – 1.88 (m, 5 H, H6 and OH), 1.62 – 1.56 (m, 2 H, H7b), 1.39 (s, 6 H, H10).
^{13}C NMR (150 MHz, $CDCl_3$): δ 146.5 (C4), 133.0 (C1), 128.5 (C3), 126.4 (C2), 72.2 (C5), 66.1 (C8), 62.5 (C9), 38.3 (C6), 26.9 (C7), 20.9 (C10).
FTIR (ν_{max}, cm^{-1}): 3426 (br m, OH), 2938 (m), 1710 (w), 1491 (s), 1474 (m), 1377 (s), 1241 (m), 1174 (w), 1118 (m), 1095 (s), 1064 (s), 1033 (m), 1013 (s), 993 (m), 961 (s), 900 (m), 853 (m), 827 (s).
HRMS (ESI): calculated for $C_{15}H_{19}O_2ClNa$ $[M+Na]^+$ 289.0966, found 289.0967.
R_f = 0.21 (30% EtOAc/hexane).

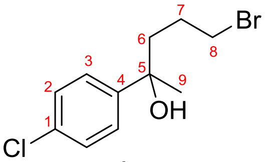

4u

5-bromo-2-(4-chlorophenyl)pentan-2-ol (4u): Following the general procedure for oxidative coupling using 2-(3-bromopropyl)-5-methoxy-2,5-dimethyl-2,5-dihydro-1,3,4-oxadiazole (0.251 g, 1.0 mmol) and 4-chlorophenylboronic acid (78.2 mg, 0.5 mmol), purified by silica gel column chromatography (eluent: 20% EtOAc/hexane) provided the title compound as a yellow oil (99.0 mg, 0.357 mmol, 87%).

^{1}H NMR (600 MHz, $CDCl_3$): δ 7.35 (d, *J* = 8.7 Hz, 2 H, H2), 7.30 (d, *J* = 8.7 Hz, 2 H, H3), 3.39 – 3.29 (m, 2 H, H8), 1.97 – 1.82 (m, 3 H, H6 and H7a), 1.76 (br s, 1 H, OH), 1.72 – 1.63 (m, 1 H, H7b), 1.56 (s, 3 H, H9).
^{13}C NMR (150 MHz, $CDCl_3$): δ 145.9 (C4), 132.7 (C1), 128.5 (C3), 126.4 (C2), 74.2 (C5), 42.7 (C6), 34.3 (C8), 30.8 (C9), 27.6 (C7).
FTIR (ν_{max}, cm^{-1}): 3422 (br w, OH), 2967 (w), 1598 (w), 1490 (m), 1453 (w), 1397 (w), 1373 (w), 1293 (w), 1254 (m), 1202 (w), 1163 (w), 1093 (s), 1013 (s), 956 (w), 889 (w), 831 (s), 767 (w).
HRMS (ESI): calculated for $C_{11}H_{14}OBrClNa$ $[M+Na]^+$ 298.9809, found 298.9823.
$\boldsymbol{R_f}$ = 0.34 (20% EtOAc/hexane).

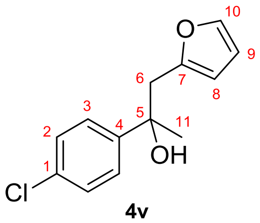

4v

2-(4-chlorophenyl)-1-(furan-2-yl)propan-2-ol (4v): Following the general procedure for oxidative coupling using 2-(furan-2-ylmethyl)-5-methoxy-2,5-dimethyl-2,5-dihydro-1,3,4-oxadiazole (0.210 g, 1.0 mmol) and 4-chlorophenylboronic acid (78.2 mg, 0.5 mmol), purified by silica gel column chromatography (eluent: 30% Et_2O/CH_2Cl_2) provided the title compound as an orange gum (90.2 mg, 0.381 mmol, 76%).

^{1}H NMR (600 MHz, $CDCl_3$): δ 7.37 (d, *J* = 8.6 Hz, 2 H, H2), 7.31 (d, *J* = 1.9 Hz, 1 H, H10), 7.29 (d, *J* = 8.6 Hz, 2 H, H3), 6.26 (dd, *J* = 3.0, 1.9 Hz, 1 H, H9), 5.97 (d, *J* = 3.0 Hz, 1 H, H8), 3.13 (d, *J* = 15.0 Hz, 1 H, H6a), 3.08 (d, *J* = 15.0 Hz, 1 H, H6b), 2.45 (br s, 1 H, OH), 1.54 (s, 3 H, H11).
^{13}C NMR (150 MHz, $CDCl_3$): δ 151.6 (C7), 145.8 (C4), 142.0 (C10), 132.7 (C1), 128.3 (C3), 126.4 (C2), 110.5 (C9), 108.6 (C8), 74.0 (C5), 42.7 (C6), 29.7 (C11).
FTIR (ν_{max}, cm^{-1}): 3429 (br w, OH), 2974 (w), 2910 (w), 1596 (w), 1490 (m), 1455 (w), 1400 (w), 1376 (w), 1266 (w), 1179 (m), 1147 (m), 1090 (s), 1012 (s), 951 (m), 935 (m), 885 (w), 856 (m), 828 (s).
HRMS (ESI): calculated for $C_{13}H_{13}O_2ClNa$ $[M+Na]^+$ 259.0496, found 259.0485.
$\boldsymbol{R_f}$ = 0.21 (10% EtOAc/hexane).

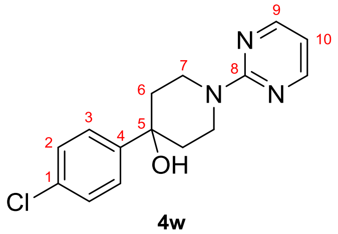

4w

4-(4-chlorophenyl)-1-(pyrimidin-2-yl)piperidin-4-ol (4w): Following the general procedure for oxidative coupling using 3-methoxy-3-methyl-8-(pyrimidin-2-yl)-4-oxa-1,2,8-triazaspiro[4.5]dec-1-ene (0.263 g, 1.0 mmol) and 4-chlorophenylboronic acid (78.2 mg, 0.5 mmol), purified by silica gel column chromatography (eluent: 30% Et_2O/CH_2Cl_2) provided the title compound as a colourless gum (83.3 mg, 0.287 mmol, 57%).

^{1}H NMR (600 MHz, $CDCl_3$): δ 8.30 (d, *J* = 4.8 Hz, 2 H, H9), 7.41 (d, *J* = 8.6 Hz, 2 H, H2), 7.31 (d, *J* = 8.6 Hz, 2 H, H3), 6.47 (t, *J* = 4.8 Hz, 1 H, H10), 4.72 – 4.64 (m, 2 H, H7a), 3.39

(td, J = 13.1, 2.6 Hz, 2 H, H7b), 2.07 (br s, 1 H, OH), 2.02 (td, J = 13.4, 4.8 Hz, 2 H, H6a), 1.84 – 1.76 (m, 2 H, H6b).
^{13}C NMR (150 MHz, $CDCl_3$): δ 161.6 (C8), 157.9 (C9), 146.8 (C4), 133.1 (C1), 128.6 (C3), 126.2 (C2), 109.7 (C10), 71.8 (C5), 40.0 (C7), 38.1 (C6).
FTIR (ν_{max}, cm^{-1}): 3361 (br w, OH), 3027 (w), 2998 (w), 2950 (w), 2920 (w), 2868 (w), 1584 (s), 1546 (s), 1493 (s), 1456 (s), 1392 (m), 1362 (s), 1306 (m), 1272 (m), 1254 (m), 1238 (w), 1215 (m), 1177 (w), 1132 (w), 1091 (m), 1027 (m), 1012 (m), 980 (s), 923 (m), 825 (s), 794 (s).
HRMS (ESI): calculated for $C_{15}H_{17}N_3OCl$ $[M+H]^+$ 290.1055, found 290.1053.
***R*$_f$** = 0.22 (30% Et_2O/CH_2Cl_2).

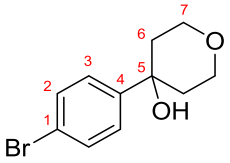

4x

4-(4-bromophenyl)tetrahydro-2*H*-pyran-4-ol (4x): Following the general procedure for oxidative coupling using 3-methoxy-3-methyl-4,8-dioxa-1,2-diazaspiro[4.5]dec-1-ene (0.186 g, 1.0 mmol) and 4-bromophenylboronic acid (100.4 mg, 0.5 mmol), purified by silica gel column chromatography (eluent: 40% EtOAc/hexane) provided the title compound as a white crystalline solid (91.0 mg, 0.354 mmol, 71%), m.p. 114-116 °C. Data are consistent with a reported example.[24]

^{1}H NMR (600 MHz, $CDCl_3$): δ 7.48 (d, J = 8.7 Hz, 2 H, H2), 7.34 (d, J = 8.7 Hz, 2 H, H3), 3.88 (td, J = 11.9, 2.0 Hz, 2 H, H7a), 3.85 – 3.79 (m, 2 H, H7b), 2.13 (br s, 1 H, OH), 2.12 – 2.04 (m, 2 H, H6a), 1.65 – 1.58 (m, 2 H, H6b).
^{13}C NMR (150 MHz, $CDCl_3$): δ 147.3 (C4), 131.6 (C2), 126.5 (C3), 121.2 (C1), 70.5 (C5), 63.8 (C7), 38.7 (C6).
FTIR (ν_{max}, cm^{-1}): 3399 (br m, OH), 2954 (m), 2869 (m), 1589 (w), 1492 (m), 1388 (m), 1302 (w), 1237 (m), 1221 (w), 1126 (s), 1103 (s), 1073 (m), 1033 (s), 1009 (s), 965 (w), 915 (w), 821 (s), 799 (w).
HRMS (ESI): calculated for $C_{11}H_{13}O_2BrNa$ $[M+Na]^+$ 278.9991, found 278.9994.
***R*$_f$** = 0.22 (40% EtOAc/hexane).

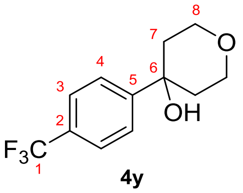

4y

4-(4-(trifluoromethyl)phenyl)tetrahydro-2*H*-pyran-4-ol (4y): Following the general procedure for oxidative coupling using 3-methoxy-3-methyl-4,8-dioxa-1,2-diazaspiro[4.5]dec-1-ene (0.186 g, 1.0 mmol) and 4-(trifluoromethyl)phenylboronic acid (95.0 mg, 0.5 mmol), purified by silica gel column chromatography (eluent: 40% EtOAc/hexane) provided the title compound as a white crystalline solid (85.9 mg, 0.349 mmol, 70%), m.p. 82-84 °C.

^{1}H NMR (600 MHz, $CDCl_3$): δ 7.65 – 7.58 (m, 4 H, H3 and H4), 3.95 – 3.89 (m, 2 H, H8a), 3.89 – 3.83 (m, 2 H, H8b), 2.21 – 2.10 (m, 2 H, H7a), 2.03 (br s, 1 H, OH), 1.69 – 1.60 (m, 2 H, H7b).

^{13}C NMR (150 MHz, CDCl$_3$): δ 152.1 (q, *J* = 0.9 Hz, C5), 129.5 (q, *J* = 32.4 Hz, C2), 125.5 (q, *J* = 3.7 Hz, C3), 125.1 (C4), 124.2 (q, *J* = 272.0 Hz, C1), 70.8 (C6), 63.8 (C8), 38.7 (C7).
^{19}F NMR (376 MHz, CDCl$_3$): δ -62.5 (s, 3 F, F1).
FTIR (ν_{max}, cm^{-1}): 3402 (br w, OH), 2958 (w), 2873 (w), 1618 (w), 1469 (w), 1409 (w), 1389 (w), 1324 (s), 1303 (m), 1239 (w), 1224 (w), 1164 (m), 1106 (s), 1071 (s), 1034 (m), 1016 (m), 966 (w), 918 (w), 835 (s), 799 (w), 777 (w).
HRMS (ESI): calculated for $C_{12}H_{13}F_3O_2Na$ $[M+Na]^+$ 269.0760, found 269.0752.
R$_f$ = 0.28 (40% EtOAc/hexane).

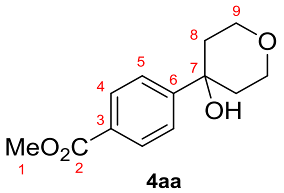

4aa

Methyl 4-(4-hydroxytetrahydro-2*H*-pyran-4-yl)benzoate (4aa): Following the general procedure for oxidative coupling using 3-methoxy-3-methyl-4,8-dioxa-1,2-diazaspiro[4.5]dec-1-ene (0.186 g, 1.0 mmol) and 4-methoxycarbonylphenylboronic acid (90.0 mg, 0.5 mmol), purified by silica gel column chromatography (eluent: 50% EtOAc/hexane) provided the title compound as a white crystalline solid (36.4 mg, 0.154 mmol, 31%), m.p. 97-98 °C.

^{1}H NMR (600 MHz, CDCl$_3$): δ 7.99 (d, *J* = 8.6 Hz, 2 H, H4), 7.54 (d, *J* = 8.6 Hz, 2 H, H5), 3.94 – 3.89 (m, 2 H, H9a), 3.88 (s, 3 H, H1), 3.85 (dd, *J* = 11.3, 5.0 Hz, 2 H, H9b), 2.34 (br s, 1 H, OH), 2.14 (td, *J* = 13.2, 5.0 Hz, 2 H, H8a), 1.64 (d, *J* = 13.2 Hz, 2 H, H8b).
^{13}C NMR (150 MHz, CDCl$_3$): δ 167.0 (C2), 153.3 (C6), 129.9 (C4), 128.9 (C3), 124.7 (C5), 70.8 (C7), 63.8 (C9), 52.3 (C1), 38.6 (C8).
FTIR (ν_{max}, cm^{-1}): 3420 (br w, OH), 2954 (w), 2870 (w), 1722 (s, C=O), 1610 (w), 1575 (w), 1436 (m), 1407 (w), 1387 (w), 1281 (s), 1239 (w), 1224 (w), 1192 (w), 1104 (s), 1035 (w), 1018 (m), 967 (w), 917 (w), 856 (w), 840 (m), 773 (m).
HRMS (ESI): calculated for $C_{13}H_{16}O_4Na$ $[M+Na]^+$ 259.0941, found 259.0945.
R$_f$ = 0.30 (50% EtOAc/hexane).

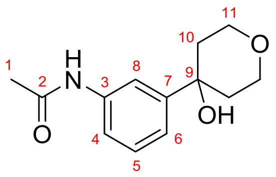

4ab

***N*-(3-(4-hydroxytetrahydro-2*H*-pyran-4-yl)phenyl)acetamide (4ab):** Following the general procedure for oxidative coupling using 3-methoxy-3-methyl-4,8-dioxa-1,2-diazaspiro[4.5]dec-1-ene (0.186 g, 1.0 mmol) and 3-acetamidophenylboronic acid (89.5 mg, 0.5 mmol), purified by silica gel column chromatography (eluent: 10% MeOH/CH_2Cl_2) provided the title compound as a white flaky solid (62.3 mg, 0.265 mmol, 53%), m.p. 128-130 °C.

^{1}H NMR (600 MHz, MeOD-*d*$_4$): δ 7.68 (t, *J* = 1.8 Hz, 1 H, H8), 7.45 (ddd, *J* = 7.9, 1.8, 1.1 Hz, 1 H, H4), 7.29 (t, *J* = 7.8 Hz, 1 H, H5), 7.27 – 7.23 (m, 1 H, H6), 3.93 (td, *J* = 11.5, 2.0 Hz, 2 H, H11a), 3.81 (dd, *J* = 11.5, 4.7 Hz, 2 H, H11b), 2.16 – 2.06 (m, 2 H, H10a), 2.12 (s, 3 H, H1), 1.68 – 1.63 (m, 2 H, H10b).

^{13}C NMR (150 MHz, MeOD-*d*$_4$): δ 171.7 (C2), 150.9 (C7), 139.8 (C3), 129.7 (C5), 121.6 (C6), 119.8 (C4), 117.9 (C8), 71.1 (C9), 65.0 (C11), 39.7 (C10), 23.8 (C1).
FTIR (ν_{max}, cm^{-1}): 3388 (br m, OH), 3299 (m, NH), 2957 (m), 2872 (w), 1665 (s, C=O), 1610 (s), 1592 (m), 1553 (s), 1489 (s), 1428 (s), 1372 (m), 1302 (s), 1263 (m), 1239 (m), 1185 (w), 1125 (m), 1095 (s), 1041 (m), 1017 (m), 984 (w), 963 (w), 928 (w), 884 (w), 855 (w), 833 (m), 791 (m).
HRMS (ESI): calculated for $C_{13}H_{17}NO_3Na$ [M+Na]$^+$ 258.1101, found 258.1110.
R_f = 0.33 (10% MeOH/CH_2Cl_2).

5. Synthesis of cyclobutylated boronic ester 5 and further derivatisation

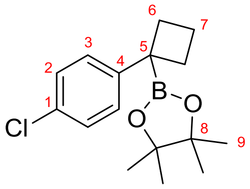

5

2-(1-(4-chlorophenyl)cyclobutyl)-4,4,5,5-tetramethyl-1,3,2-dioxaborolane (5): A solution of 7-methoxy-7-methyl-8-oxa-5,6-diazaspiro[3.4]oct-5-ene (0.781 g, 5.0 mmol, 2.0 equiv.), 4-chlorophenylboronic acid (0.391 g, 2.5 mmol, 1.0 equiv.) and DIPEA (0.85 mL, 5.0 mmol, 2.0 equiv.) in CH_2Cl_2 (50 mL) was pumped at a flow rate of 0.125 mL min^{-1} through a Vapourtec UV-150 photochemical reactor (10 mL reactor volume, FEP tubing), irradiated by a 310 nm UV lamp (9 W output power), held at 10 °C and the reactor output was monitored using a FlowIR® device (SiComp head, 2100-2000 cm^{-1} and 1750-1700 cm^{-1}). When the FlowIR® showed that the reaction mixture was starting to exit the output stream (by monitoring the MeOAc C=O stretch at 1750-1700 cm^{-1}), the output was directed into a flask containing pinacol (1.48 g, 12.5 mmol, 5.0 equiv.). After 400 min once the reaction mixture has fully been taken up by the pump, the input was swapped to CH_2Cl_2 solvent and the output collected until the FlowIR® showed that the reaction mixture had finished exiting the reactor, then mixture stirred further at r.t. for 16 h. The mixture was then evaporated under reduced pressure and purified by silica gel column chromatography (eluent: 1% EtOAc/hexane) to provide the title compound as a white crystalline solid (0.549 g, 1.88 mmol, 74%), m.p. 89-92 °C.

^{1}H NMR (600 MHz, $CDCl_3$): δ 7.21 (d, J = 8.4 Hz, 2 H, H2), 7.03 (d, J = 8.4 Hz, 2 H, H3), 2.57 – 2.48 (m, 2 H, H6a), 2.27 – 2.18 (m, 2 H, H6b), 2.09 – 1.98 (m, 1 H, H7a), 1.88 – 1.79 (m, 1 H, H7b), 1.18 (s, 12 H, H9).
^{13}C NMR (150 MHz, $CDCl_3$): δ 147.7 (C4), 130.2 (C1), 128.2 (C2), 127.1 (C3), 83.7 (C8), 32.1 (C7), 24.6 (C6), 18.9 (C9). (C5 broadened by quadrupolar relaxation with boron).
FTIR (ν_{max}, cm^{-1}): 2978 (m), 2868 (w), 1597 (w), 1489 (m), 1470 (w), 1446 (w), 1380 (m), 1372 (m), 1351 (s), 1313 (s), 1273 (w), 1215 (s), 1167 (w), 1145 (s), 1119 (s), 1093 (s), 1013 (m), 964 (m), 924 (w), 844 (s), 825 (m).
HRMS (ESI): calculated for $C_{16}H_{23}BO_2Cl$ [M+H]$^+$ 293.1474, found 293.1474.
R_f = 0.24 (1% EtOAc/hexane).

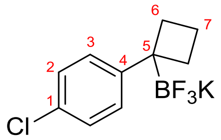

Potassium (1-(4-chlorophenyl)cyclobutyl)trifluoroborate: To a solution of 2-(1-(4-chlorophenyl)cyclobutyl)-4,4,5,5-tetramethyl-1,3,2-dioxaborolane (0.146 g, 0.5 mmol) in MeOH (2.5 mL) was added saturated aqueous KHF_2 solution (0.4 mL) and then stirred vigorously at r.t. for 30 min. The reaction mixture was evaporated under reduced pressure (50 °C water bath temperature). The residue was redissolved in MeOH/water (4 mL, 3:2) and evaporated under reduced pressure, repeating this process five times to remove pinacol. Hot acetone (5 × 5 mL) was used to dissolve the trifluoroborate salt, filtering through a hot fritted funnel to remove inorganic salts. The filtrate was then evaporated under reduced pressure to provide the title compound as a white amorphous solid (0.136 g, 0.499 mmol, 99%), m.p. >250 °C.

^{1}H NMR (600 MHz, MeCN-d_3): δ 7.13 (d, J = 8.4 Hz, 2 H, H2), 6.95 (d, J = 8.4 Hz, 2 H, H3), 2.39 – 2.32 (m, 2 H, H6a), 2.16 – 2.07 (m, 1 H, H7a), 2.01 – 1.94 (m, 2 H, H6b), 1.70 (qt, J = 9.7, 3.2 Hz, 1 H, H7b).
^{13}C NMR (150 MHz, MeCN-d_3): δ 156.9 (C4), 128.1 (C3), 127.9 (C1), 127.6 (C2), 32.6 (C6), 18.5 (C7). (C5 broadened by quadrupolar relaxation with boron).
^{19}F NMR (376 MHz, MeCN-d_3): δ -152.2 (br s, 3 F, BF_3).
FTIR (ν_{max}, cm^{-1}): 1151 (m), 959 (s), 927 (s), 803 (m), 758 (m).
HRMS (ESI): calculated for $C_{10}H_{10}BF_3Cl$ $[M-K]^-$ 233.0522, found 233.0520.

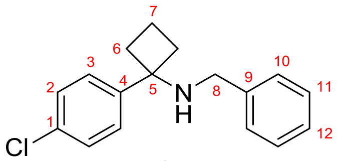

6

***N*-benzyl-1-(4-chlorophenyl)cyclobutan-1-amine (6):** To a sealed vial containing a suspension of potassium (1-(4-chlorophenyl)cyclobutyl)trifluoroborate (54.5 mg, 0.2 mmol, 1.0 equiv.) in anhydrous DCE (0.4 mL) was added $SiCl_4$ (0.05 mL, 0.4 mmol, 2.0 equiv.) and the mixture stirred further at r.t. for 2 h. Benzyl azide (0.05 mL, 0.4 mmol, 2.0 equiv.) was then added and the mixture heated to 80 °C and stirred for 1 h. The mixture was cooled to r.t., diluted with THF (0.4 mL), quenched with 2 M aqueous NaOH solution and then stirred further for 1 h. The mixture was extracted with Et_2O (3 × 10 mL) and the combined organic extracts washed with brine (10 mL), dried ($MgSO_4$) and evaporated under reduced pressure. The residue was purified by silica gel column chromatography (eluent: 5% → 10% EtOAc/hexane) to provide the title compound as a yellow oil (37.9 mg, 0.139 mmol, 70%).

^{1}H NMR (600 MHz, $CDCl_3$): δ 7.40 (d, J = 8.5 Hz, 2 H, H2), 7.34 (d, J = 8.5 Hz, 2 H, H3), 7.31 – 7.27 (m, 2 H, H11), 7.27 – 7.24 (m, 2 H, H10), 7.22 (t, J = 7.0 Hz, 1 H, H12), 3.36 (s, 2 H, H8), 2.47 – 2.40 (m, 2 H, H6a), 2.26 – 2.18 (m, 2 H, H6b), 2.09 – 2.00 (m, 1 H, H7a), 1.85 – 1.76 (m, 1 H, H7b), 1.60 (br s, 1 H, NH).
^{13}C NMR (150 MHz, $CDCl_3$): δ 145.1 (C4), 140.9 (C9), 132.2 (C1), 128.5 (C11), 128.4 (C3), 128.3 (C10), 127.9 (C2), 127.0 (C12), 63.0 (C5), 47.7 (C8), 34.3 (C6), 14.8 (C7).
FTIR (ν_{max}, cm^{-1}): 2979 (w), 2939 (w), 2839 (w), 1602 (w), 1492 (m), 1453 (m), 1398 (w), 1357 (w), 1286 (w), 1244 (w), 1154 (m), 1092 (s), 1058 (w), 1027 (w), 1012 (s), 908 (w), 826 (s).
HRMS (ESI): calculated for $C_{17}H_{19}NCl$ $[M+H]^+$ 272.1201, found 272.1210.
$\boldsymbol{R_f}$ = 0.28 (10% EtOAc/hexane).

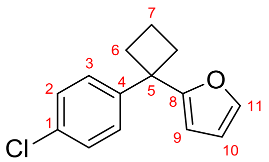

7

2-(1-(4-chlorophenyl)cyclobutyl)furan (7): A solution of furan (0.03 mL, 0.4 mmol, 2.0 equiv.) in anhydrous THF (1 mL) was cooled to -78 °C. ^{n}BuLi (0.21 mL, 0.4 mmol, 2.0 equiv., 1.92 M in hexanes) was added slowly dropwise, then the mixture warmed to r.t. and stirred further for 1 h. The mixture was cooled down again to -78 °C and a solution of 2-(1-(4-chlorophenyl)cyclobutyl)-4,4,5,5-tetramethyl-1,3,2-dioxaborolane (64.5 mg, 0.2 mmol, 1.0 equiv.) in anhydrous THF (0.3 mL) was added dropwise. The flask containing the Bpin ester was washed further with THF (0.1 mL) and the washings were added to the reaction mixture, which was then stirred further at -78 °C for 2 h. A solution of NBS (71.2 mg,

0.4 mmol, 2.0 equiv.) in anhydrous THF (1.0 mL) was then added dropwise and the mixture stirred further at -78 °C for 1 h. The reaction was quenched with saturated aqueous $Na_2S_2O_3$ solution (3 mL), warmed to r.t. and then extracted with CH_2Cl_2 (3 × 10 mL). The combined organic extracts were dried ($MgSO_4$) and evaporated under reduced pressure. The residue was purified by silica gel column chromatography (eluent: hexane) to provide the title compound as a white amorphous solid (22.1 mg, 0.095 mmol, 47%), m.p. 57-59 °C.

^{1}H NMR (600 MHz, $CDCl_3$): δ 7.32 (dd, *J* = 1.8, 0.8 Hz, 1 H, H11), 7.28 (d, *J* = 8.6 Hz, 2 H, H2), 7.21 (d, *J* = 8.6 Hz, 2 H, H3), 6.27 (dd, *J* = 3.2, 1.8 Hz, 1 H, H10), 5.95 (dd, *J* = 3.2, 0.8 Hz, 1 H, H9), 2.74 – 2.66 (m, 2 H, H6a), 2.64 – 2.56 (m, 2 H, H6b), 2.21 – 2.11 (m, 1 H, H7a), 1.96 – 1.87 (m, 1 H, H7b).
^{13}C NMR (150 MHz, $CDCl_3$): δ 160.8 (C8), 145.7 (C4), 141.8 (C11), 131.9 (C1), 128.4 (C2), 127.9 (C3), 110.1 (C10), 104.9 (C9), 46.6 (C5), 33.7 (C6), 16.9 (C7).
FTIR (ν_{max}, cm^{-1}): 2987 (m), 2945 (m), 1596 (w), 1492 (s), 1400 (w), 1294 (w), 1220 (w), 1154 (m), 1093 (s), 1013 (s), 919 (w), 884 (w), 831 (m), 803 (w), 775 (w).
HRMS (ESI): calculated for $C_{14}H_{14}OCl$ $[M+H]^+$ 233.0728, found 233.0724.
R_f = 0.44 (hexane).

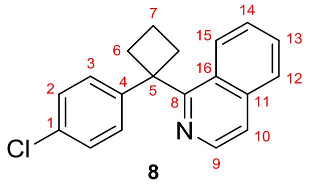

8

1-(1-(4-chlorophenyl)cyclobutyl)isoquinoline (8): A solution of 1-isoquinolinecarbonitrile (38.5 mg, 0.25 mmol, 1.0 equiv.), 2-(1-(4-chlorophenyl)cyclobutyl)-4,4,5,5-tetramethyl-1,3,2-dioxaborolane (87.8 mg, 0.30 mmol, 1.2 equiv.) and $[Ir(dF(CF_3)ppy)_2(dtbpy)]PF_6$ (2.8 mg, 2.5 µmol, 0.01 equiv.) in acetone (1 mL) was pumped at a flow rate of 0.1 mL min^{-1} through a Vapourtec UV-150 photochemical reactor (10 mL reactor volume, FEP tubing), irradiated by 420 nm LEDs (17 W total output power), held at 60 °C and the reactor output was monitored using a FlowIR® device. When the FlowIR® showed that the reaction mixture was starting to exit the output stream, the output was collected until the reaction plug finished eluting from the reactor. The reaction mixture was then evaporated under reduced pressure and the residue purified by silica gel column chromatography (eluent: 5% → 10% EtOAc/hexane) to provide the title compound as a colourless gum (35.9 mg, 0.122 mmol, 49%).

^{1}H NMR (600 MHz, $CDCl_3$): δ 8.56 (d, *J* = 5.7 Hz, 1 H, H9), 7.78 (d, *J* = 8.2 Hz, 1 H, H12), 7.67 (d, *J* = 8.6 Hz, 1 H, H15), 7.56 – 7.52 (m, 2 H, H10 and H13), 7.38 – 7.33 (m, 3 H, H2 and H14), 7.23 (d, *J* = 8.7 Hz, 2 H, H3), 3.25 – 3.15 (m, 2 H, H6a), 2.85 – 2.75 (m, 2 H, H6b), 2.26 – 2.15 (m, 1 H, H7a), 2.00 – 1.91 (m, 1 H, H7b).
^{13}C NMR (150 MHz, $CDCl_3$): δ 165.7 (C8), 146.1 (C4), 141.2 (C9), 137.2 (C11), 131.7 (C1), 129.5 (C13), 128.7 (C3), 127.7 (C12), 127.5 (C2), 126.6 (C15), 126.5 (C14), 125.6 (C16), 119.9 (C10), 53.3 (C5), 34.9 (C6), 16.7 (C7).
FTIR (ν_{max}, cm^{-1}): 3051 (w), 2943 (w), 2867 (w), 1622 (w), 1586 (w), 1558 (w), 1489 (m), 1458 (w), 1399 (w), 1372 (w), 1357 (w), 1323 (w), 1289 (w), 1259 (w), 1216 (w), 1164 (w), 1141 (w), 1123 (w), 1094 (m), 1045 (w), 1012 (m), 870 (w), 821 (s), 800 (m).
HRMS (ESI): calculated for $C_{19}H_{17}NCl$ $[M+H]^+$ 294.1044, found 294.1041.
R_f = 0.46 (10% EtOAc/hexane).

6. Synthesis of (±)-baclofen

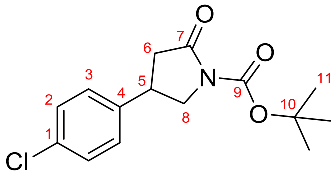

9

***tert*-butyl 4-(4-chlorophenyl)-2-oxopyrrolidine-1-carboxylate (9):** To a mixture of *tert*-butyl 3-(4-chlorophenyl)pyrrolidine-1-carboxylate (73.8 mg, 0.262 mmol) and $RuO_2 \cdot H_2O$ (3 mg) in EtOAc (1 mL) was added 10% aqueous $NaIO_4$ (3 mL). The mixture was stirred vigorously at r.t. for 3 h, then extracted with EtOAc (3 × 5 mL). The combined organic extracts were treated with 2-propanol (2 mL) and stirred further at r.t. for 2 h to destroy any remaining RuO_4 oxidant, then the mixture filtered through a pad of Celite, eluting with EtOAc. The filtrate was then dried ($MgSO_4$) and evaporated under reduced pressure. The residue was purified by silica gel column chromatography (eluent: 25% EtOAc/hexane) to provide the title compound as a white amorphous solid (39.0 mg, 0.132 mmol, 50%), m.p. 101-103 °C (lit. m.p.[9] 103-103.5 °C). Data are consistent with a reported example.[9]

^{1}H NMR (600 MHz, $CDCl_3$): δ 7.31 (d, *J* = 8.4 Hz, 2 H, H2), 7.16 (d, *J* = 8.4 Hz, 2 H, H3), 4.14 (dd, *J* = 10.9, 8.2 Hz, 1 H, H6a), 3.64 (dd, *J* = 10.9, 8.4 Hz, 1 H, H6b), 3.55 – 3.46 (m, 1 H, H5), 2.88 (dd, *J* = 17.2, 8.5 Hz, 1 H, H8a), 2.65 (dd, *J* = 17.2, 9.7 Hz, 1 H, H8b), 1.52 (s, 9 H, H11).
^{13}C NMR (150 MHz, $CDCl_3$): δ 172.7 (C7), 149.9 (C9), 139.2 (C4), 133.3 (C1), 129.2 (C2), 128.2 (C3), 83.3 (C10), 53.0 (C8), 40.3 (C6), 35.9 (C5), 28.1 (C11).
FTIR (ν_{max}, cm^{-1}): 2980 (w), 2931 (w), 1783 (m, C=O), 1749 (m, C=O), 1713 (m, C=O), 1495 (m), 1457 (w), 1394 (w), 1367 (m), 1346 (m), 1310 (s), 1290 (s), 1256 (m), 1223 (w), 1149 (s), 1090 (m), 1039 (w), 1014 (m), 902 (w), 830 (m), 778 (m).
HRMS (ESI): calculated for $C_{15}H_{18}NO_3ClNa$ $[M+Na]^+$ 318.0867, found 318.0881.
R_f = 0.26 (25% EtOAc/hexane).

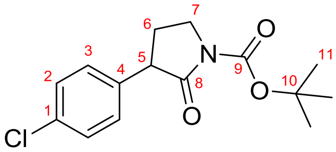

***tert*-butyl 3-(4-chlorophenyl)-2-oxopyrrolidine-1-carboxylate:** Following the oxidation of *tert*-butyl 3-(4-chlorophenyl)pyrrolidine-1-carboxylate, the title compound was also isolated as a white amorphous solid (22.4 mg, 0.076 mmol, 29%), m.p. 113-115 °C (lit. m.p.[9] 116-117 °C). Data are consistent with a reported example.[9]

^{1}H NMR (600 MHz, $CDCl_3$): δ 7.32 (d, *J* = 8.4 Hz, 2 H, H2), 7.21 (d, *J* = 8.4 Hz, 2 H, H3), 3.90 (ddd, *J* = 11.1, 8.5, 2.8 Hz, 1 H, H7a), 3.76 – 3.68 (m, 2 H, H5 and H7b), 2.46 (dddd, *J* = 12.8, 8.7, 7.1, 2.8 Hz, 1 H, H6a), 2.19 – 2.09 (m, 1 H, H6b), 1.54 (s, 9 H, H11).
^{13}C NMR (150 MHz, $CDCl_3$): δ 173.4 (C8), 150.5 (C9), 136.1 (C4), 133.5 (C1), 129.6 (C3), 129.0 (C2), 83.4 (C10), 49.3 (C5), 44.5 (C7), 28.2 (C11), 26.7 (C6).
FTIR (ν_{max}, cm^{-1}): 3012 (w), 2983 (w), 1778 (m, C=O), 1748 (m, C=O), 1714 (m, C=O), 1494 (m), 1457 (w), 1394 (w), 1367 (m), 1299 (s), 1256 (m), 1148 (s), 1091 (m), 1083 (m), 1041 (w), 1015 (m), 989 (m), 902 (m), 848 (m), 832 (m), 804 (w), 778 (m).
HRMS (ESI): calculated for $C_{15}H_{18}NO_3ClNa$ $[M+Na]^+$ 318.0867, found 318.0872.
R_f = 0.32 (25% EtOAc/hexane).

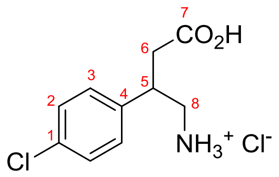

10

(±)-baclofen (10): *tert*-butyl 4-(4-chlorophenyl)-2-oxopyrrolidine-1-carboxylate (33.8 mg, 0.114 mmol) in 6 N aqueous HCl (5 mL) was refluxed for 16 h. The mixture was then evaporated under reduced pressure (60 °C water bath temperature) and dried under high vacuum at 60 °C to provide the title compound as an off-white amorphous solid (28.5 mg, 0.114 mmol, 99%), m.p. 212-214 °C (lit. m.p.[9] 215 °C). Data are consistent with a reported example.[9]

^{1}H NMR (600 MHz, D_2O): δ 7.45 (d, *J* = 8.4 Hz, 2 H, H2), 7.36 (d, *J* = 8.4 Hz, 2 H, H3), 3.49 – 3.36 (m, 2 H, H5 and H8a), 3.30 – 3.22 (dd, *J* = 12.3, 11.0 Hz, 1 H, H8b), 2.87 (dd, *J* = 16.2, 5.9 Hz, 1 H, H6a), 2.76 (dd, *J* = 16.2, 9.0 Hz, 1 H, H6b).
^{13}C NMR (150 MHz, D_2O): δ 175.2 (C7), 137.0 (C4), 133.3 (C1), 129.4 (C3), 129.2 (C2), 43.6 (C8), 39.4 (C5), 38.2 (C6).
FTIR (ν_{max}, cm^{-1}): 2915 (br, NH and OH), 1709 (s, C=O), 1598 (m), 1493 (s), 1414 (m), 1288 (w), 1177 (m), 1119 (m), 1091 (s), 1014 (m), 958 (w), 822 (s).
HRMS (ESI): calculated for $C_{10}H_{13}NO_2Cl$ [of free base, $M+H]^+$ 214.0629, found 214.0635.

7. Differential scanning calorimetry data

Differential scanning calorimetry data for oxadiazolines and selected diazo-containing compounds.

Compound	Start of decomposition / °C	Onset temperature / °C	Enthalpy of decomposition / J g^{-1}
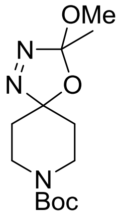	94	114	-540
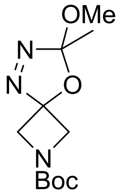	93	114	-835
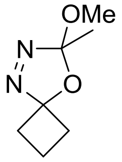	102	117	-673
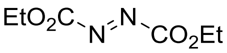	96	167	-1466
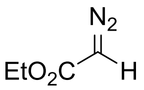 (with 15 wt% CH_2Cl_2)	50	79	-1762

Differential scanning calorimetry (DSC) data for three oxadiazolines indicate similar decomposition and onset temperatures, of *ca*. 90-100 °C and *ca*. 115 °C respectively, significantly higher than the operating temperature of the cross-coupling reaction conditions. Enthalpies of decomposition are also lower than compared to commonly utilised diazo-containing reagents, diethyl azodicarboxylate (DEAD) and ethyl diazoacetate.[25]

8. NMR spectra

8.1. NMR spectra for oxadiazolines

3-methoxy-3-methyl-4,8-dioxa-1,2-diazaspiro[4.5]dec-1-ene (1):

^{1}H NMR, 600 MHz, $CDCl_3$:

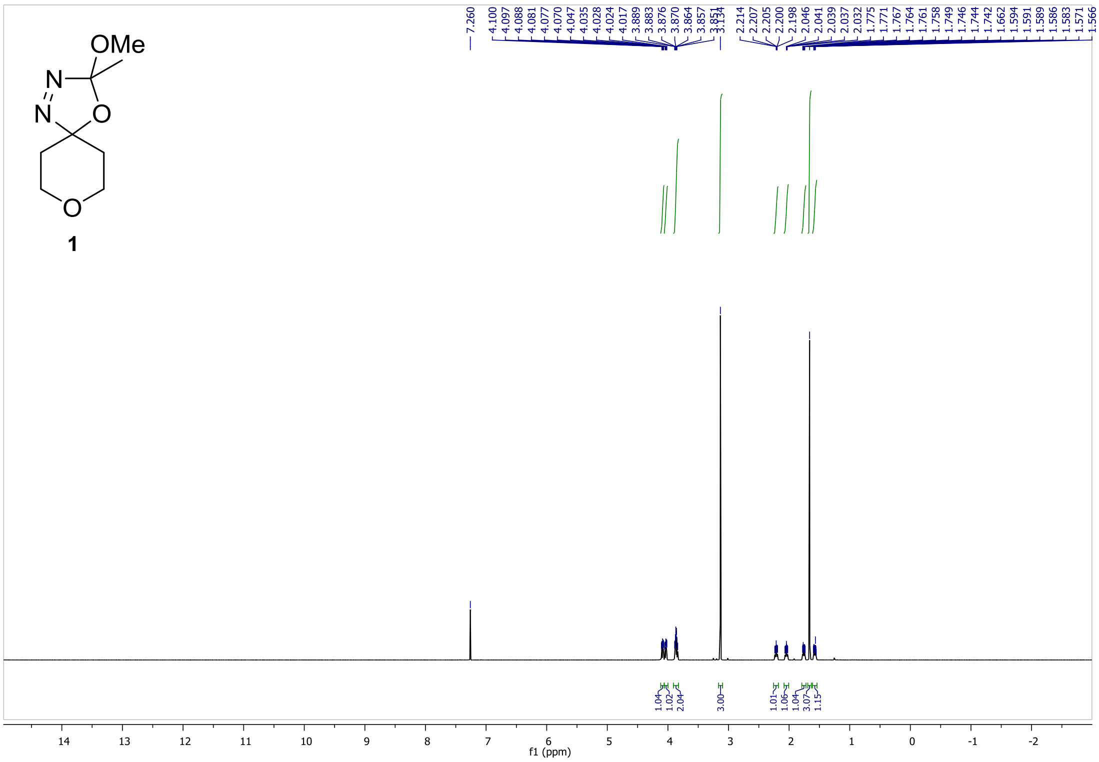

^{13}C NMR, 150 MHz, $CDCl_3$:

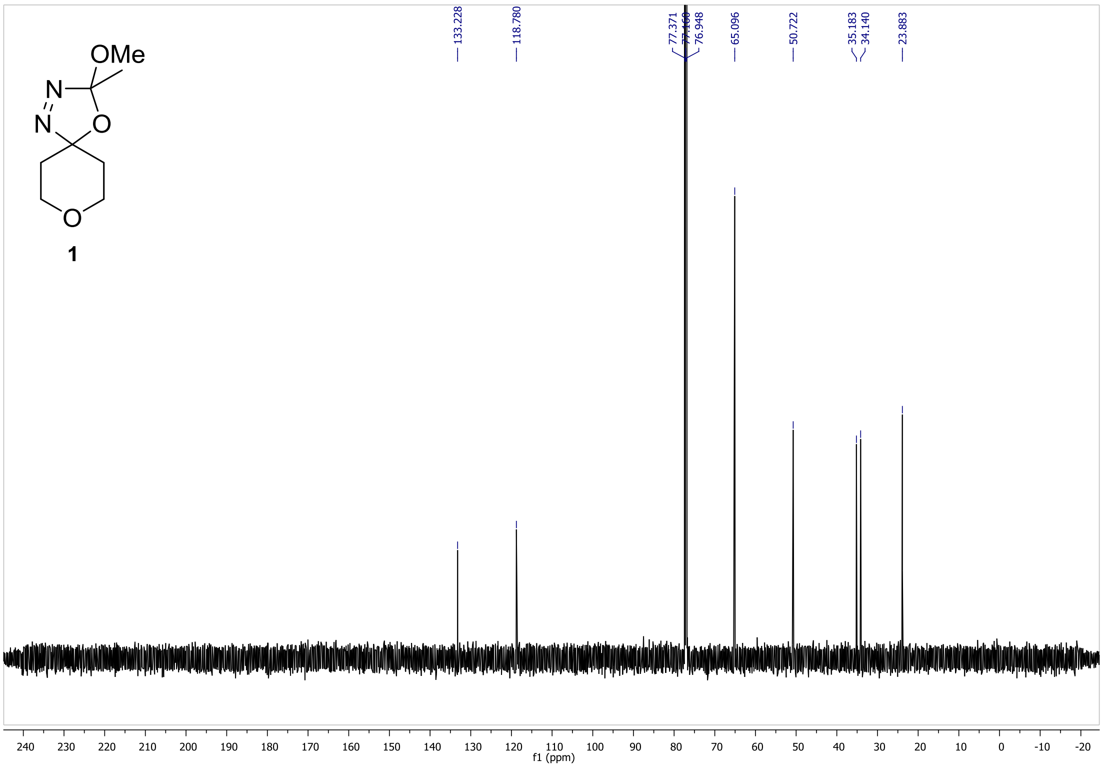

3-methoxy-3-methyl-4,7-dioxa-1,2-diazaspiro[4.4]non-1-ene:

^{1}H NMR, 600 MHz, $CDCl_3$:

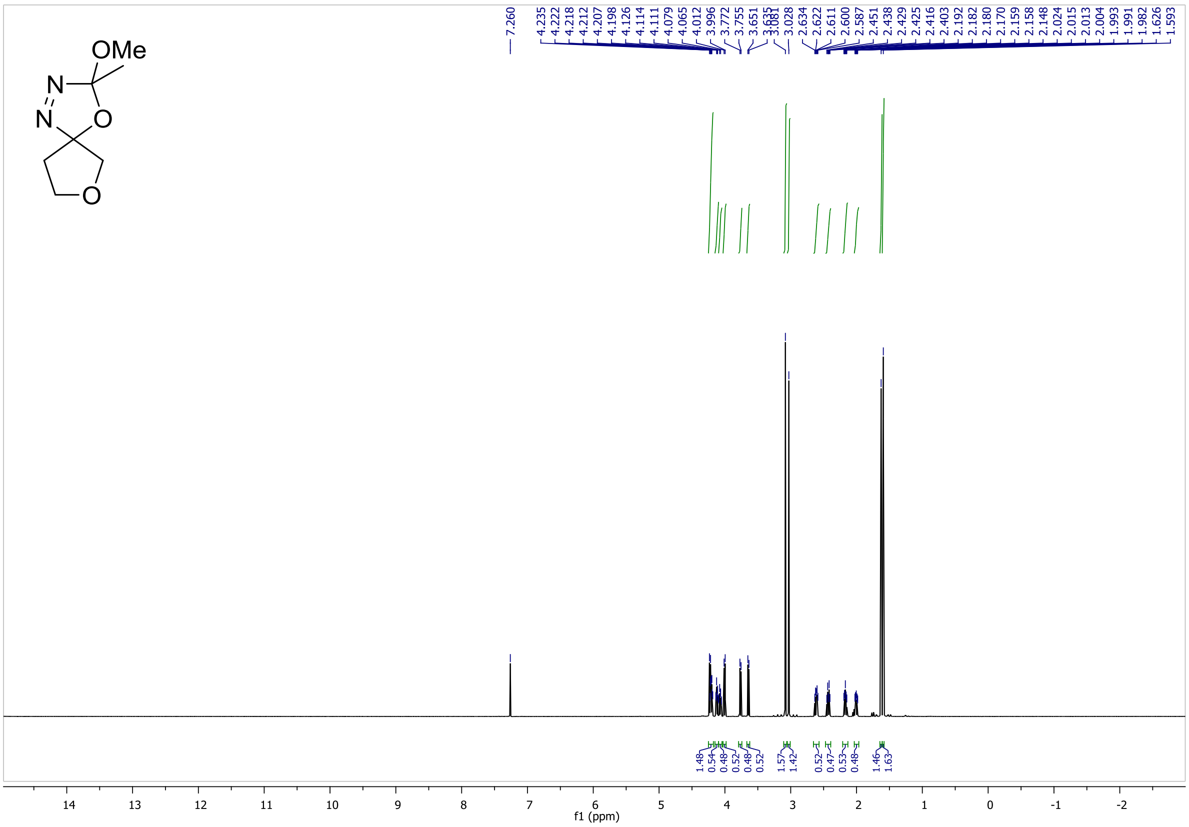

^{13}C NMR, 150 MHz, $CDCl_3$:

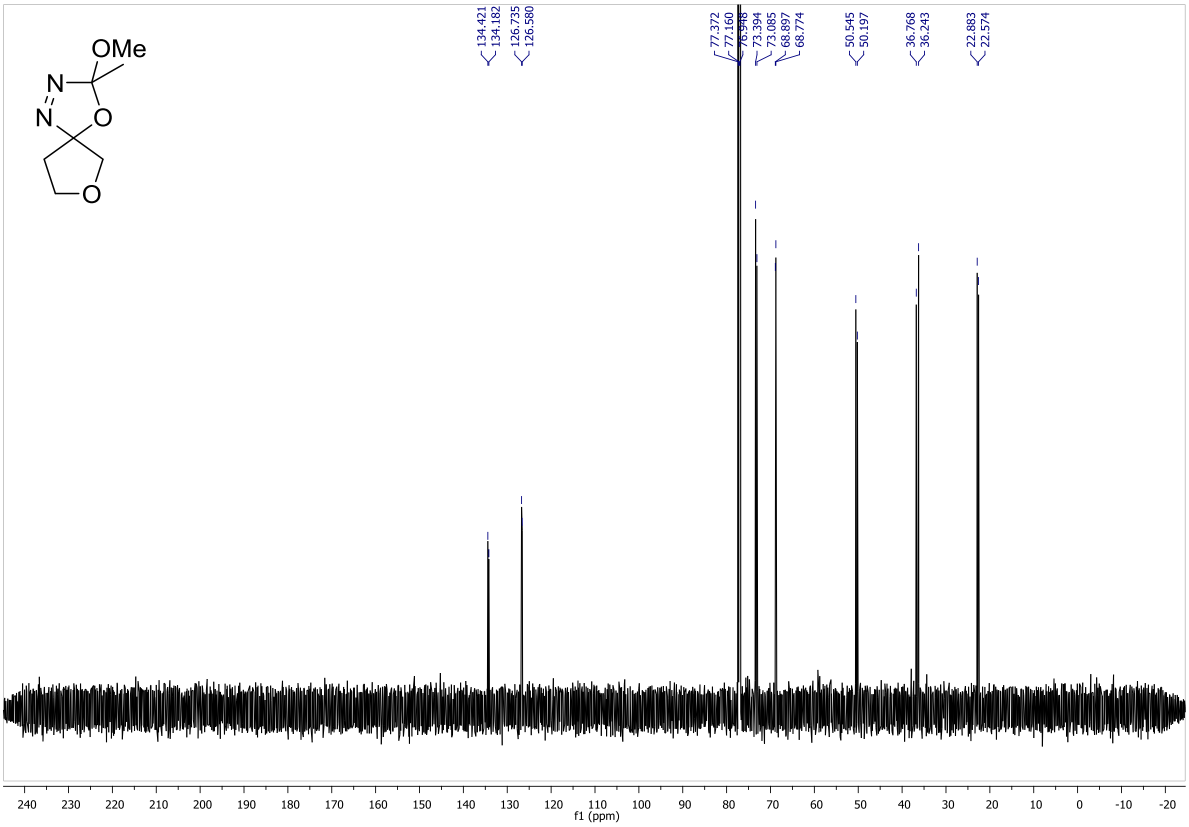

3-methoxy-3-methyl-4-oxa-8-thia-1,2-diazaspiro[4.5]dec-1-ene:

^{1}H NMR, 600 MHz, $CDCl_3$:

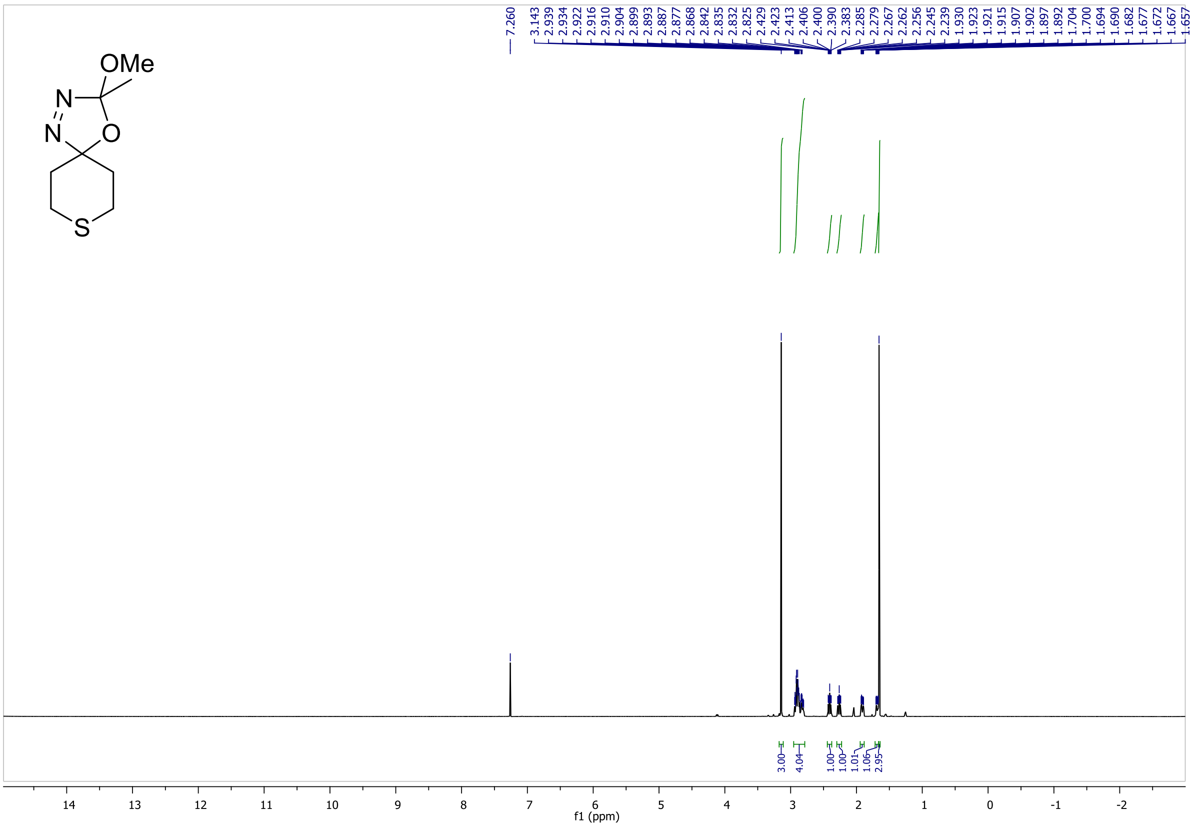

^{13}C NMR, 150 MHz, $CDCl_3$:

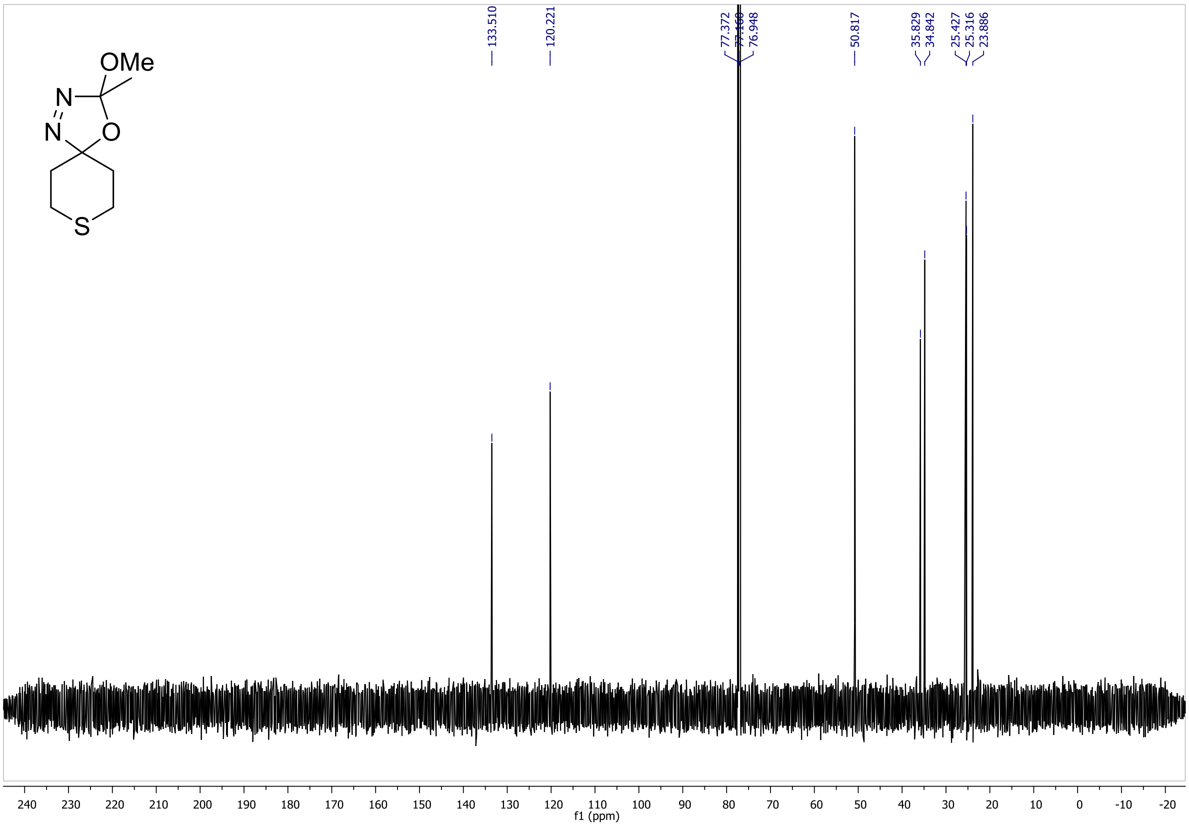

3-methoxy-3-methyl-4-oxa-7-thia-1,2-diazaspiro[4.4]non-1-ene:

^{1}H NMR, 600 MHz, $CDCl_3$:

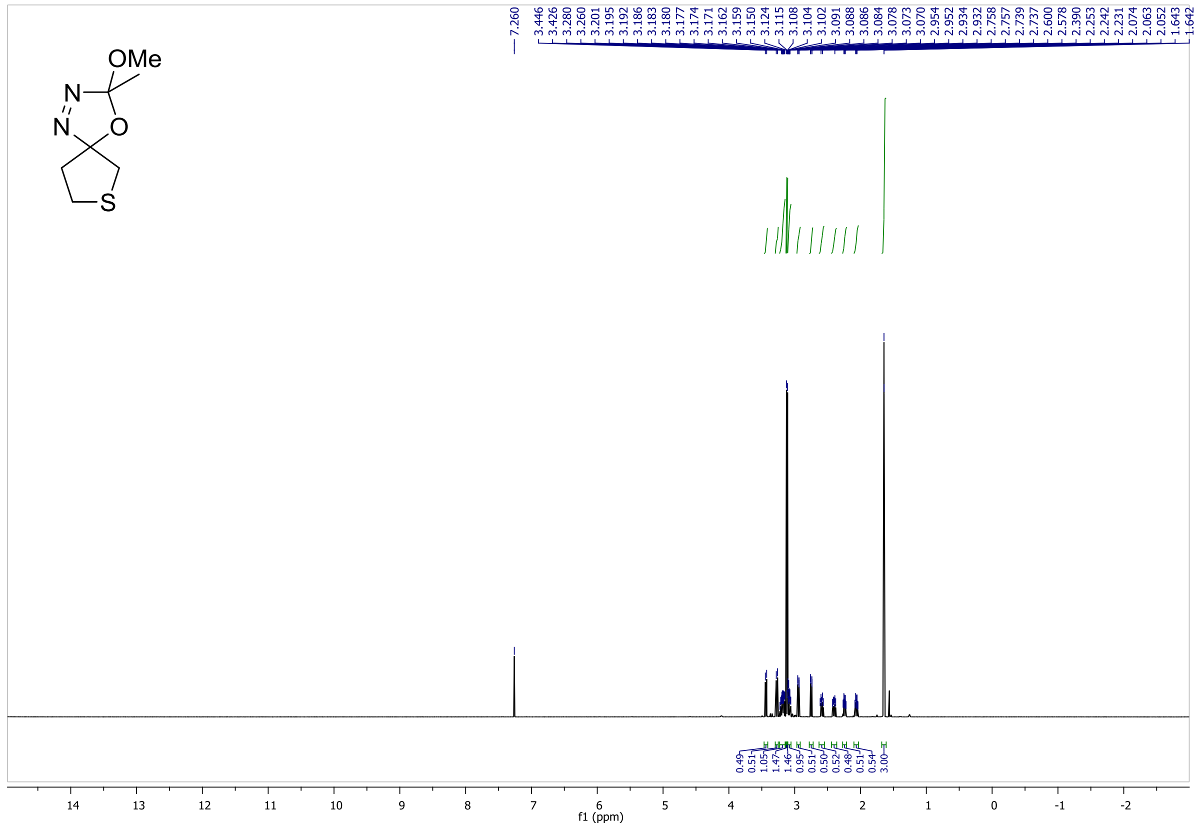

^{13}C NMR, 150 MHz, $CDCl_3$:

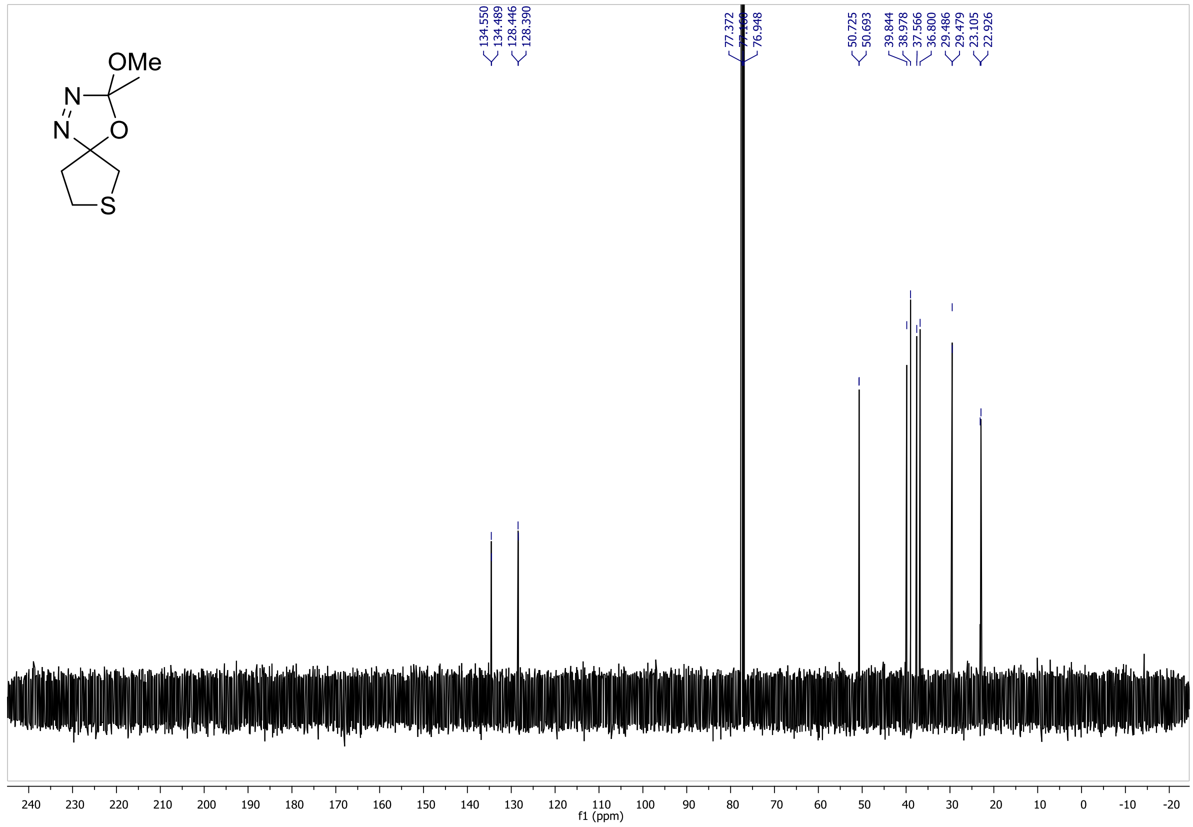

7-methoxy-7-methyl-8-oxa-2-thia-5,6-diazaspiro[3.4]oct-5-ene:

^{1}H NMR, 600 MHz, $CDCl_3$:

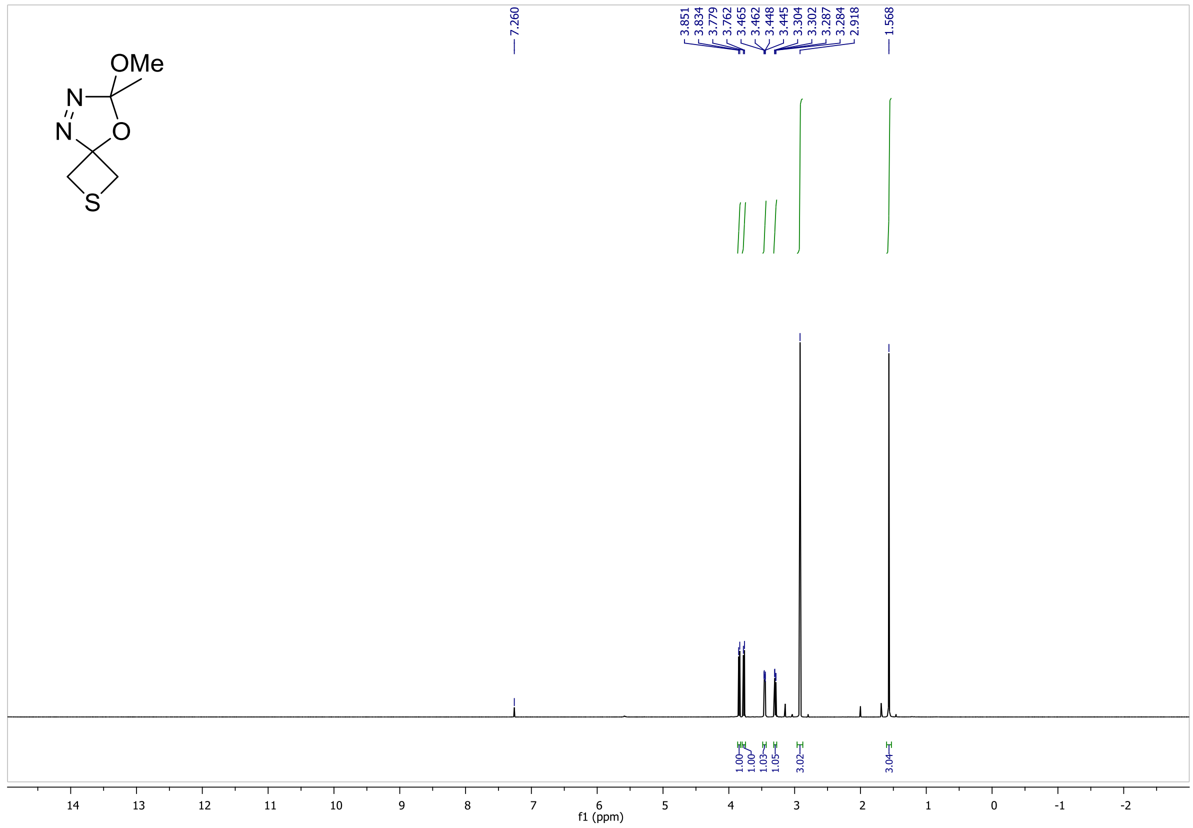

^{13}C NMR, 150 MHz, $CDCl_3$:

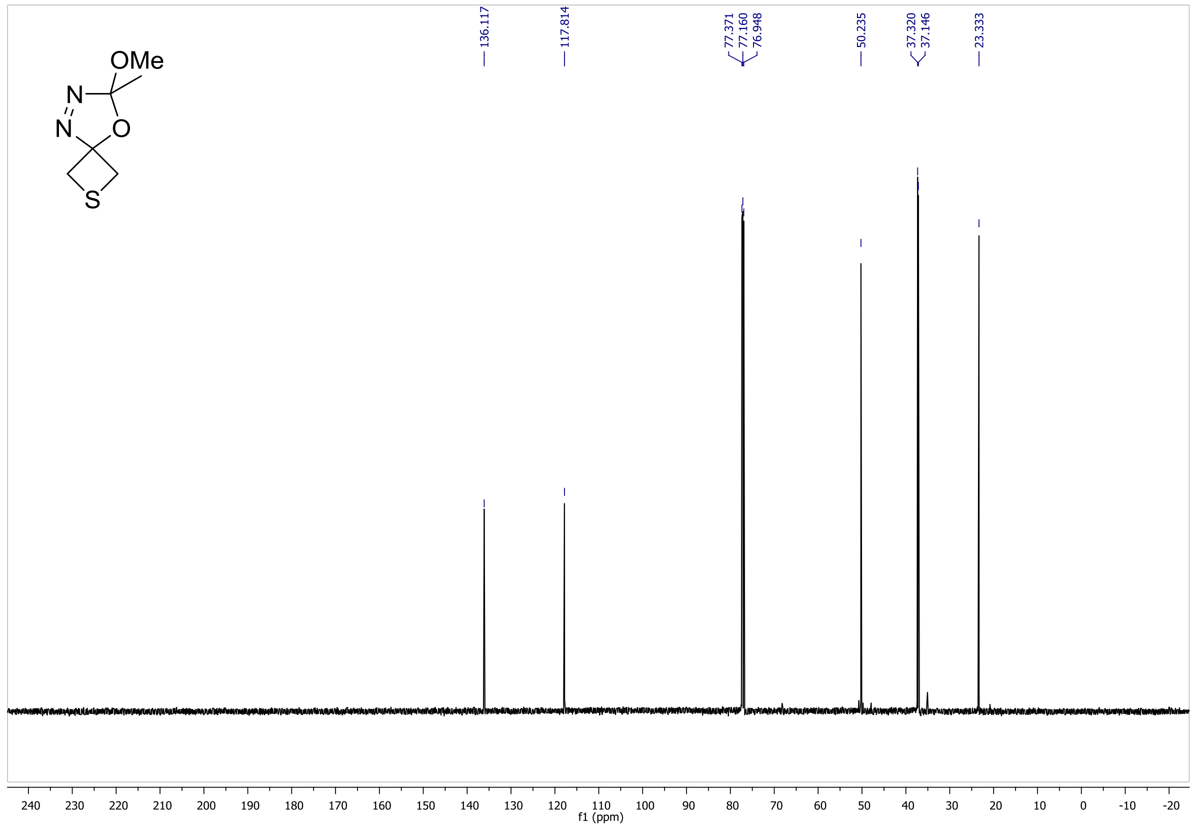

***tert*-butyl 3-methoxy-3-methyl-4-oxa-1,2,8-triazaspiro[4.5]dec-1-ene-8-carboxylate:**

^{1}H NMR, 600 MHz, $CDCl_3$:

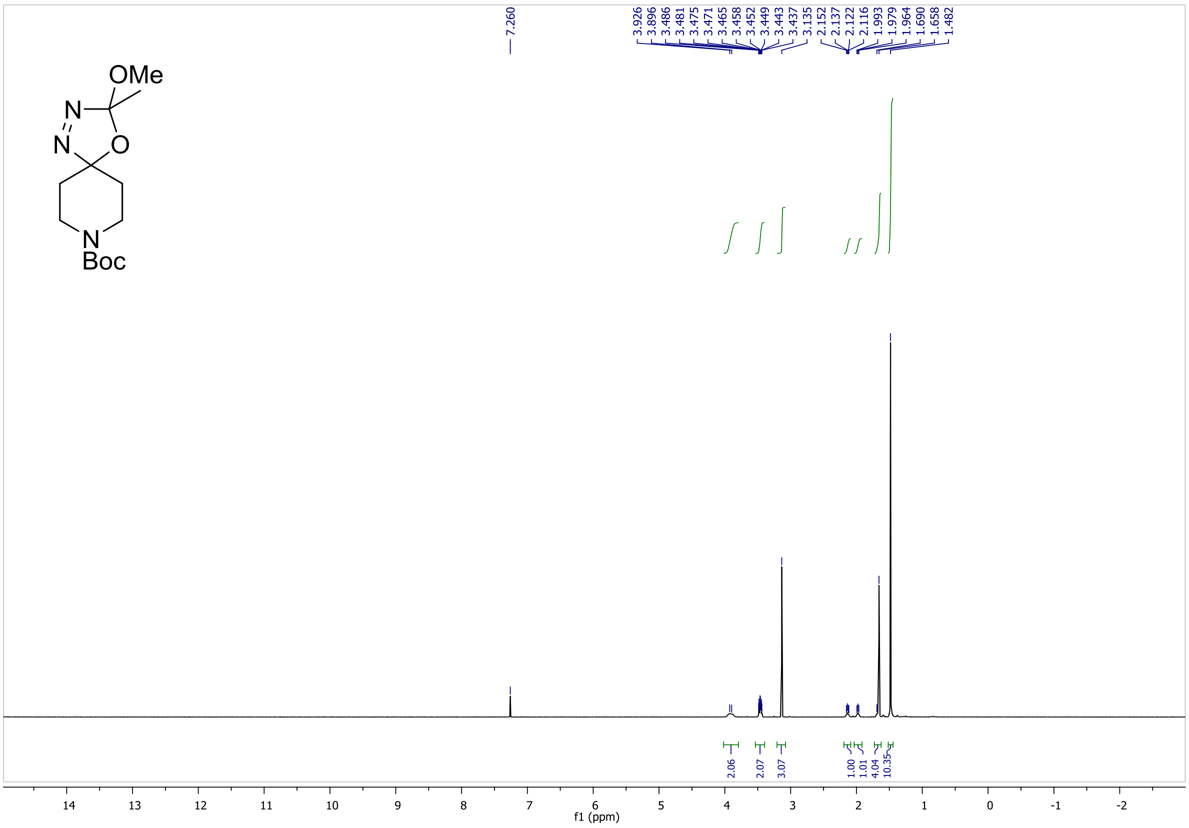

^{13}C NMR, 150 MHz, $CDCl_3$:

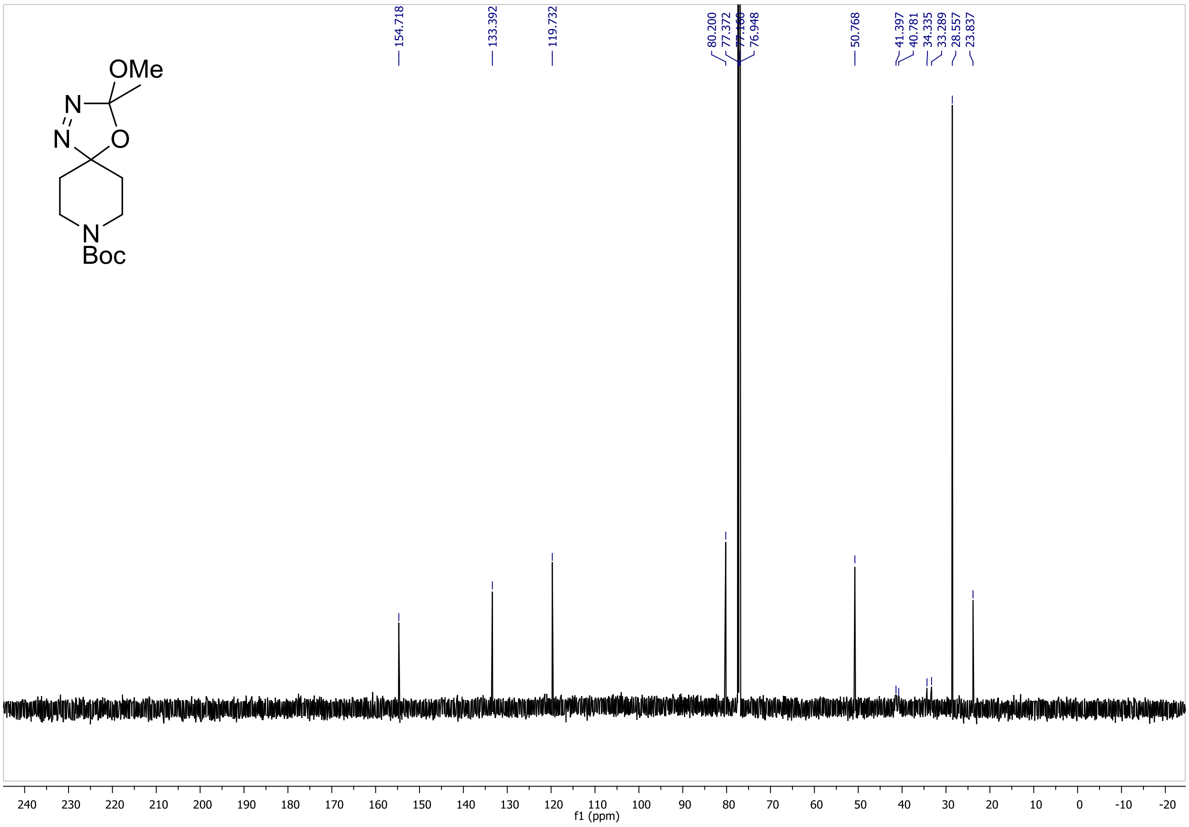

***tert*-butyl 3-methoxy-3-methyl-4-oxa-1,2,7-triazaspiro[4.4]non-1-ene-7-carboxylate:**

^{1}H NMR, 600 MHz, MeOD-d_4:

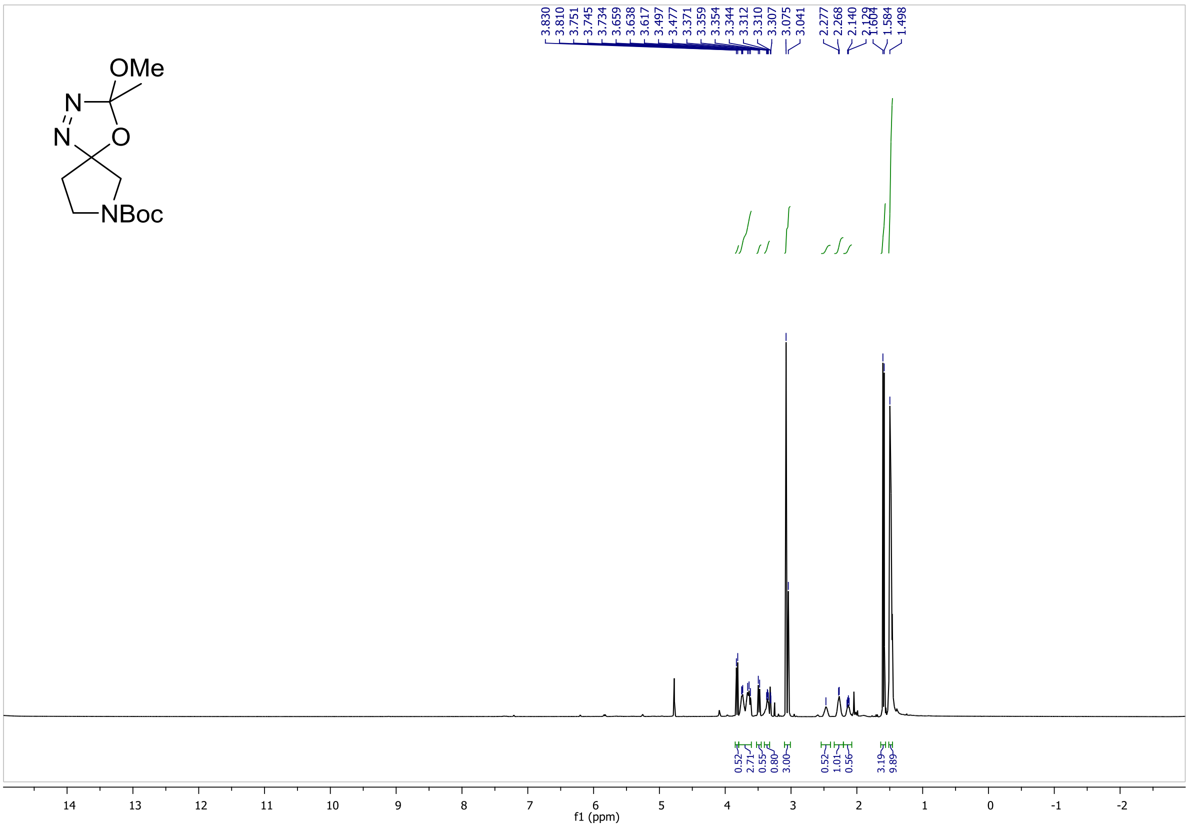

^{13}C NMR, 150 MHz, MeOD-d_4:

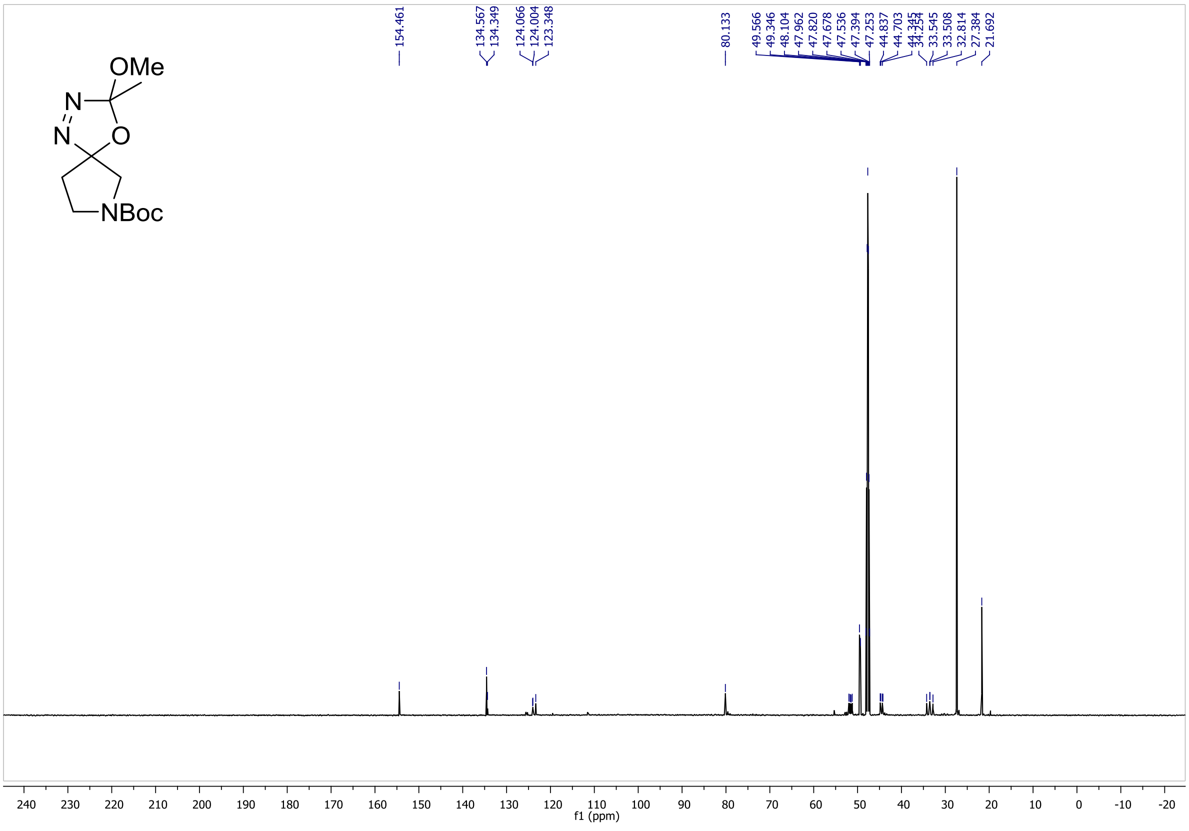

***tert*-butyl 7-methoxy-7-methyl-8-oxa-2,5,6-triazaspiro[3.4]oct-5-ene-2-carboxylate:**

^{1}H NMR, 600 MHz, $CDCl_3$:

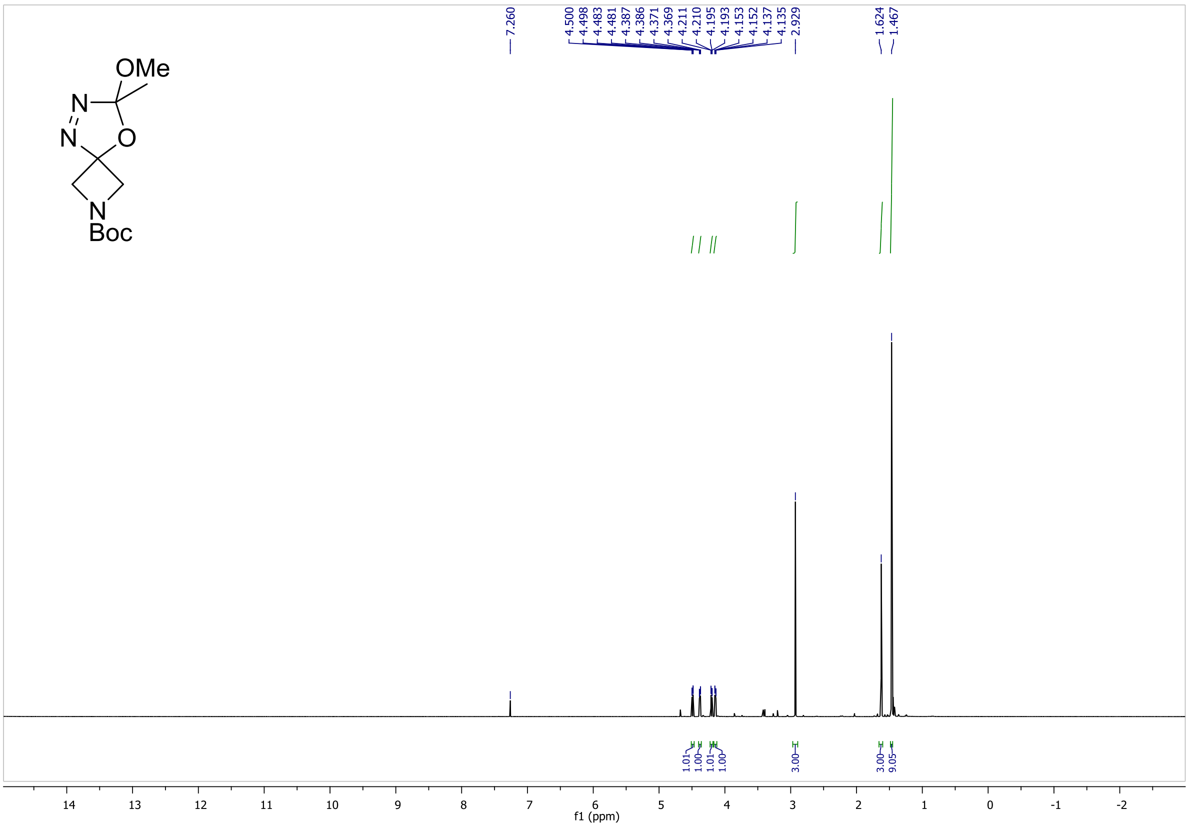

^{13}C NMR, 150 MHz, $CDCl_3$:

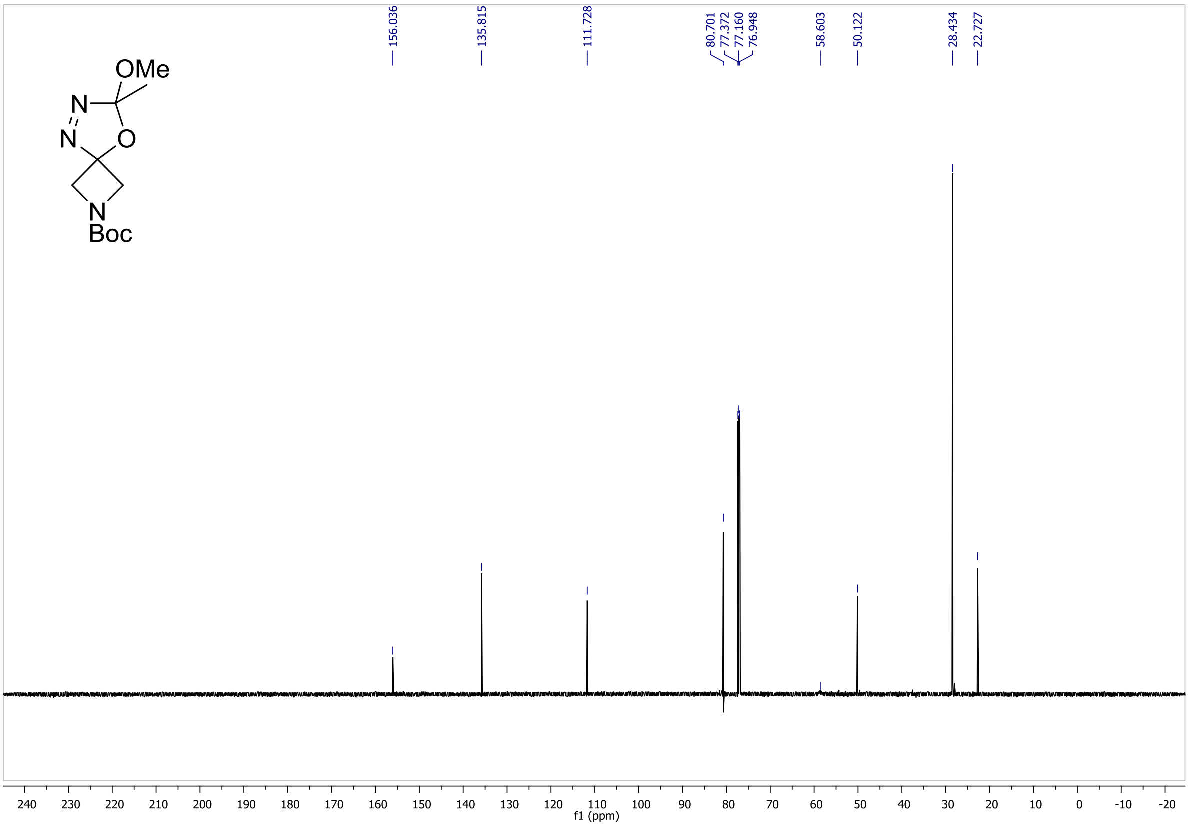

3-methoxy-3-methyl-4-oxa-1,2-diazaspiro[4.5]dec-1-ene:

^{1}H NMR, 600 MHz, $CDCl_3$:

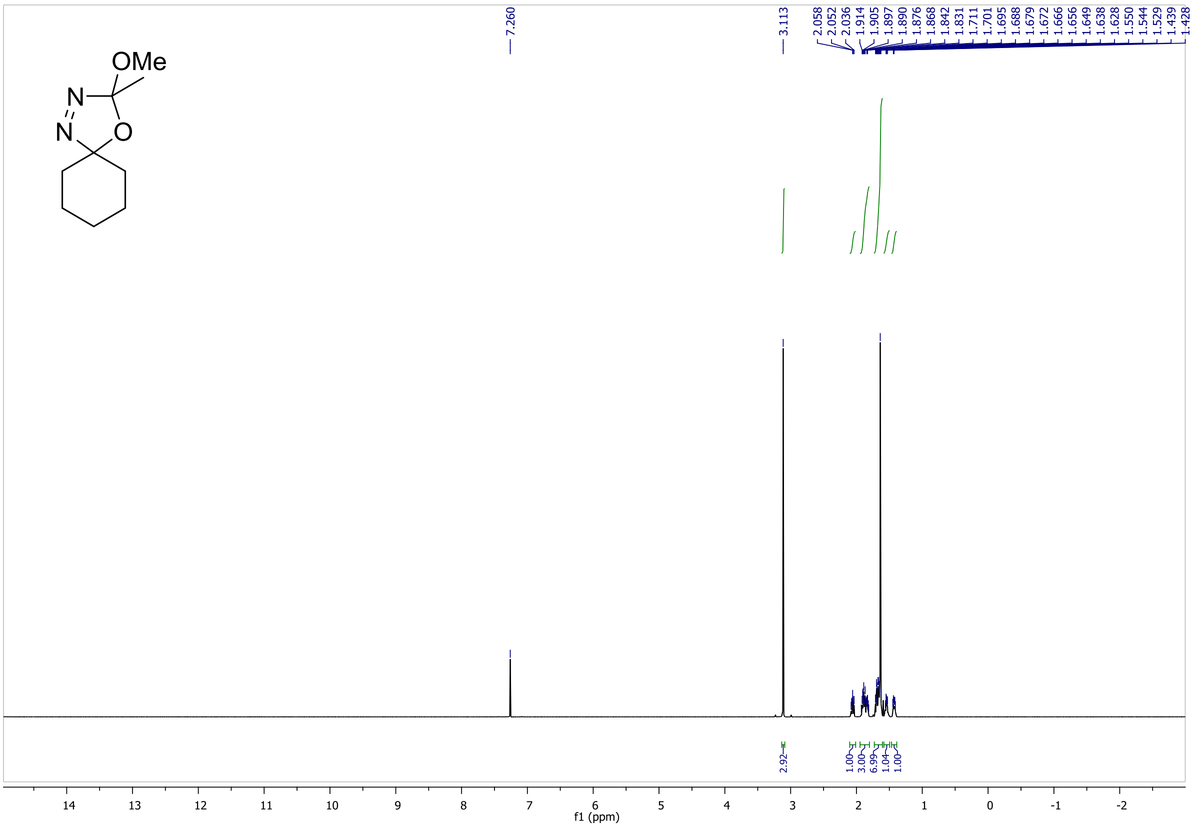

^{13}C NMR, 150 MHz, $CDCl_3$:

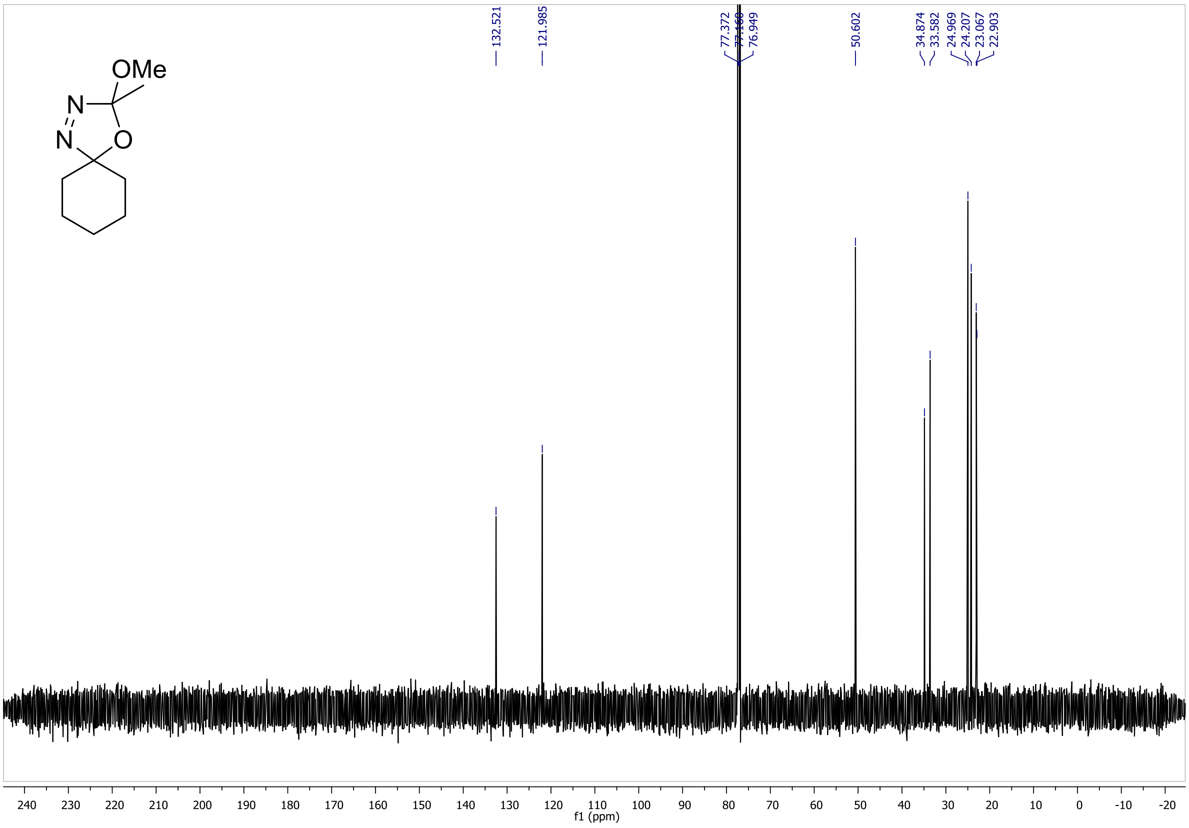

3-methoxy-3-methyl-4-oxa-1,2-diazaspiro[4.4]non-1-ene:

^{1}H NMR, 600 MHz, $CDCl_3$:

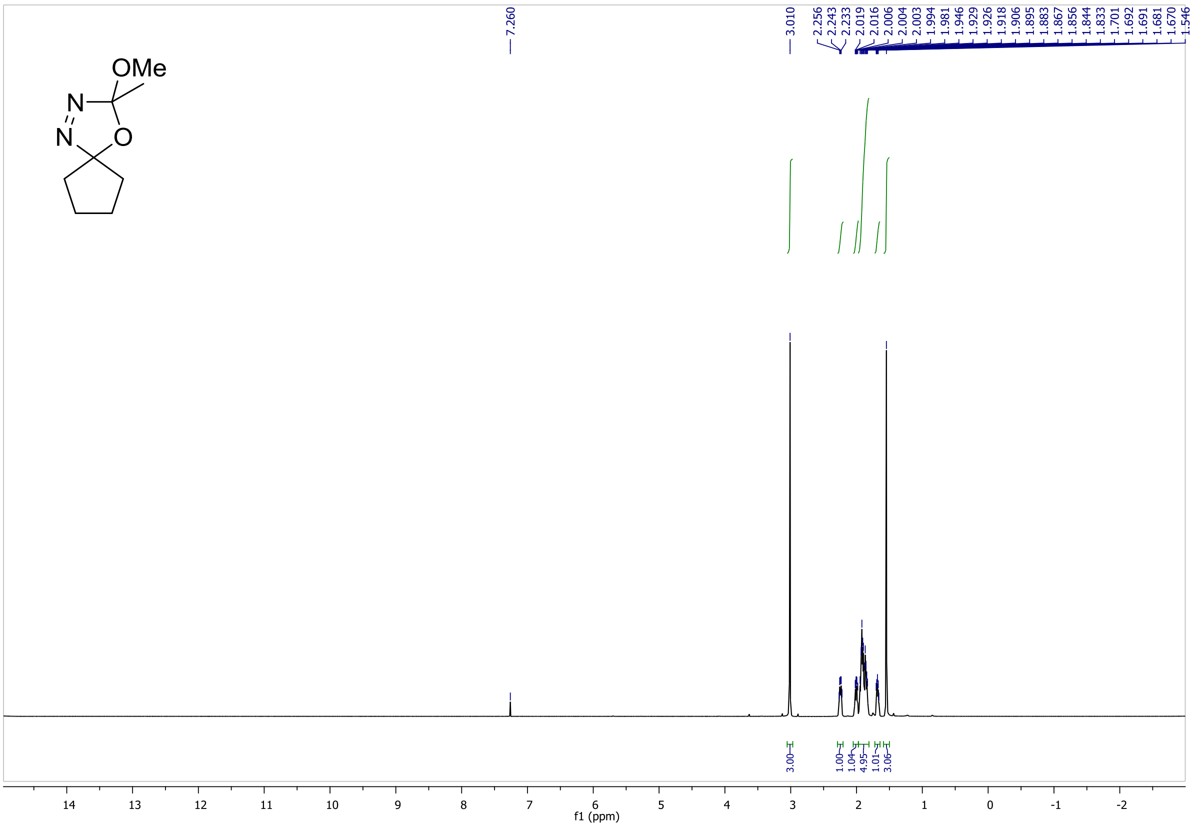

^{13}C NMR, 150 MHz, $CDCl_3$:

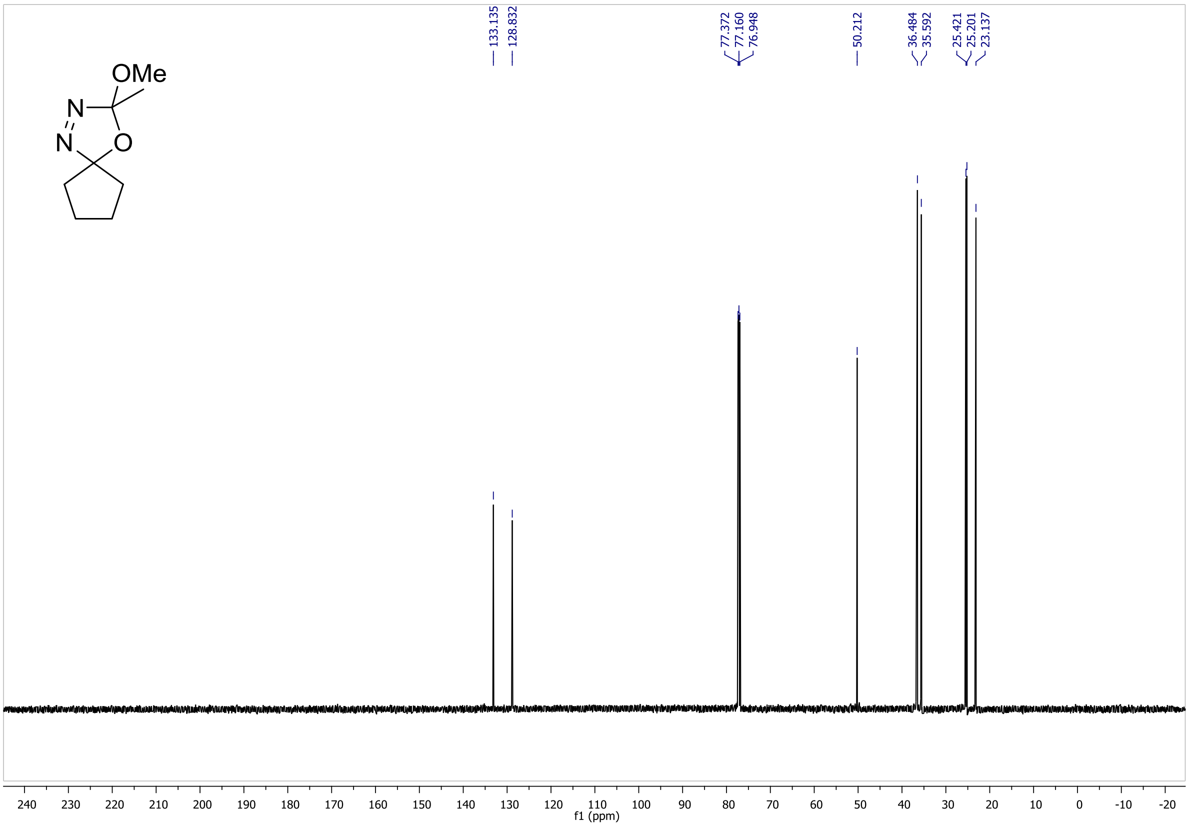

7-methoxy-7-methyl-8-oxa-5,6-diazaspiro[3.4]oct-5-ene:

^{1}H NMR, 600 MHz, $CDCl_3$:

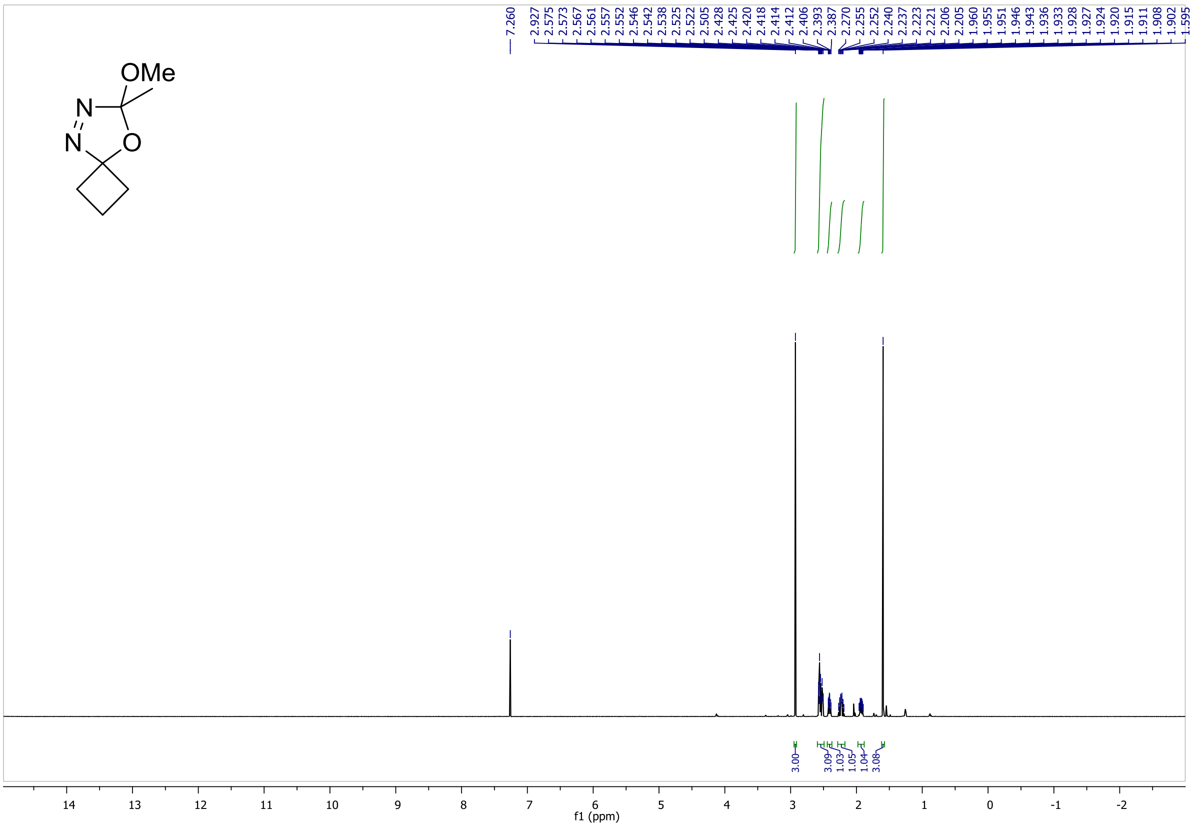

^{13}C NMR, 150 MHz, $CDCl_3$:

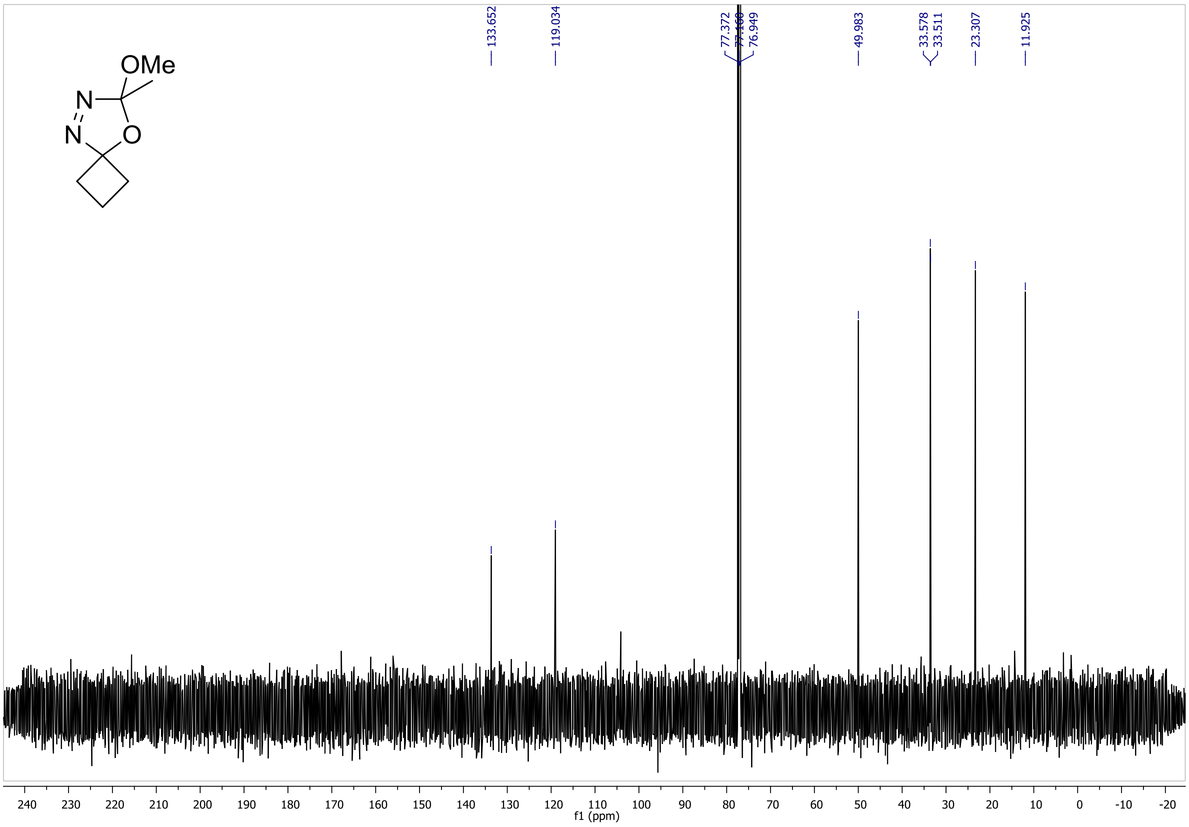

5'-methoxy-5'-methyl-3,4-dihydro-1*H*,5'*H*-spiro[naphthalene-2,2'-[1,3,4]oxadiazole]:

^{1}H NMR, 600 MHz, $CDCl_3$:

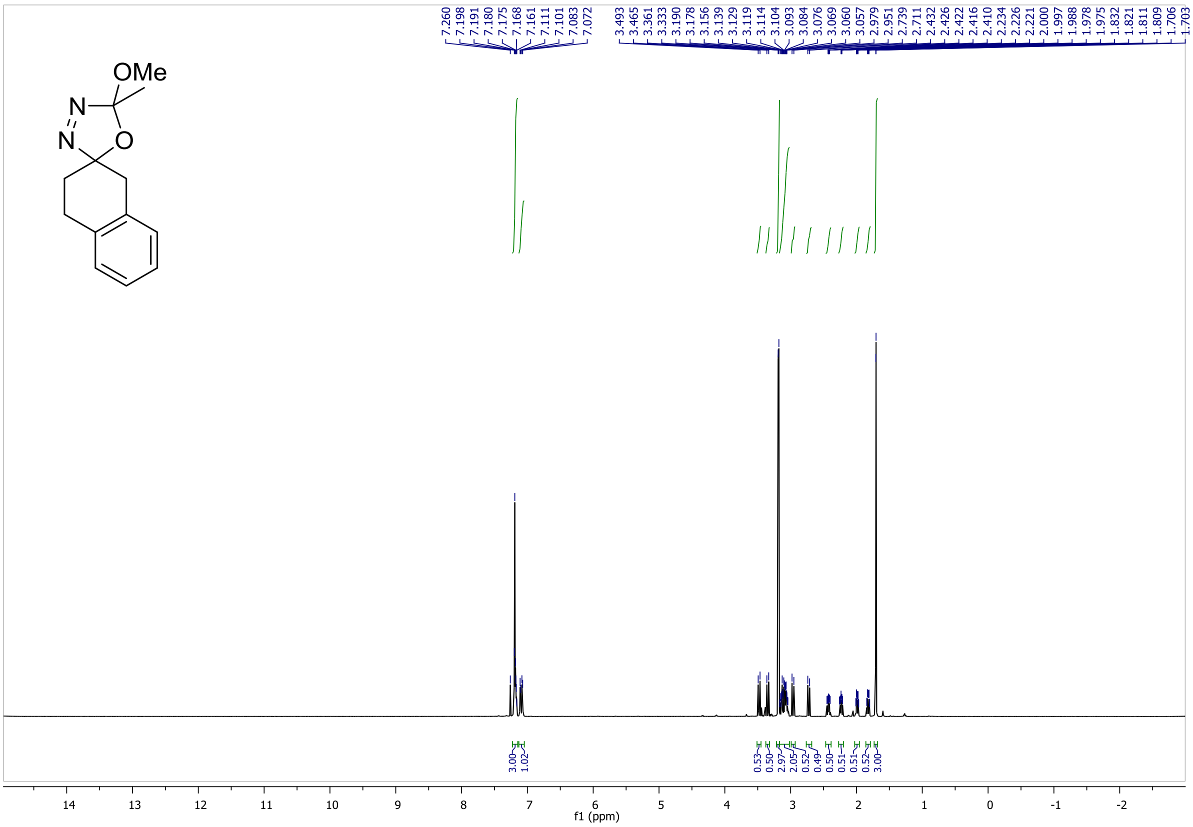

^{13}C NMR, 150 MHz, $CDCl_3$:

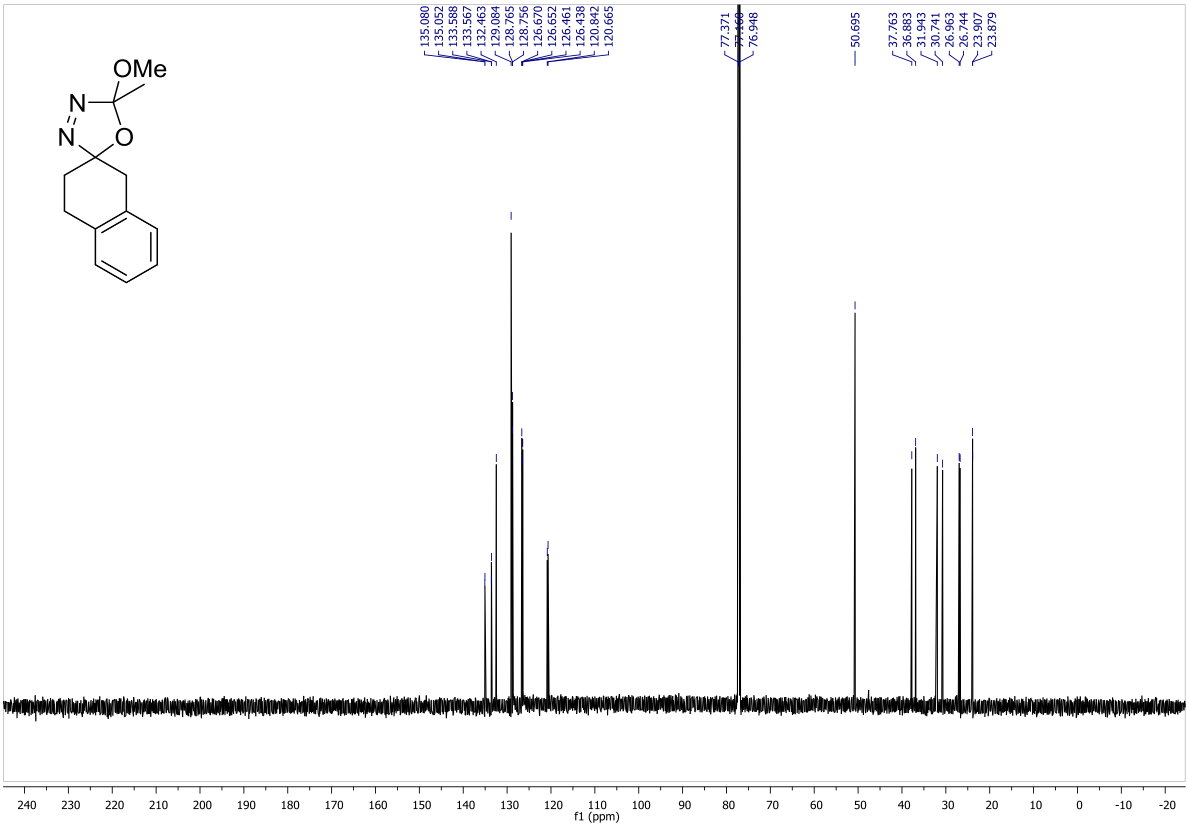

5'-methoxy-5'-methyl-5'*H*-spiro[adamantane-2,2'-[1,3,4]oxadiazole]:

^{1}H NMR, 600 MHz, $CDCl_3$:

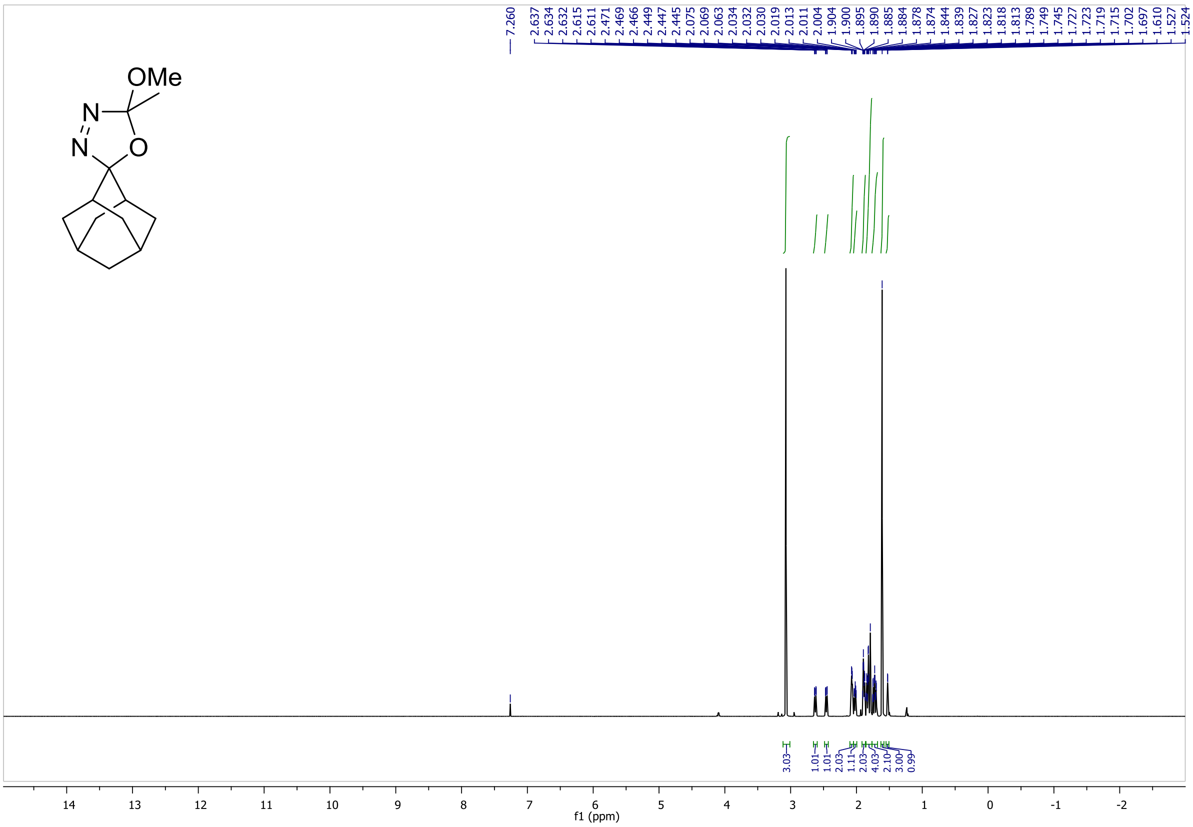

^{13}C NMR, 150 MHz, $CDCl_3$:

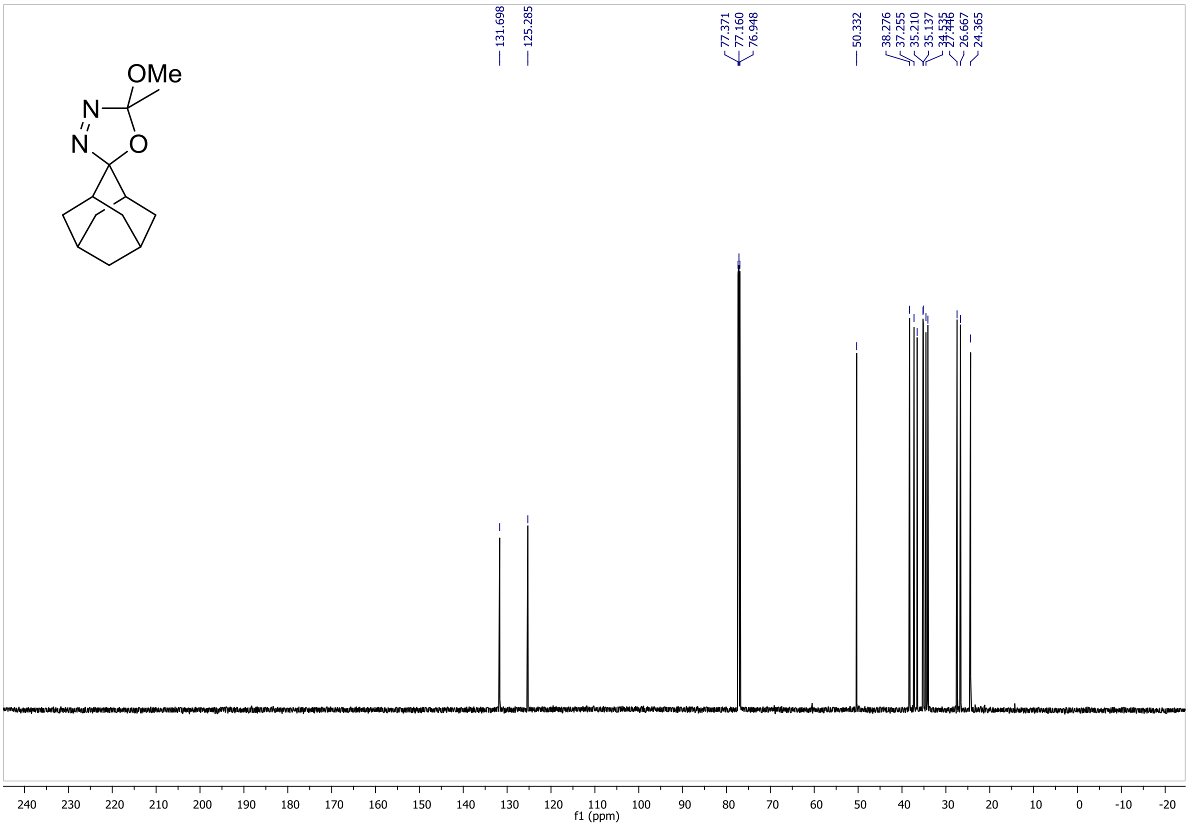

2-cyclopropyl-5-methoxy-2,5-dimethyl-2,5-dihydro-1,3,4-oxadiazole:

^{1}H NMR, 600 MHz, $CDCl_3$:

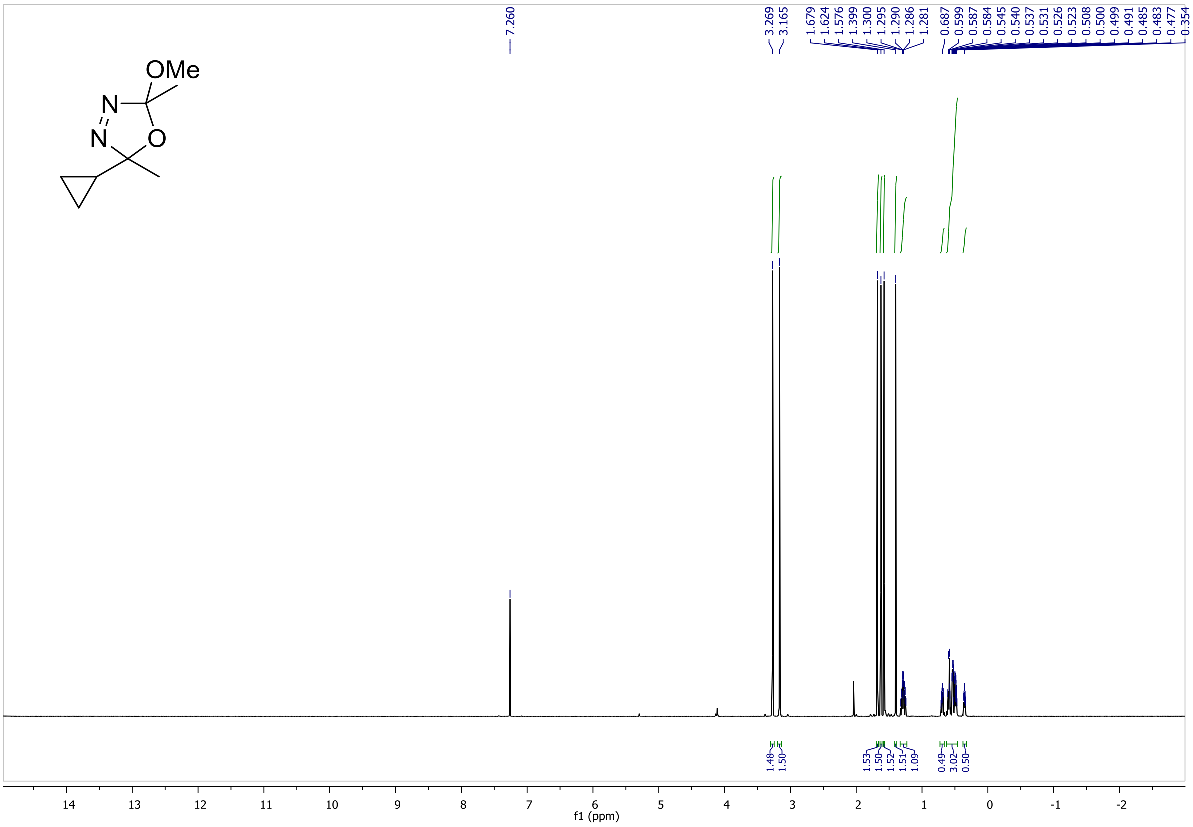

^{13}C NMR, 150 MHz, $CDCl_3$:

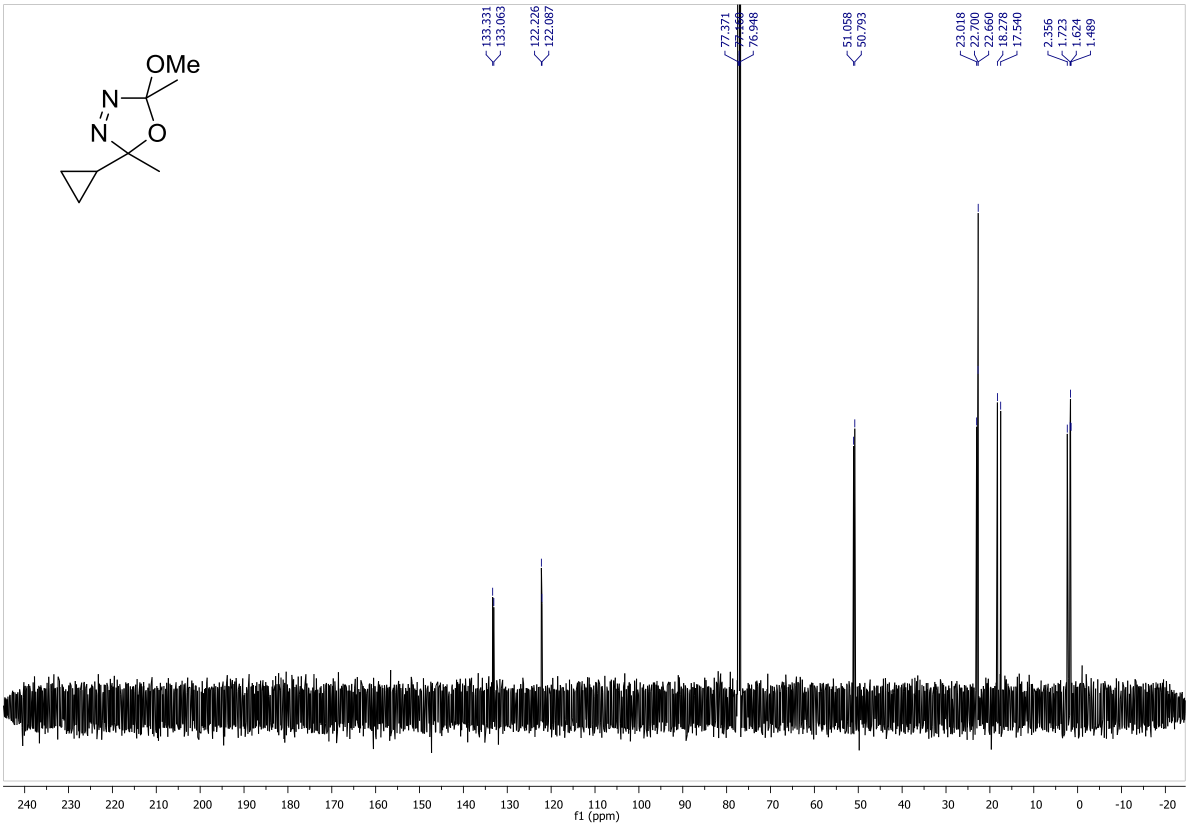

2-(but-3-en-1-yl)-5-methoxy-2,5-dimethyl-2,5-dihydro-1,3,4-oxadiazole:

^{1}H NMR, 600 MHz, $CDCl_3$:

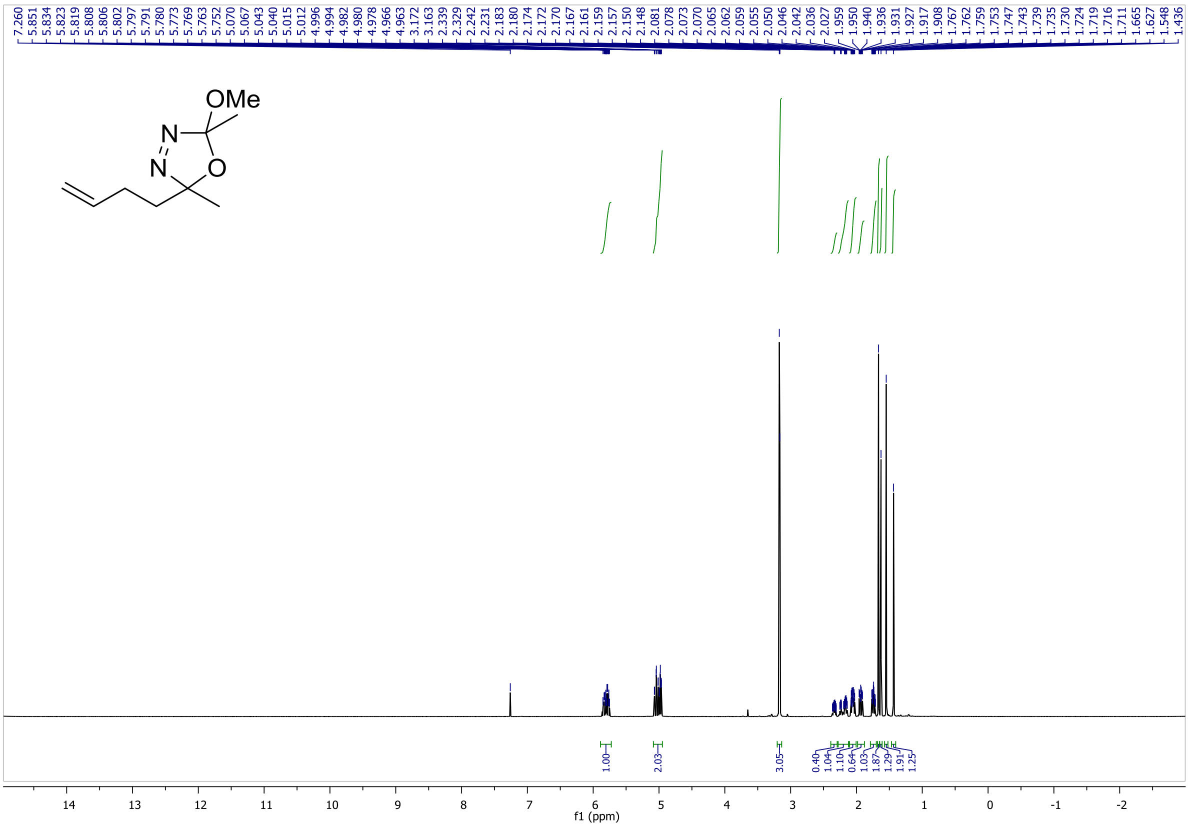

^{13}C NMR, 150 MHz, $CDCl_3$:

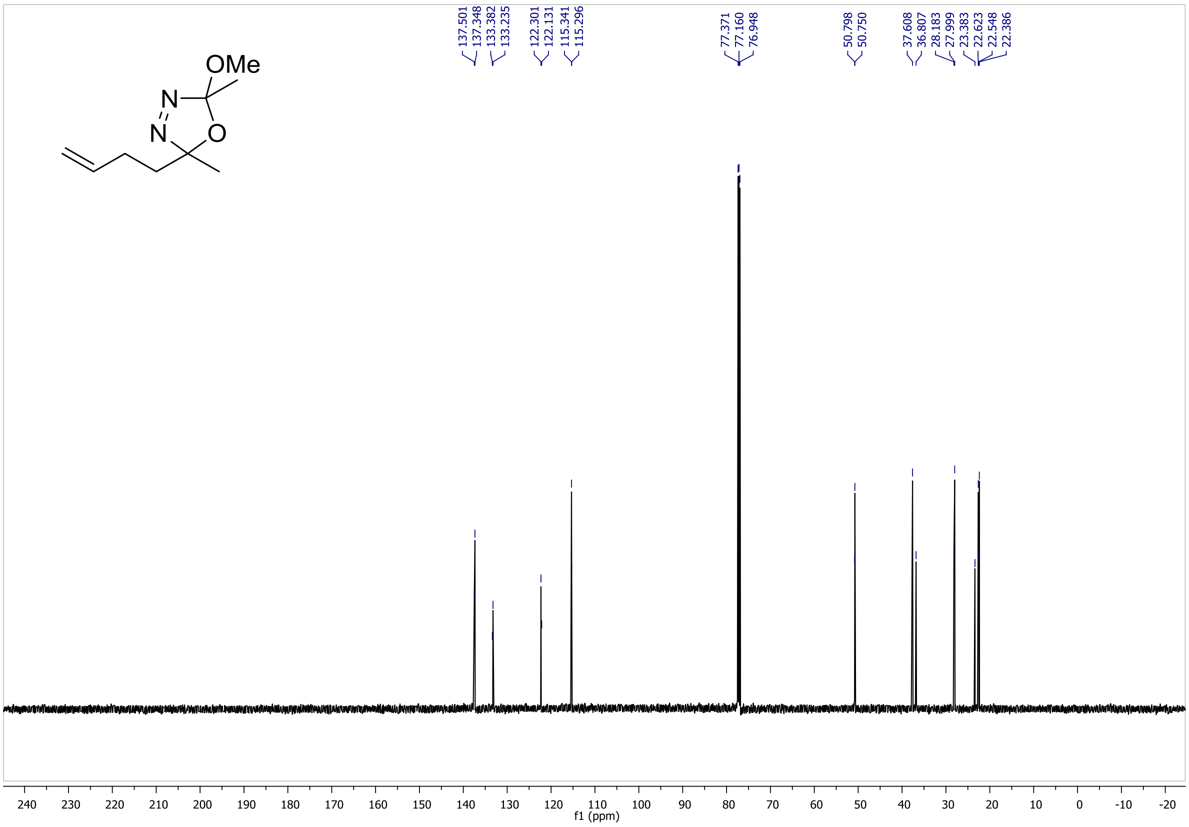

2-(2,2-dimethylpent-4-yn-1-yl)-5-methoxy-2,5-dimethyl-2,5-dihydro-1,3,4-oxadiazole:

^{1}H NMR, 600 MHz, $CDCl_3$:

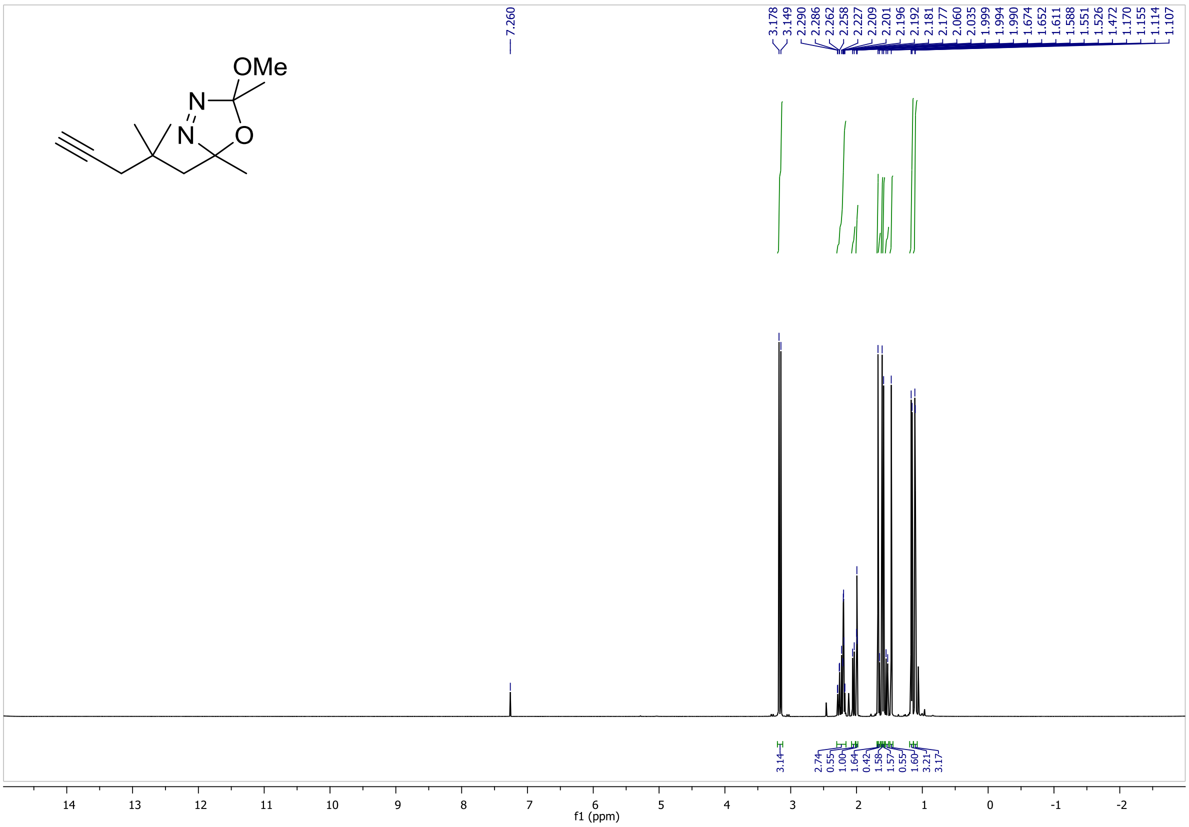

^{13}C NMR, 150 MHz, $CDCl_3$:

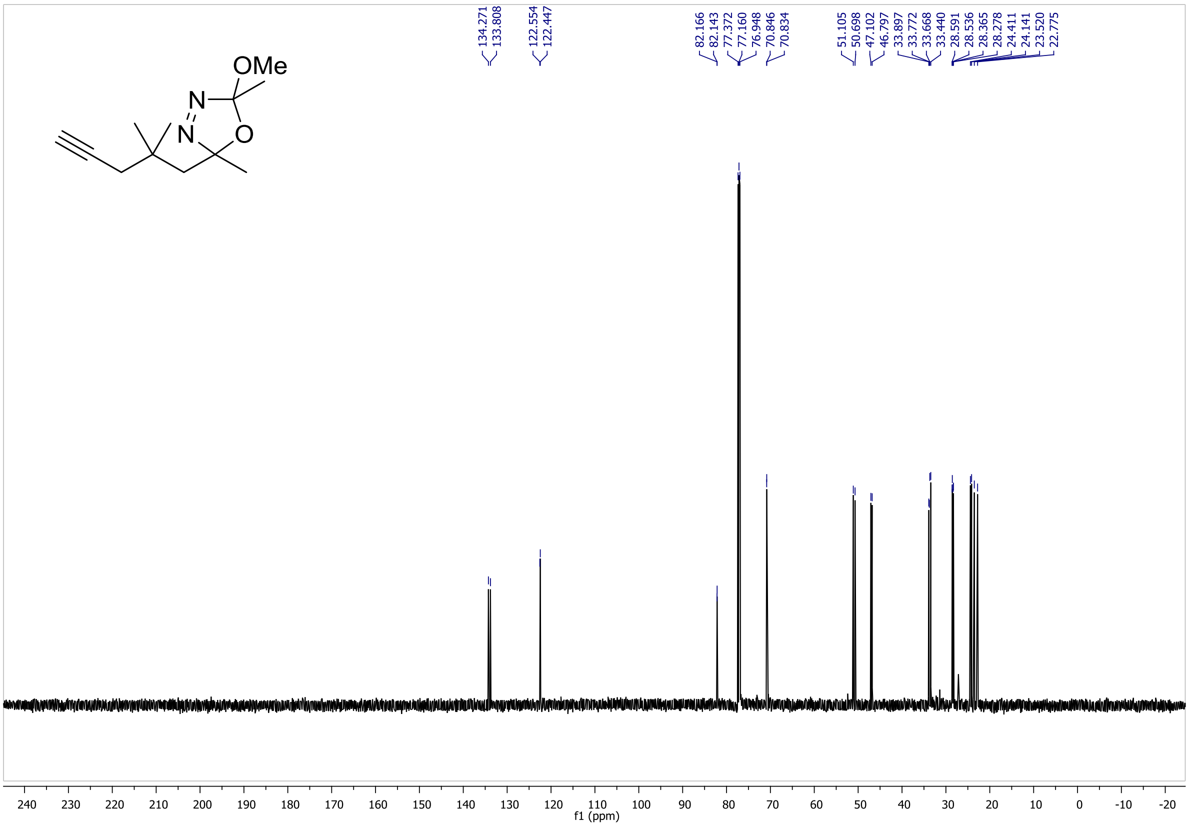

3-methoxy-3-methyl-4,9,12-trioxa-1,2-diazadispiro[4.2.4^8.2^5]tetradec-1-ene:

^{1}H NMR, 600 MHz, $CDCl_3$:

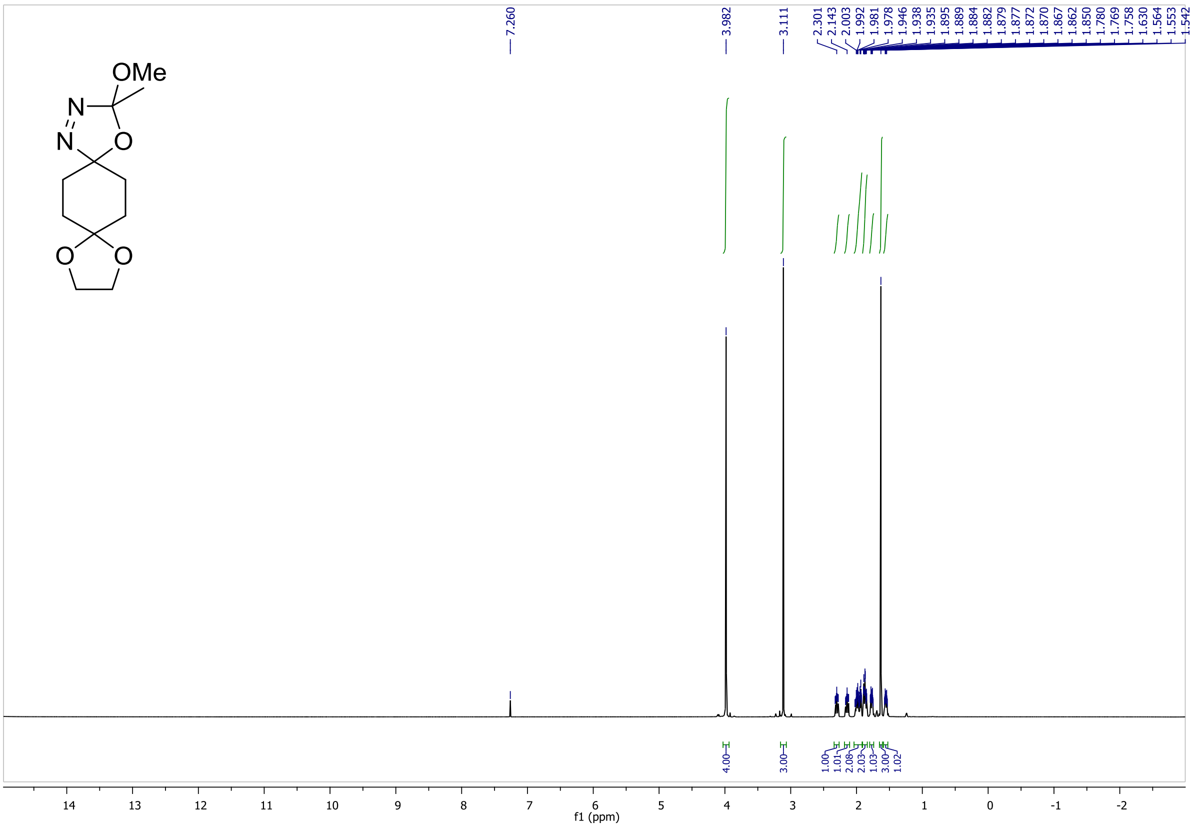

^{13}C NMR, 150 MHz, $CDCl_3$:

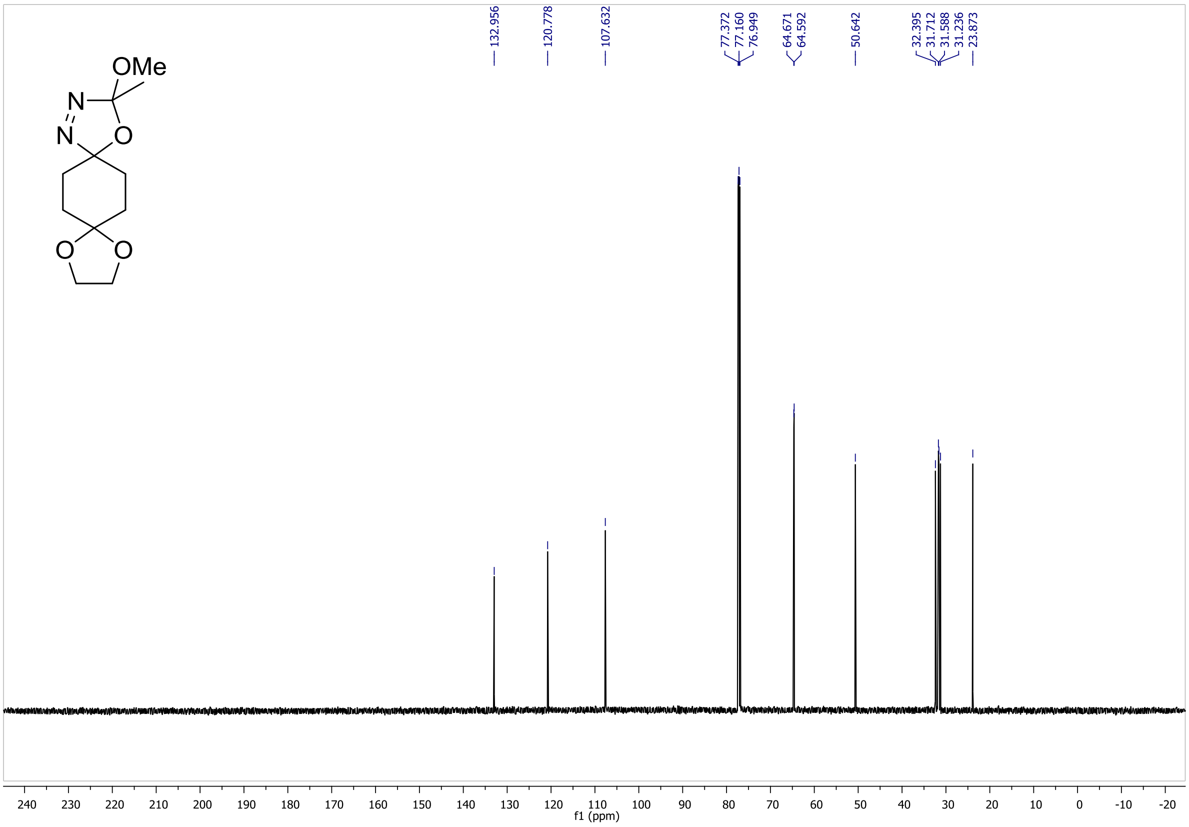

Diethyl ((5-methoxy-2,5-dimethyl-2,5-dihydro-1,3,4-oxadiazol-2-yl)methyl)phosphonate:

^{1}H NMR, 600 MHz, $CDCl_3$:

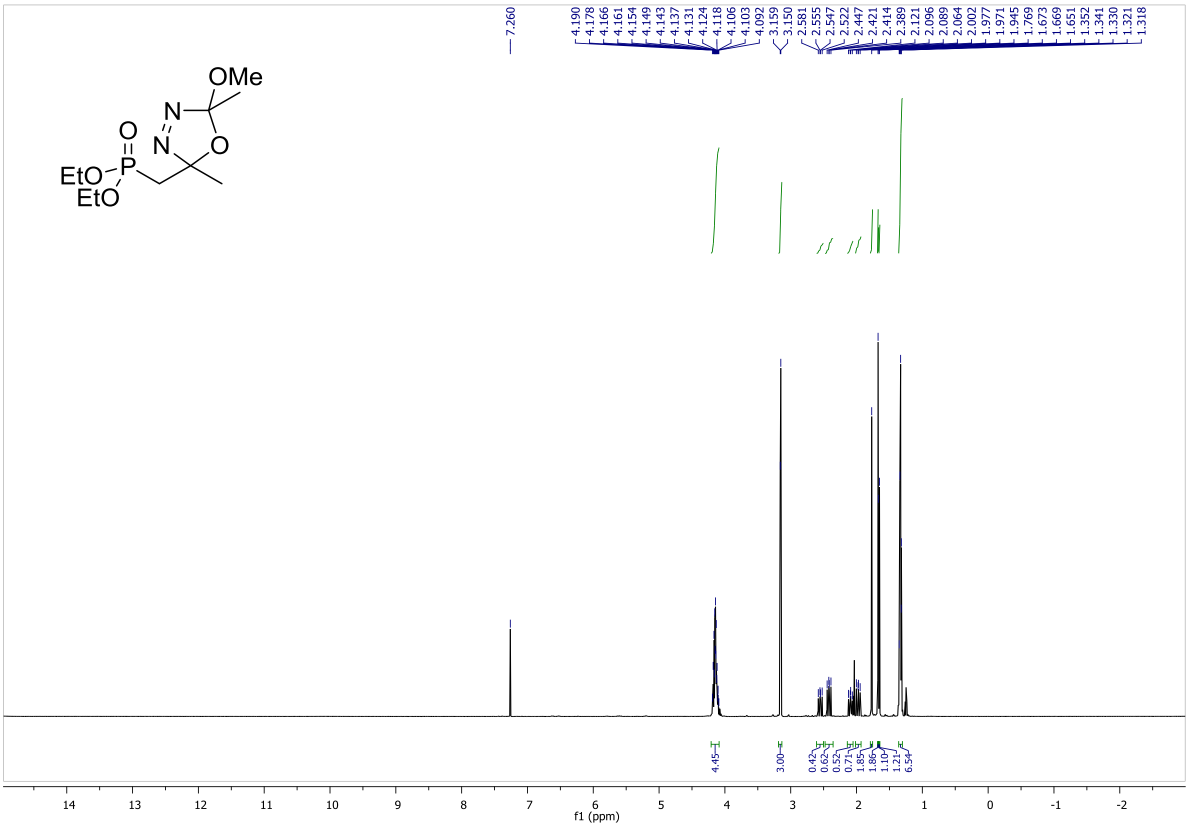

^{13}C NMR, 150 MHz, $CDCl_3$:

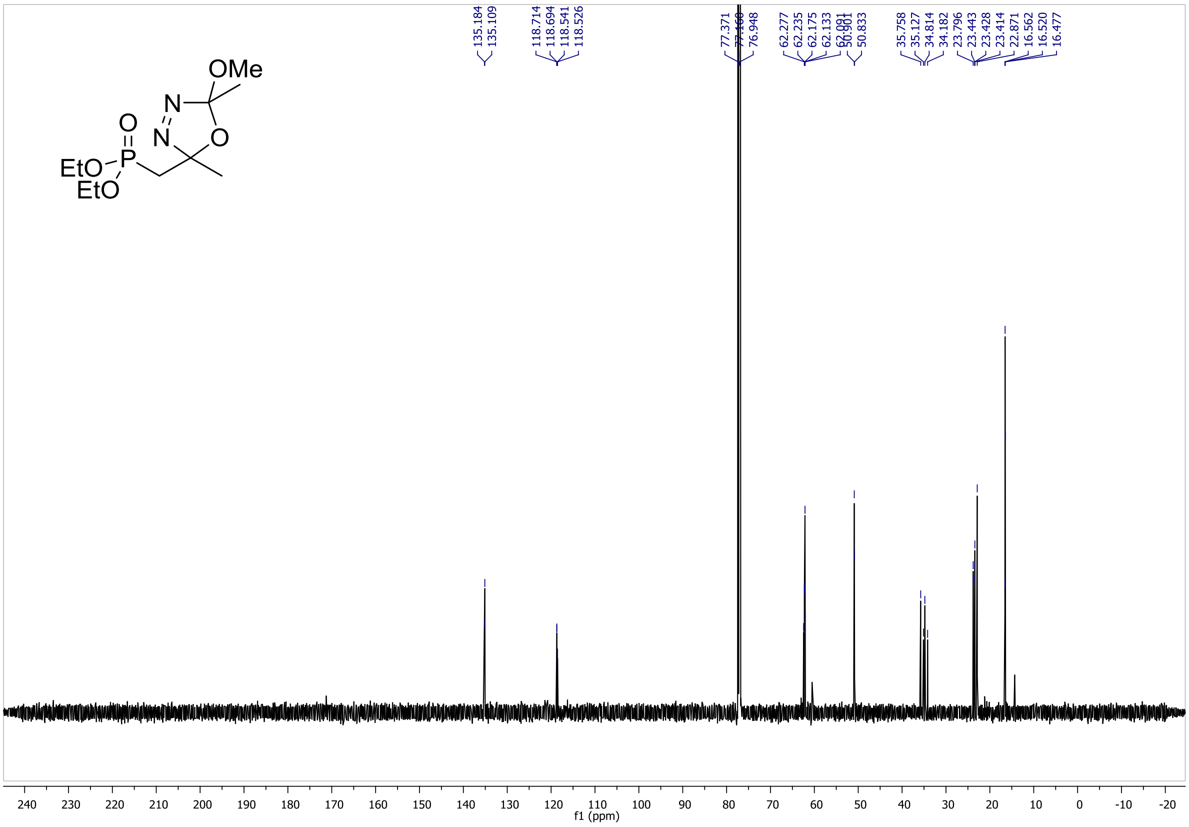

^{31}**P NMR, 245 MHz, CDCl$_3$:**

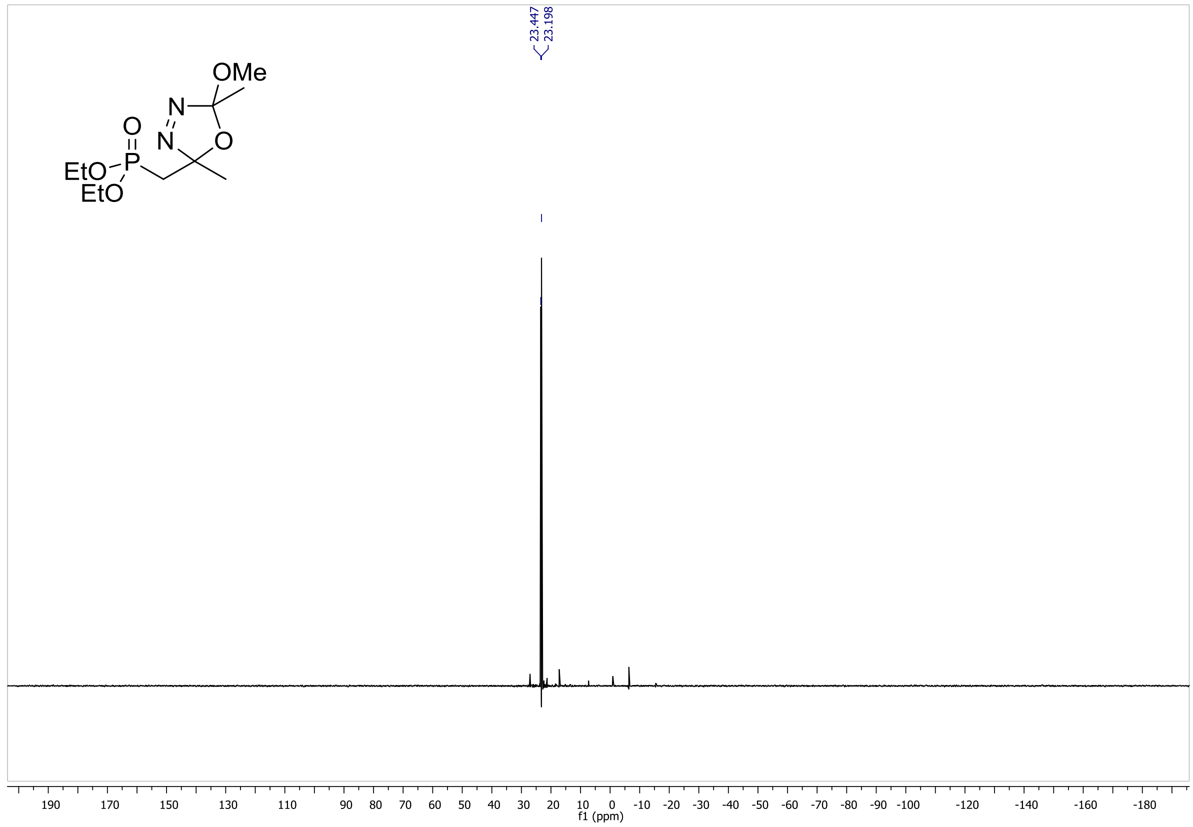

3-methoxy-3-methyl-4-oxa-8-thia-1,2-diazaspiro[4.5]dec-1-ene 8,8-dioxide:

^{1}H NMR, 600 MHz, $CDCl_3$:

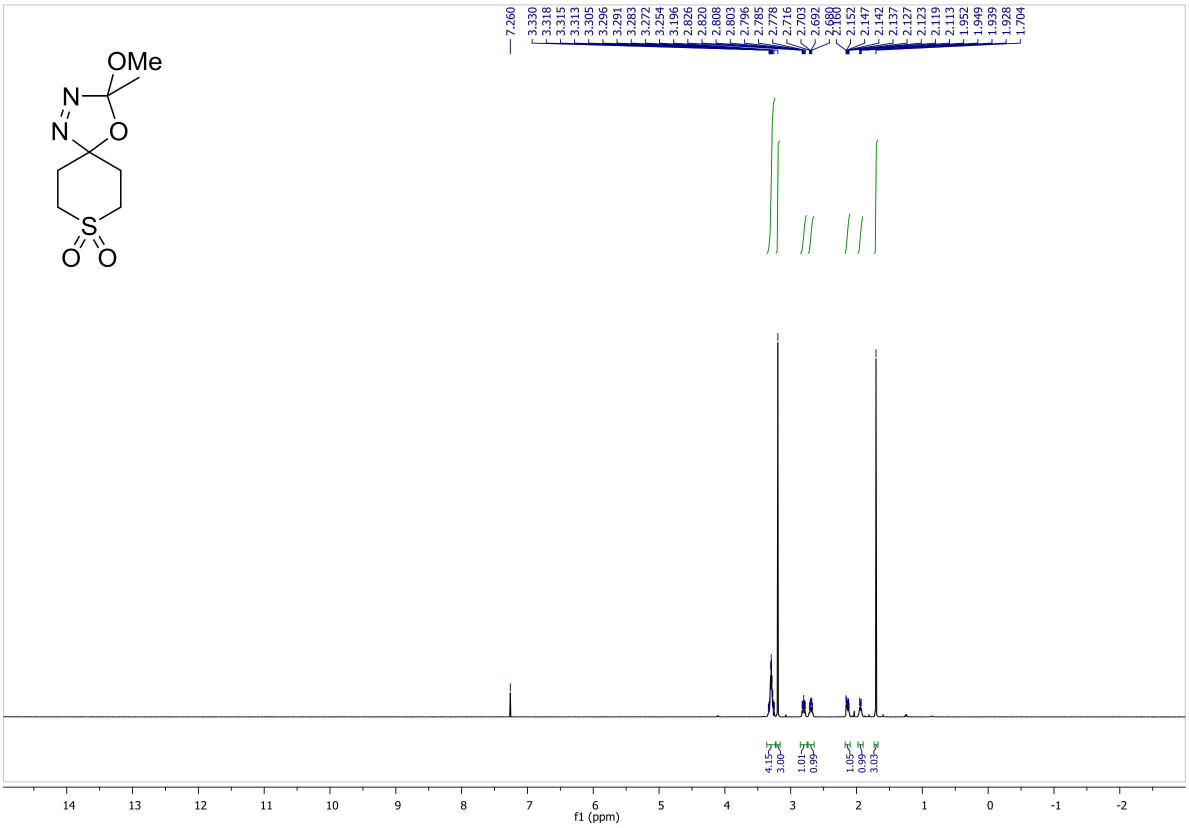

^{13}C NMR, 150 MHz, $CDCl_3$:

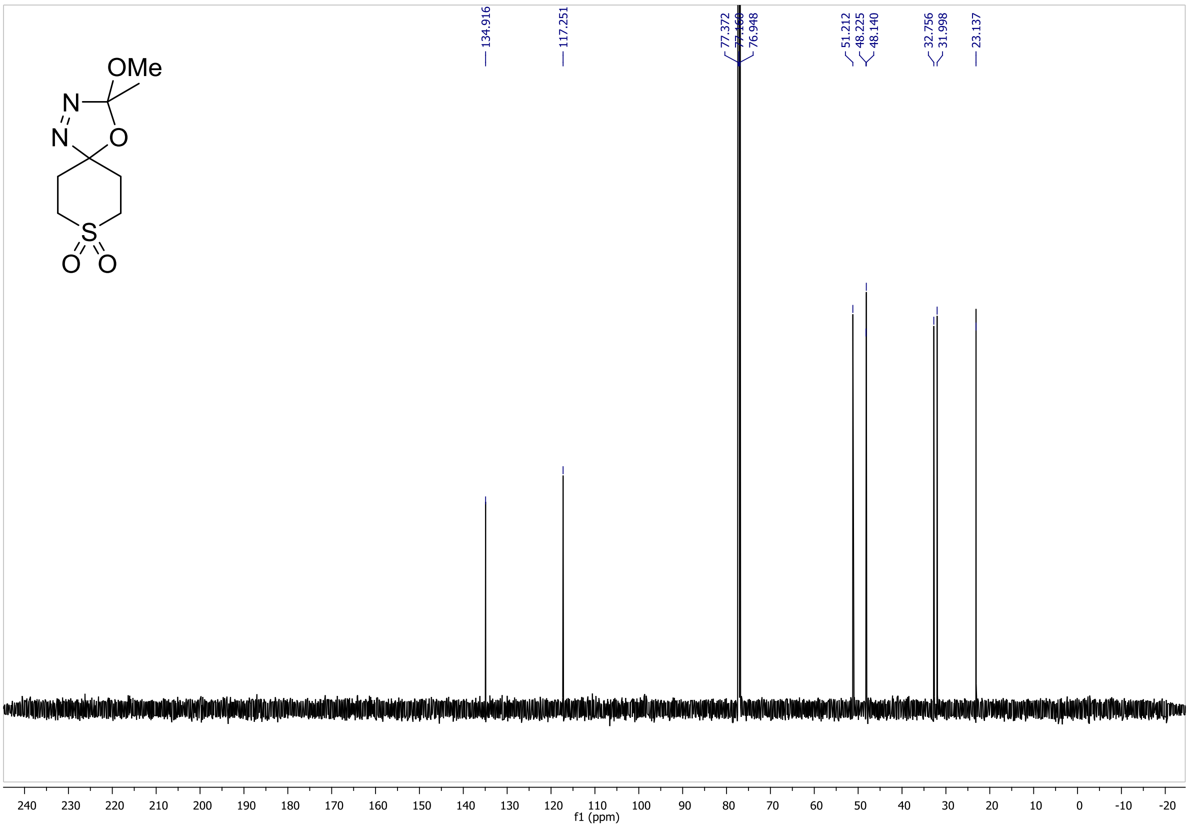

8-(propan-2-ylidene)-1,4-dioxaspiro[4.5]decane:

^{1}H NMR, 600 MHz, $CDCl_3$:

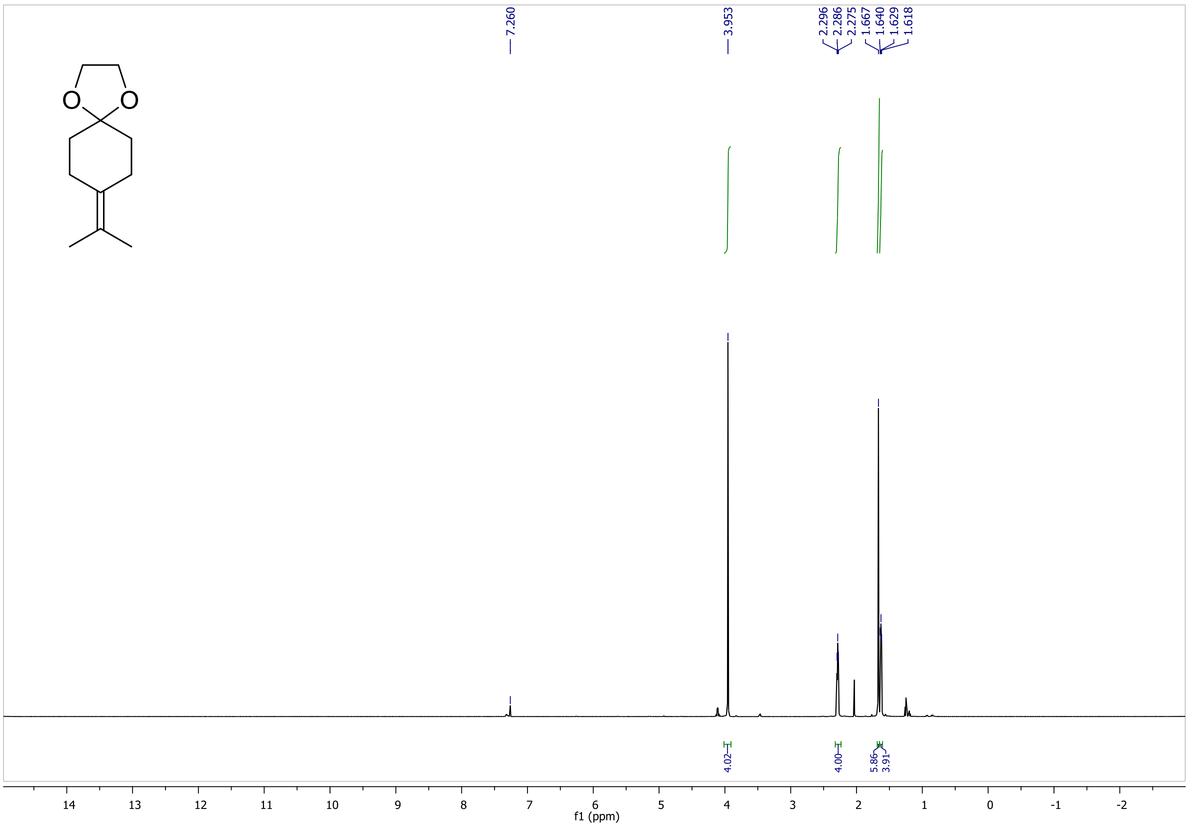

^{13}C NMR, 150 MHz, $CDCl_3$:

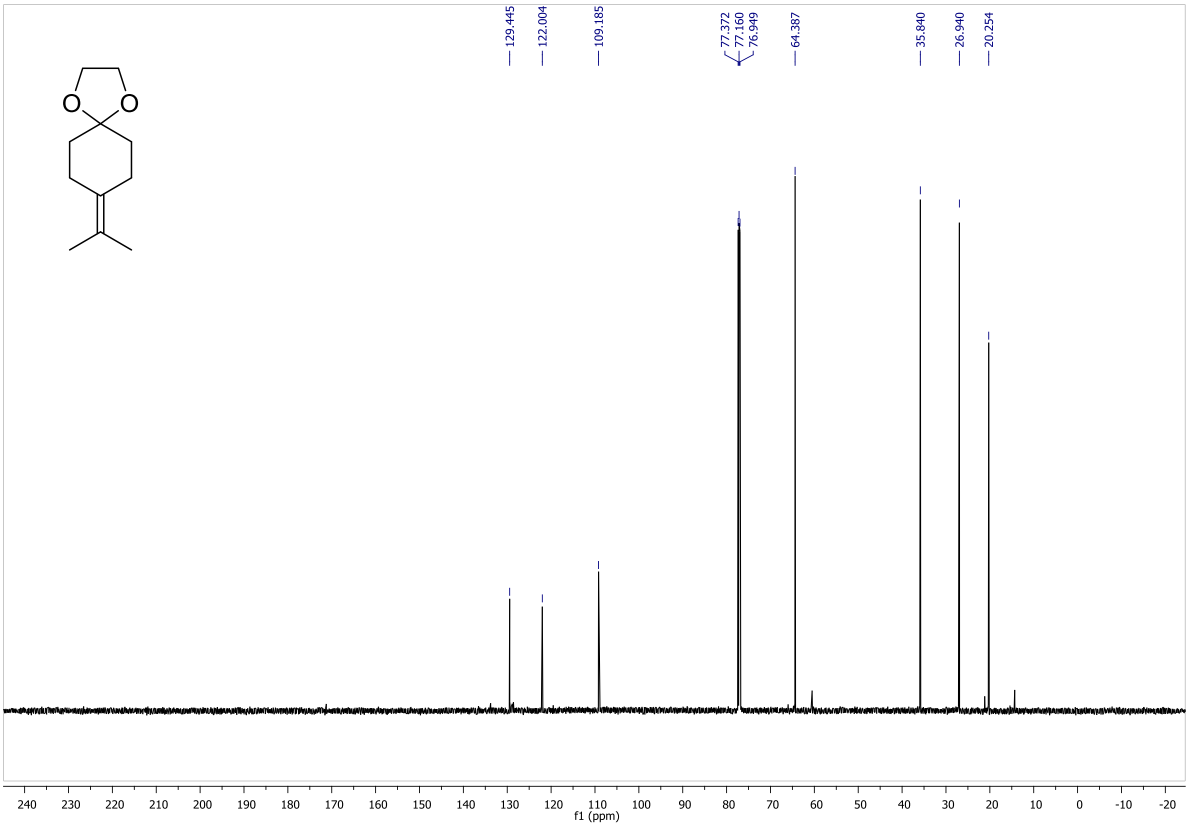

4-(propan-2-ylidene)cyclohexan-1-one:

^{1}H NMR, 600 MHz, $CDCl_3$:

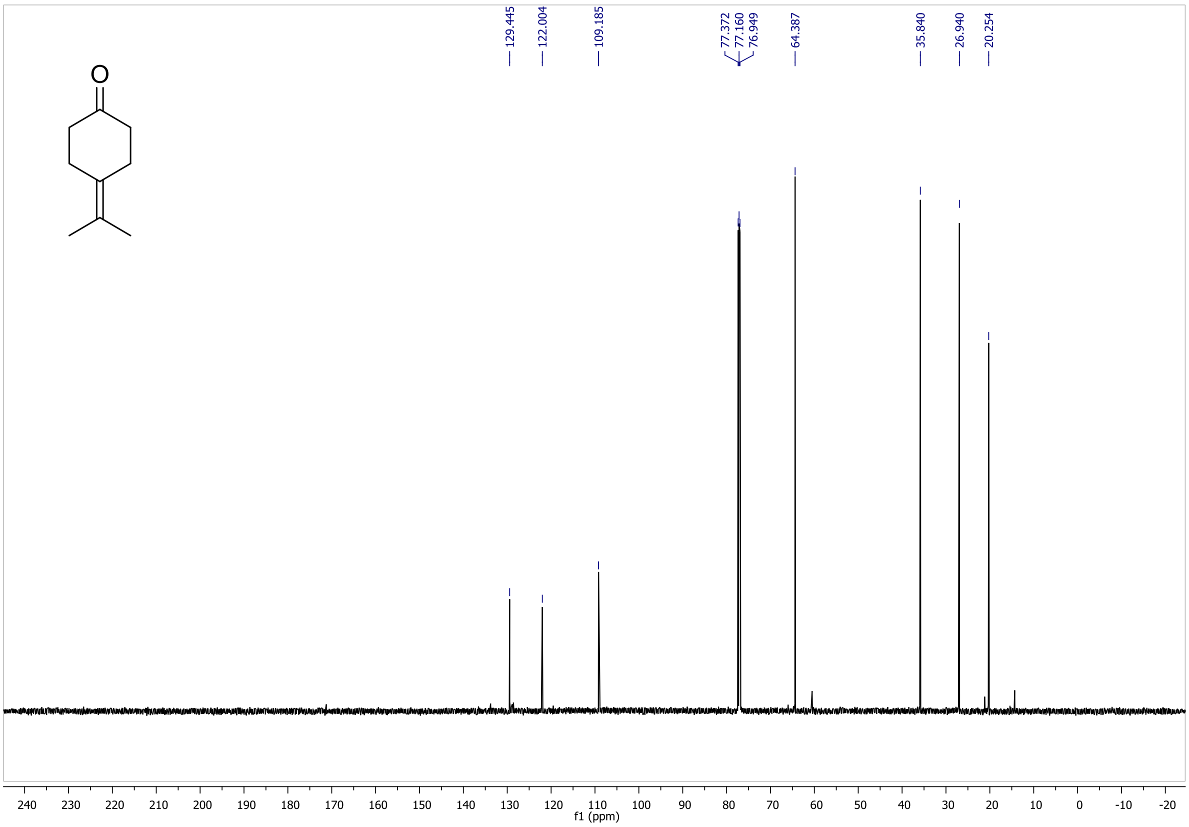

^{13}C NMR, 150 MHz, $CDCl_3$:

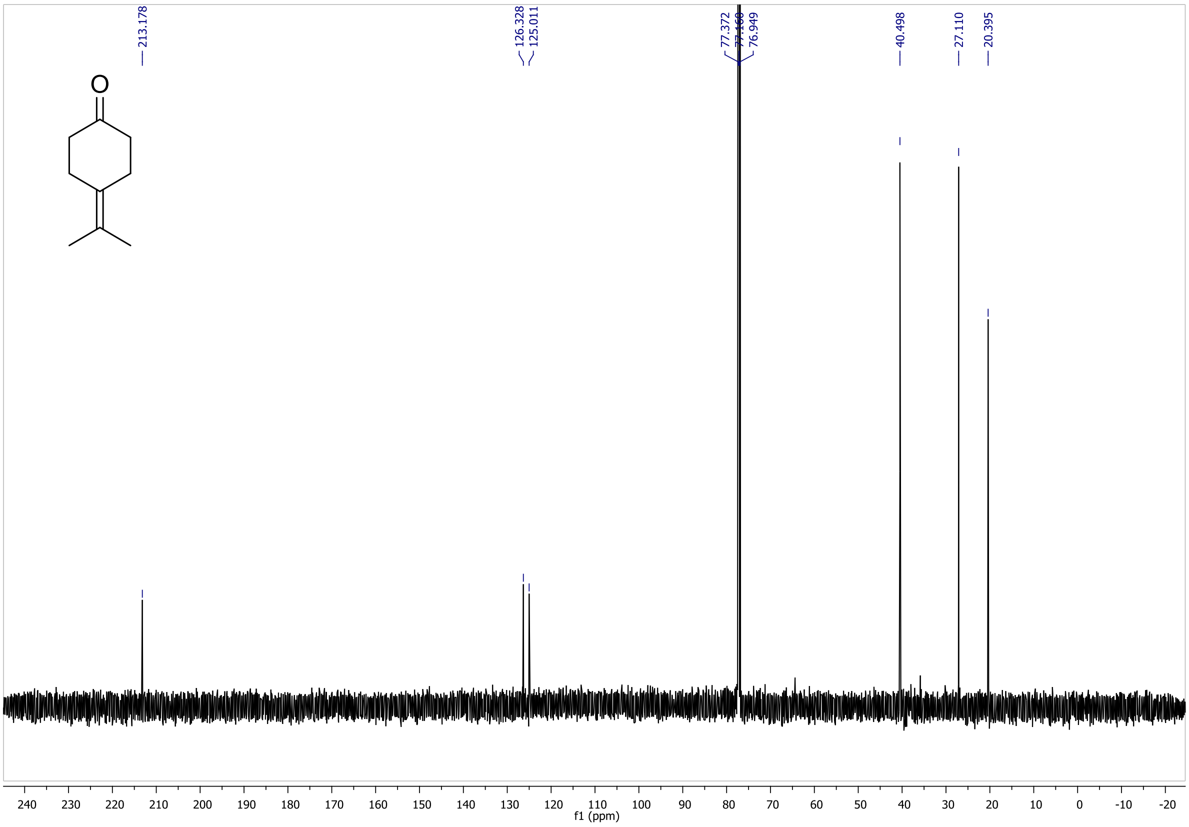

2,2-dimethyl-1-oxaspiro[2.5]octan-6-one:

^{1}H NMR, 600 MHz, $CDCl_3$:

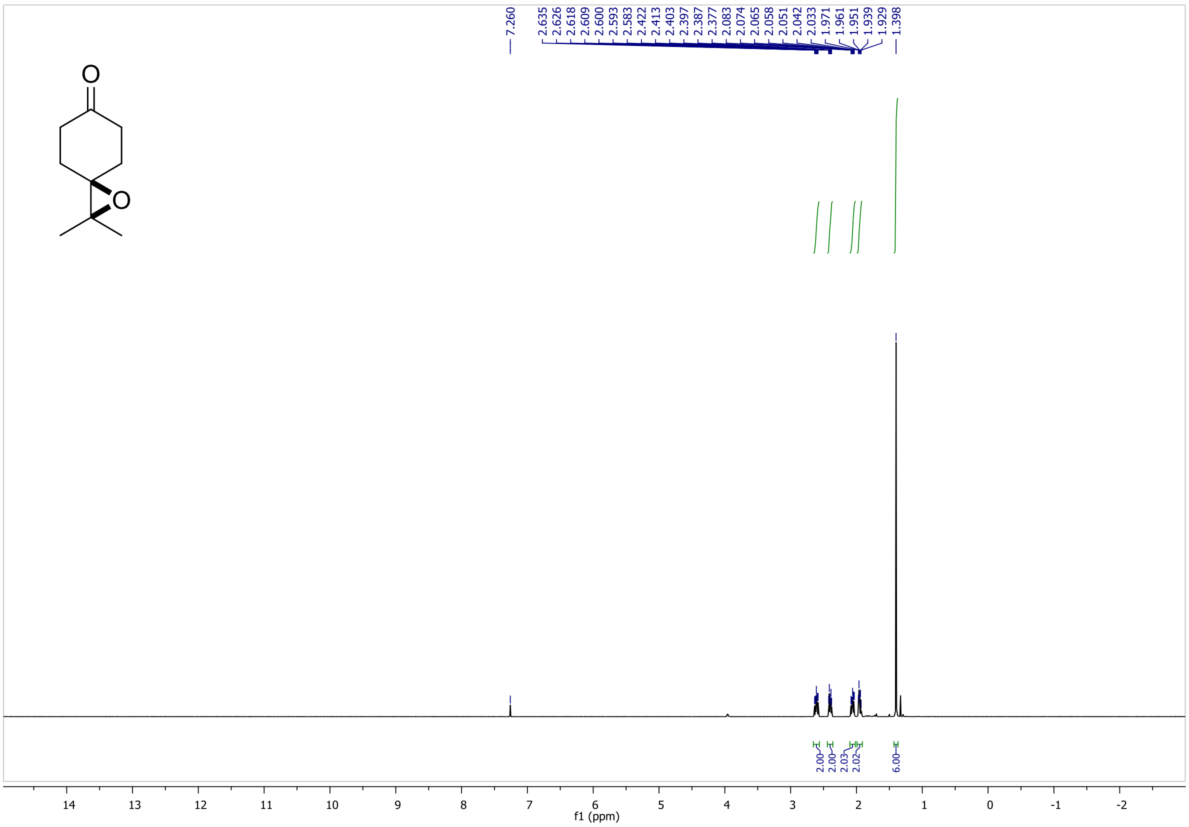

^{13}C NMR, 150 MHz, $CDCl_3$:

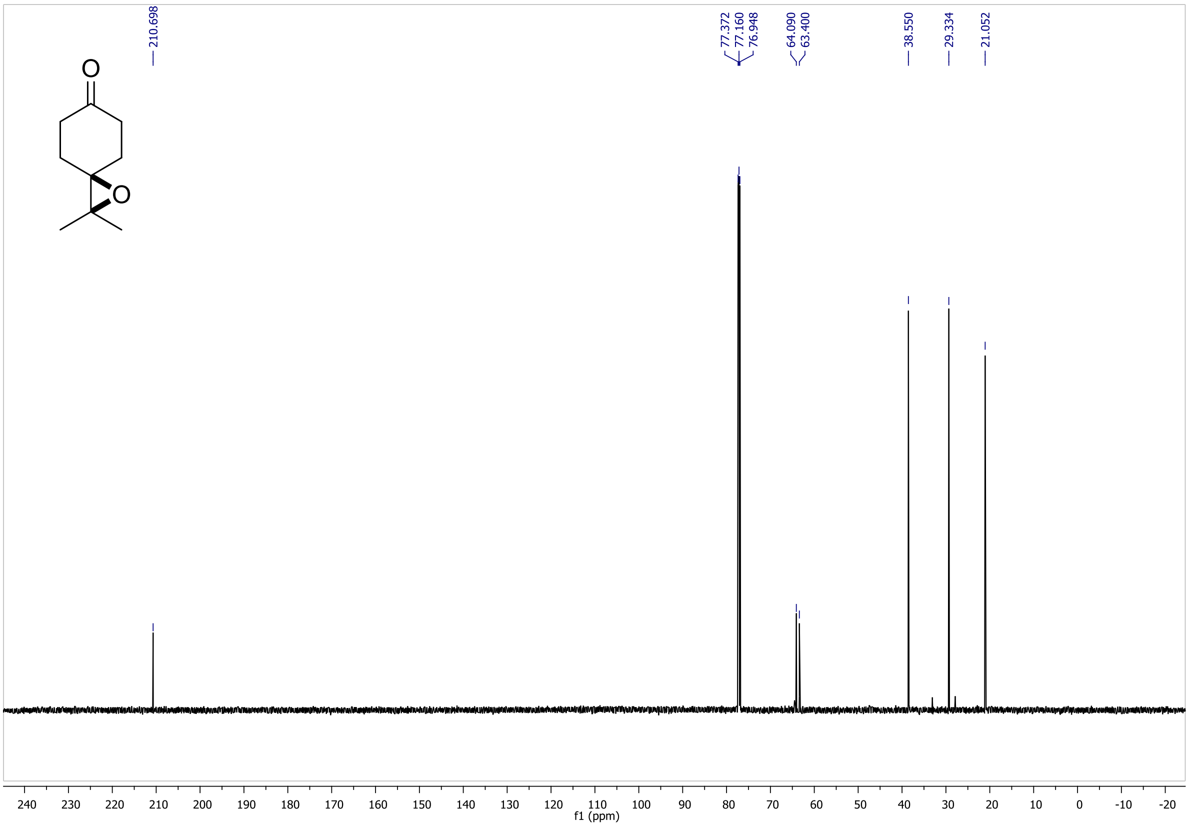

9-methoxy-2,2,9-trimethyl-1,10-dioxa-7,8-diazadispiro[2.2.4^{6}.2^{3}]dodec-7-ene:

^{1}H NMR, 600 MHz, $CDCl_3$:

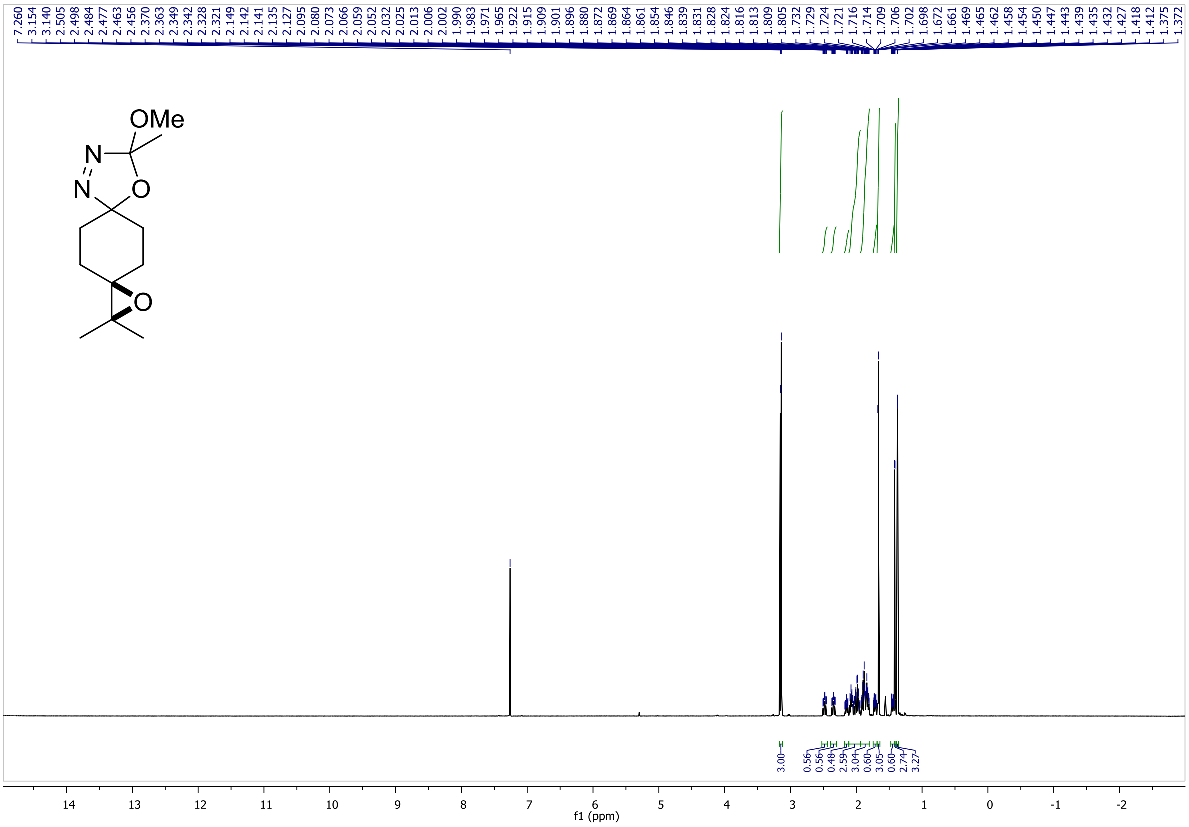

^{13}C NMR, 150 MHz, $CDCl_3$:

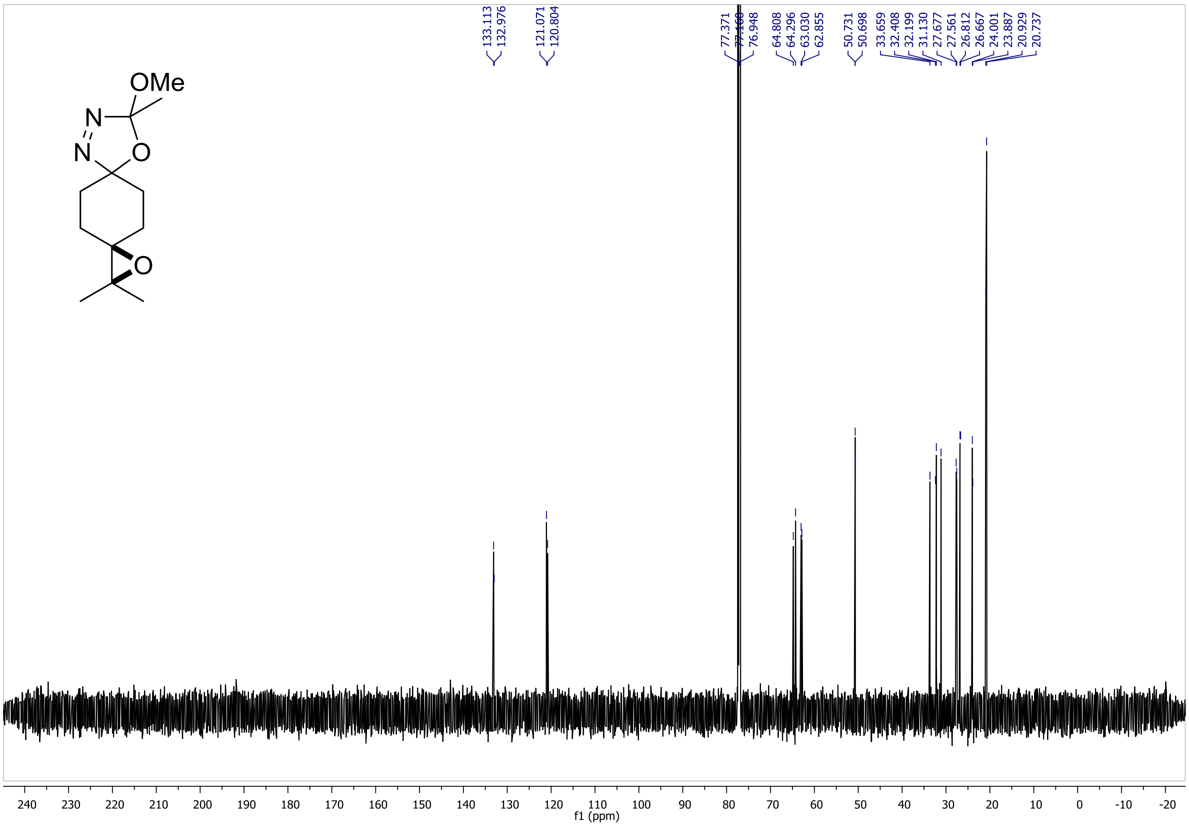

3-(5-methoxy-2,5-dimethyl-2,5-dihydro-1,3,4-oxadiazol-2-yl)propan-1-ol:

^{1}H NMR, 600 MHz, $CDCl_3$:

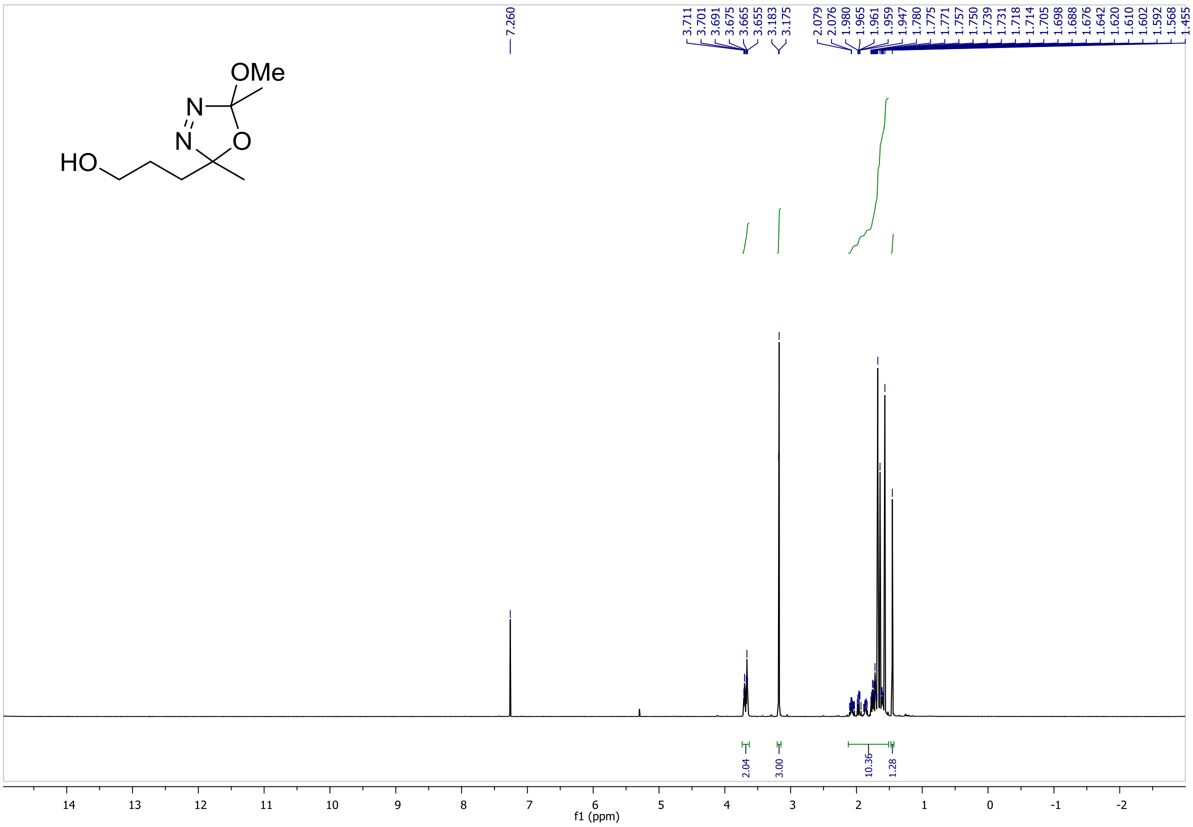

^{13}C NMR, 150 MHz, $CDCl_3$:

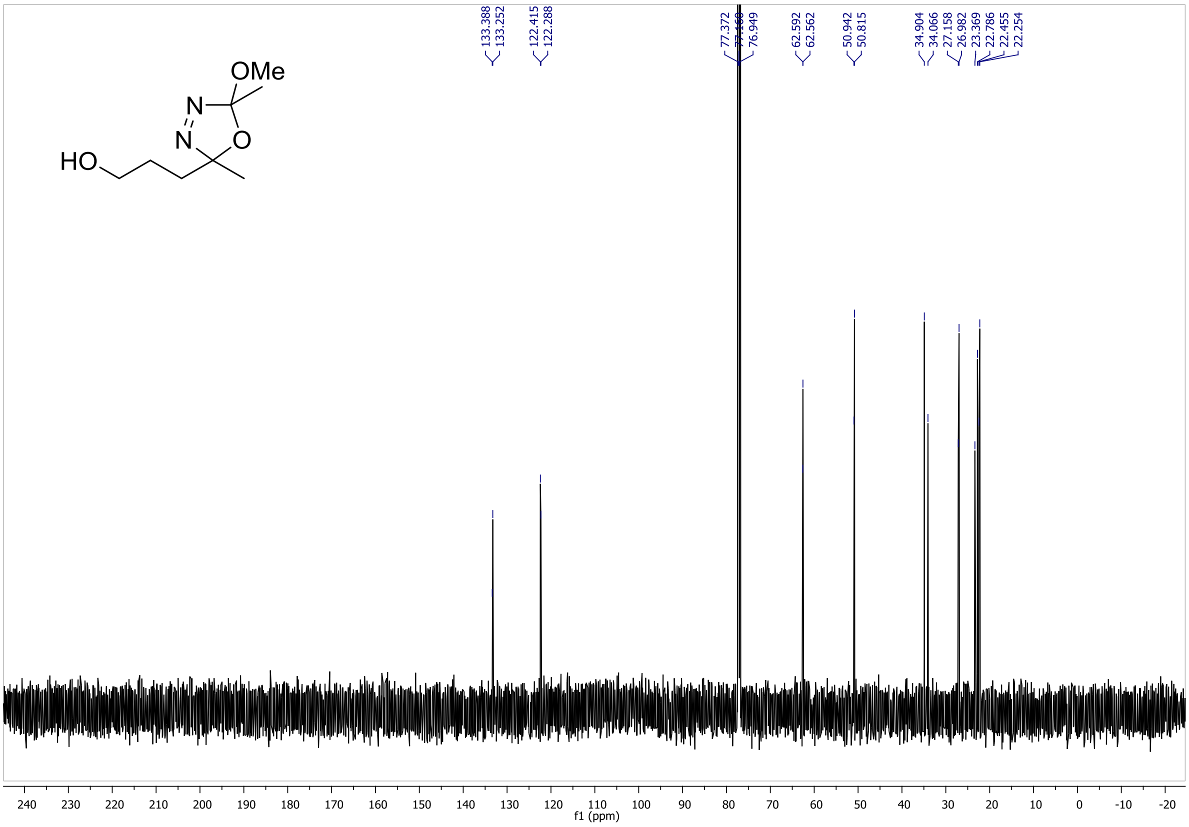

2-(3-bromopropyl)-5-methoxy-2,5-dimethyl-2,5-dihydro-1,3,4-oxadiazole:

^{1}H NMR, 600 MHz, $CDCl_3$:

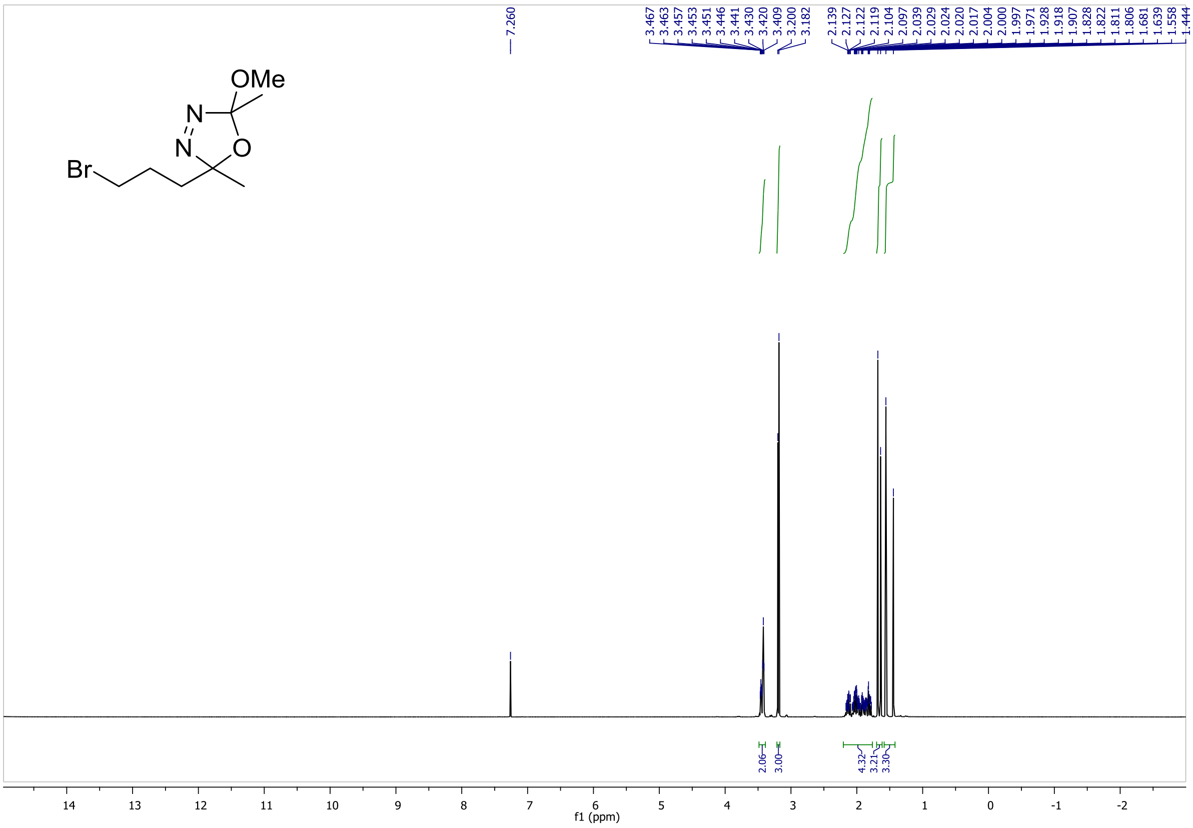

^{13}C NMR, 150 MHz, $CDCl_3$:

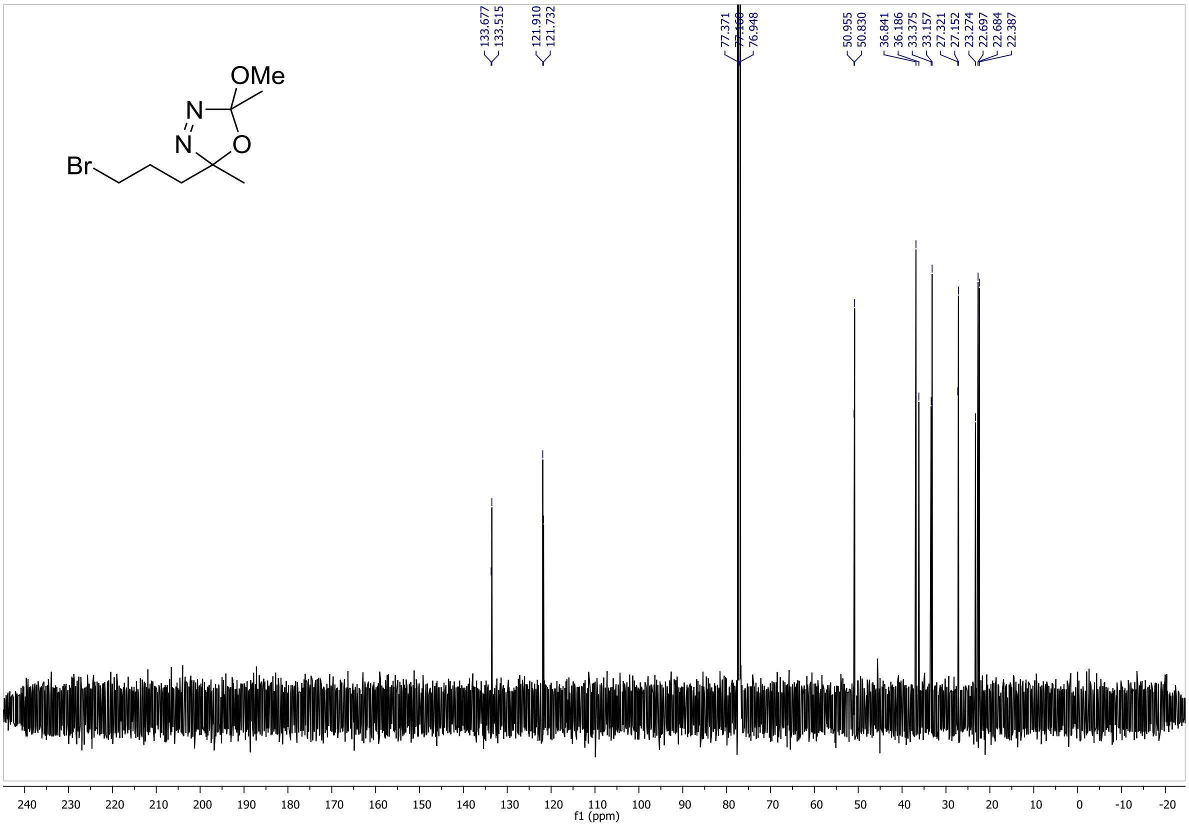

2-(furan-2-ylmethyl)-5-methoxy-2,5-dimethyl-2,5-dihydro-1,3,4-oxadiazole:

^{1}H NMR, 600 MHz, $CDCl_3$:

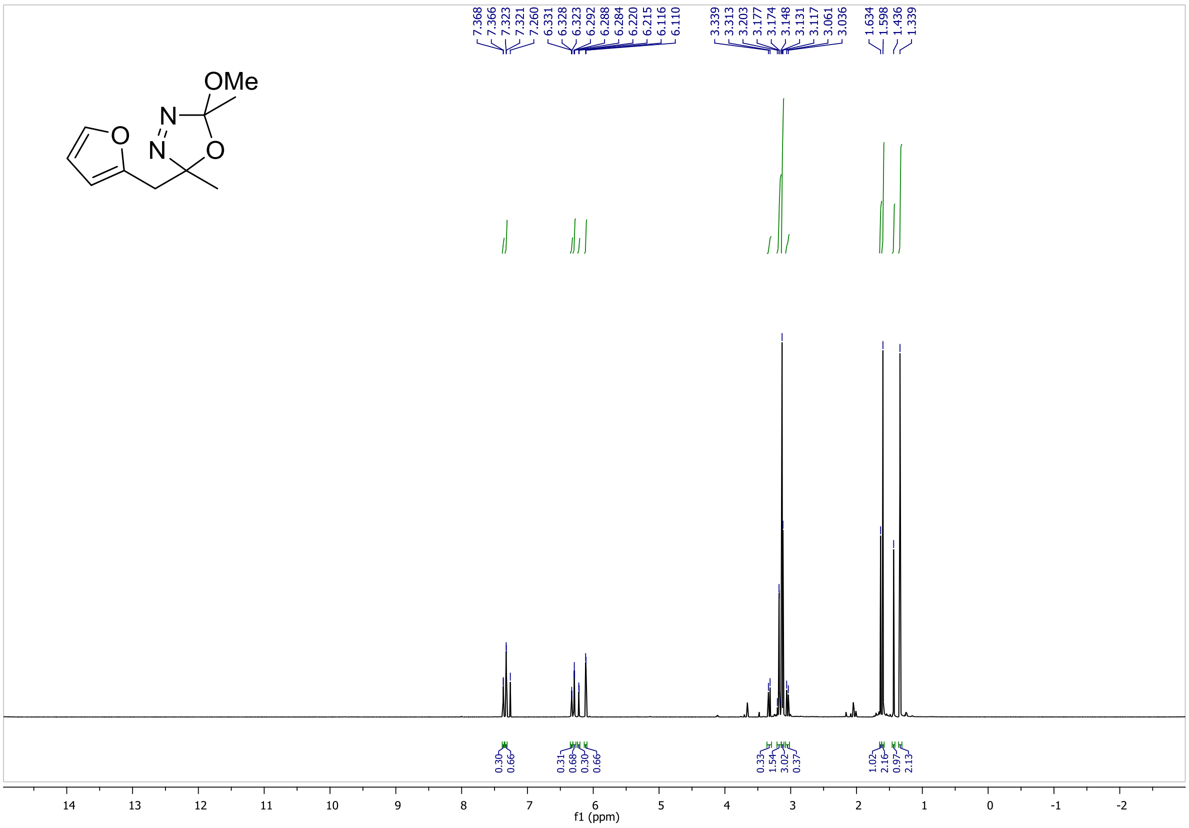

^{13}C NMR, 150 MHz, $CDCl_3$:

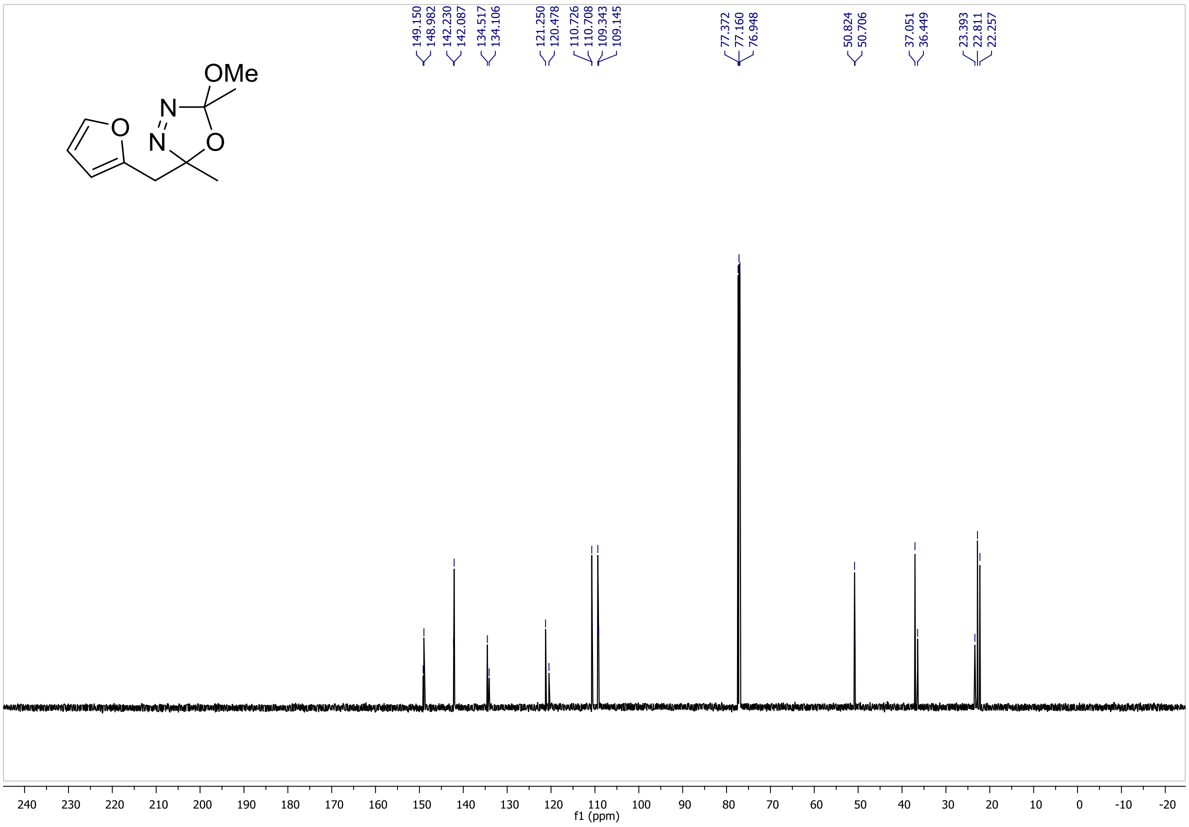

3-methoxy-3-methyl-8-(pyrimidin-2-yl)-4-oxa-1,2,8-triazaspiro[4.5]dec-1-ene:

^{1}H NMR, 600 MHz, $CDCl_3$:

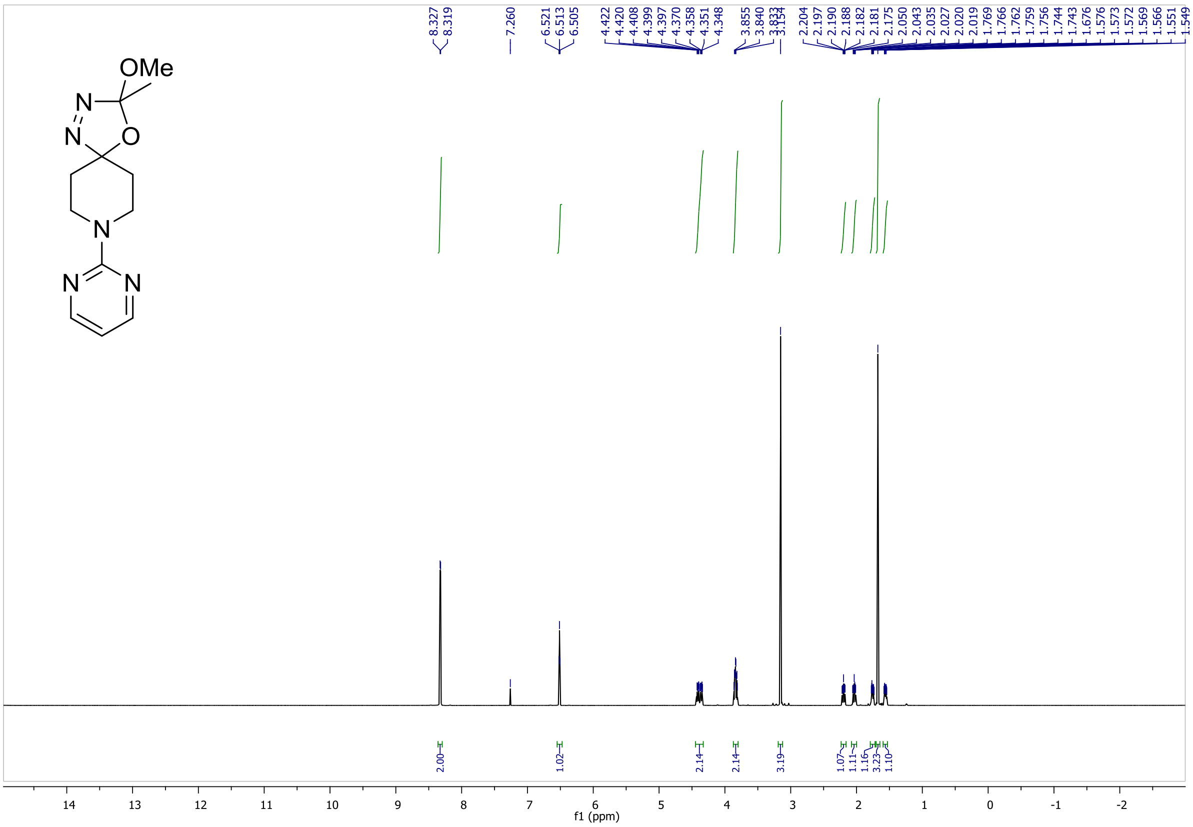

^{13}C NMR, 150 MHz, $CDCl_3$:

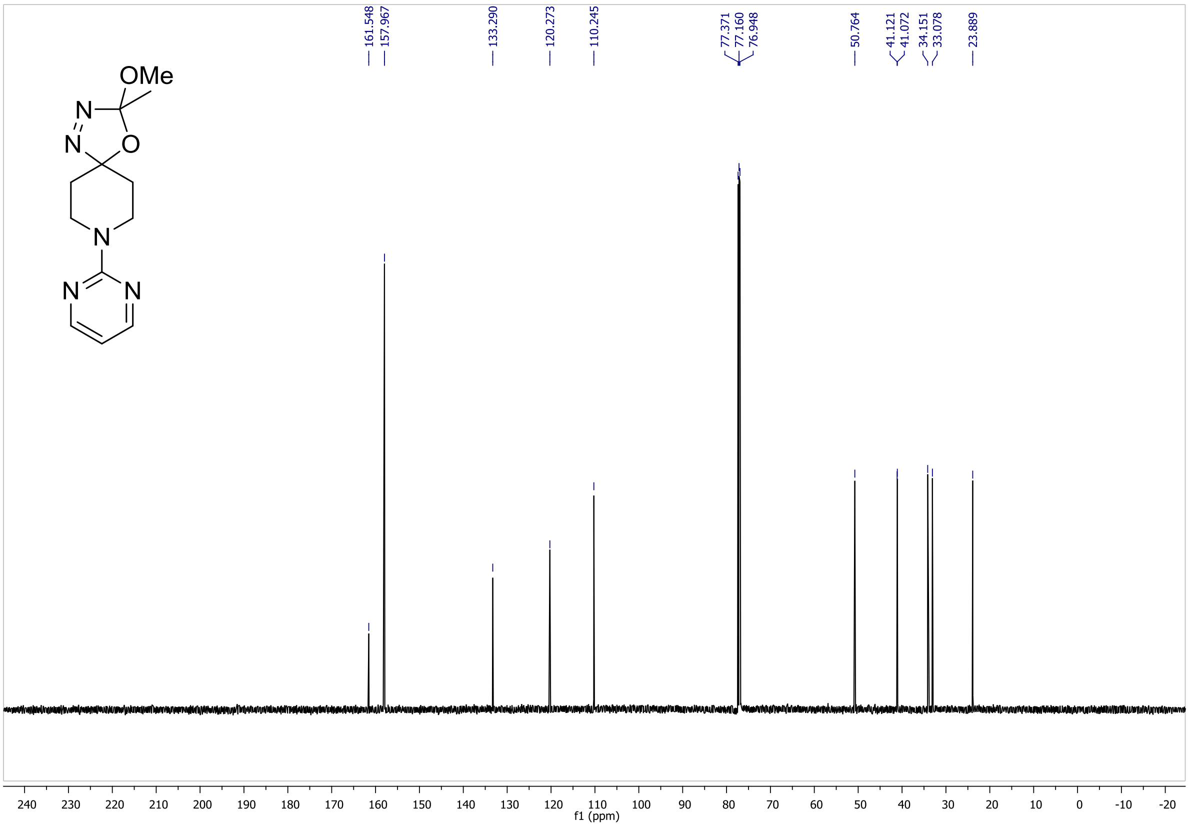

8.2. NMR spectra for protodeboronative coupling products

4-(4-chlorophenyl)tetrahydro-2*H*-pyran (3a):

^{1}H NMR, 600 MHz, $CDCl_3$:

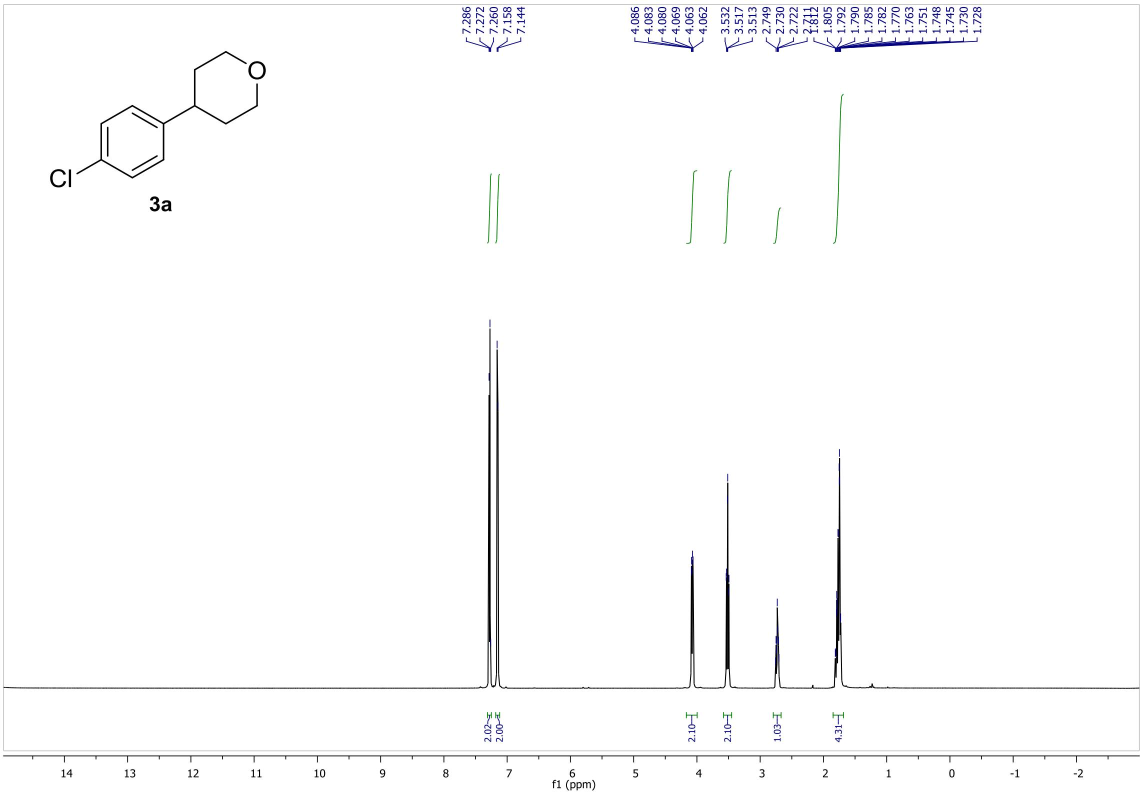

^{13}C NMR, 150 MHz, $CDCl_3$:

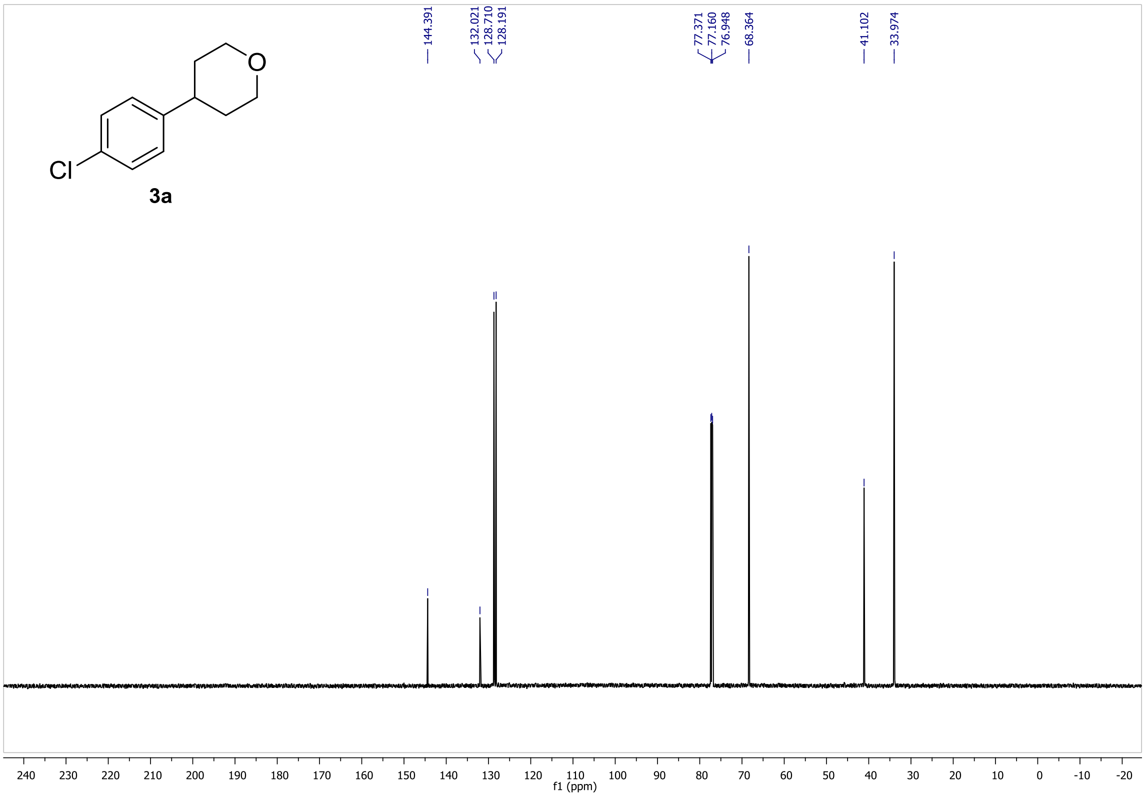

3-(4-chlorophenyl)tetrahydrofuran (3b):

^{1}H NMR, 600 MHz, $CDCl_3$:

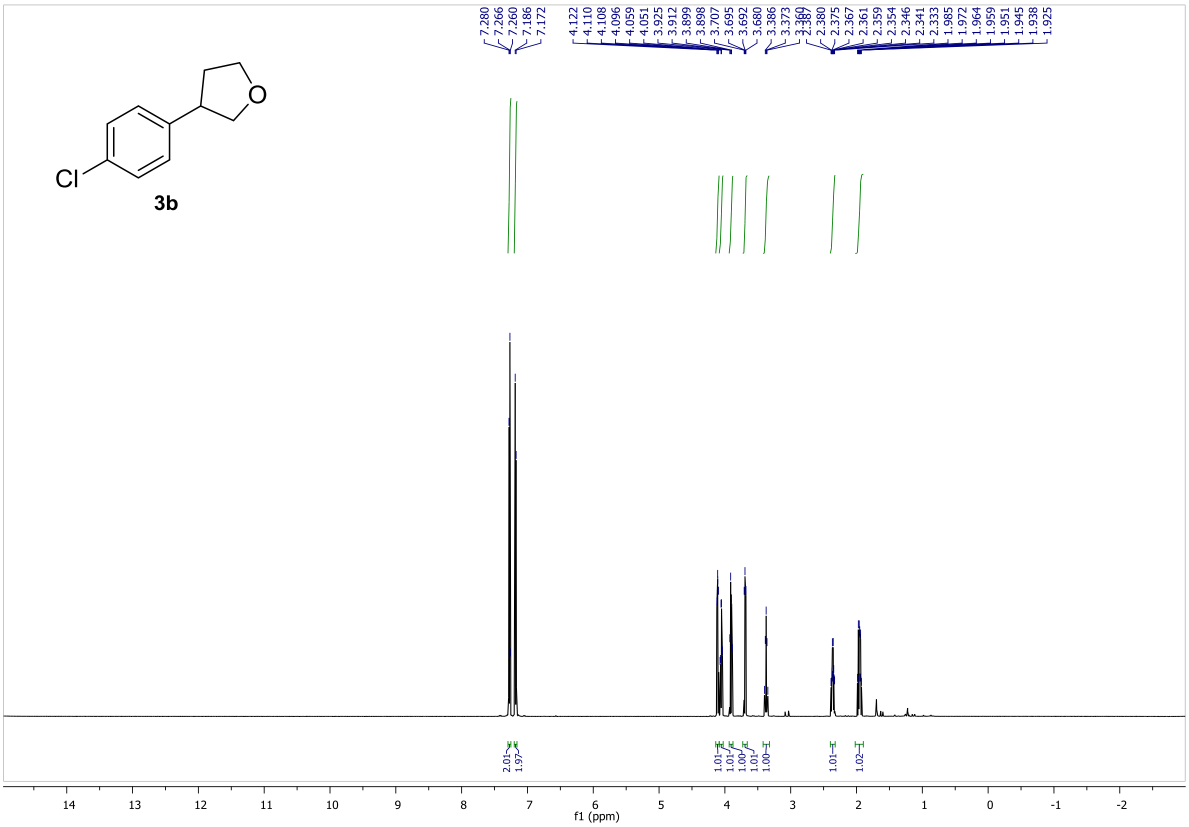

^{13}C NMR, 150 MHz, $CDCl_3$:

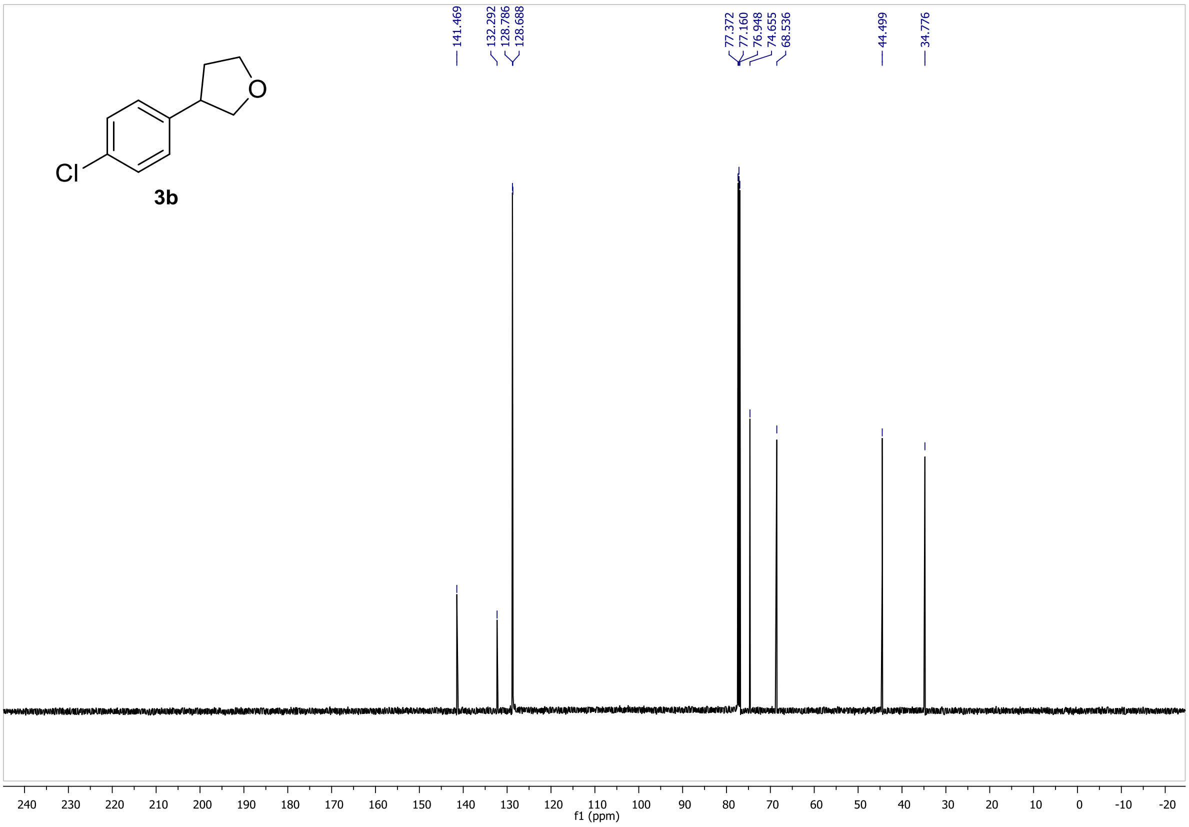

4-(4-chlorophenyl)tetrahydro-2*H*-thiopyran (3c):

^{1}H NMR, 600 MHz, $CDCl_3$:

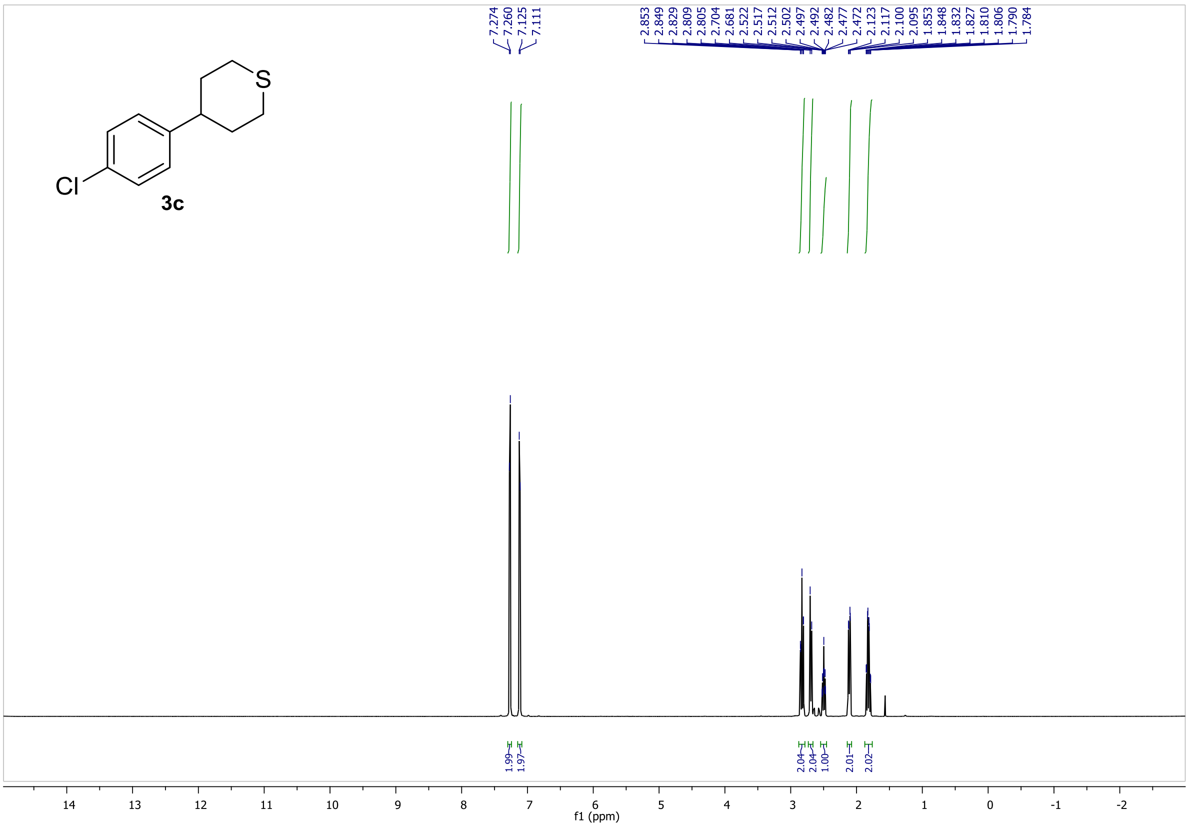

^{13}C NMR, 150 MHz, $CDCl_3$:

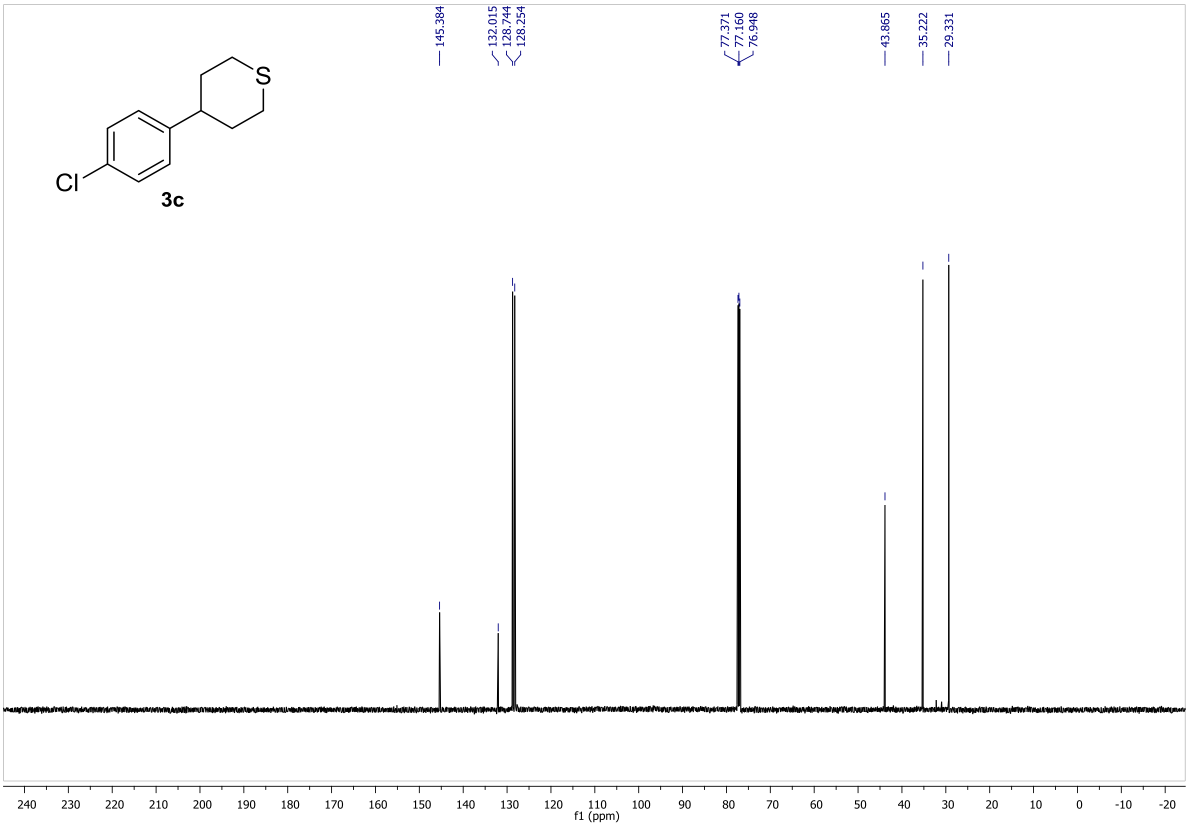

3-(4-chlorophenyl)tetrahydrothiophene (3d):

^{1}H NMR, 600 MHz, $CDCl_3$:

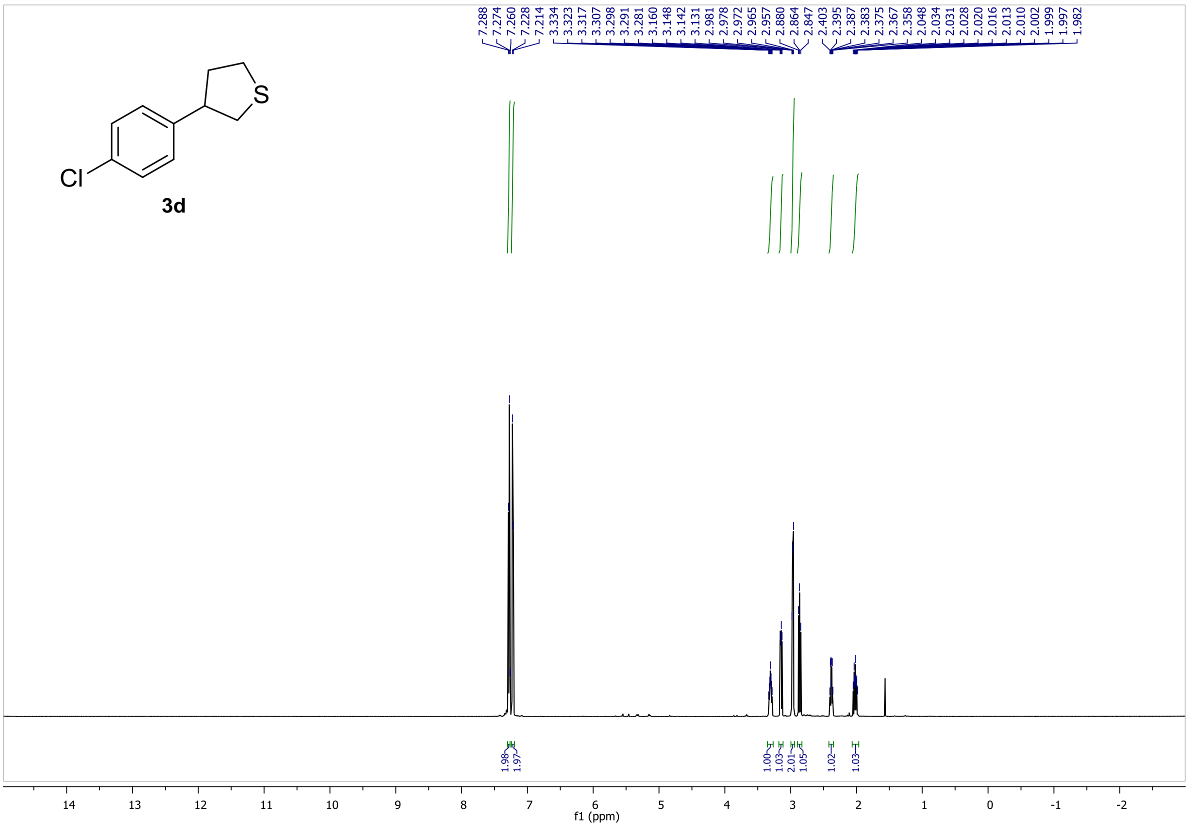

^{13}C NMR, 150 MHz, $CDCl_3$:

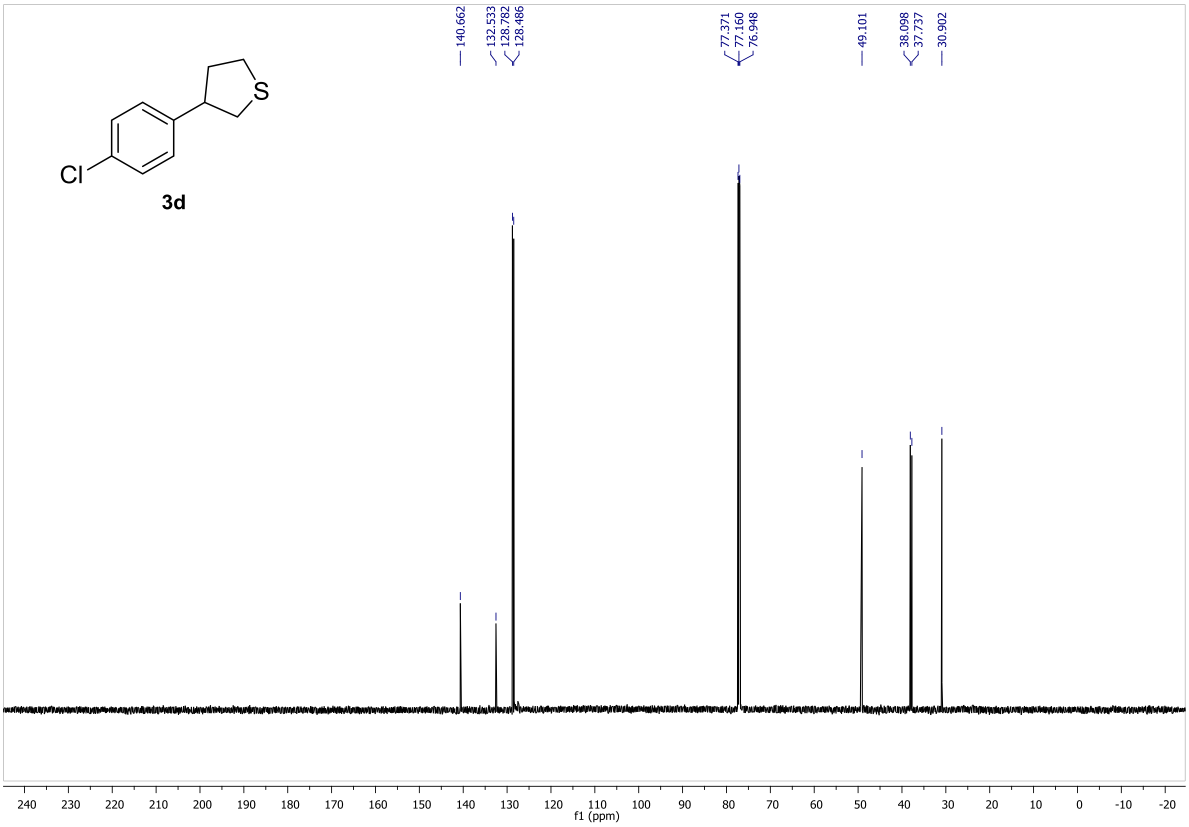

3-(4-chlorophenyl)thietane (3e):

^{1}H NMR, 600 MHz, $CDCl_3$:

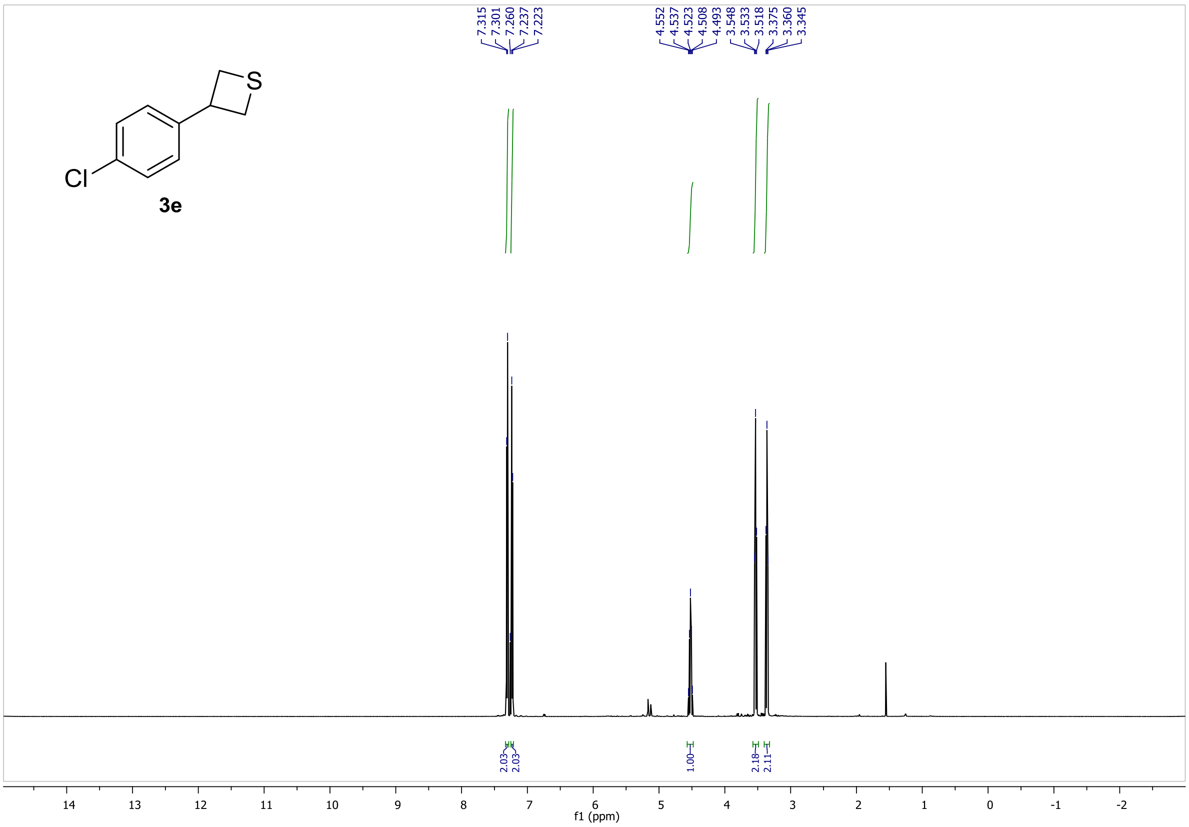

^{13}C NMR, 150 MHz, $CDCl_3$:

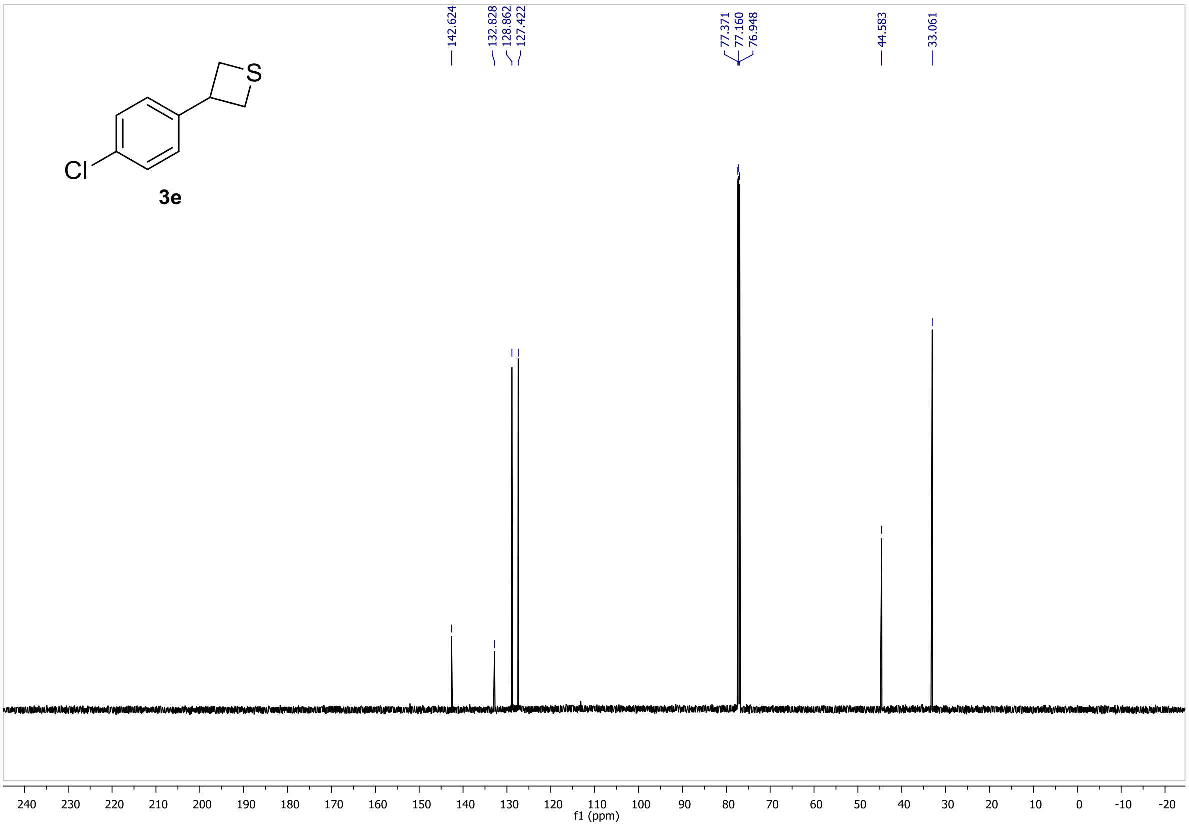

***tert*-butyl 4-(4-chlorophenyl)piperidine-1-carboxylate (3f):**

^{1}H NMR, 600 MHz, $CDCl_3$:

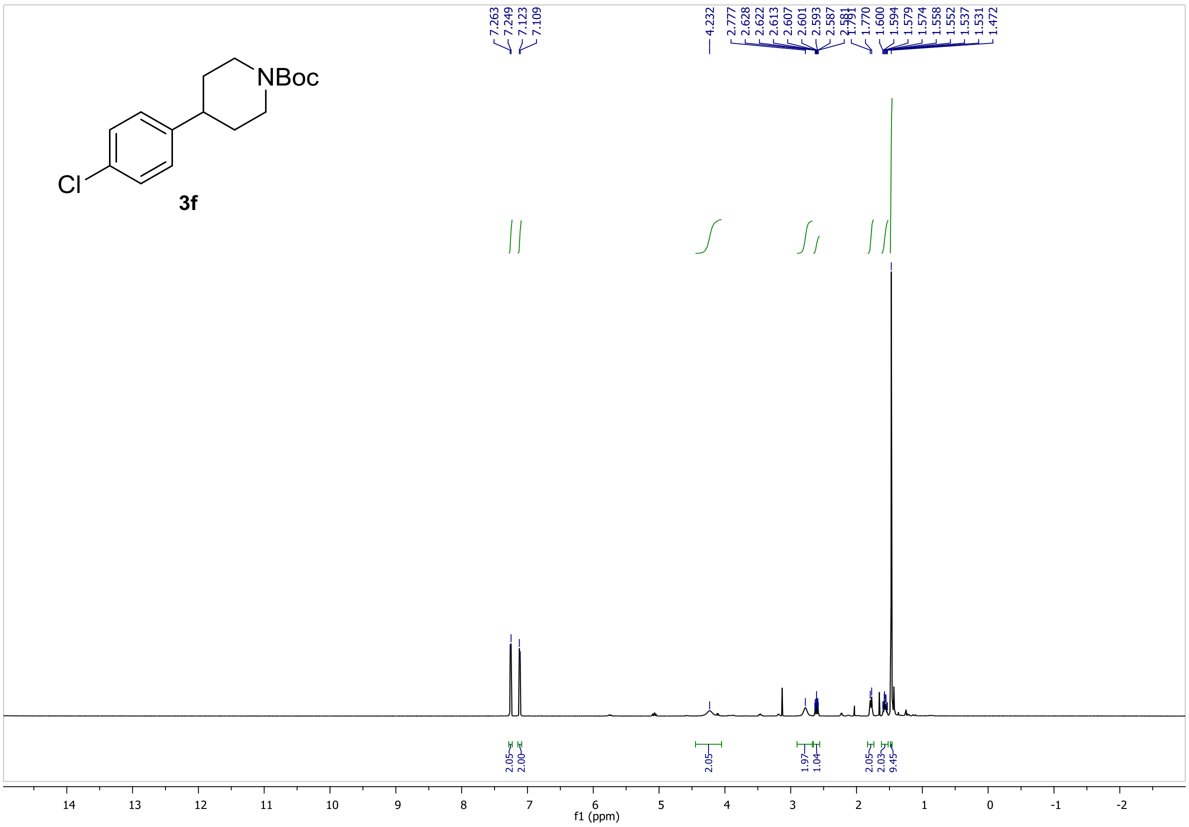

^{13}C NMR, 150 MHz, $CDCl_3$:

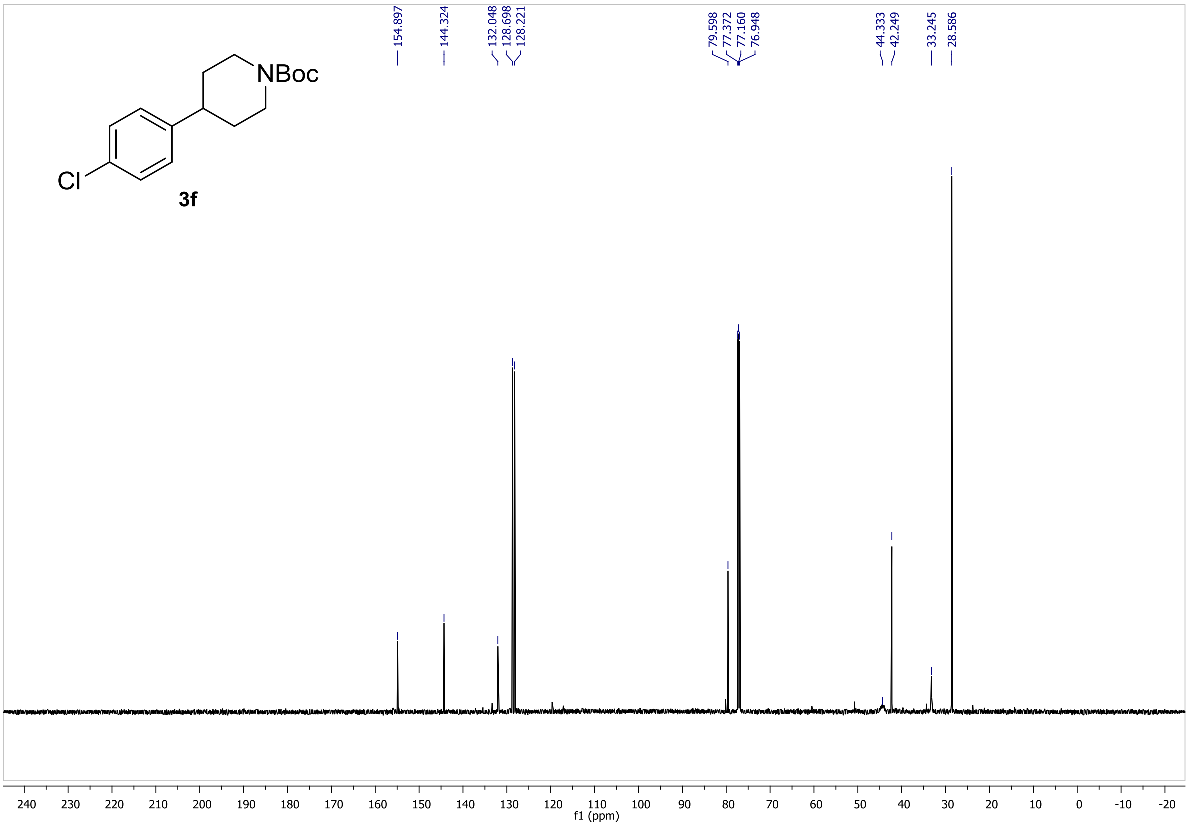

***tert*-butyl 3-(4-chlorophenyl)pyrrolidine-1-carboxylate (3g):**

^{1}H NMR, 600 MHz, $CDCl_3$:

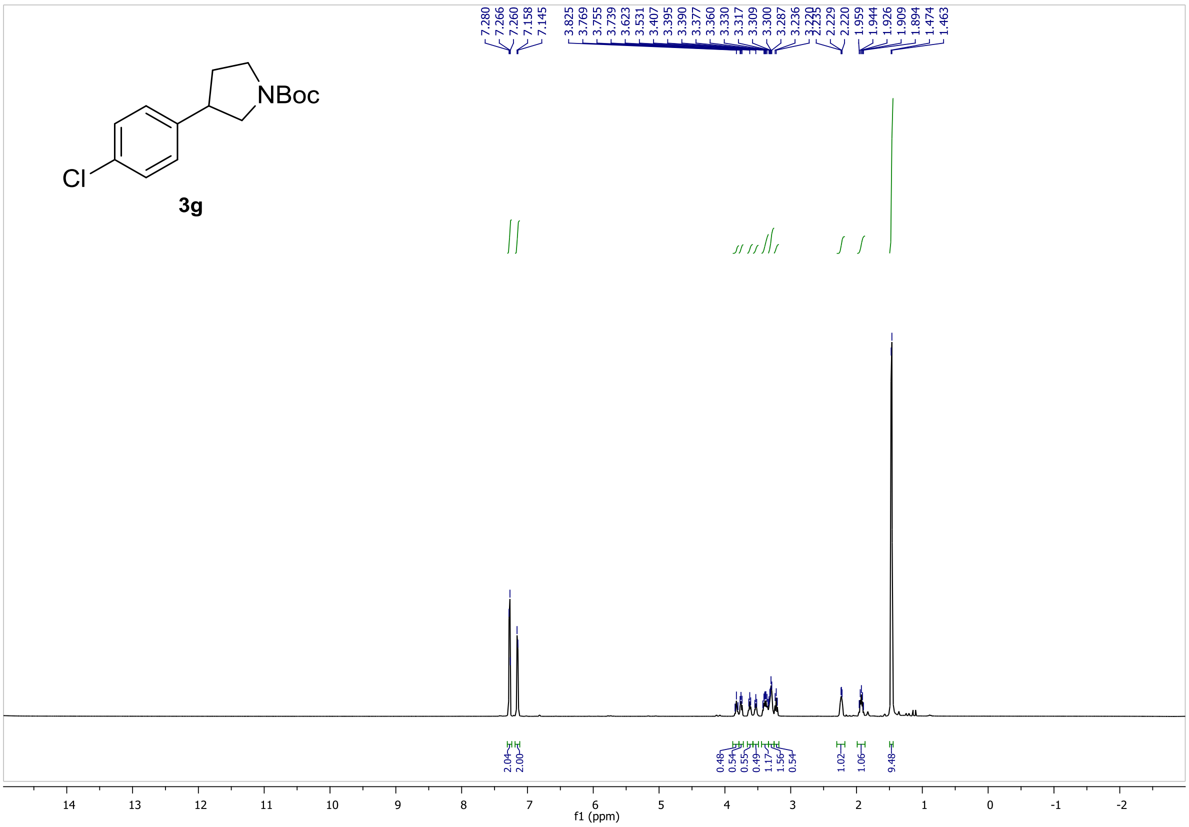

^{13}C NMR, 150 MHz, $CDCl_3$:

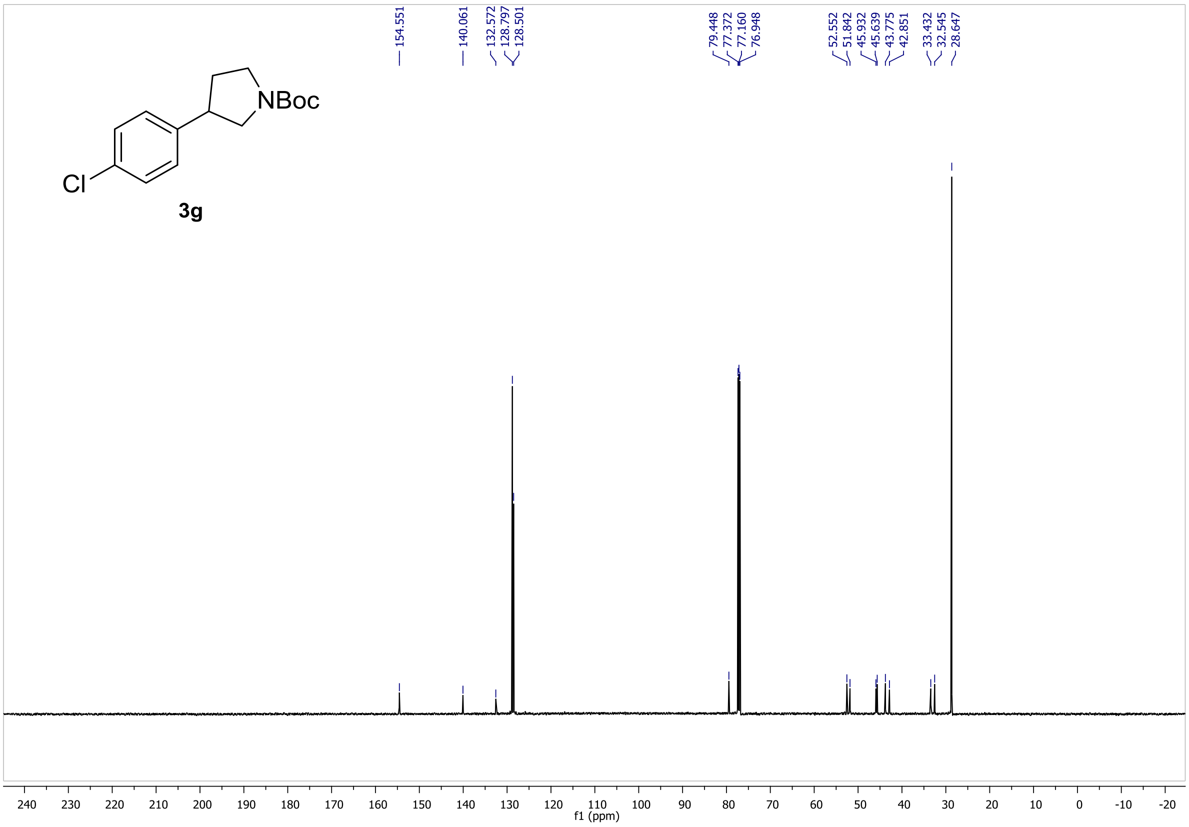

***tert*-butyl 3-(4-chlorophenyl)azetidine-1-carboxylate (3h):**

^{1}H NMR, 600 MHz, $CDCl_3$:

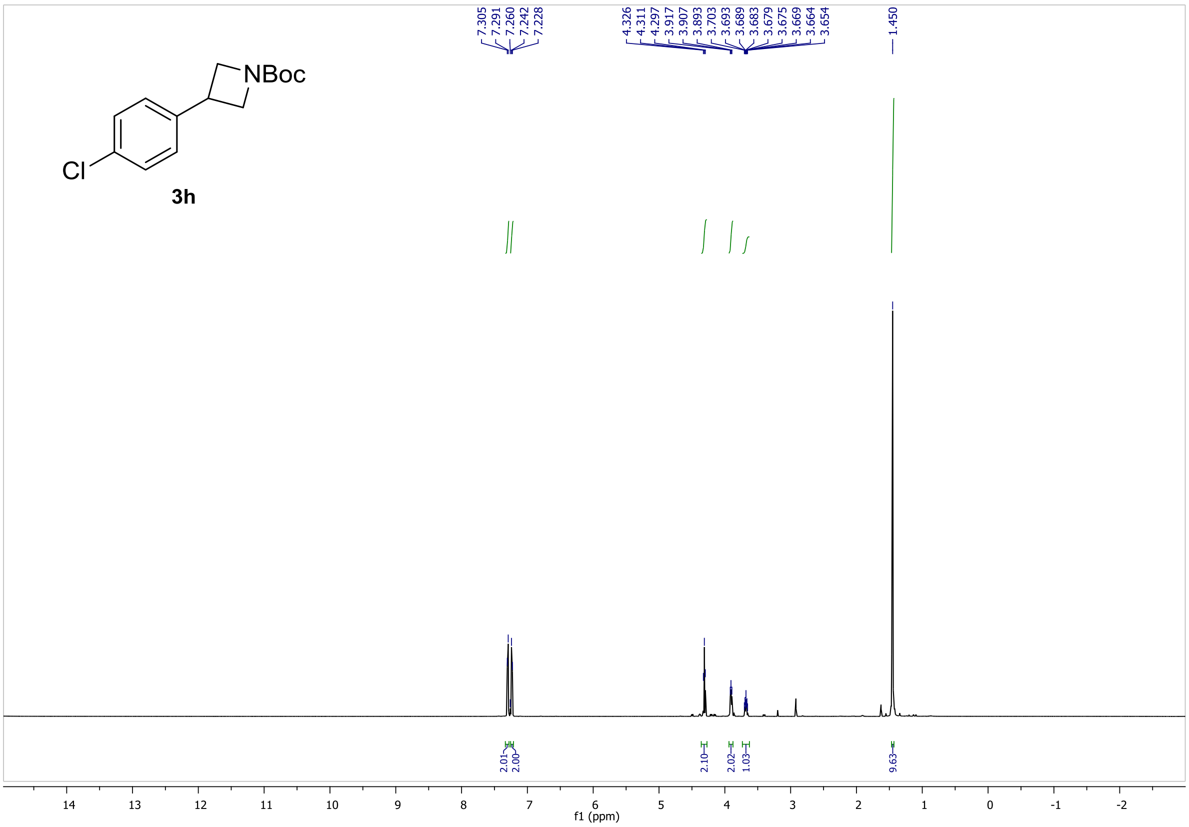

^{13}C NMR, 150 MHz, $CDCl_3$:

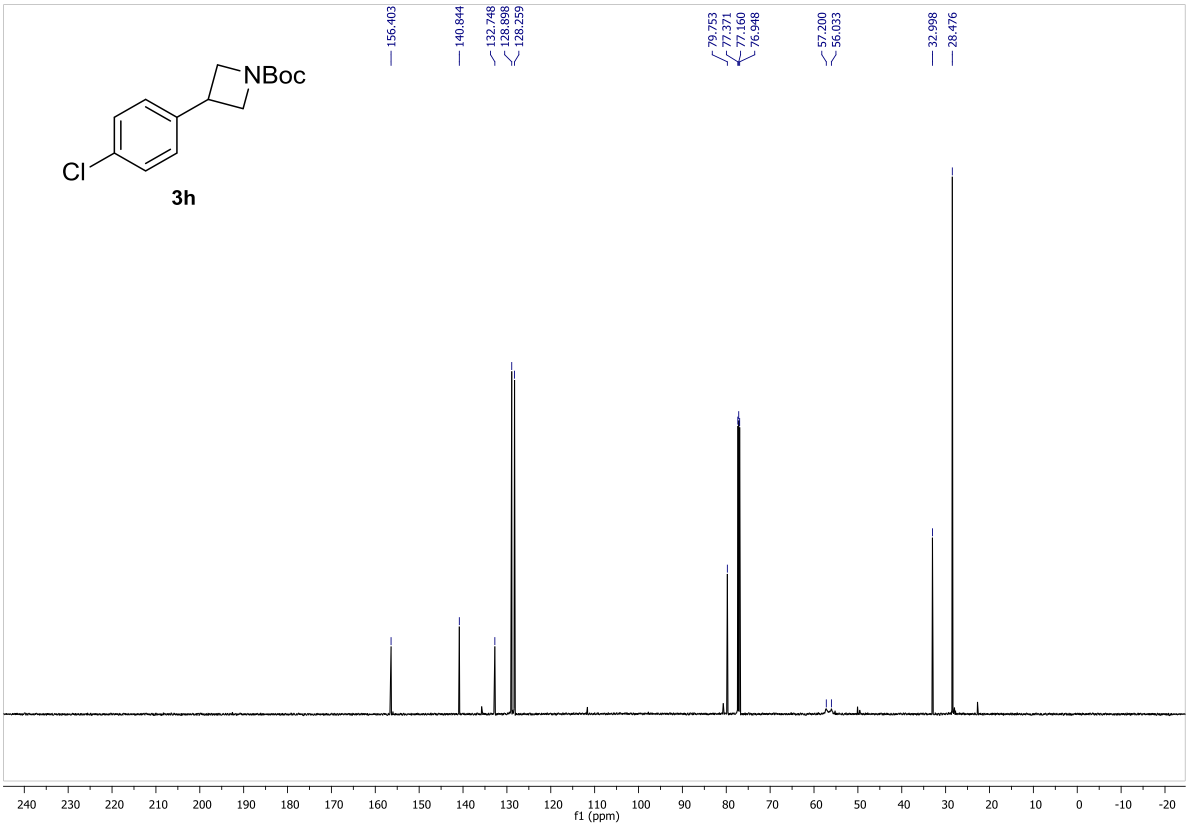

1-chloro-4-cyclohexylbenzene (3i):

^{1}H NMR, 600 MHz, $CDCl_3$:

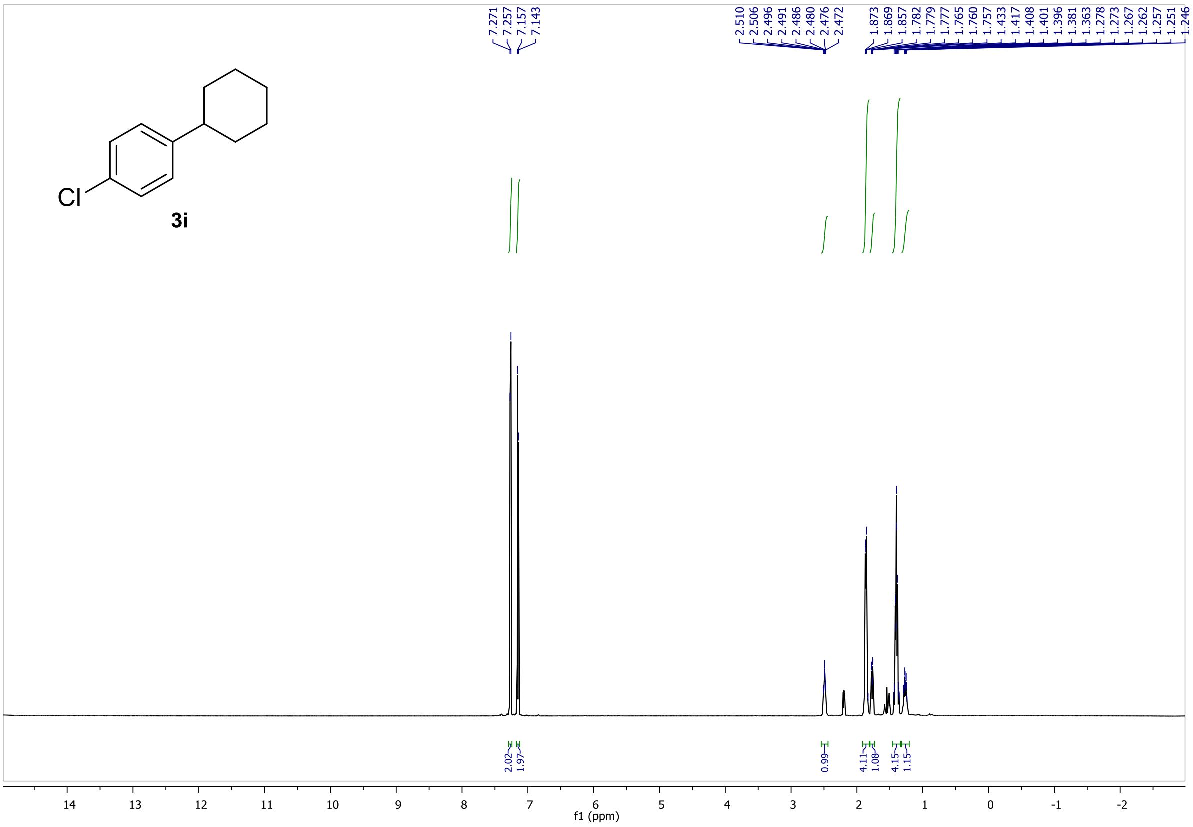

^{13}C NMR, 150 MHz, $CDCl_3$:

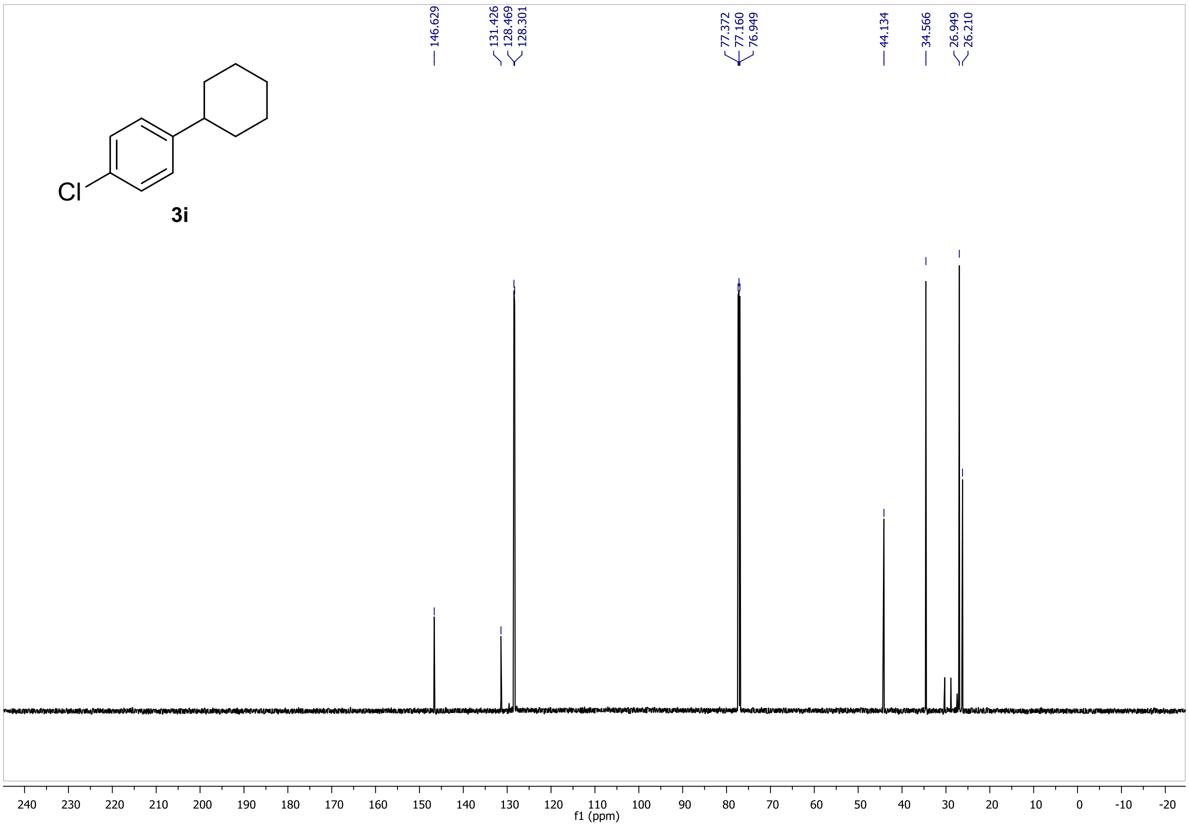

1-chloro-4-cyclopentylbenzene (3j):

^{1}H NMR, 600 MHz, $CDCl_3$:

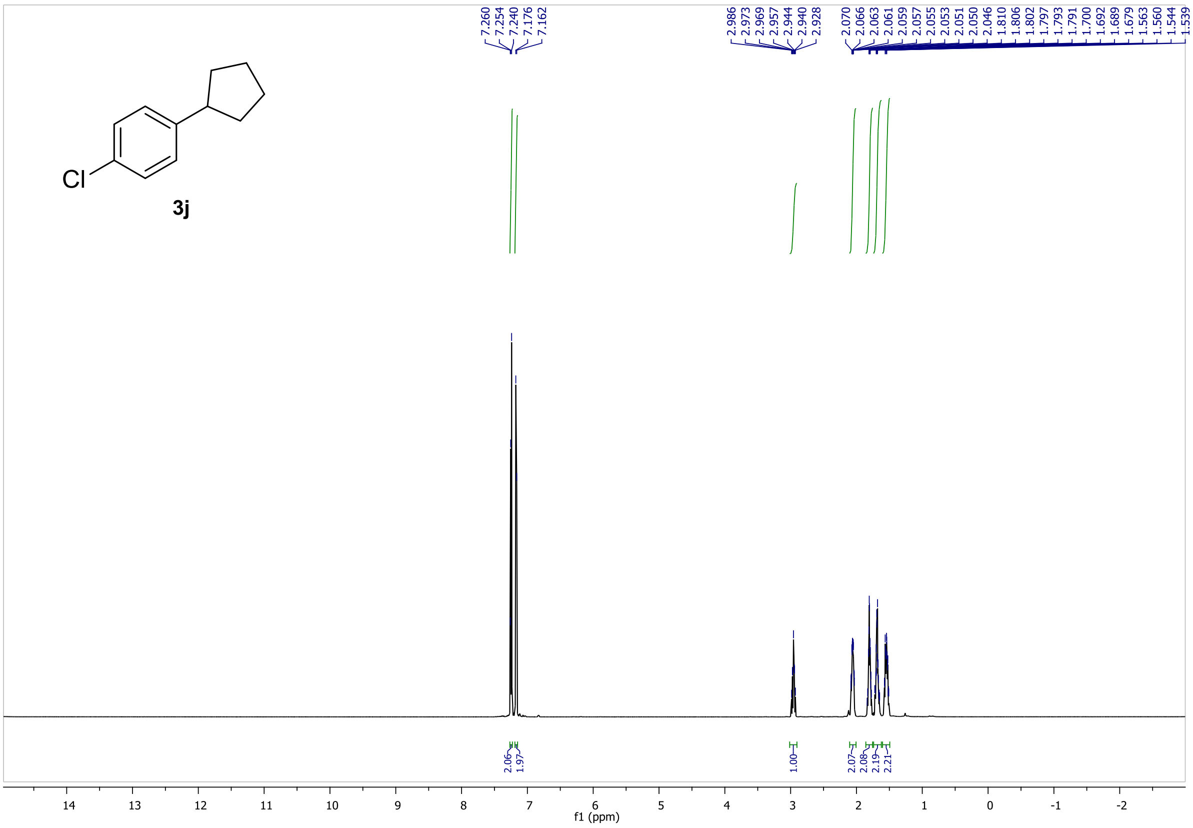

^{13}C NMR, 150 MHz, $CDCl_3$:

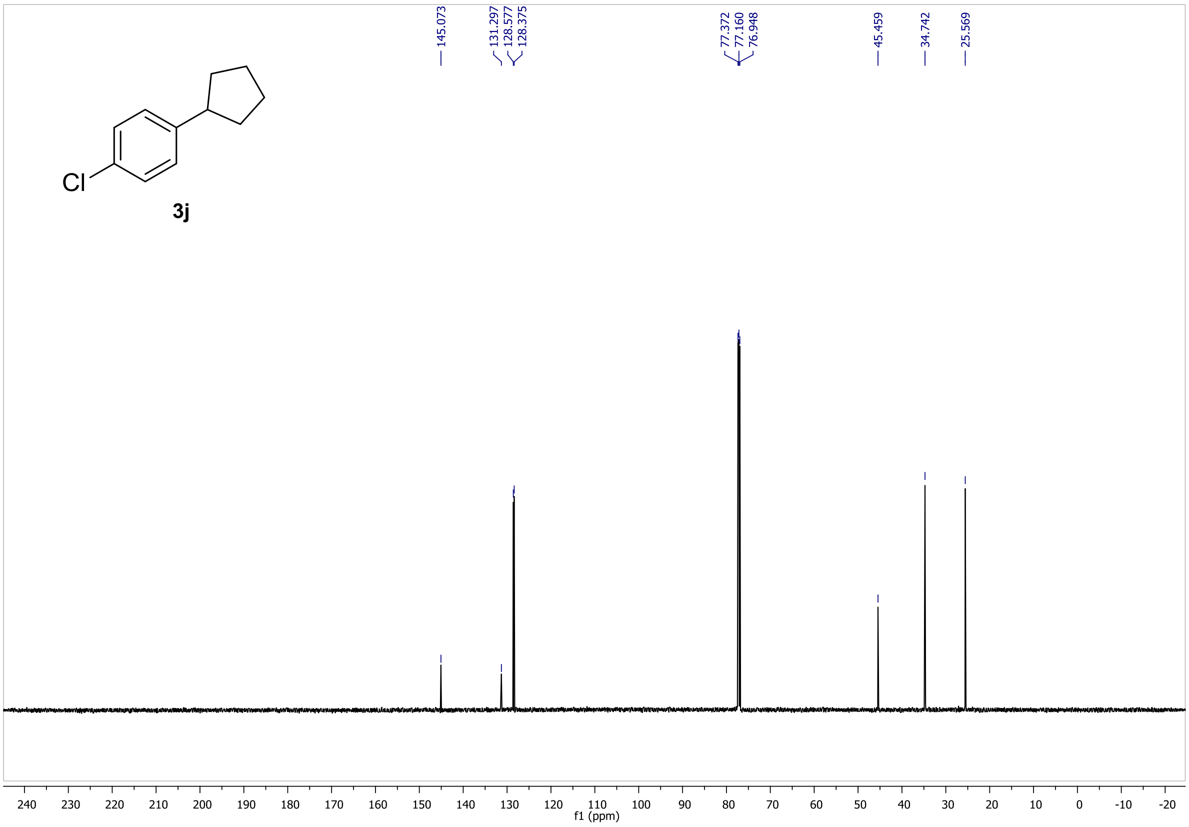

1-chloro-4-cyclobutylbenzene (3k):

^{1}H NMR, 600 MHz, $CDCl_3$:

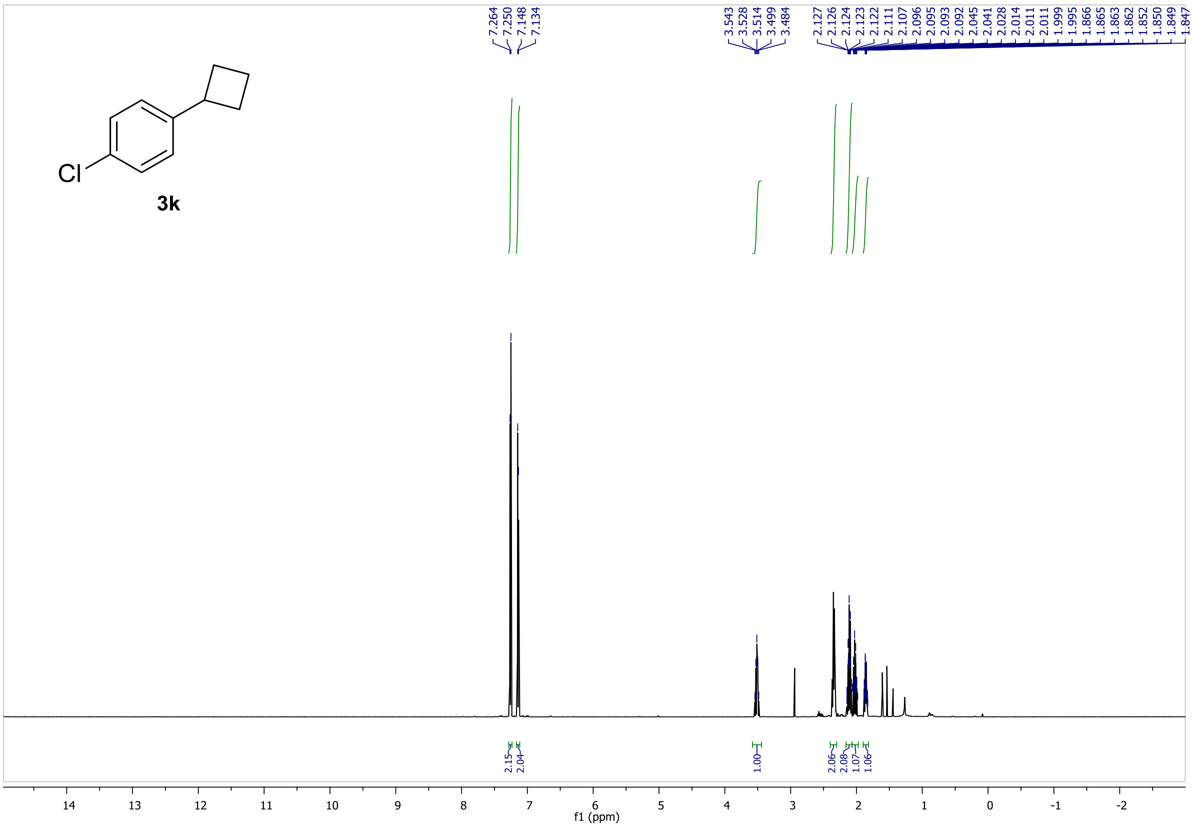

^{13}C NMR, 150 MHz, $CDCl_3$:

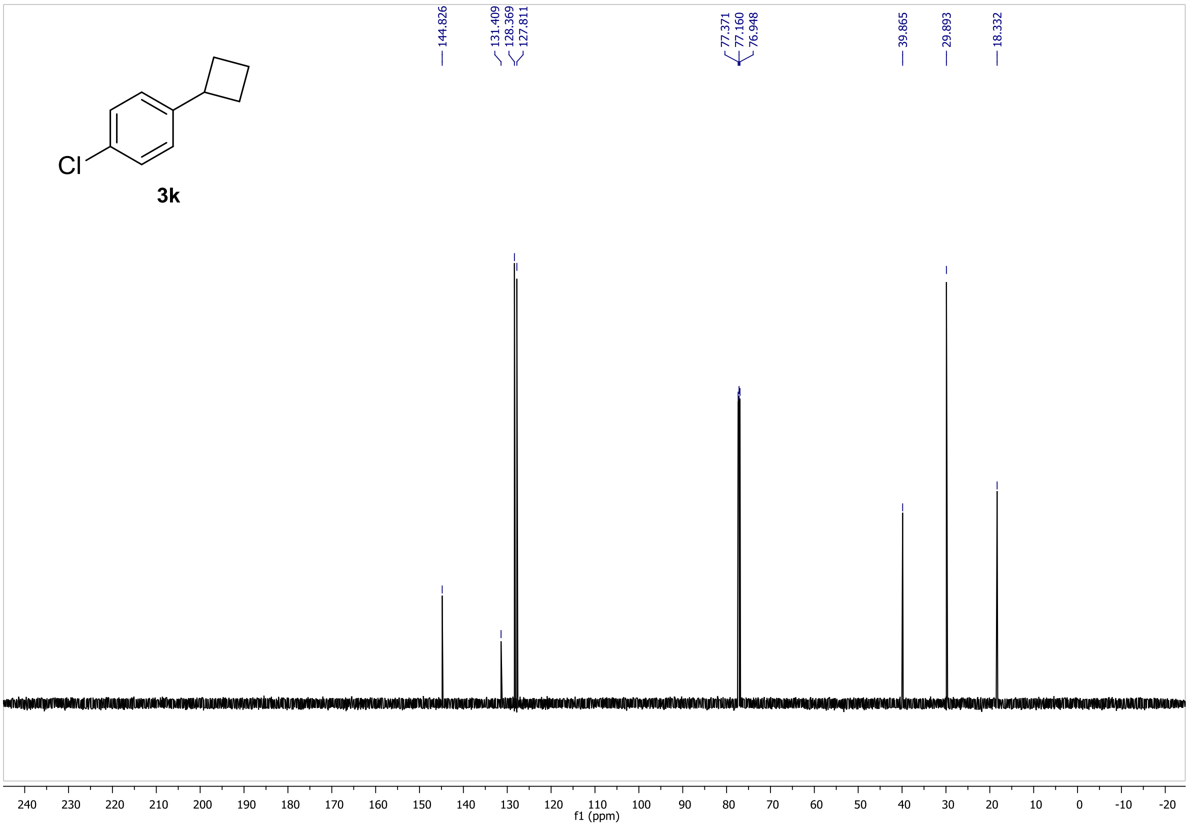

2-(4-chlorophenyl)-1,2,3,4-tetrahydronaphthalene (3l):

^{1}H NMR, 600 MHz, $CDCl_3$:

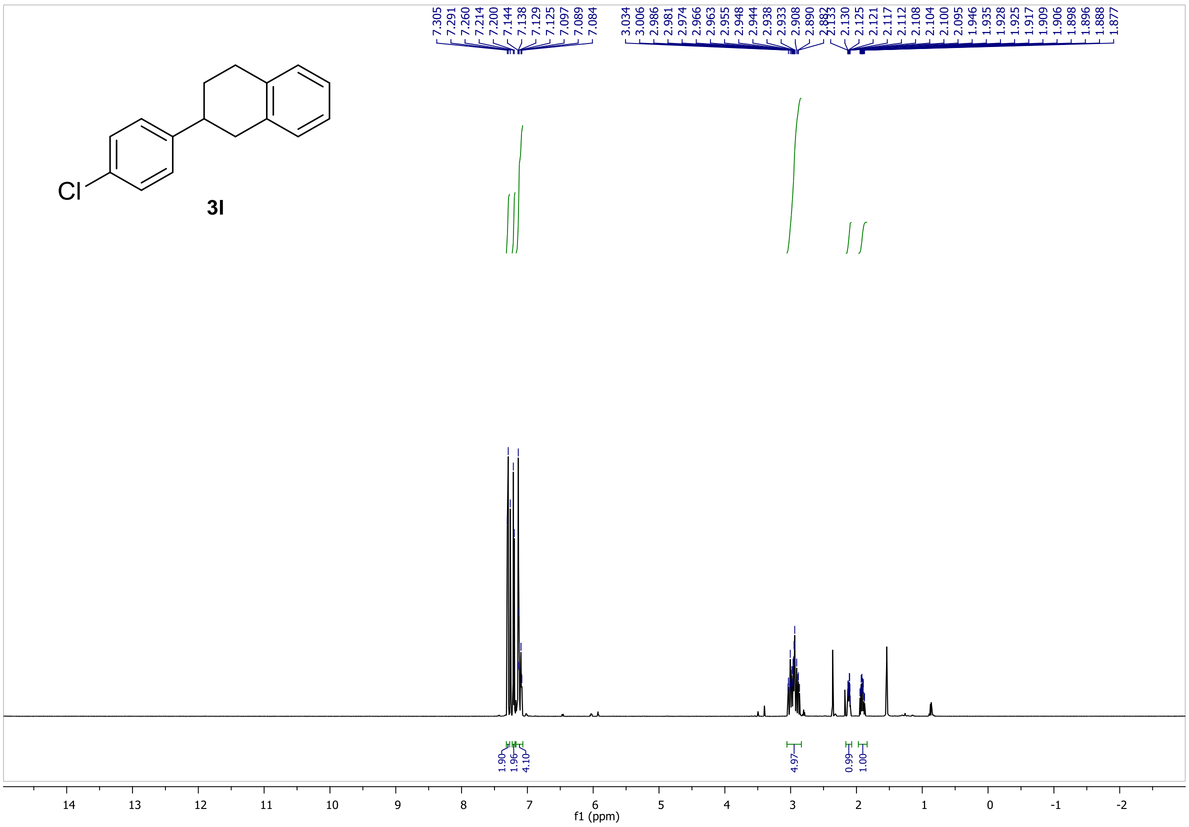

^{13}C NMR, 150 MHz, $CDCl_3$:

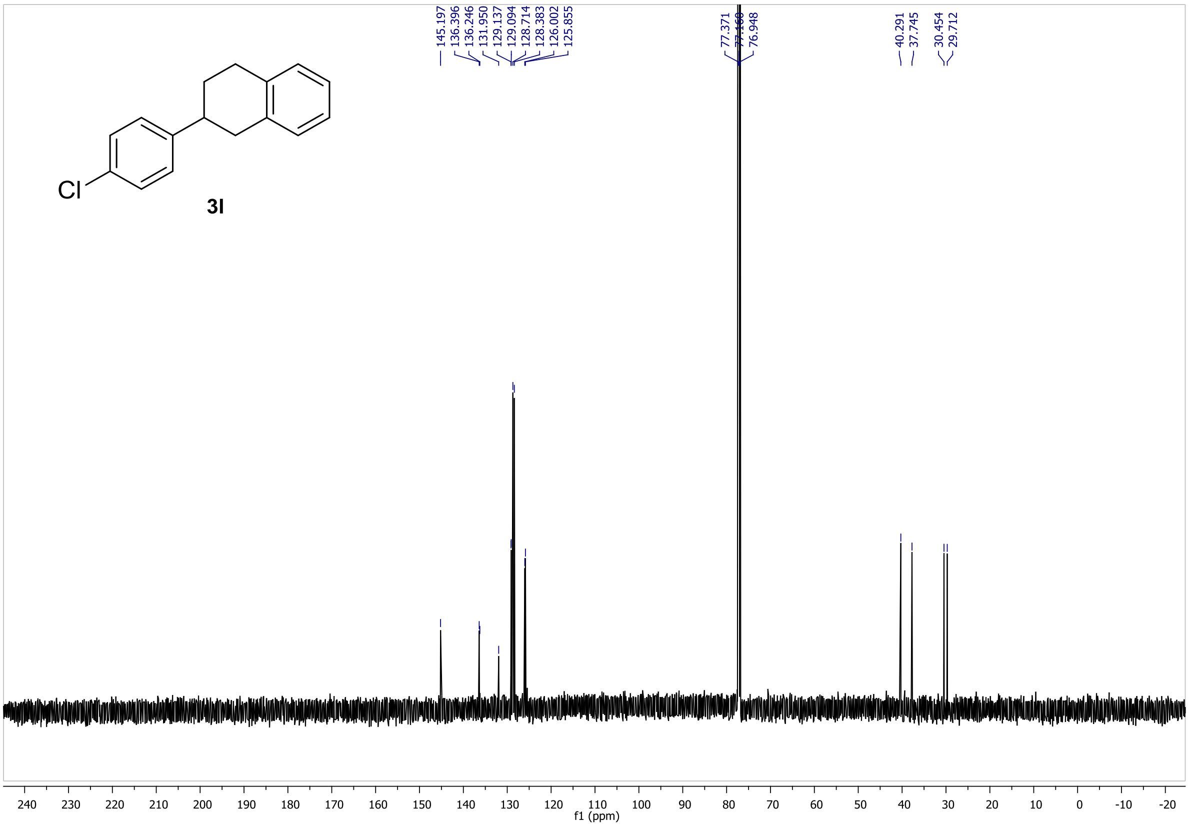

2-(4-chlorophenyl)adamantane (3m):

^{1}H NMR, 600 MHz, $CDCl_3$:

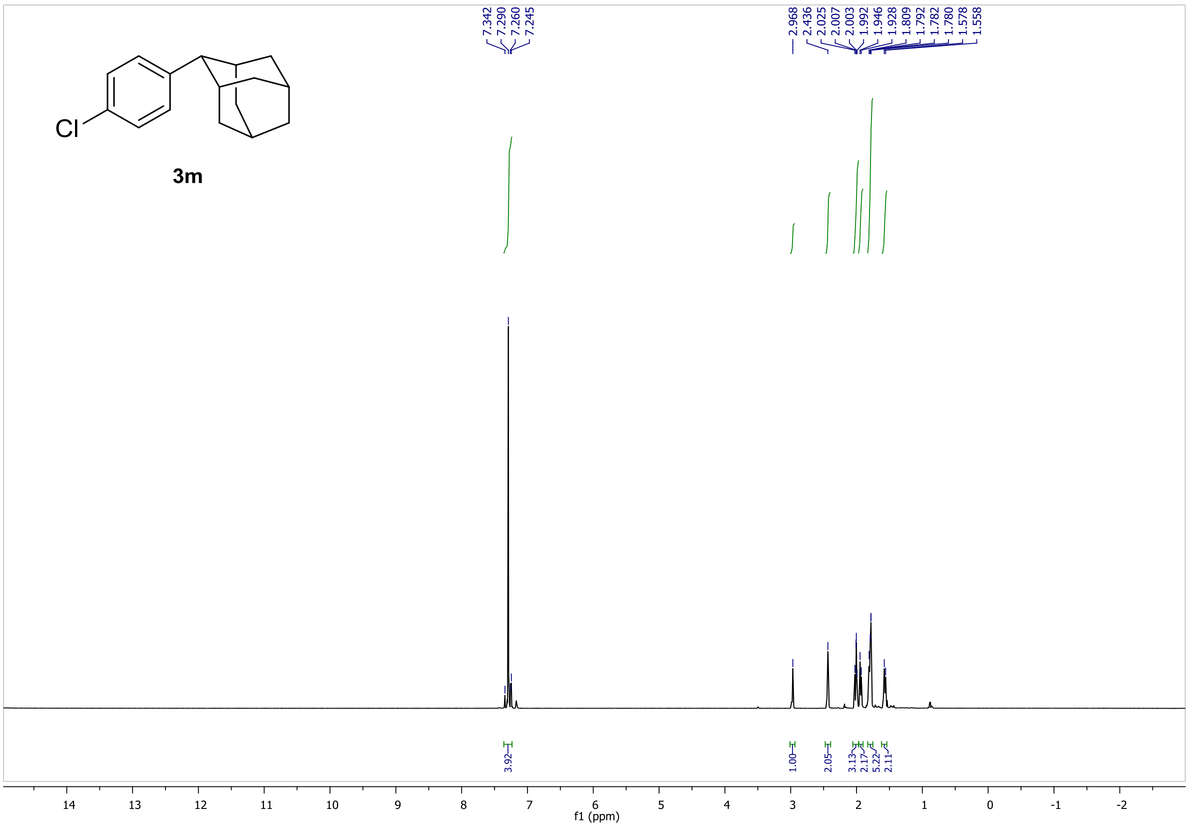

^{13}C NMR, 150 MHz, $CDCl_3$:

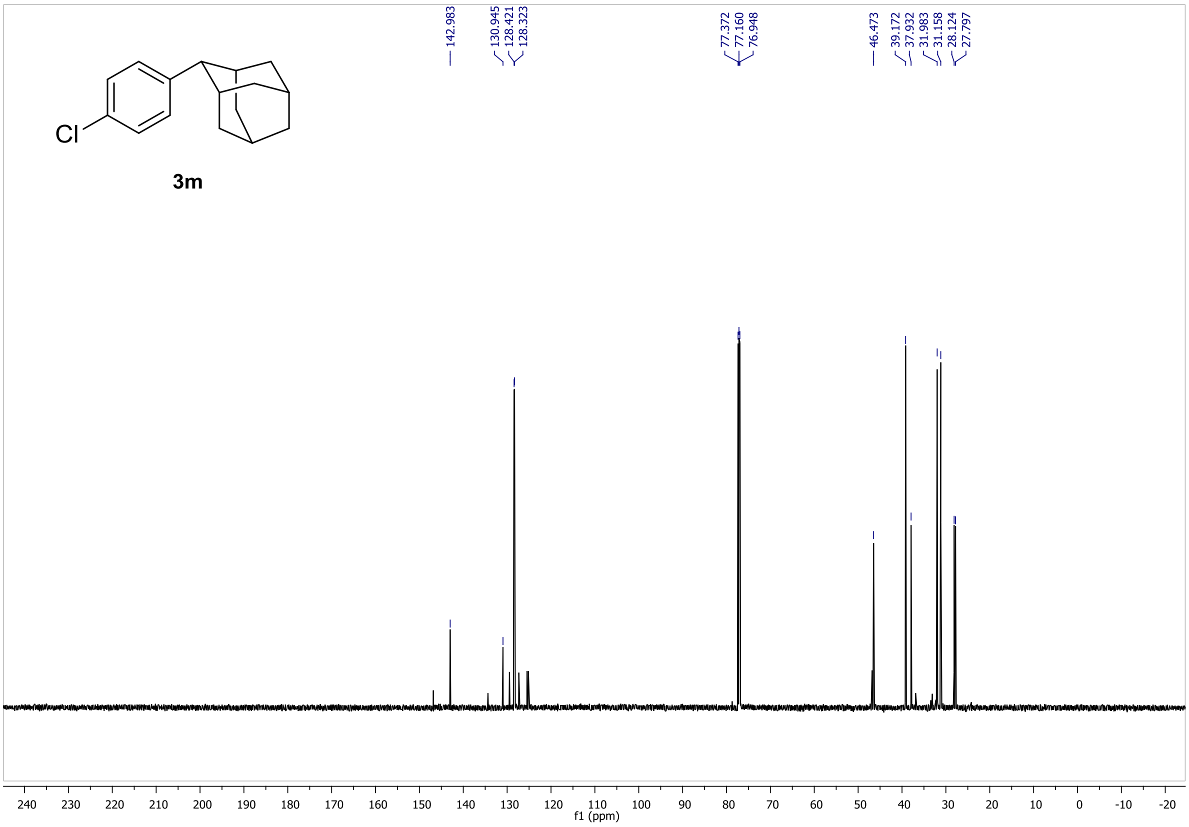

1-chloro-4-(1-cyclopropylethyl)benzene (3n):

^{1}H NMR, 600 MHz, $CDCl_3$:

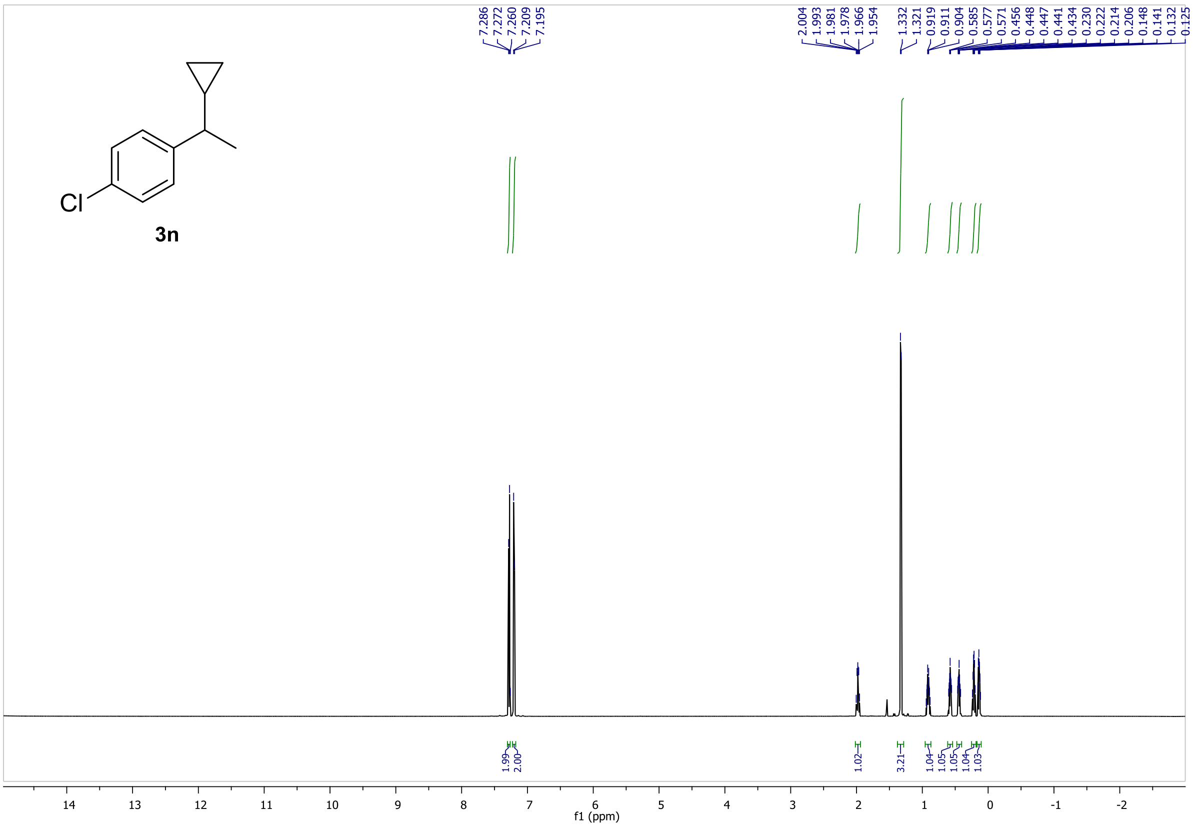

^{13}C NMR, 150 MHz, $CDCl_3$:

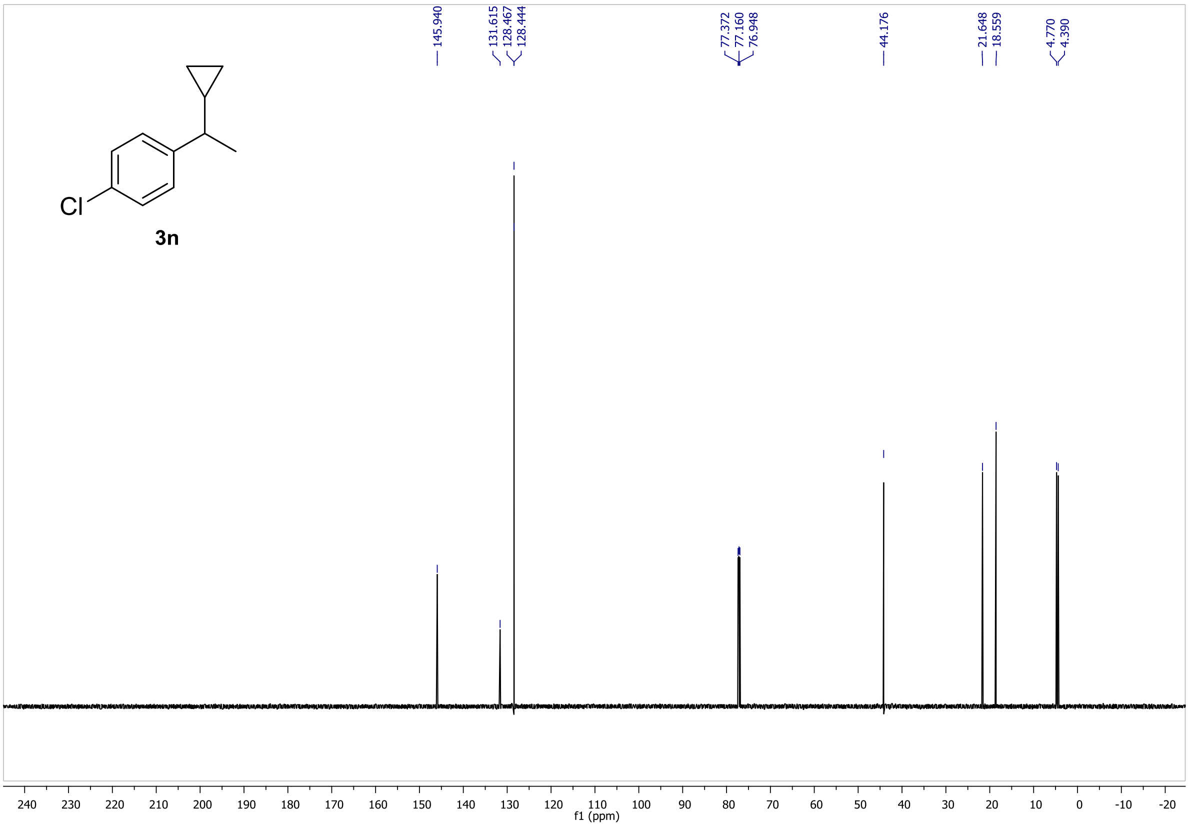

1-chloro-4-(hex-5-en-2-yl)benzene (3o):

^{1}H NMR, 600 MHz, $CDCl_3$:

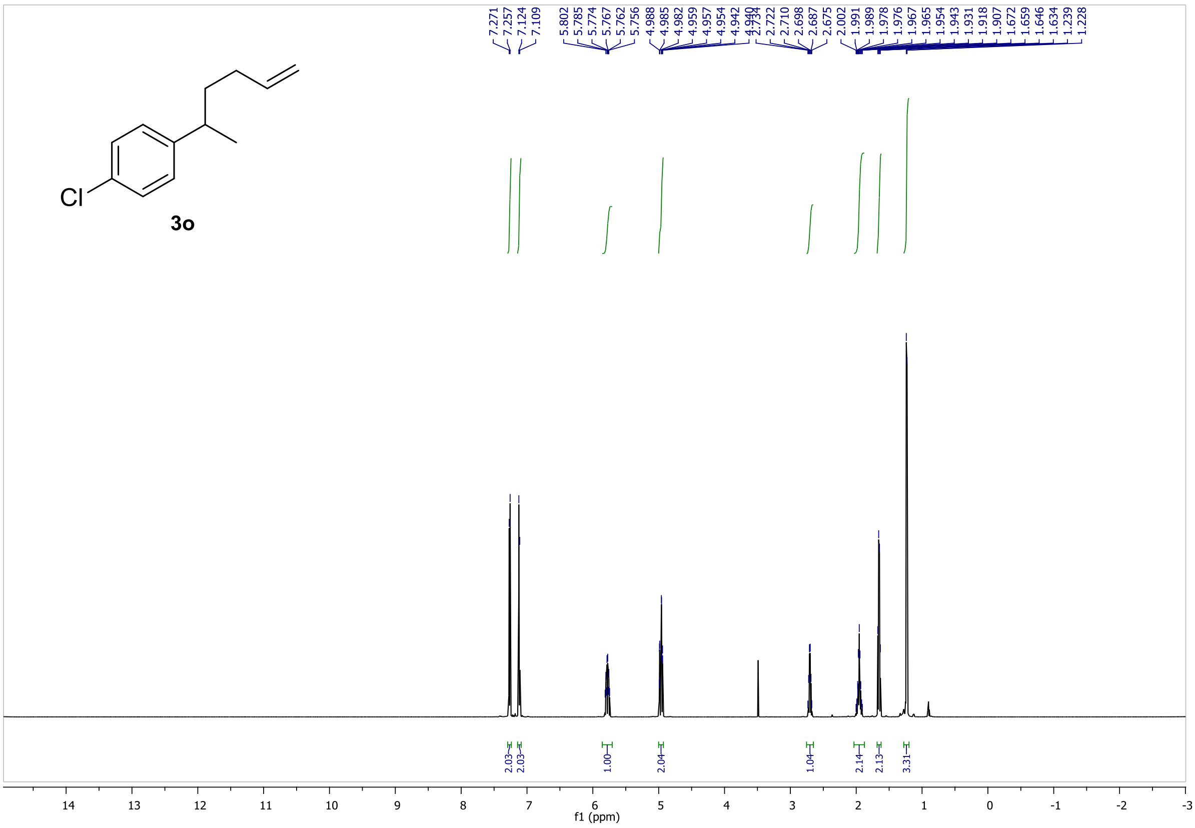

^{13}C NMR, 150 MHz, $CDCl_3$:

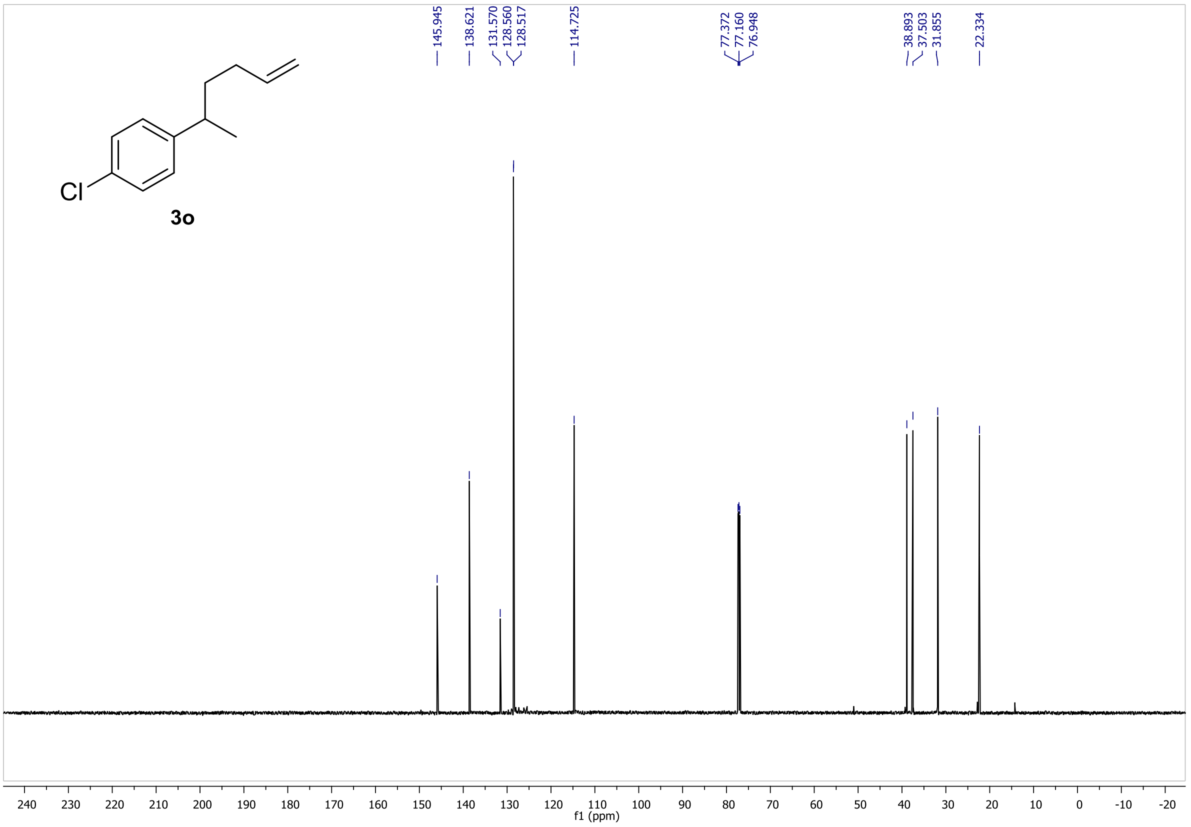

1-chloro-4-(4,4-dimethylhept-6-yn-2-yl)benzene (3p):

^{1}H NMR, 600 MHz, $CDCl_3$:

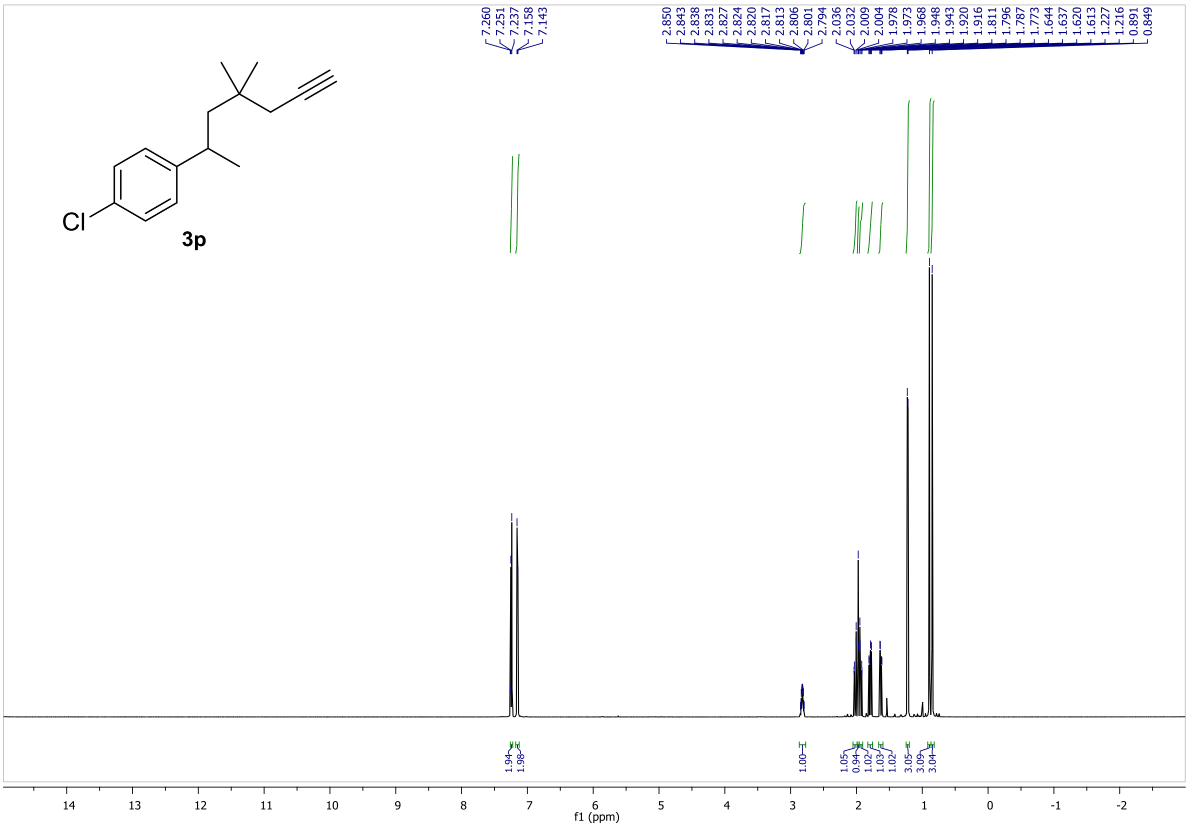

^{13}C NMR, 150 MHz, $CDCl_3$:

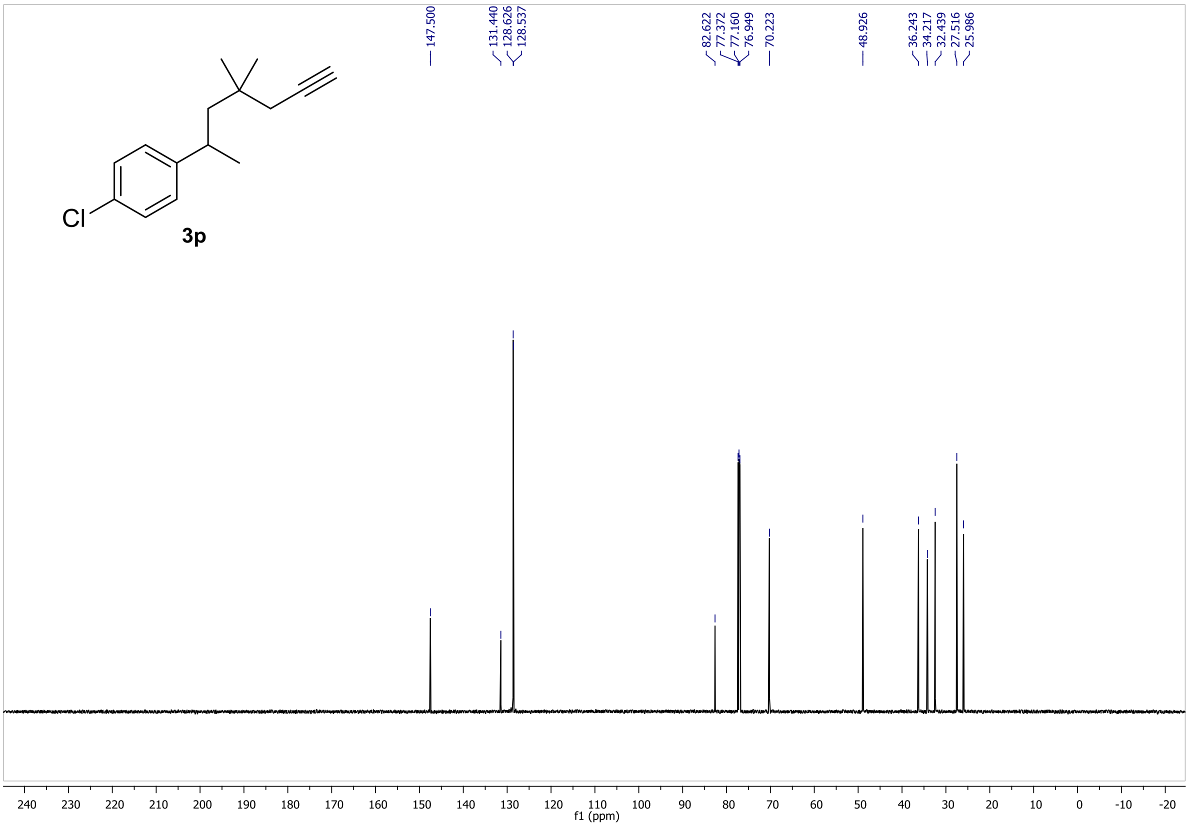

8-(4-chlorophenyl)-1,4-dioxaspiro[4.5]decane (3q):

^{1}H NMR, 600 MHz, $CDCl_3$:

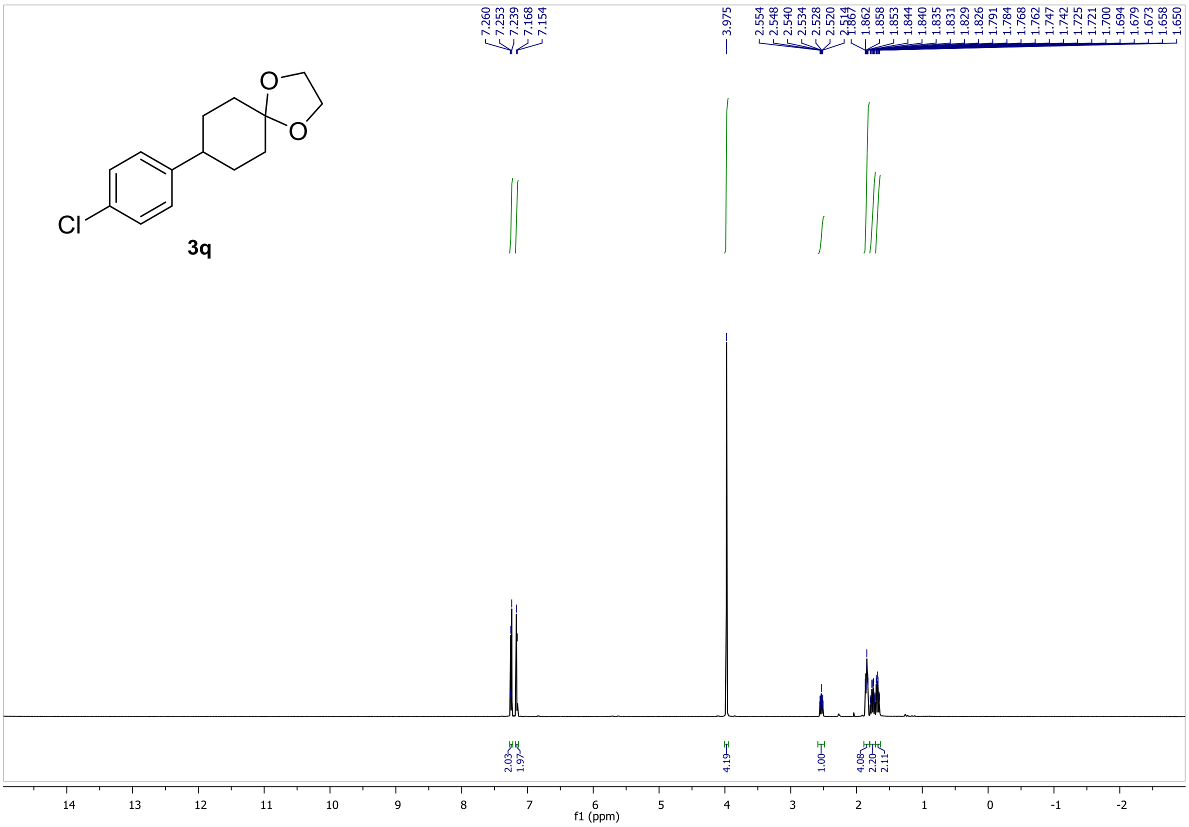

^{13}C NMR, 150 MHz, $CDCl_3$:

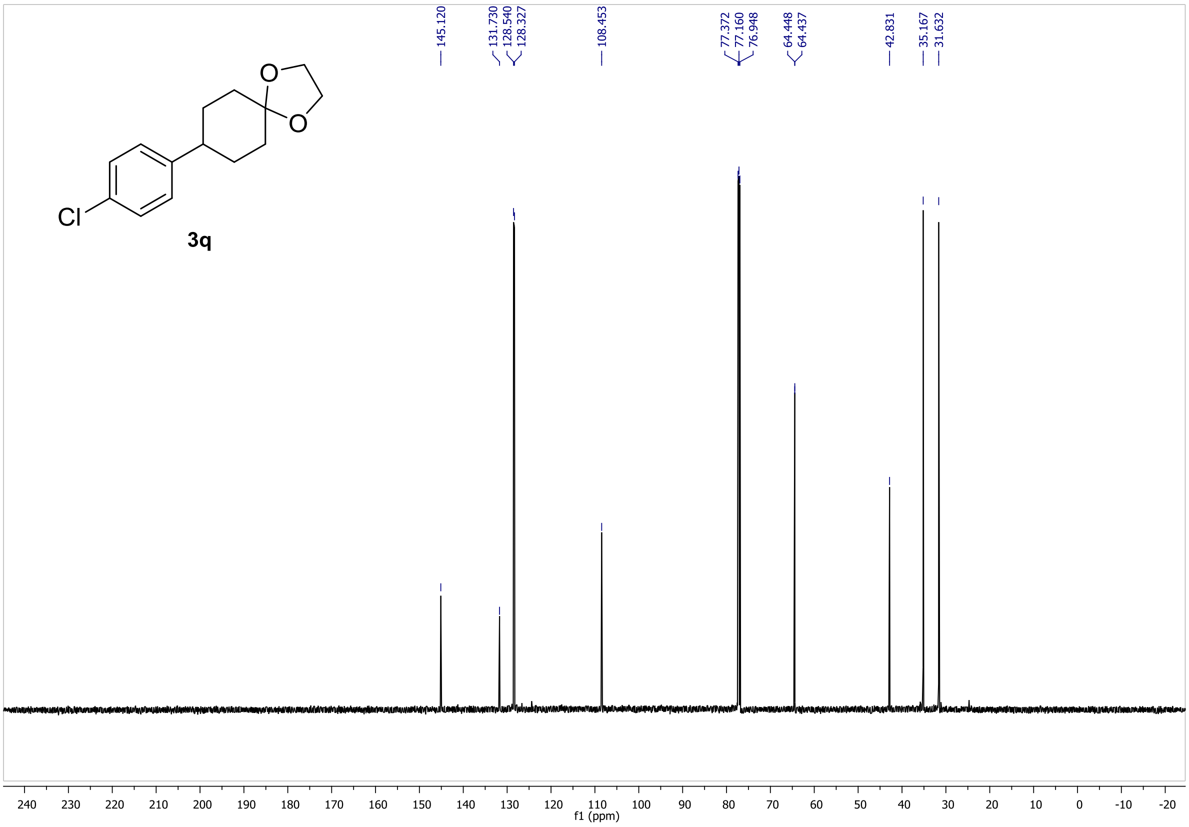

Diethyl (2-(4-chlorophenyl)propyl)phosphonate (3r):

^{1}H NMR, 600 MHz, $CDCl_3$:

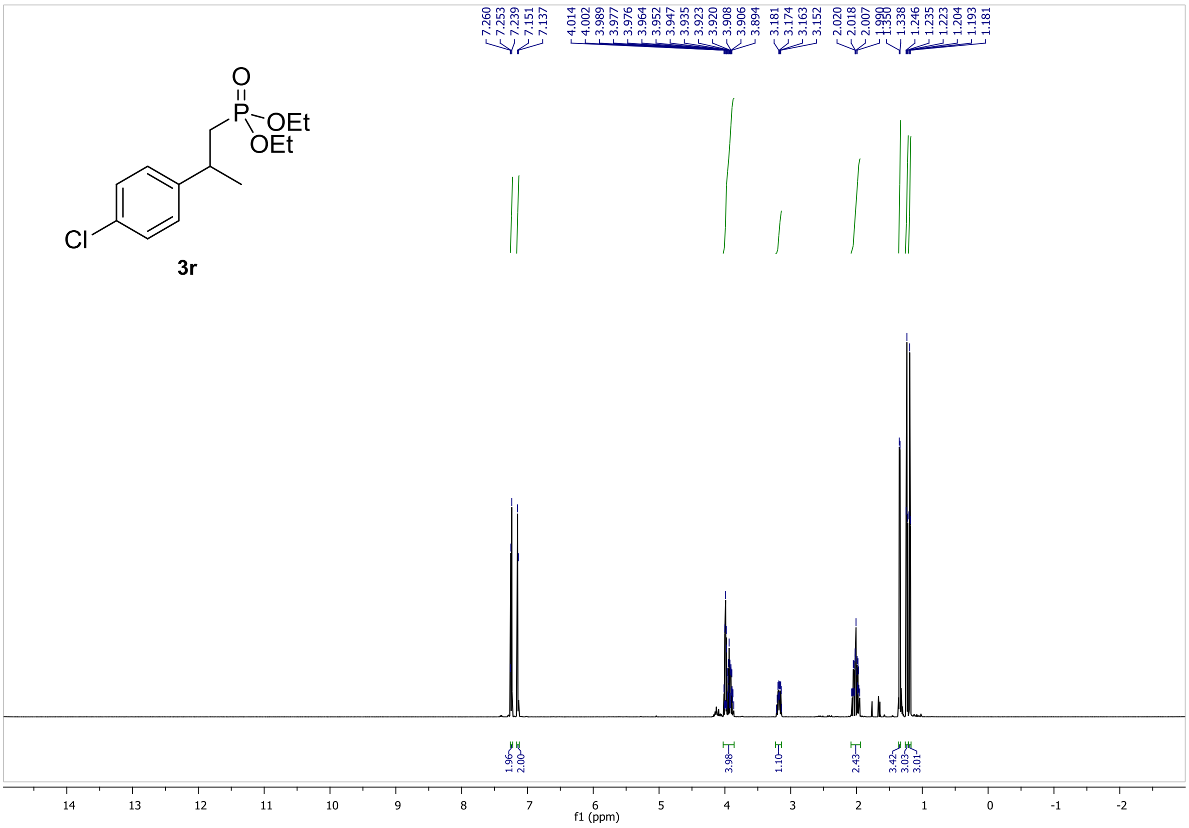

^{13}C NMR, 150 MHz, $CDCl_3$:

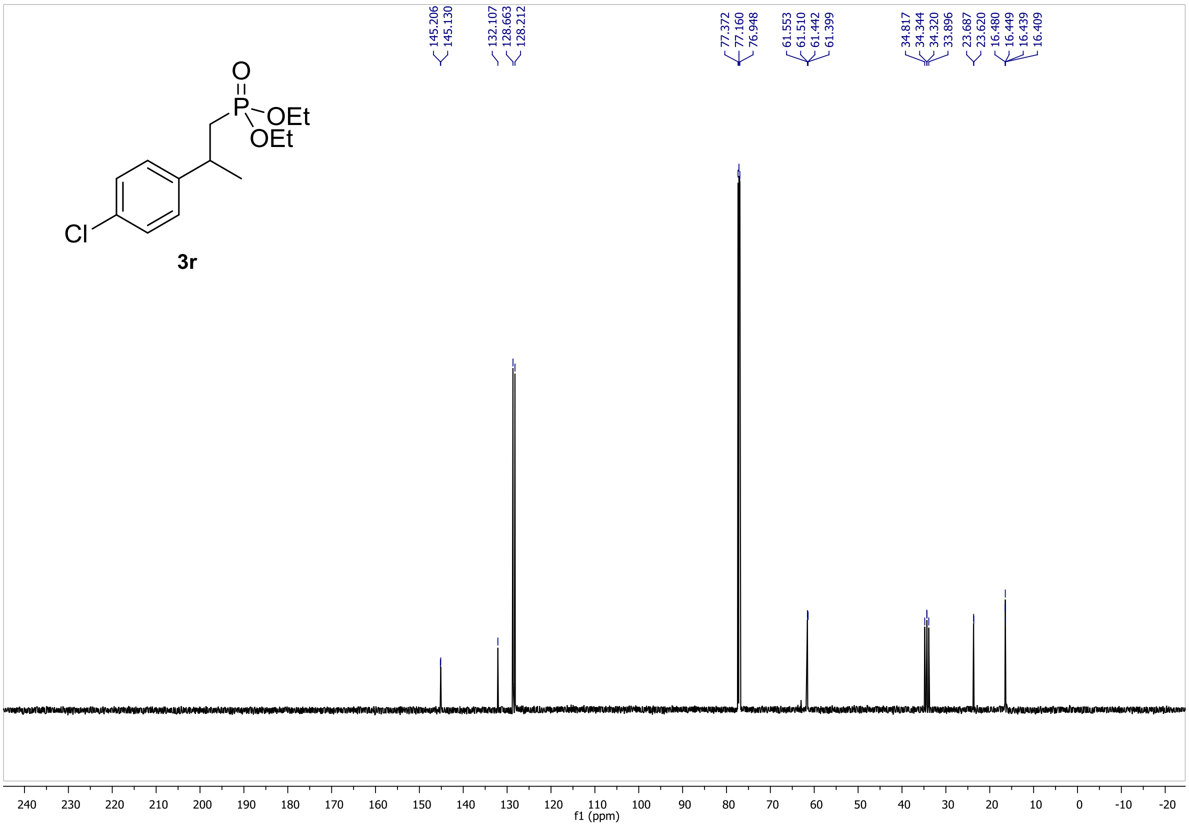

^{31}P NMR, 245 MHz, CDCl$_3$:

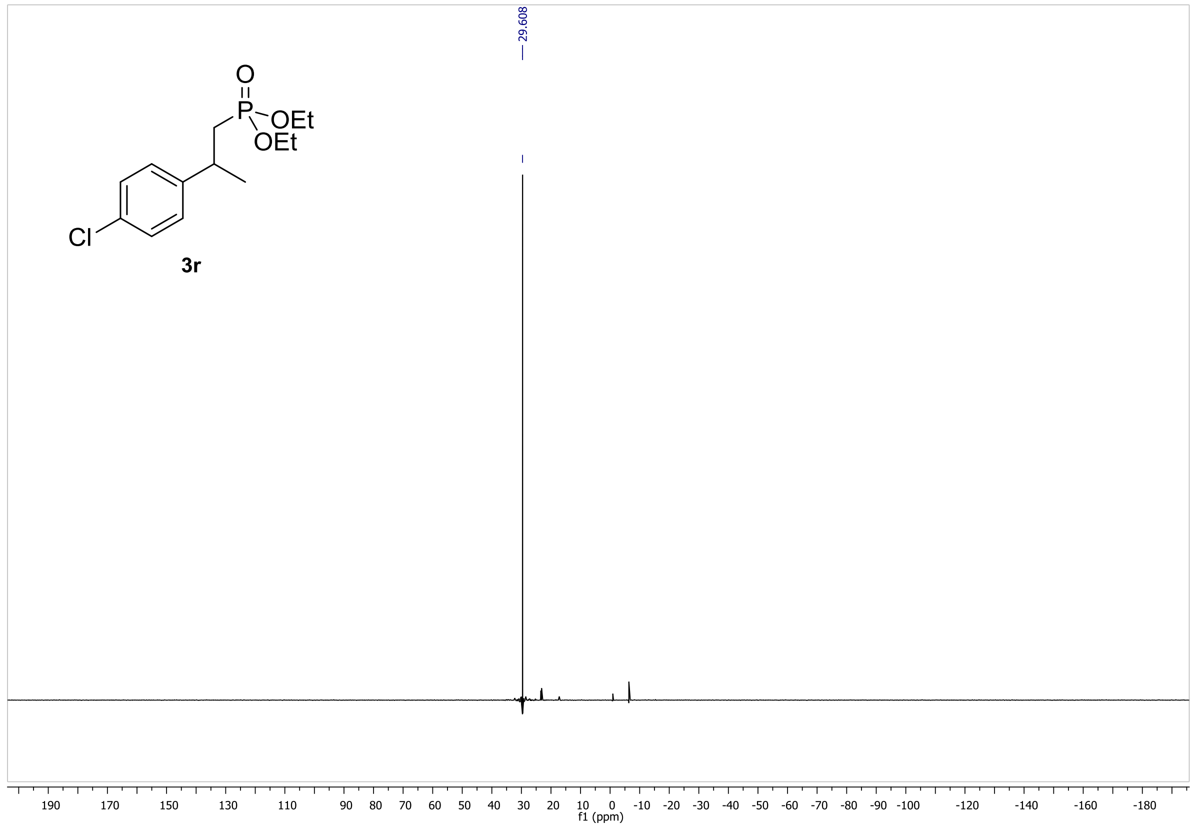

4-(4-chlorophenyl)tetrahydro-2*H*-thiopyran 1,1-dioxide (3s):

^{1}H NMR, 600 MHz, $CDCl_3$:

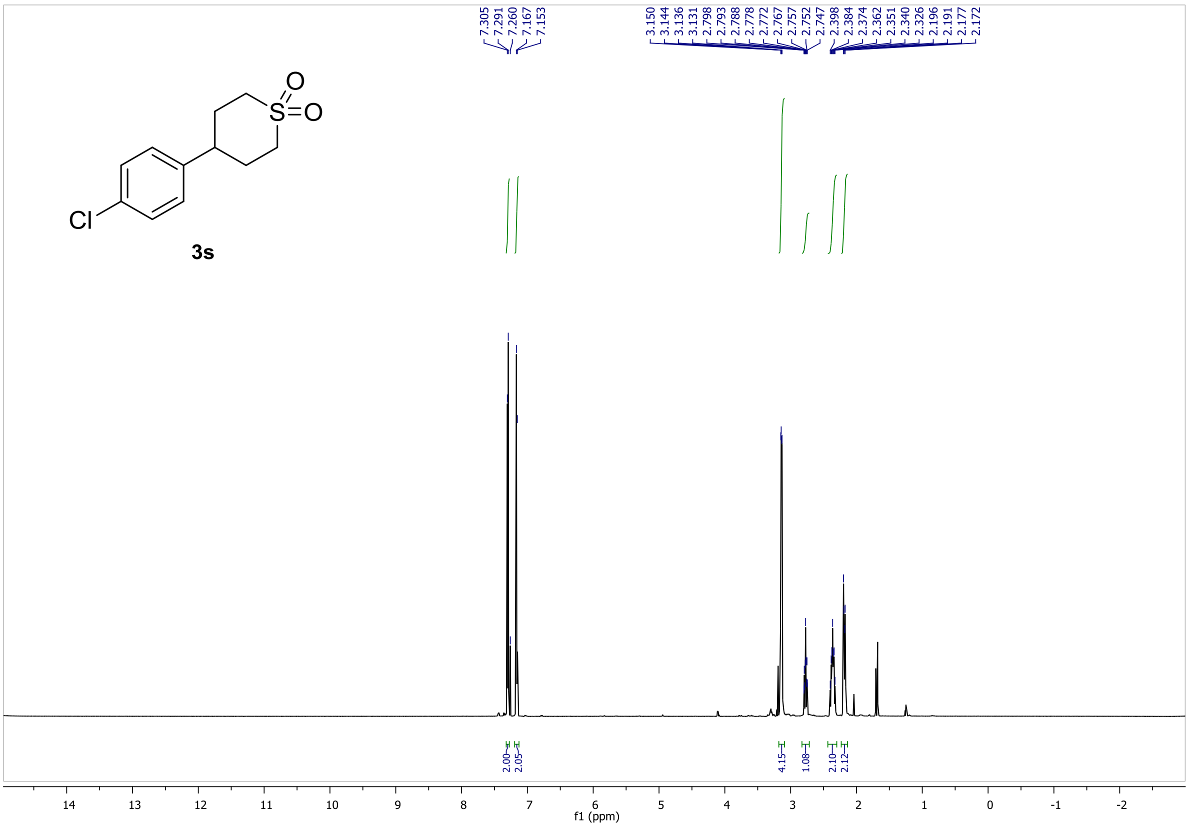

^{13}C NMR, 150 MHz, $CDCl_3$:

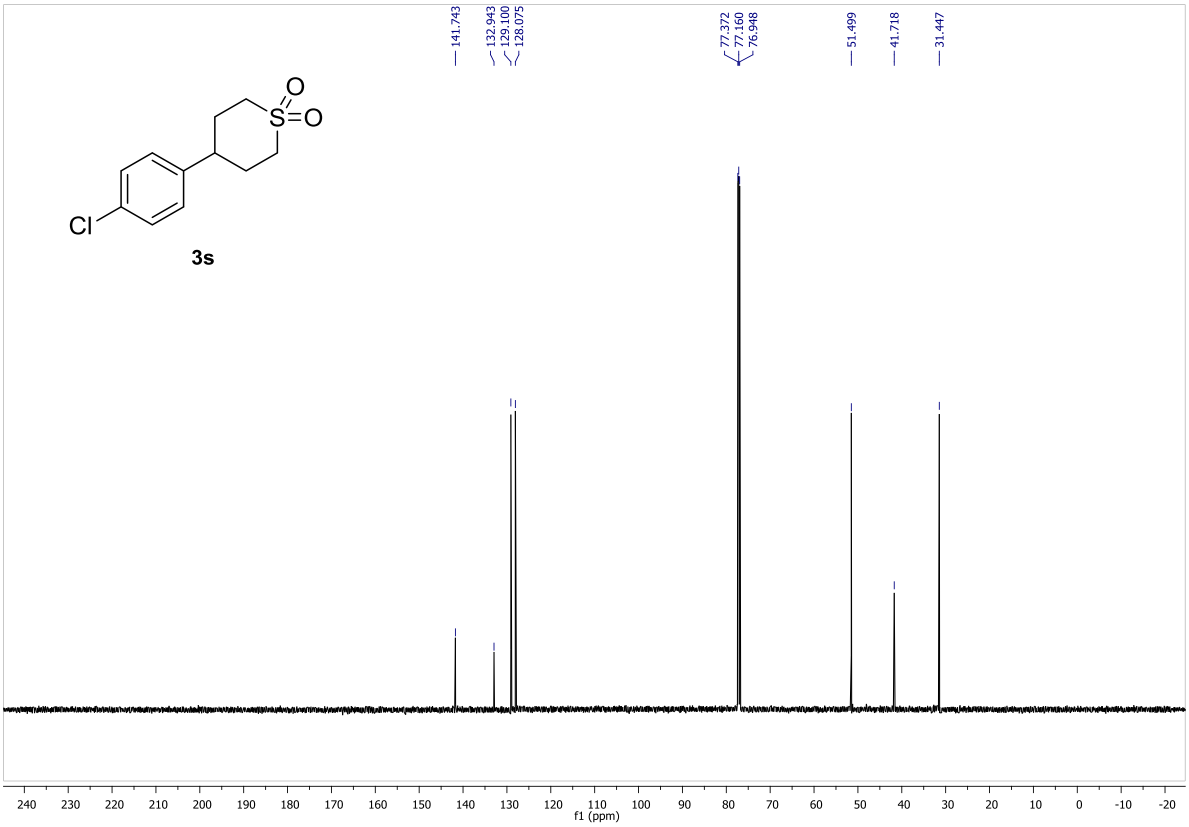

6-(4-chlorophenyl)-2,2-dimethyl-1-oxaspiro[2.5]octane (3t):

Diastereomer A:

^{1}H NMR, 600 MHz, $CDCl_3$:

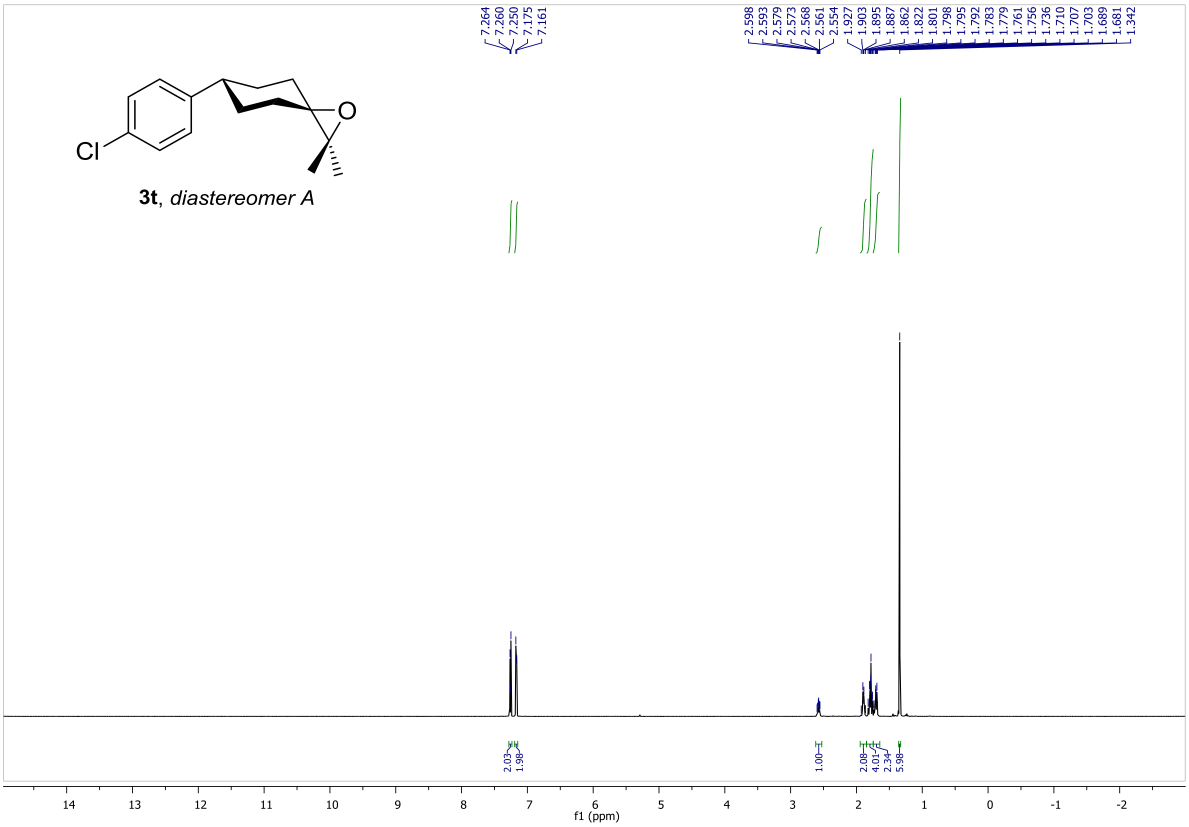

^{13}C NMR, 150 MHz, $CDCl_3$:

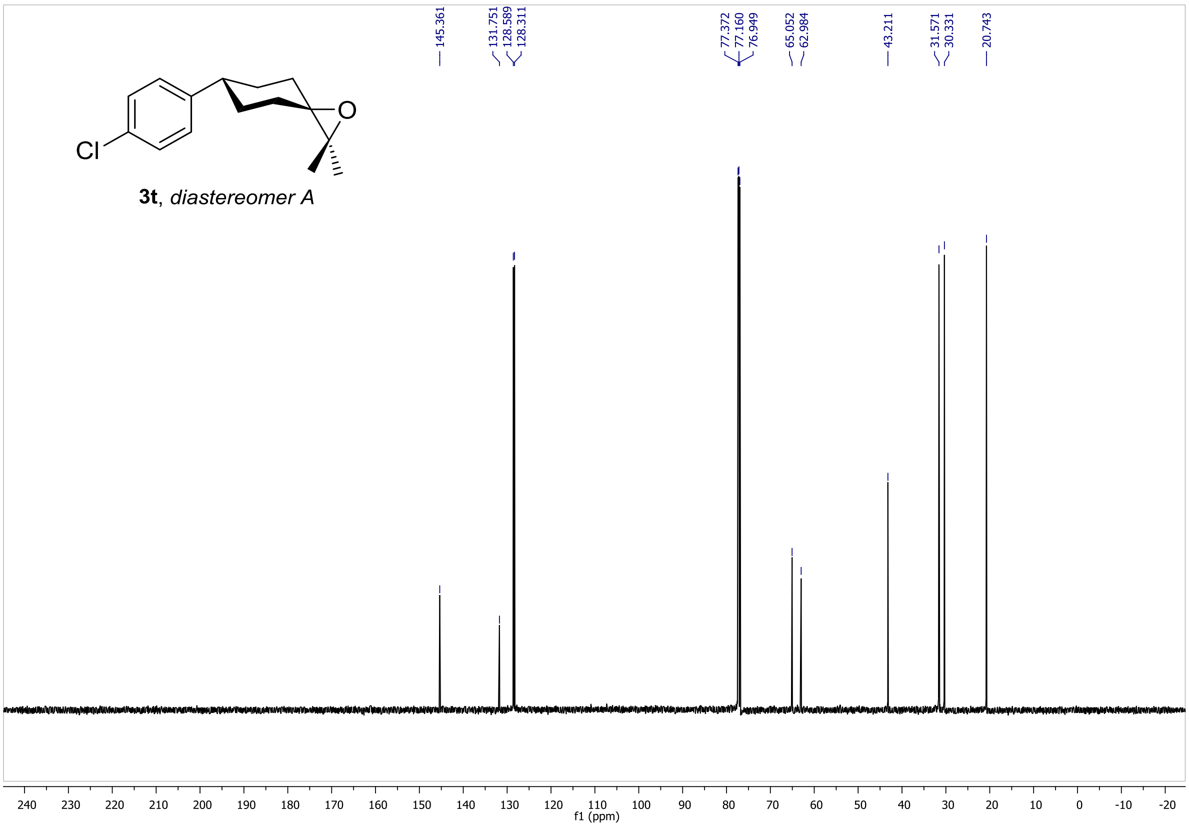

6-(4-chlorophenyl)-2,2-dimethyl-1-oxaspiro[2.5]octane (3t):

Diastereomer B:

^{1}H NMR, 600 MHz, $CDCl_3$:

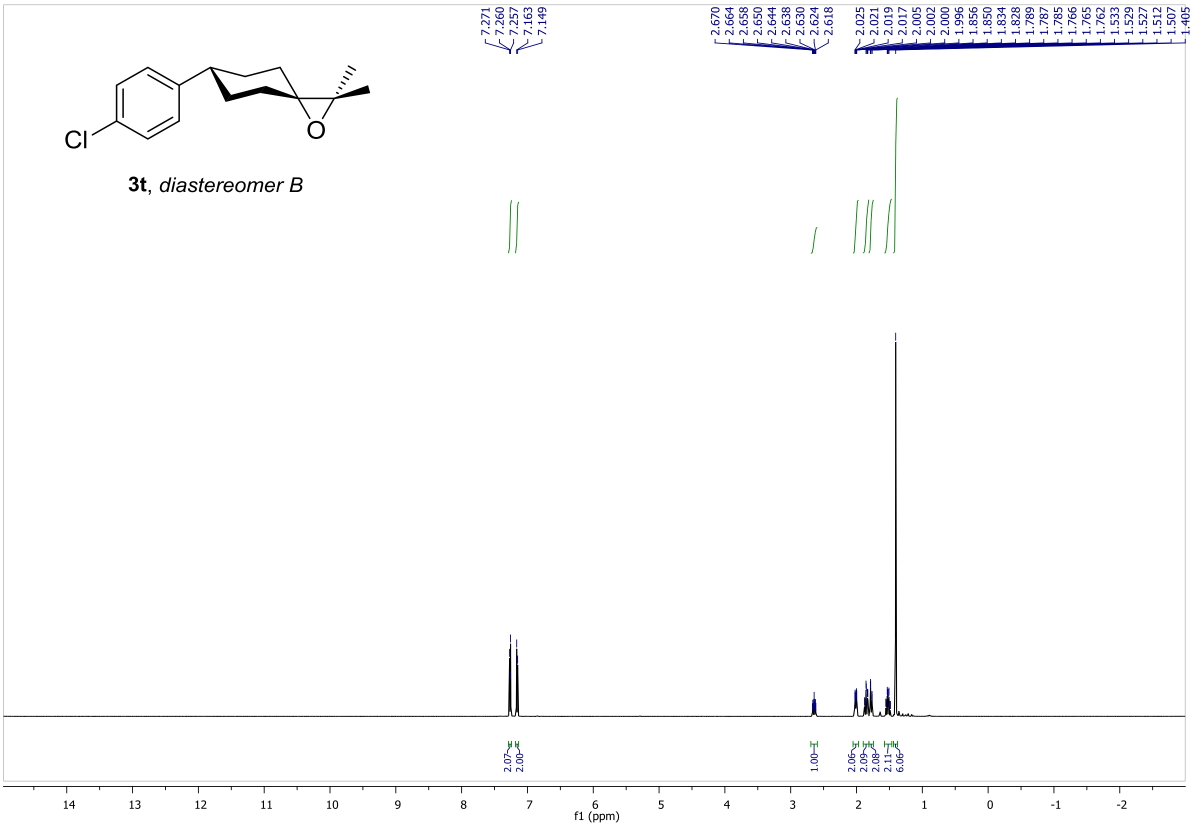

^{13}C NMR, 150 MHz, $CDCl_3$:

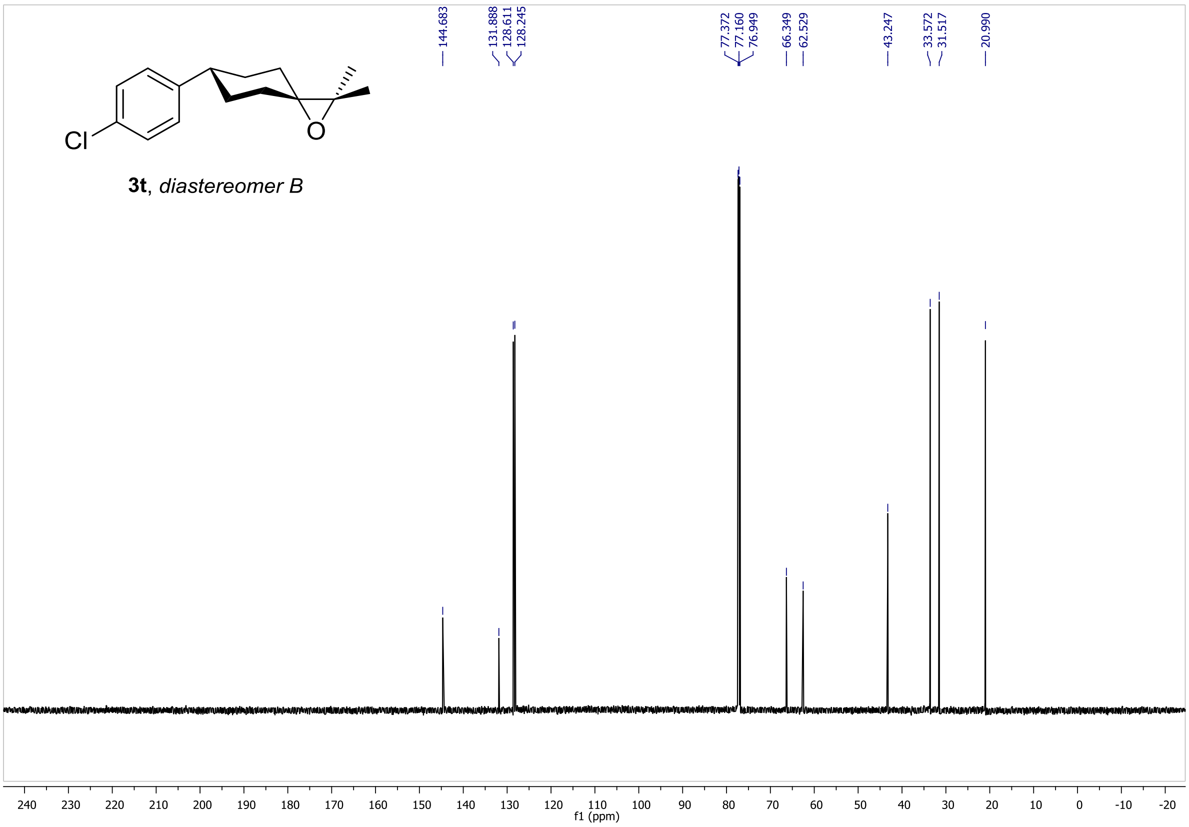

1-(5-bromopentan-2-yl)-4-chlorobenzene (3u):

^{1}H NMR, 600 MHz, $CDCl_3$:

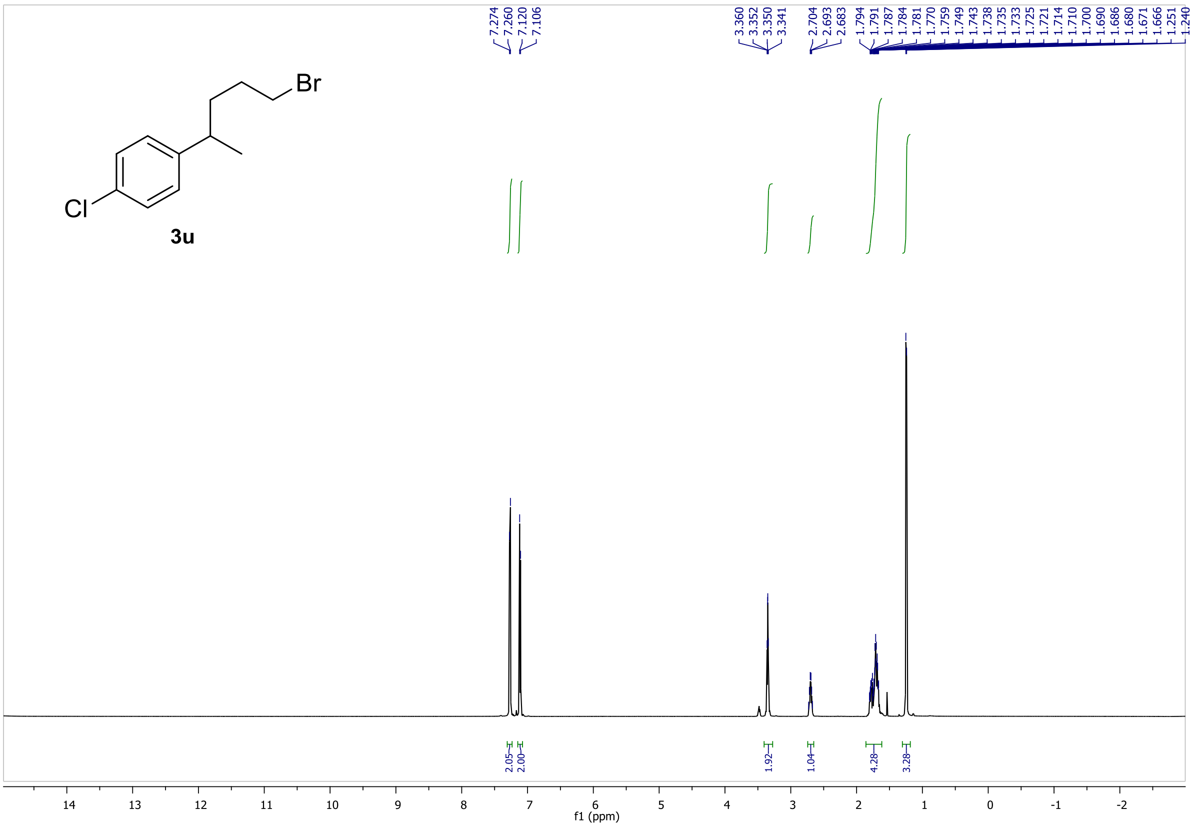

^{13}C NMR, 150 MHz, $CDCl_3$:

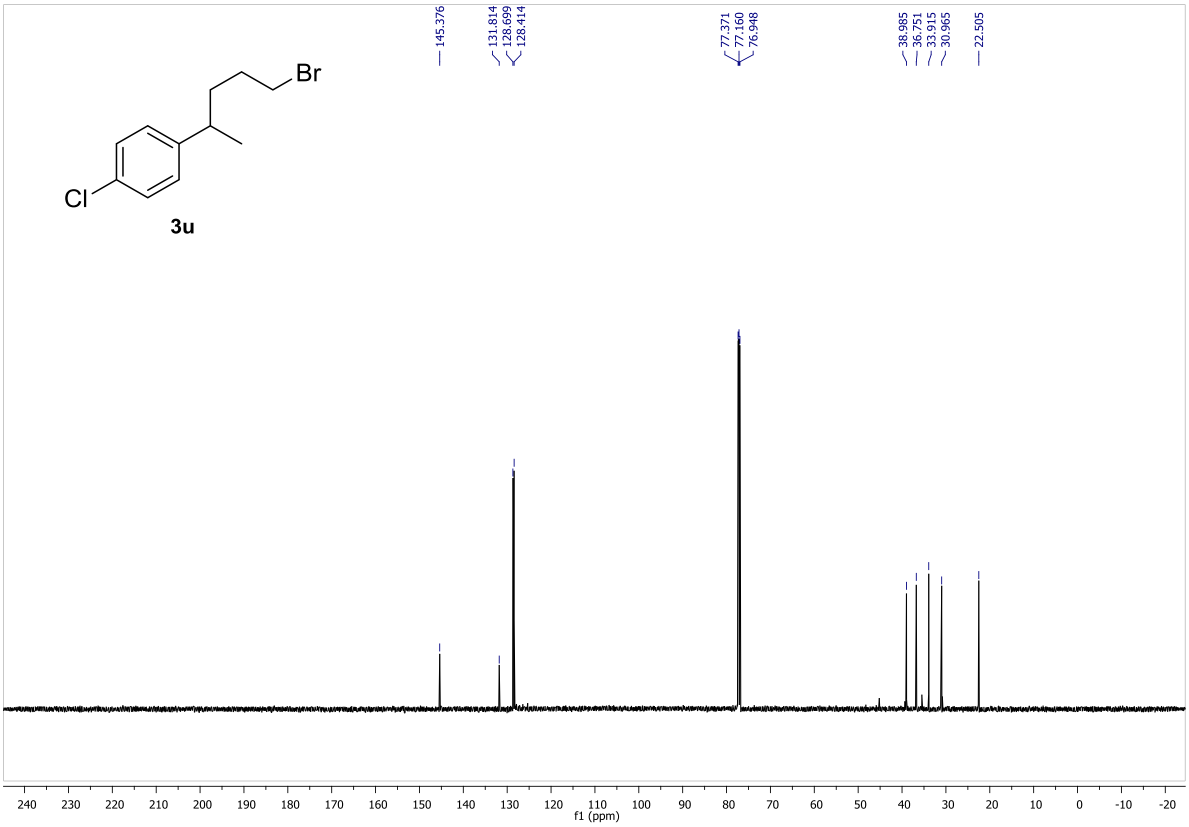

2-(2-(4-chlorophenyl)propyl)furan (3v):

^{1}H NMR, 600 MHz, $CDCl_3$:

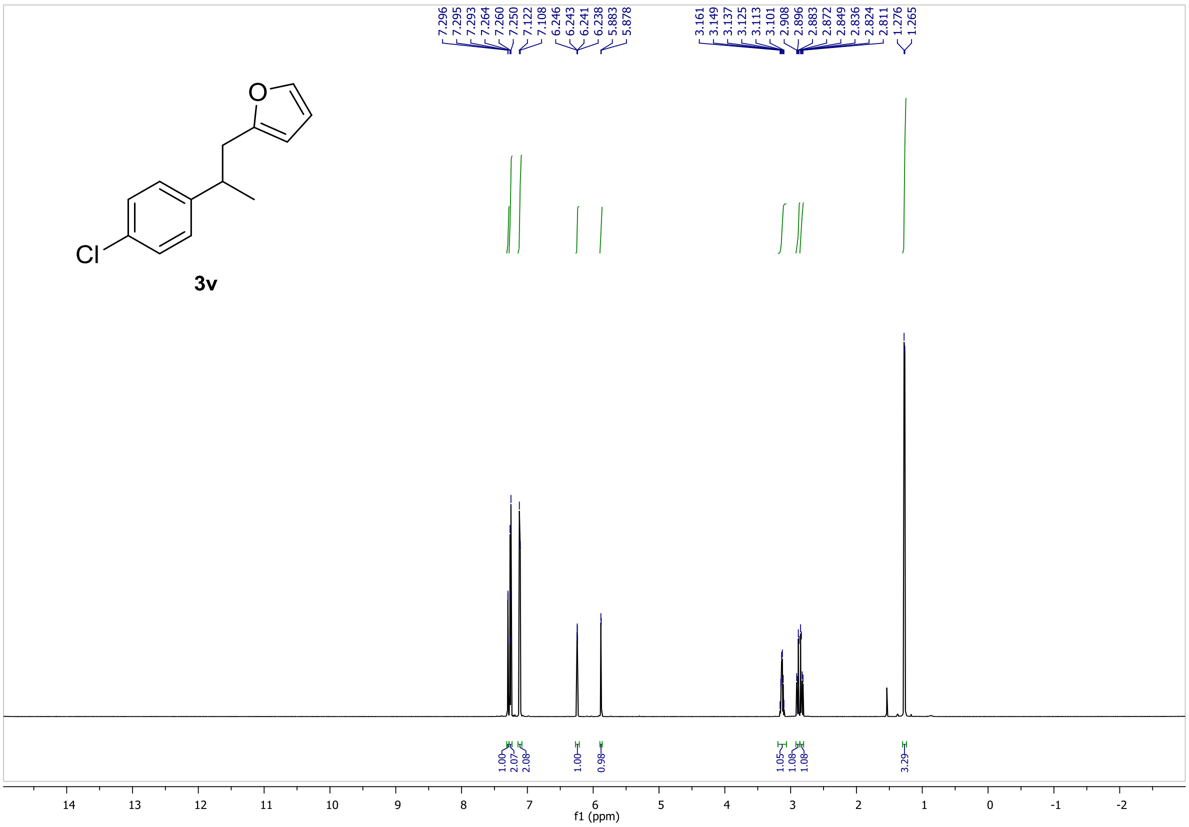

^{13}C NMR, 150 MHz, $CDCl_3$:

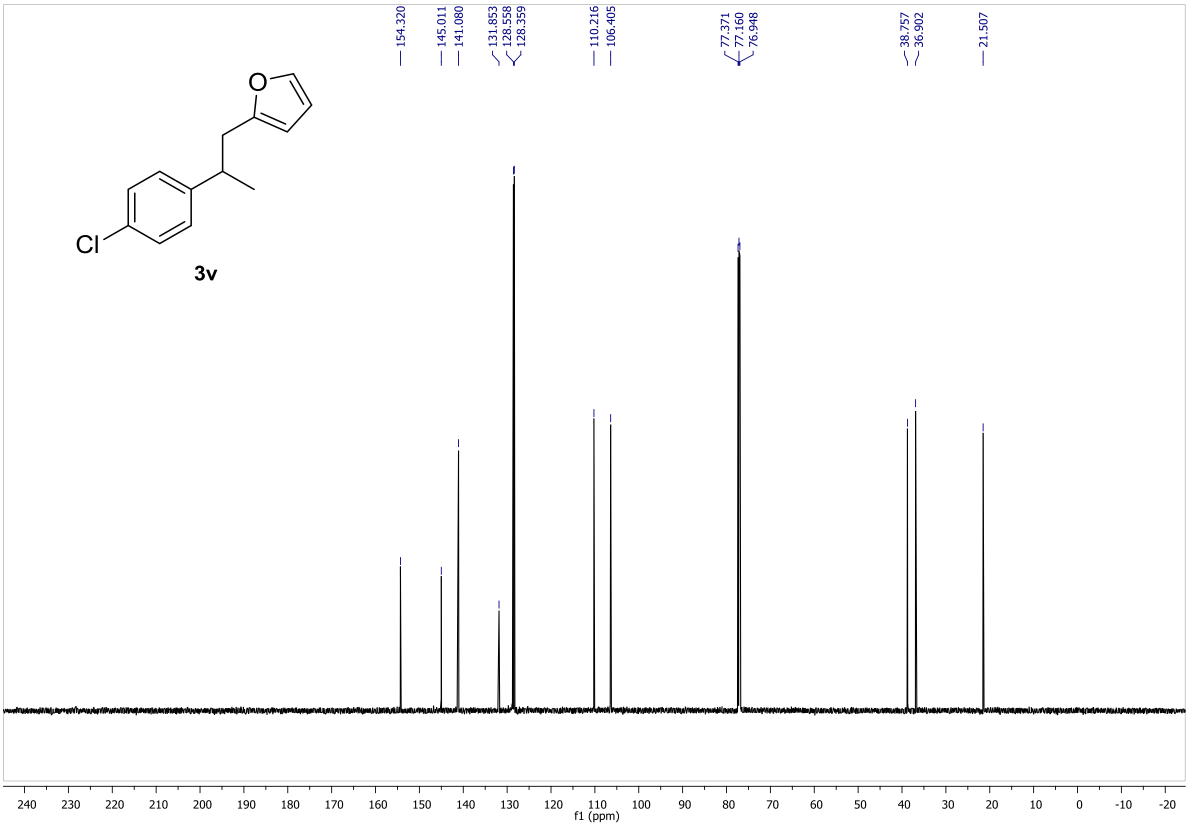

2-(4-(4-chlorophenyl)piperidin-1-yl)pyrimidine (3w):

^{1}H NMR, 600 MHz, $CDCl_3$:

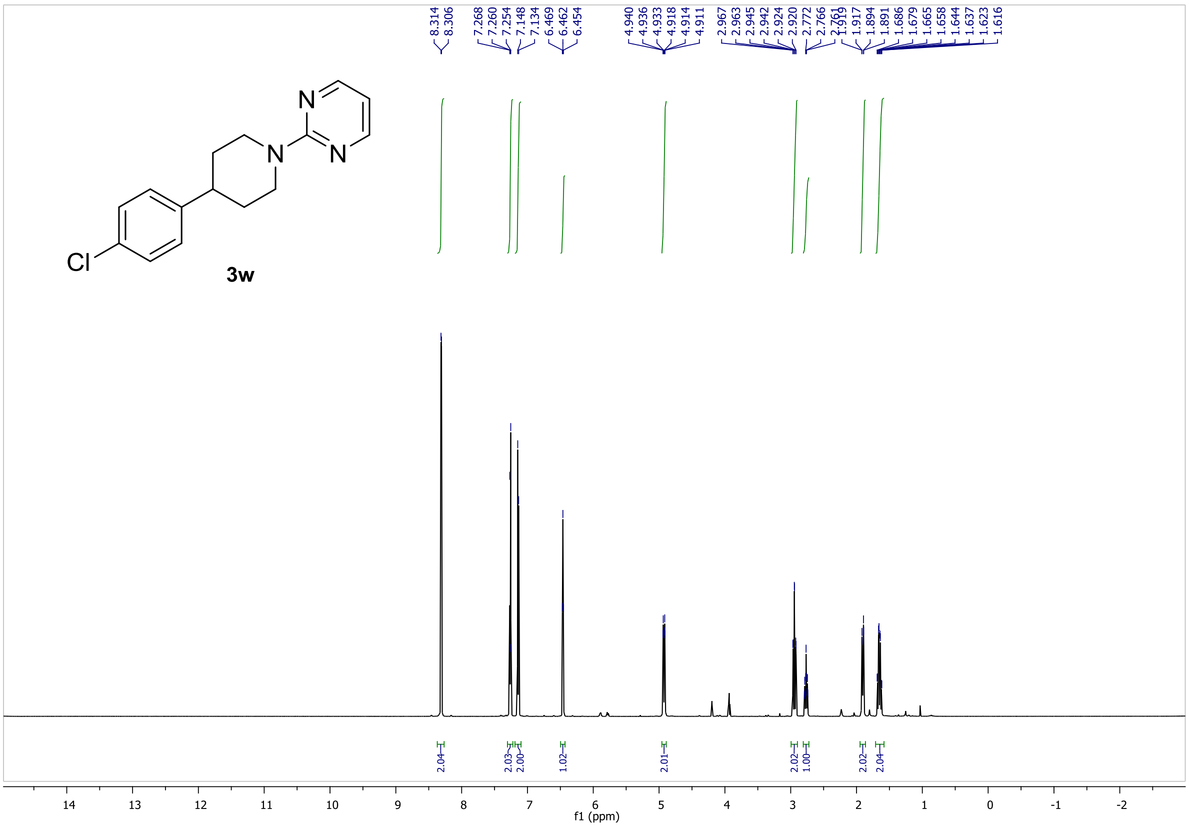

^{13}C NMR, 150 MHz, $CDCl_3$:

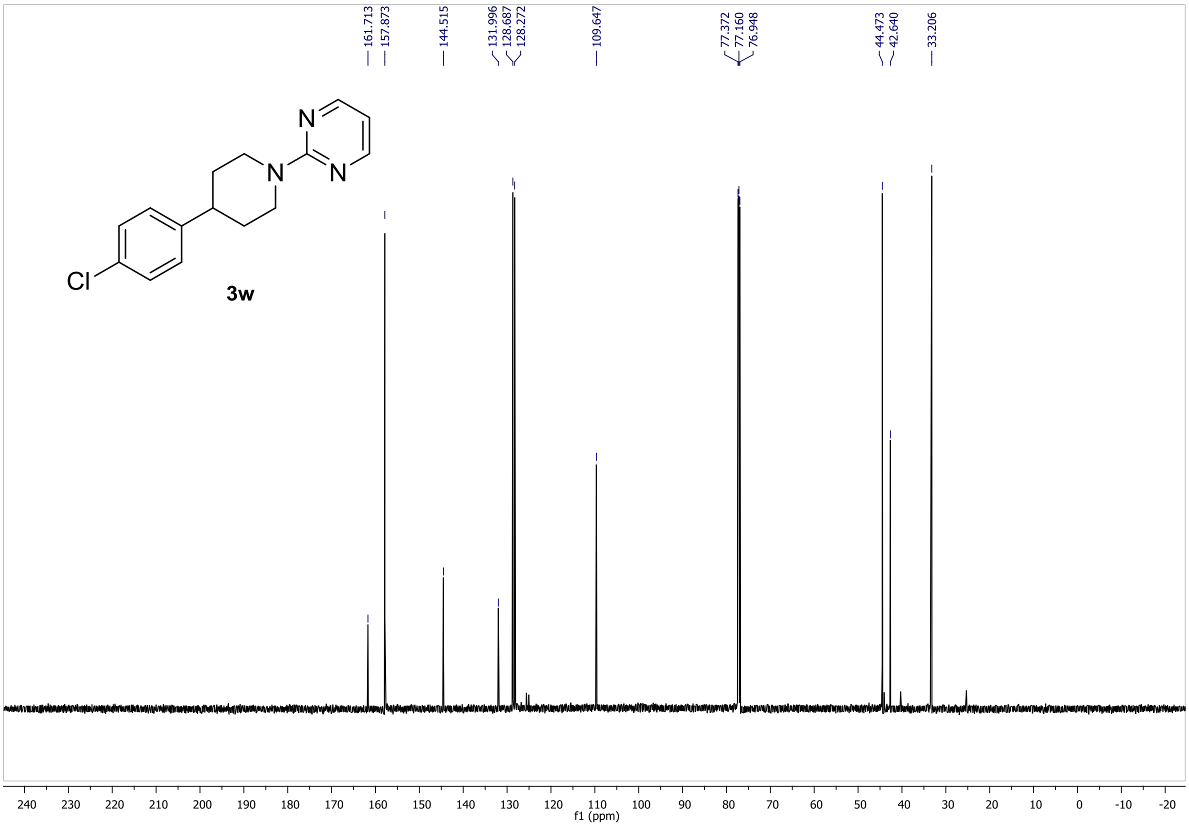

4-(4-bromophenyl)tetrahydro-2*H*-pyran (3x):

^{1}H NMR, 600 MHz, $CDCl_3$:

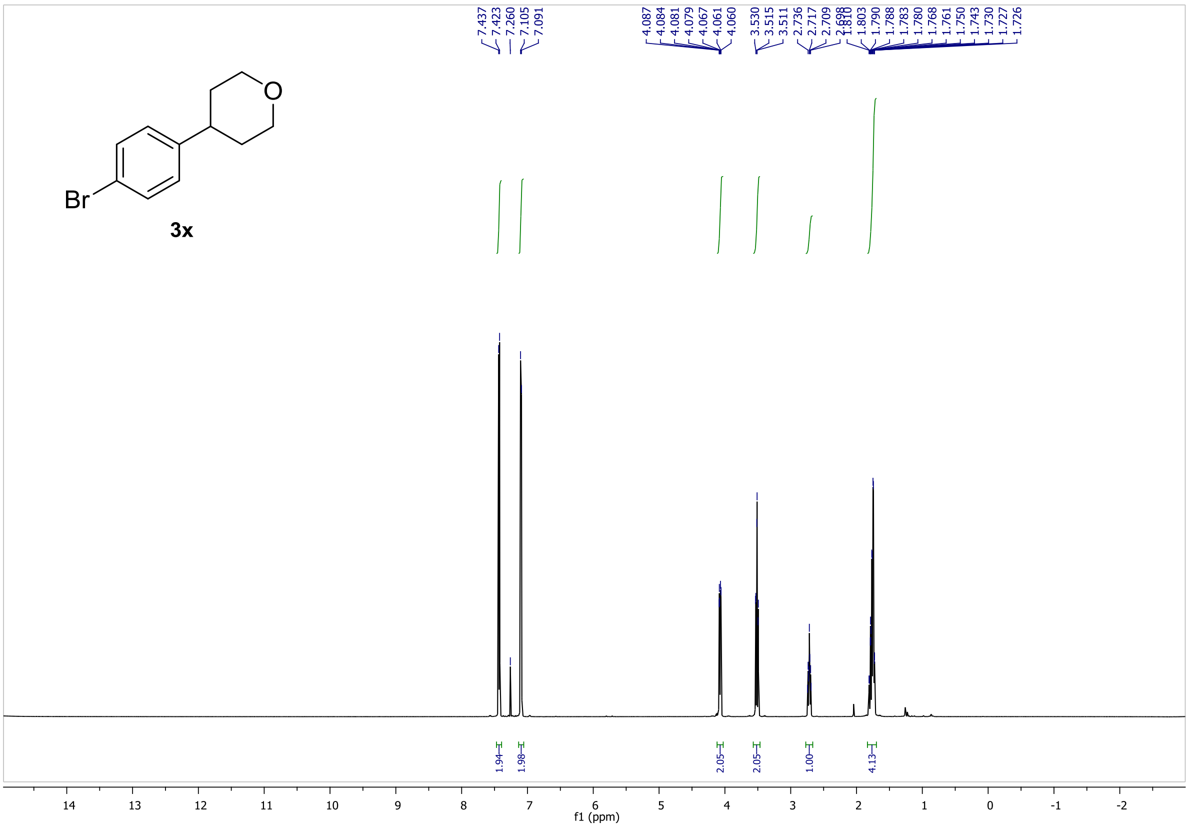

^{13}C NMR, 150 MHz, $CDCl_3$:

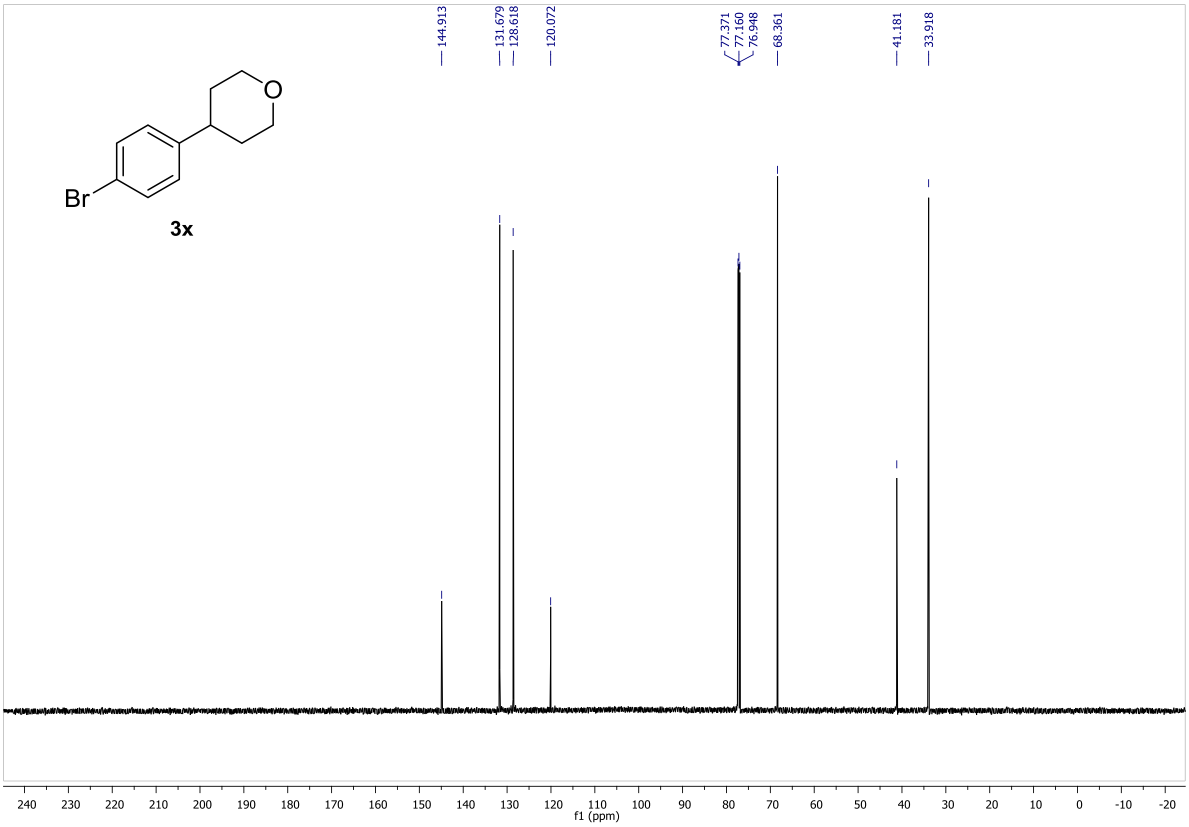

4-(4-(trifluoromethyl)phenyl)tetrahydro-2*H*-pyran (3y):

^{1}H NMR, 600 MHz, $CDCl_3$:

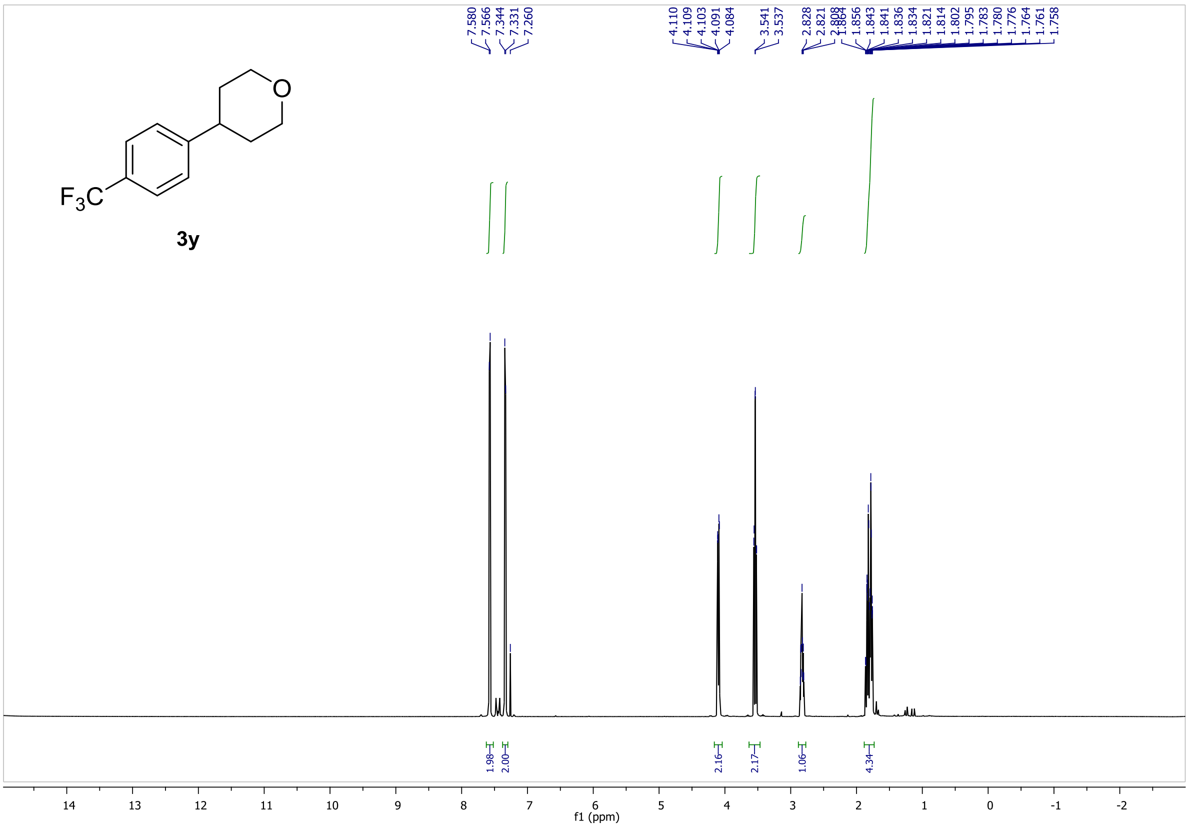

^{13}C NMR, 150 MHz, $CDCl_3$:

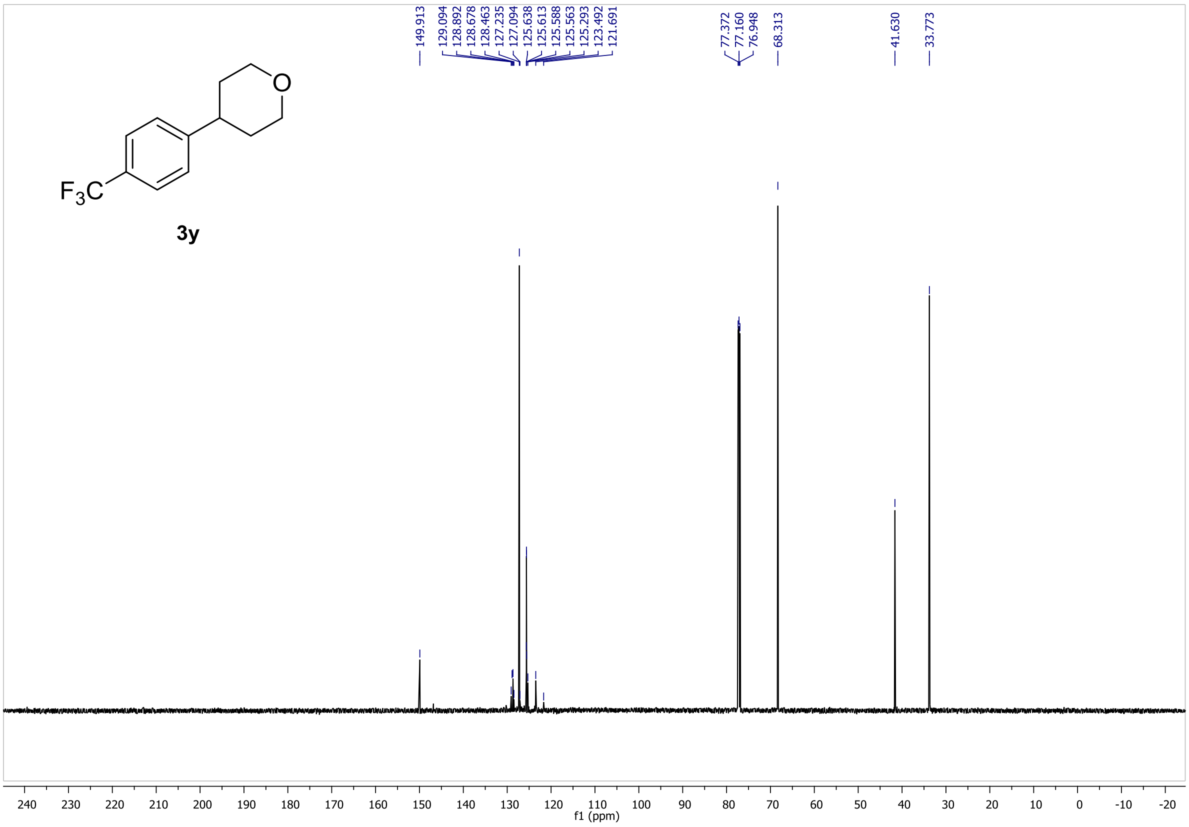

^{19}F NMR, 376 MHz, $CDCl_3$:

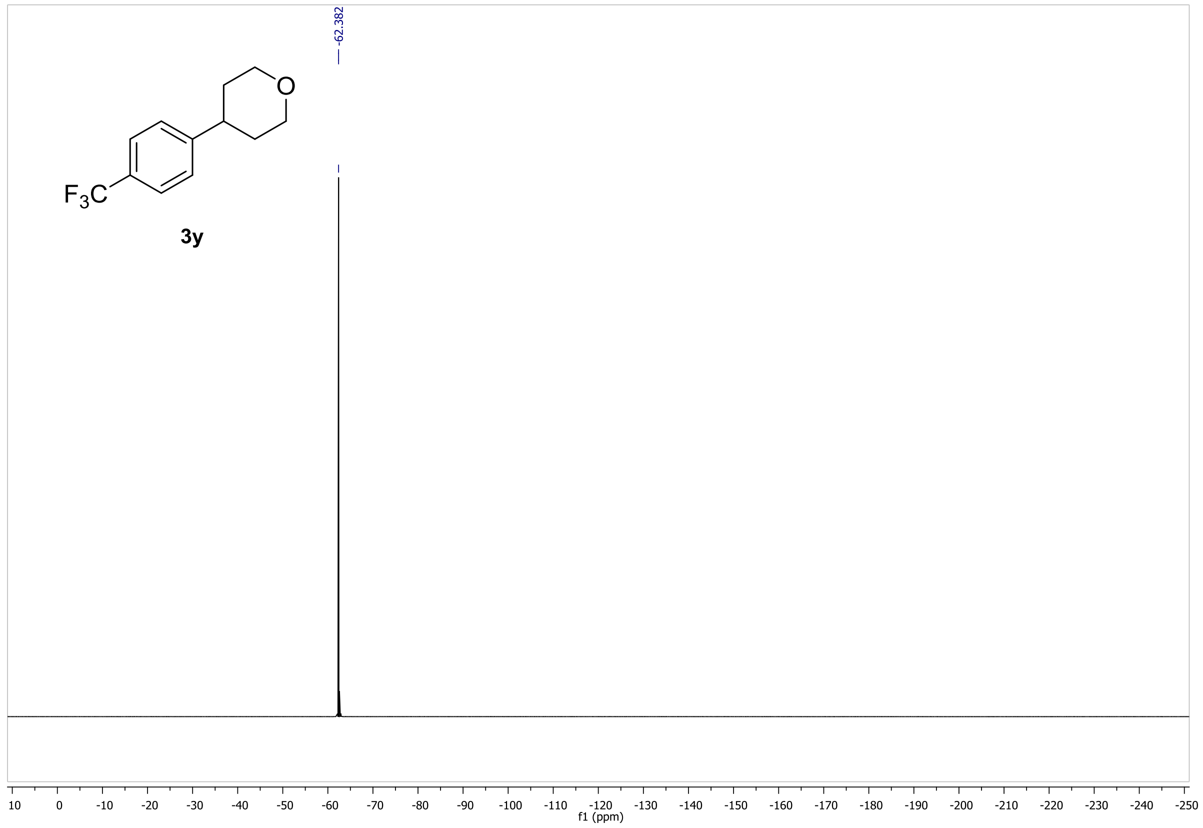

4-(tetrahydro-2*H*-pyran-4-yl)benzonitrile (3z):

^{1}H NMR, 600 MHz, $CDCl_3$:

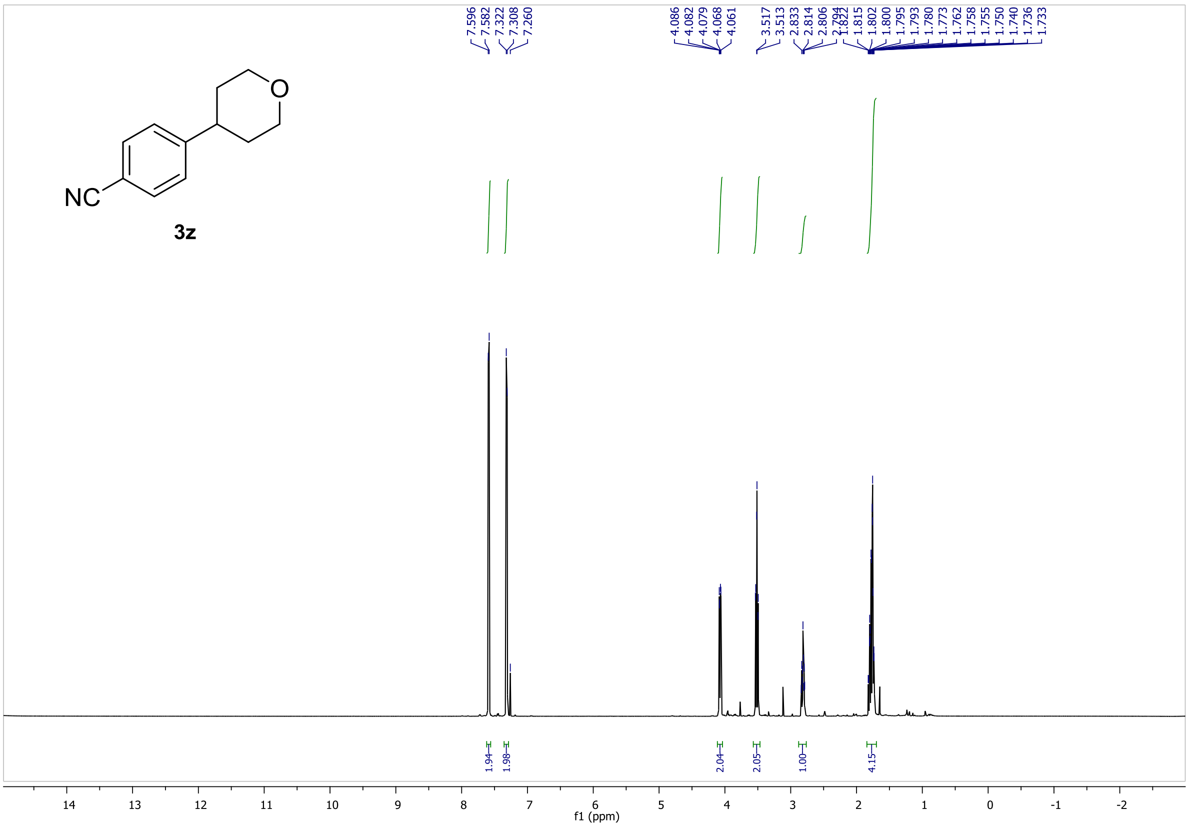

^{13}C NMR, 150 MHz, $CDCl_3$:

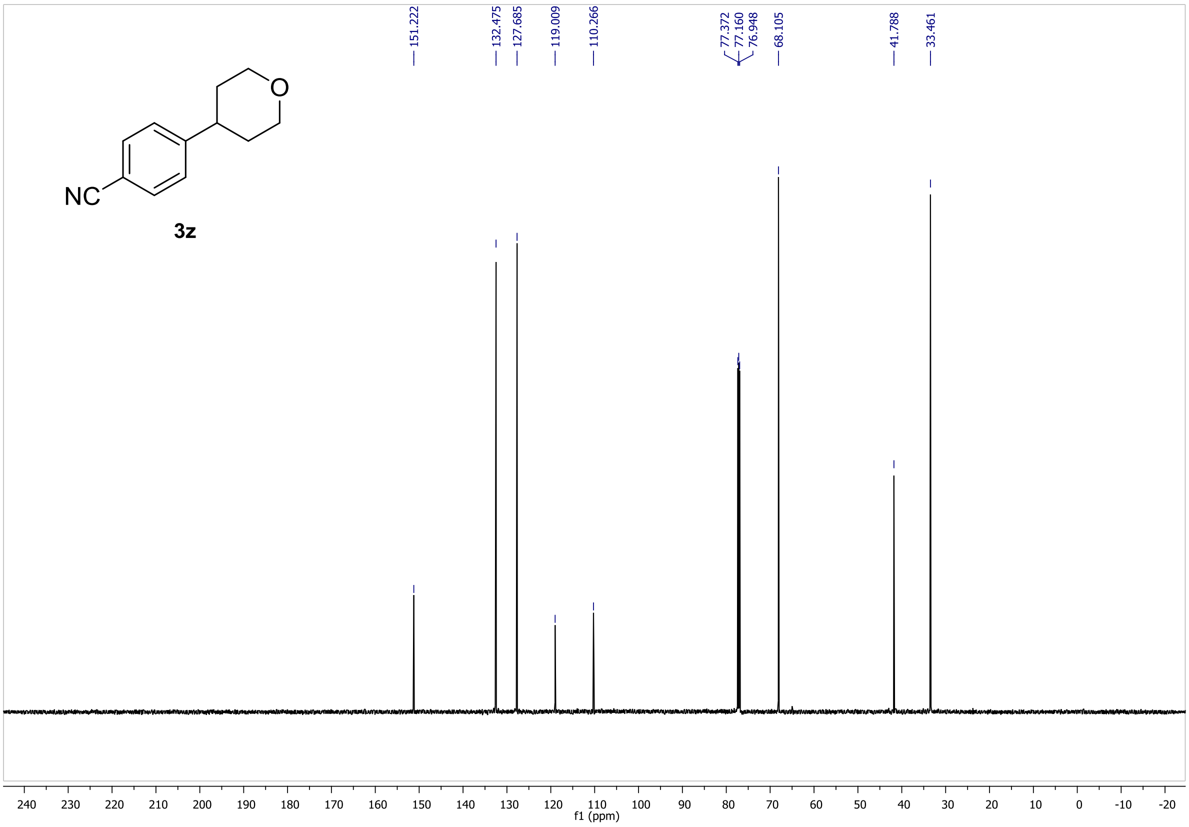

Methyl 4-(tetrahydro-2*H*-pyran-4-yl)benzoate (3aa):

^{1}H NMR, 600 MHz, $CDCl_3$:

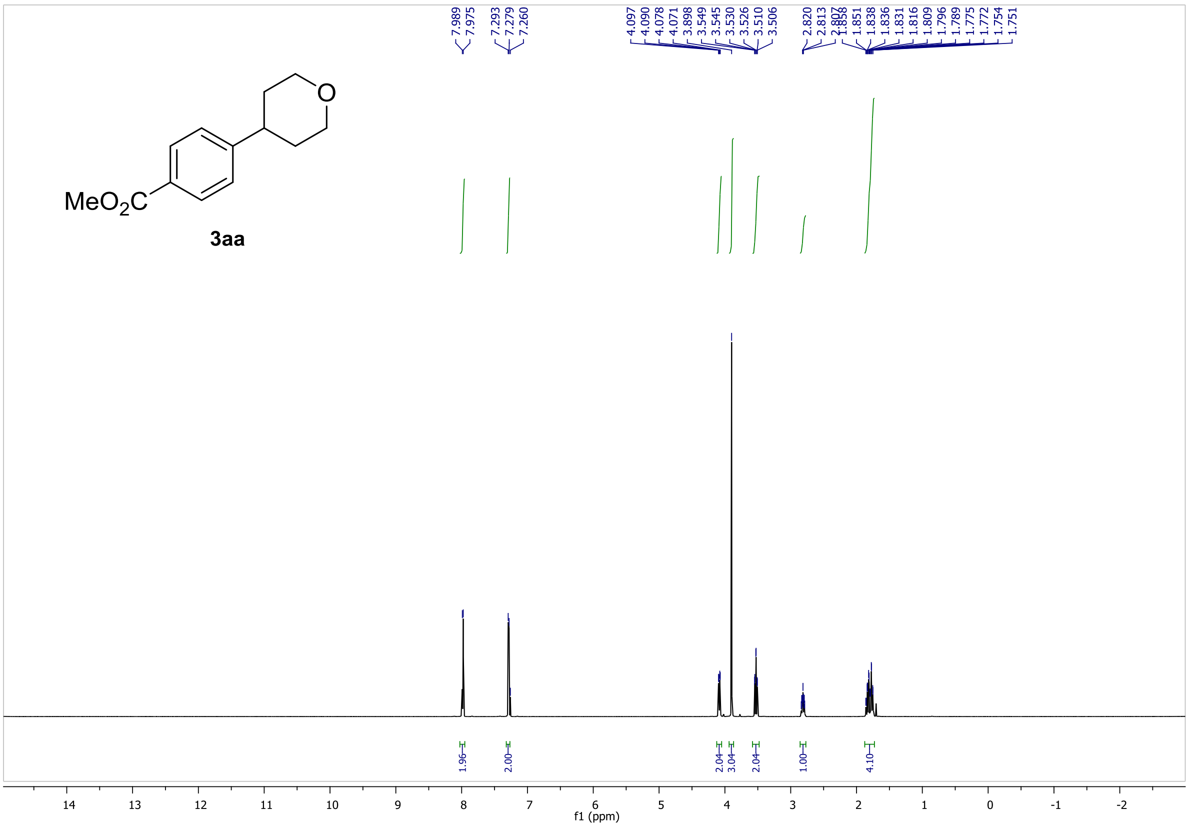

^{13}C NMR, 150 MHz, $CDCl_3$:

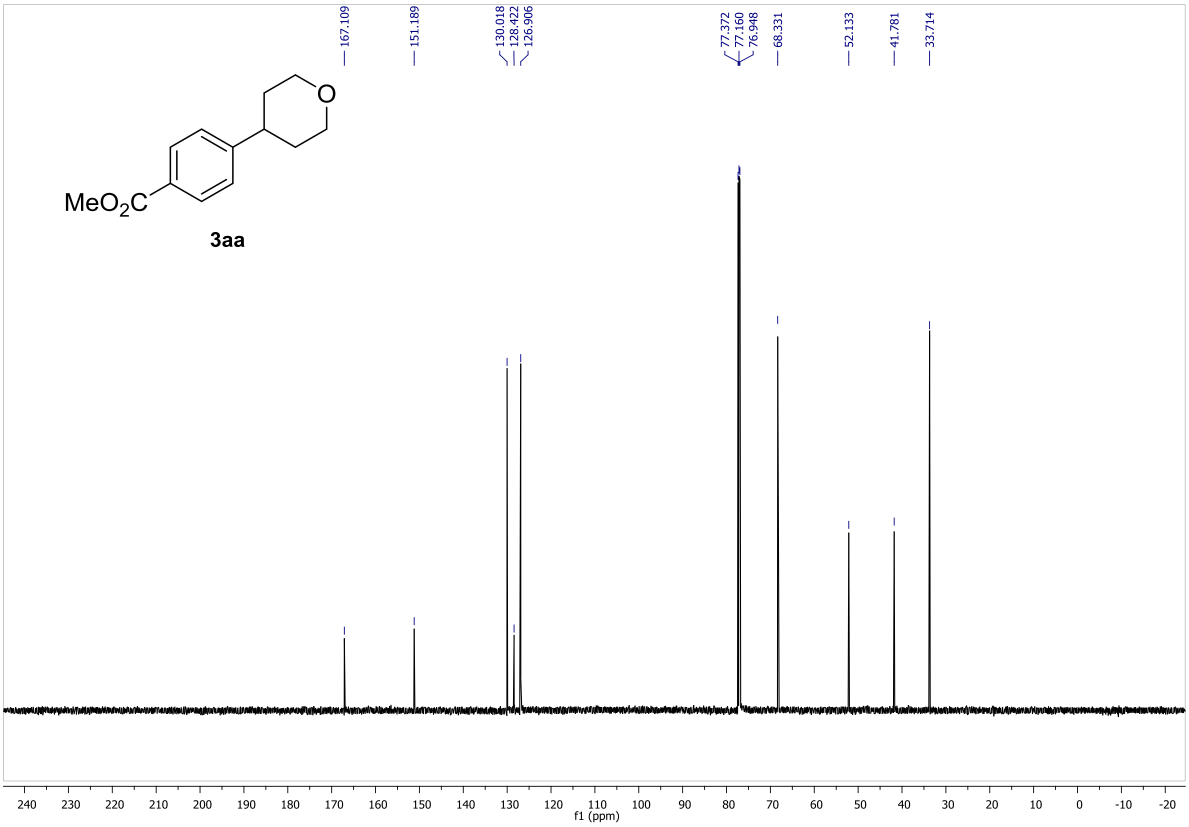

N-(3-(tetrahydro-2*H*-pyran-4-yl)phenyl)acetamide (3ab):

^{1}H NMR, 600 MHz, CDCl$_3$:

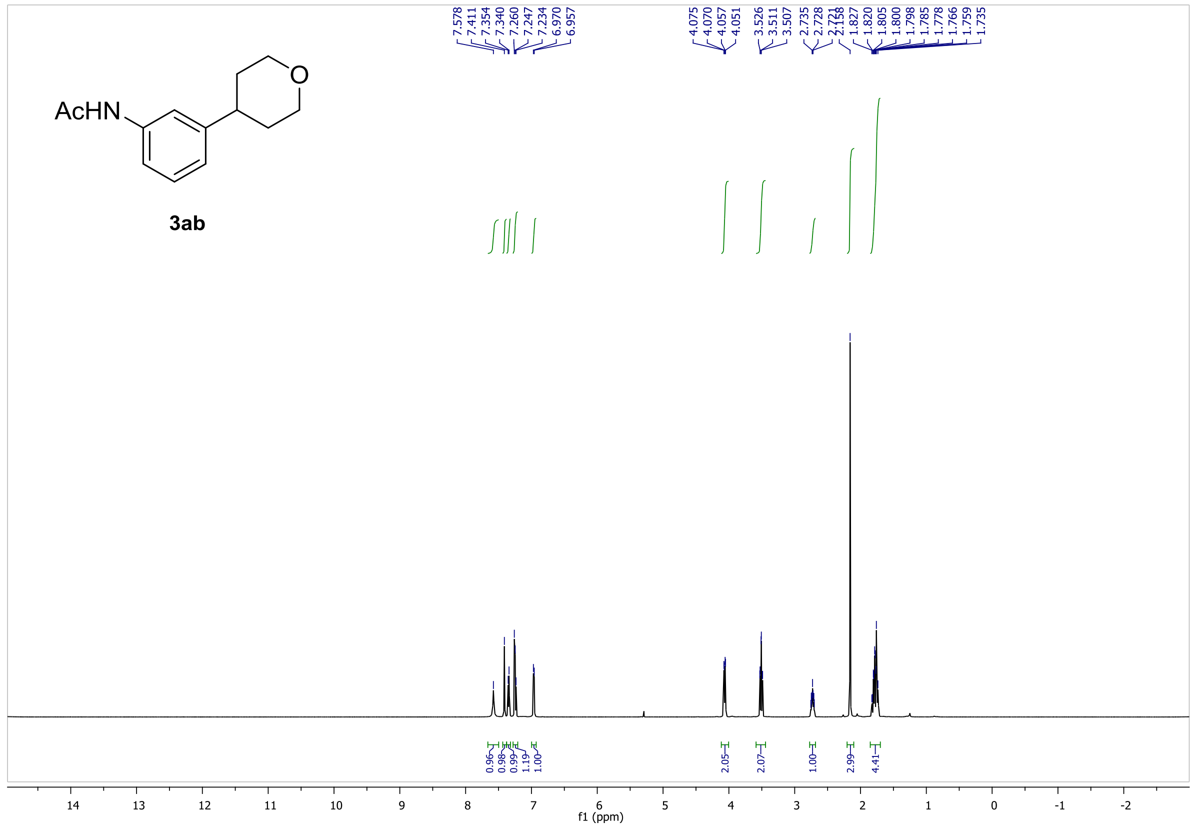

^{13}C NMR, 150 MHz, CDCl$_3$:

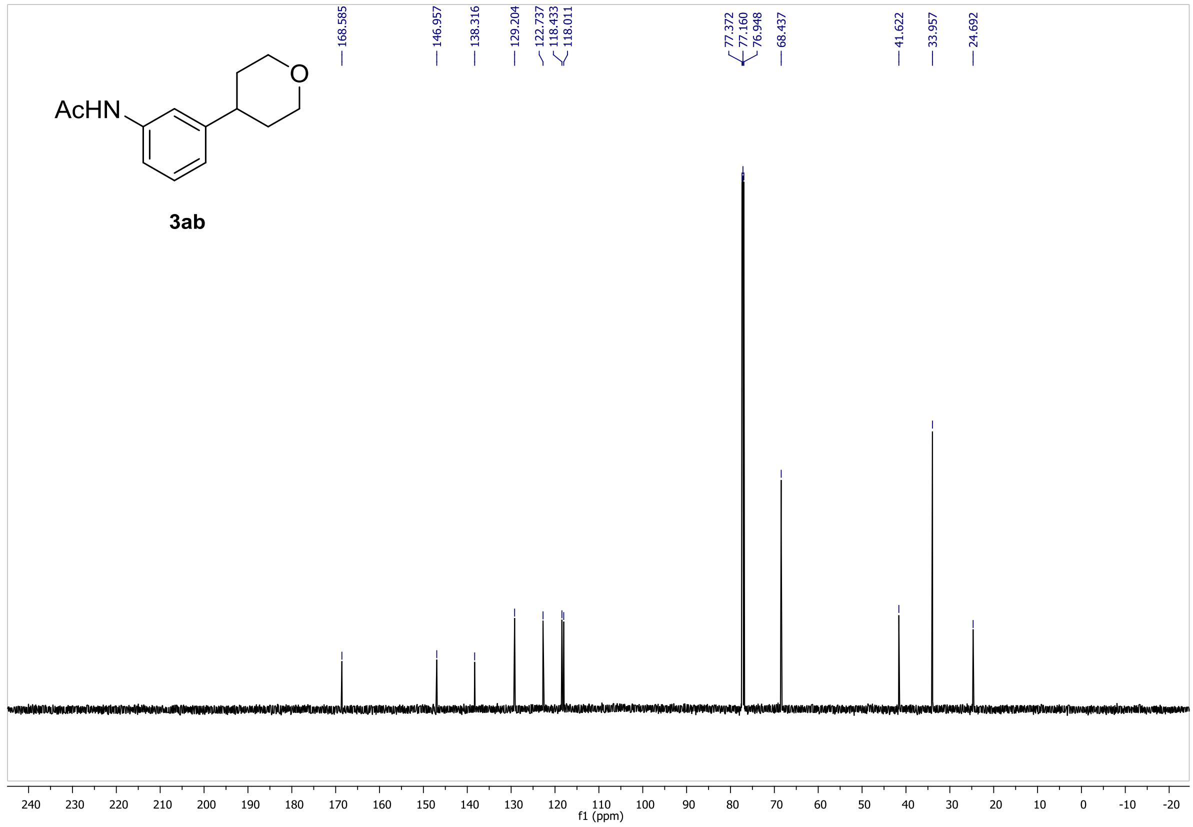

1-(1-cyclopropylethyl)-4-methoxybenzene (3ac):

^{1}H NMR, 600 MHz, $CDCl_3$:

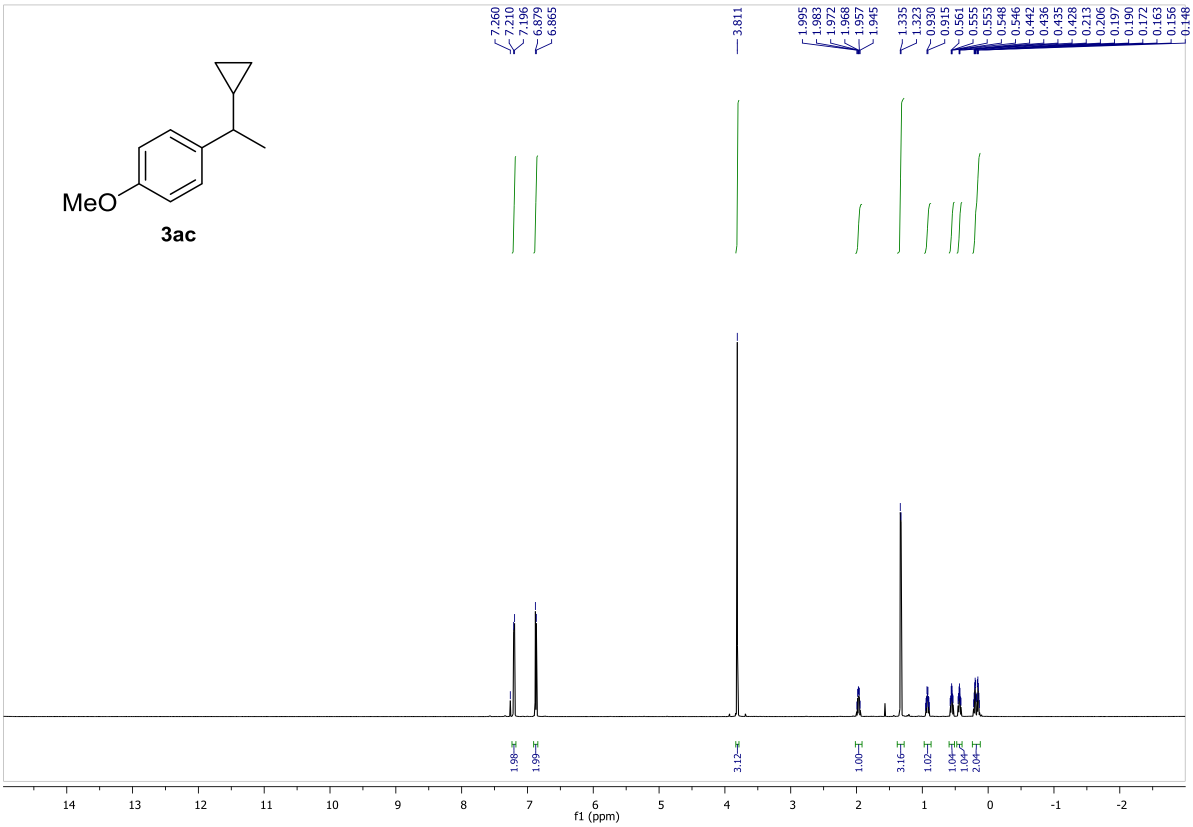

^{13}C NMR, 150 MHz, $CDCl_3$:

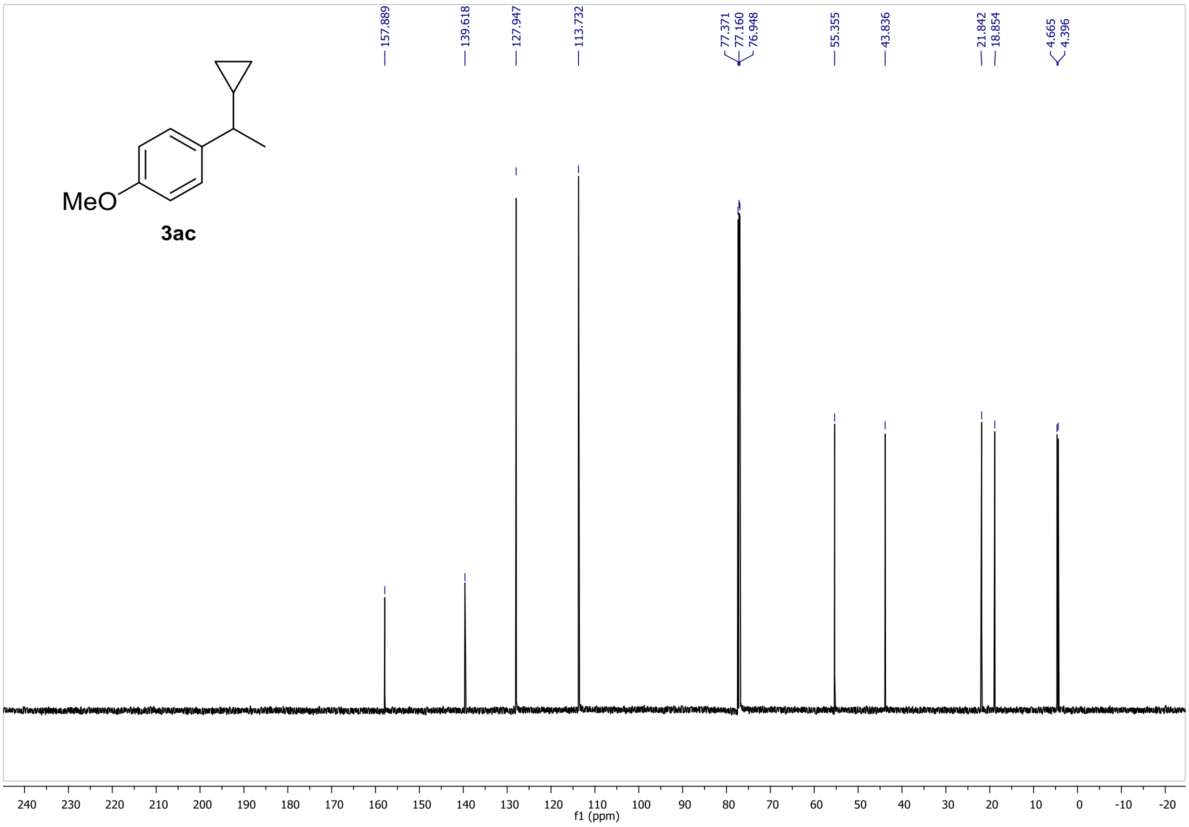

4-(*o*-tolyl)tetrahydro-2*H*-pyran (3ad):

^{1}H NMR, 600 MHz, CDCl$_3$:

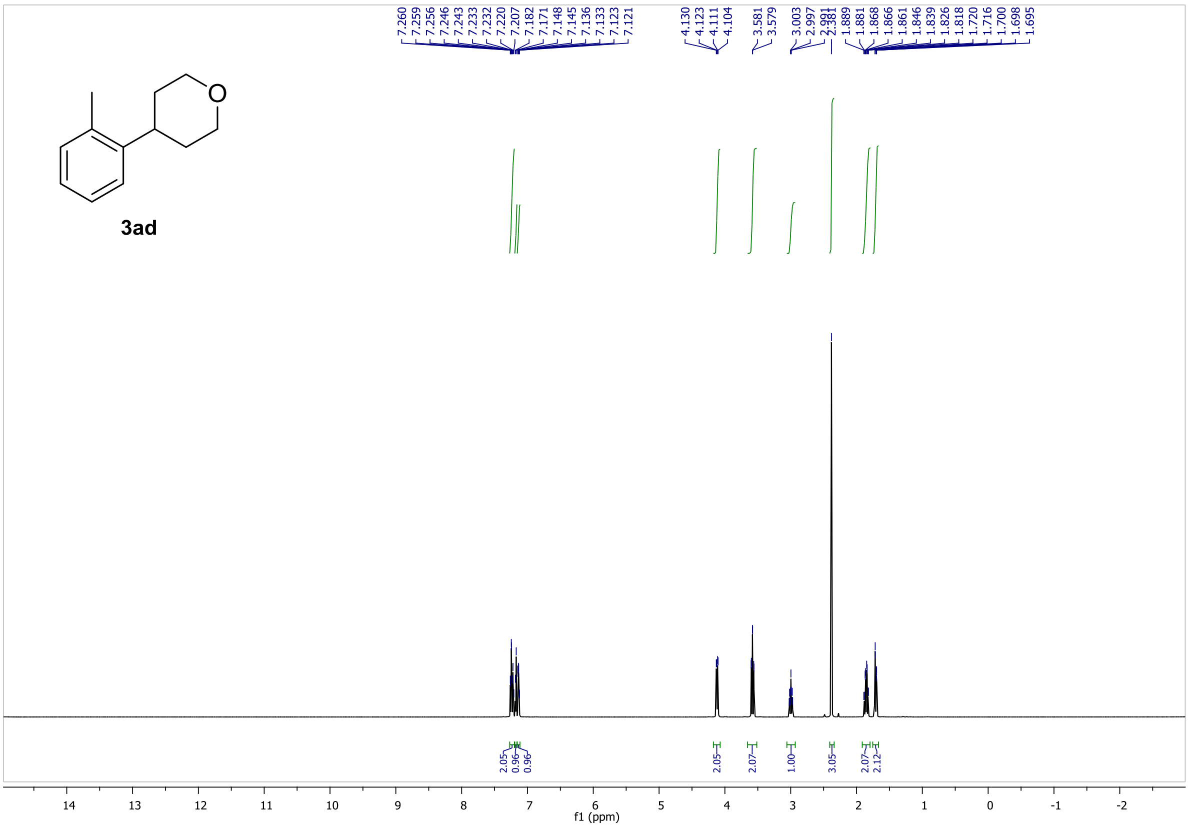

^{13}C NMR, 150 MHz, CDCl$_3$:

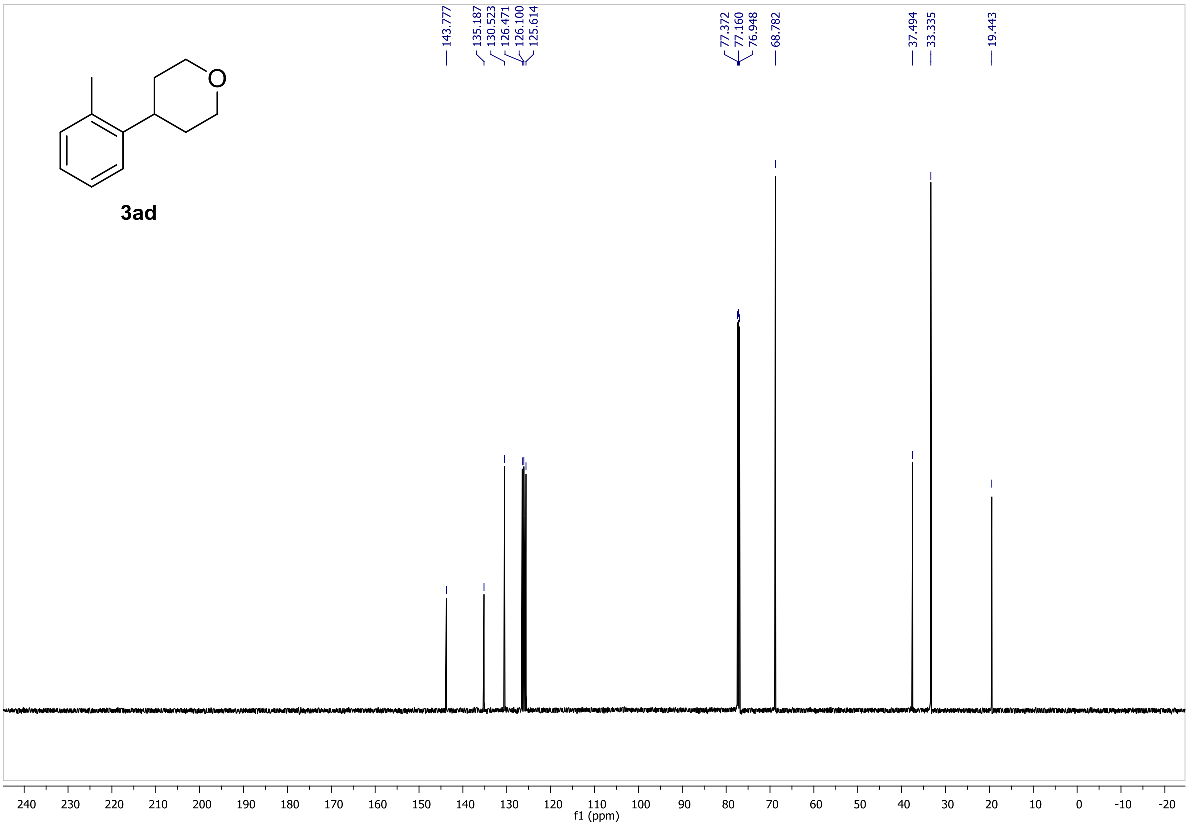

3-(tetrahydro-2*H*-pyran-4-yl)pyridine (3ae):

^{1}H NMR, 600 MHz, $CDCl_3$:

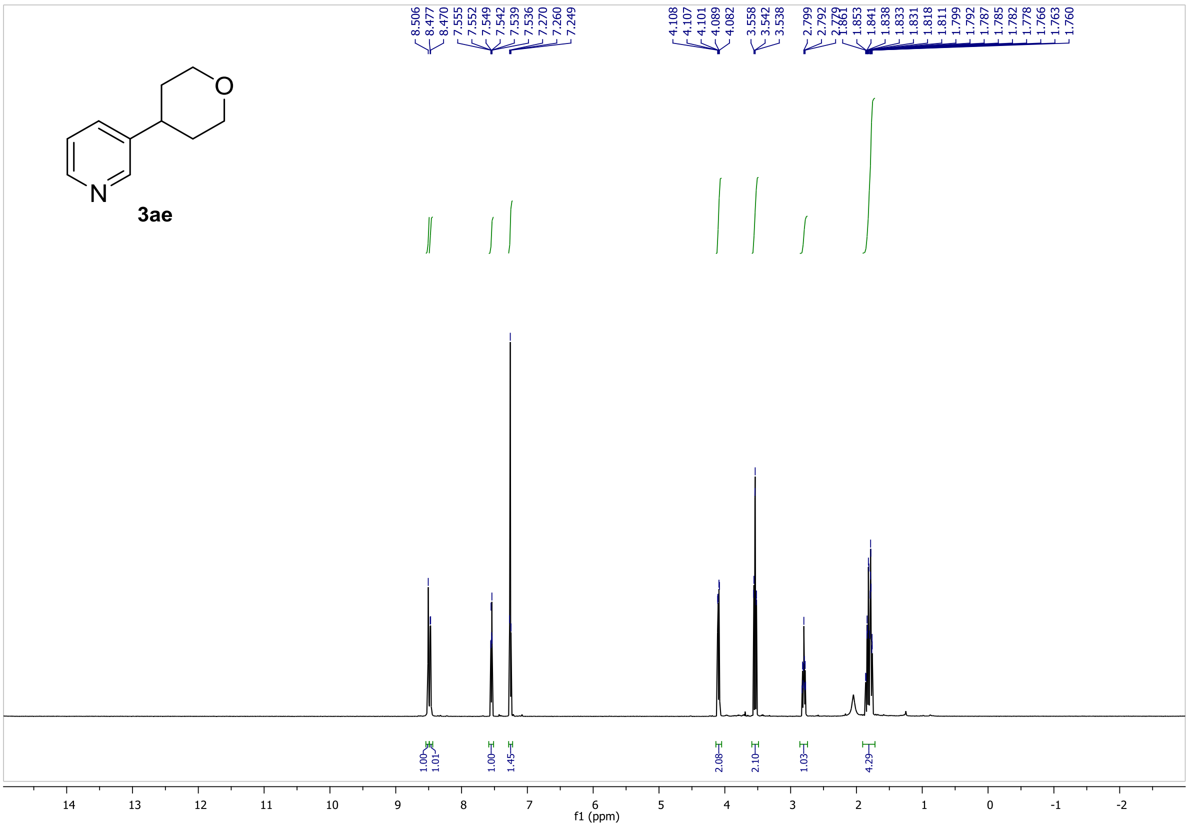

^{13}C NMR, 150 MHz, $CDCl_3$:

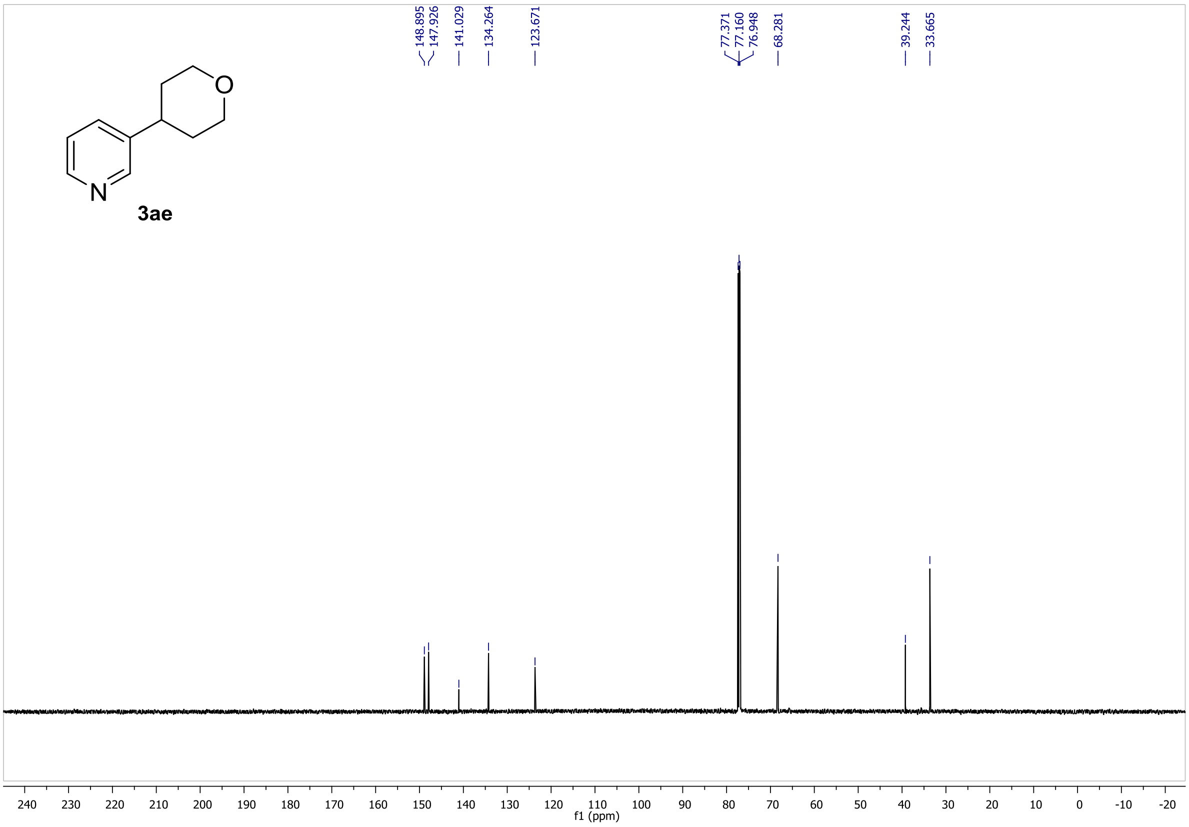

4-(thiophen-2-yl)tetrahydro-2*H*-pyran (3af):

^{1}H NMR, 600 MHz, $CDCl_3$:

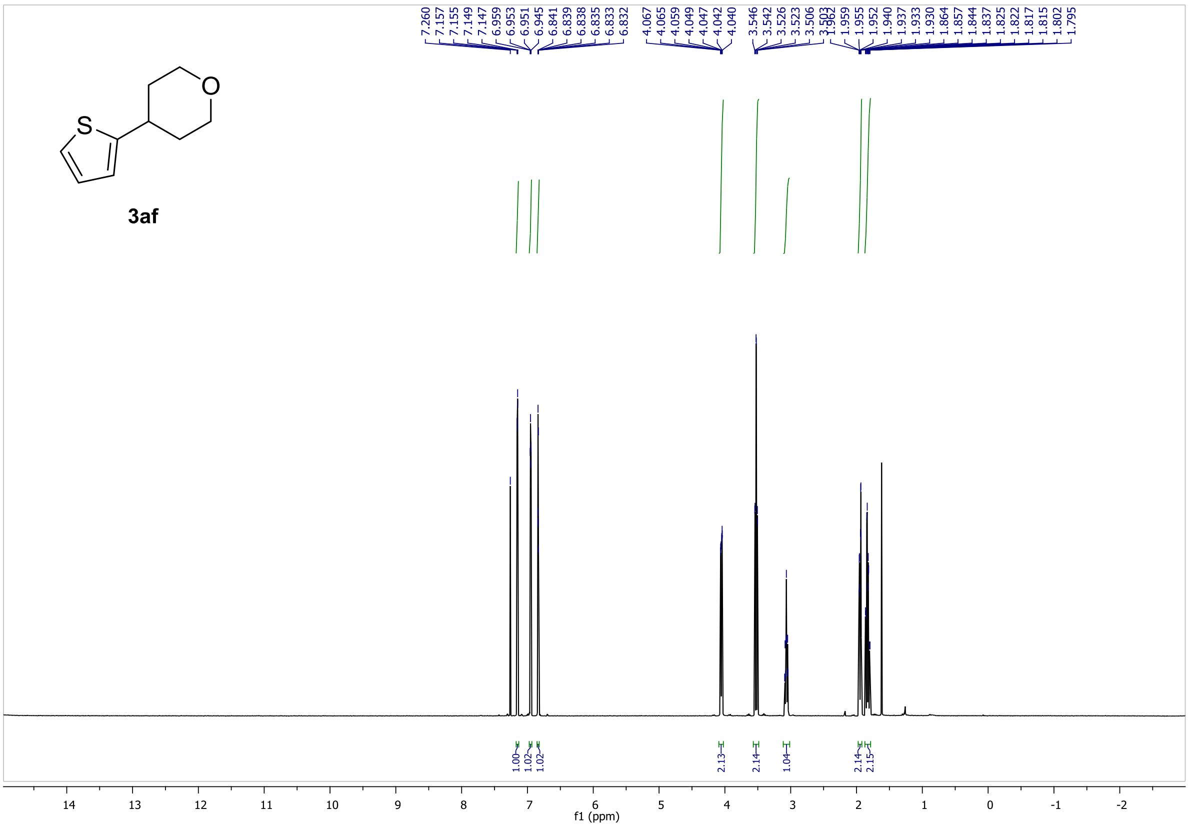

^{13}C NMR, 150 MHz, $CDCl_3$:

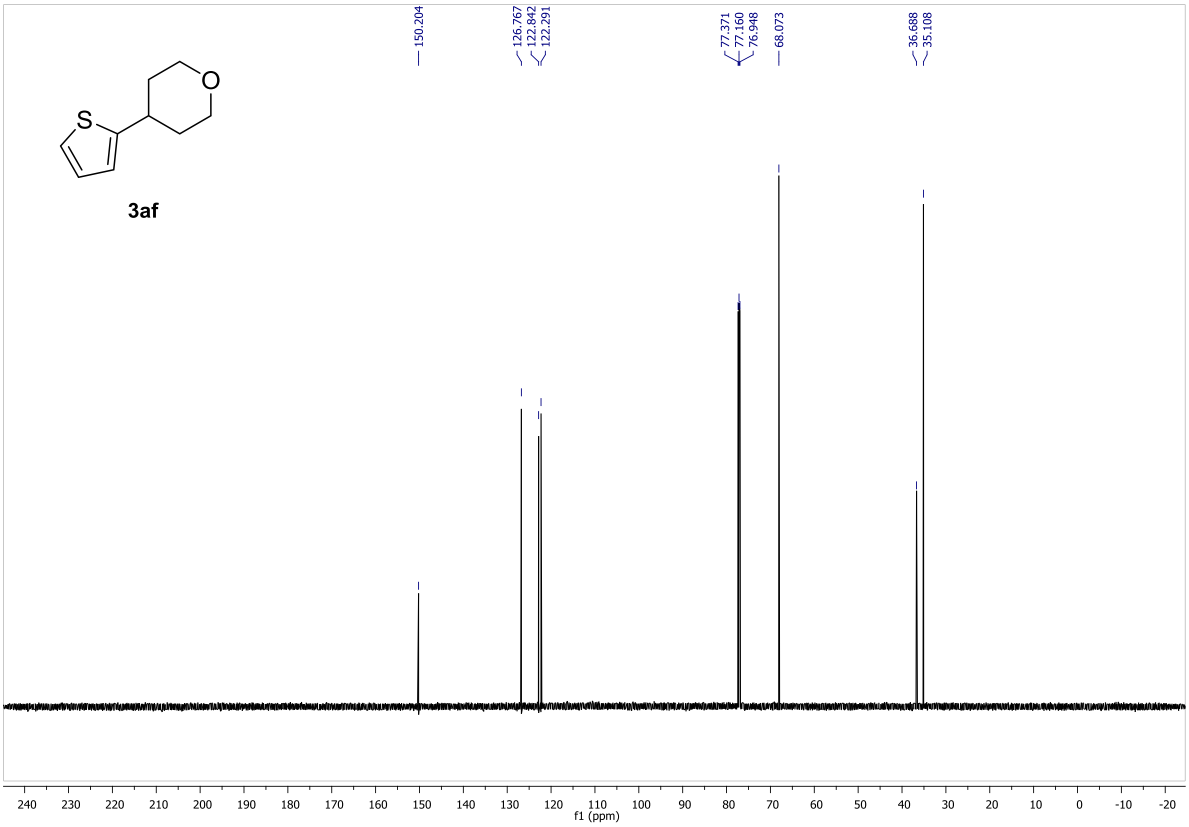

8.3. NMR spectra for oxidative coupling products

4-(4-chlorophenyl)tetrahydro-2*H*-pyran-4-ol (4a):

^{1}H NMR, 600 MHz, $CDCl_3$:

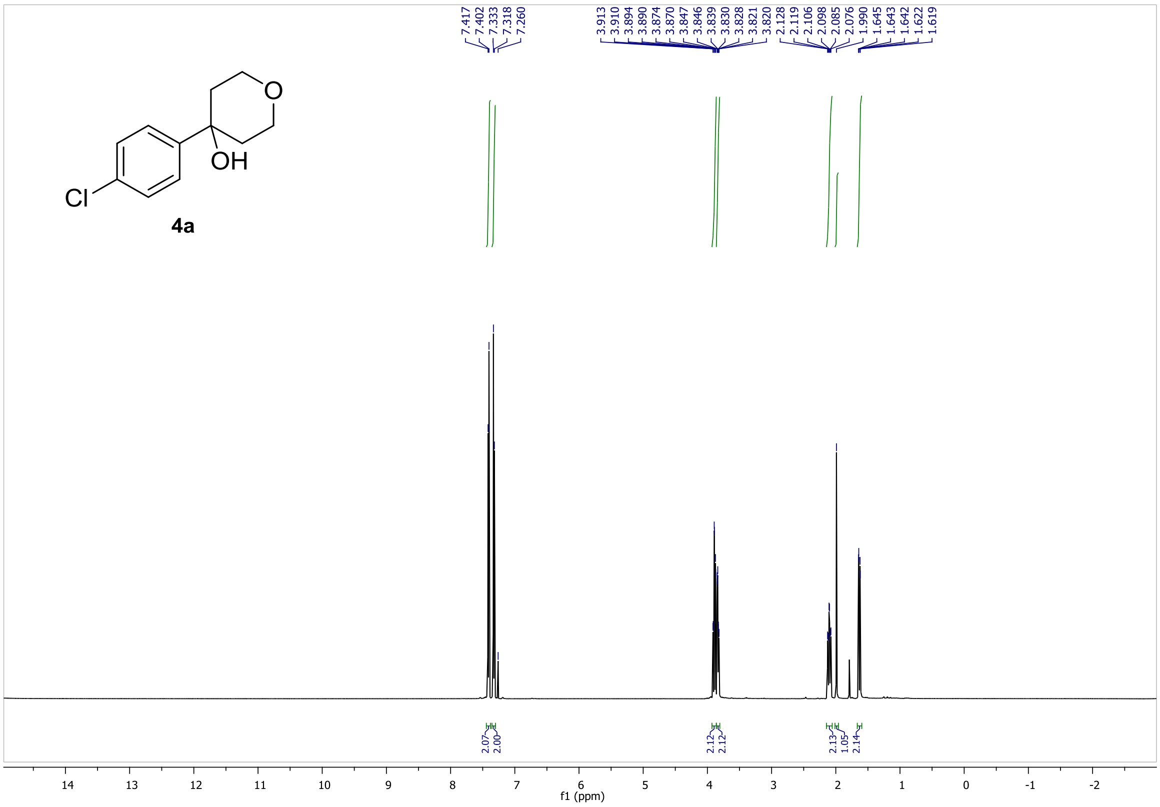

^{13}C NMR, 150 MHz, $CDCl_3$:

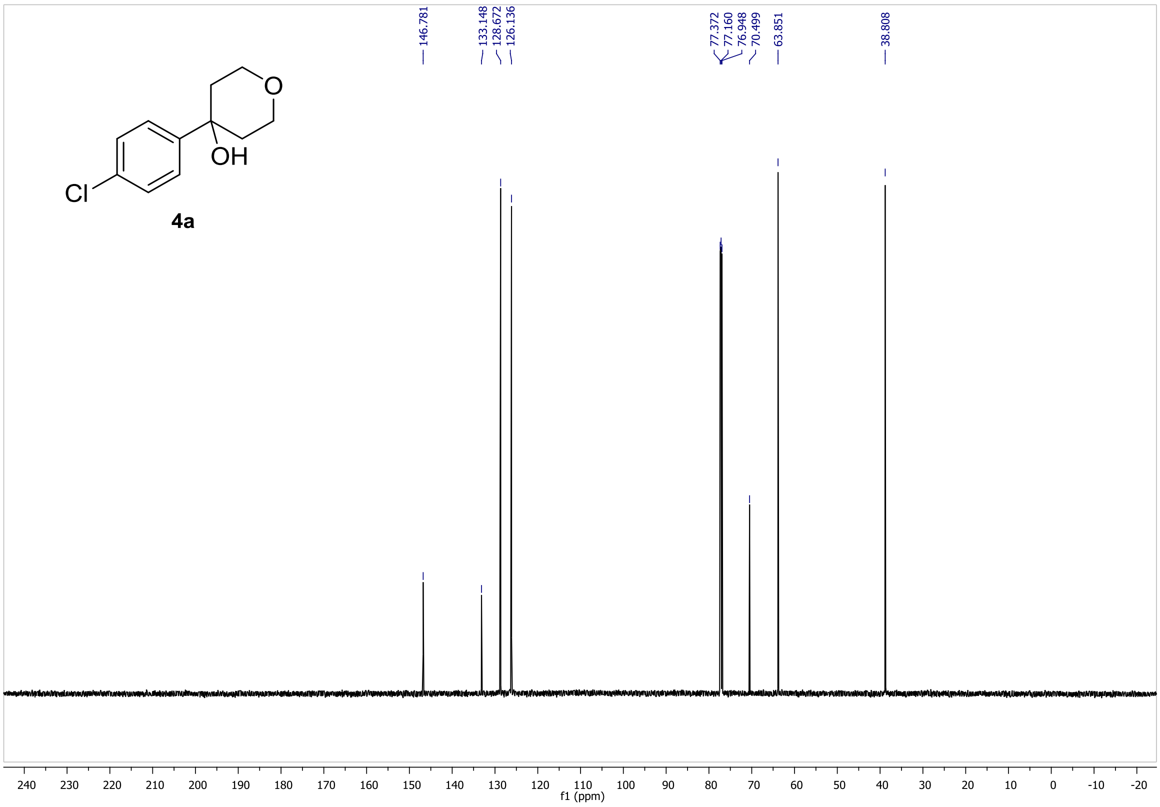

3-(4-chlorophenyl)tetrahydrofuran-3-ol (4b):

^{1}H NMR, 600 MHz, $CDCl_3$:

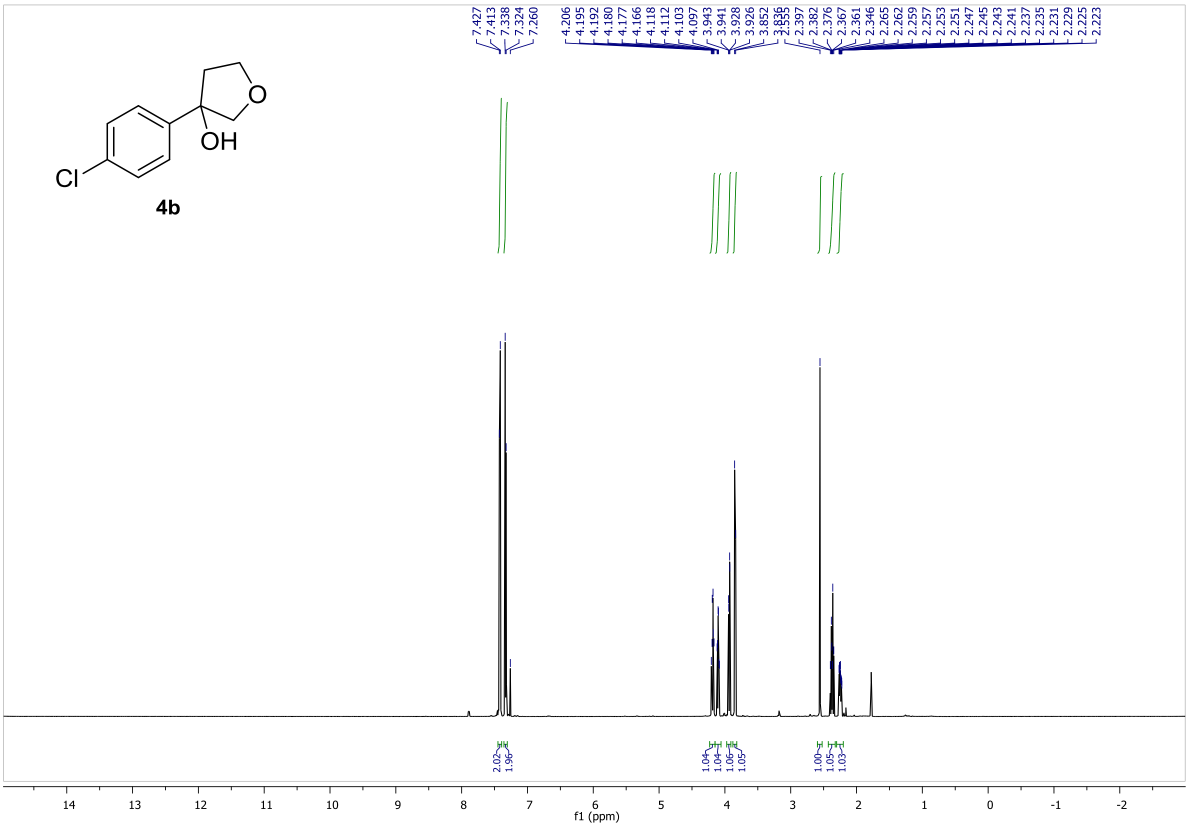

^{13}C NMR, 150 MHz, $CDCl_3$:

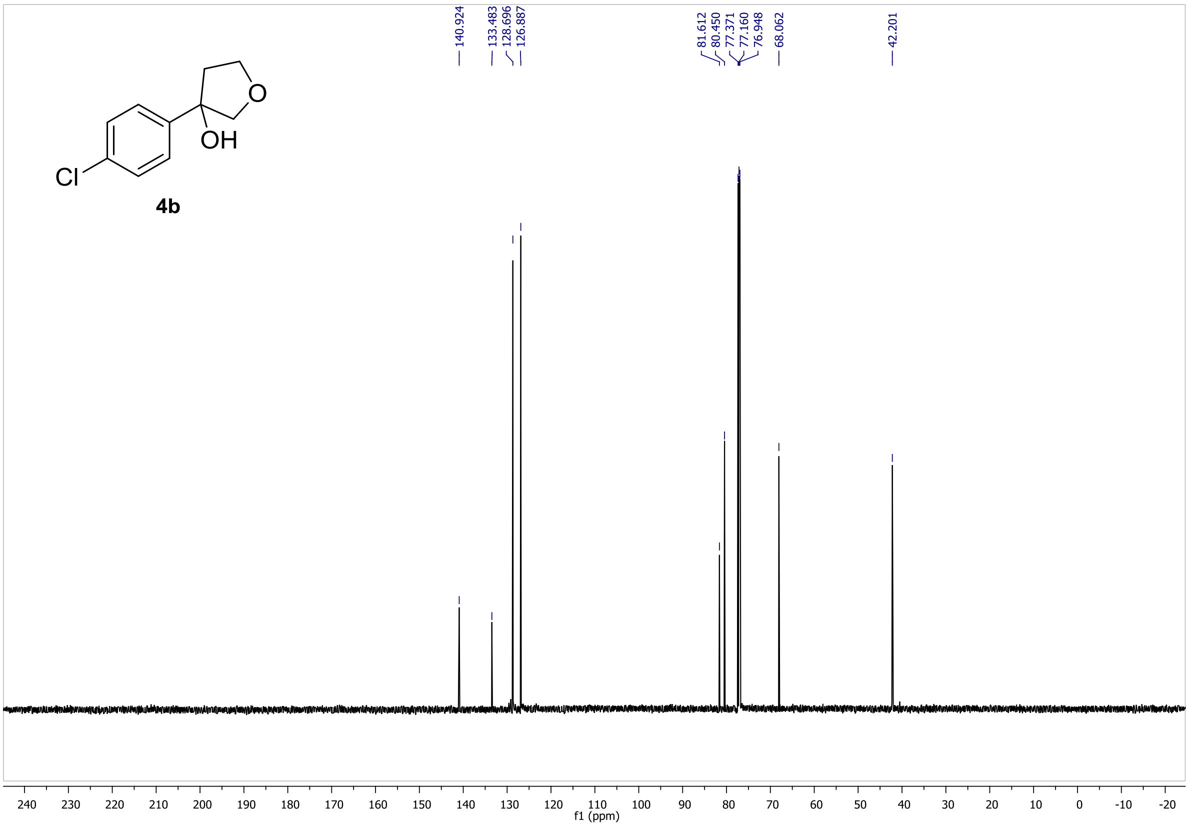

4-(4-chlorophenyl)tetrahydro-2*H*-thiopyran-4-ol (4c):

^{1}H NMR, 600 MHz, $CDCl_3$:

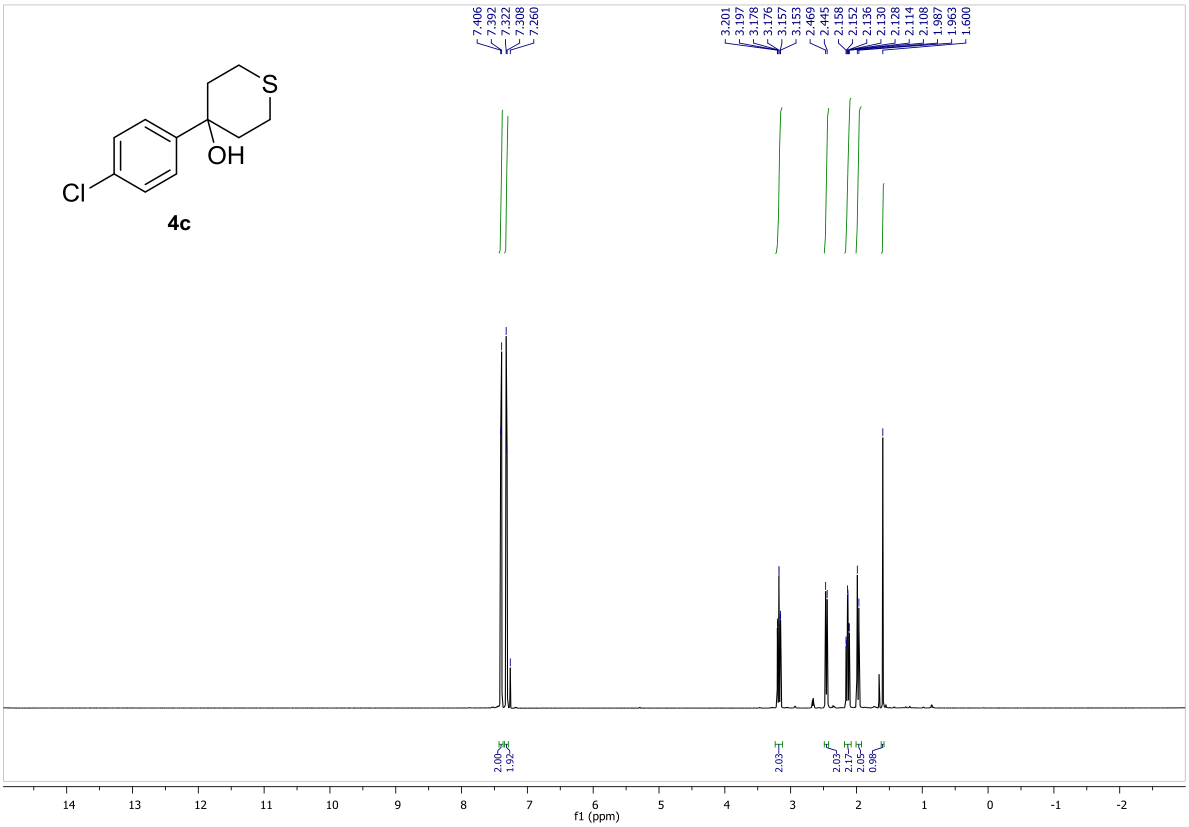

^{13}C NMR, 150 MHz, $CDCl_3$:

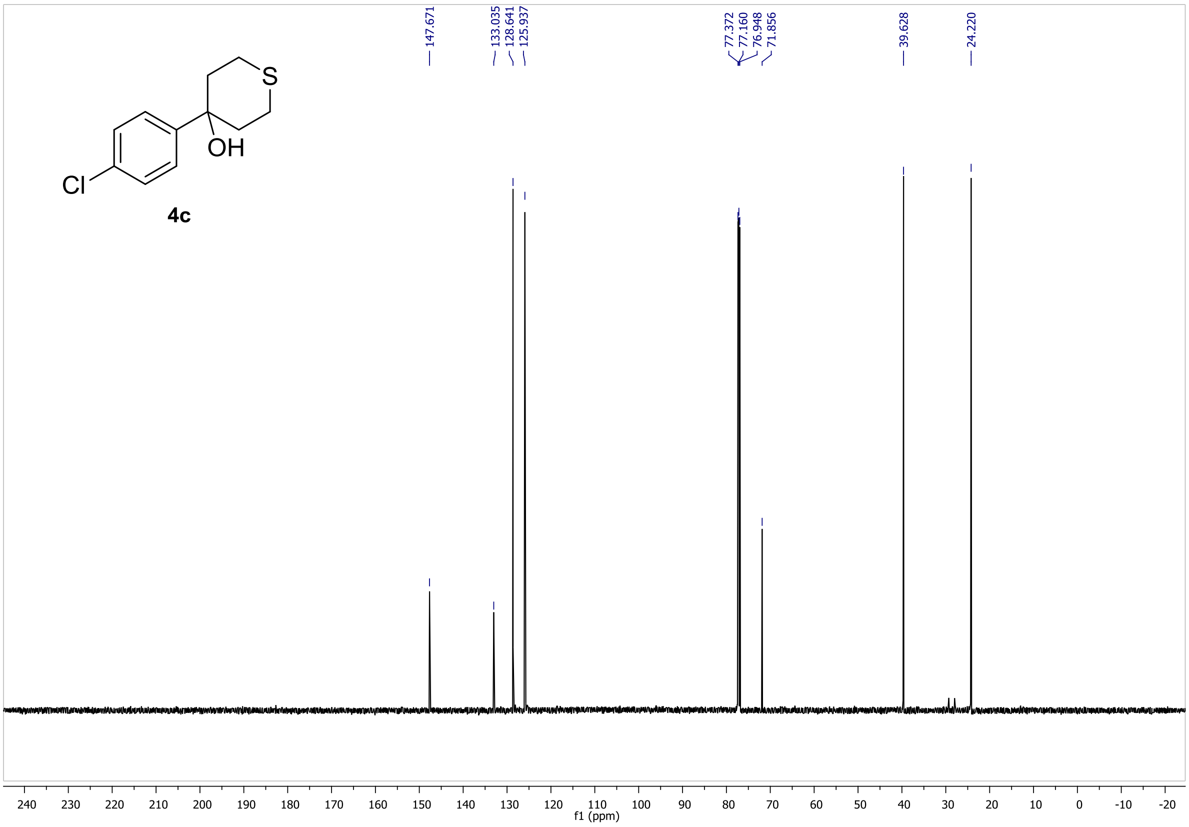

3-(4-chlorophenyl)tetrahydrothiophen-3-ol (4d):

^{1}H NMR, 600 MHz, CDCl$_3$:

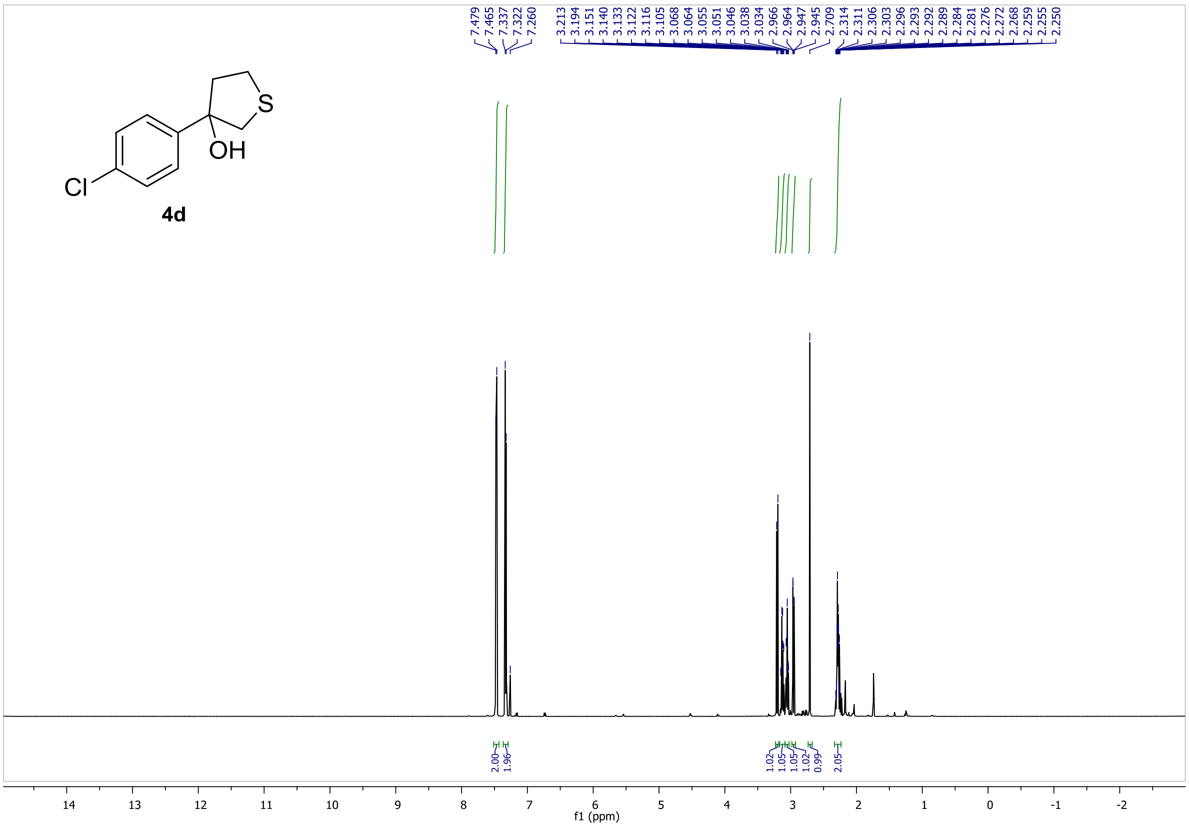

^{13}C NMR, 150 MHz, CDCl$_3$:

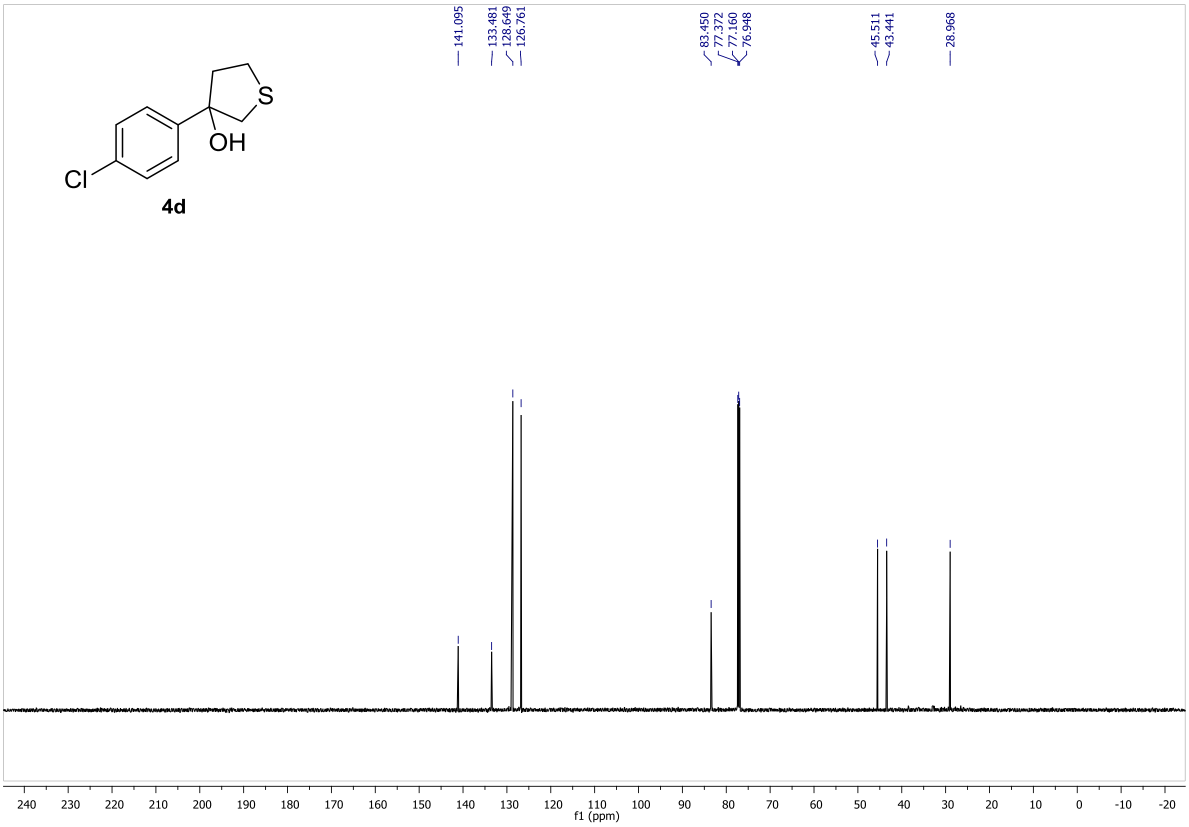

3-(4-chlorophenyl)thietan-3-ol (4e):

^{1}H NMR, 600 MHz, $CDCl_3$:

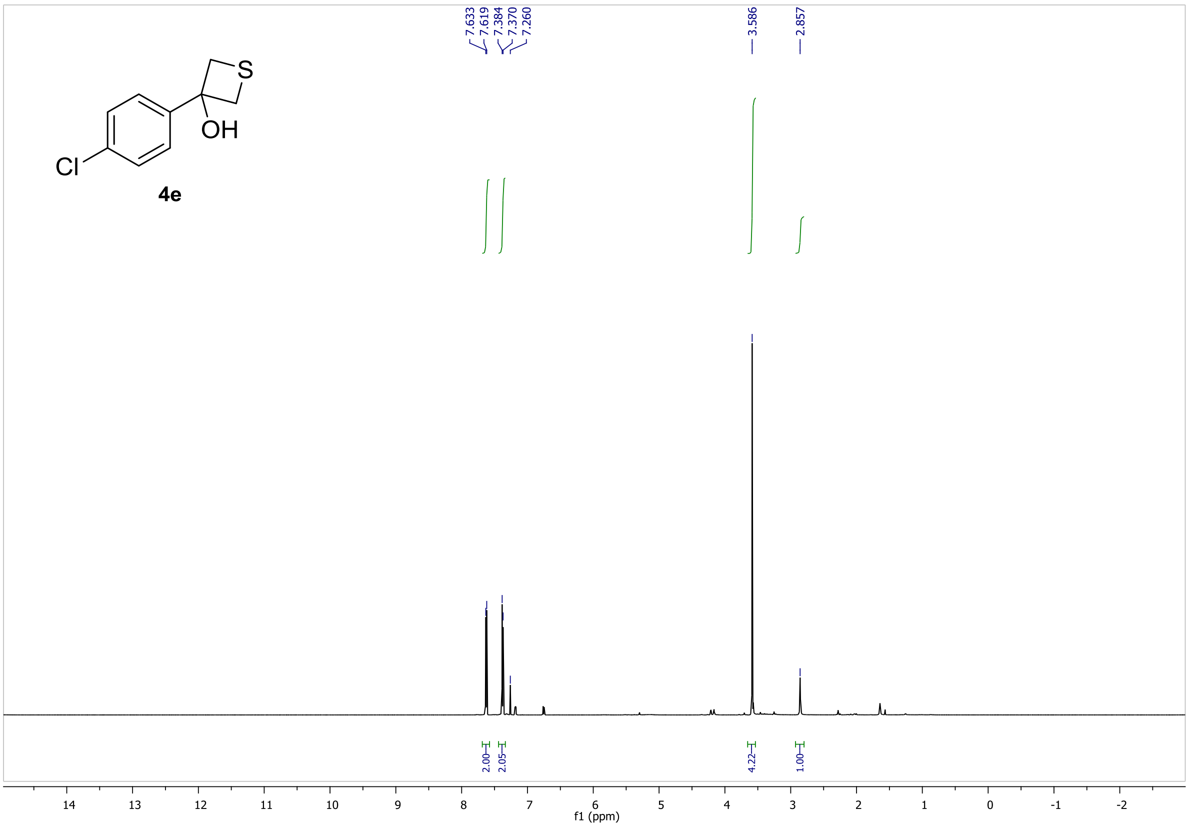

^{13}C NMR, 150 MHz, $CDCl_3$:

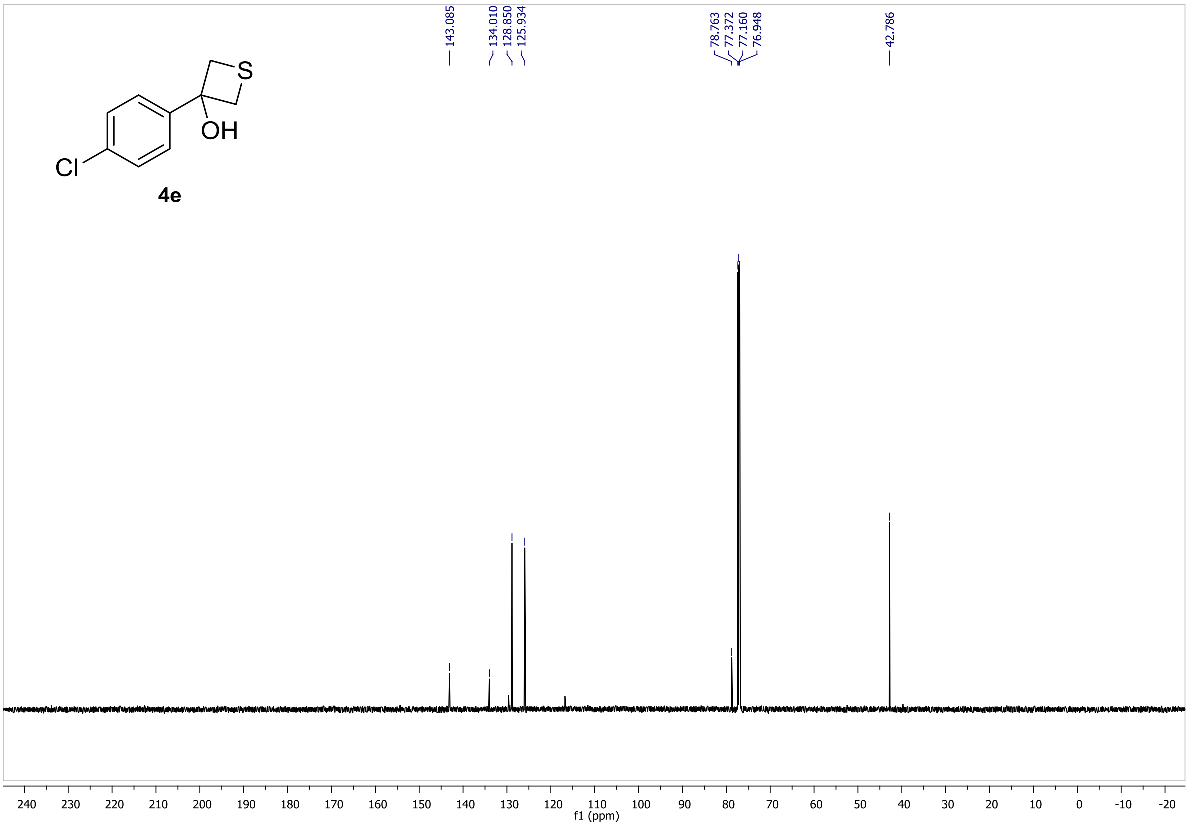

***tert*-butyl 4-(4-chlorophenyl)-4-hydroxypiperidine-1-carboxylate (4f):**

^{1}H NMR, 600 MHz, $CDCl_3$:

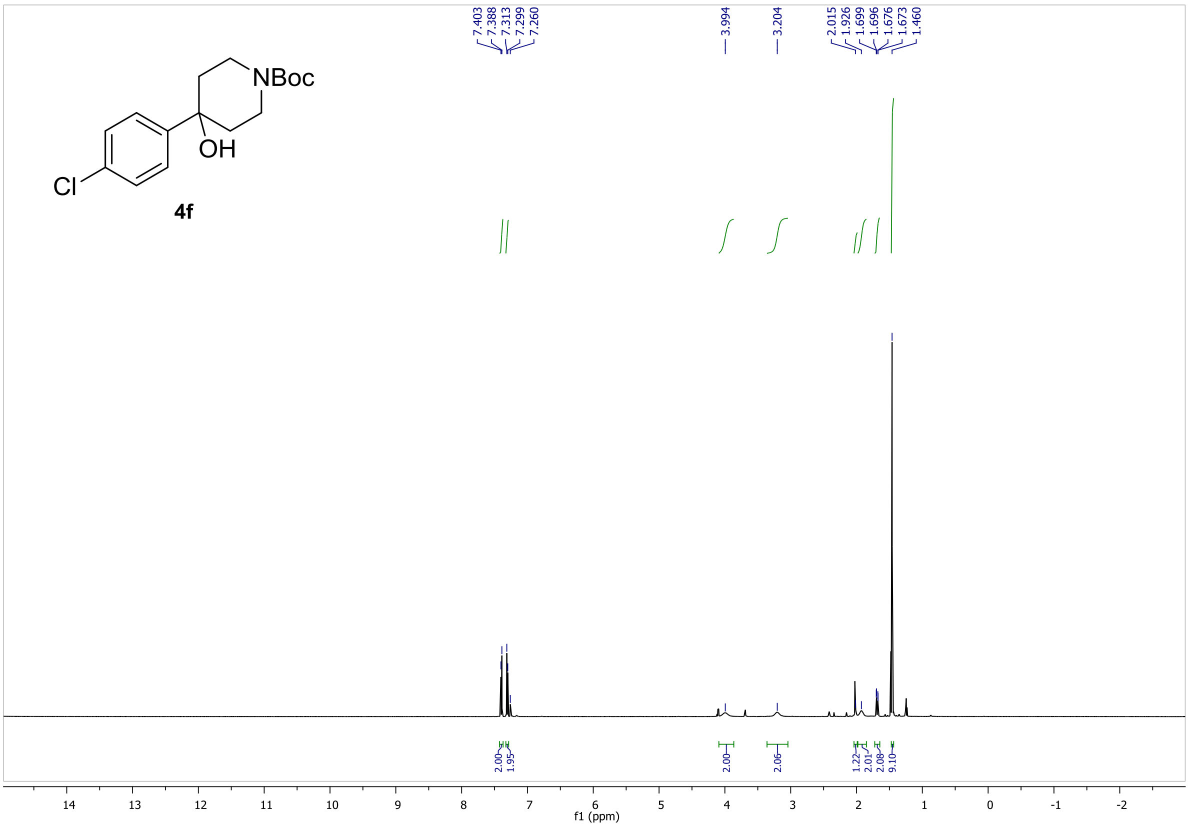

^{13}C NMR, 150 MHz, $CDCl_3$:

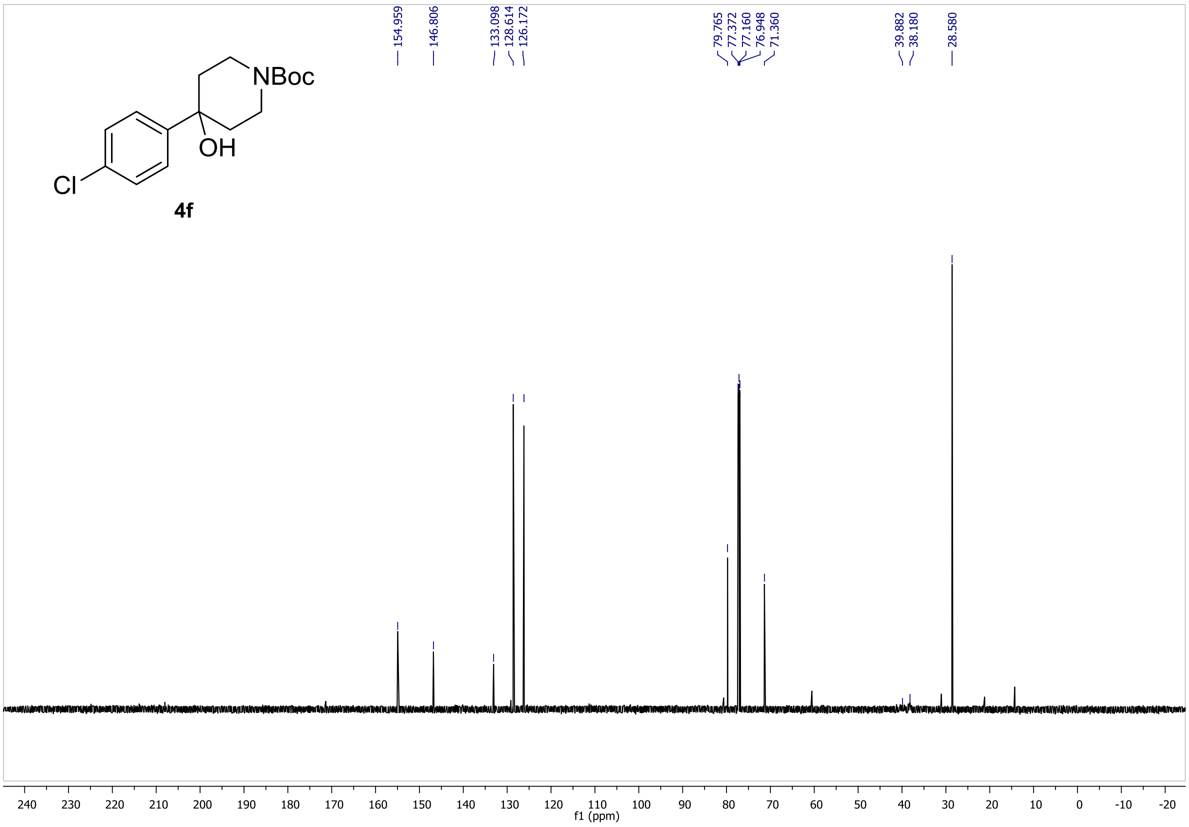

***tert*-butyl 3-(4-chlorophenyl)-3-hydroxypyrrolidine-1-carboxylate (4g):**

^{1}H NMR, 600 MHz, $CDCl_3$:

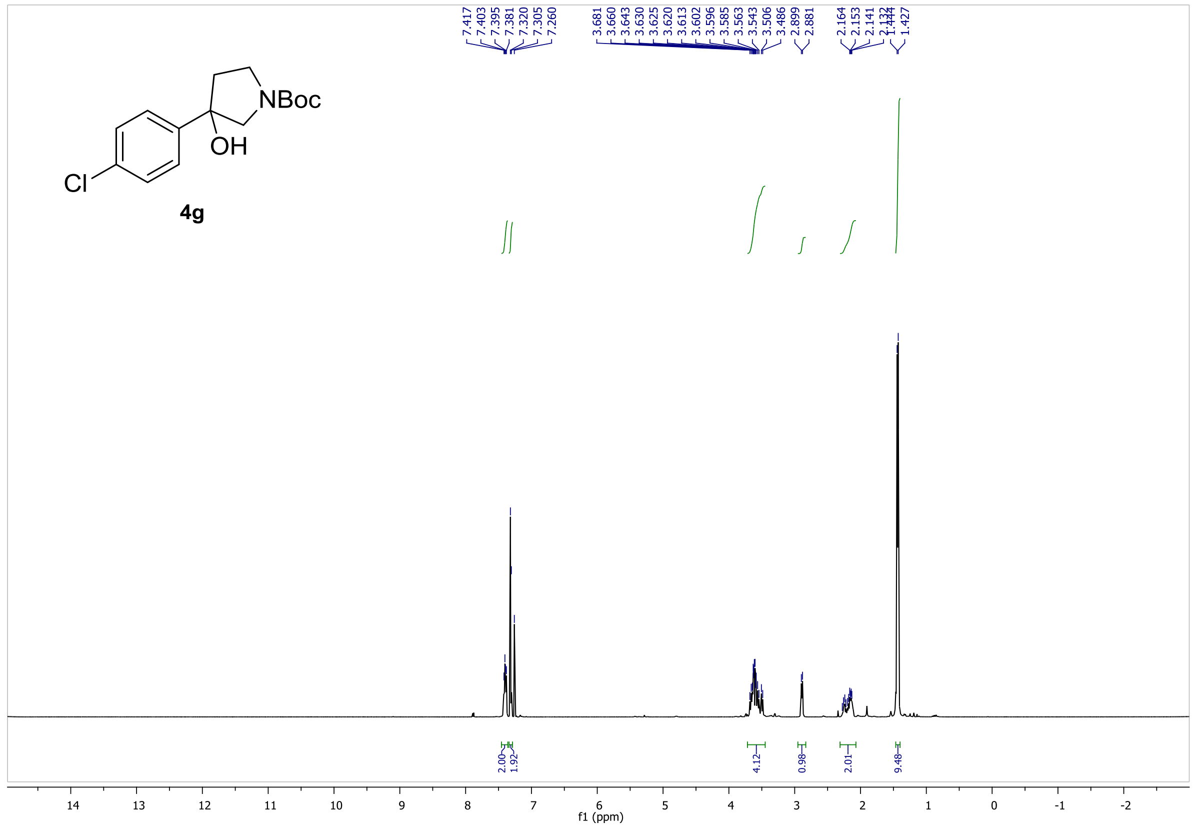

^{13}C NMR, 150 MHz, $CDCl_3$:

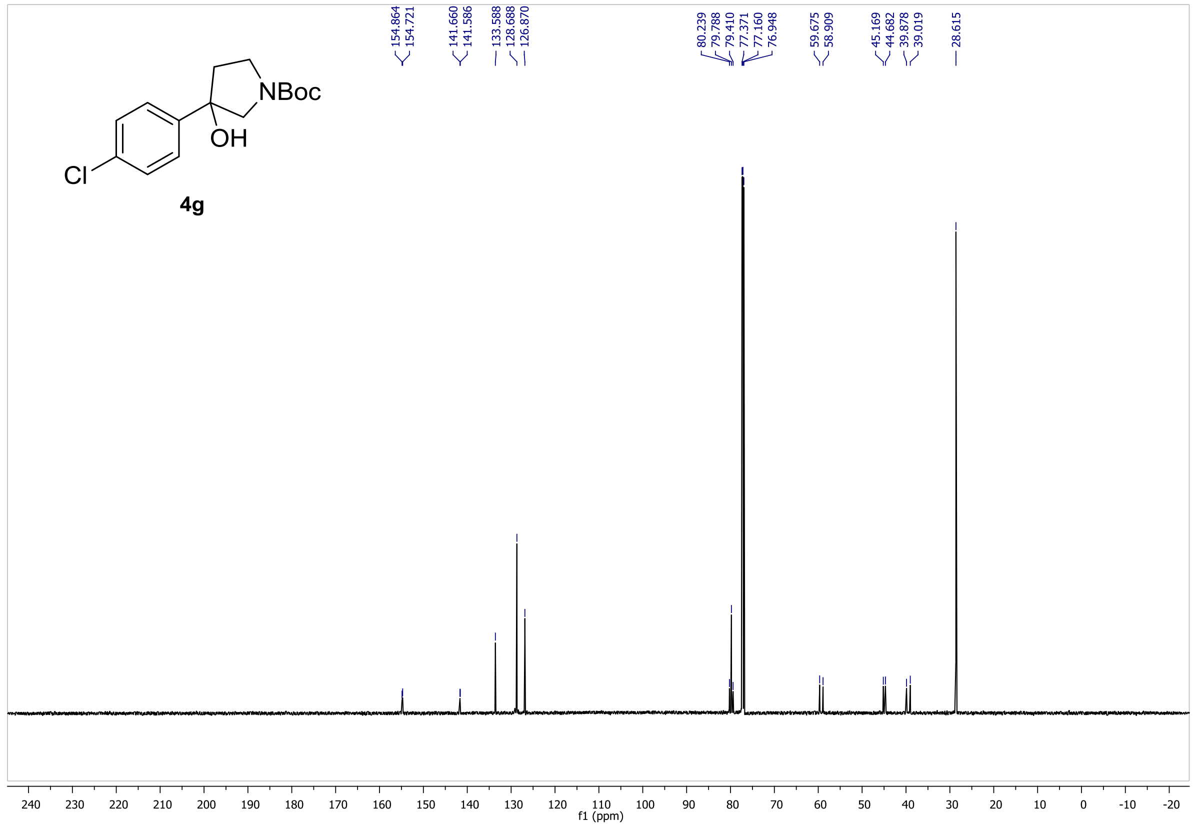

***tert*-butyl 3-(4-chlorophenyl)-3-hydroxyazetidine-1-carboxylate (4h):**

^{1}H NMR, 600 MHz, CDCl$_3$:

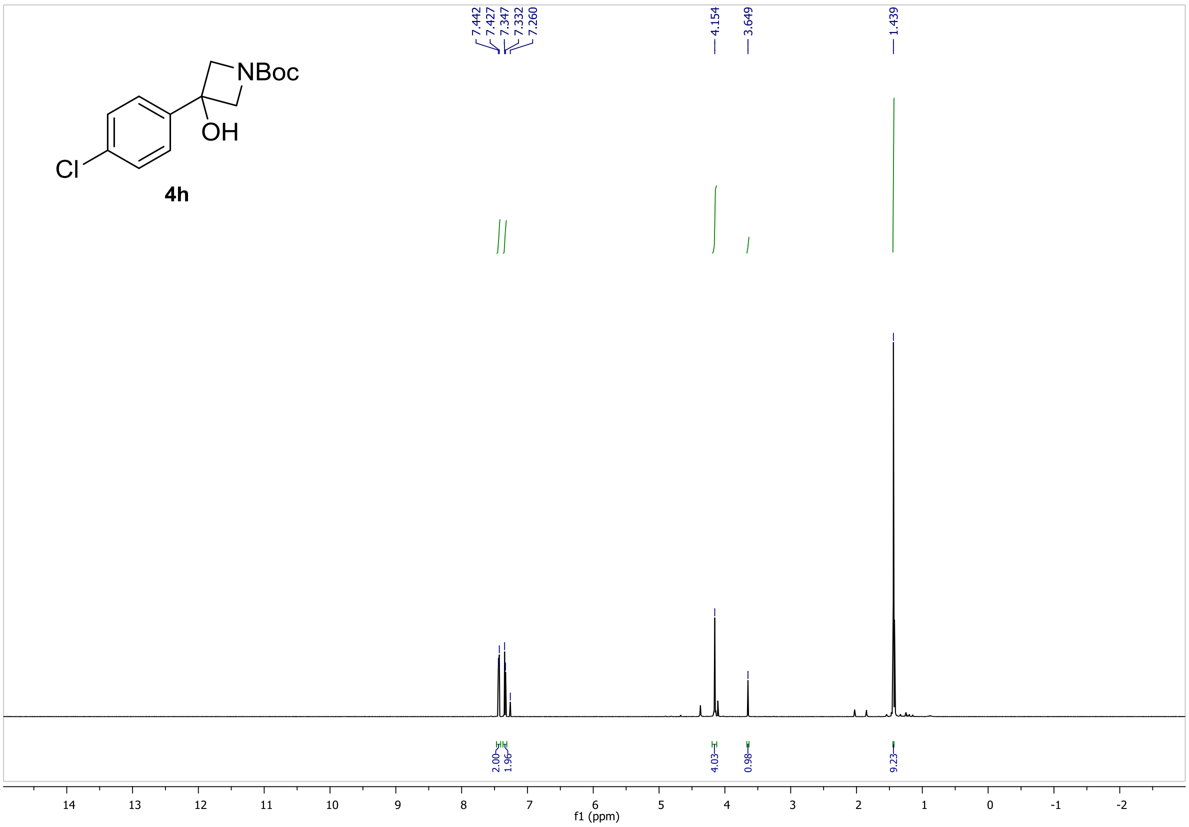

^{13}C NMR, 150 MHz, CDCl$_3$:

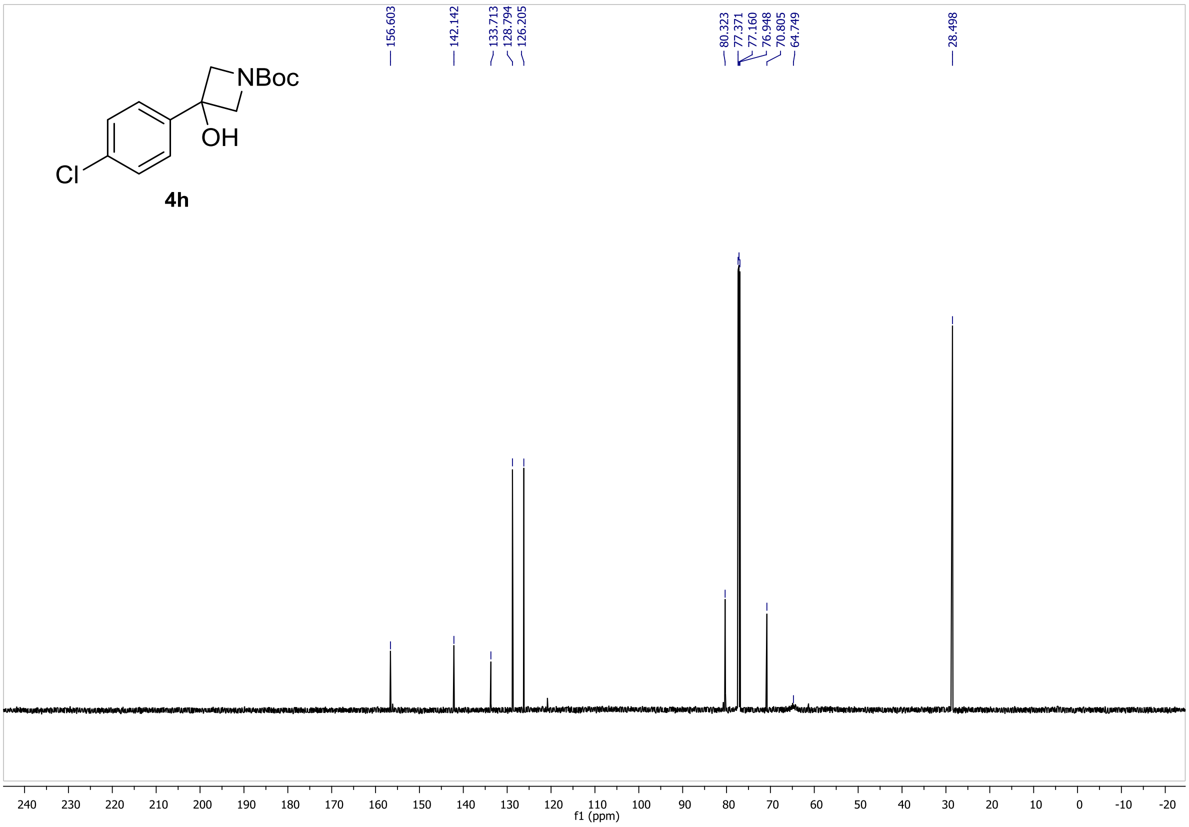

1-(4-chlorophenyl)cyclohexan-1-ol (4i):

^{1}H NMR, 600 MHz, $CDCl_3$:

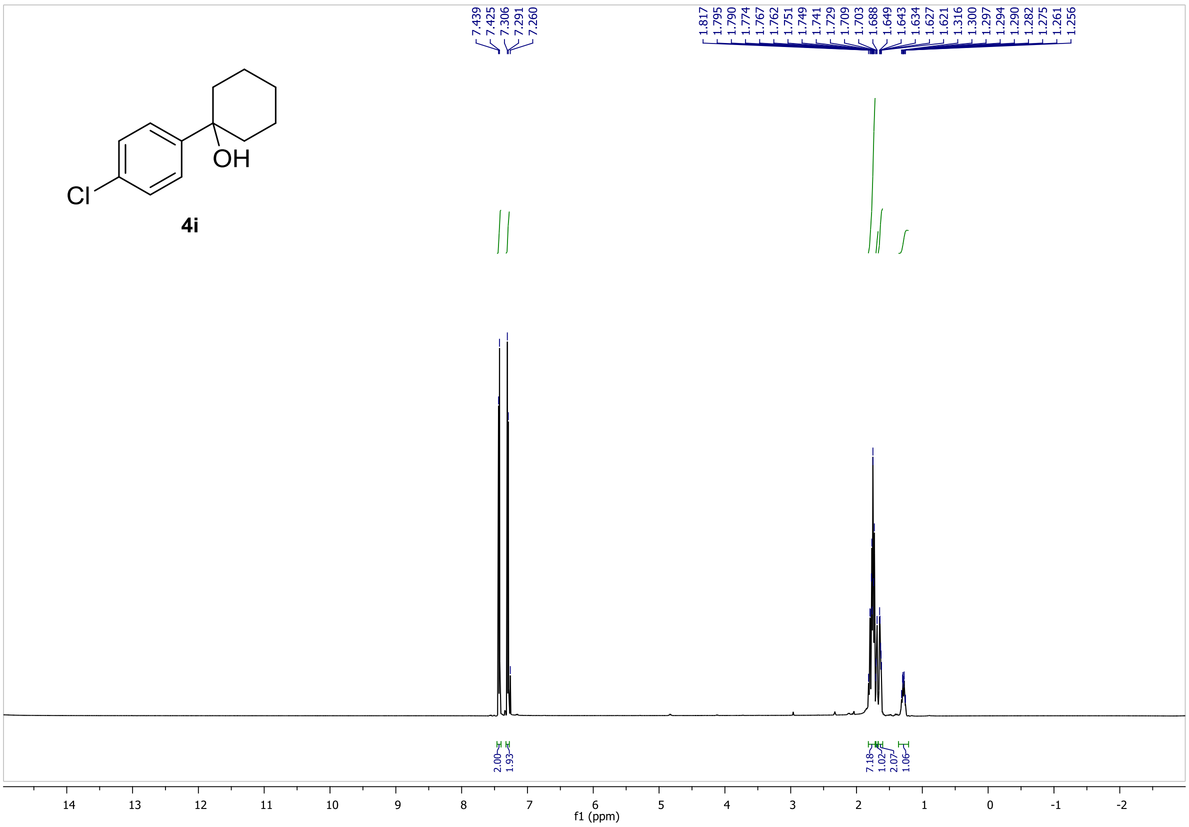

^{13}C NMR, 150 MHz, $CDCl_3$:

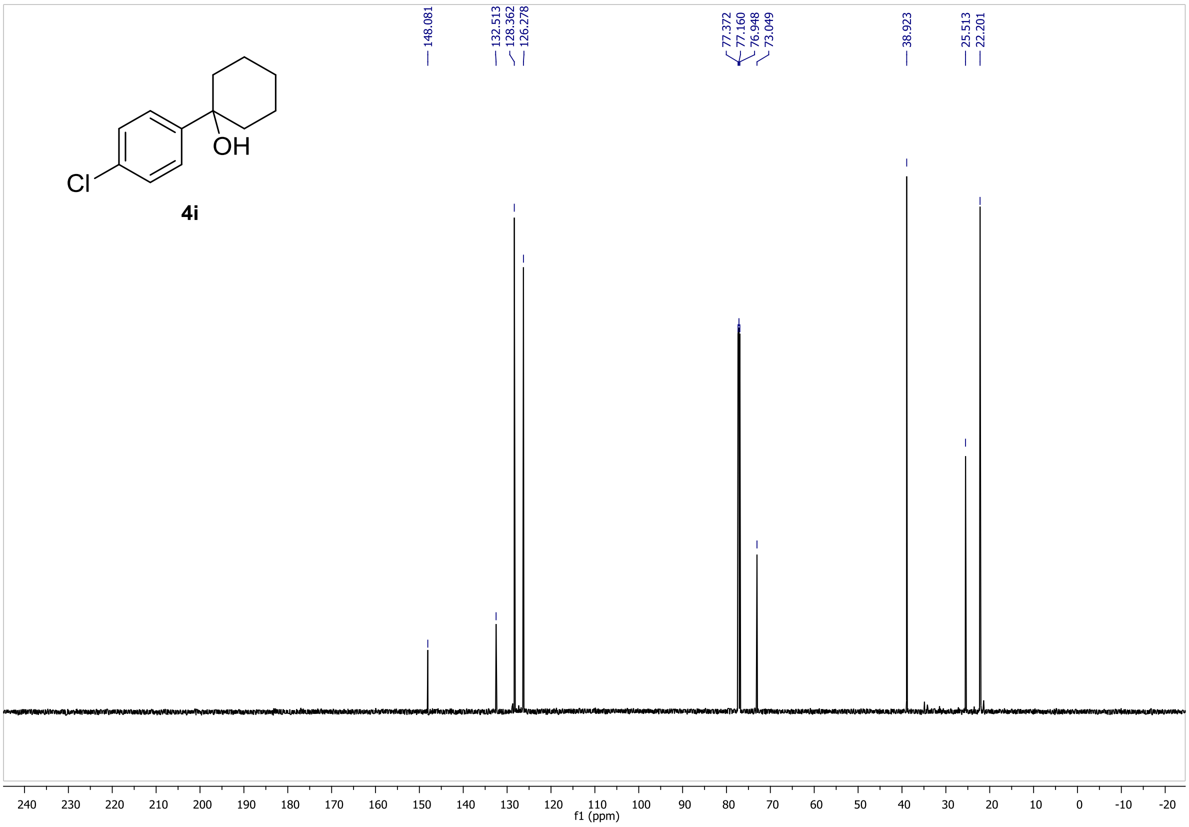

1-(4-chlorophenyl)cyclopentan-1-ol (4j):

^{1}H NMR, 600 MHz, $CDCl_3$:

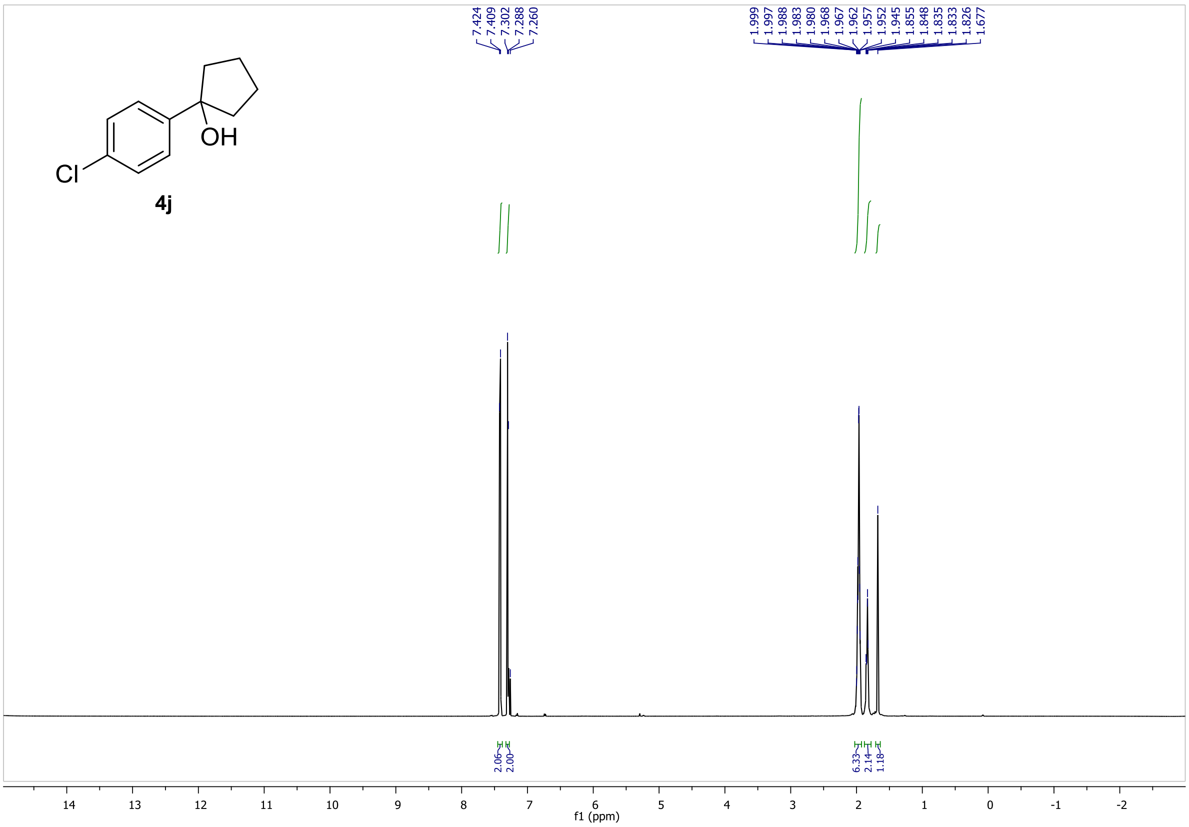

^{13}C NMR, 150 MHz, $CDCl_3$:

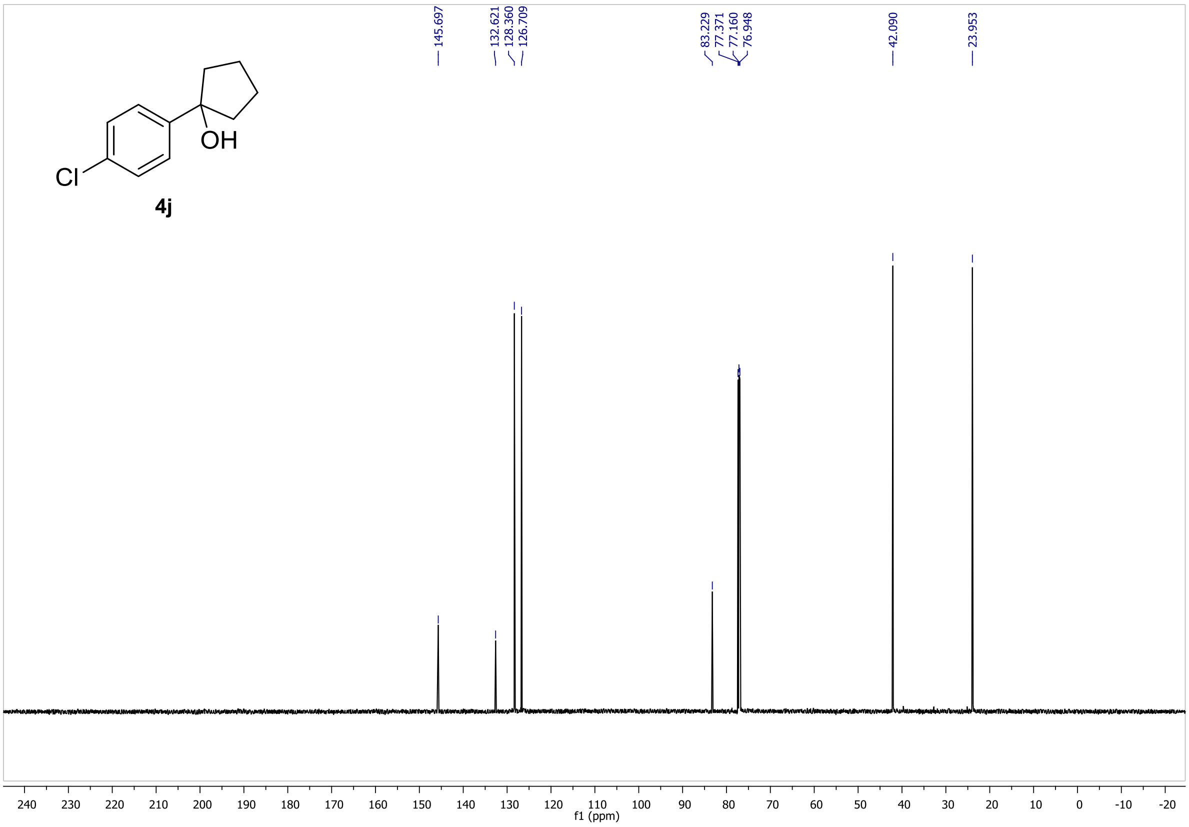

1-(4-chlorophenyl)cyclobutan-1-ol (4k):

^{1}H NMR, 600 MHz, $CDCl_3$:

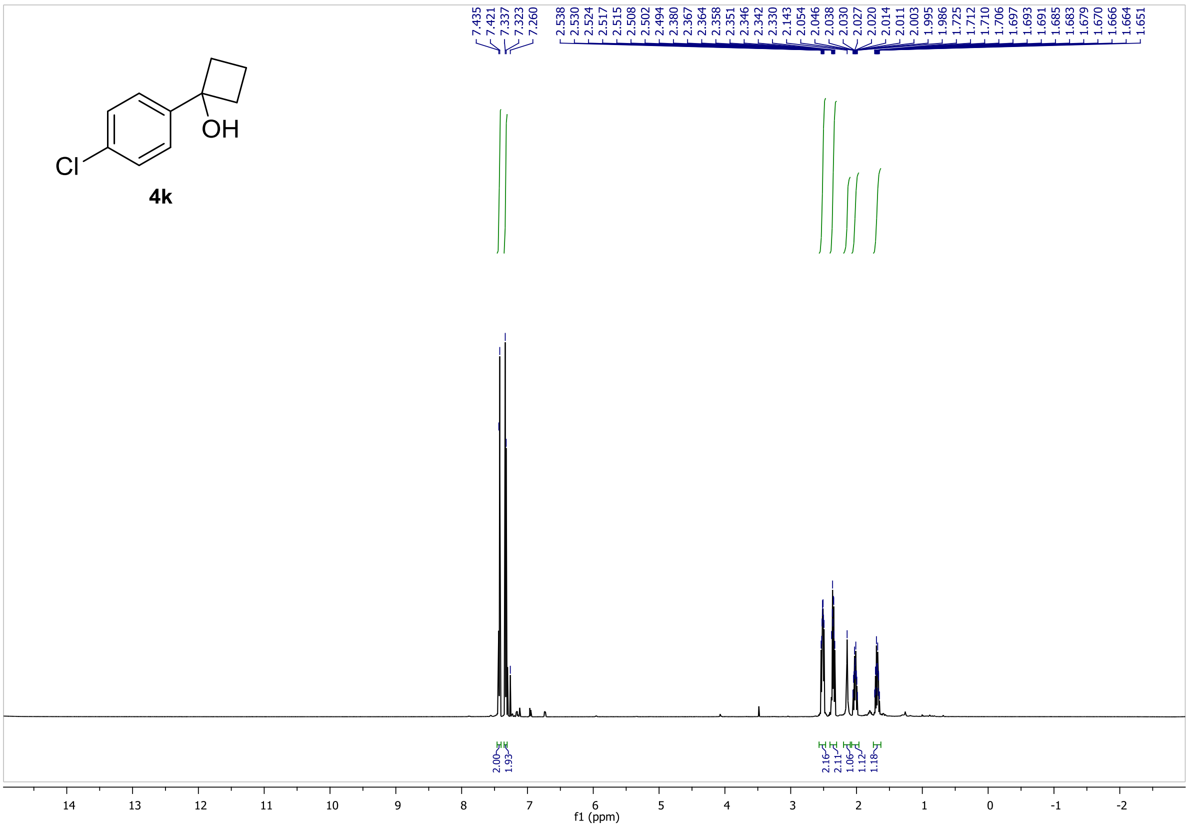

^{13}C NMR, 150 MHz, $CDCl_3$:

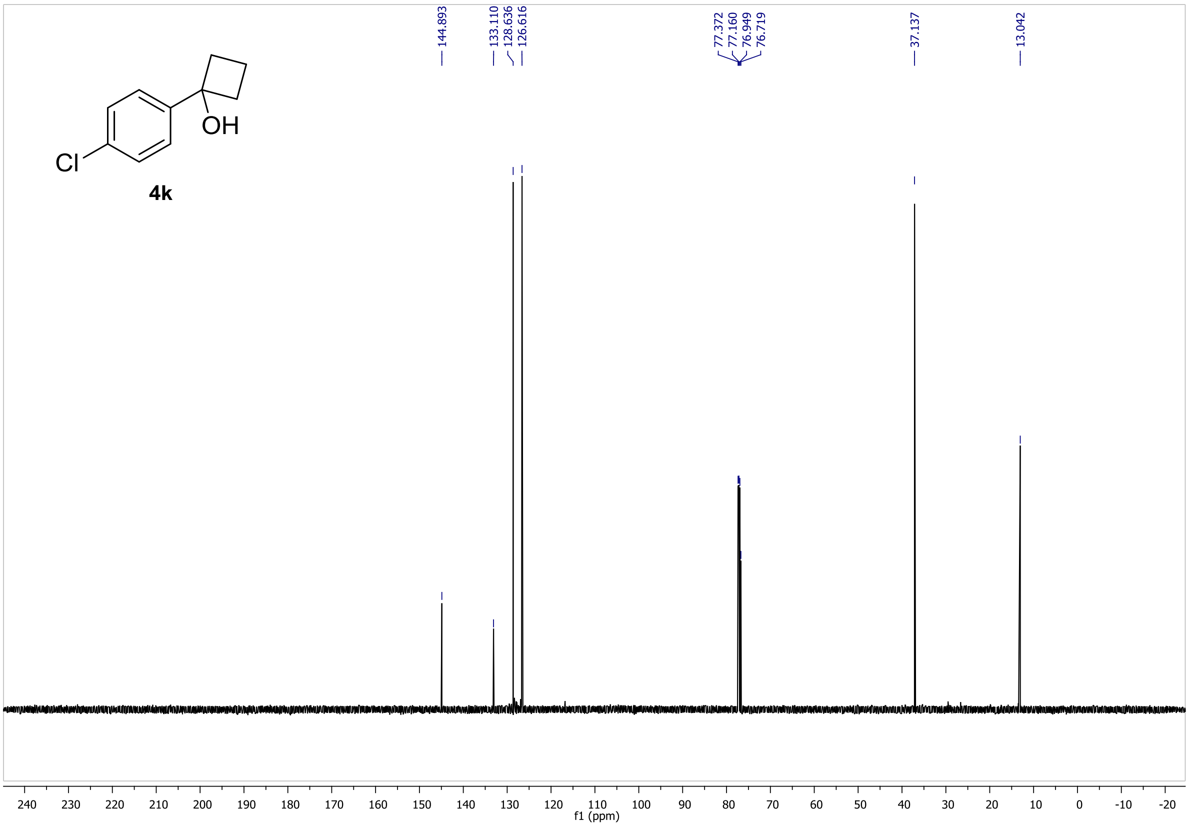

2-(4-chlorophenyl)-1,2,3,4-tetrahydronaphthalen-2-ol (4l):

^{1}H NMR, 600 MHz, CDCl$_3$:

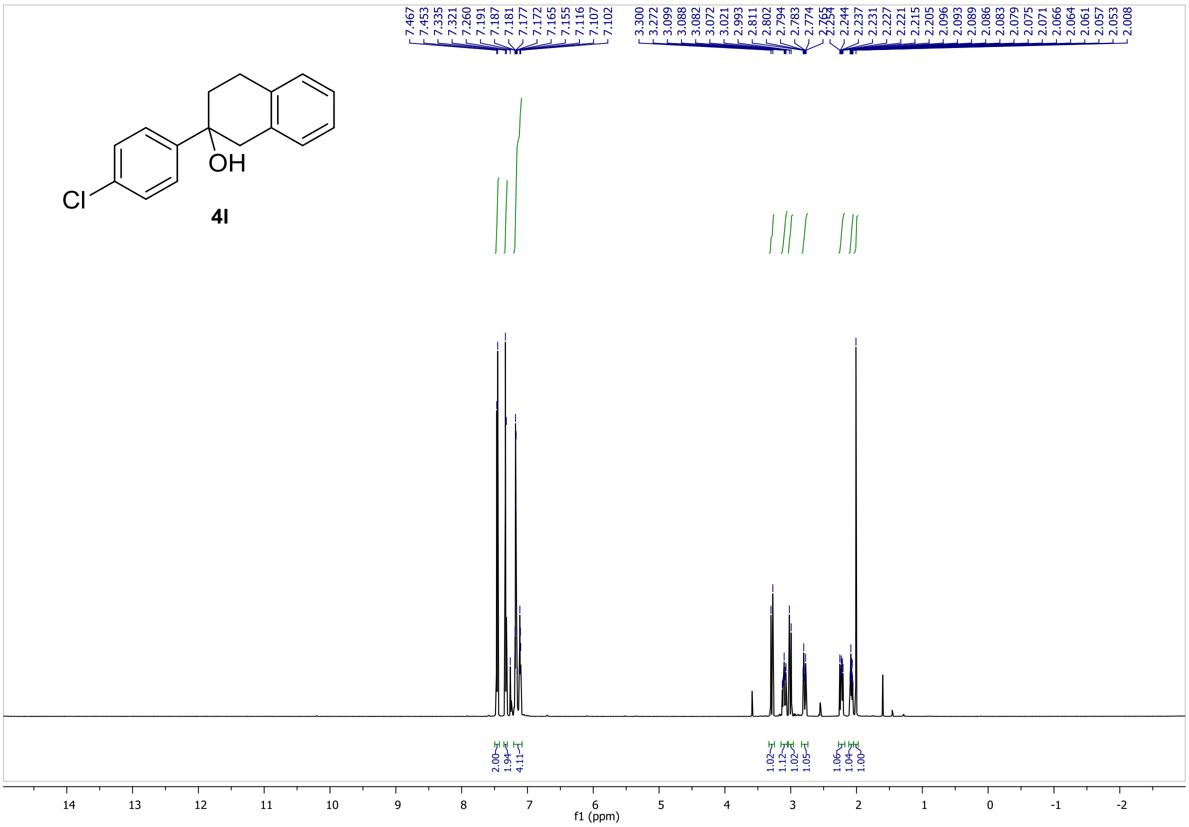

^{13}C NMR, 150 MHz, CDCl$_3$:

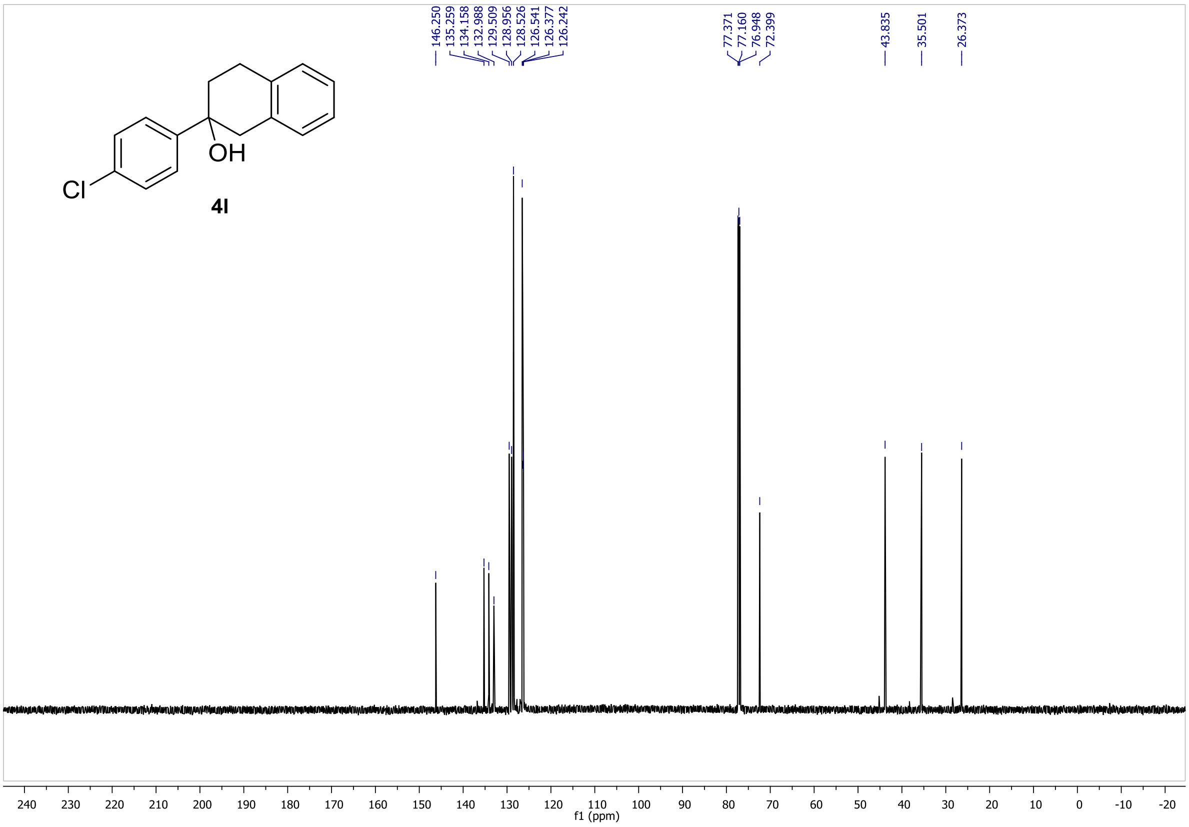

1-(4-chlorophenyl)-1-cyclopropylethan-1-ol (4n):

^{1}H NMR, 600 MHz, $CDCl_3$:

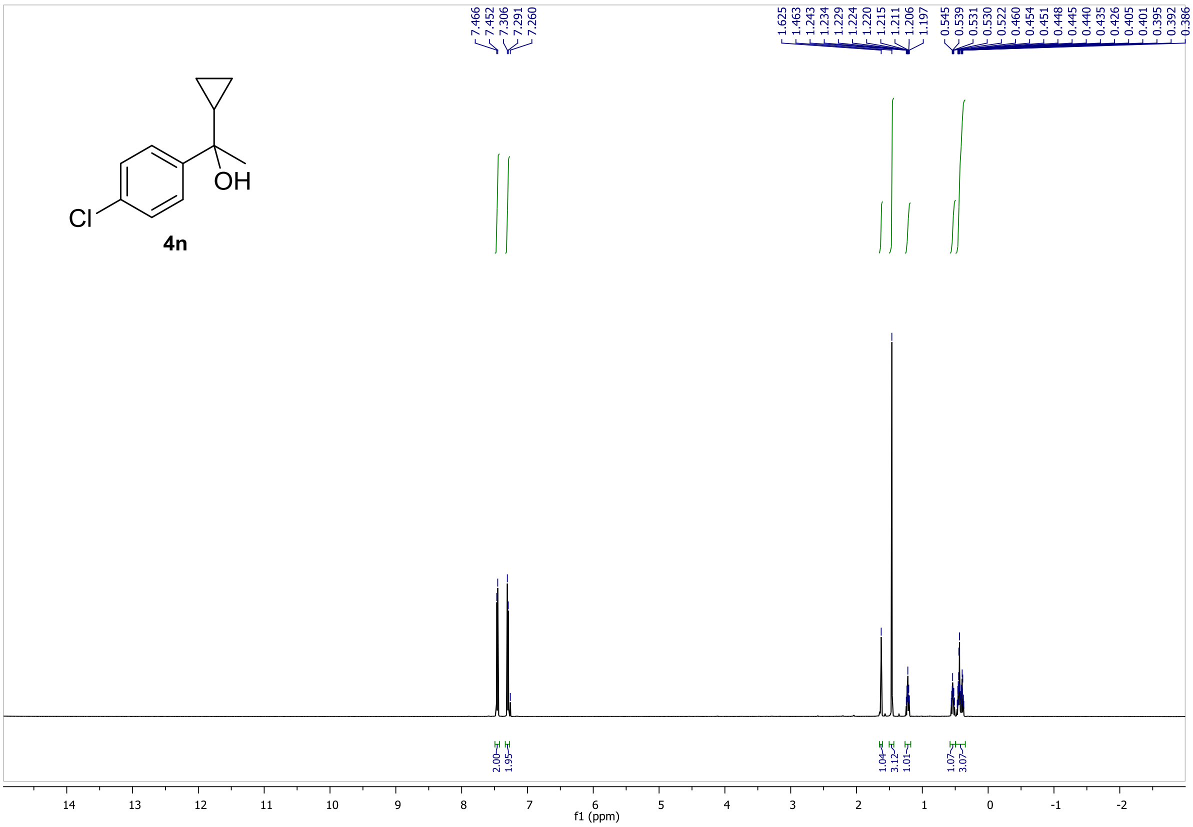

^{13}C NMR, 150 MHz, $CDCl_3$:

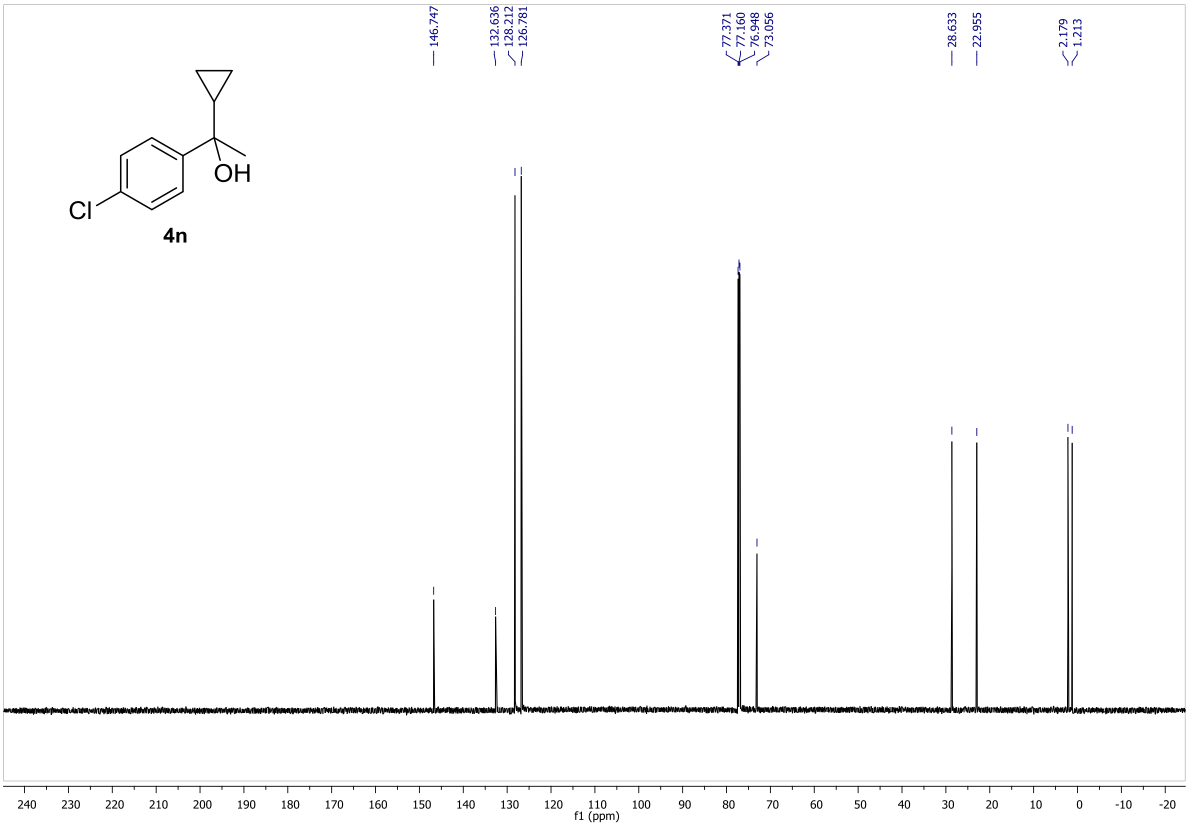

2-(4-chlorophenyl)hex-5-en-2-ol (4o):

^{1}H NMR, 600 MHz, $CDCl_3$:

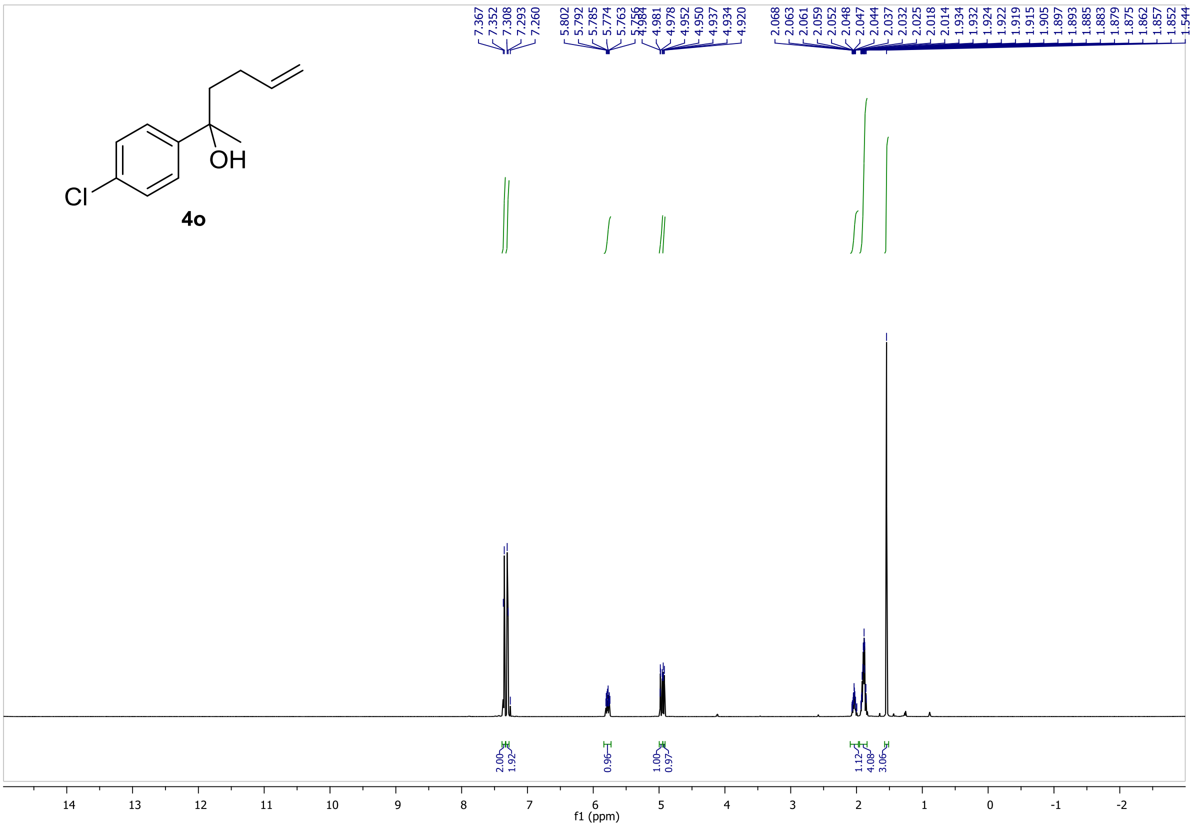

^{13}C NMR, 150 MHz, $CDCl_3$:

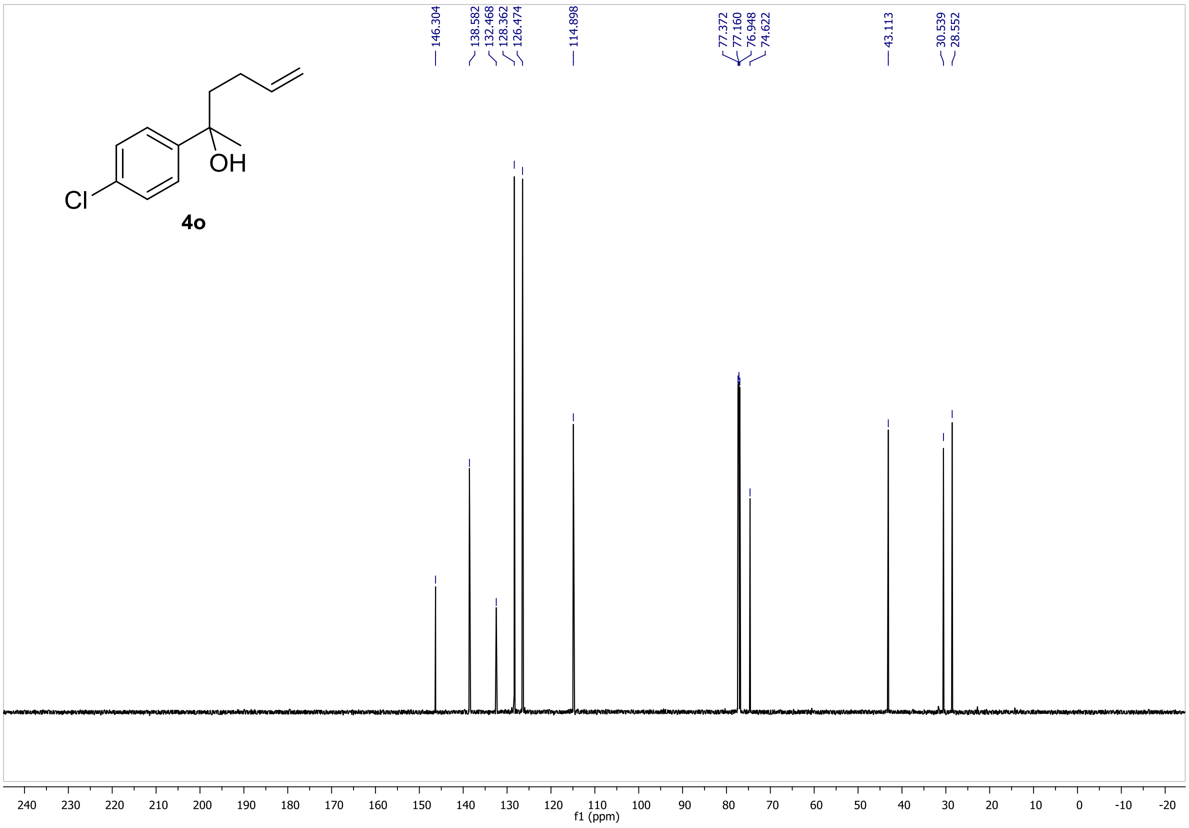

2-(4-chlorophenyl)-4,4-dimethylhept-6-yn-2-ol (4p):

^{1}H NMR, 600 MHz, $CDCl_3$:

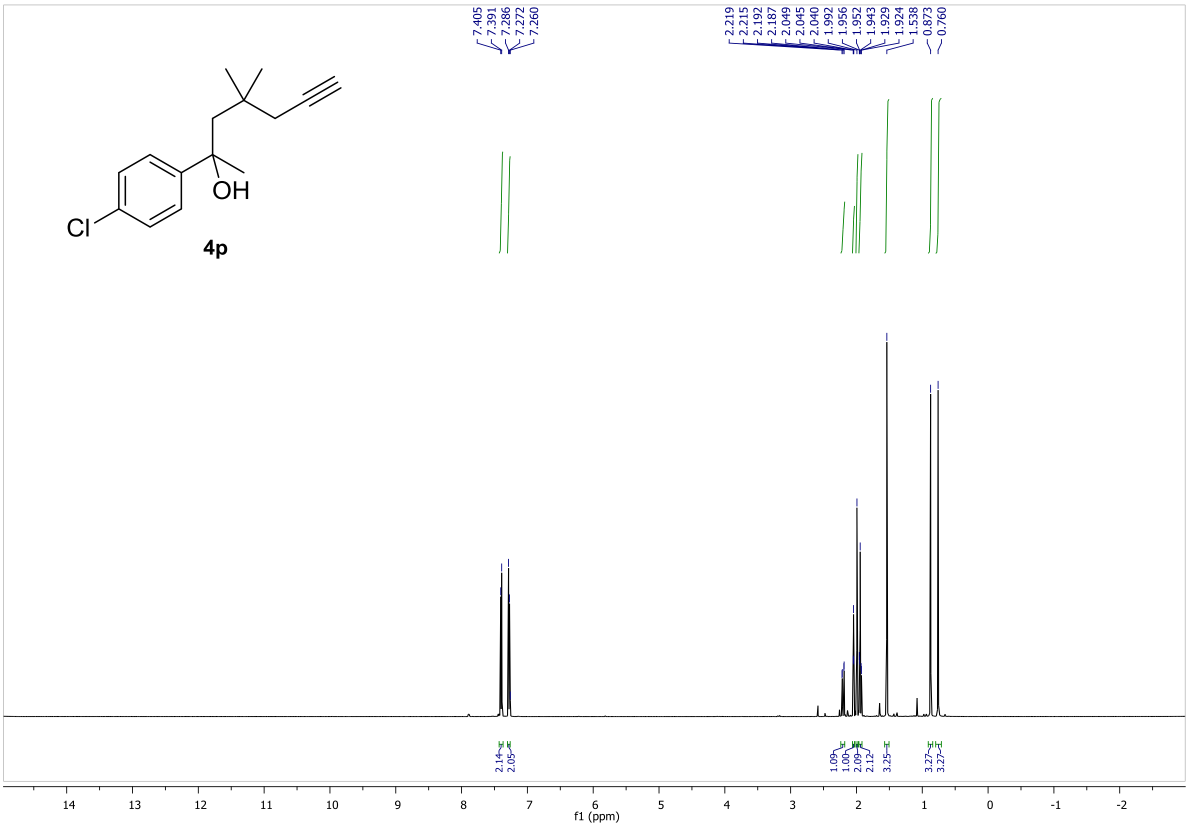

^{13}C NMR, 150 MHz, $CDCl_3$:

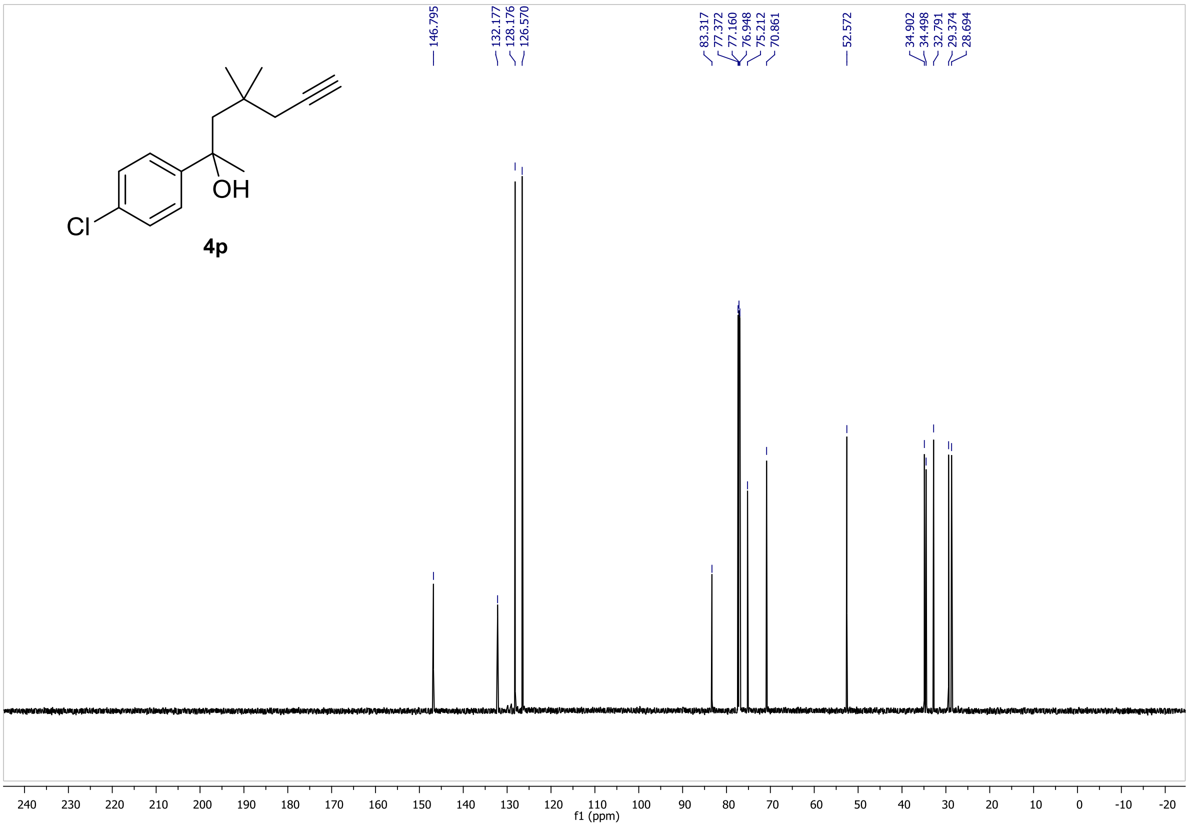

8-(4-chlorophenyl)-1,4-dioxaspiro[4.5]decan-8-ol (4q):

^{1}H NMR, 600 MHz, $CDCl_3$:

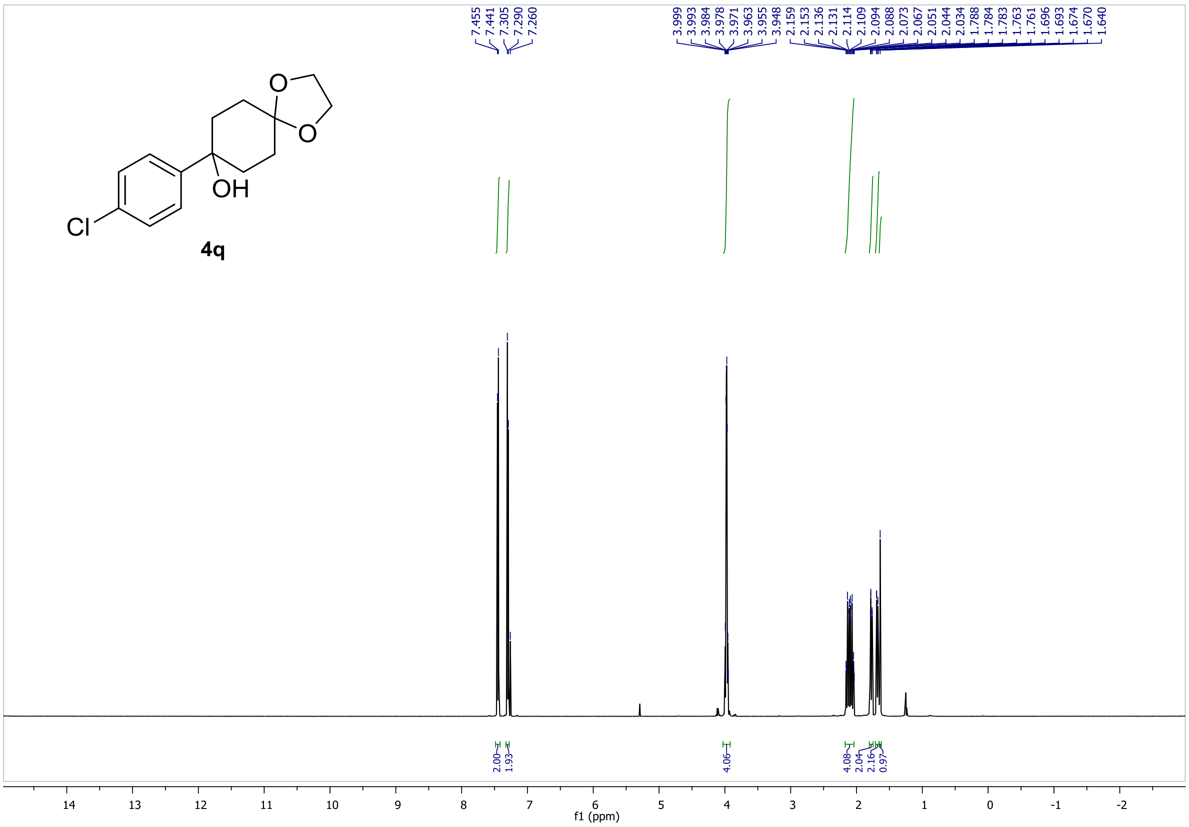

^{13}C NMR, 150 MHz, $CDCl_3$:

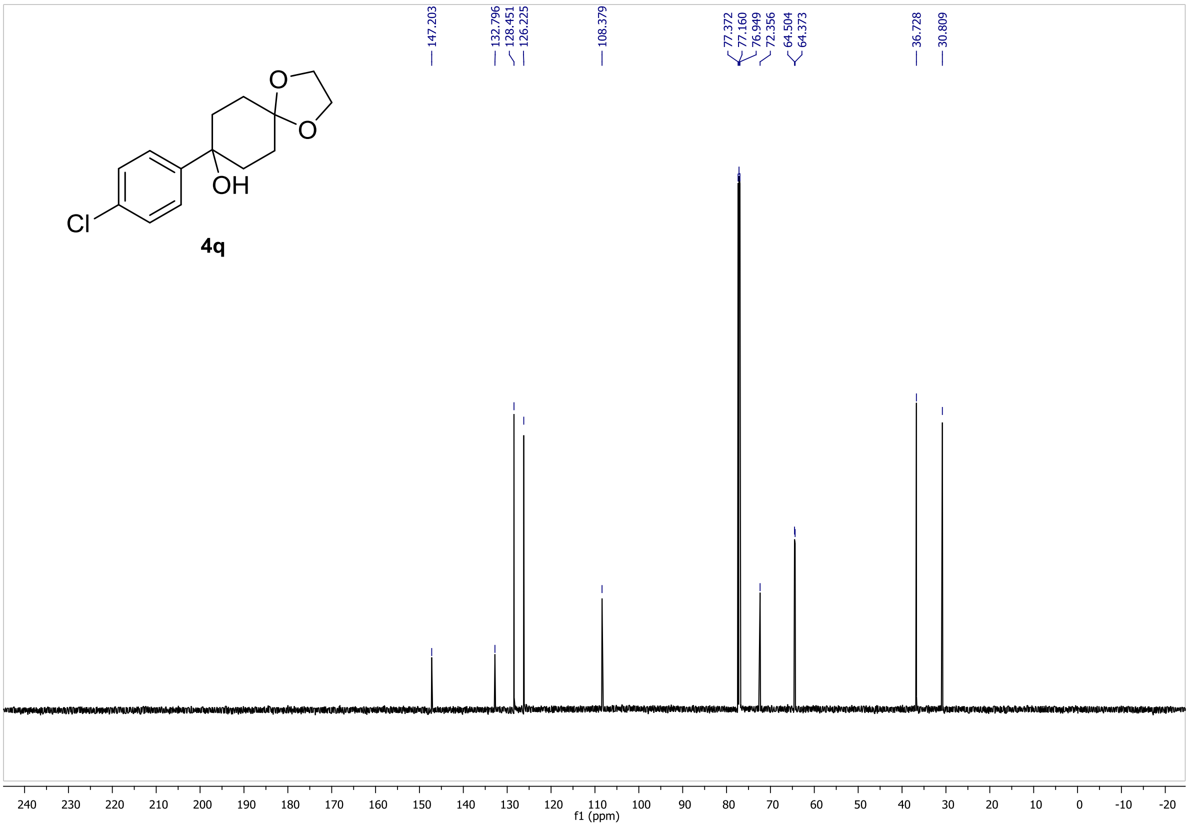

Diethyl (2-(4-chlorophenyl)-2-hydroxypropyl)phosphonate (4r):

^{1}H NMR, 600 MHz, CDCl$_3$:

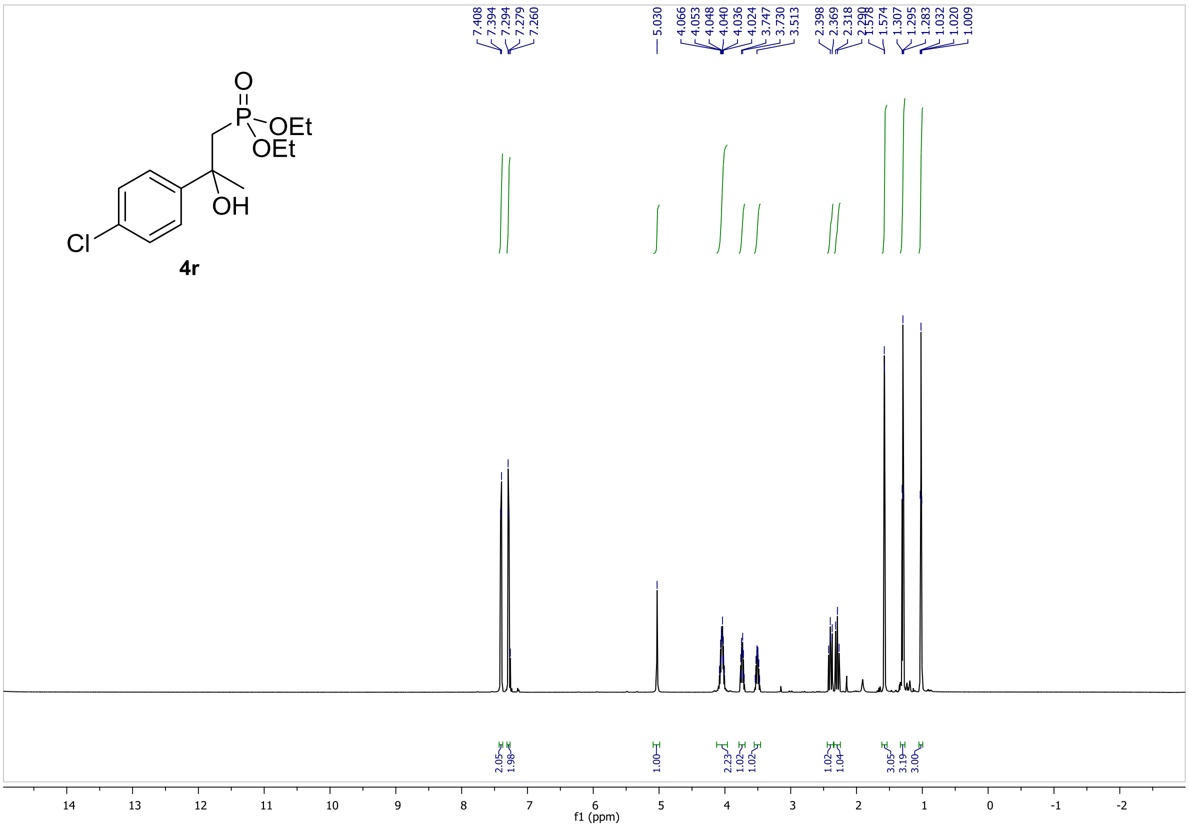

^{13}C NMR, 150 MHz, CDCl$_3$:

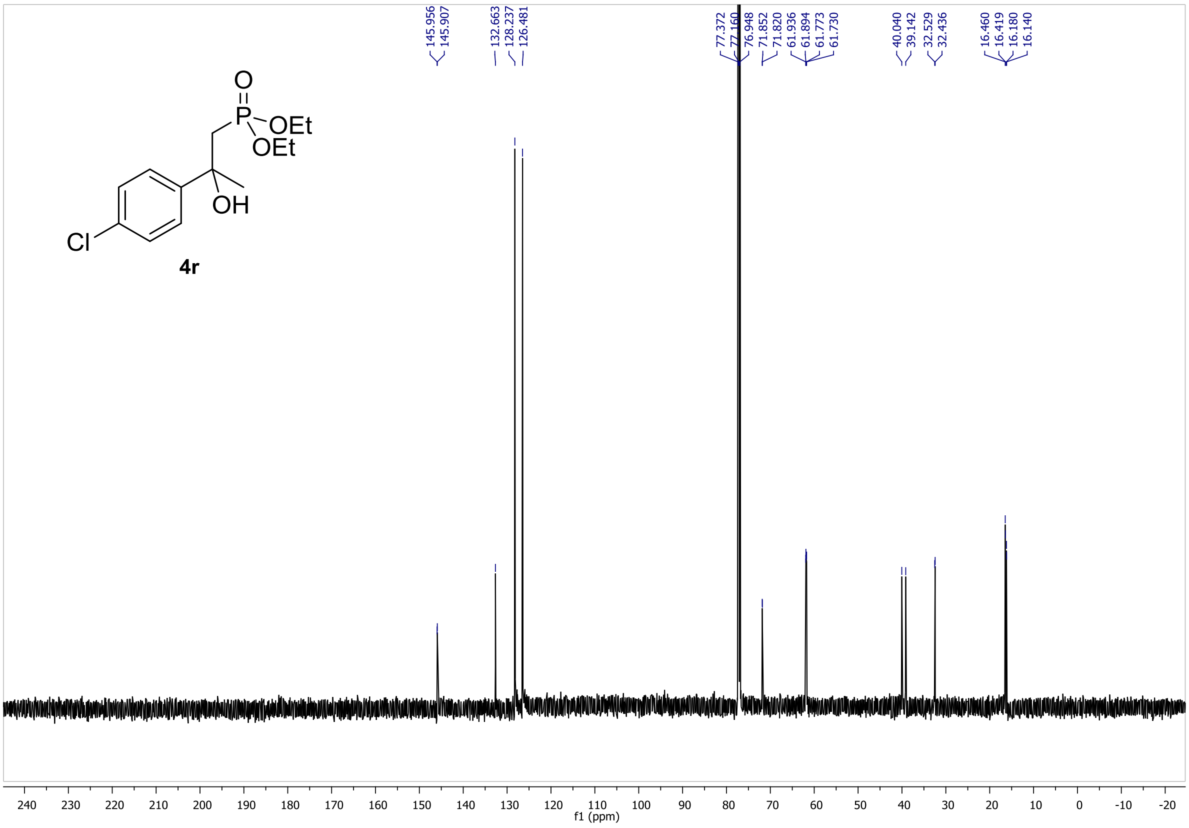

^{31}P NMR, 245 MHz, $CDCl_3$:

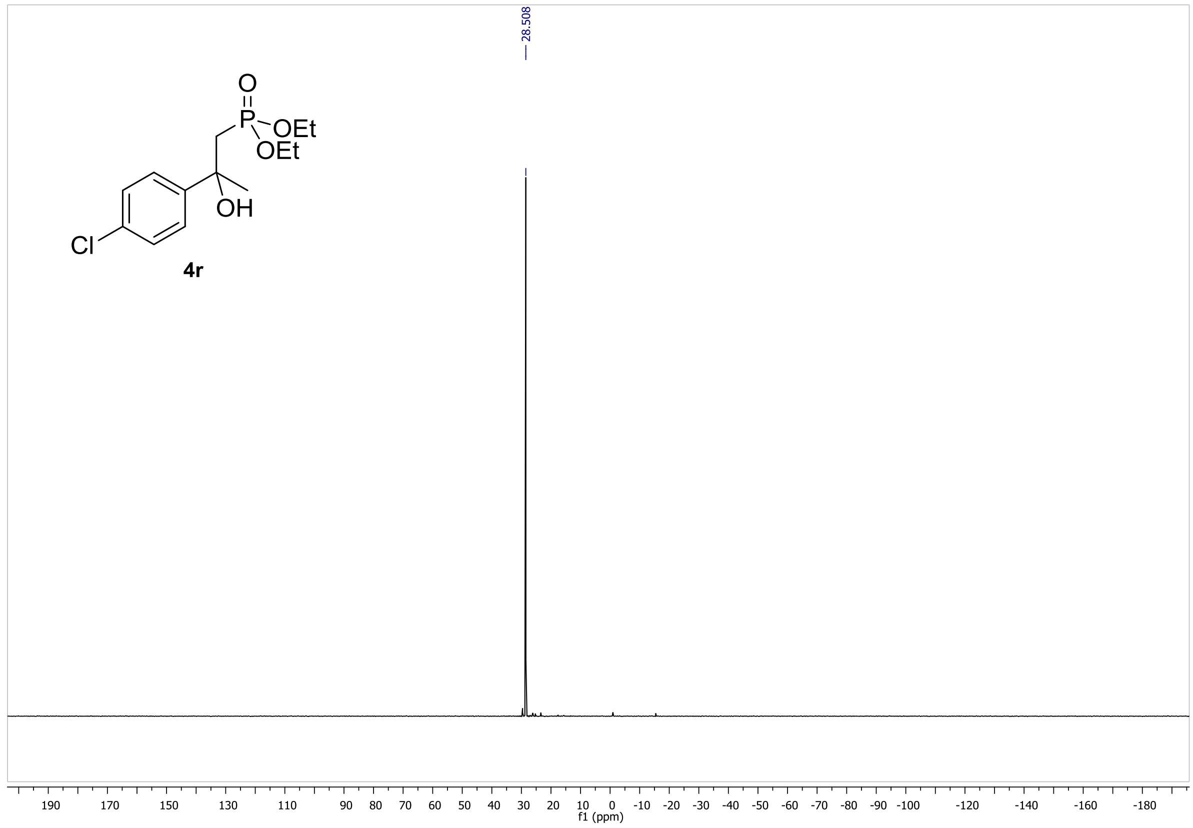

4-(4-chlorophenyl)-4-hydroxytetrahydro-2*H*-thiopyran 1,1-dioxide (4s):

^{1}H NMR, 600 MHz, $CDCl_3$:

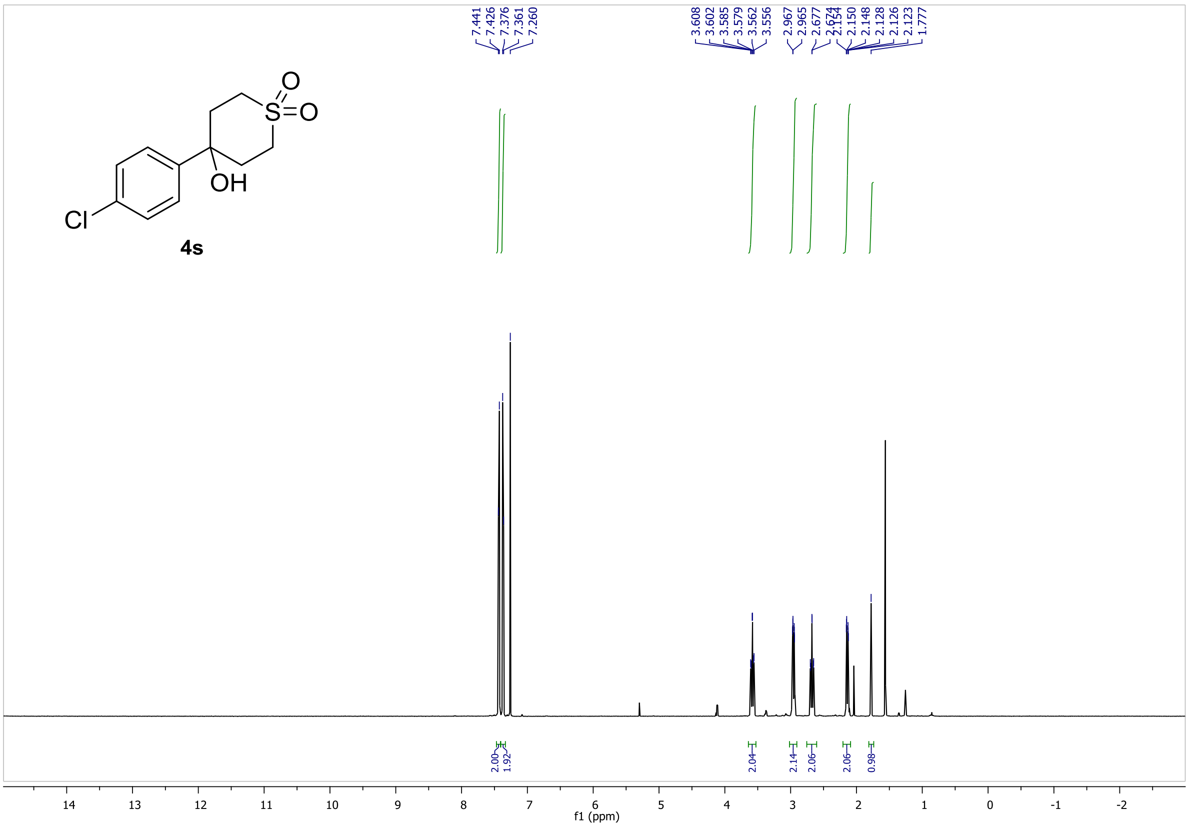

^{13}C NMR, 150 MHz, $CDCl_3$:

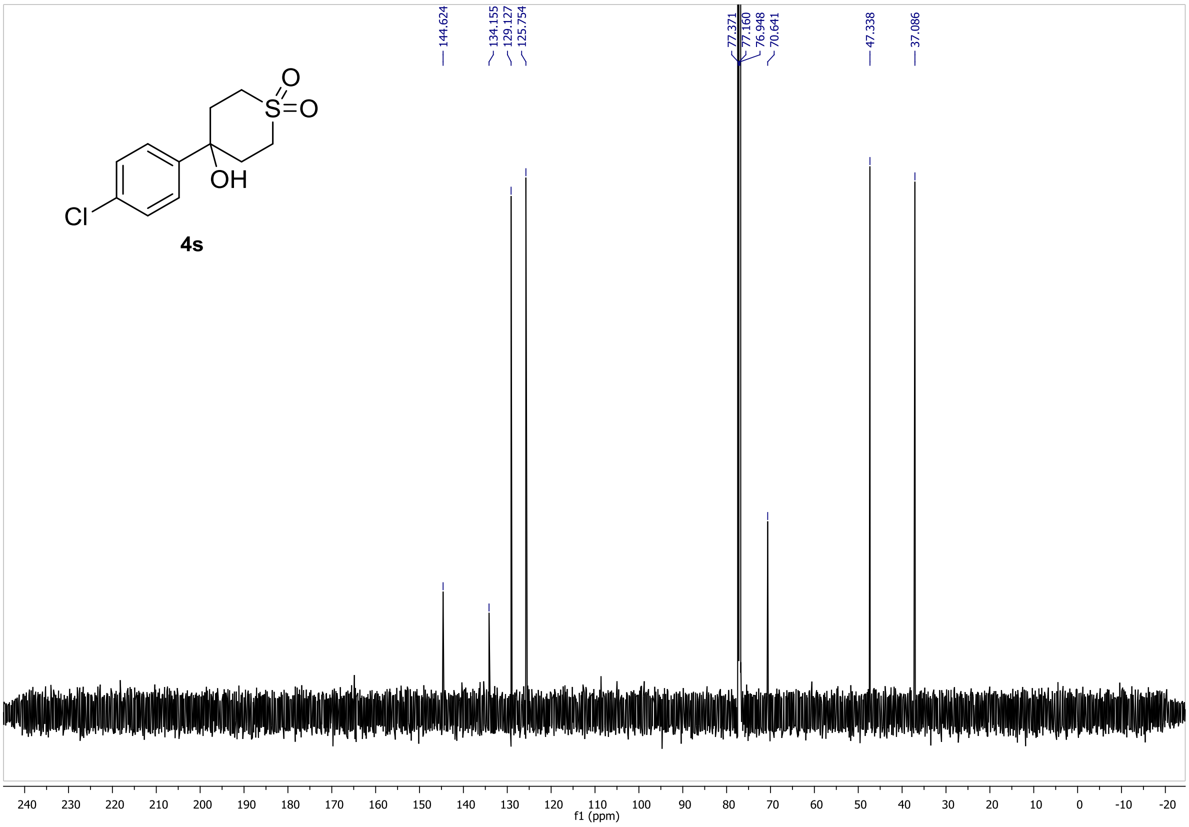

6-(4-chlorophenyl)-2,2-dimethyl-1-oxaspiro[2.5]octan-6-ol (4t):
Diastereomer A:

^{1}H NMR, 600 MHz, $CDCl_3$:

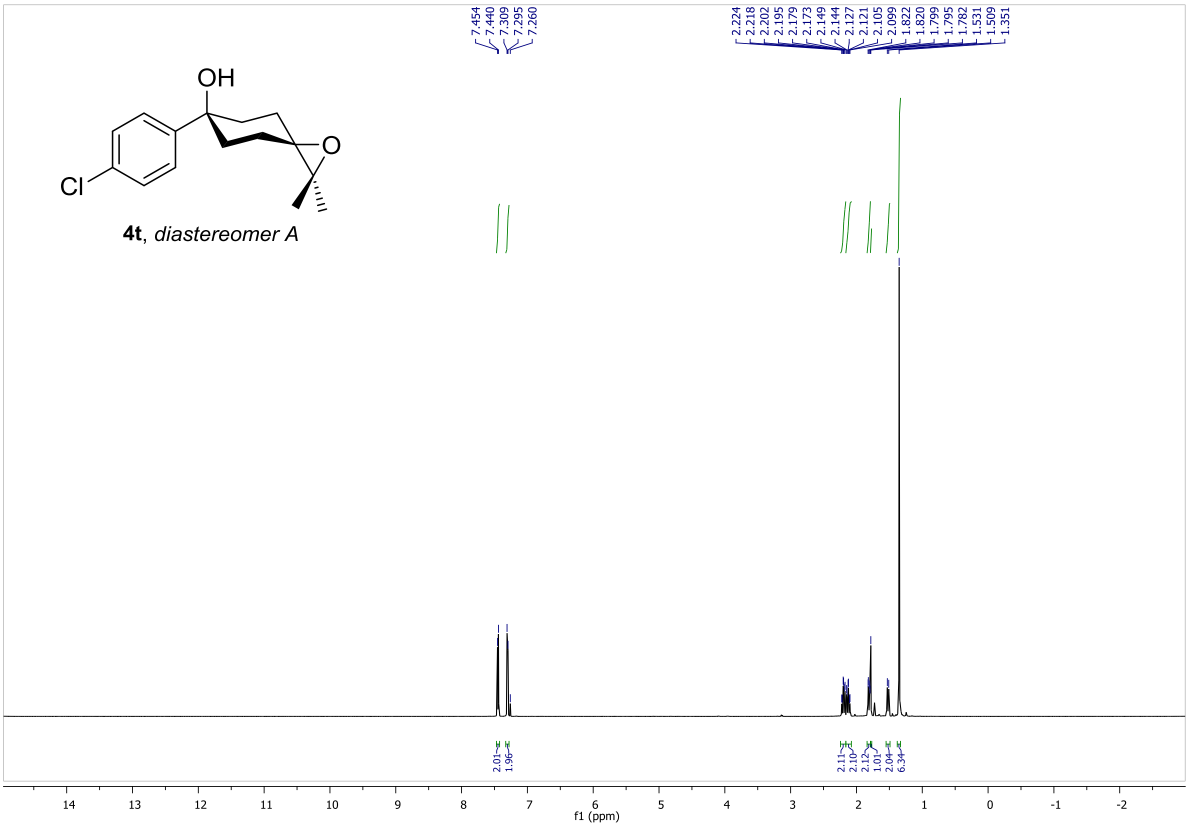

^{13}C NMR, 150 MHz, $CDCl_3$:

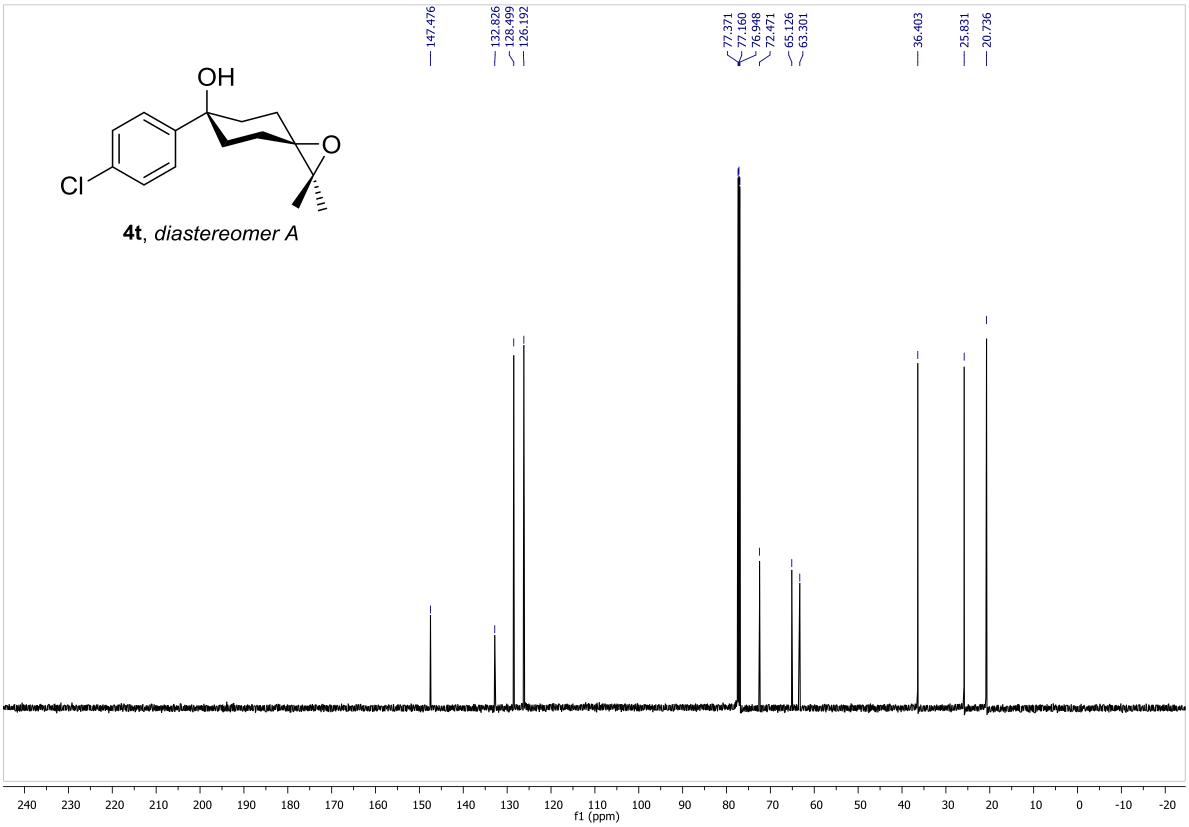

6-(4-chlorophenyl)-2,2-dimethyl-1-oxaspiro[2.5]octan-6-ol (4t):
Diastereomer B:

^{1}H NMR, 600 MHz, $CDCl_3$:

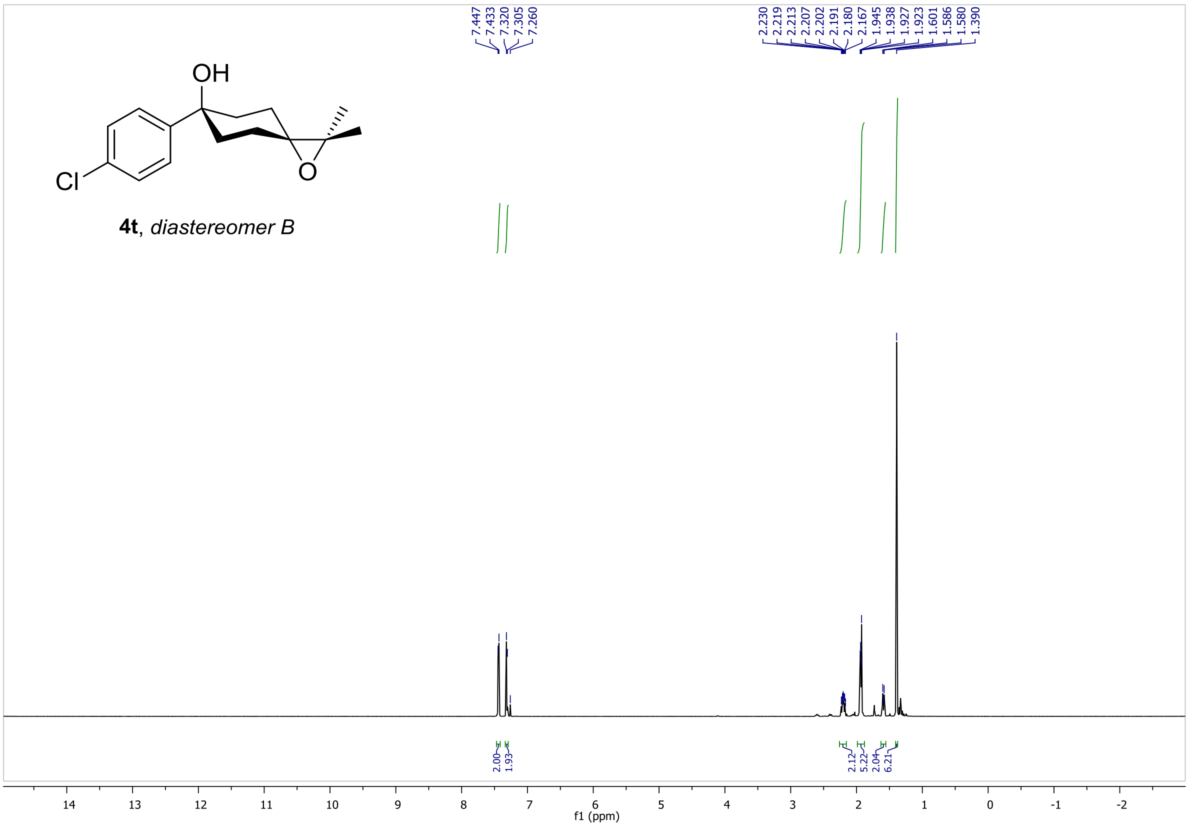

^{13}C NMR, 150 MHz, $CDCl_3$:

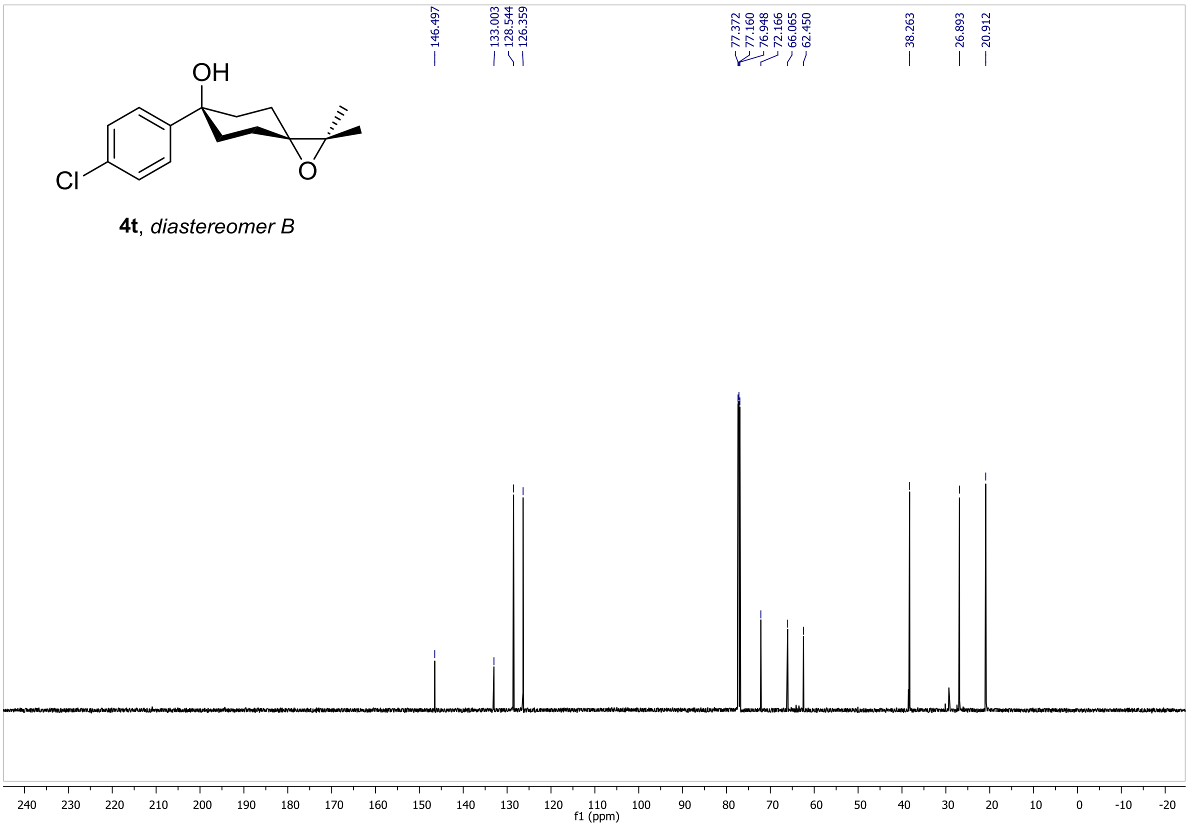

5-bromo-2-(4-chlorophenyl)pentan-2-ol (4u):

^{1}H NMR, 600 MHz, $CDCl_3$:

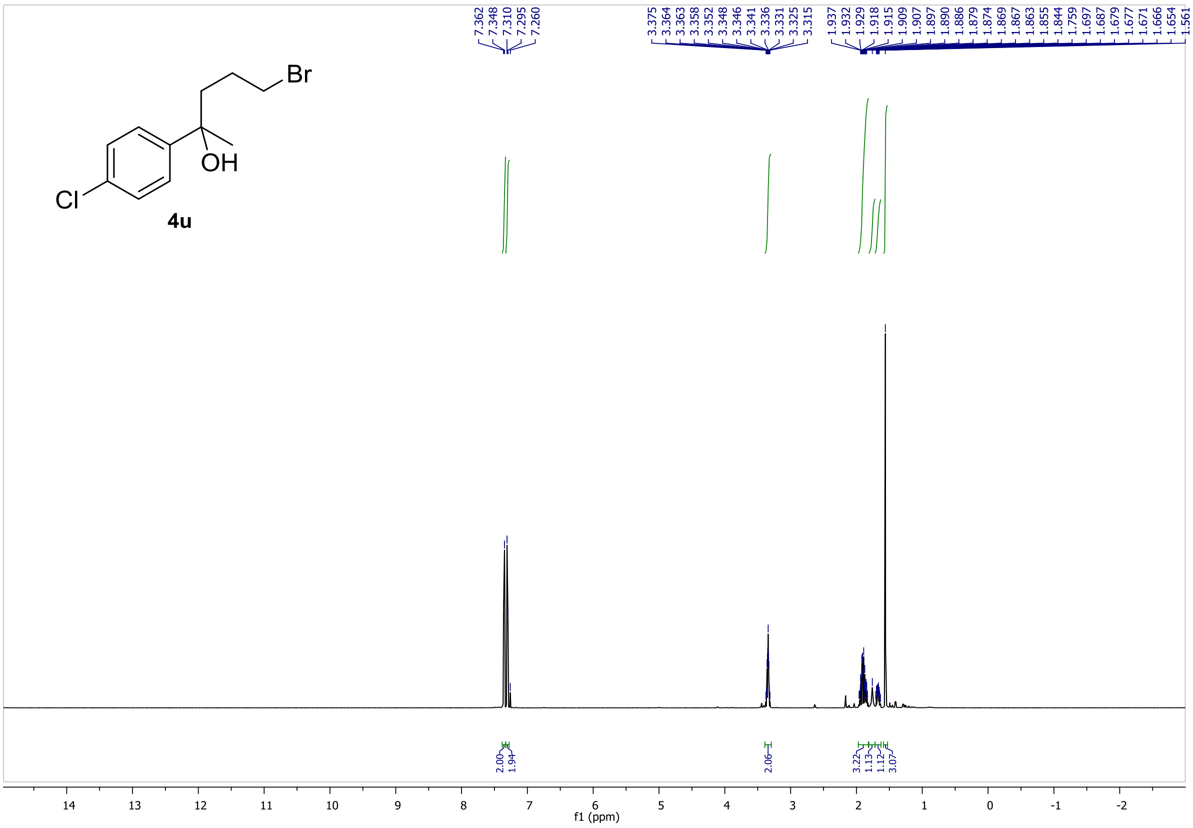

^{13}C NMR, 150 MHz, $CDCl_3$:

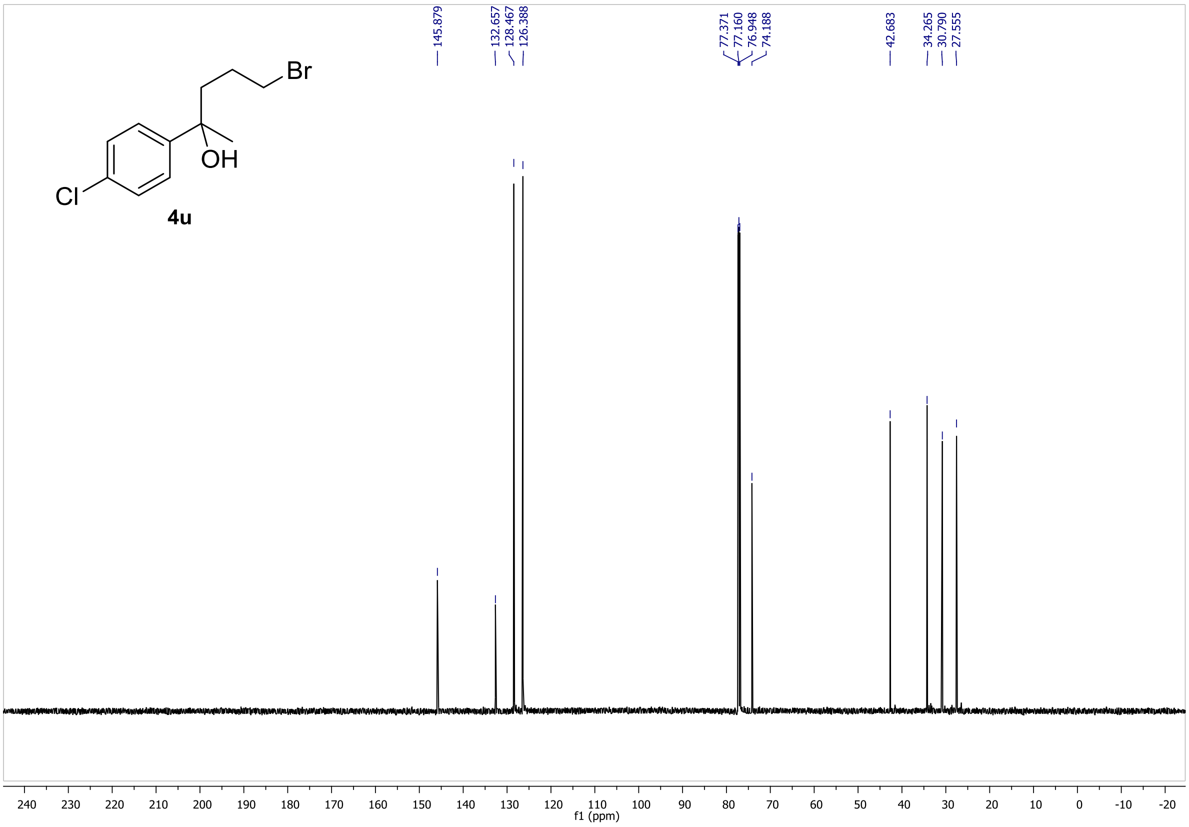

2-(4-chlorophenyl)-1-(furan-2-yl)propan-2-ol (4v):

^{1}H NMR, 600 MHz, $CDCl_3$:

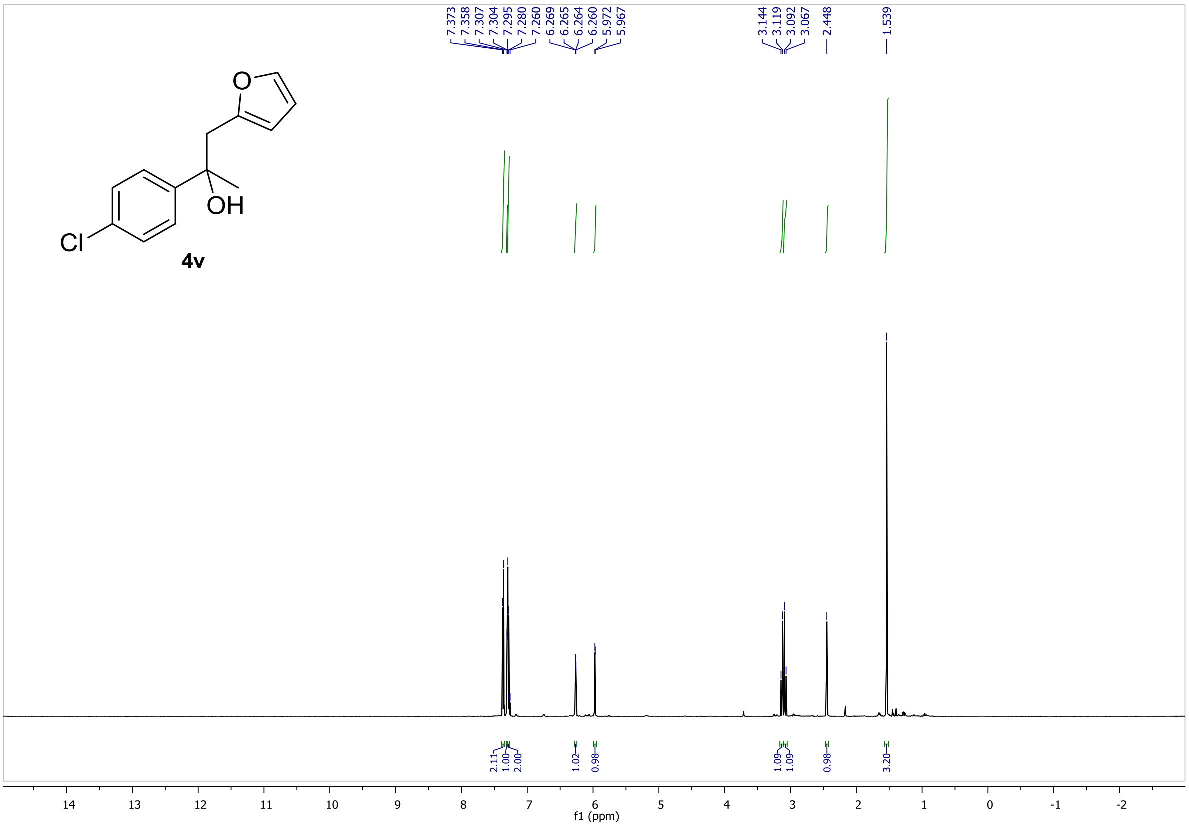

^{13}C NMR, 150 MHz, $CDCl_3$:

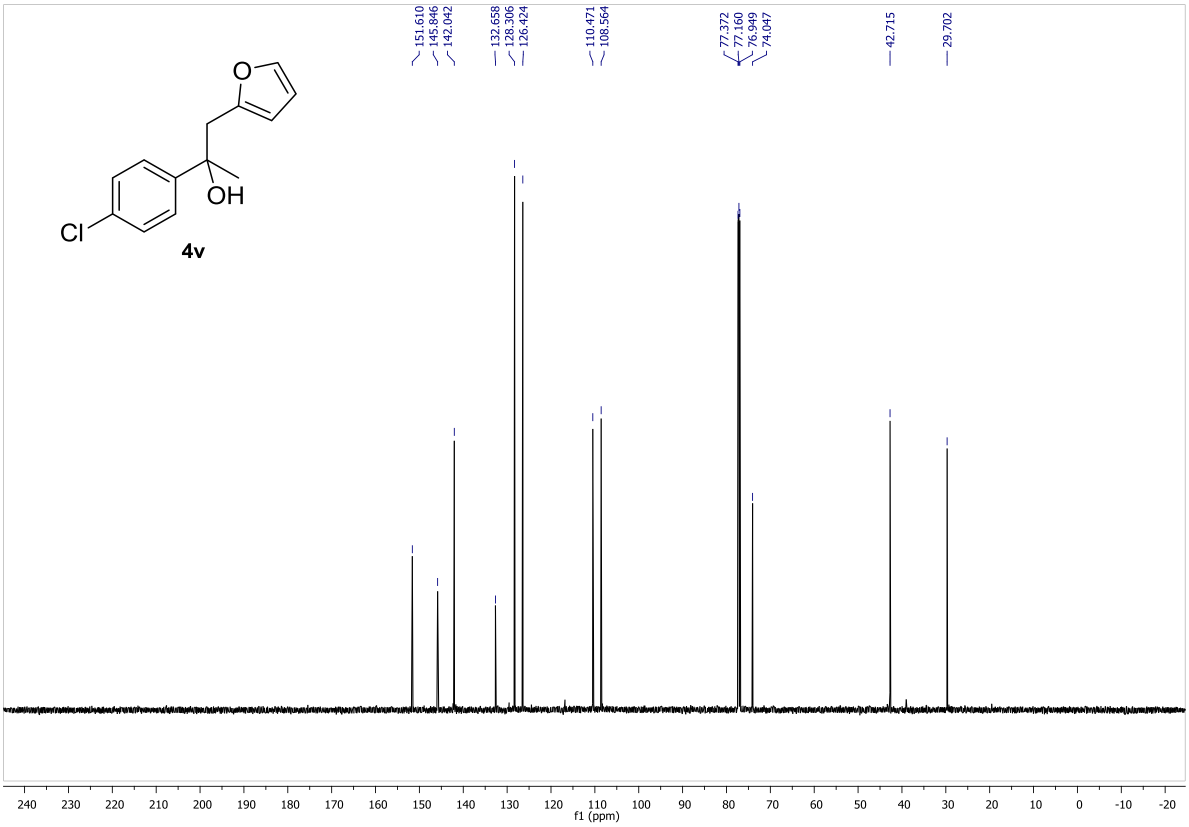

4-(4-chlorophenyl)-1-(pyrimidin-2-yl)piperidin-4-ol (4w):

^{1}H NMR, 600 MHz, $CDCl_3$:

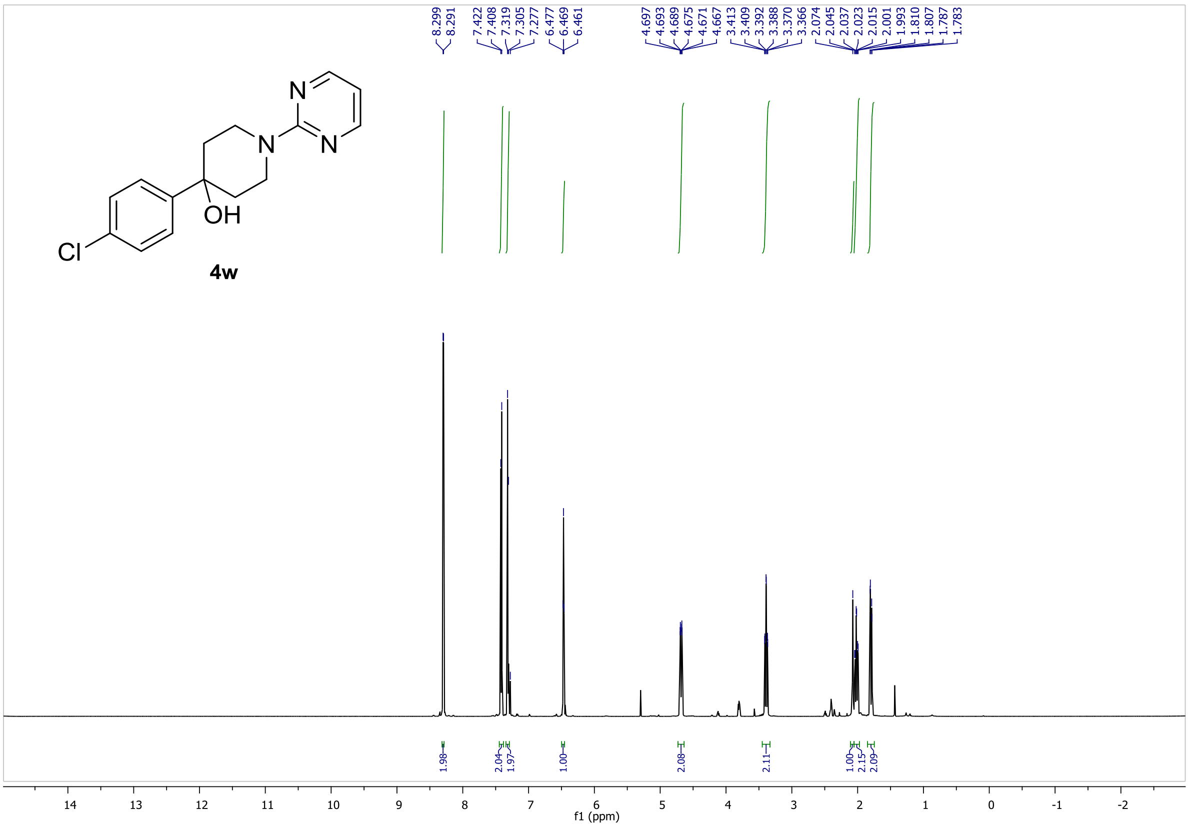

^{13}C NMR, 150 MHz, $CDCl_3$:

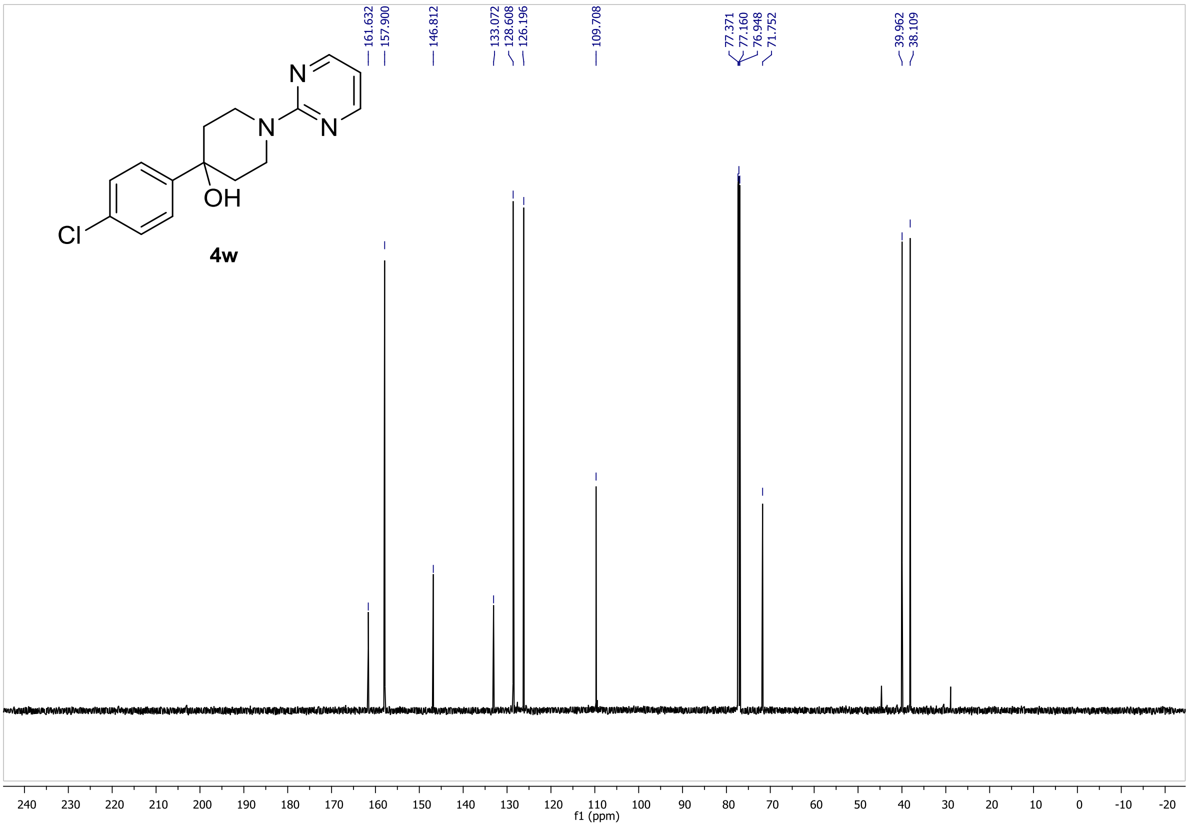

4-(4-bromophenyl)tetrahydro-2*H*-pyran-4-ol (4x):

^{1}H NMR, 600 MHz, $CDCl_3$:

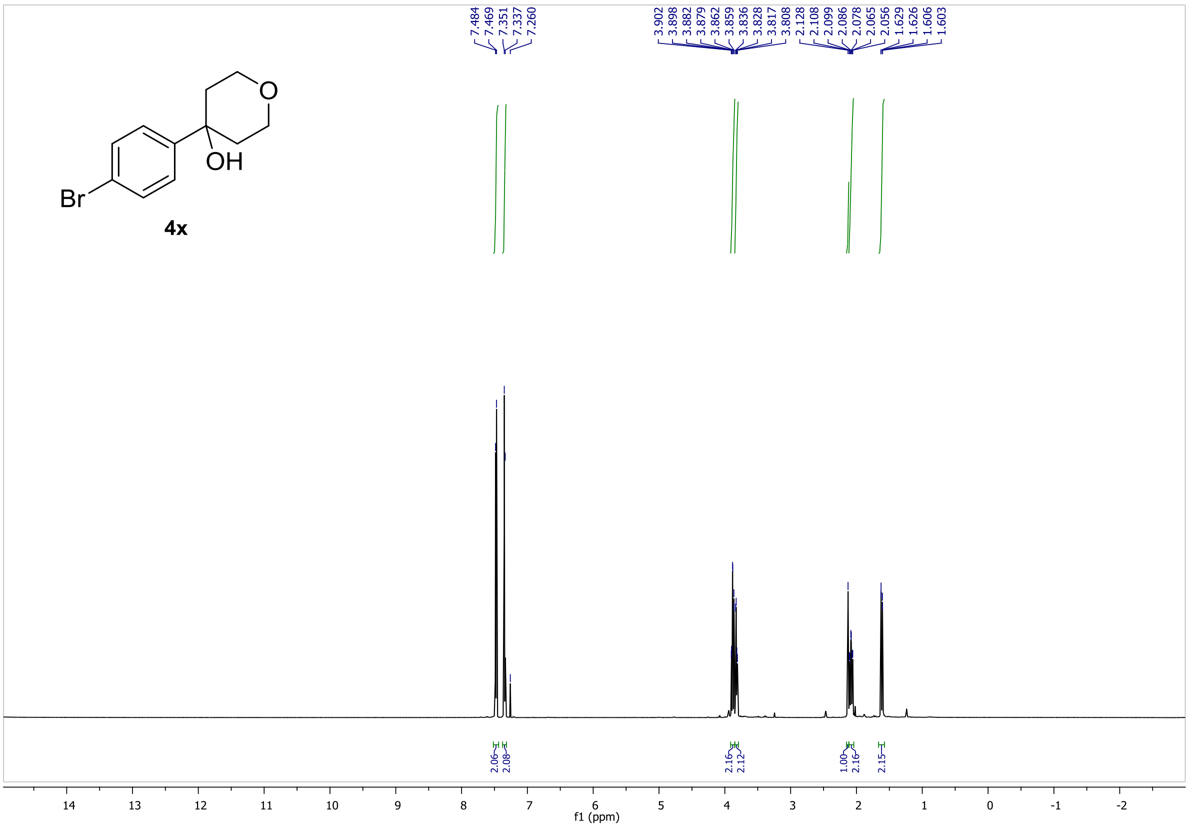

^{13}C NMR, 150 MHz, $CDCl_3$:

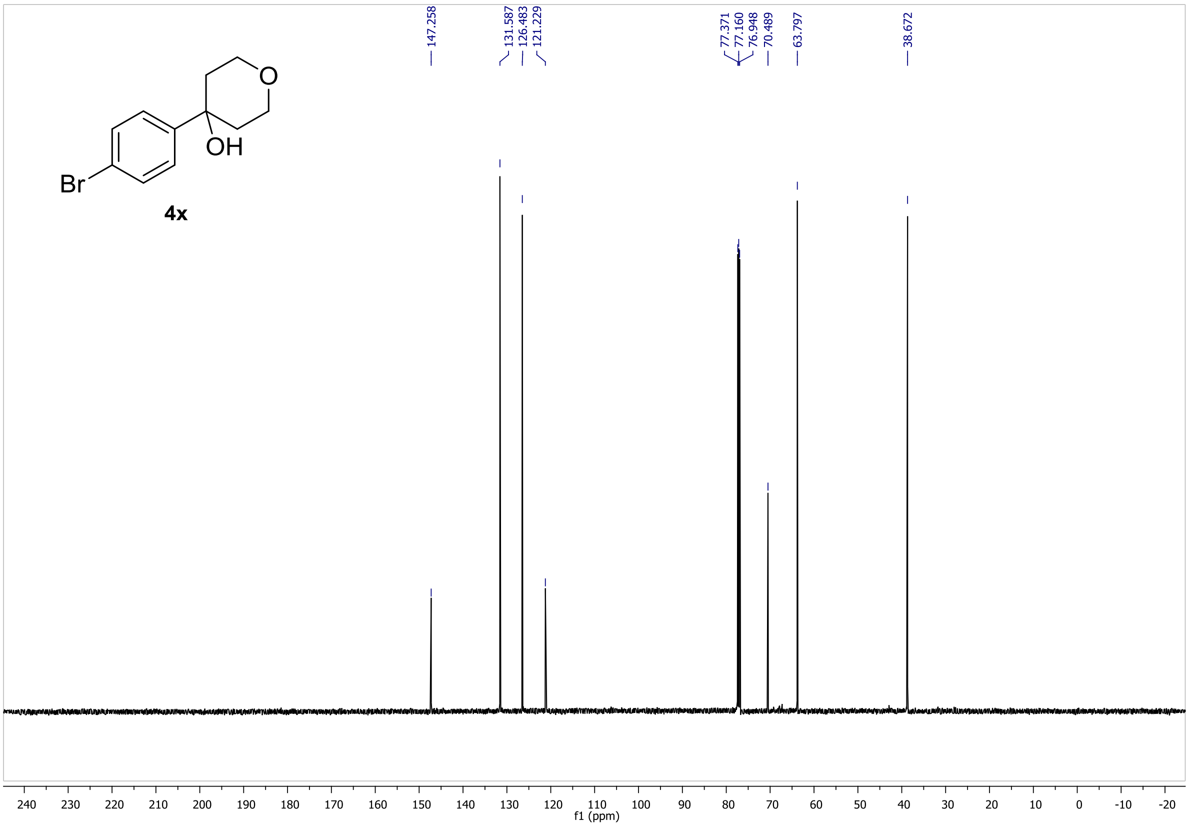

4-(4-(trifluoromethyl)phenyl)tetrahydro-2*H*-pyran-4-ol (4y):

^{1}H NMR, 600 MHz, $CDCl_3$:

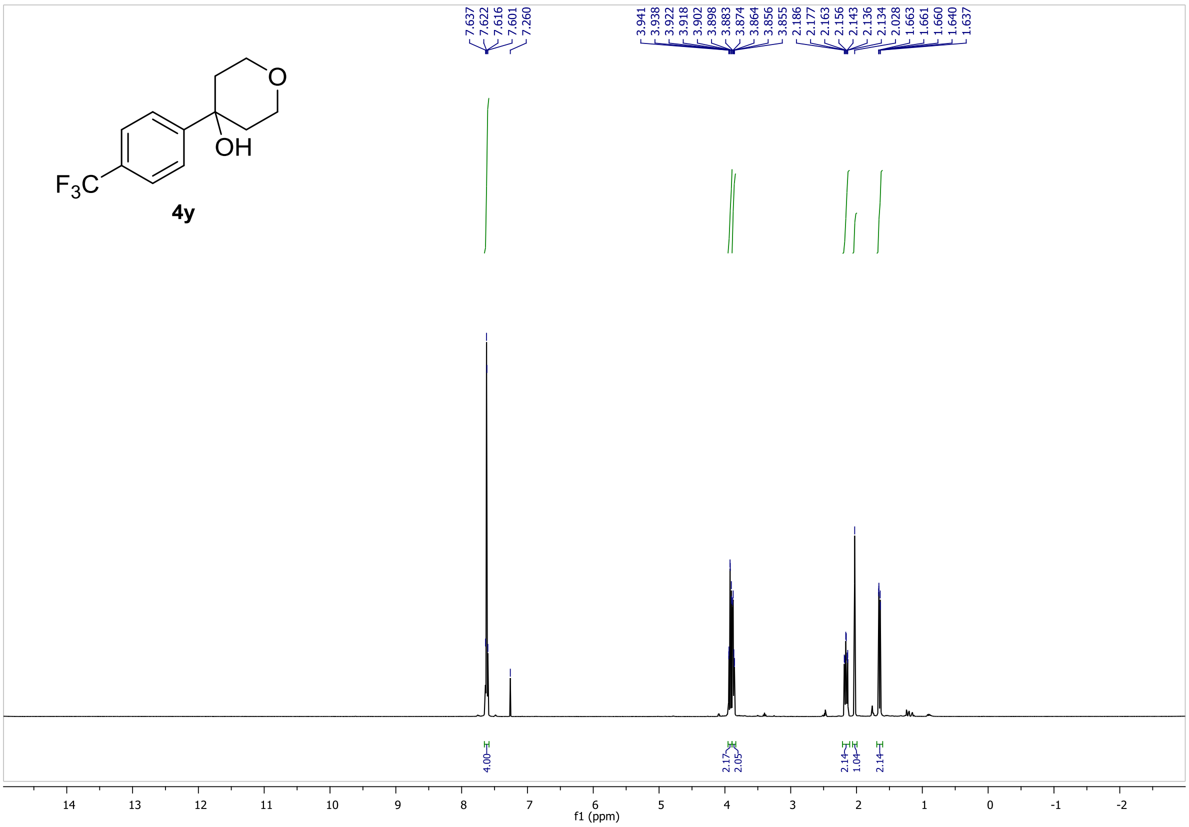

^{13}C NMR, 150 MHz, $CDCl_3$:

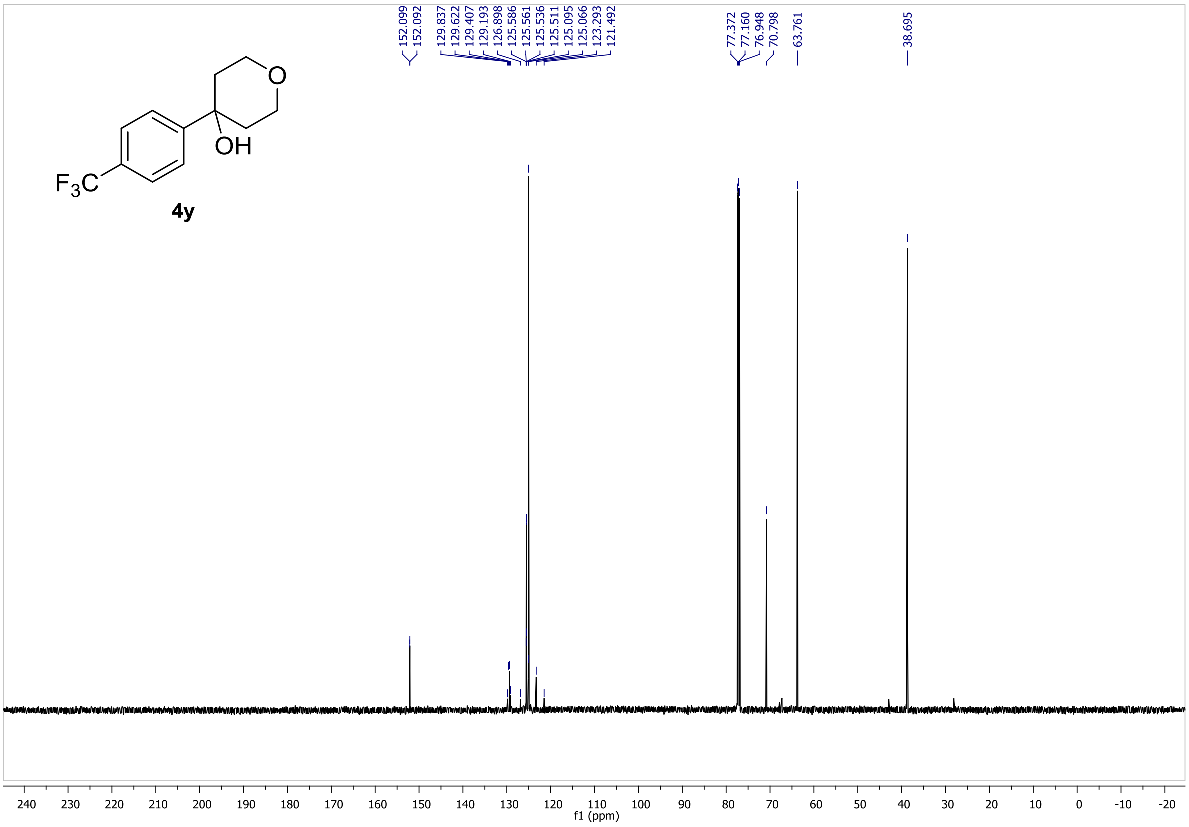

^{19}F NMR, 376 MHz, $CDCl_3$:

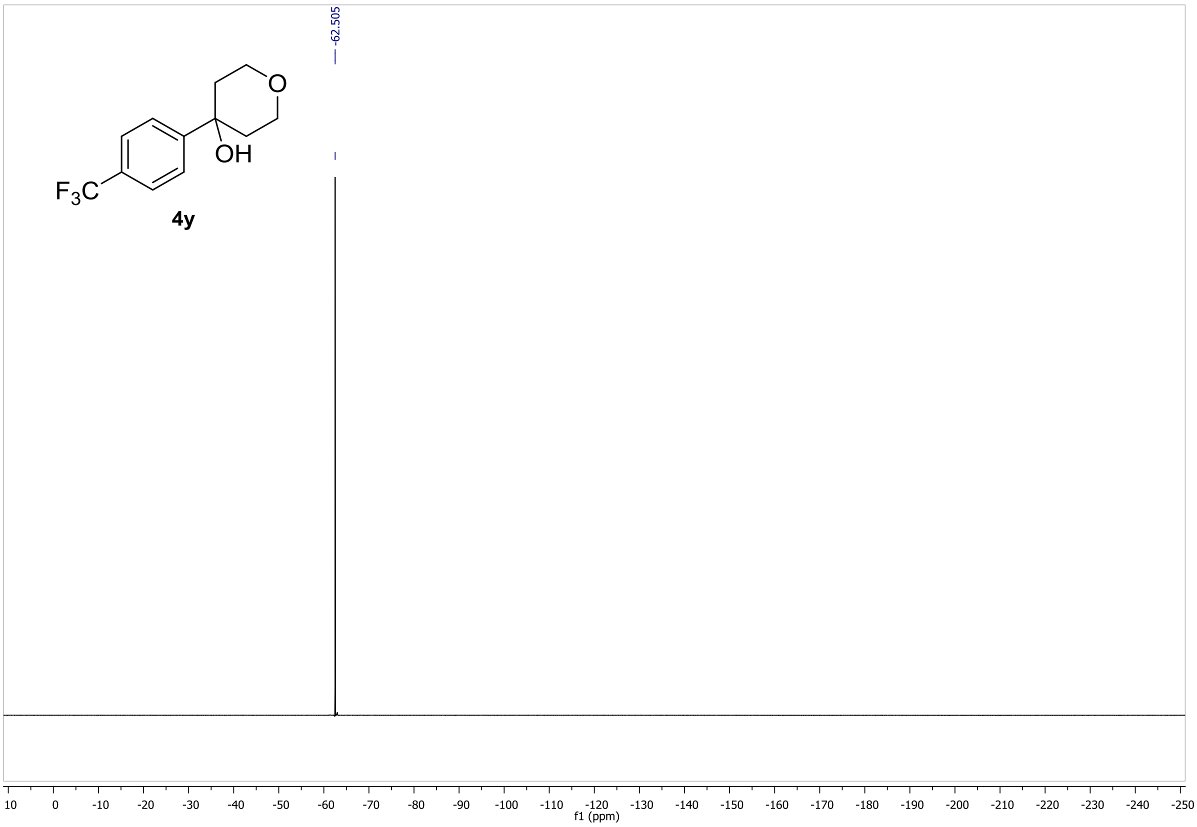

Methyl 4-(4-hydroxytetrahydro-2*H*-pyran-4-yl)benzoate (4aa):

^{1}H NMR, 600 MHz, $CDCl_3$:

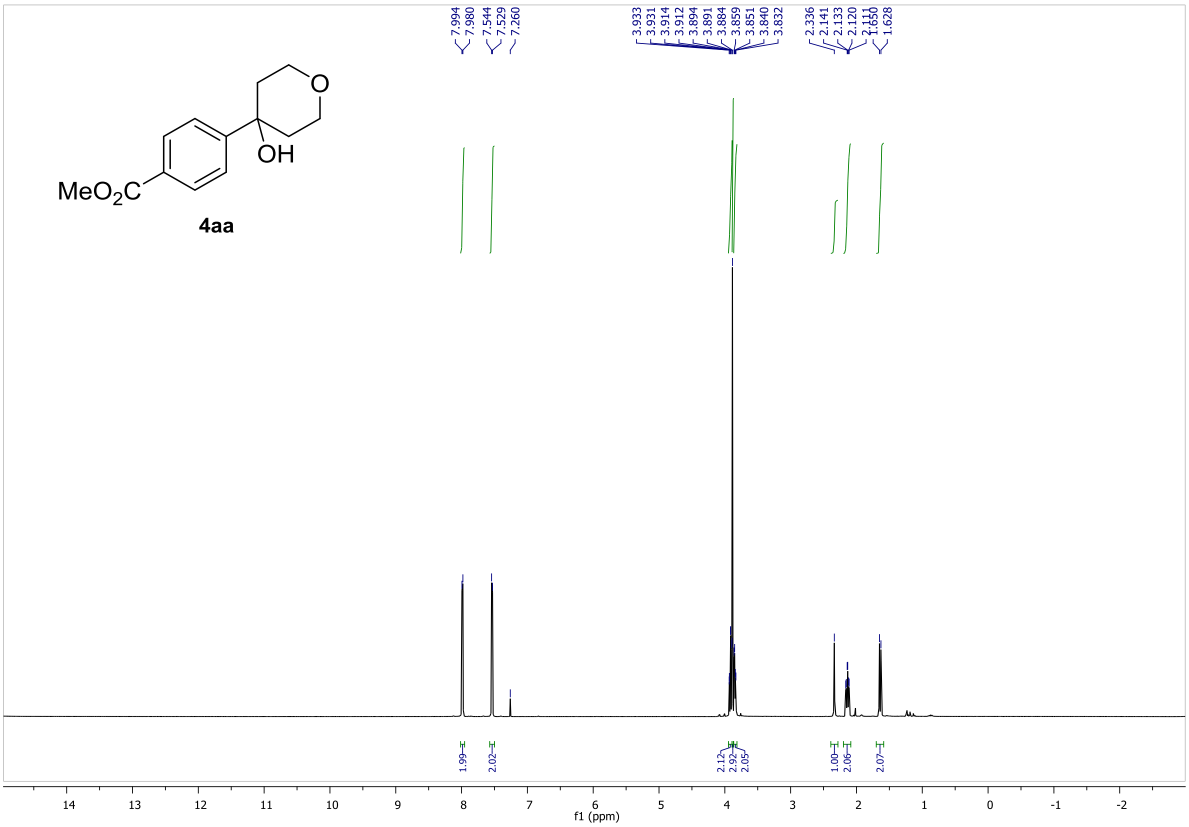

^{13}C NMR, 150 MHz, $CDCl_3$:

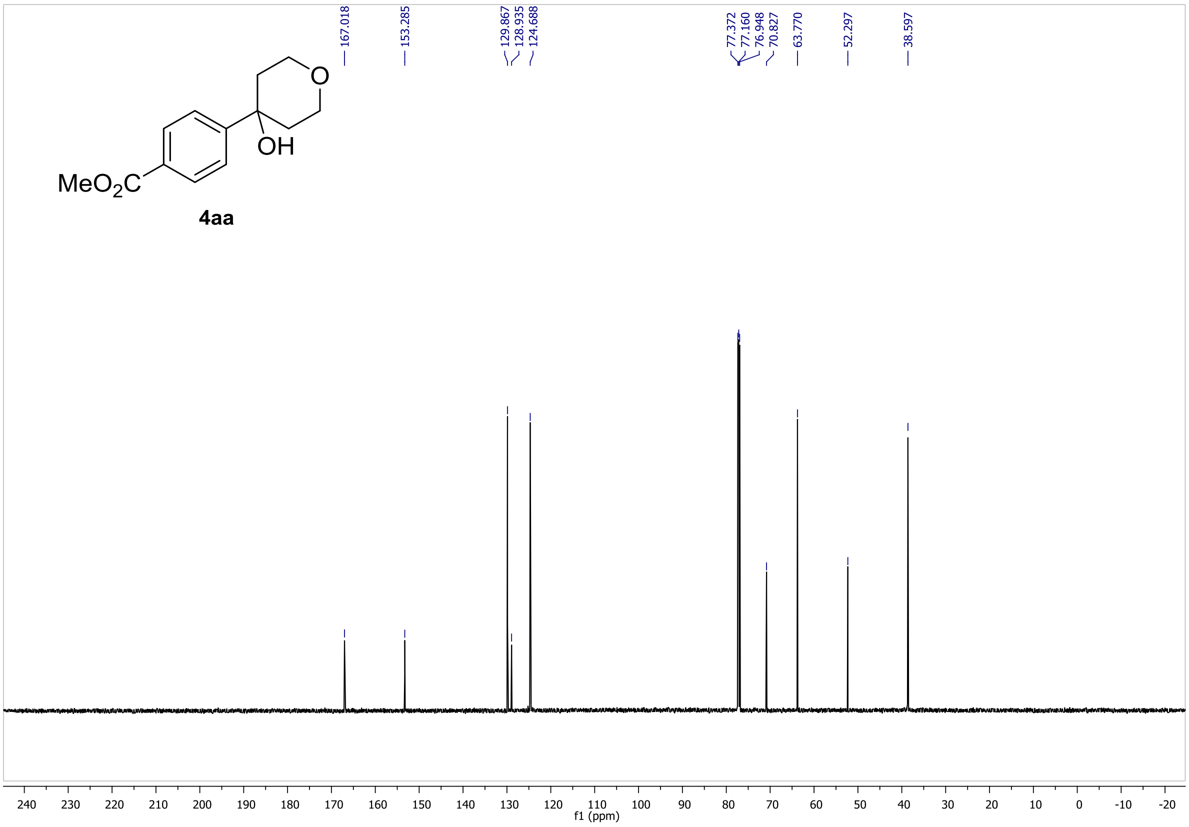

***N*-(3-(4-hydroxytetrahydro-2*H*-pyran-4-yl)phenyl)acetamide (4ab):**

^{1}H NMR, 600 MHz, MeOD-d_4:

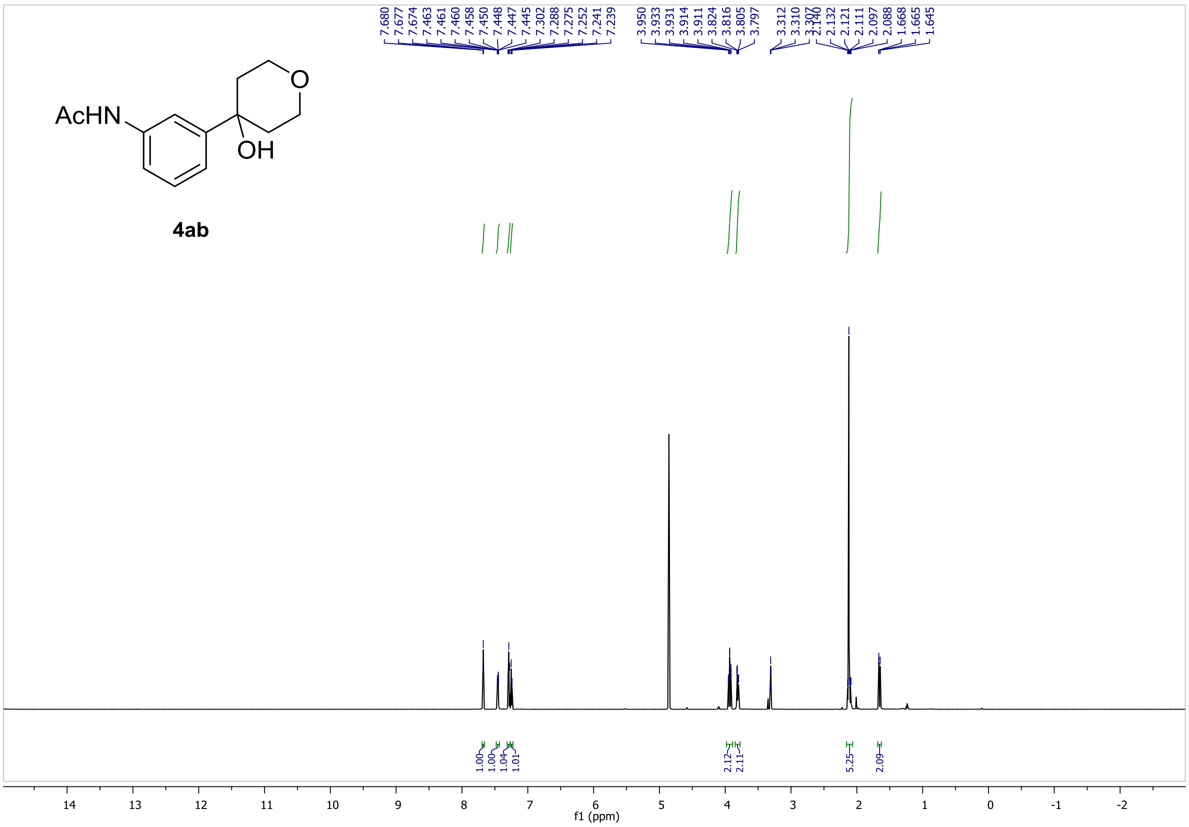

^{13}C NMR, 150 MHz, MeOD-d_4:

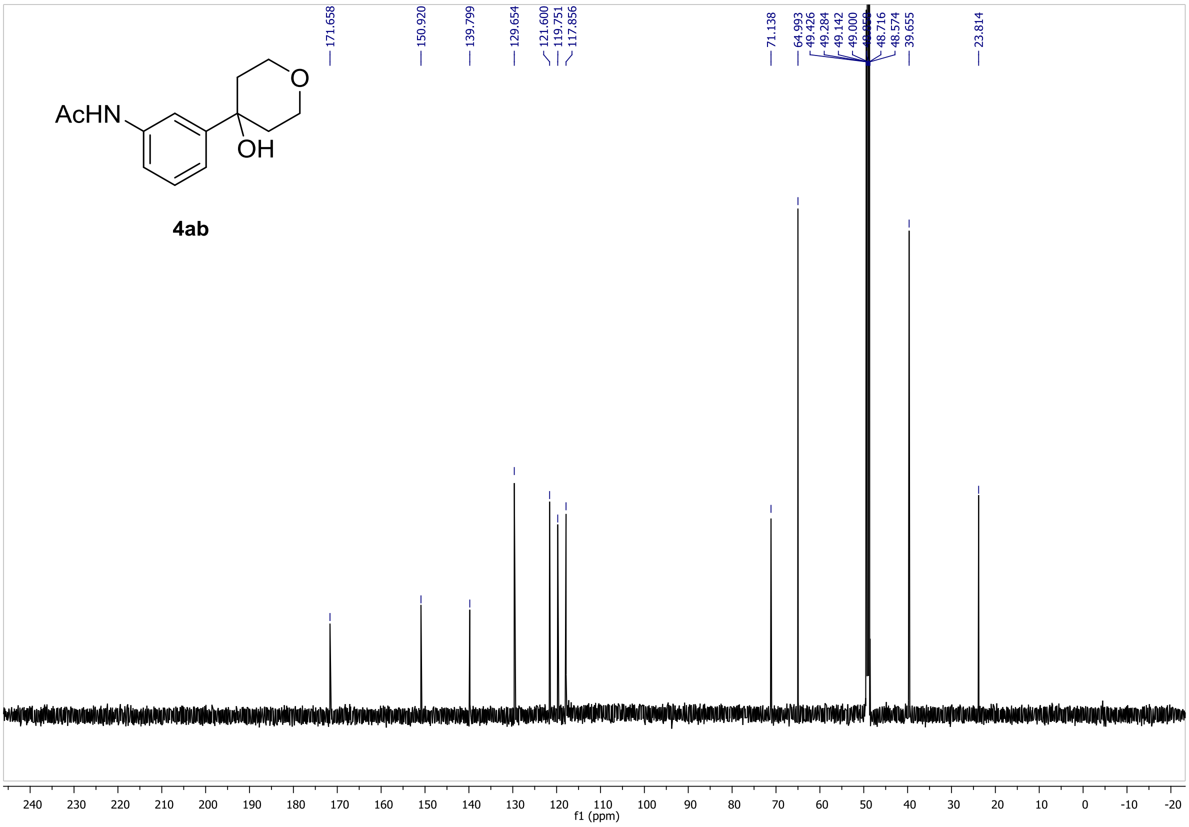

8.4. NMR spectra for compound 5 and derivatised products

2-(1-(4-chlorophenyl)cyclobutyl)-4,4,5,5-tetramethyl-1,3,2-dioxaborolane (5):

^{1}H NMR, 600 MHz, $CDCl_3$:

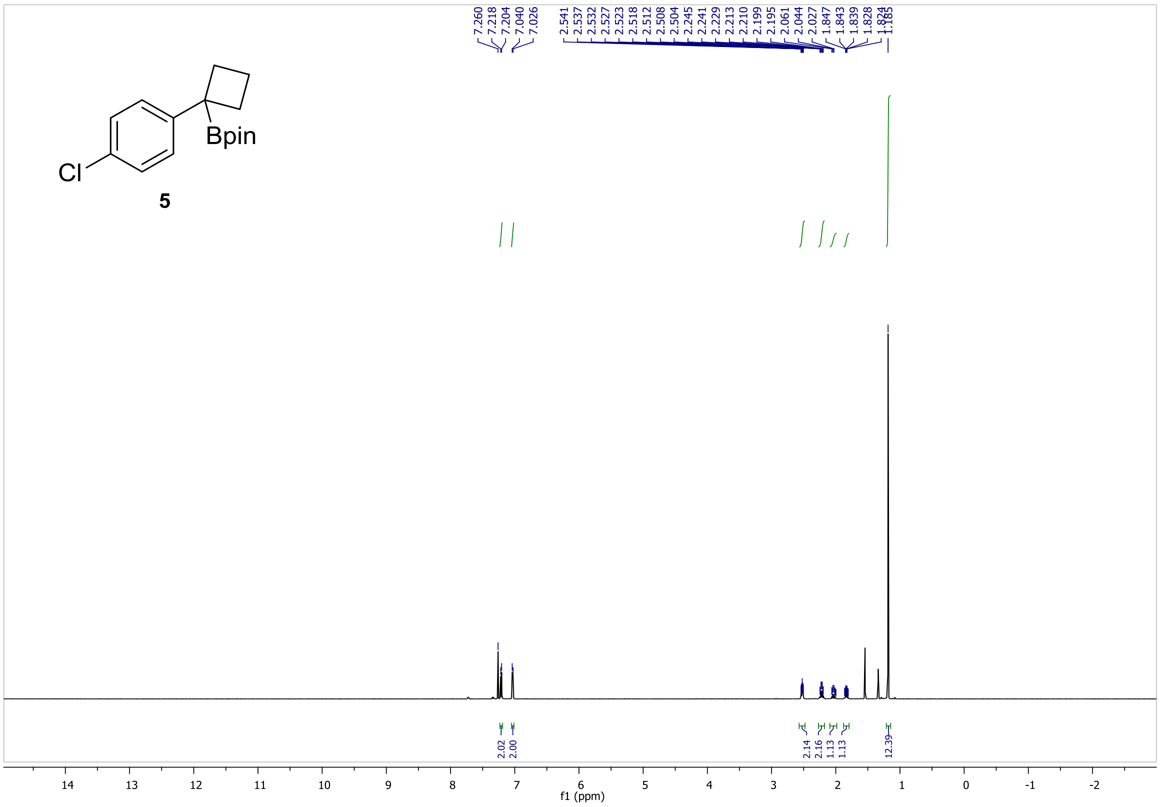

^{13}C NMR, 150 MHz, $CDCl_3$:

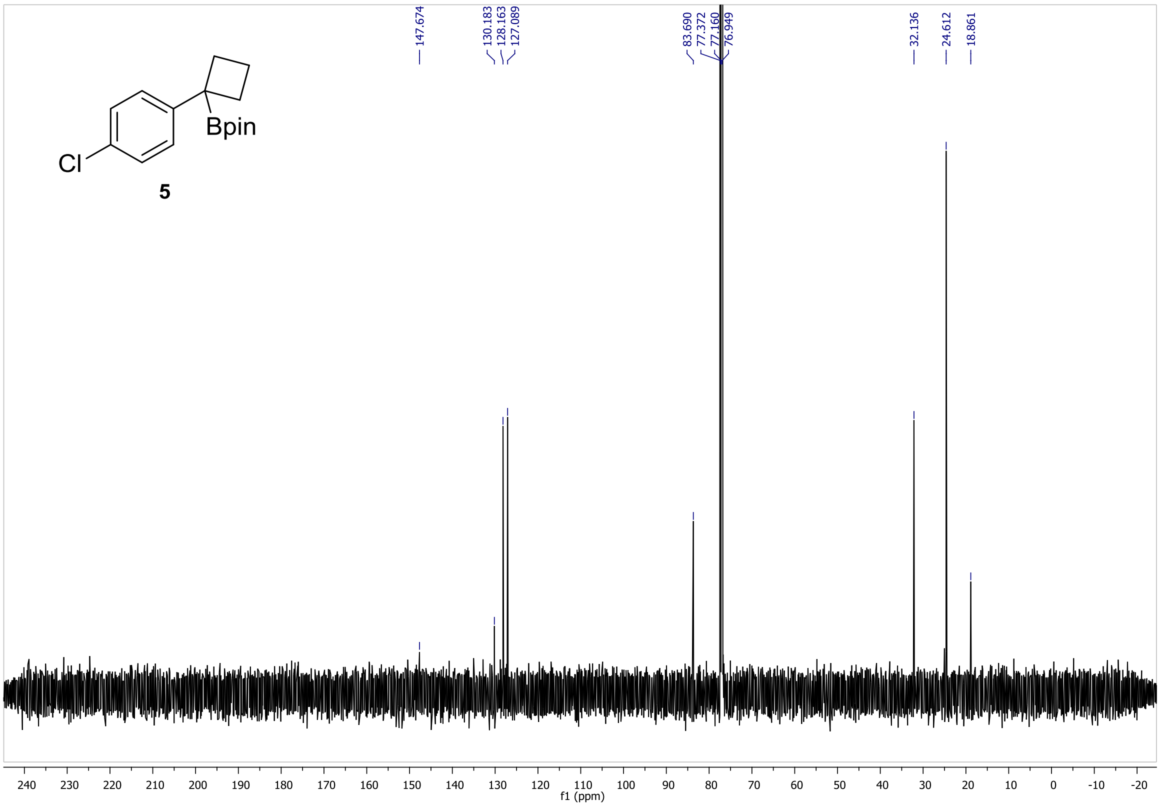

Potassium (1-(4-chlorophenyl)cyclobutyl)trifluoroborate:

^{1}H NMR, 600 MHz, MeCN-d_3:

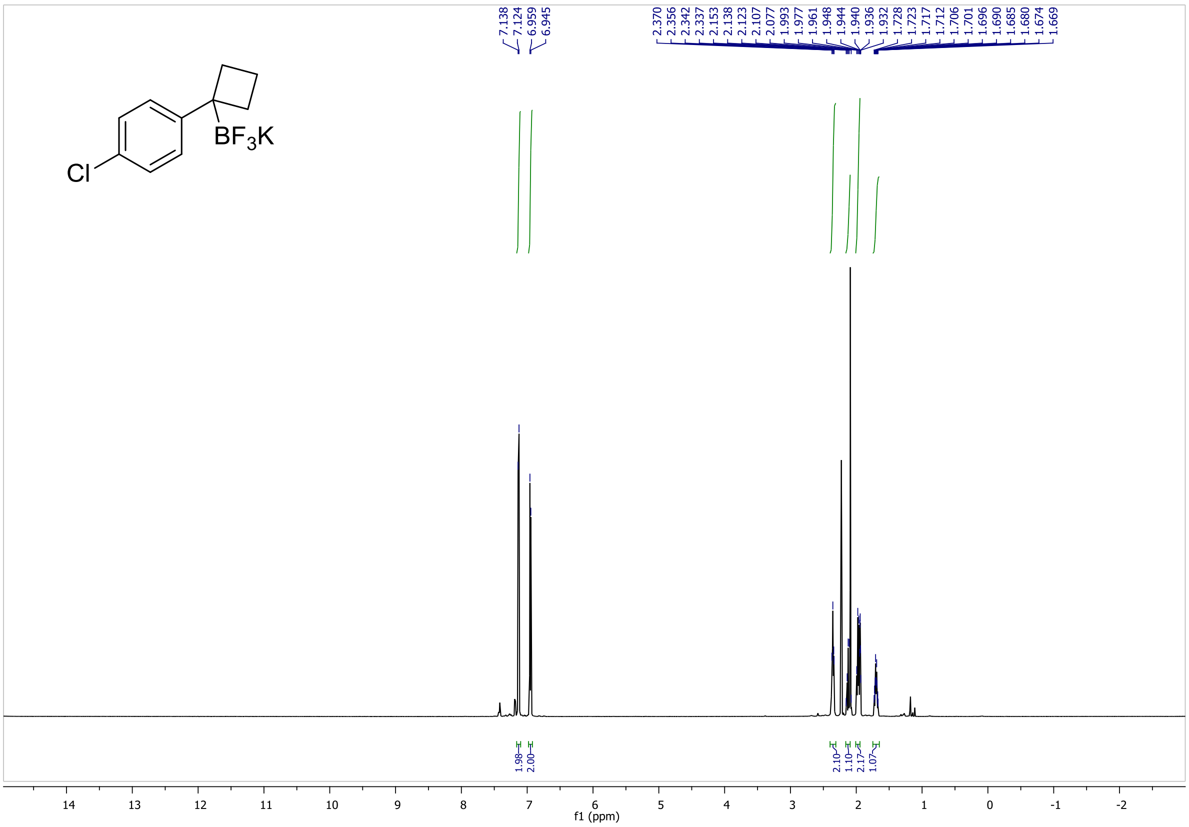

^{13}C NMR, 150 MHz, MeCN-d_3:

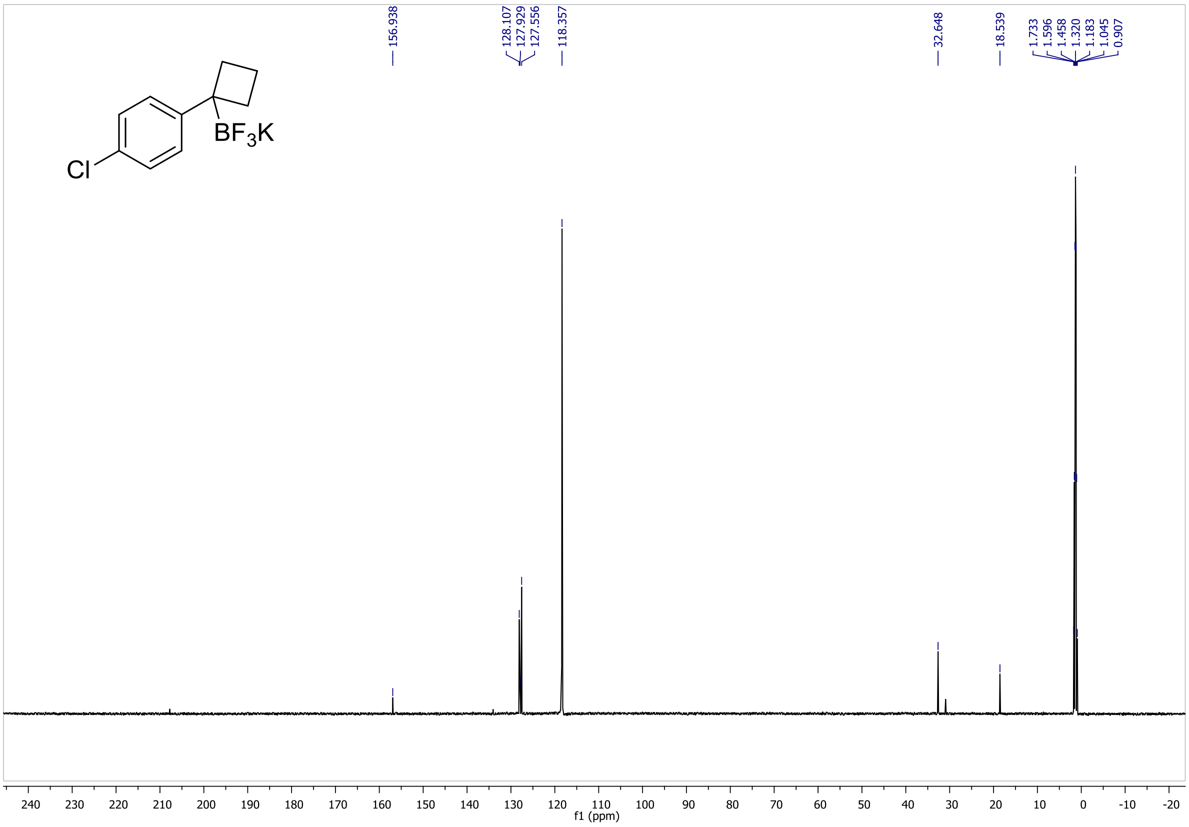

^{19}F NMR, 376 MHz, MeCN-d_3:

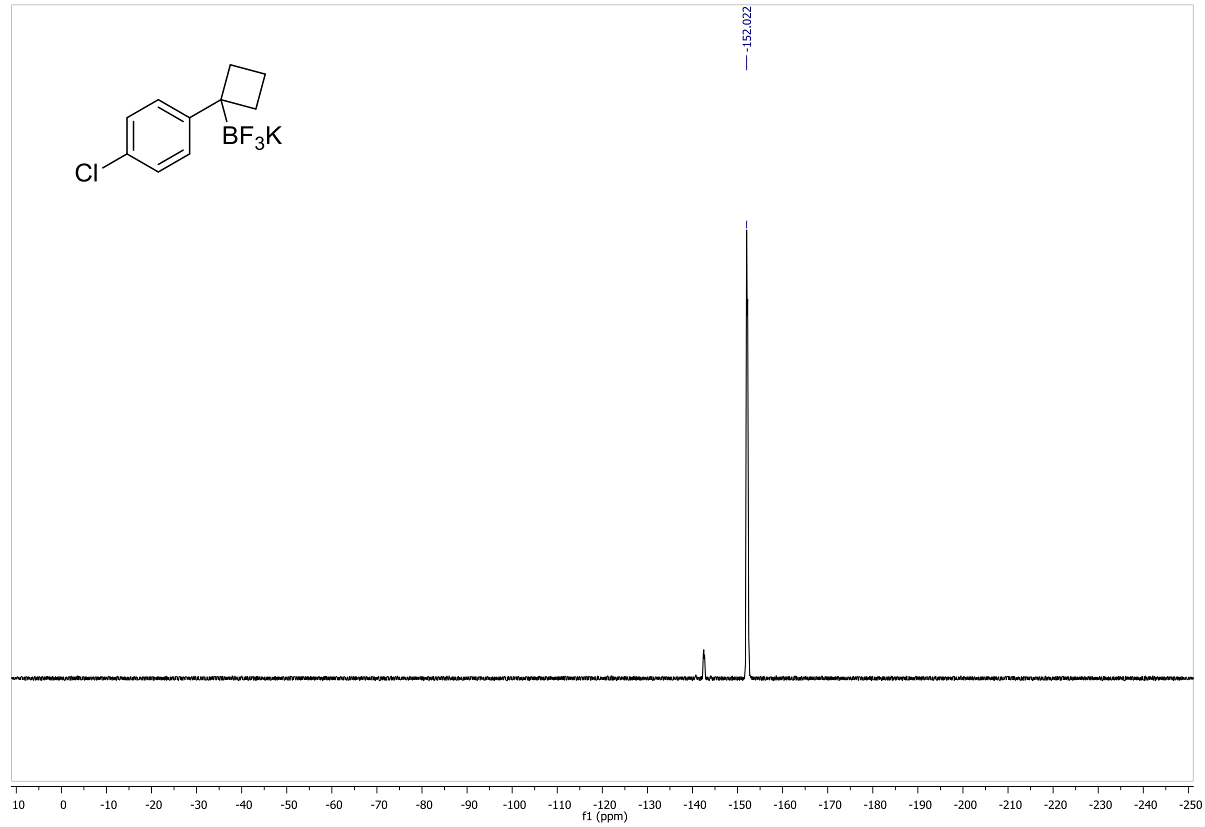

N-benzyl-1-(4-chlorophenyl)cyclobutan-1-amine (6):

^{1}H NMR, 600 MHz, $CDCl_3$:

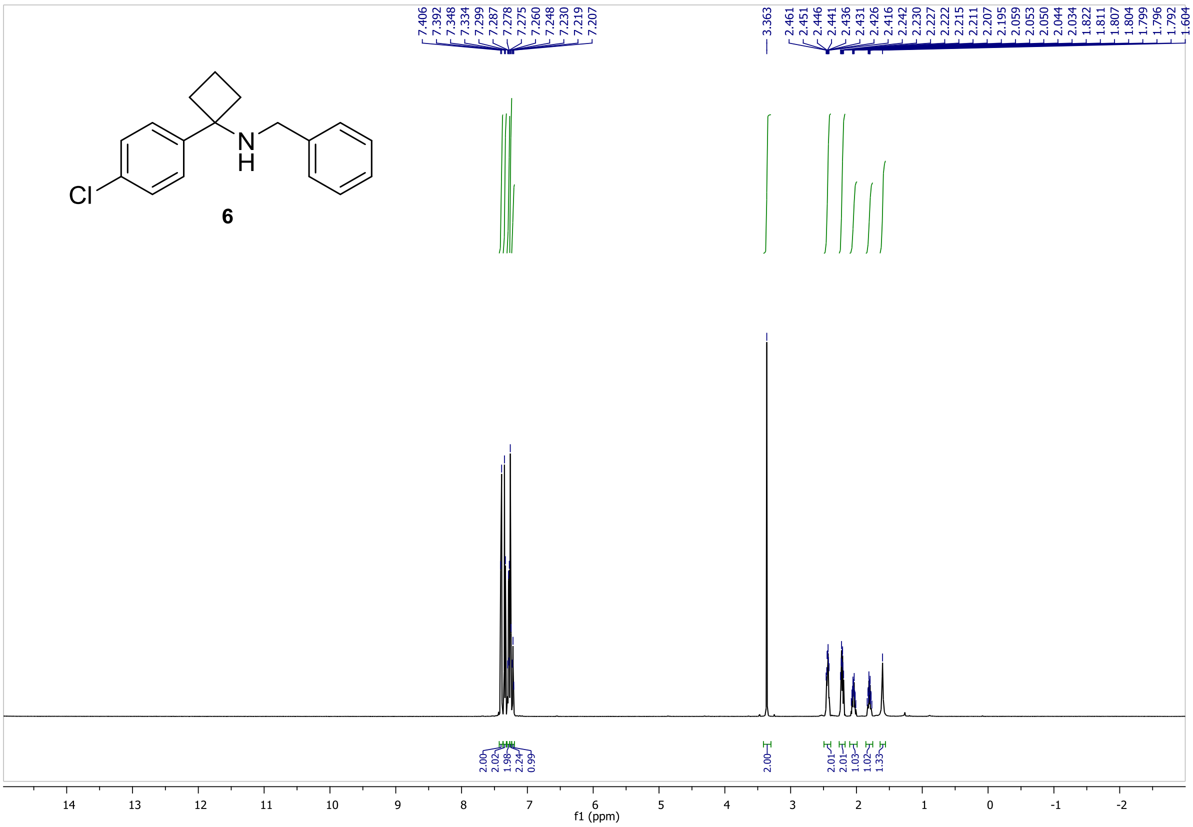

^{13}C NMR, 150 MHz, $CDCl_3$:

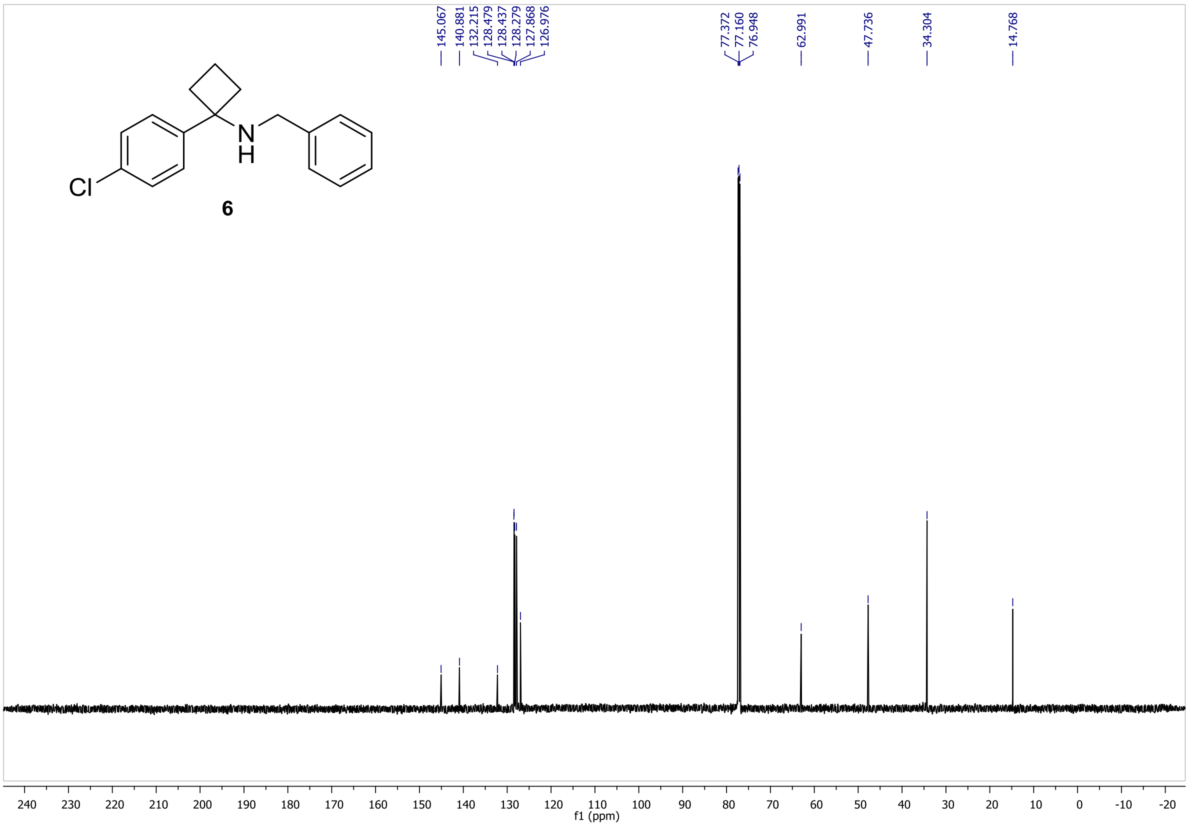

2-(1-(4-chlorophenyl)cyclobutyl)furan (7):

^{1}H NMR, 600 MHz, $CDCl_3$:

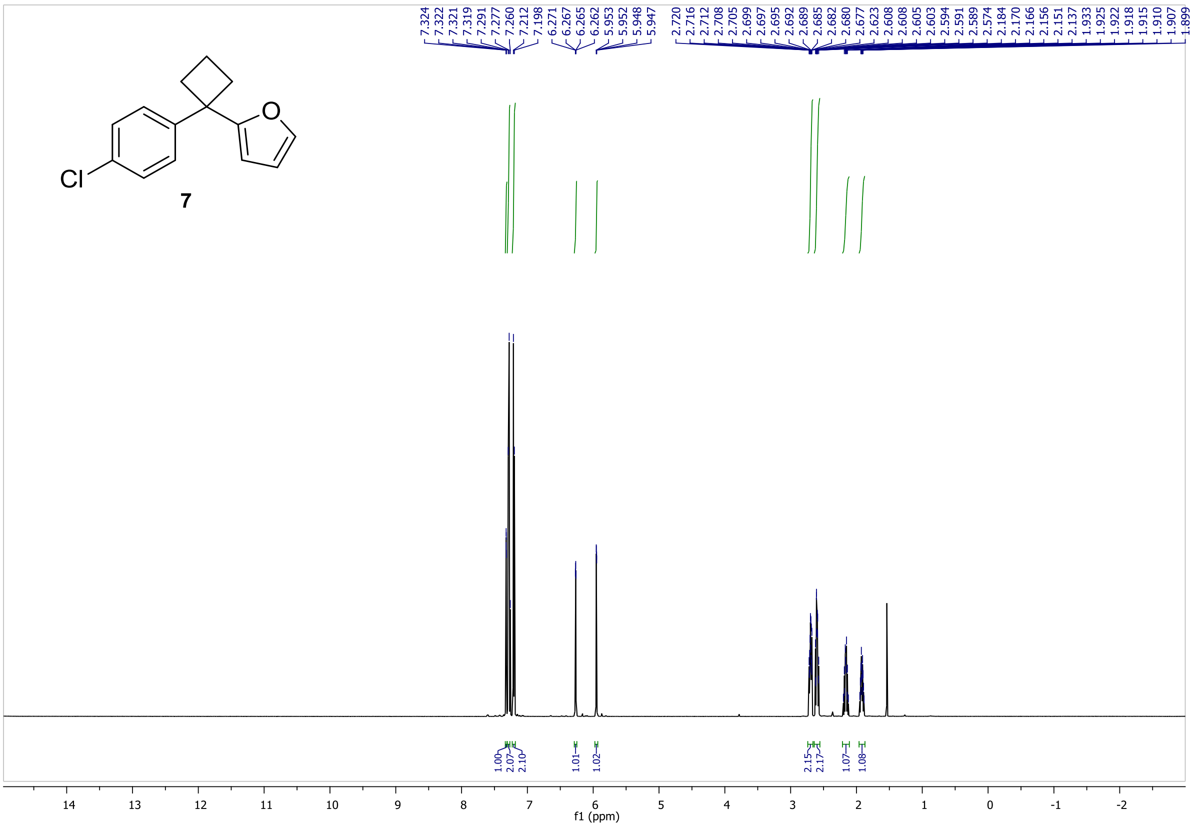

^{13}C NMR, 150 MHz, $CDCl_3$:

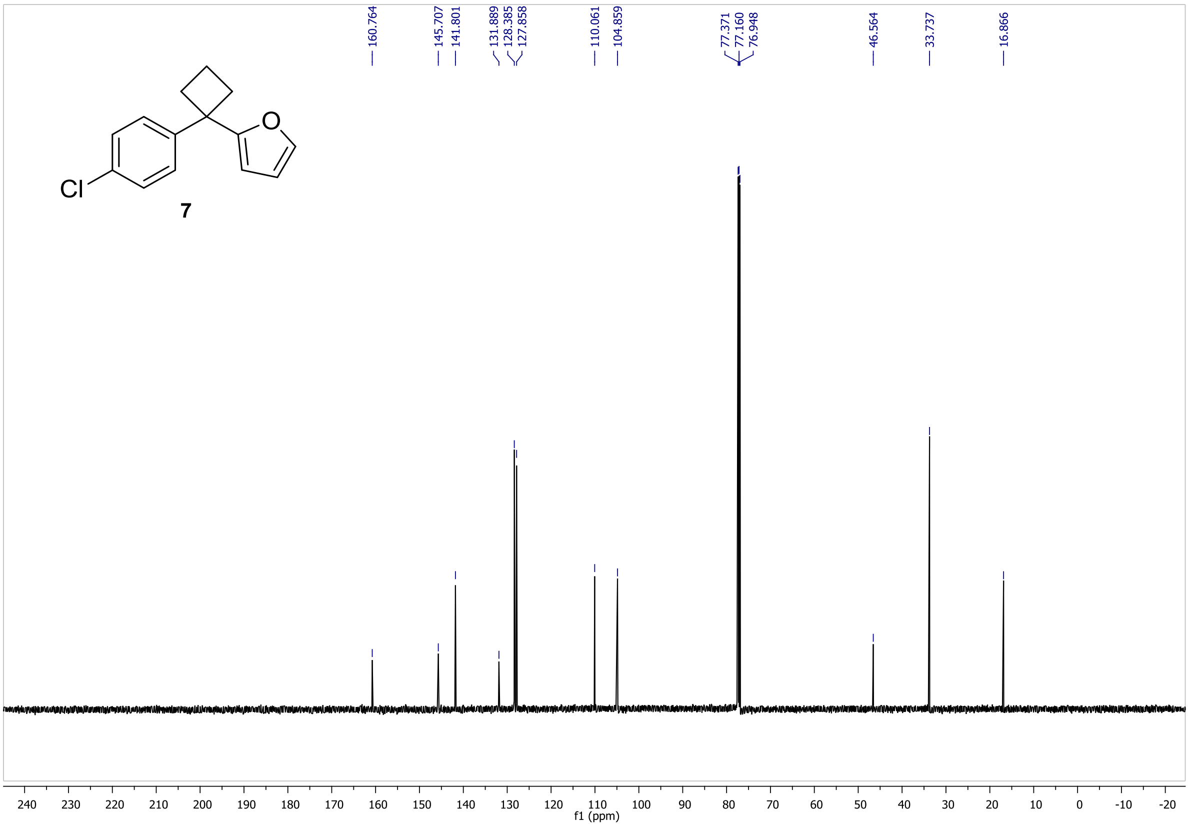

1-(1-(4-chlorophenyl)cyclobutyl)isoquinoline (8):

^{1}H NMR, 600 MHz, $CDCl_3$:

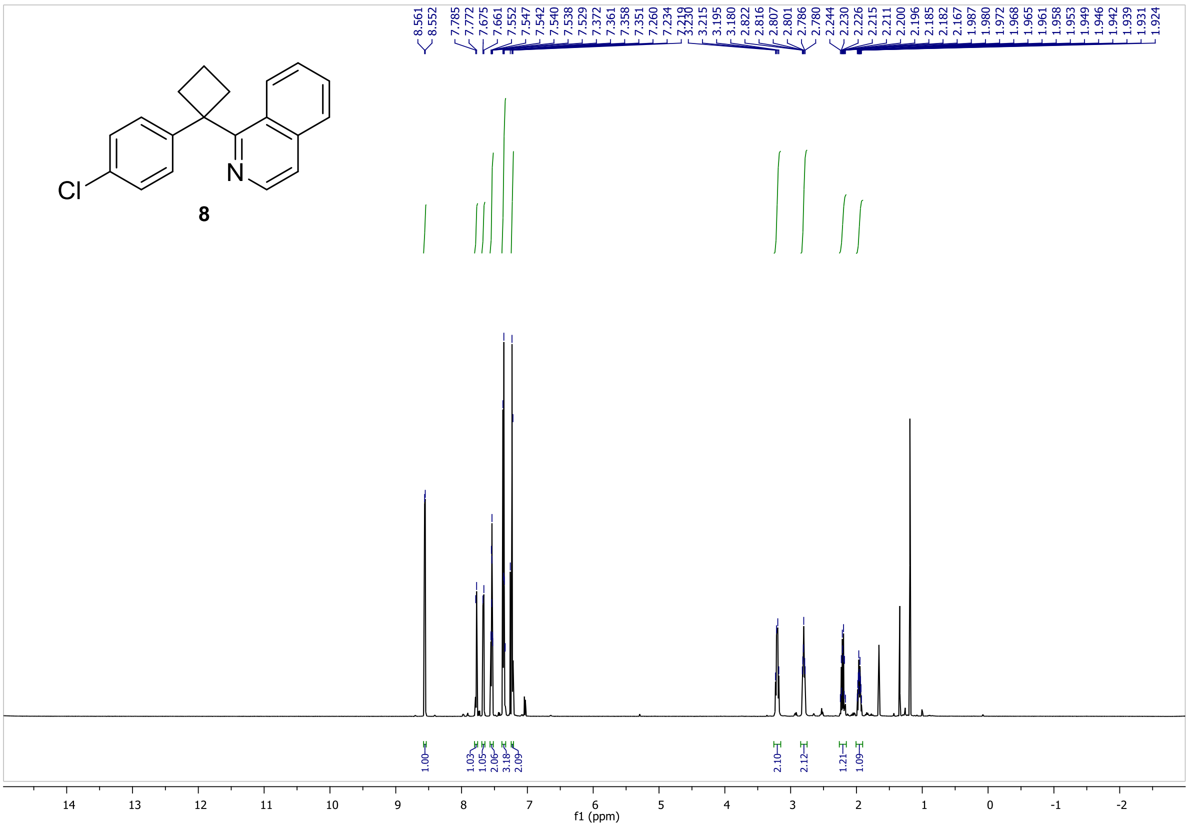

^{13}C NMR, 150 MHz, $CDCl_3$:

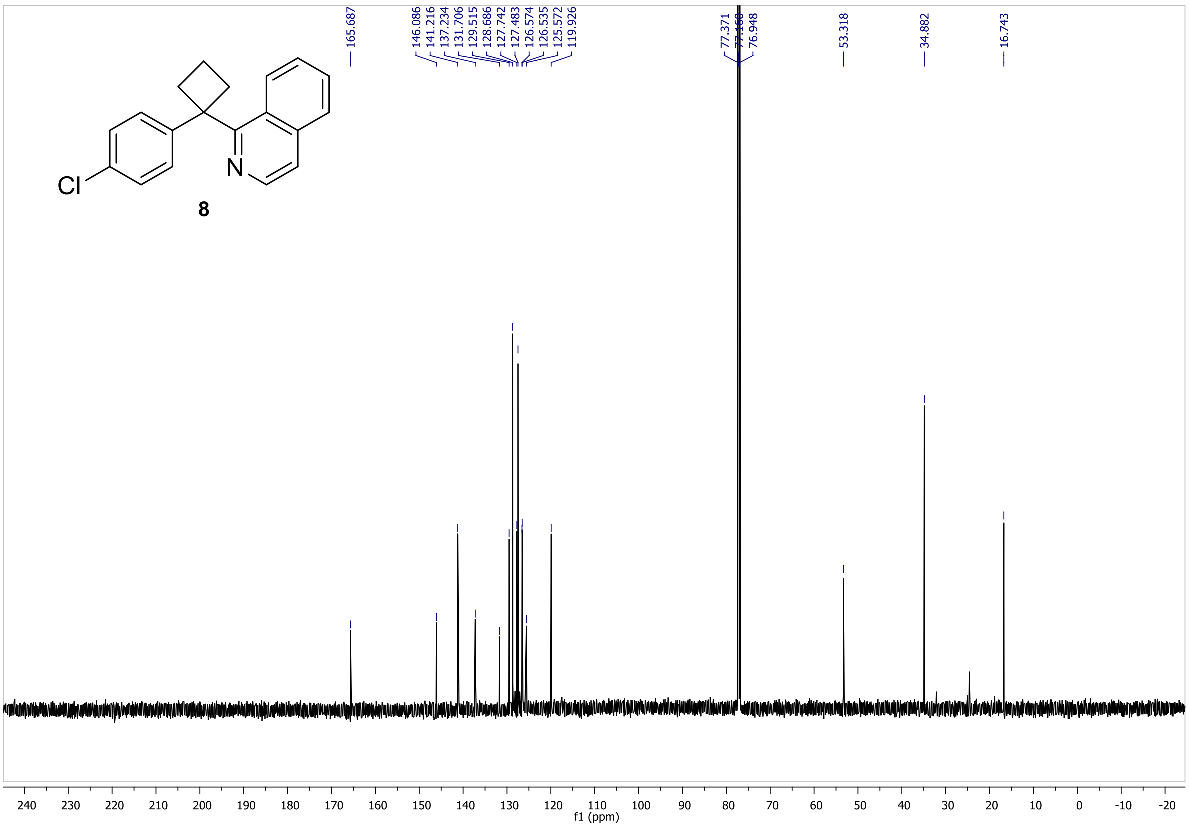

8.5. NMR spectra for (±)-baclofen synthesis

tert-butyl 4-(4-chlorophenyl)-2-oxopyrrolidine-1-carboxylate (9):

^{1}H NMR, 600 MHz, $CDCl_3$:

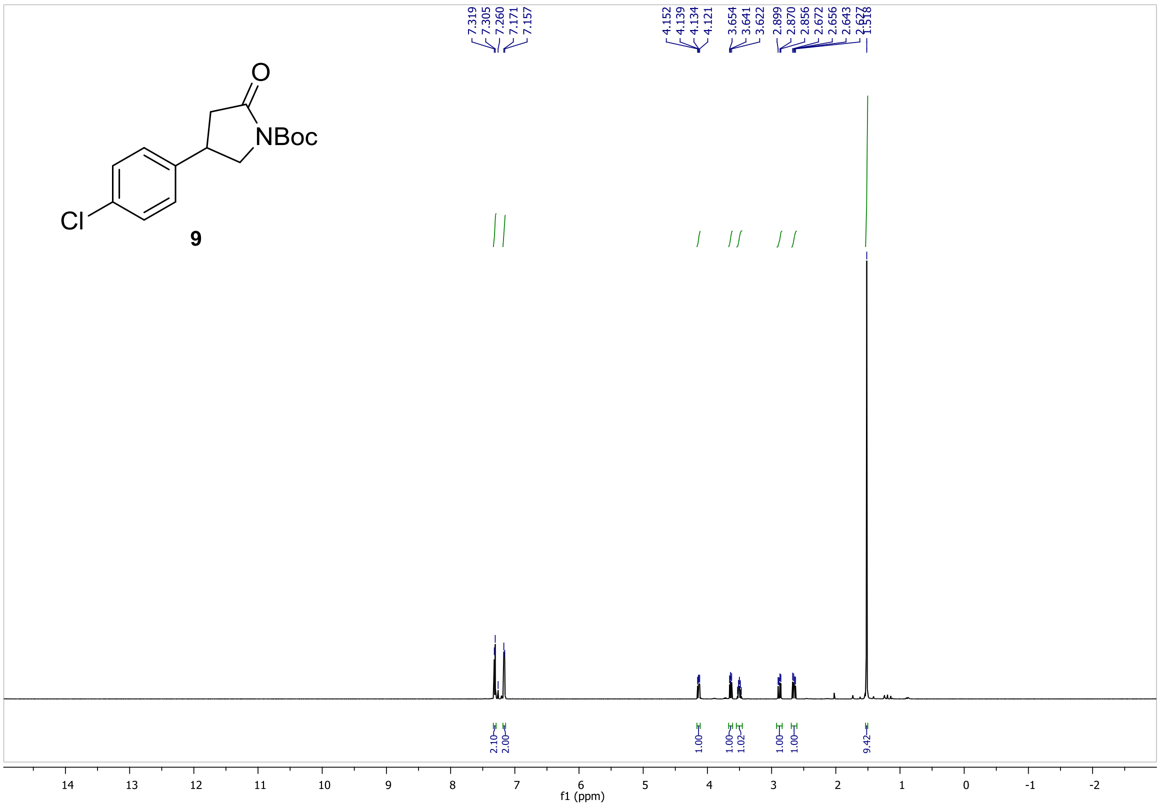

^{13}C NMR, 150 MHz, $CDCl_3$:

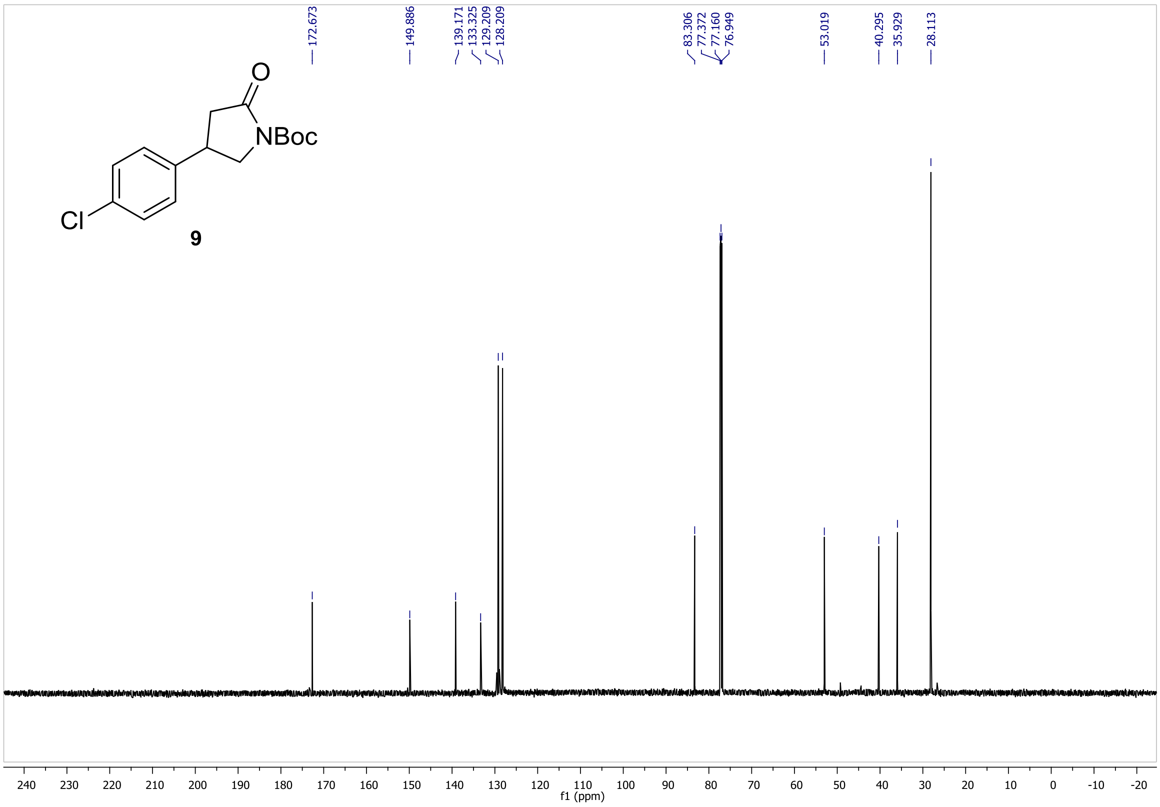

***tert*-butyl 3-(4-chlorophenyl)-2-oxopyrrolidine-1-carboxylate:**

^{1}H NMR, 600 MHz, $CDCl_3$:

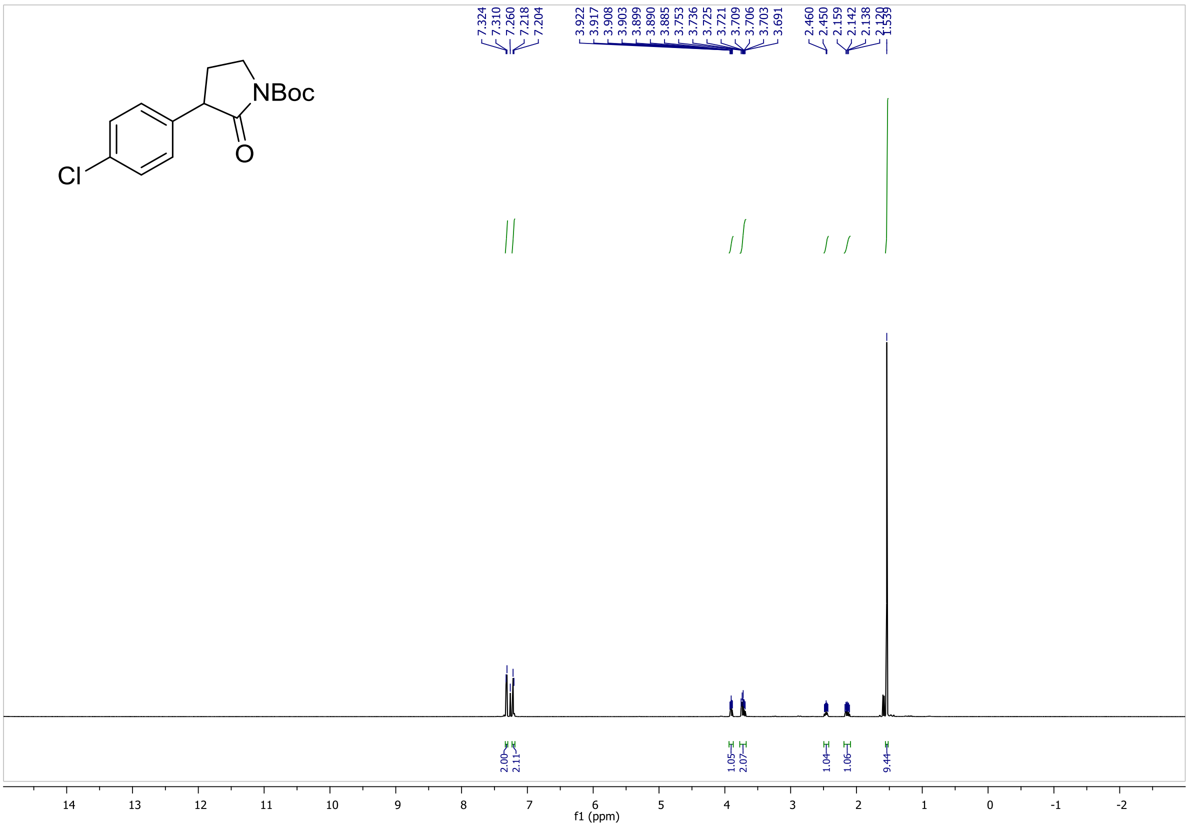

^{13}C NMR, 150 MHz, $CDCl_3$:

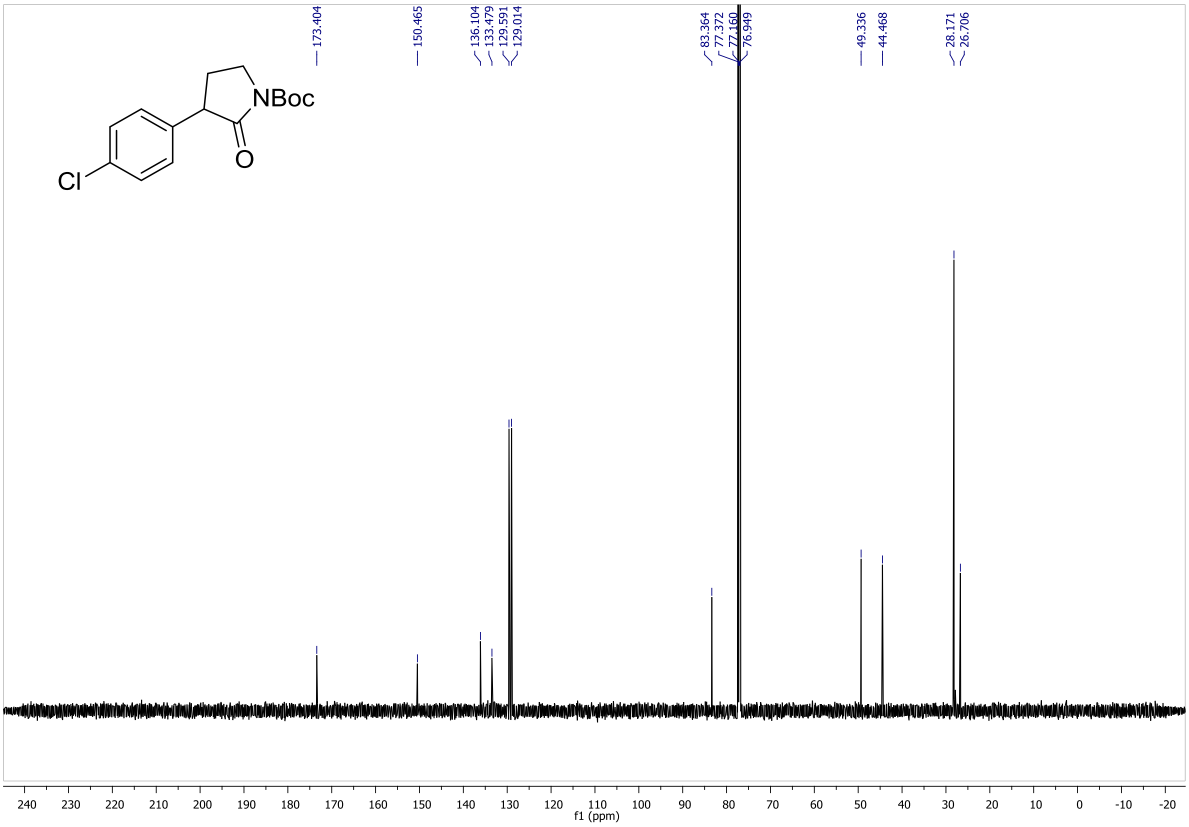

(±)-baclofen (10):

^{1}H NMR, 600 MHz, D_2O:

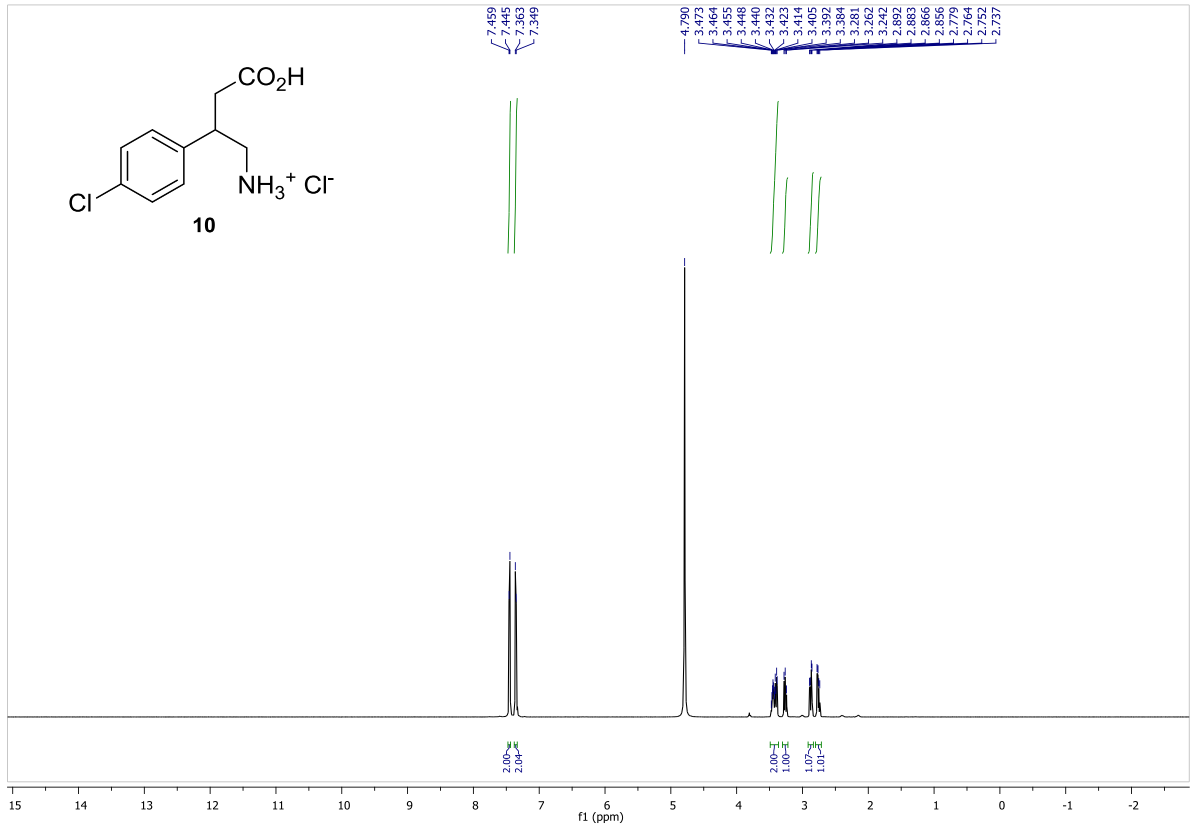

^{13}C NMR, 150 MHz, D_2O:

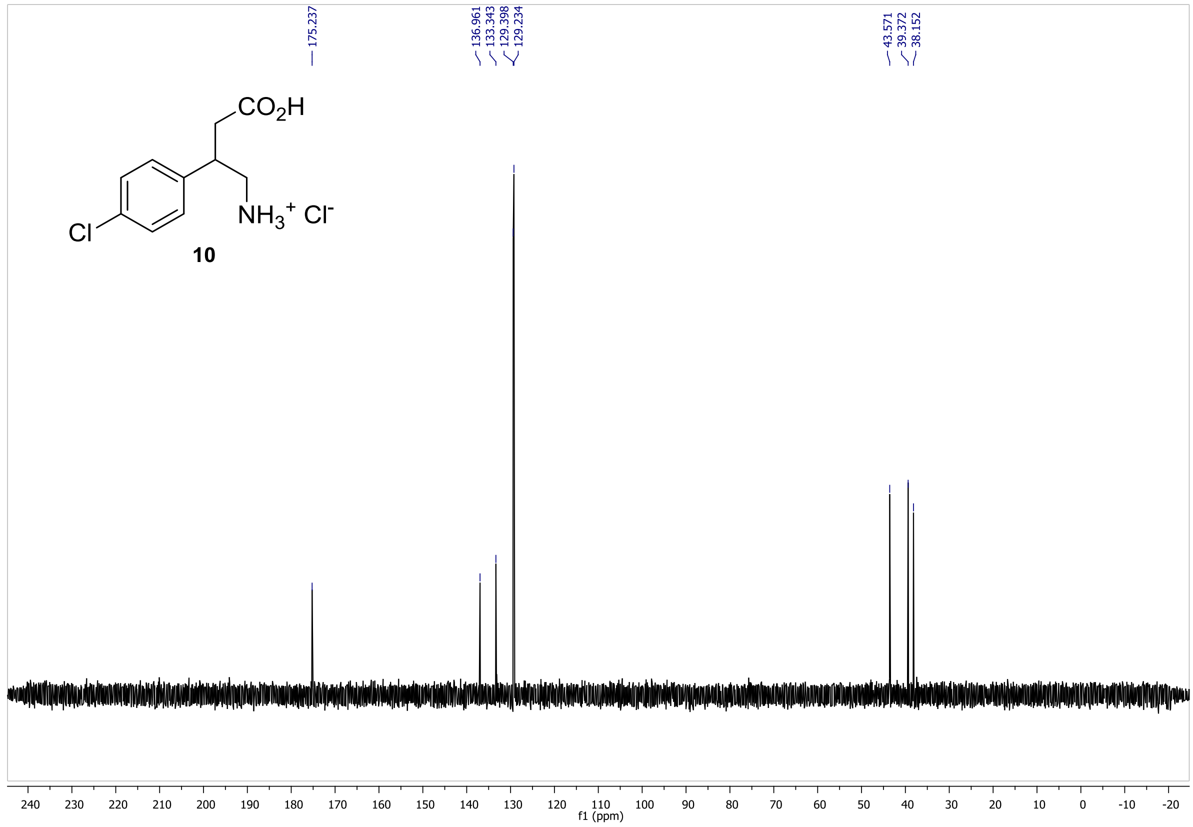

9. Differential scanning calorimetry curves

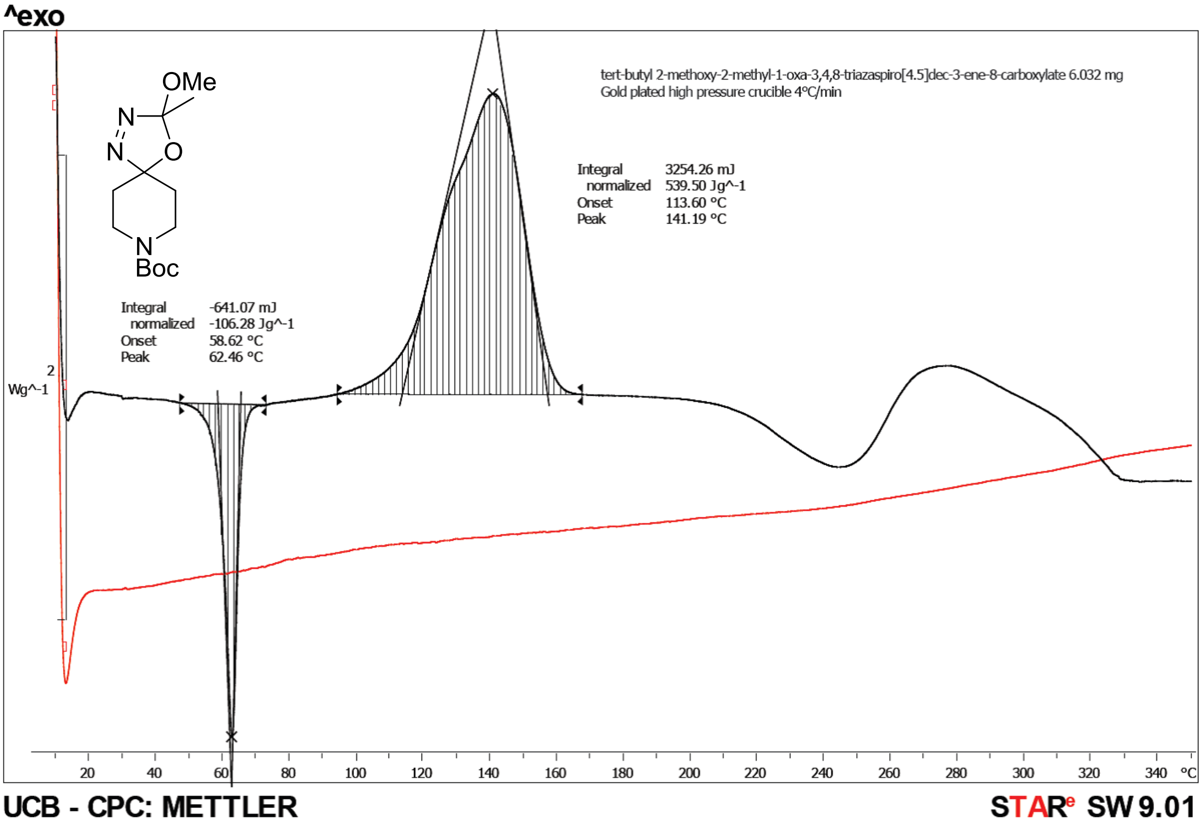

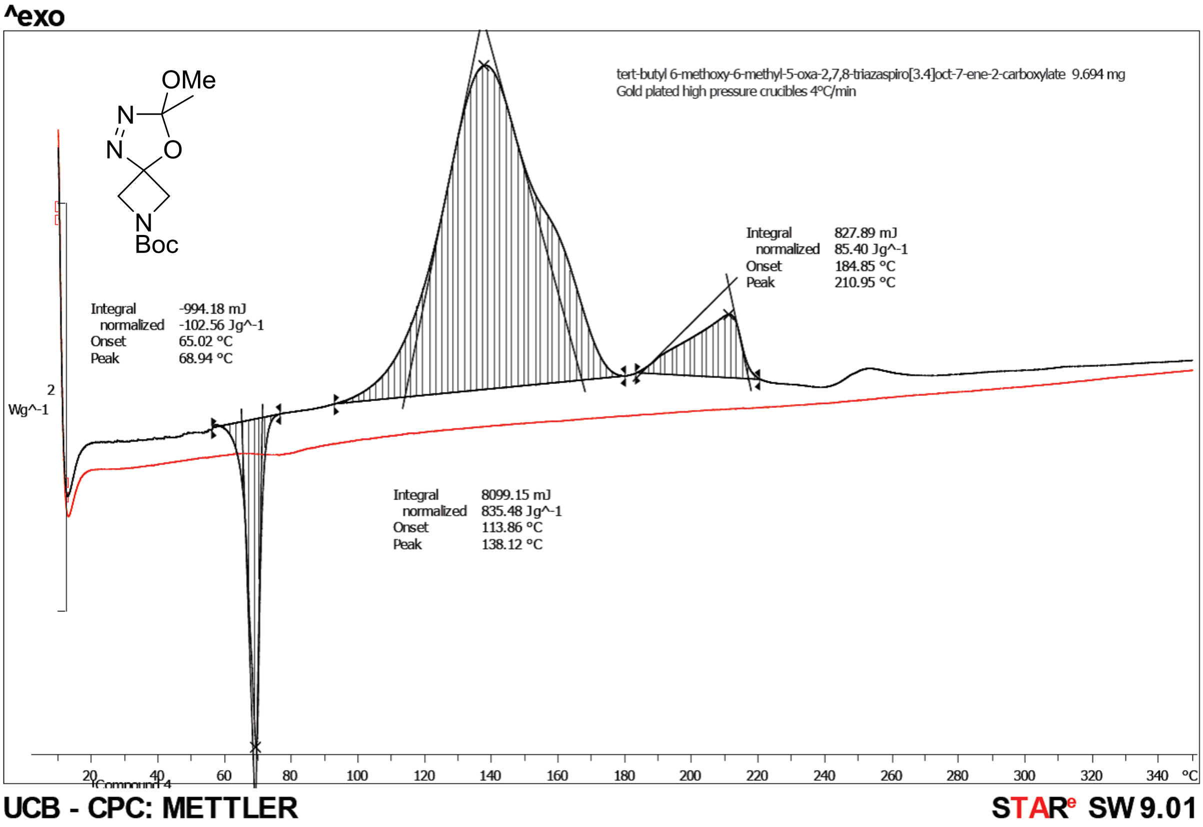

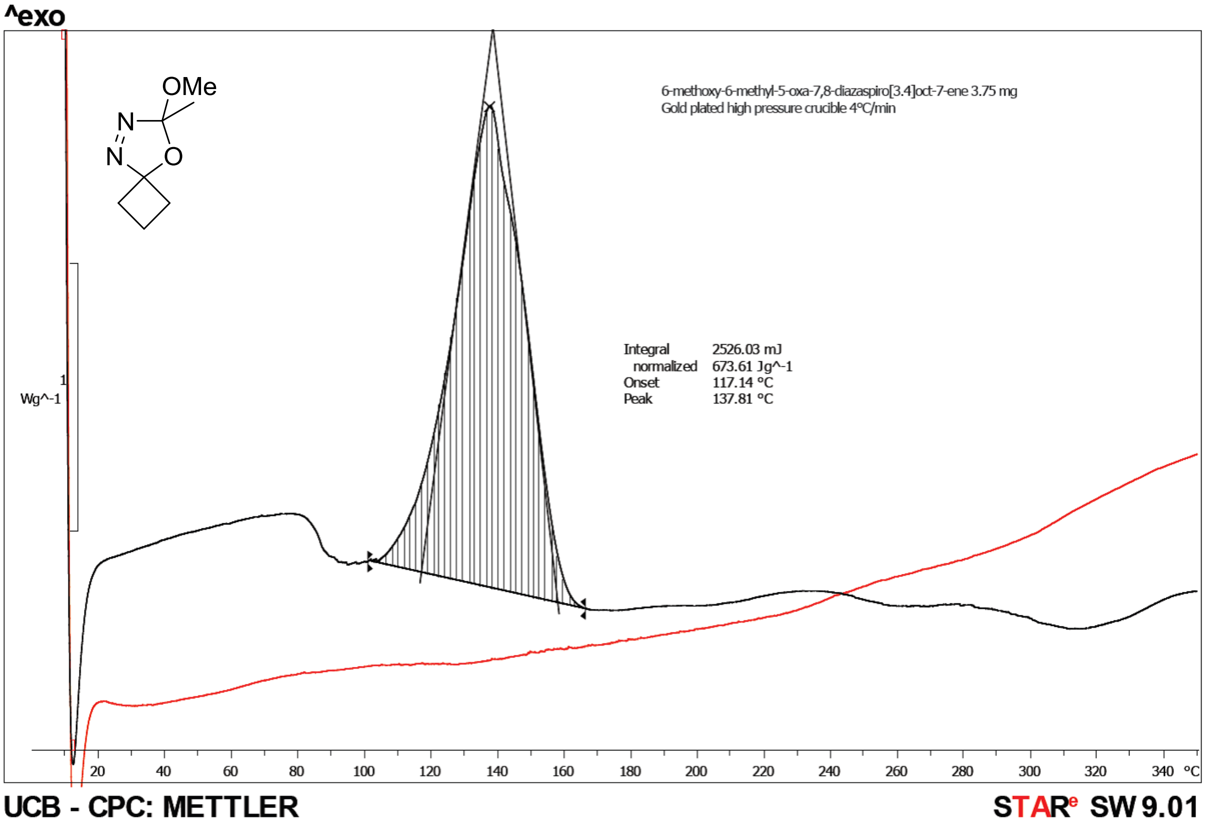
^exo
OMe
N
N
O
6-methoxy-6-methyl-5-oxa-7,8-diazaspiro[3.4]oct-7-ene 3.75 mg
Gold plated high pressure crucible 4°C/min
Integral 2526.03 mJ
normalized 673.61 Jg^-1
Onset 117.14 °C
Peak 137.81 °C
1
Wg^-1
20 40 60 80 100 120 140 160 180 200 220 240 260 280 300 320 340 °C
UCB - CPC: METTLER
STARe SW 9.01

References

1. https://www.vapourtec.com/
2. M. W. Majchrzak, M. Bekhazi, I. Tse-Sheepy and J. Warkentin, *J. Org. Chem.* **1989**, *54*, 1842-1845.
3. A. S. K. Hashmi, M. A. Grundl, A. R. Nass, F. Naumann, J. W. Bats and M. Bolte, *Eur. J. Org. Chem.* **2001**, 4705-4732.
4. G. Revial, I. Jabin and M. Pfau, *Tetrahedron: Asymmetry* **2000**, *11*, 4975-4983.
5. Y. Gaoni, *Tetrahedron* **1972**, *28*, 5525-5531.
6. L. Pehlivan, E. Métay, D. Delbrayelle, G. Mignani and M. Lemaire, *Eur. J. Org. Chem.* **2012**, 4689-4693.
7. C. R. Johnson and D. McCants Jr., *J. Am. Chem. Soc.* **1965**, *87*, 5404-5409.
8. D. M. Allwood, D. C. Blakemore, A. D. Brown and S. V. Ley, *J. Org. Chem.* **2014**, *79*, 328-338.
9. S. Yoshifuji and M. Kaname, *Chem. Pharm. Bull.* **1995**, *43*, 1302-1306.
10. K. Ueda, H. Umihara, S. Yokoshima and T. Fukuyama, *Org. Lett.* **2015**, *17*, 3191-3193.
11. D. A. Powell, T. Maki and G. C. Fu, *J. Am. Chem. Soc.* **2005**, *127*, 510-511.
12. T. Konno, K. Shimizu, K. Ogata and S. Fukuzawa, *J. Org. Chem.* **2012**, *77*, 3318-3324.
13. J. Wang, T. Qin, T.-G. Chen, L. Wimmer, J. T. Edwards, J. Cornella, B. Vokits, S. A. Shaw and P. S. Baran, *Angew. Chem. Int. Ed.* **2016**, *55*, 9676-9679.
14. P. Zhang, C. Le and D. W. C. MacMillan, *J. Am. Chem. Soc.* **2016**, *138*, 8084-8087.
15. D. N. Primer, I. Karakaya, J. C. Tellis and G. A. Molander, *J. Am. Chem. Soc.* **2015**, *137*, 2195-2198.
16. K. Tran, K. D. Berlin, E. M. Holt, R. Hallford, M. A. Eastman, V. K. Yu and K. D. Praliev, *Phosphorus, Sulfur, and Silicon* **2005**, *180*, 53-66.
17. A. Thaxton, S. Izenwasser, D. Wade, E. D. Stevens, D. L. Mobley, V. Jaber, S. A. Lomenzo and M. L. Trudell, *Bioorg. Med. Chem. Lett.* **2013**, *23*, 4404-4407.
18. Y. Wen, G. Chen, S. Huang, Y. Tang, J. Yang and Y. Zhang, *Adv. Synth. Catal.* **2016**, *358*, 947-957.
19. E. Riva, S. Gagliardi, M. Martinelli, D. Passarella, D. Vigo and A. Rencurosi, *Tetrahedron* **2010**, *66*, 3242-3247.
20. H. Fujioka, H. Komatsu, A. Miyoshi, K. Murai and Y. Kita, *Tetrahedron Lett.* **2011**, *52*, 973-975.
21. C. Singh, S. Pandey, G. Saxena, N. Srivastava and M. Sharma, *J. Org. Chem.* **2006**, *71*, 9057-9061.
22. D. R. Williams and M. P. Clark, *Tetrahedron Lett.* **1998**, *39*, 7629-7632.
23. B. N. Roy, G. P. Singh, P. S. Lathi, M. K. Agrawal, R. Mitra and A. Trivedi, Lupin Limited, Patent WO 2012/153162 A1, 15 November 2012.
24. M. A. Brodney, Pfizer Products Inc., Patent WO 2006/106416 A1, 12 October 2006.
25. A. Berger and K. D. Wehrstedt, *J. Loss Prevent. Proc.* **2010**, *23*, 734-739.
